# A Mechanochemical Kolbe–Schmitt Reaction: Catechol Carboxylation Provides Building Blocks for Renewable Plasticizers

**DOI:** 10.1002/anie.202519827

**Published:** 2026-02-09

**Authors:** Dries De Vos, Victoria S. Pfennig, Arno Goddé, Robby Vroemans, Tobias Krückel, Nicole Marcinkowska, Ettore Bartalucci, Thomas Wiegand, Carsten Bolm, Bert U. W. Maes

**Affiliations:** ^1^ Division of Organic Synthesis Department of Chemistry University of Antwerp Antwerp Belgium; ^2^ Institute of Organic Chemistry RWTH Aachen University Aachen Germany; ^3^ Max Planck Institute For Chemical Energy Conversion Mülheim a. d. Ruhr Germany; ^4^ Institute of Technical and Macromolecular Chemistry RWTH Aachen University Aachen Germany

**Keywords:** carboxylation, catechol, Kolbe–Schmitt, mechanochemistry, plasticizer

## Abstract

Catechol, an important aromatic platform molecule which can be derived from biomass, was carboxylated by mechanochemical Kolbe–Schmitt reaction of disodium catecholate with CO_2_, providing a mixture of mono‐ and dicarboxylated catechol derivatives. While classical protocols require harsh reaction conditions, involving a high temperature and/or CO_2_ pressure, a mild ball milling method was developed. This represents the first mechanochemical Kolbe–Schmitt reaction featuring a low CO_2_ pressure and reactivity at room temperature. From the individual catechol‐based mono‐ and dicarboxylic acid reaction products, a library of novel renewable plasticizers was synthesized through esterification of the carboxylic acid functionalities and *O*‐acylation of the phenolic hydroxy groups. The resulting esters were evaluated in poly(vinylchloride) (PVC) and poly(lactic acid) (PLA), revealing plasticizing efficiencies competitive to benchmark commercial plasticizers. These efficiencies were maintained when the best performing ester substitution pattern was installed on the ball mill‐derived mixture of mono‐ and dicarboxylated catechols, making resource intensive separation (e.g. chromatographic separation) of these *ortho*‐dihydroxybenzene(di)carboxylic acids redundant.

## Graphical abstract

A mechanochemical Kolbe–Schmitt reaction of disodium catecholate enables the synthesis of mono‐ and dicarboxylated catechols at low temperature and CO_2_ pressure. Directly derivatizing the resulting carboxylic acid and phenolic moieties in the obtained mixture provides plasticizer blends with efficiencies competitive to commercial benchmarks.

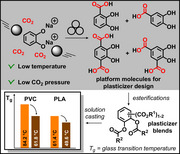

## 1 Introduction

**FIGURE 1:**
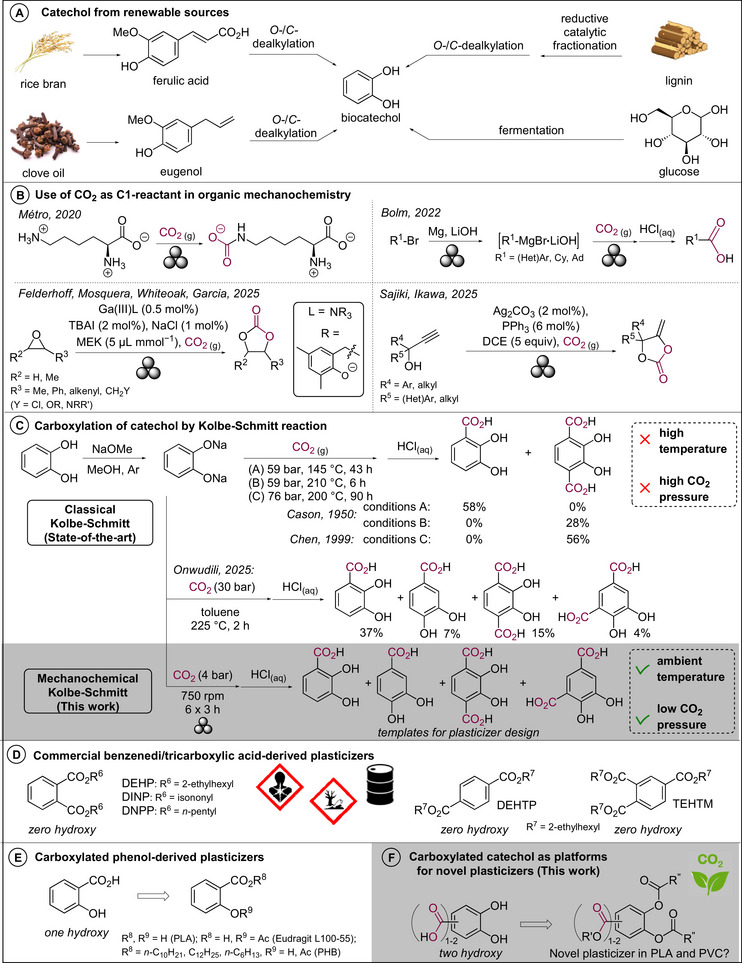
State‐of‐the‐art and aim of the work.

With an annual production of 40 kt, catechol is an important commodity chemical prepared from phenol derived from crude oil [1]. In recent years, it was shown that it is accessible from platform molecules obtained in biorefineries using diverse non‐edible biomass. Notably, 4‐propylguaiacol derived from softwood by reductive catalytic fractionation [2], eugenol obtained from clove oil by steam distillation and extraction [3], and ferulic acid from rice bran oil by saponification and extraction [4], can each be *O*‐ and *C*‐defunctionalized to obtain biocatechol (Figure 1A) [1, 5, 6, 7, 8, 9]. Alternatively, fermentation of sugars toward catechol has also been explored [10, 11].

Given the need to replace fossil carbon by renewable carbon in chemicals to combat global warming, procedures to transform catechol into drop‐in and new chemicals is a contemporary field of research. An interesting transformation is its carboxylation with the greenhouse gas CO_2_ as a C1‐reactant, thereby allowing to generate dihydroxybenzoic acids with 100% renewable carbon featuring handles for further derivatization [12, 13, 14, 15]. The carboxylation of such phenolic compounds using CO_2_ can be achieved through the Kolbe–Schmitt reaction [16, 17]. Although several modifications of this reaction have been developed, generally, these require high temperatures (up to 225°C) and/or CO_2_ pressures (up to 100 bar) (Figure 1C) [18, 19, 20]. Recently, the solvent‐based classical Kolbe–Schmitt reaction on catechol was revised and shown to form a mixture of four distinct mono‐ and dicarboxylated catechols in contrast to the original reports [20]. Attempts to use milder carboxylations at lower temperature only alleviate the CO_2_ pressure requirements or are limited to a specific substrate, excluding catechol [21, 22, 23]. To counteract these restrictions, we envisioned exploring mechanochemistry for the reaction, an emerging field in which ball milling devices among others are used to apply mechanical energy to chemical reactions [24, 25, 26, 27, 28, 29]. Mechanochemical activation enables reactions to occur in the solid state without a liquid medium and allows for reactivity at decreased bulk temperatures. Recently, the use of gaseous reactants and solid gas surrogates in ball mills has increased steadily, including CO_2_ [30, 31, 32, 33, 34, 35, 36]. However, the use of gaseous CO_2_ as C1‐reactant in synthetic organic mechanochemistry has only received limited attention (Figure 1B) [37, 38]. Métro and coworkers observed the formation of l‐lysine ammonium carbamate when milling l‐lysine in the presence of gaseous CO_2_ [39]. Bolm and coworkers reported a one‐pot three step mechanochemical synthesis of carboxylic acids starting from organobromides through the combination of an in situ formed Grignard reagent and CO_2_ gas [40]. This method has recently been extended to a one‐step procedure in an electromagnetic mill [41]. Recently, Felderhoff, Mosquera, Whiteoak, Garcia, and coworkers developed a Ga(III)‐catalyzed mechanochemical synthesis of cyclic carbonates from CO_2_ gas and epoxides [42]. Sajiki, Ikawa, and coworker likewise obtained cyclic carbonates from propargyl alcohols and CO_2_ gas using Ag(I)‐catalysis [43]. Given only such few examples of organic mechanosynthesis with gaseous CO_2_ are known and the Kolbe–Schmitt reaction requires harsh conditions, the development of a mild mechanochemical Kolbe–Schmitt reaction on catechol providing *ortho*‐dihydroxybenzene(di)carboxylic acids is appealing, as these are interesting templates for the development of novel renewable plasticizers.

Plasticizers are added to rigid and brittle polymer materials to increase flexibility and ease polymer processing [44, 45], and are the most used additives (both by value and by volume) in plastics worldwide with a total market share of $19.8 billion in 2024 and a forecasted value of $28.0 billion by 2030 [46]. These are performance chemicals which can be individual compounds or mixtures. PVC is the third most produced polymer globally and approximately 80%‒90% of the total amount of plasticizers produced is eventually blended with PVC [47, 48], while PLA is the market leader in the field of biobased plastics [49, 50]. Despite their advantages, both PVC and PLA are known to be rigid and brittle polymers. As a result, PVC and PLA are thoroughly researched polymers in the plasticizer field. Aromatic di/triesters such as phthalates, terephthalates, and trimellitates (Figure 1D), despite being commonly used and petroleum oil derived, have been under heavy scrutiny based on their human and ecotoxicity issues (e.g. endocrine disruption, bioaccumulation, carcinogenic, mutagenic, and reprotoxic) [51, 52]. Hence, structurally different aromatic compounds easily derived from biomass are required. Considering esters of the phenolic hydroxy and carboxylate groups of salicylic acid are known as plasticizers [53, 54, 55] (Figure 1E), the introduction of an additional hydroxy group for esterification, that is *ortho*‐dihydroxybenzenecarboxylic acid, is expected to be beneficial (Figure 1F). Also, when an additional second carboxylic acid, that is *ortho*‐dihydroxybenzenedicarboxylic acids, can be introduced in the Kolbe–Schmitt reaction, it offers additional handles for further introduction of ester moieties. Hence, a mild mechanochemical Kolbe–Schmitt reaction of disodium catecholate was envisioned to access these platform molecules (Figure 1C).

## 2 Results and Discussion

### 2.1 Mechanochemical Kolbe–Schmitt Carboxylation of Catechol

The reactions were conducted in a planetary mill in ZrO_2_‐M milling vessels with gas valves using milling balls of the same material. The vessels loaded with the reaction mixture and grinding balls were sealed, pressurized with 4 bar of gaseous CO_2_ and subjected to the milling procedure. Based on the classical Kolbe–Schmitt reaction (Figure 1C) the doubly deprotonated disodium catecholate (**1‐Na_2_**) was selected as starting material. This disodium salt was prepared in quantitative yield from catechol using sodium methanolate (NaOMe) as a base in methanol to allow an easy removal of the conjugated acid byproduct MeOH by simple evaporation [18, 19, 20]. An initial experiment using disodium catecholate (**1‐Na_2_**) with 4 bar of CO_2_ pressure yielded a small amount of 2,3‐dihydroxybenzoic acid (**2a**) after 4 h reaction time and aqueous, acidic work‐up (Table 1, entry 1). With extended milling time to a total of 12 h (entry 2), the conversion drastically increased, providing a mixture of **2a**, 3,4‐dihydroxybenzoic acid (**2b**), 2,3‐terephthalic acid (**2c**), and 4,5‐dihydroxyisoterephthalic acid (**2d**). The total yield of mono and dicarboxylated catechols was 63% with 17% of unreacted catechol (**1**) remaining.

**TABLE 1:** Optimization of the Kolbe–Schmitt mechanochemical carboxylation of disodium catecholate (**1‐Na_2_**)^a^.

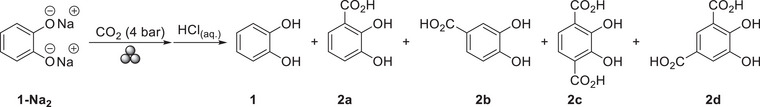								
	Grinding conditions		Yield^b^					
Entry	*F* [rpm]	*T* [min]	1 [%]	2a [%]	2b [%]	2c [%]	2d [%]	Σ(2a‐d) [%]
1	800	240	n. d.^c^	3	—	—	—	—
2	800	3×240	17	26	18	13	6	63
3^d^	800	3×240	13	26	21	11	11	69
4	800	6×180	20	32	23	9	5	69
5	850	6×180	20	24	18	5	5	52
6^e^	850	6×180	11	22	22	8	12	64
7^f^	850	6×180	8	7	7	1	3	18
8	750	6×180	19	33	26	13	10	82
9	700	6×180	37	33	20	7	4	64
10	400	6×180	62	10	7	1	Traces	18

^a^ Reaction conditions: **1‐Na_2_** was weighed into ZrO_2_‐M milling vessels with gas valves and 5 ZrO_2_‐M balls (*Ø*  =  10 mm) inside a glovebox and the vessels were sealed, 4 bar CO_2_ were added outside the glovebox and the mixture was milled at the indicated rotational frequency for the indicated time. In the case of multiple cycles, 30 min breaks were taken after each cycle, with a renewal of the CO_2_ after the first cycle, followed by aqueous work‐up with HCl as described in the supplementary information.

^b^ Identification and quantification by ^1^H NMR spectroscopy with 1,3,5‐trimethoxybenzene (TMB) as internal standard.

^c^ Not determined

^d^ Instead of 4 bar, the vessel was filled with 8 bar CO_2_‐pressure.

^e^ 10 ZrO_2_‐M balls (*Ø*  =  10 mm) were used instead of 5.

^f^ Instead of 5 ZrO_2_‐M balls (*Ø*  =  10 mm), 80 ZrO_2_‐Y balls (*Ø*  =  5 mm) were used. After the grinding steps, the reaction mixture was a black, soot‐like powder.

While an increase in CO_2_‐pressure to 8 bar inside the vessel barely improved the conversion and individual yields (entry 3), the extension of the milling time to a total of 18 h favored higher yields for the monocarboxylation products **2a** and **2b** (entry 4). Attempts to increase the energy input by using a milling frequency to 850 rpm (entry 5) and additionally increasing the number of balls (entry 6) did not further improve the conversion of **1‐Na_2_** to the desired carboxylic acids **2a‐d**. Moreover, replacing the increased number of grinding balls with smaller balls amounting to the same weight proved detrimental for the mass balance (entry 7). Gratifyingly, at a decreased milling speed of 750 rpm an 82% yield of carboxylated catechol products **2a‐d** was observed (entry 8). This counterintuitive result, where lowering milling speed results in better performance, is a known phenomenon in mechanochemical literature, where higher energy input does not universally lead to better performance [56]. Excessive impact energy may reduce mixing efficiency or alter the impact regime. Furthermore, planetary ball milling is well documented to generate significant heat at higher rotational frequencies [57]. When comparing the results of our Kolbe–Schmitt reaction at different rotational speeds [i.e. the reaction at 750 rpm (entry 8), 800 rpm (entry 4), and 850 rpm (entry 5)] the remaining amount of catechol (**1**) is always 19%–20%. Nevertheless, the combined yield of products **2a**‐**2d** is 82%, 69%, and 52%, respectively. This indicates that the partial degradation of the reaction products under the milling conditions is also a result of more energy input. With rotational speeds below 750 rpm, such as 700 rpm (entry 9) and 400 rpm (entry 10), we observe a larger amount of catechol (**1**) remaining (resp. 37% and 62%), concomitantly with lower combined yields of carboxylated products (resp. 64% and 18%).

**FIGURE 2:**
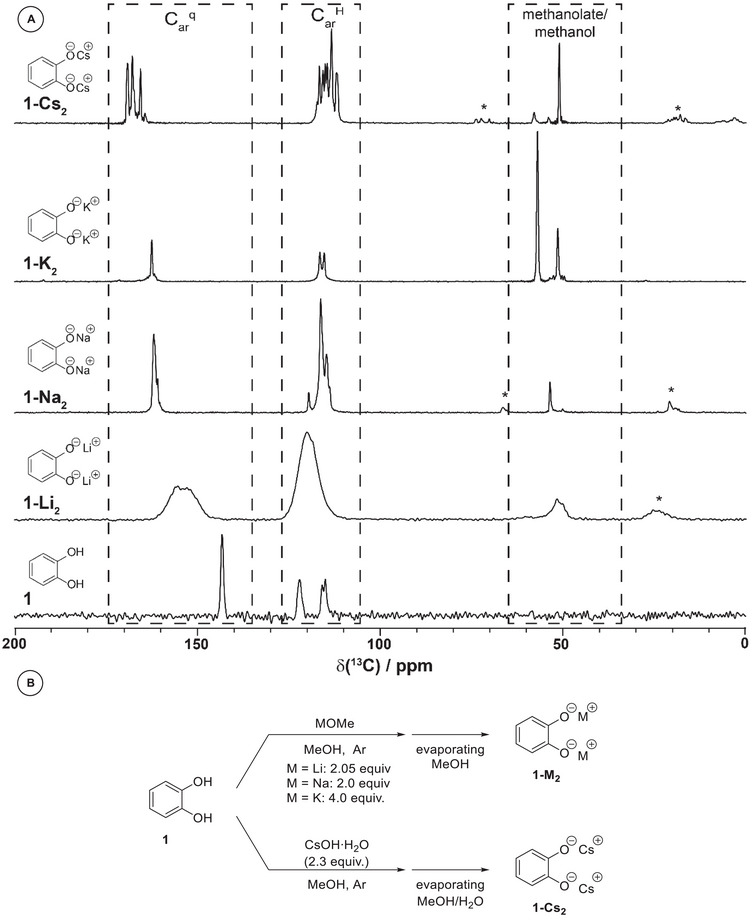
(A) Comparison of the ^1^H‐^13^C CP‐MAS spectra of catechol (**1**, bottom) and the prepared dialkali metal catecholates, (**1‐Li_2_**, **1‐Na_2_**, **1–K_2_**, **1‐Cs_2_**), * indicate MAS sidebands. Resonance assignments are given in the Figure. All spectra have been recorded at a static magnetic‐field strength of 11.7 T and MAS frequencies ranging in‐between 12 to 20 kHz. (B) Synthetic procedure for the synthesis of **1‐M_2_**.

Next, we investigated the influence of the alkali metal cation (Cs, K, Na, Li) on the selectivity of the mechanochemical carboxylation. Hence, the corresponding dialkali metal catecholates **1‐M_2_** were synthesized (see Figure 2B) and tested under the optimum milling conditions (Table 2). While with **1‐Cs_2_** and **1‐K_2_** only traces of the desired carboxylic acids **2a‐d** were obtained, **1‐Li_2_** gave 66% overall yield of carboxylated catechol products **2a‐d** (Table 2, entries 1, 2, and 4) [58]. This was lower than obtained with **1‐Na_2_**. Sodium was therefore the preferred cation (entry 3). Next, we studied whether monoalkali metal catecholate (**1‐M_1_**) would be sufficient to activate the aromatic compound for carboxylation. To investigate this hypothesis, monosodium catecholate (**1‐Na_1_**) was synthesized [59] and compared to the performance of **1‐Na_2_** under mechanochemical carboxylation conditions (Table 2, entries 5 and 6). However, the ^1^H NMR spectrum of the crude product showed only catechol (**1**). The additional proton completely blocked the reaction, indicating that acidic protons are not tolerated.

**TABLE 2:** Effect of alkali metal on the Kolbe–Schmitt mechanochemical carboxylation reaction of dialkali metal catecholates (**1‐M_2_**)^a^.

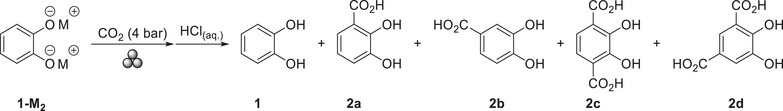								
			Yield^b^					
Entry	1‐M_2_	*n*(1‐M_2_)^c^ [mmol]	1 [%]	2a [%]	2b [%]	2c [%]	2d [%]	Σ(2a‐d) [%]
1	**1‐Cs_2_**	1.0	99	Traces	Traces	Traces	Traces	Traces
2	**1‐K_2_**	1.5	99	Traces	Traces	Traces	Traces	Traces
3	**1‐Na_2_**	1.5	19	33	26	13	10	82
4^d^	**1‐Li_2_**	2.0	27	37	19	6	4	66
5^e^	**1‐Na_1_** ^f^	1.5	94	0	0	0	0	0
6^e^	**1‐Na_2_**	1.5	37	33	20	7	4	64

^a^ Reaction conditions: **1‐M_2_** was weighed into ZrO_2_‐M milling vessels with gas valves and 5 ZrO_2_‐M balls (*Ø*  =  10 mm) inside a glovebox and the vessels were sealed, 4 bar CO_2_ were added outside the glovebox and the mixture was milled at 750 rpm for 6×180 min with 30 min break after each cycle and with a renewal of the CO_2_ pressure after the first 180 min, followed by aqueous work‐up with HCl as described in the Supporting Information.

^b^ Identification and quantification by ^1^H NMR spectroscopy with 1,3,5‐trimethoxybenzene (TMB) as internal standard.

^c^ The amount of substance had to be adjusted to ensure that approximately the same masses were present in the milling vessel for optimal mixing of the different salts.

^d^ Average over three reactions.

^e^ The reaction was milled at 700 rpm for 6 x 180 min with 30 min break after each cycle and a renewal of the CO_2_ pressure after the first 180 min.

^f^ **1‐Na_1_** = monosodium catecholate.

Remarkably, only CO_2_ (gas) was a suitable reactant, as the addition of sodium methyl carbonate (NaOCO_2_Me) and dimethylcarbonate (DMC) as CO_2_ surrogates proved detrimental (Table 3, entry 2–3). The addition of sodium‐complexing 15‐crown‐5 inhibited the reaction completely (entry 4). The use of liquid‐assisted grinding (LAG) agents had a negative influence too (entries 5–6). Finally, the addition of bases [Na_2_CO_3_, NaOMe, 1,8‐diazabicyclo(5.4.0)undec‐7‐ene (DBU)] was studied. While NaOMe and DBU blocked the reaction, Na_2_CO_3_ gave a moderate reactivity (entries 7–9). The result with DBU is surprising as it is known for its ability to enhance CO_2_ availability from the gas phase to chemical reactions in the liquid phase [60, 61, 62]. Hence, the optimal reaction conditions involve **1‐Na_2_**, 4 bar of CO_2_ milled at 750 rpm for 6×180 min by 5 ZrO_2_‐M balls (*Ø*  =  1 cm) providing a mixture of products **2a**‐**d** (ratio of 46:31:12:11) (Table 1, entry 8).

**TABLE 3:** Effect of additives on the Kolbe–Schmitt mechanochemical carboxylation reaction of disodium catecholate (**1‐Na_2_**)^a^.

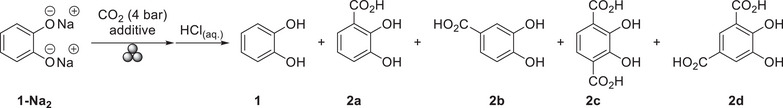									
				Yield^b^					
Entry	Additive	Additive [equiv.]	*η* [µL mg^−1^]	1 [%]	2a [%]	2b [%]	2c [%]	2d [%]	Σ(2a‐d) [%]
1	—	—	—	19	33	26	13	10	82
2	NaOCO_2_Me	1.0	—	69	19	10	2	Traces	31
3	DMC	1.0	0.55	99	—	—	—	—	—
4	15‐crown‐5	1.0	—	99	—	—	—	—	—
5	Cyrene	0.38	0.25	51	25	16	5	3	49
6	Toluene	0.36	0.25	24	33	24	11	8	76
7	Na_2_CO_3_	1.05	—	38	17	11	9	1	38
8	NaOMe	1.05	—	98	—	—	—	—	—
9	DBU^c^	3.0	2.91	81	—	—	—	—	—

^a^ Reaction conditions: **1‐Na_2_** was weighed into ZrO_2_‐M milling vessels with gas valves and 5 ZrO_2_‐M balls (*Ø*  =  10 mm) inside a glovebox and the vessels were sealed, 4 bar CO_2_ were added outside the glovebox and the mixture was milled at 750 rpm for 6×180 min with 30 min break after each cycle and with a renewal of the CO_2_ pressure after the first 180 min, followed by aqueous work‐up with HCl as described in the Supporting Information.

^b^ Identification and quantification by ^1^H NMR spectroscopy with 1,3,5‐trimethoxybenzene (TMB) as internal standard.

^c^ 1,8‐Diazabicyclo(5.4.0)undec‐7‐ene.

### 2.2 Solid‐State NMR Characterization of the Dialkali Metal Catecholate Salts (1‐M_2_)

The dialkali metal catecholates **1‐M_2_** were characterized by solid‐state NMR spectroscopy under magic‐angle spinning (MAS) conditions to explore whether their solid‐state properties reveal explanations for the observed differences in reactivity in the mechanochemical carboxylation reactions. In that vein, ^1^H–^13^C cross polarization (CP‐MAS) [63, 64, 65] spectra have shown to be a highly beneficial tool in studying mechanochemical reactions and were therefore recorded in the present case as well [66]. In general, the range of ^13^C chemical‐shift values of the catechol and dialkali metal catecholate salts observed in the spectra agree with the expectations (Table S2). The quaternary carbon nuclei shift to higher ^13^C frequencies in the series Li, Na, K, and Cs (Figure 2A), which is for instance in contrast to a previous study on *m*‐bromocinnamates for which no systematic variation in ^13^C chemical‐shift values of the carboxylate group as a function of the coordinating alkali metal ion was observed [67]. Furthermore, there is no pronounced change in the ^13^C chemical‐shift values of other aromatic signals, suggesting that they possess comparable electron density and nucleophilicity. In the aromatic region, more sets of resonances than expected are observed for the Na (**1‐Na_2_**) and Cs (**1‐Cs_2_**) dialkali metal catecholates, pointing to several crystallographically‐distinct molecules in the asymmetric unit, the presence of polymorphs or pseudo‐polymorphs (solvent molecules are incorporated in the **1‐M_2_** structures) or an incomplete formation of the dialkali catecholates (remaining alkali metal methanolate). The ^13^C resonances of **1‐Li_2_** are furthermore strongly broadened, suggesting pronounced structural disorder in this sample. Additionally, the spectra of **1‐M_2_** reveal ^13^C resonances ranging in‐between 50–55 ppm assigned to alkali metal methanolate and methanol species, originating from the synthesis of **1‐M_2_** (^13^C spectra of NaOMe and KOMe support such assignments, see Figure S4). Although present in all **1‐M_2_** spectra, the methanol/methanolate resonances are more prevalent in the **1‐Cs_2_** and **1‐K_2_** samples which required a higher excess of base in their synthesis (Figure 2B). We also observe intense alkali methanolate resonances for the dipotassium catecholate sample (**1‐K_2_**), whose spectrum points to the presence of only a single (pseudo‐)polymorph, hence the presence of multiple (pseudo‐)polymorphs is most likely not the reason for the non‐reactivity of **1‐K_2_**. Note, however, that all ^13^C MAS‐NMR spectra have been recorded with a cross‐polarization transfer step, such that a direct quantification of the ^13^C resonances needs to be done with some care.

We hypothesize that the presence of remaining alkali metal methanolate (or methanol by‐product) in the solid matrix influences the reactivity of the dialkali metal catecholates **1‐M_2_** in the mechanochemical carboxylation reaction. After all, these resonances are present in **1‐K_2_** and **1‐Cs_2_** (50‐55 ppm, see Figure 2A), which are both unreactive (Table 2, entries 1–2). Furthermore, externally added alkali methanolate inhibits the reaction of **1‐Na_2_** (Table 3, entry 8), which can be explained by its reaction with CO_2_ producing sodium methyl carbonate, a known inhibitor in our reaction as well (Table 3, entry 2). Additionally, remaining protic species (such as methanol) could also be detrimental to Kolbe–Schmitt‐type reactivity [17]. Hence, the lack of reactivity of **1‐K_2_** and **1‐Cs_2_** likely originates from the way they were synthesized, requiring excess base, and may therefore not necessarily reflect their innate properties. Comparing the ^13^C CP‐MAS spectra of monosodium catecholate (**1‐Na_1_**) to the ones of the corresponding disodium catecholate **1‐Na_2_** clearly revealed the absence of the monoalkali catecholate phase in the latter (Figure S2), which we found to be non‐reactive in the mechanochemical reaction (Table 2, entry 5). All these observations underline the importance of solid‐state NMR spectroscopy to analyze solids used in mechanochemical reactions for the presence of remaining (by‐)products from their synthesis.

### 2.3 Synthesis and Evaluation of Novel Plasticizers

**FIGURE 3:**
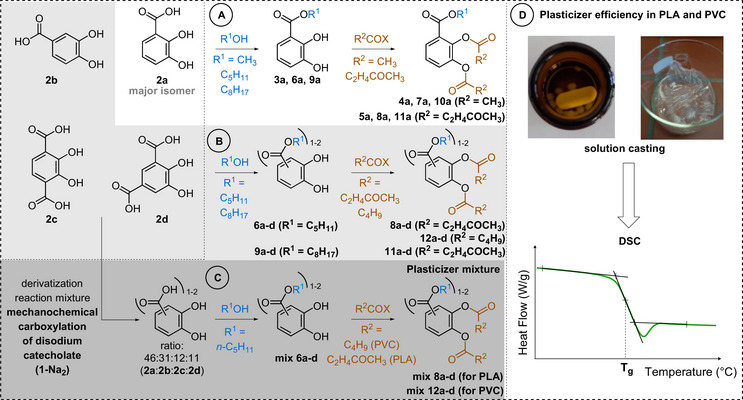
Strategy for the synthesis and efficiency evaluation of novel plasticizers by derivatizing the products of the ball mill mixture. (A) Esterification of the carboxyl group and *O*‐acylation of the phenolic hydroxy groups of the major component 2,3‐dihydroxybenzoic acid (**2a**); (B) Esterification of the carboxyl group and *O*‐acylation of the phenolic hydroxy groups of the other dihydroxybenzoic acids (**2b**, **2c**, **2d**) present in the ball mill mixture (Table 1, entry 8); (C) Esterification of the carboxyl group and *O*‐acylation of the phenolic hydroxy groups of the ball‐mill reaction mixture (**2a‐2d**). (D) Preparation of PVC and PLA blended with 10 wt% plasticizer. Determination of the glass transition temperature (*T*_g_). C_5_H_11_ = *n*‐pentyl, C_8_H_17_ = 2‐ethylhexyl, C_4_H_9_ = *n‐*butyl.

Next, the transformation of the mixture of 2,3‐dihydroxybenzoic acid (**2a**), 3,4‐dihydroxybenzoic acid (**2b**), 2,3‐dihydroxyterephthalic acid (**2c**), and 4,5‐dihydroxyisophthalic acid (**2d**) obtained by the mechanochemical Kolbe–Schmitt carboxylation reaction of **1‐Na_2_** (Table 1, entry 8) in renewable plasticizers for PLA and PVC was studied. To this end, the major component of the obtained mixture, 2,3‐dihydroxybenzoic acid (**2a**), was first derivatized and evaluated. The carboxyl group of **2a** was esterified with methanol, pentan‐1‐ol, or 2‐ethylhexan‐1‐ol (Figure 3A). The three esters still feature two free phenolic hydroxyl groups which were subsequently *O*‐acylated, that is *O*‐acetylated and *O*‐levulinoylated, resulting in a total of nine compounds for plasticizer evaluation. The carboxylic acids [68, 69, 70, 71] and alcohols [72, 73, 74, 75] selected for derivatization into esters were chosen because they can be retrieved from renewable sources and are also known plasticizer motifs [44, 76, 77, 78, 79, 80, 81].

**FIGURE 4:**
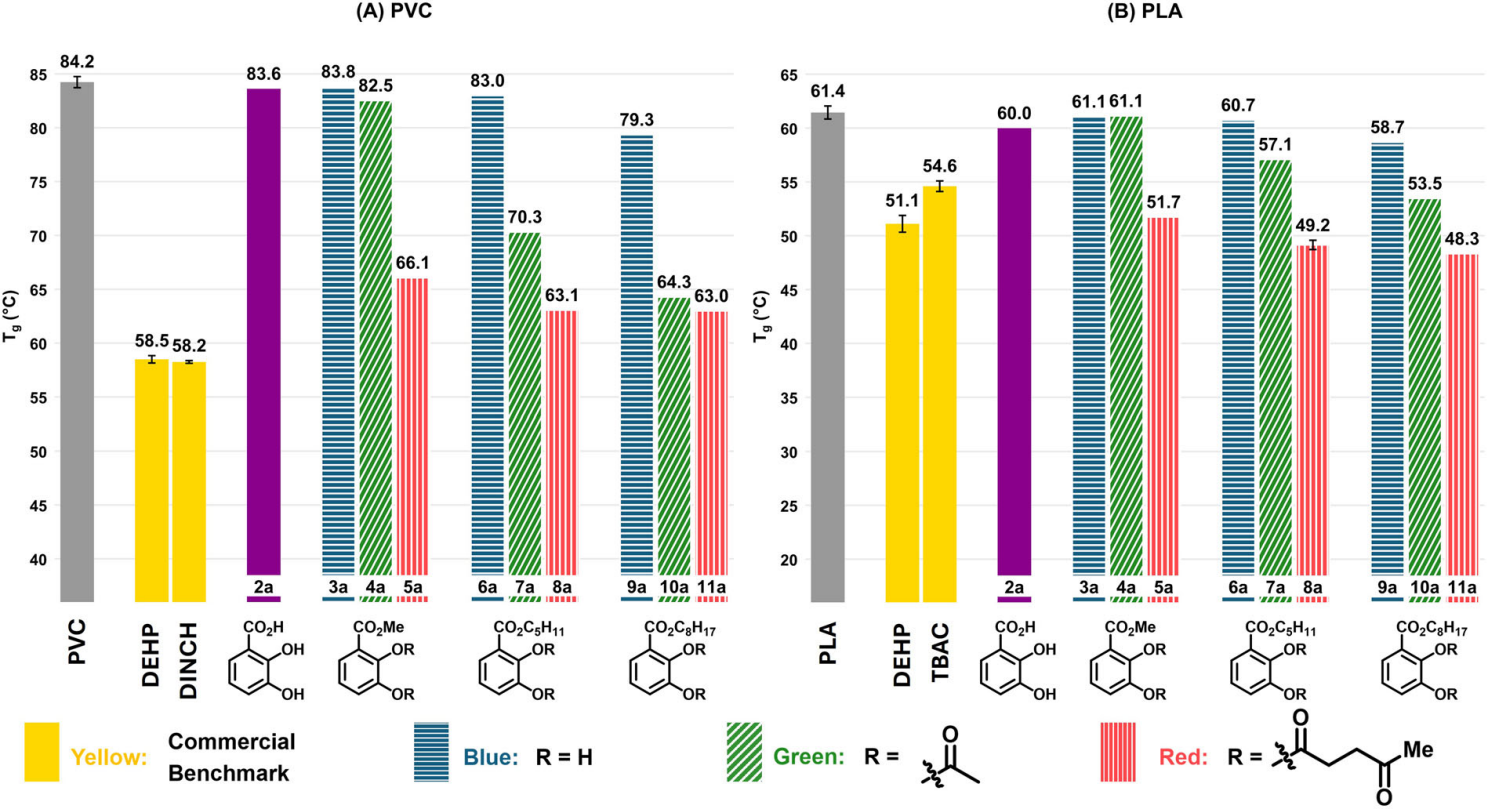
Plasticizing efficiency of 2,3‐dihydroxybenzoic acid (**2a**)‐derived plasticizer candidates in PVC (A) and PLA (B). DEHP: bis(2‐ethylhexyl) phthalate; DINCH: bis(7‐methyloctyl) cyclohexane‐1,2‐dicarboxylate; TBAC: tributyl *O‐*acetyl citrate. Glass transition temperature (*T*_g_) determined using Differential Scanning Calorimetry (DSC) of polymers containing 10 wt% of the respective plasticizer candidate. *T*_g_ values for pure polymer and benchmark plasticizers are shown as the average of four measurements with standard deviation shown. *T*_g_ values for **2a**‐**11a** originate from single measurements, except for **8a** in PLA, which is shown as the average of four measurements with standard deviation shown. C_5_H_11_ = *n*‐pentyl, C_8_H_17_ = 2‐ethylhexyl.

With the synthesized plasticizer candidates derived from 2,3‐dihydroxybenzoic acid (**2a**) in hand, their plasticizer performance was investigated in PVC and PLA (Figure 3D). To this end, the solution casting method (Figure S21) was used in which plasticizer candidates (10 wt%) were mixed with the polymers PVC or PLA (90 wt%) by fully dissolving both components in a compatible solvent (for detailed experimental procedures, see Supporting Information section 4). The glass transition temperature (*T*_g_) determined by differential scanning calorimetry (DSC) serves as a measure for plasticizing efficiency in which a decrease of the *T*_g_ indicates an increased plasticizing effect of the added plasticizer. The performance of the compounds was consequently compared with state‐of‐the‐art commercial benchmark plasticizers such as bis(2‐ethylhexyl) phthalate (DEHP) (for a complete overview, see Figures S23 and S24). The non‐derivatized 2,3‐dihydroxybenzoic acid (**2a**) does not possess a typical plasticizer structure (lacking ester spacer groups) and was therefore used as a reference. As expected, no plasticizing effect was observed for **2a** in PVC and PLA indicating that derivatization is crucial to access plasticizers (Figure 4, purple). The benzenecarboxylic ester derivatives featuring free hydroxyl groups (**3a**, **6a**, **9a**) also do not show considerable plasticizing properties in PVC and PLA (Figure 4, blue). Only the 2‐ethylhexyl ester **9a** shows slight plasticizing effect in both matrices. The insufficient presence of spacer moieties circumvents efficient plasticization here. Therefore, the phenolic hydroxyl groups of the esterified derivatives of **2a** (i.e. **3a**, **6a**, **9a**) were further *O*‐acylated with acetyl (**4a**, **7a**, **10a**) (Figure 4, green) and levulinoyl groups (**5a**, **8a**, **11a**) (Figure 4, red). Examination of the plasticizing results revealed that the *O*‐acetylated alkyl 2,3‐dihydroxybenzoates exhibited superior performance with pentyl (**7a**, **8a**) and 2‐ethylhexyl (**10a**, **11a**) esters, relative to the compounds with free hydroxyl groups (**6a**, **9a**) (Figure 4). On the other hand, the *O*‐acetylated methyl ester **4a** only showed a marginal effect both in PVC and PLA. Interestingly, in both polymer matrices, the *O*‐levulinoylated compounds (**5a**, **8a**, **11a**) (Figure 4, red) generally showed enhanced performance versus the *O*‐acetylated compounds (**4a**, **7a**, **10a**) (Figure 4, green), irrespective of the benzenecarboxylic ester. The *O*‐acylations introduce additional ester functionalities, which are clearly beneficial in terms of plasticizing properties. The *O*‐levulinoylation offers a longer carbon chain spacer (featuring a ketone moiety) compared to the *O*‐acetyl derivatives explaining their superiority. Upon comparing the different *O*‐levulinoylated esters, again the methyl ester **5a** displays the lowest performance, while the longer chain pentyl (**8a**) and 2‐ethylhexyl (**11a**) esters deliver the best results.

**FIGURE 5:**
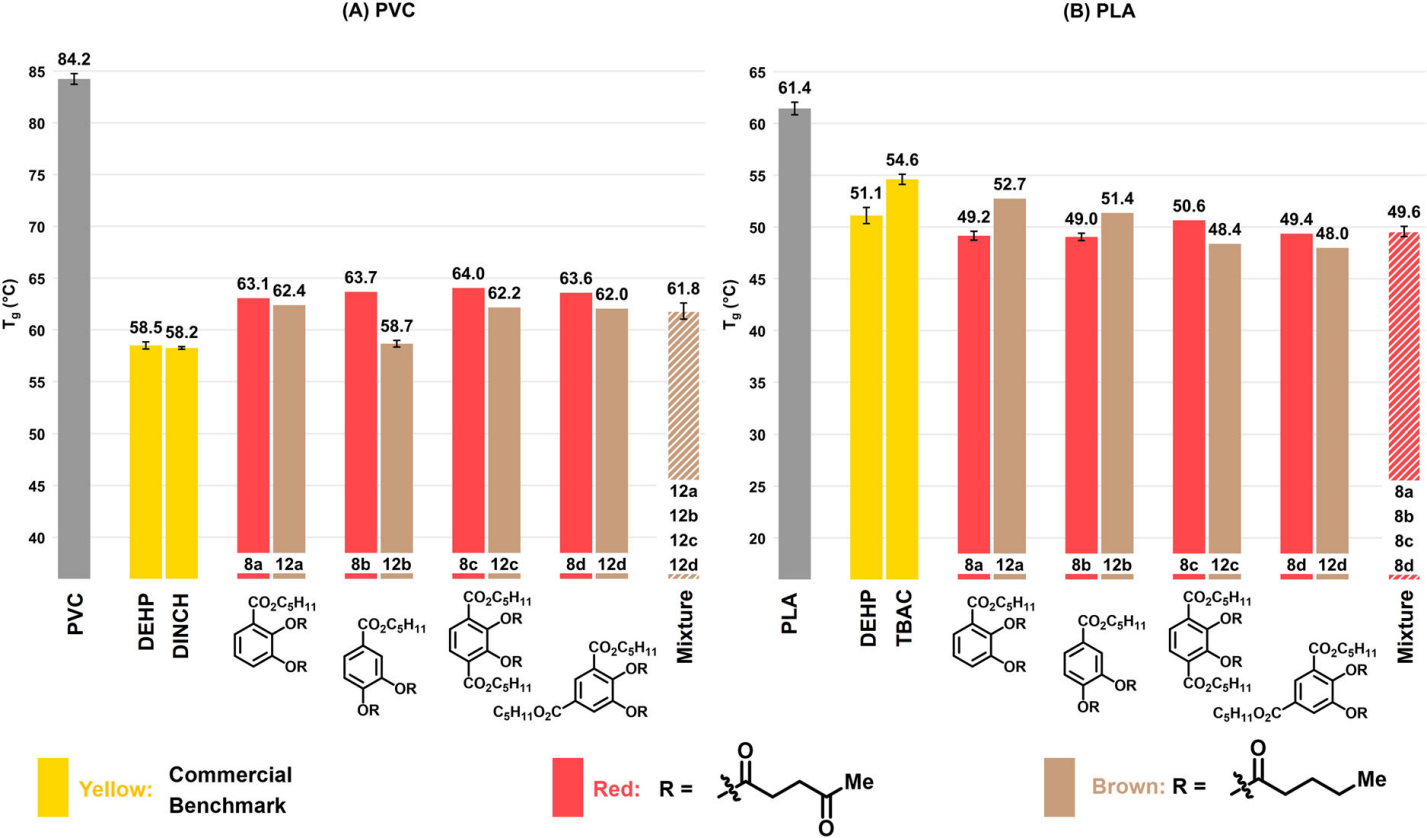
Plasticizer efficiency the *O*‐pentanoylated pentyl esters **12a**‐**12d** (brown) compared with that of the *O*‐levulinoylated pentyl esters **8a**‐**8d** (red) in PVC (A) and PLA (B). Mixtures of plasticizer families **12a**‐**12d** for PVC and **8a**‐**8d** for PLA were synthesized from the mixture of **2a**‐**2d** obtained from catechol via mechanochemical carboxylation (Table 1, entry 8) and compared to the individual compounds in PVC and PLA, respectively. DEHP: bis(2‐ethylhexyl) phthalate; DINCH: bis(7‐methyloctyl) cyclohexane‐1,2‐dicarboxylate; TBAC: tributyl *O‐*acetyl citrate. Glass transition temperature (*T*_g_) determined using Differential Scanning Calorimetry (DSC) of polymers containing 10 wt% of the respective plasticizer candidate. *T*_g_ values for pure polymer, benchmark plasticizers, mixtures (**12a**‐**12d** in PVC and **8a**‐**8d** in PLA), and best performing individual plasticizers (**12b** in PVC and **8a**, **8b** in PLA) are shown as the average of four measurements with standard deviation shown. All other *T*_g_ values originate from single measurements. C_5_H_11_ = *n‐*pentyl.

Following the plasticizing performance of 2,3‐dihydroxybenzoic acid‐derivatives, the *O*‐levulinoylated pentyl (**8b**, **8c**, **8d**) and *O*‐levulinoylated 2‐ethylhexyl esters (**11b**, **11c**, **11d**) of the other mono‐ [3,4‐dihydroxybenzoic acid (**2b**)] and dicarboxylated catechols [2,3‐dihydroxyterephthalic acid (**2c**) and 4,5‐dihydroxyisophthalic acid (**2d**)] present in the mechanochemical reaction mixture were synthesized (Figure 3B) and each tested separately as plasticizer in PVC and PLA (see Supporting Information section 7). Surprisingly, the plasticizing properties of these other compounds are largely independent on the regioisomer and the presence of one or two carboxylic acids. These behave very similar to those derived from the major component **2a** in both PVC and PLA, which is very appealing as the compound mixture **2a**‐**2d** generated in the mechanochemical synthesis can then be derivatized (esterification with alcohol and carboxylic acid) and used as a plasticizer mixture. In PVC, the *O*‐levulinoylated pentyl esters (**8a**, **8b**, **8c**, **8d**) (Figure 5A) and the *O*‐levulinoylated 2‐ethylhexyl esters (**11a**, **11b**, **11c**, **11d**) (Figure S26A) each show good plasticizer potential, with *T*_g_ values between 63.1‒64.0°C and 63.0‒65.1°C, respectively (vs. 58.2°C for the commercial DINCH benchmark). In PLA, the *O*‐levulinoylated pentyl (**8a**, **8b**, **8c**, **8d**) (Figure 5B) and 2‐ethylhexyl esters (**11a**, **11b**, **11c**, **11d**) (Figure S26B) all outperformed the benchmarks, with *T*_g_ values between 49.0‒50.6°C and 47.9‒49.4°C, respectively (vs. 51.1°C for commercial DEHP benchmark). Due to the high boiling point of 2‐ethylhexan‐1‐ol, its removal after Fischer esterification was tedious and energy‐intensive. For this reason, we opted to continue with the *O*‐levulinoylated pentyl esters (**8a**, **8b**, **8c**, **8d**) for both PVC and PLA.

With the commercial benchmarks hitherto not matched in PVC, the search toward improved plasticizers was continued with the exchange of the levulinoyl group in the best performing pentyl ester family (**8a**, **8b**, **8c**, **8d**) by the less polar pentanoyl group. The *O*‐pentanoylated derivatives (**12a**, **12b**, **12c**, and **12d**) generally performed better compared to their *O*‐levulinoylated analogues with *T*_g_ values between 58.7‒62.4°C (vs. 58.2°C for commercial DINCH benchmark) in PVC (Figure 5A). Gratifyingly, the 3,4‐dihydroxy regioisomer (**12b**) was now found to be competitive with the commercial benchmarks. To allow a proper comparison, these *O*‐pentanoylated derivatives **12a**, **12b**, **12c**, and **12d** were also tested in PLA. Interestingly, the relative performance compared to the *O*‐levulinoylated derivatives is dependent on the derivative of **2a**‐**d**. With *T*_g_ values between 48.0‒52.7°C (vs. 51.1°C for DEHP benchmark), the *O*‐levulinoylated derivatives are still preferred in PLA (Figure 5B). The difference in polarity between the *O*‐levulinoylated and *O*‐pentanoylated compounds, matching polarity of the polymer better, explain their varying behavior in PVC and PLA.

**FIGURE 6:**
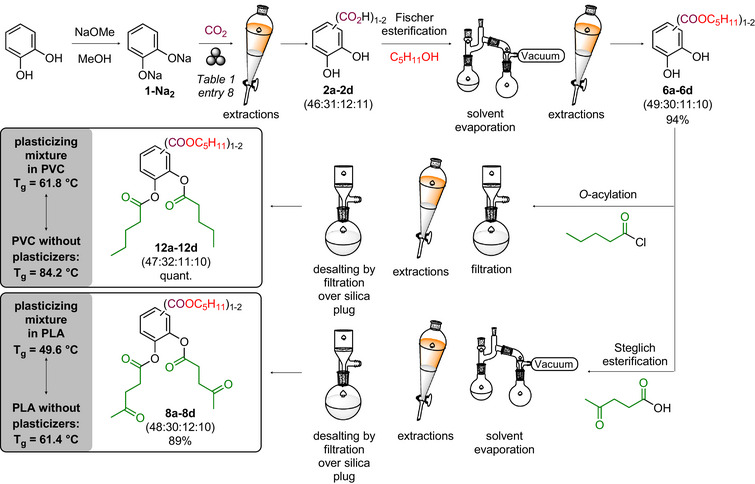
Transformation of the mixture of carboxylated catechols (**2a**‐**2d**) obtained by mechanochemistry into novel plasticizer families for PVC (**12a**−**12d**) and PLA (**8a**−**8d**) using esterifications and simple and cheap work‐up techniques (i.e. distillation, extraction, filtration). C_5_H_11_ = *n*‐pentyl.

Considering the mechanochemical synthesis directly delivers a mixture of **2a**, **2b**, **2c**, and **2d**, the derived *O*‐pentanoylated pentyl esters (**12a**, **12b**, **12c**, and **12d**) and *O*‐levulinoylated pentyl esters (**8a**, **8b**, **8c**, and **8d**) were then synthesized starting from the mixture of the mono‐ and dicarboxylated catechols (**2a**, **2b**, **2c**, and **2d**) obtained in the mechanochemical procedure (ratios: 46:31:12:11) (Table 1, entry 8). This allows the assessment of their performance as a mixture, which avoids a separation of the regioisomeric carboxylic acids. After all, the mixture might perform similar in terms of plasticizing properties compared to the individual compounds and specialty chemicals are commonly applied as mixtures [76, 82]. To this end, the mixture of carboxylic acids obtained from the mechanochemical Kolbe–Schmitt reaction was derivatized in two esterification steps toward two plasticizer families in high yield, using simple and cheap work‐up techniques avoiding column chromatography (for synthesis optimization and details, see Supporting Information sections 10 and 11) (Figure 6). In PVC, the mixture of *O*‐pentanoylated pentyl esters (**12a**, **12b**, **12c**, and **12d**) resulted in excellent plasticizing properties with a *T*_g_ of (61.8 ± 0.8)°C, roughly corresponding to the weighted average of the *T*_g_’s of the individual compounds (Figure 5A). In PLA, the mixture of *O*‐levulinoylated pentyl esters (**8a**, **8b**, **8c**, and **8d**) resulted in a *T*_g_ of (49.6 ± 0.5)°C, which is comparable with each of the individual components and outperforms the commercial benchmarks in PLA (Figure 5B). Gratifyingly, the mixtures perform similar to the individual compounds, thereby allowing to directly valorize the mixture of the carboxylic acids (**2a**, **2b, 2c**, and **2d**) obtained in the ball milling carboxylation reaction. Furthermore, mixtures **12a**–**12d** and **8a**–**8d** both demonstrate concentration‐dependent reductions in *T*_g_ in respectively PVC and PLA (Figure S30).

## 3 Conclusion

In conclusion, a mild mechanochemical method for the carboxylation of catechol using gaseous CO_2_ has been disclosed. Under the optimized conditions, a mixture of two mono‐ and two dicarboxylic acids was obtained. Similar to the thermal process disodium catecholate is used as substrate for the Kolbe–Schmitt reaction. Solid‐state NMR spectroscopy was employed to characterize the synthesized disodium catecholate and allows to check for remaining unreactive monosodium catecholate as well as sodium methanolate base and methanol byproduct inhibiting the reaction. From the obtained individual mono‐ and dicarboxylic acids, a library of plasticizers was synthesized by Fischer esterification of the carboxylic acid groups and *O*‐acylation of the phenolic hydroxyls. Subsequently, the individual synthesized compounds were tested in PVC and PLA, showcasing dihydroxybenzoic, ‐terephthalic and ‐isophthalic acids as novel and versatile renewable platform molecules. Interesting trends in plasticizing behavior were observed. While in PVC, a single isomer, that is pentyl 3,4‐bis(pentanoyloxy)benzoate, was found to be competitive with commercial petrochemical plasticizers (DEHP and DINCH), in PLA, each member of the family of *O*‐levulinoylated pentyl esters outcompeted the commercial petrochemical (DEHP) and edible citric acid‐derived renewable (TBAC) benchmark plasticizers. Moreover, plasticizer blends directly derived from the mixture of dihydroxybenzoic, ‐terephthalic and ‐isophthalic acids obtained by the mechanochemical Kolbe–Schmitt reaction and subsequent esterifications, also showed excellent plasticizer performance in PVC and PLA. This allows to valorize the ball mill reaction mixture without separation and, combined with simple and cheap esterification downstream processing gives access to a novel class of renewable plasticizers. Further work will involve the evaluation of other (mechanical) properties of the plasticized PVC and PLA as well as the (bio)degradability of the plasticizers. While this work directly valorizes the mixture of carboxylated catechols (**2a**‐**2d**), separation of the isomers in individual compounds or mixtures in other ratios will open other applications. Membrane separation has shown to be a promising technique to (partly) separate these structurally similar and polar phenolic carboxylic acids [83].

## Conflicts of Interest

The authors declare no conflicts of interest.

## Supporting information




**Supporting File 1**: anie71291‐sup‐0001‐SuppMat.pdf.

## Data Availability

The data that support the findings of this study are available in the supplementary material of this article.
