# Supplementary material for: A Mechanochemical Kolbe–Schmitt Reaction: Catechol Carboxylation Provides Building Blocks for Renewable Plasticizers
Source: Angew Chem Int Ed Engl. 2026 Feb 9;65(12):e19827. doi: 10.1002/anie.202519827 (PMC12990962; doi:10.1002/anie.202519827)
Supplement: Supplementary file 1 — Supporting File 1: anie71291‐sup‐0001‐SuppMat.pdf. [file ANIE-65-e19827-s001.pdf]

Supporting Information

A Mechanochemical Kolbe-Schmitt reaction: Catechol Carboxylation Provides Building Blocks for Renewable Plasticizers

D. De Vos,[a] V. S. Pfennig,[b#] A. Goddé,[a#] R. Vroemans,[a] T. Krückel,[b,d] N. Marcinkowska,[c] E. Bartalucci,[c,d] T. Wiegand,[c,d] C. Bolm,[b*] B. U. W. Maes[a*]

a Division of Organic Synthesis, Department of Chemistry, University of Antwerp, Groenenborgerlaan 171, B-2020 Antwerp, Belgium

b Institute of Organic Chemistry, RWTH Aachen University, Landoltweg 1, 52074 Aachen, Germany

c Institute of Technical and Macromolecular Chemistry, RWTH Aachen University, Worringerweg 2, 52074 Aachen, Germany

d Max Planck Institute for Chemical Energy Conversion, Stiftstr. 34-36, 45470 Mülheim a. d. Ruhr, Germany

# equal contributions

Table of contents

1. General considerations 4

2. Synthetic procedures and characterization of the catecholate salts 7

2.1 Synthesis of catecholates 7

2.1.1 Synthesis of dialkali metal catecholates 7

2.1.2 Synthesis of monoalkali metal catecholates 8

2.2 Solid-state NMR characterization of catecholate salts 10

3. Synthetic procedures and characterization of the mechanochemical carboxylation reaction 15

3.1 Initial experiments 15

3.2 Identification of the obtained reaction products 17

3.3 Optimization of the mechanochemical carboxylation 27

3.3.1 Solid-state NMR spectroscopy of dialkali metal catecholates 29

3.3.2 Addition of additives and control reactions 30

3.3.3 Optimized general procedure for mechanochemical carboxylation reactions 31

3.3.4 Calculation of the amount of CO_2 added to milling vessels 33

3.3.5 Upscaling procedure for ball-mill derived plasticizer mixture 33

3.4 Quantification of the ball mill mixtures for plasticizer candidate synthesis 34

4. Solution casting of polymers with plasticizers and DSC characterization 35

4.1 Procedure for PVC samples 35

4.2 Procedure for PLA samples 35

4.3 Photographic report of solution casting and DSC characterization 36

5. Plasticizer performance of commercial benchmark plasticizers 38

6. Derivatizations on 2,3-dihydroxybenzoic acid (**2a**) to obtain plasticizing properties 40

6.1 Derivatization of 2,3-dihydroxybenzoic acid (**2a**) towards plasticizer candidates for PVC and PLA 40

6.1.1 Fischer esterification 40

6.1.2 *O*-Acylation of the phenolic hydroxyl groups 41

6.2 Testing the plasticizer efficiency of 2,3-dihydroxybenzoic acid derivatives **3a**-**11a** in PVC and PLA 43

7. Derivatization of 3,4-dihydroxybenzoic acid (**2b**), 2,3-dihydroxyterephthalic acid (**2c**) and 4,5-dihydroxyisophthalic acid (**2d**) to obtain plasticizing properties. 45

7.1 Synthesis of *O*-levulinoylated pentyl esters **8a**-**8d** and *O*-levulinoylated 2-ethylhexyl esters **11a**-**11d** 45

7.2 Testing of the plasticizing efficiency of *O*-levulinoylated pentyl esters **8a**-**8d** and *O*-levulinoylated 2-ethylhexyl esters **11a**-**11d** in PVC and PLA 47

8. Exchanging *O*-levulinoylation by *O*-pentanoylation: comparing *O*-pentanoylated pentyl esters **12a**-**12d** with *O*-levulinoylated pentyl esters **8a**-**8d** 48

8.1 Synthesis of *O*-pentanoylated pentyl esters **12a**-**12d** 48

8.2 Testing the plasticizing efficiency of *O*-pentanoylated pentyl esters **12a**-**12d** in PVC and PLA and comparing them to *O*-levulinoylated pentyl esters **8a**-**8d** 48

9. Optimization of synthesis of *O*-levulinoylated pentyl esters **8a**-**8d** and *O*-pentanoylated pentyl esters **12a**-**12d** 50

9.1 *O*-Levulinoylation reaction 50

9.1.1 Fischer esterification 50

9.1.2 Steglich esterification with greener solvents 51

9.1.3 *O*-Acylation with (pseudo)levulinoyl chloride (**13**) 51

9.2 *O*-Pentanoylation reaction 56
10. Derivatization of model mixture (**2a**-**2d**) towards plasticizer candidates and testing 57
10.1 Derivatization of model mixture (**2a**-**2d**) towards mixture of *O*-pentanoylated pentyl esters (**12a-12d**) and *O*-levulinoylated pentyl esters (**8a**-**8d**) 57
10.2 Comparison of the plasticizer efficiency of mixtures of *O*-pentanoylated pentyl esters (**12a**-**12d**) and *O*-levulinoylated pentyl esters (**8a**-**8d**) with each individual pure component 59
10.3 Evaluation of the plasticizer efficiency at different concentrations 60
11. Derivatization and plasticizer performance of ball mill-derived plasticizer mixtures 61
12. Synthetic protocols and characterization of plasticizer candidates 63
12.1 General procedures 63
12.2 Experimental protocols and characterization of plasticizer candidates **3a**-**11a** 64
12.3 Experimental protocols and characterization of plasticizer candidates **6b**-**6d** and **9b**-**9d** 70
12.4 Experimental protocols and characterization of plasticizer candidates **8b**-**8d** and **11b**-**11d** 75
12.5 Experimental protocols and characterization of plasticizer candidates **12a**-**12d** 79
12.6 Characterization of isolated byproducts 82
12.7 Experimental protocols for synthesis and characterization of reactants 83
12.8 Experimental protocols for synthesis and characterization of mixtures of **8a**-**8d** and **12a**-**12d** starting from model mixtures of **2a**-**2d** 85
12.9 Experimental protocols for synthesis and characterization of mixtures of **8a**-**8d** and **12a**-**12d** starting from ball mill-derived mixture of **2a**-**2d** 92
13. NMR spectra of plasticizer candidates 96
14. DSC thermograms 156
14.1 DSC thermograms in PVC 156
14.2 DSC thermograms in PLA 181
References 210

1. General considerations

For the synthesis and characterization of (di)metal catecholates and ball milling reactions:

For the synthesis and work-up steps that were carried out in solution, the used glassware was cleaned by immersion in a KOH/isopropanol bath, then in a 4 M HCl bath and finally in deionized H_2O and dried at 120 °C for at least five hours. Reaction steps in solution were stirred with Teflon-coated magnetic stirring bars and magnetic stirring plates. Solvents were either purchased with the required degree of purity or obtained in technical purity and then distilled before use [*i.e.* diethyl ether (Et_2O) and *n*-pentane used for column chromatography]. An MB-SPS-5 solvent purification system from MBRAUN was used to obtain dry toluene and dry tetrahydrofuran (THF). Rotary evaporators with a water bath temperature of 40–55 °C and correspondingly reduced pressure were used to distill or separate solvents.

For work steps that were not carried out in ambient air, standard Schlenk technique with argon as a protective gas was used. Solvents and other liquid chemicals were transferred using plastic syringes with stainless steel needles. Glassware, syringes, cannulas, ball milling accessories and solid chemicals were subjected to three cycles of evacuation and flushing with argon before use. Weighing and refilling under inert conditions was carried out in a LabMaster 130 glovebox from MBRAUN in an argon atmosphere.

For column chromatography, silica gel 60 (63–200 µm particle size) or 60 M (40–63 µm particle size) from Macherey-Nagel was used as the stationary phase. For loading the columns, crude products were adsorbed onto Hyflo Super Cel® diatomaceous earth (Celite) from Aldrich. For this purpose, the mixture to be separated was dissolved in Et_2O, an appropriate amount of Celite was added and the solvent was removed using a rotary evaporator. For elution, an overpressure of 0.1–0.5 bar compressed air was applied, and solvent mixtures were used in the specified volume ratio (V/V). Chemicals were purchased from common suppliers and used without further purification unless otherwise stated. Gaseous CO_2 was obtained from Westfalen with a purity of at least 99.8%.

Mechanochemical reaction steps were conducted in a planetary or mixer mill with respective specific equipment. As planetary mill, a Fritsch Pulverisette 7 premium line was used. As equipment, magnesia-stabilized zirconia (ZrO_2-M) with 20 mL inside volume and gas valves were used. Milling balls equally consisted of ZrO_2-M. As mixer mill, a Retsch MM400 was used with vessels of either stainless steel (10 or 20 mL inside volume), yttrium-stabilized zirconia (ZrO_2-Y, 10 mL inside volume) or tungsten carbide (10 mL inside volume) and balls of the same material, respectively.
Thin layer chromatography (TLC) was performed using an organic solvent as mobile phase and silica-coated aluminum plates (Macherey-Nagel Precoated TLC sheets Alugram® SIL G/UV254) as stationary phase. Compounds were detected by irradiation with UV light (254 nm).

The recording of liquid phase NMR spectra was carried out on a Varian Mercury 300, VNMRS 400 or VNMRS 600 spectrometer or a Bruker Avance Neo 400 or Avance Neo 600 spectrometer, each at room temperature, unless otherwise stated. ^{13}C NMR experiments were measured with proton broadband decoupling, indicated as $^{13}C\{^1H\}$. The Mestrenova program from Mestrelab Research was used to analyze the experiments.[1] Chemical shifts δ are given in parts per million (ppm) and were referenced to the residual proton signal of the incompletely deuterated solvent used for ^1H and $^{13}C\{^1H\}$ NMR spectra.[2] The multiplicities of the signals are named with the following abbreviations or combinations thereof: s (singlet), d (doublet), t (triplet), q (quartet), quint (quintet), sext. (sextet), sept (septet), m (multiplet), br (broad signal). Spin-spin coupling constants *J* are given in Hertz (Hz).

Solid-state NMR experiments were performed on a Bruker AVANCE III HD 500 MHz spectrometer in wide-bore Bruker standard triple-resonance 3.2 mm and 4.0 mm probes. Chemical-shift values were referenced to TMS, making use of an external calibration on the ^{13}C-detected spectrum of adamantane recorded in the same probe directly before the measurements. All experiments were recorded with temperature control, setting the VTU target temperature to 270 K for NaOMe and KOMe samples and 280 K for all other measurements.

For the synthesis and characterization of the plasticizer candidates:
Unless stated otherwise, all solvents and commercially available reagents and reactants were used as received. Chemicals were purchased from Aldrich, TCI Europe, Acros Organics, Thermo Fisher Scientific, Fluorochem, AmBeed, BLD Pharm or Euroisotop. PVC was purchased from Aldrich with a M_w of 80000 and M_n of 47000. PLA was purchased from NatureWorks with product name Ingeo™ Biopolymer 2500HP. Heptanes, used for flash chromatography, were distilled prior to use. Non-commercial starting materials were prepared as described below. Water was deionized using a EUROTEC L4 reverse osmosis plant. The used water had a conductivity of max. 0.5 $\mu S \cdot cm^{-1}$.

Chromatographic purification was performed using an automated flash chromatography Büchi Pure C-850 Flashprep system with on-line UV and evaporative light scattering (ELS) detection utilizing commercially available Silica Flash Cartridges (12, 40, or 80 g) at a flow rate of 30-40 mL/min. Thin-layer chromatography (TLC) analysis was performed using precoated TLC aluminum sheets ALUGRAM SIL G/UV_{254} (layer: 0.20 mm silica gel with fluorescent indicator UV254). The spots were detected with UV light at 254 nm or 366 nm. Melting points were measured on a Melting Point B-545 apparatus and are uncorrected. DSC measurements were performed on a TA DSC Q2000, with a TA refrigerated cooling system, using open aluminum sample pans.

Nuclear magnetic resonance (NMR) spectra were recorded on a Bruker Avance III 400 Fourier transform NMR spectrometer in $CDCl_3$ or DMSO-d_6 at 303 K (unless stated otherwise). Samples were prepared using *ca.* 10−50 mg of compound dissolved in 1.0 mL of deuterated solvents ($CDCl_3$ or DMSO-d_6). Spectra were referenced to the TMS reference peak for samples in $CDCl_3$ whenever possible (δ = 0.00 ppm for ^1H and δ =

0.00 ppm for ^{13}C). In the absence of the TMS reference peak for samples in $CDCl_3$, the solvent residual peak was used as reference (δ = 7.26 ppm for ^{1}H and δ = 77.16 ppm for ^{13}C). Spectra were referenced to the solvent residual peak for samples in DMSO-d_6 (δ = 2.50 ppm for ^{1}H and δ = 39.52 ppm for ^{13}C). Chemical shifts (δ) are reported in ppm; coupling constants (*J*) are reported in Hz; splitting patterns are assigned as singlet (s), broad singlet (br s), doublet (d), triplet (t), quartet (q), quintet (quint), multiplet (m) or combinations thereof. All spectra can be found in section 13. For all synthesized compounds, ^{1}H, $^{13}C\{^{1}H\}$ (all were measured with proton decoupling and further denoted as ^{13}C), ^{13}C APT and ^{1}H COSY spectra are recorded.

High-resolution mass spectrometry (HRMS) samples were prepared by dissolving 1.5 mg of the compound in MeOH/H_2O (50/50) + 0.1% formic acid and diluting to a concentration of 10^{-5} mol/L. 5 µL of each sample was injected using the Acquity UPLC system (Waters, Manchester, UK) and electrosprayed using a standard electrospray source at 40 µL/min. Samples were injected with an interval of three minutes. Accurate mass spectra were acquired using a Waters Synapt G2 system. The MS was calibrated prior to use with a 0.1% H_3PO_4 solution. The spectra were lock mass corrected using the known m/z of the nearest H_3PO_4 cluster (from a H_3PO_4 standard solution which was injected three times before a measurement and again between every six samples) or nearest known background ion. Analytes were detected as protonated and/or sodiated molecules unless stated otherwise. Some substrates featuring a free catechol moiety could not be detected using this method.

ASAP-MS unit resolution mass spectra were recorded on an expression-L mass spectrometer equipped with a APCI source and Atmospheric Solids Analysis Probe ASAP (Advion Interchim Scientific). Samples were taken by dipping the probe in the sample, allowing any solvent to evaporate and introducing the sample with the probe into the APCI source. Both positive and negative ion spectra were recorded. Capillary temperature: 200°C, Capillary voltage: 120 V, Source gas temperature: 350 °C, APCI corona discharge: 5 µA.

TLC-MS mass spectra were recorded on a Micromass ZQ (Waters) mass spectrometer equipped with an ESI source in positive ion mode. Capillary voltage 1.5 kV, Cone voltage: 20 V, Source temperature: 120 °C, Desolvation temperature: 400 °C, Desolvation gas (N_2): 600 L/h, Cone gas: 0-50 L/h. Samples were extracted for 30 s from a TLC plate using a Plate Express (Advion Interchim Scientific). The flow rate was set at 0.3 mL/min and consisted of MeOH/H_2O (9:1).

Differential Scanning Calorimetry (DSC) measurements were performed on a TA DSC Q2000 with TA cooling system and autosampler, in open aluminum pans under nitrogen flow.

2. Synthetic procedures and characterization of the catecholate salts

2.1 Synthesis of catecholates

2.1.1 Synthesis of dialkali metal catecholates

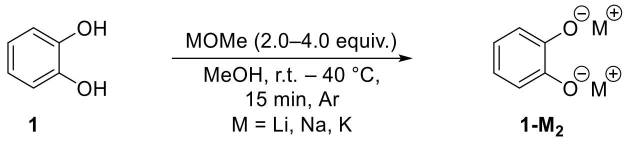

Scheme S1: Synthesis of 1-M$_2$ by reaction of 1 with alkali metal methanolates (M = Li, Na, K).

1-M$_2$ (Li, Na, K). Catechol (**1**, 1652 mg, 15 mmol, 1.0 equiv.) was added to a 100 mL Schlenk flask equipped with a septum and a magnetic stirring rod and subjected to three cycles of evacuation and flushing with argon gas. Dry MeOH (12 mL) was added through a septum and then the light in the fume cupboard was switched off. NaOMe (5.4 M in MeOH, 5.6 mL, 30.0 mmol, 2.0 equiv.) or LiOMe (2.2 M in MeOH, 14 mL, 30.8 mmol, 2.05 equiv.) or KOMe (25% in MeOH, 19.8 mL, 60.0 mmol, 4.0 equiv.) was added through a syringe and the septum was replaced with a lid in argon counterflow. The mixture was heated from room temperature to 40 °C for 15 min while stirring with a water bath. The solvent was then distilled off under reduced pressure into an inert secondary cooling trap and the resulting precipitate was then dried under vacuum at 60 °C for at least 12 h. The flask was transferred to the glovebox and the cream-colored to pale green solid was transferred to a snap-cap vial. Due to the considerable air sensitivity of the products, no further properties of the solids were analyzed apart from the $^1H \rightarrow ^{13}C$-CPMAS spectroscopic analysis (see Figure S1).

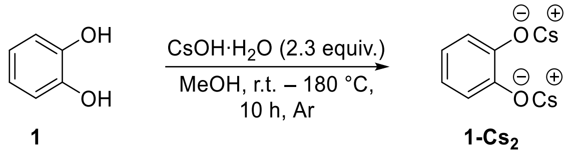

Scheme S2: Synthesis of 1-Cs$_2$ by reaction of 1 with cesium hydroxide.

1-Cs$_2$. Catechol (**1**, 551 mg, 5.0 mmol, 1.0 equiv.) and $CsOH \cdot H_2O$ (1931 mg, 11.5 mmol, 2.3 equiv.) were added to a 100 mL Schlenk flask equipped with a septum and a magnetic stirring rod and subjected to three cycles of evacuation and flushing with argon gas. Dry MeOH (12 mL) was added through a septum, the light in the fume cupboard was switched off and the septum was replaced with a lid in argon counterflow. The mixture was stirred until complete dissolution of the $CsOH.H_2O$ salt. Next, the solvent was distilled off under reduced pressure into an inert secondary cooling trap and the resulting precipitate was dried under vacuum at 180 °C for 10 h. The flask was transferred to the glovebox and the cream-colored solid was transferred to a snap-cap vial. Due to the considerable air sensitivity of the product, no further properties of the solid were analyzed apart from the ^1H-^{13}C CP-MAS spectroscopic analysis (see Figure S1).

2.1.2 Synthesis of monoalkali metal catecholates

While monosodium catecholate (**1-Na$_1$**) was prepared according to a specification by Couhorn and Dronskowski,[3] monopotassium catecholate (**1-K$_1$**) was the only product obtained in a synthesis that aimed at synthesizing dipotassium catecholate (**1-K$_2$**) using 2.1 equiv. KOMe and was therefore not attempted to be produced in other ways.

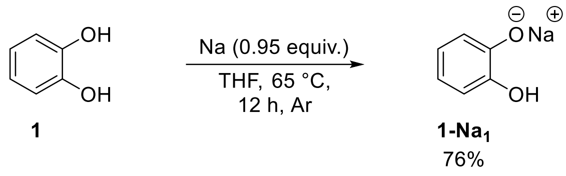

Scheme S3: Synthesis of 1-Na$_1$ by reaction of 1 with sodium.

1-Na$_1$. Freshly cut sodium (315 mg, 13.7 mmol, 1.0 equiv.) was rinsed with *n*-pentane and weighed into 100 mL Schlenk flask under a stream of argon. A magnetic stirring rod and an adjusted amount of catechol (**1**, 1584 mg, 14.4 mmol, 1.05 equiv.) were added and the flask was subjected to three cycles of evacuation and flushing with argon gas. Using a septum, dry THF (11 mL) was added, and a dried reflux condenser was attached under argon counterflow. The condenser was connected to a closed cooling water system and remained under argon for the entire duration of the reaction. The mixture was heated to 65 °C in an oil bath for 18 h while stirring. The mixture was then allowed to cool to room temperature, the reflux condenser was replaced with a septum and the solvent was filtered through a cannula with filter paper under slight argon overpressure into another dry Schlenk flask, such that the forming precipitate remained in the reaction flask. The residue was mixed with fresh, dry THF (2×10 mL) and the solvent was also pressed out through the filter cannula. The solid was then dried under vacuum at 60 °C for at least 12 h. The flask was then transferred into the glovebox and the resulting monosodium catecholate (**1-Na$_1$**, 1374 mg, 10.4 mmol, 76%) was obtained as a colorless solid and transferred to a snap cap vial. Due to the considerable air sensitivity of the product which turned dark green upon exposure to ambient air for a few minutes, no further properties of the solid were analyzed apart from the ^{1}H-^{13}C CP-MAS spectroscopic analysis (see Figure S2).

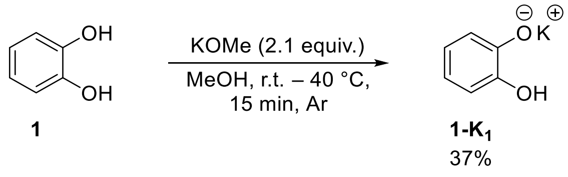

Scheme S4: Synthesis of 1-K$_1$ with potassium methanolate.

1-K$_1$. Catechol (**1**, 1652 mg, 15 mmol, 1.0 equiv.) was placed in a 100 mL Schlenk flask equipped with a septum and a magnetic stirring rod. Dry MeOH (40 mL) was added and the light in the fume cupboard was switched off. KOMe (25% in MeOH, 10.4 mL, 31.5 mmol, 2.1 equiv.) was added using a syringe and the septum was replaced with a lid. The mixture was heated from room temperature to 40 °C in a water bath and stirred for 15 min. The solvent was then transferred under reduced pressure into a secondary cooling trap until a precipitate began to form. An argon atmosphere

was re-established in the flask and dry THF (10 mL) was added. The precipitate dissolved and the solvent mixture was distilled off again under reduced pressure into a secondary cold trap until only approximately 2 mL remained and the precipitate had formed again. The lid was replaced with a septum and the solvent was filtered through a cannula into another dry Schlenk flask under slight argon overpressure, such that the formed precipitate remained in the reaction flask. The solid was mixed with fresh, cooled, THF (5 mL) and the solvent was also pressed out through the filter cannula. The solid was then dried under vacuum at 60 °C for at least 12 h. The flask was then transferred to the glovebox and the resulting monopotassium catecholate (**1-K$_1$**, 821 mg, 5.5 mmol, 37%) was obtained as a colorless solid and transferred to a snap cap vial. Due to the considerable air sensitivity of the product which turned dark green upon exposure to ambient air for a few minutes, no further properties of the solid were analyzed apart from the $^{1}H \rightarrow ^{13}C$-CPMAS spectroscopic analysis (see Figure S3).

2.2 Solid-state NMR characterization of catecholate salts

The MAS-NMR rotors were packed in a glovebox under argon atmosphere and Teflon was placed over the samples to further protect them. All ^{13}C-detected solid-state NMR experiments were acquired at 11.7 static magnetic-field strength in wide-bore Bruker standard triple-resonance 3.2 mm and 4 mm probes (for the detailed experimental parameters, see Table S1). All spectra were processed with the software Topspin (versions 3.6.4 and 4.1.3, Bruker Biospin). Chemical-shift values were referenced to TMS, making use of an external calibration on the ^{13}C-detected spectrum of adamantane recorded in the same probe directly before the measurements. For all spectra, zero filling was applied, up to double the amount of acquired data points. All experiments were recorded with temperature control, setting the VTU target temperature to 270 K for NaOMe and KOMe samples, and 280 K for all other measurements.

Table S1: Overview of experimental parameters for solid-state NMR data.

Sample	1	1-Li_2	1-Na_2	1-K_2	1-Cs_2	1-Na_1	1-K_1	1-K_{1+2}	KOMe	NaOMe
**Experiment**					**^1H-^{13}C-CPMAS**					
ν_r / kHz	20	12	12	17	12	12	12	12	17	17
B_0 / T	11.7	11.7	11.7	11.7	11.7	11.7	11.7	11.7	11.7	11.7
**Transfer I**					**^1H-^{13}C-CP**					
$\nu_1(^1H)$ / kHz	65	50	50	50	50	50	50	50	60	60
$\nu_1(X)$ / kHz	45	40	40	40	40	40	40	40	43	43
Shape					Tangent shape					
^{13}C carrier / ppm	134.6	99.6	99.6	103	99.6	99.6	99.6	99.6	58.6	58.6
CP contact time / ms	1.5	2.5	2.5	4	2.5	2.5	4	2.5	1.0	3.0
t_1 increments	3072	3072	3072	3072	3072	3072	3072	3072	3072	4096
Sweep width (t_1) / ppm	795	710	710	795	710	710	710	710	795	795
Acquisition time (t_1) / ms	15.36	17.2	17.2	15.4	17.2	17.2	17.2	17.2	15.5	20.5
^1H Spinal64 decoupling / kHz	90	70	70	90	70	70	90	70	90	90
Interscan delay / s	40	40	40	40	40	40	40	40	3	25
Number of scans	1312	1331	1331	864	1331	1331	256	1331	18000	344

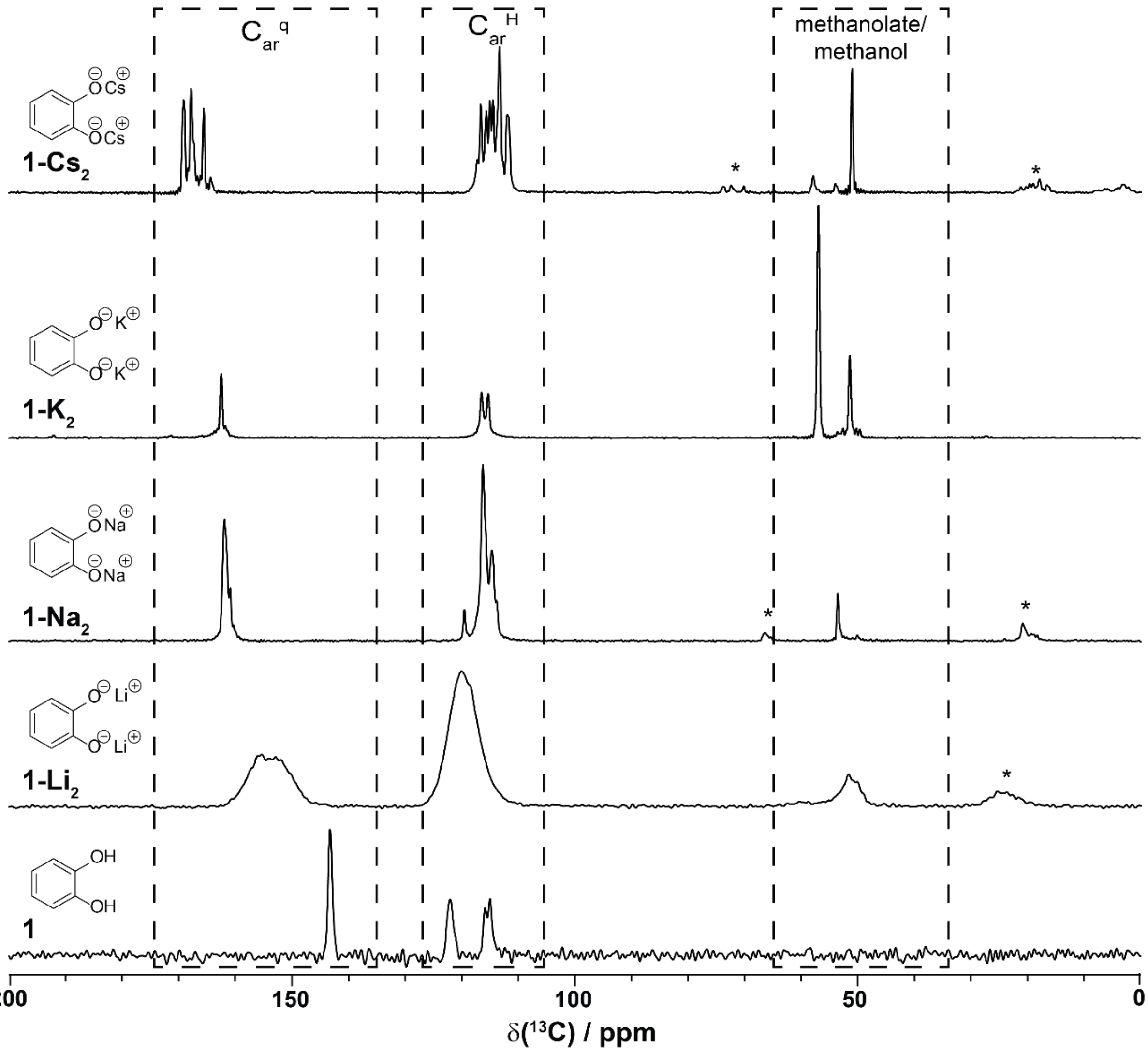

Figure S1: Comparison of the ^{1}H-^{13}C CP-MAS spectra of catechol (1, bottom) and the prepared dialkali metal catecholates, (1-Li_2, 1-Na_2, 1–K_2, 1-Cs_2), * indicate MAS sidebands. Resonance assignments are given in the Figure. All spectra have been recorded at a static magnetic-field strength of 11.7 T and MAS frequencies ranging in-between 12 to 20 kHz.

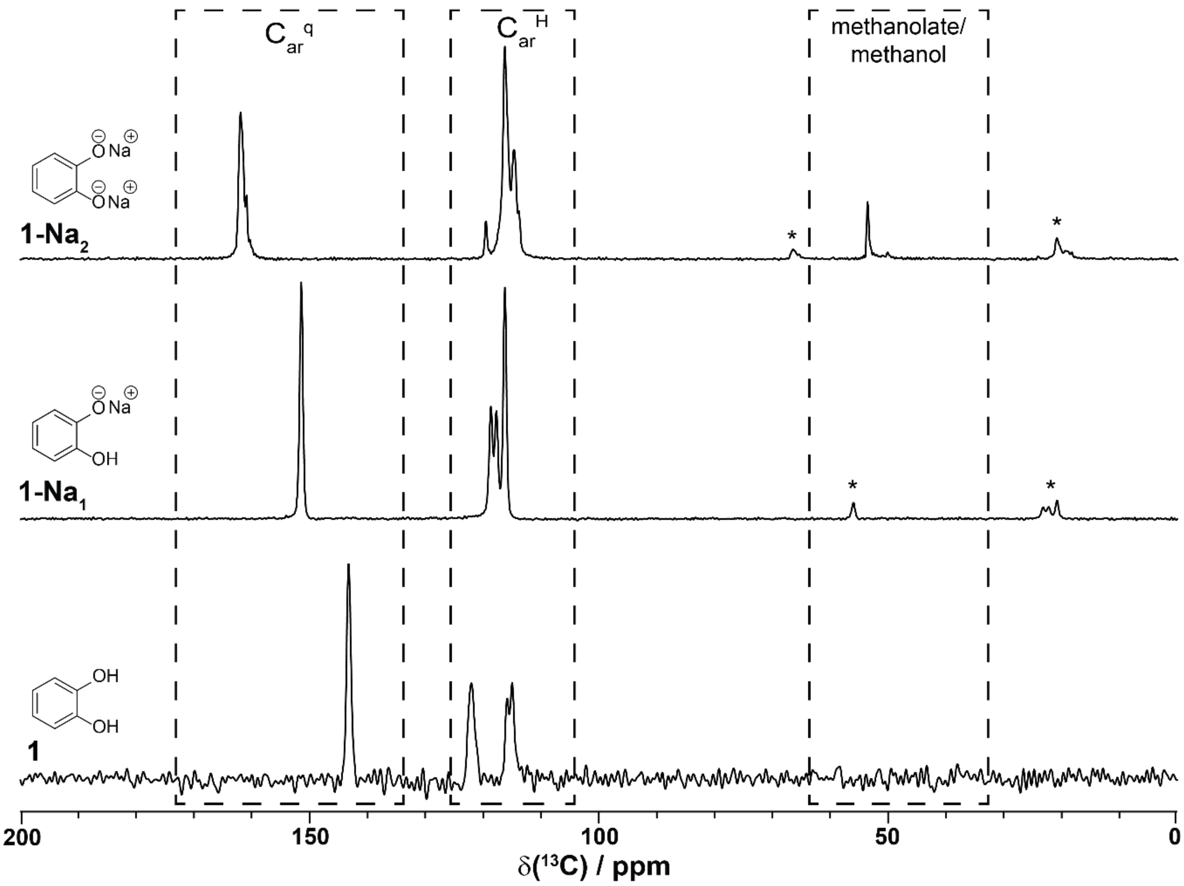

Figure S2: Comparison of the ^{1}H-^{13}C CPMAS spectra of catechol (1, bottom), the prepared monosodium catecholate (1-Na_1) and the prepared disodium catecholate (1-Na_2), * indicate MAS sidebands. Resonance assignments are given in the Figure. All spectra have been recorded at a static magnetic-field strength of 11.7 T and MAS frequencies ranging in-between 12 and 20 kHz.

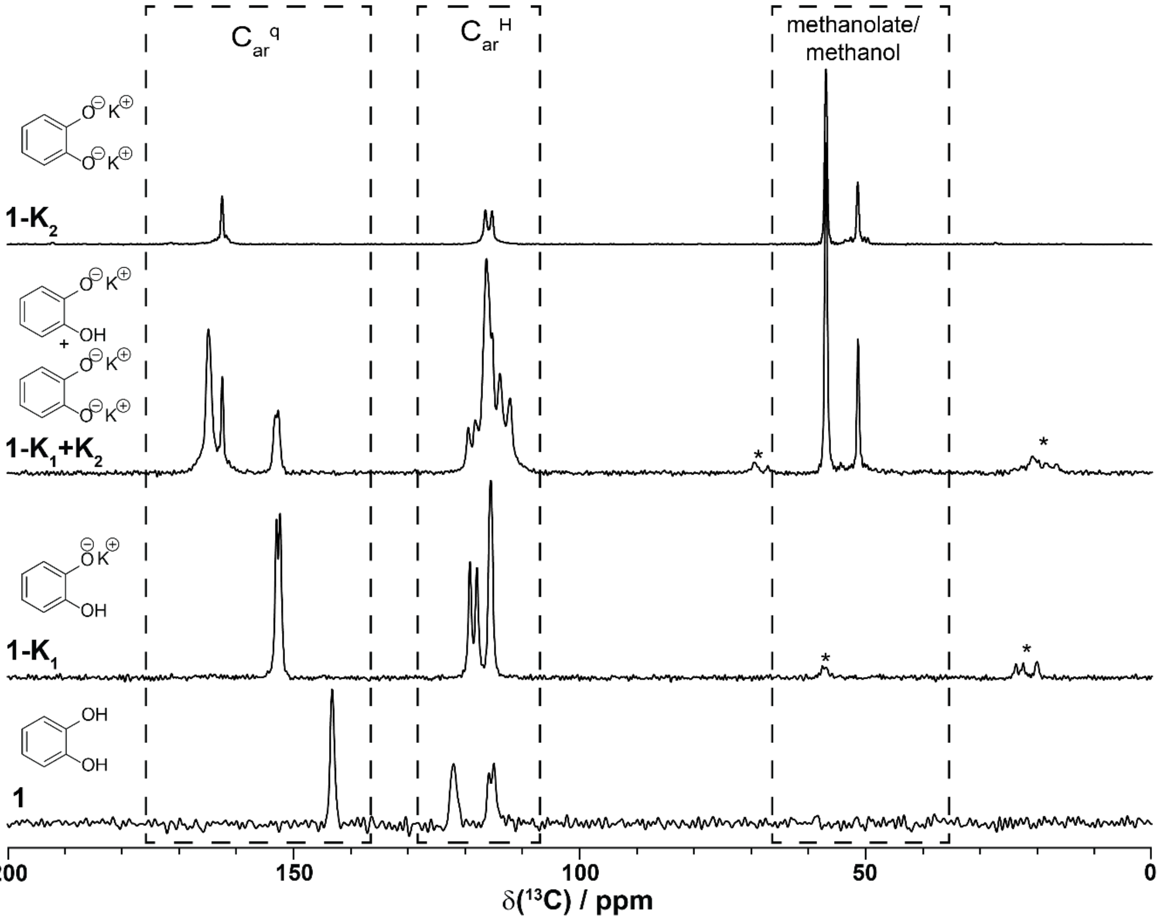

Figure S3: Comparison of the ^{1}H-^{13}C-CP-MAS spectra of catechol (1, bottom), the prepared monopotassium catecholate (1-K_1), the mixture containing both mono- and dipotassium catecholate (1-K_{1+2}) and the prepared dipotassium catecholate (1-K_2), * indicate MAS sidebands. All spectra have been recorded at a static magnetic-field strength of 11.7 T and MAS frequencies ranging in-between 12 and 17 kHz.

Table S2: ^{13}C chemical-shift values for the different catecholates reported in ppm.

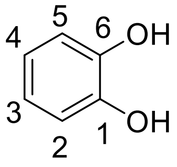

Carbon no.	1	1-Li_2[a]	1-Na_2	1-K_2	1-Cs_2
**C2-C5**	115.1<br>115.9<br>122.0<br>122.4	119.9	113.9<br>114.7<br>116.2	116.4<br>115.3	111.0 -<br>118.1
**C1, C6**	143.3	155.4	160.9<br>161.9	162.5	163.8 -<br>169.7
**methanol/ alkali methanolate**		51.6	53.5	51.4<br>56.9	51.0

[a]: Due to the broad resonances, the respective peak maximum was selected.

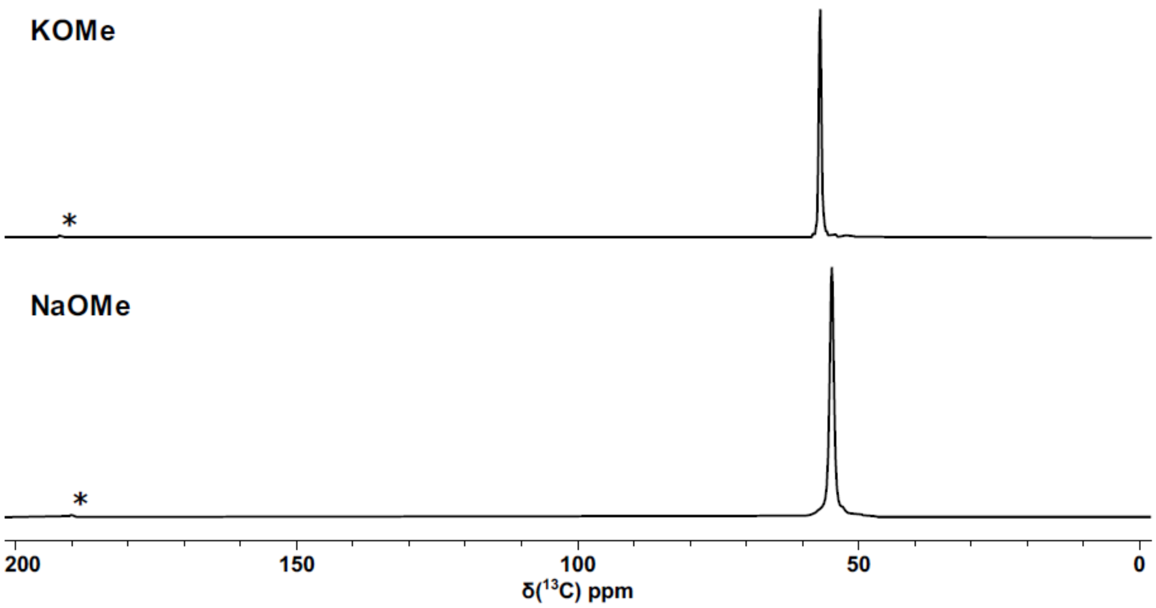

Figure S4: ^{1}H-^{13}C-CP-MAS spectra of KOMe and NaOMe, * indicate MAS sidebands. All spectra have been recorded at a static magnetic-field strength of 11.7 T and MAS frequencies ranging in-between 12 and 17 kHz.

3. Synthetic procedures and characterization of the mechanochemical carboxylation reaction

3.1 Initial experiments

In initial test reactions, the carboxylation of 4-methylcatechol (**1-Me**) was tested under *in-situ* deprotonation (Marasse-type conditions) with bases added prior to ball milling rather than synthesizing the dialkali metal catecholate upfront. This approach led to no conversion of the starting material after aqueous acidic work-up (see Tables S3 and S4).

Table S3: Experiments on the mechanochemical carboxylation of 4-methylcatechol (1-Me) according to the Marasse modification of the Kolbe-Schmitt reaction with dried carbonates and nitrogen bases.

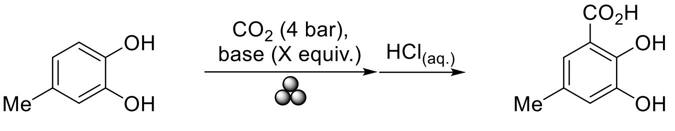

No.	$p(CO_2)$ [bar]	Base	Base [equiv.]	Grinding conditions *f* [rpm]	*t* [min]	Yield [%]
1[a]	4	K_2CO_3	3.0	800	240	-
2[b]	4	K_2CO_3	3.0	800	240	-
3	4	K_2CO_3	5.0	800	180	-
4[c]	4	K_2CO_3	5.0	800	180	-
5[a]	4	K_2CO_3	5.0	800	240	-
6[a,d]	4	K_2CO_3	3.0	800	7×180	-
7[b,d]	4	K_2CO_3	3.0	800	7×180	-
8	2	Na_2CO_3	2.2	800	90	-
9	4	Na_2CO_3	2.2	800	240	-
10[a]	4	Na_2CO_3	2.2	800	240	-
11[d]	4	Na_2CO_3	3.0	850	7×180	-
12[a,c]	4	Na_2CO_3	3.0	800	90	-
13[a]	4	Na_2CO_3	3.0	850	240	-
14[a]	4	Na_2CO_3	5.0	850	240	-
15	4	$CaCO_3$	3.0	800	240	-
16	2	DMAP	2.2	800	90	-
17	4	DMAP	3.0	800	90	-
18	2	DBU	2.2	800	90	-
19[d]	4	DBU	3.0	800	7×180	-
20	2	KOH	2.2	800	90	-

[a] 2.0 equiv. of SiO_2 were added as an additive. [b] 4.0 equiv. of SiO_2 were added as an additive. [c] 0.5 equiv. of *n*-Bu_4NBr were added as an additive. [d] Grinding was paused every 180 min for 30 min each.

Table S4: Experiments on the mechanochemical carboxylation of 4-methylcatechol (1-Me) according to the Marasse modification of the Kolbe-Schmitt reaction with dried carbonates.

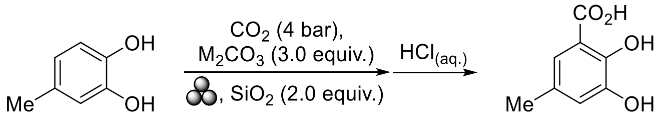

No.	M	SiO_2 [equiv.]	Grinding conditions *f* [rpm]	*t* [min]	Yield [%]
1	K	2.0	800	240	-
2	K	-	800	240	-
3	Cs	2.0	800	240	-
4	Cs	-	800	240	-
5	Na	2.0	800	240	-
6	Na	-	800	240	-

As these attempts to carboxylate catechol moieties remained fruitless, the upfront double deprotonation of both phenolic groups of catechol (**1**) was envisioned to increase the nucleophilicity of the compound sufficiently for a mechanochemical Kolbe-Schmitt-type reaction to occur. The synthesis of the dialkali metal catecholates is described in section 2.1.1.

Firstly, the freshly dried disodium catecholate (**1-Na2**) was tested for the mechanochemical carboxylation using various pressures of carbon dioxide without any additives (Table S5). The ^{1}H NMR spectrum of the crude product showed traces of aromatic compounds other than catechol after four hours of reaction time (Table S5, entry 1). Tripling the reaction time to twelve hours helped to identify the four different carboxylic acids **2a**, **2b**, **2c**, and **2d** besides catechol (**1**) in the ^{1}H NMR spectrum of the product mixture (Table S5, entries 2 and 3), as proven in section 3.2.

Table S5: Experiments on the Kolbe-Schmitt-type mechanochemical carboxylation of disodium catecholate (1-Na2).

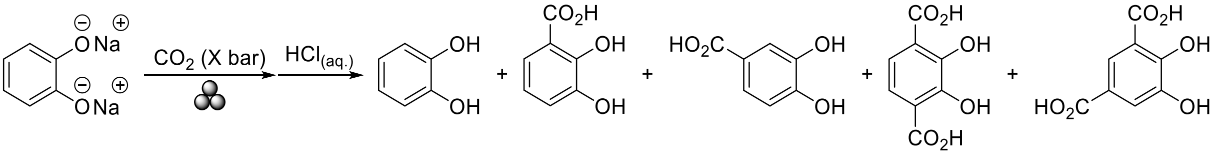

No.	$p(CO_2)$ [bar]	Grinding conditions *f* [rpm]	*t* [min]	NMR Yield[a] **1** [%]	**2a** [%]	**2b** [%]	**2c** [%]	**2d** [%]
1	4	800	240	n. d.[b]	3	-	-	-
2[c]	4	800	4×180	21	33	26	11	10
3[c]	8	800	4×180	16	32	27	13	16

[a] Identification by NMR and quantification by quantitative NMR with 1,3,5-trimethoxybenzene as internal standard. Reference compounds were commercially purchased (**2a**, **2b**, **2c**) or synthesized (**2d**). Synthesis procedure and full characterization of the latter can be found in section 12.7 (AGO-125). [b] Not determined. [c] Grinding was paused every 180 min for 30 min, respectively.

3.2 Identification of the obtained reaction products

The mechanochemical carboxylation of disodium catecholate (**1-Na₂**) delivers a mixture of four carboxylated catechol analogues. The regioisomers were identified by comparing the ^{1}H and $^{13}C\{^{1}H\}$ NMR spectra of commercial compounds 2,3-dihydroxybenzoic acid (**2a**), 3,4-dihydroxybenzoic acid (**2b**), and 2,3-dihydroxyterephthalic acid (**2c**), and synthesized compound 4,5-dihydroxyisophthalic acid (**2d**) (Figure S8-S19; see section 12.7 for the synthetic route towards **2d** as this is an entirely new compound) with those of the ball mill mixture (Figure S5-S7). A visual comparison of the ^{1}H NMR spectra of **2a**-**2d** with that of the ball mill mixture is shown in Figure S20, while the ^{1}H and $^{13}C\{^{1}H\}$ NMR signals are listed in Table S6-S7.

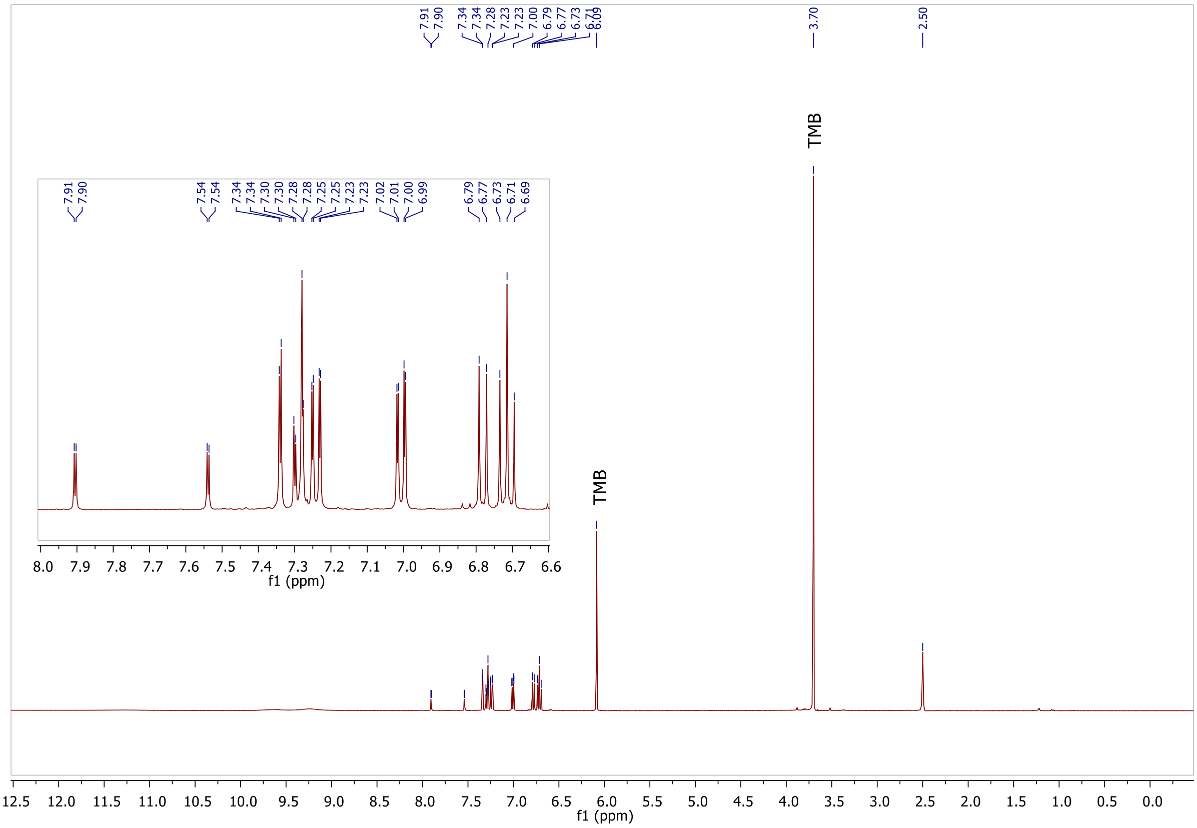

Figure S5: ^{1}H NMR (400 MHz, DMSO-d_6) spectrum of the ball mill mixture, admixed with 1,3,5-trimethoxybenzene.

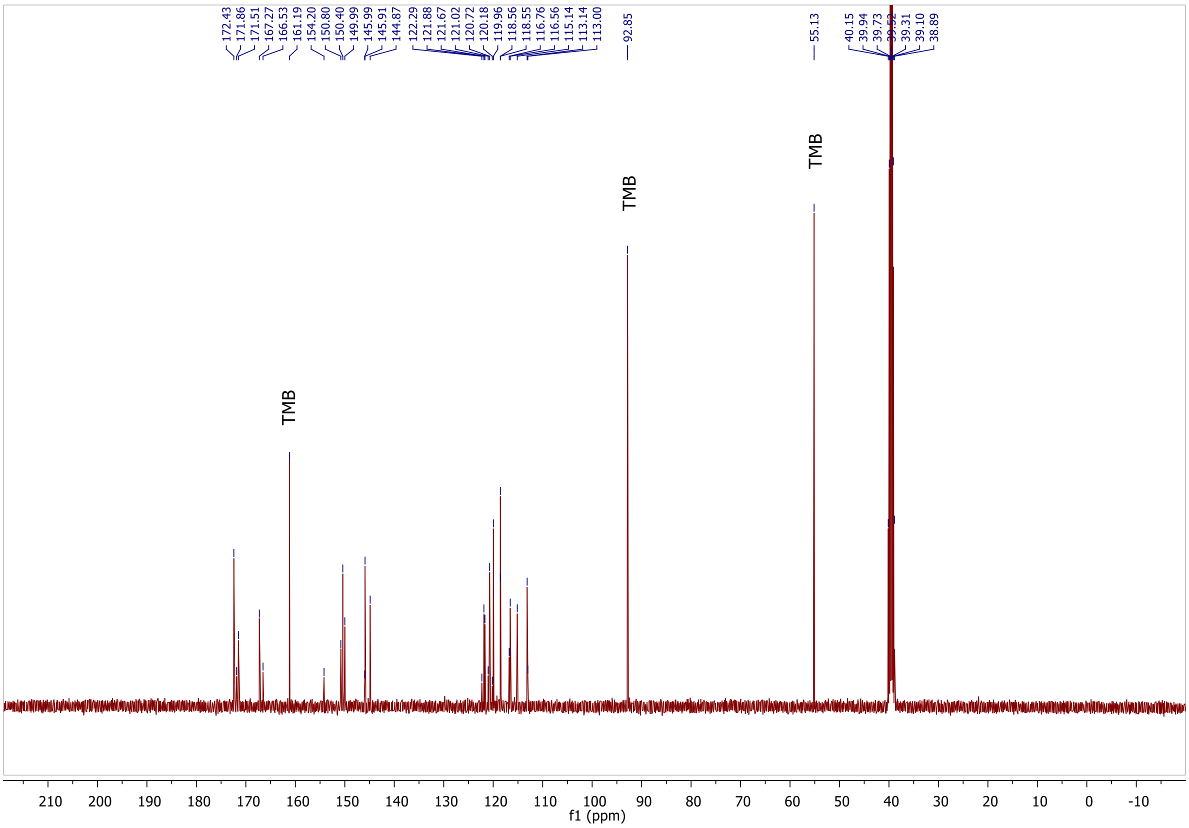

Figure S6: $^{13}C\{^{1}H\}$ NMR (101 MHz, DMSO-d_6) spectrum of the ball mill mixture, admixed with 1,3,5-trimethoxybenzene.

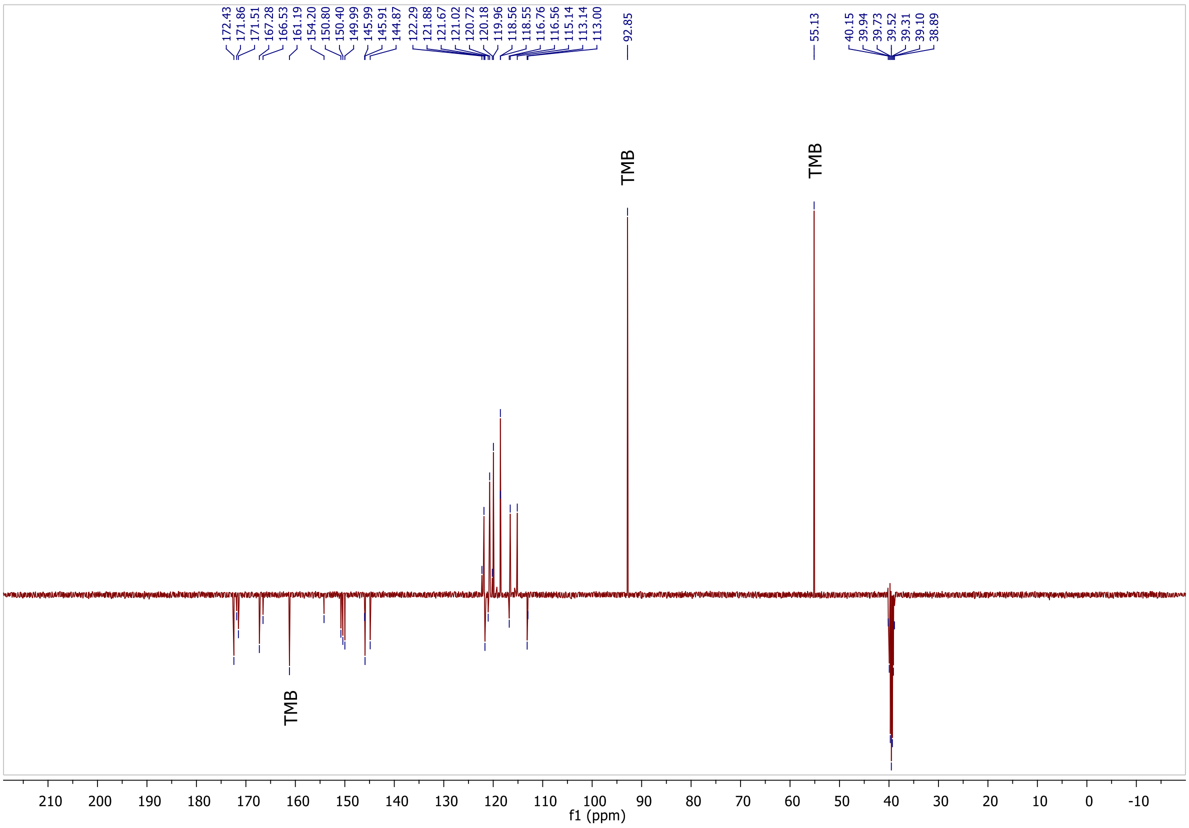

Figure S7: ^{13}C APT NMR (101 MHz, DMSO-d_6) spectrum of the ball mill mixture, admixed with 1,3,5-trimethoxybenzene.

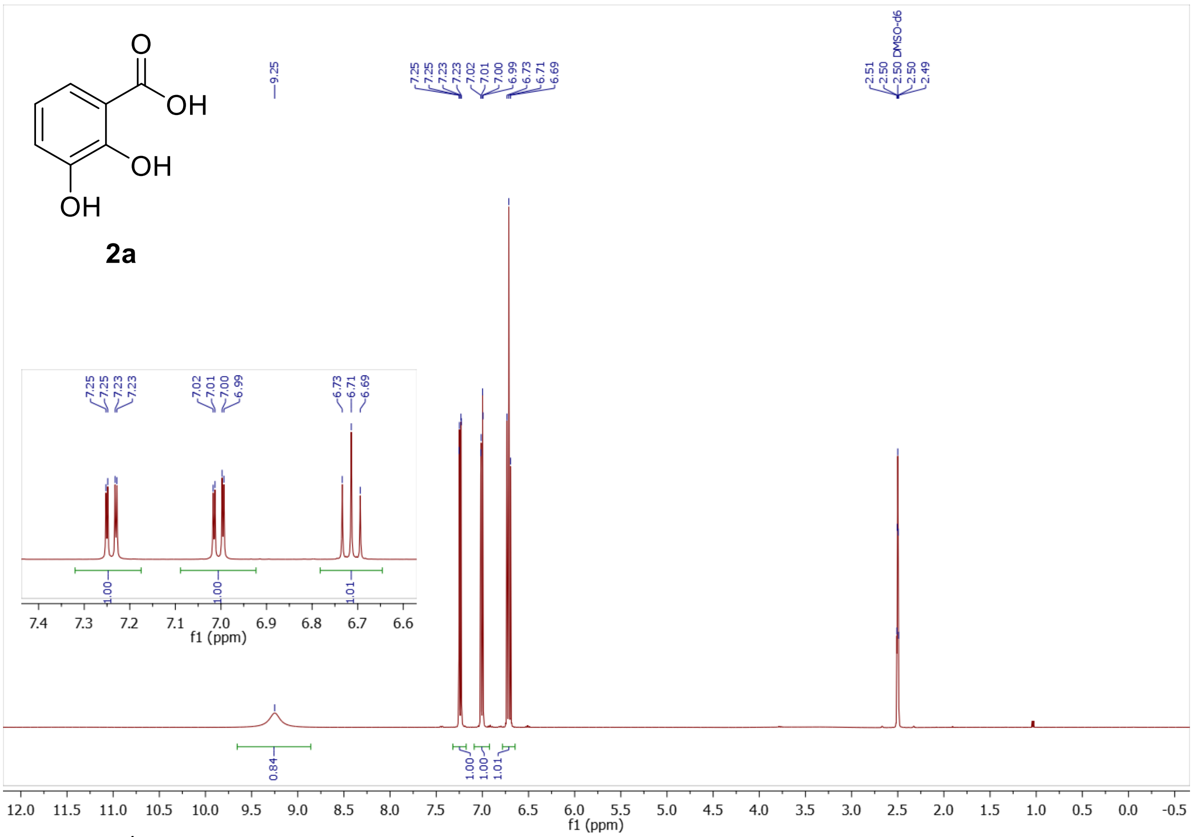

Figure S8: ^{1}H NMR (400 MHz, DMSO-d_6) spectrum of commercial 2,3-dihydroxybenzoic acid (2a).

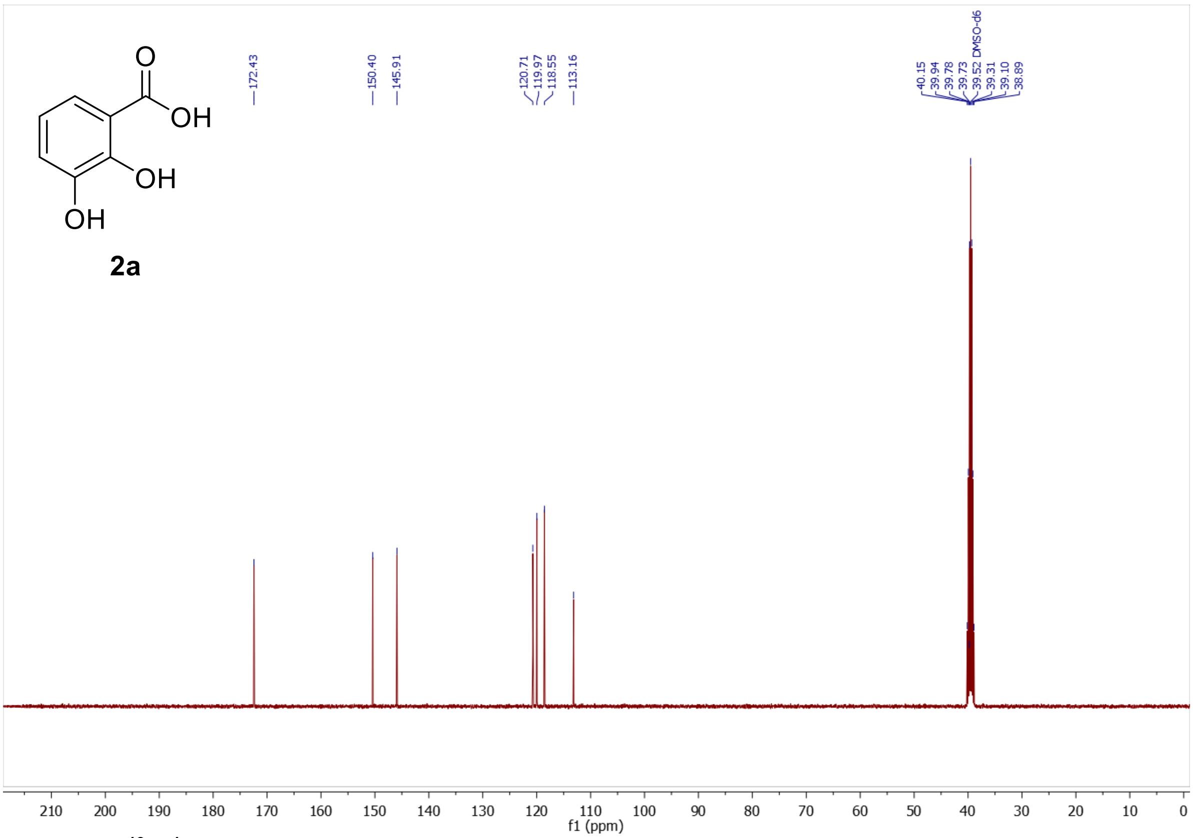

Figure S9: $^{13}C\{^1H\}$ NMR (101 MHz, DMSO-d_6) spectrum of commercial 2,3-dihydroxybenzoic acid (2a).

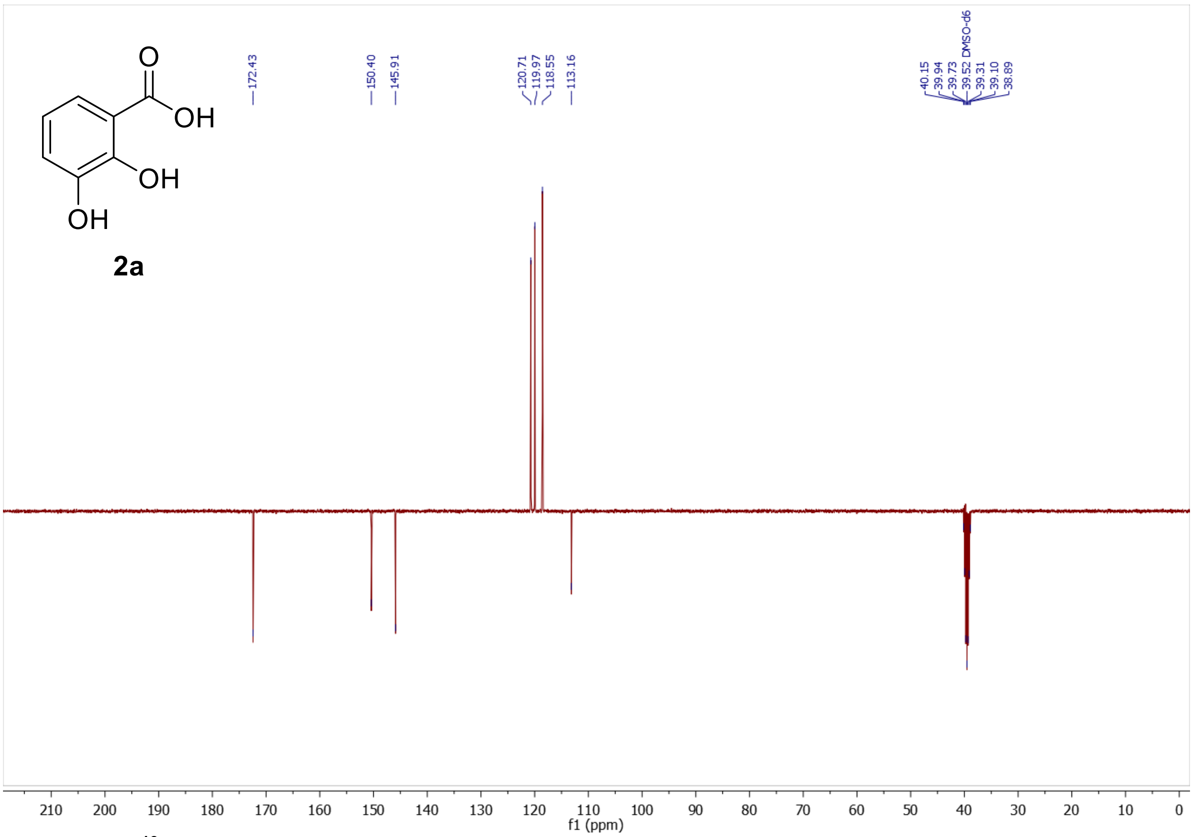

Figure S10: ^{13}C APT NMR (101 MHz, DMSO-*d*$_6$) spectrum of commercial 2,3-dihydroxybenzoic acid (2a).

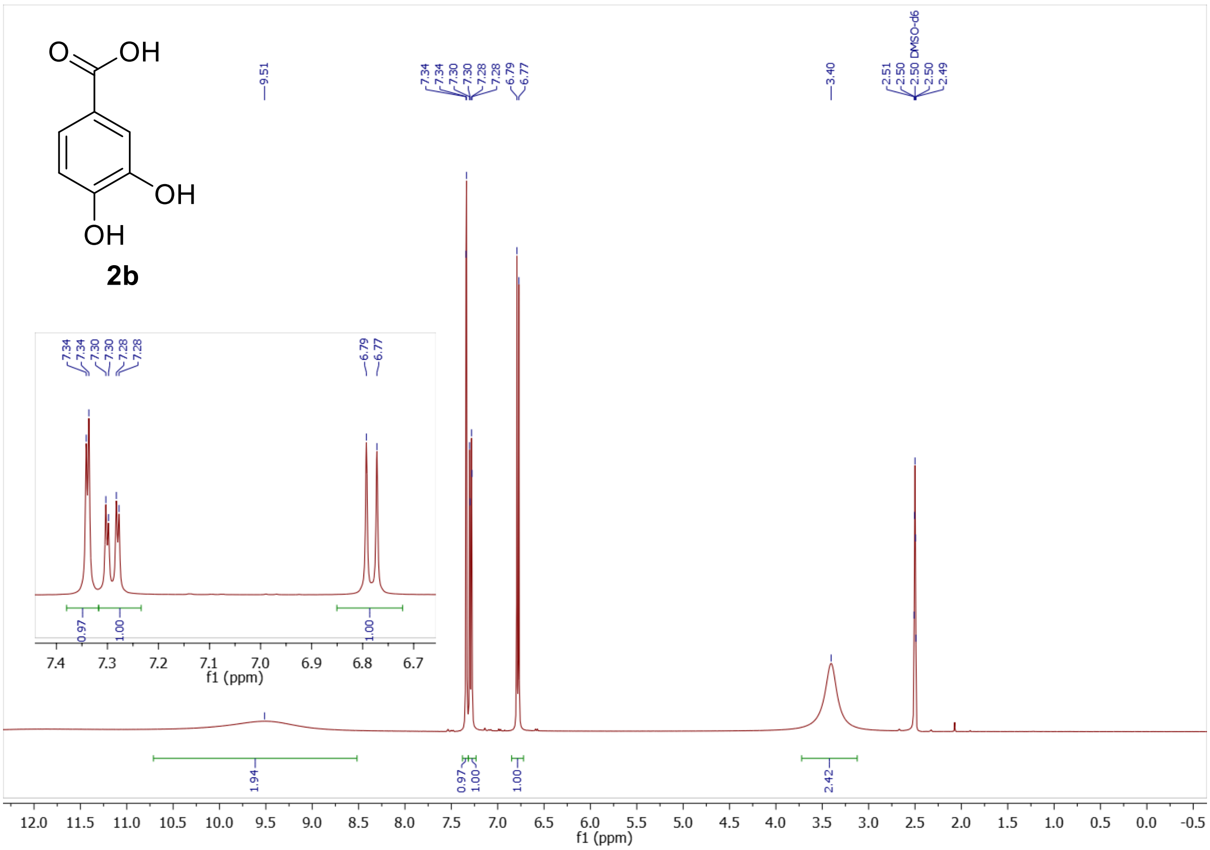

Figure S11: ^{1}H NMR (400 MHz, DMSO-*d*$_6$) spectrum of commercial 3,4-dihydroxybenzoic acid (2b).

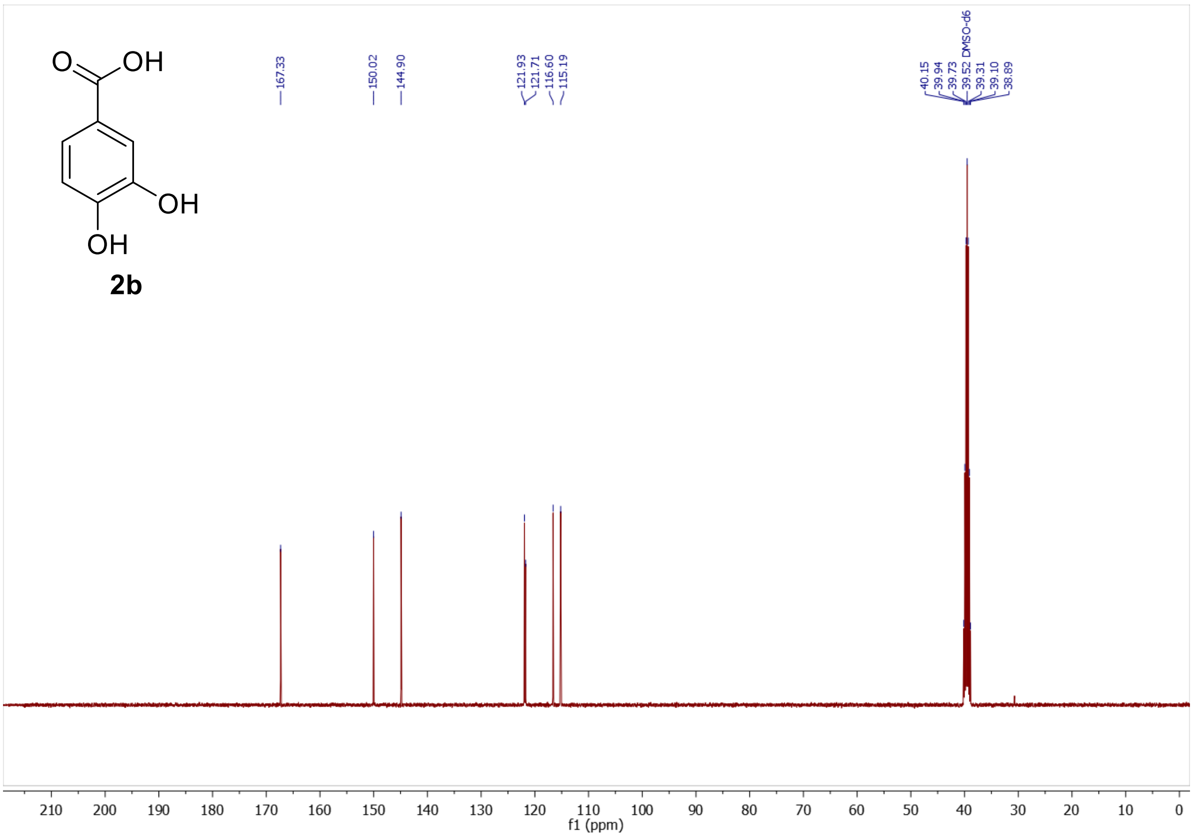

Figure S12: $^{13}C\{^1H\}$ NMR (101 MHz, DMSO-*d6*) spectrum of commercial 3,4-dihydroxybenzoic acid (2b).

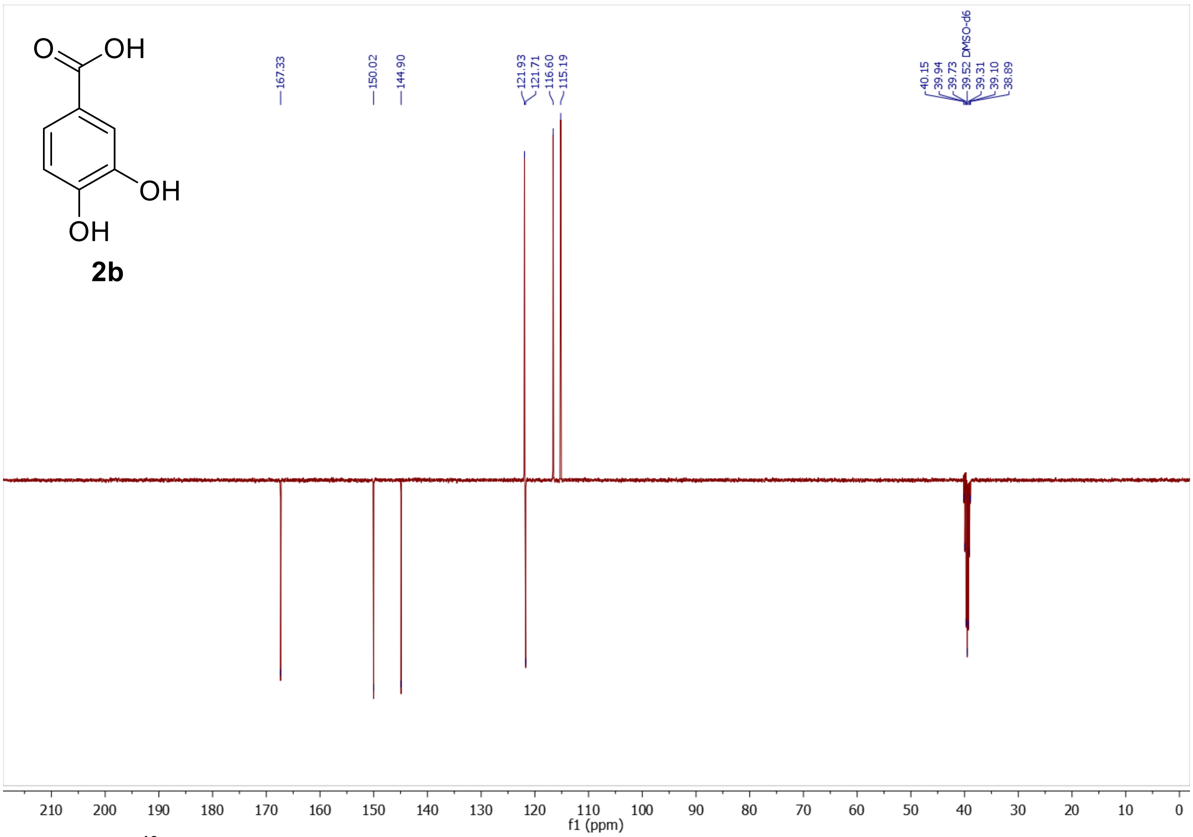

Figure S13: ^{13}C APT NMR (101 MHz, DMSO-*d6*) spectrum of commercial 3,4-dihydroxybenzoic acid (2b).

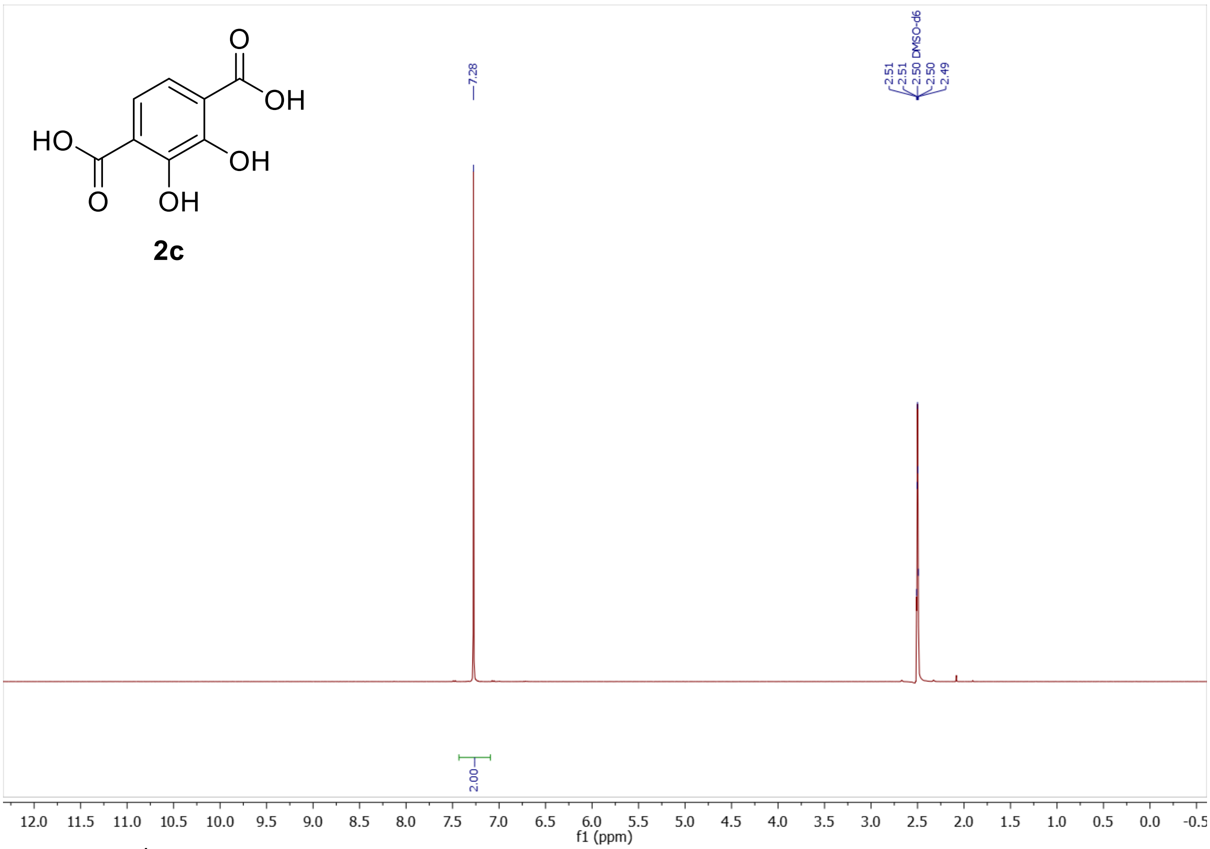

Figure S14: ^{1}H NMR (400 MHz, DMSO-*d*$_6$) spectrum of commercial 2,3-dihydroxyterephthalic acid (2c).

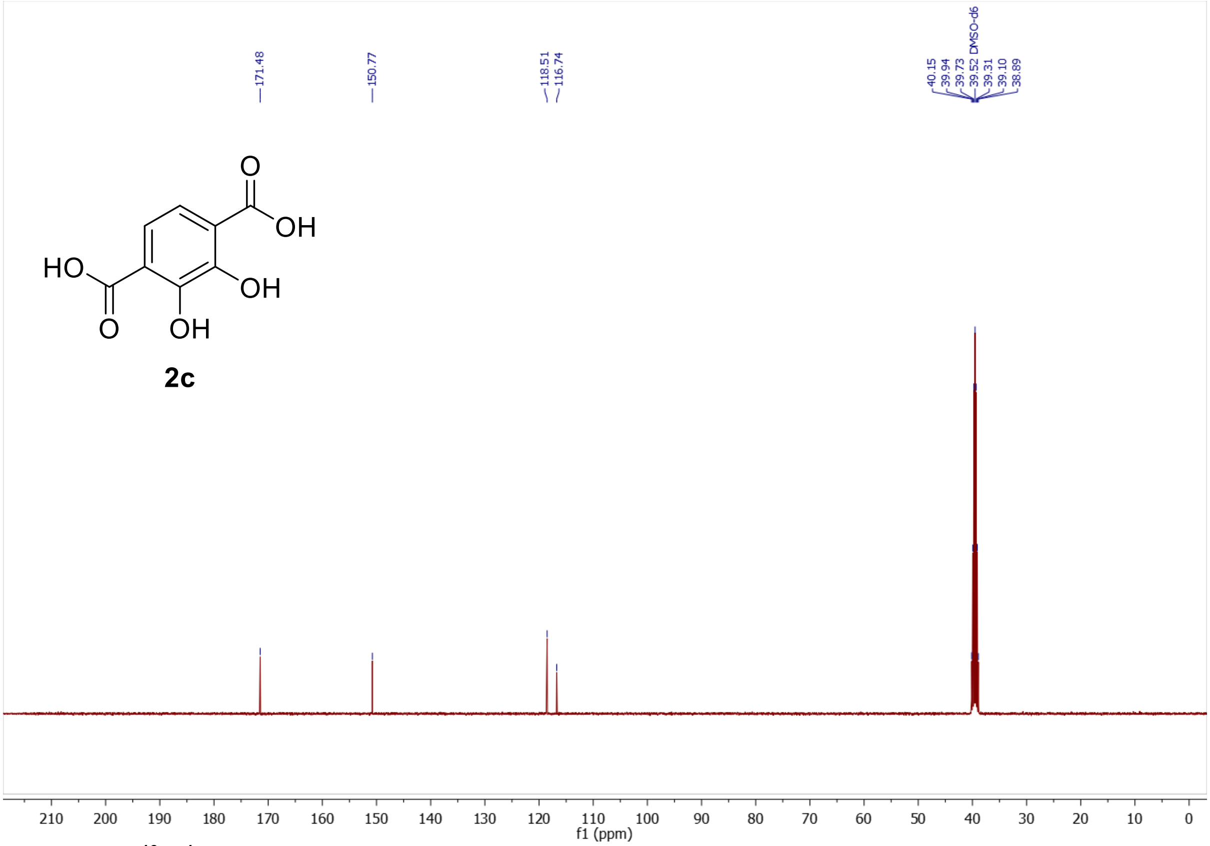

Figure S15: ^{13}C{^{1}H} NMR (101 MHz, DMSO-*d*$_6$) spectrum of commercial 2,3-dihydroxyterephthalic acid (2c).

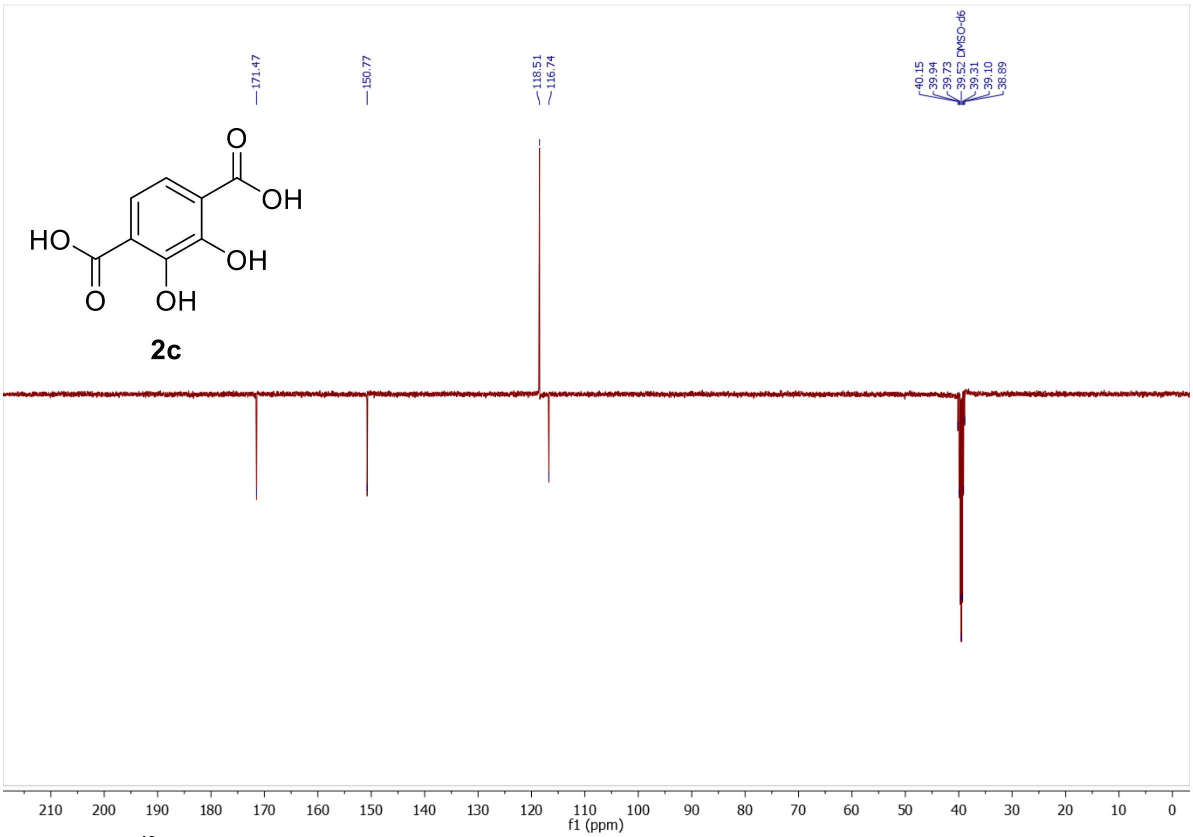

Figure S16: ^{13}C APT NMR (101 MHz, DMSO-d_6) spectrum of commercial 2,3-dihydroxyterephthalic acid (2c).

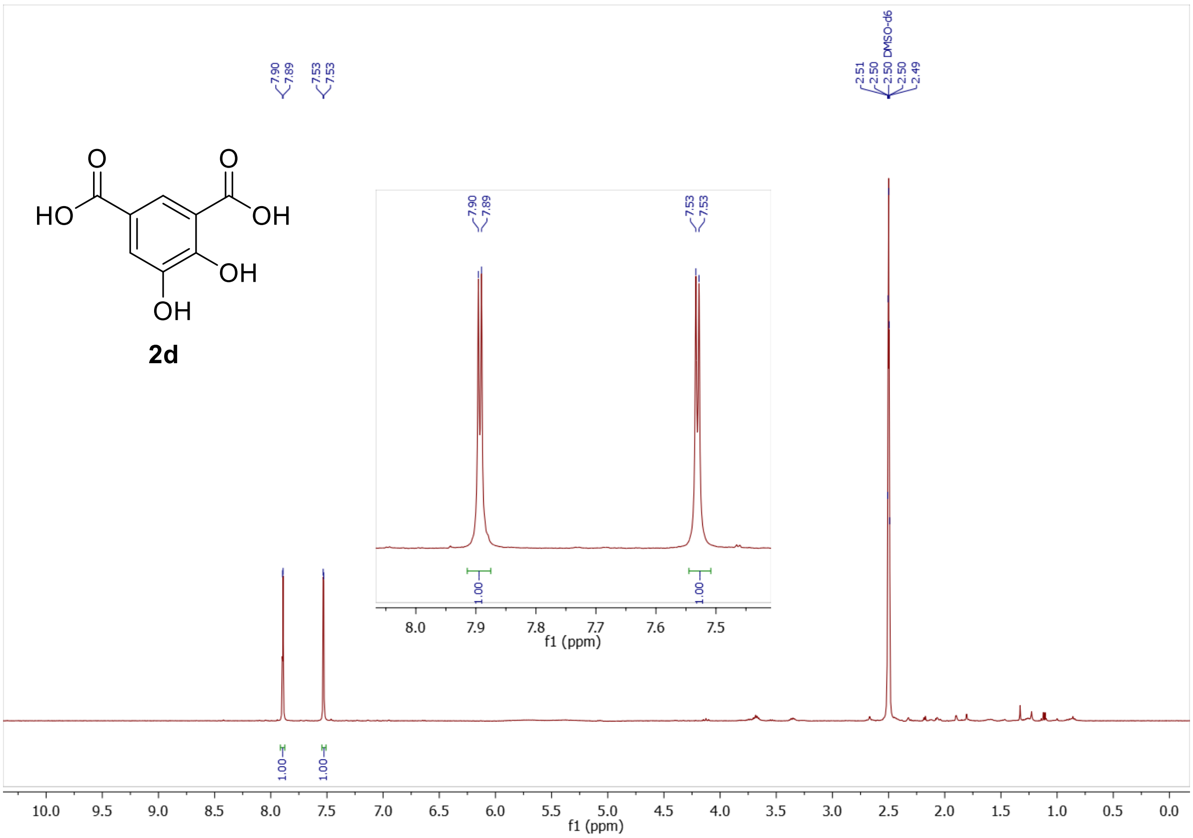

Figure S17: ^{1}H NMR (400 MHz, DMSO-d_6) spectrum of 4,5-dihydroxyisophthalic acid (2d) [AGO-125].

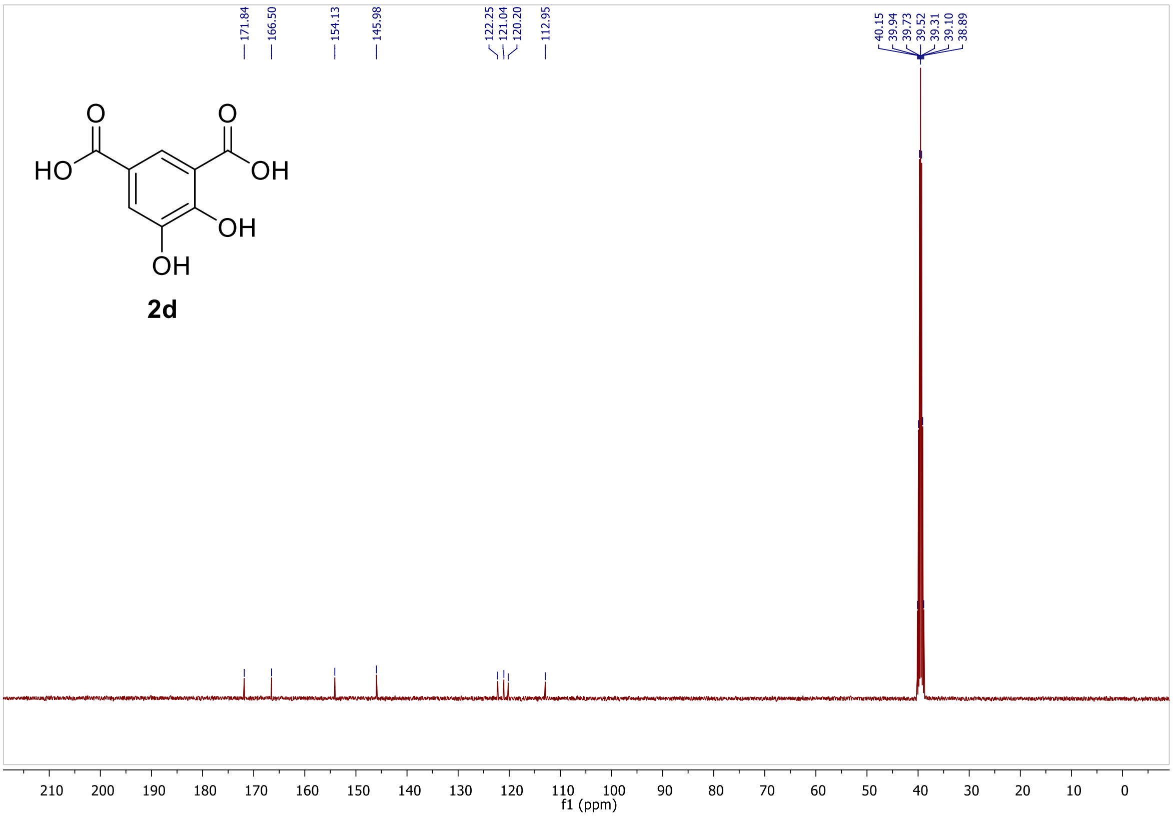

Figure S18: $^{13}C\{^{1}H\}$ **NMR (101 MHz, DMSO-*d*$_6$) spectrum of 4,5-dihydroxyisophthalic acid (2d) [AGO-125].**

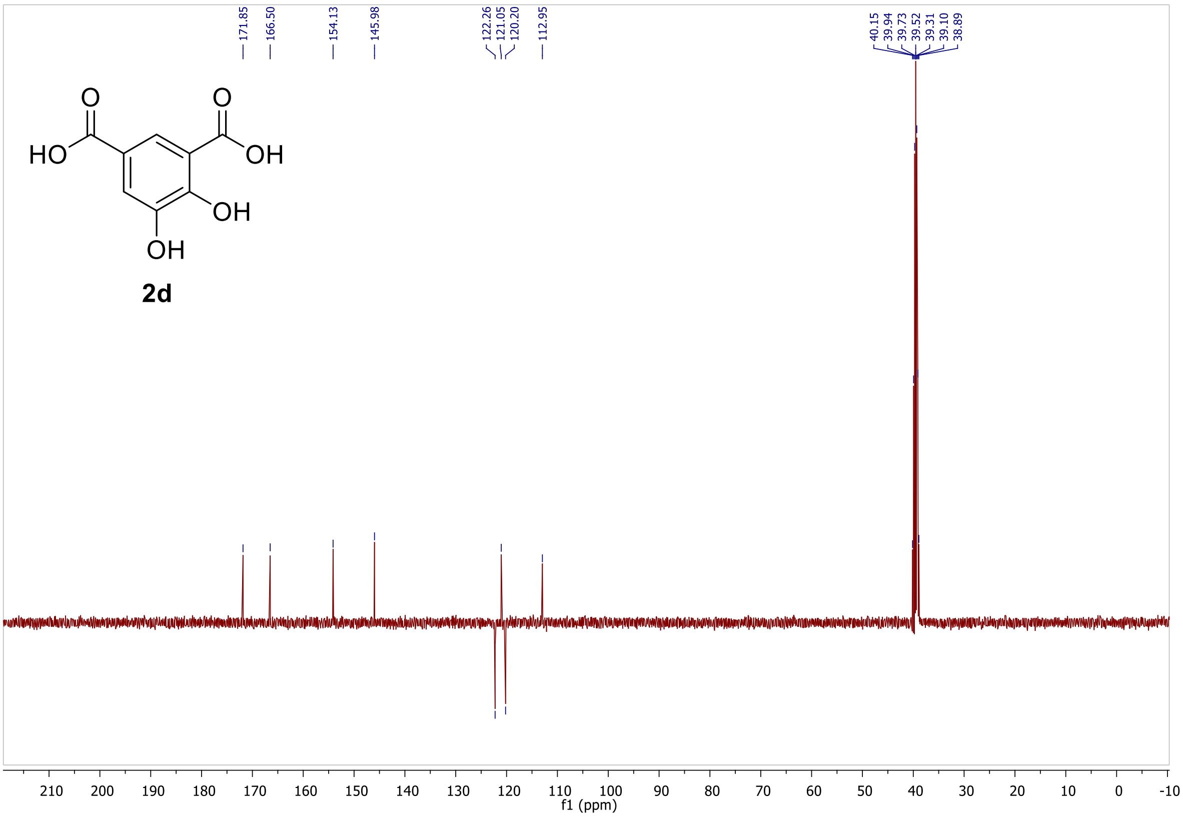

Figure S19: ^{13}C **APT NMR (101 MHz, DMSO-*d*$_6$) spectrum of 4,5-dihydroxyisophthalic acid (2d) [AGO-125].**

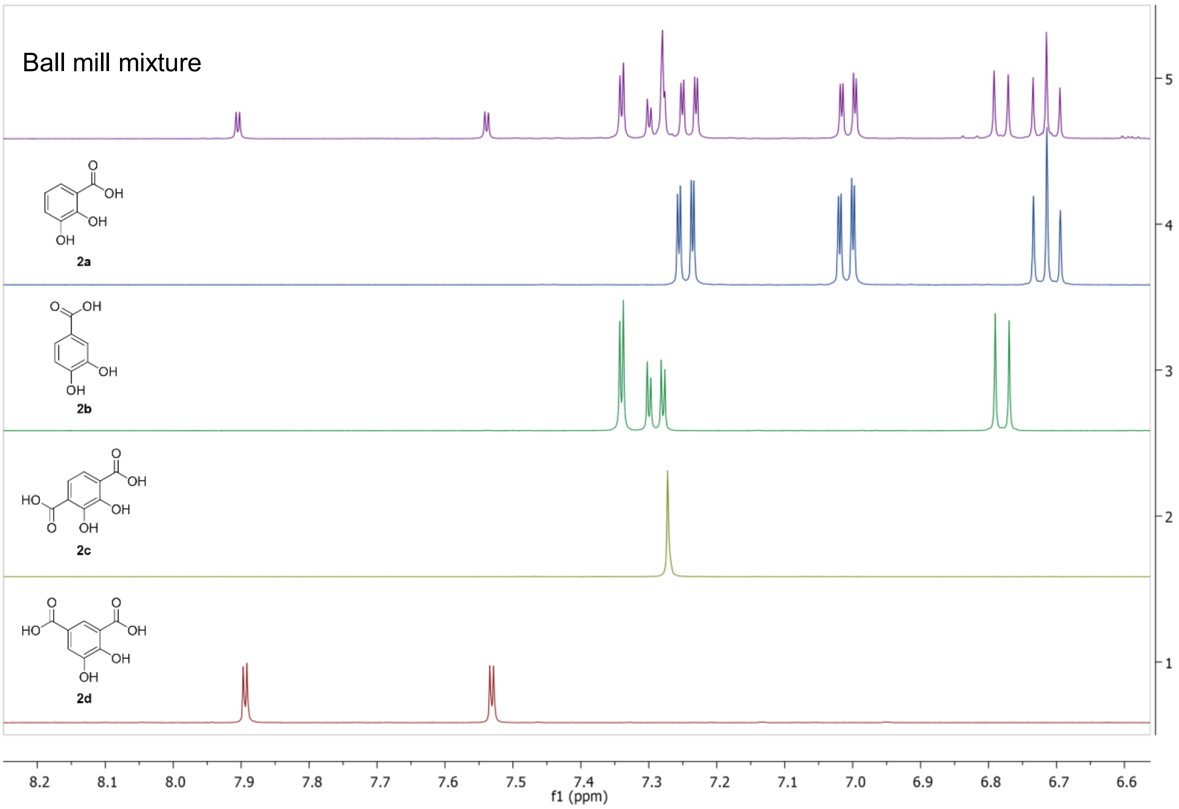

Figure S20: Comparison between the aromatic region of the ^{1}H NMR spectra of the ball mill mixture and reference compounds 2a-2d (400 MHz, DMSO-d_6).

Table S6: Comparison between the aromatic ^{1}H NMR signals of the ball mill mixture and those of reference compounds 2a-2d.[a]

δ ^{1}H (ppm) ball mill mixture	δ ^{1}H (ppm) 2a	δ ^{1}H (ppm) 2b	δ ^{1}H (ppm) 2c	δ ^{1}H (ppm) 2d
6.71 (t, *J* = 7.9 Hz)	6.71 (t, *J* = 7.9 Hz)			
6.78 (d, *J* = 8.2 Hz)		6.78 (d, *J* = 8.2 Hz)		
7.01 (dd, *J* = 7.8, 1.6 Hz)	7.01 (dd, *J* = 7.8, 1.6 Hz)			
7.24 (dd, *J* = 8.0, 1.6 Hz)	7.24 (dd, *J* = 8.0, 1.6 Hz)			
7.28 (s)			7.28 (s)	
7.29 (dd, *J* = 8.1, 2.1 Hz)		7.29 (dd, *J* = 8.2, 2.1 Hz)		
7.34 (d, *J* = 2.1 Hz)		7.34 (d, *J* = 2.1 Hz)		
7.54 (d, *J* = 2.1 Hz)				7.53 (d, *J* = 2.1 Hz)
7.90 (d, *J* = 2.1 Hz)				7.89 (d, *J* = 2.1 Hz)
**Total: 9 signals**	**Total: 3 signals**	**Total: 3 signals**	**Total: 1 signal**	**Total: 2 signals**

[a] DMSO-d_6 peak is set at 2.50 ppm.

Table S7: Comparison between the aromatic $^{13}C\{^{1}H\}$ NMR signals of the ball mill mixture and those of reference compounds 2a-2d.[a]

δ ^{13}C (ppm) ball mill mixture	δ ^{13}C (ppm) 2a	δ ^{13}C (ppm) 2b	δ ^{13}C (ppm) 2c	δ ^{13}C (ppm) 2d
113.0 (C)				113.0 (C)
113.1 (C)	113.2 (C)			
115.1 (CH)		115.2 (CH)		
116.6 (CH)		116.6 (CH)		
116.8 (C)			116.7 (C)	
118.6 (CH)	118.6 (CH)			
118.6 (CH)			118.5 (CH)	
120.0 (CH)	120.0 (CH)			
120.2 (CH)				120.2 (CH)
120.7 (CH)	120.7 (CH)			
121.0 (C)				121.1 (C)
121.7 (C)		121.7 (C)		
121.9 (CH)		121.9 (CH)		
122.3 (CH)				122.3 (CH)
144.9 (C)		144.9 (C)		
145.9 (C)	145.9 (C)			
146.0 (C)				146.0 (C)
150.0 (C)		150.0 (C)		
150.4 (C)	150.4 (C)			
150.8 (C)			150.8 (C)	
154.2 (C)				154.1 (C)
166.5 (C)				166.5 (C)
167.3 (C)		167.3 (C)		
171.5 (C)			171.5 (C)	
171.9 (C)				171.9 (C)
172.4 (C)	172.4 (C)			
**Total: 26 signals**	**Total: 7 signals**	**Total: 7 signals**	**Total: 4 signals**	**Total: 8 signals**

[a] DMSO-d_6 peak is set at 39.52 ppm.

3.3 Optimization of the mechanochemical carboxylation

With appropriate means for quantifying the results of the carboxylation reactions in hand, the ball milling parameters were optimized to ensure the maximum conversion of the catecholate towards carboxylic acids. Starting from the conditions in which all four acids **2a-d** had been formed in detectable amounts (Table S8, entry 2), the first successful approach to increase their yields was to further extend the reaction time to a total of 18 hours with the renewal of the CO_2 atmosphere inside the grinding bowl after the first three hours (Table S8, entry 4). An increase of the pressure to 8 bar had not improved the reaction outcome (Table S8, entry 3), and neither did a higher milling frequency of 850 rpm increase the yields of the acids **2a-d** (Table S8, entry 5). While adding more balls of the same size as in previous reactions only gave lower yields (Table S8, entry 6), the replacement of these balls with the equivalent weight of smaller balls to encourage better mixing even resulted in the formation of a black, soot-like crude product in which barely any acids were detected (Table S8, entry 7). These observations paralleled earlier experiences of the authors where an excessive energy input was considered destructive for freshly formed carboxylates.[4] However, the reduction of the milling frequency to 750 rpm gave the best results so far (Table S8, entry 8), with a combined NMR yield of 82% for the four acids **2a-d**. A further decrease to 700 rpm resulted in a slight decrease in yields (Table S8, entries 9 and 10), while at a frequency of 400 rpm, only small amounts of the acids were formed (Table S8, entry 11).

Table S8: Optimization of the grinding conditions for the mechanochemical carboxylation of disodium catecholate (1-Na$_2$).[a]

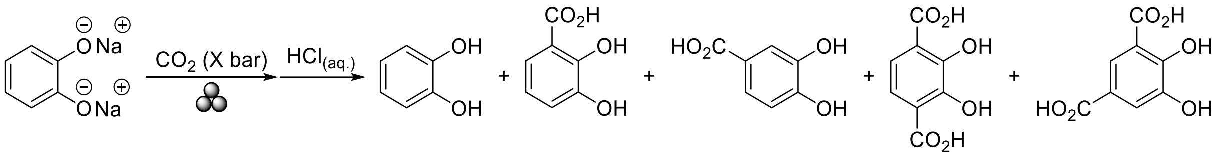

1-Na$_2$ **1** **2a** **2b** **2c** **2d**

Entry	n(1-Na$_2$) [mmol]	Grinding conditions f [rpm]	t [min]	1 [%]	NMR Yield[b] 2a [%]	2b [%]	2c [%]	2d [%]	Σ(2a-d) [%]
1	1.5	800	240	n. d.[c]	3	-	-	-	-
2	1.5	800	3×240	17	26	18	13	6	63
3[d]	1.5	800	3×240	13	26	21	11	11	69
4	1.5	800	6×180	20	32	23	9	5	69
5	1.5	850	6×180	20	24	18	5	5	52
6[e]	1.5	850	6×180	11	22	22	8	12	64
7[f]	1.5	850	6×180	8	7	7	1	3	18
8	1.5	750	6×180	19	33	26	13	10	82
9	1.5	700	6×180	37	33	20	7	4	64
10	2.0	700	6×180	56	23	13	2	-	38
11	1.5	400	6×180	62	10	7	1	traces	18

[a] Reaction conditions: **1-Na$_2$** was weighed into ZrO_2-M milling vessels with gas valves and 5 ZrO_2-M balls (Ø = 1 cm) inside a glovebox and the vessels were sealed, 4 bar CO_2 were added outside the glovebox and the mixture was milled at the indicated rotational frequency for the indicated time. In the case of multiple cycles, 30 min breaks were taken after each cycle, with a renewal of the CO_2 after the first cycle, followed by aqueous work-

up with HCl. [b] Identification and quantification by quantitative ^1H NMR spectroscopy with 1,3,5-trimethoxybenzene as internal standard. [c] Not determined. [d] Instead of 4 bar, the vessel was filled with 8 bar CO_2-pressure. [e] Ten ZrO_2-M balls (Ø = 10 mm) were used instead of five. [f] Instead of five ZrO_2-M balls (Ø = 10 mm), 80 ZrO_2-Y balls (Ø = 5 mm) were used. After the grinding steps, the reaction mixture was a black, soot-like powder.

According to these results, the carboxylation selectivity of the mechanochemical Kolbe-Schmitt reaction was hardly influenced by the grinding conditions alone as a mixture of four acids was obtained each time. Next the influence of the alkali metal on the preferred carboxylation positions was investigated. For this, the corresponding dialkali metal catecholates **1-M₂** were prepared (see section 2.1.1) using the respective alkali metal methanolates for lithium and potassium catecholates (**1-Li₂** and **1-K₂**) and cesium hydroxide for cesium catecholate (**1-Cs₂**).

Table S9: Use of different dialkali metal catecholates for mechanochemical carboxylation.[a]

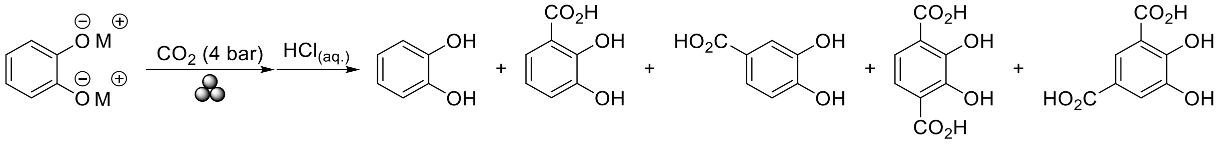

Entry	1-M	*n*(1-M) [mmol]	Grinding conditions *f* [rpm]	*t*[c] [min]	NMR Yield[b] 1 [%]	2a [%]	2b [%]	2c [%]	2d [%]	Σ(2a-d) [%]
1	**1-Cs₂**	1.5	800	6×180	87	6	7	-	-	13
2	**1-Cs₂**	1.0	800	6×180	82	7	11	-	-	18
3	**1-Cs₂**	1.5	850	6×180	99	traces	traces	traces	traces	traces
4	**1-Cs₂**	1.0	850	6×180	84	6	10	-	-	16
5	**1-Cs₂**	1.0	750	6×180	99	traces	traces	traces	traces	traces
6[d]	**1-K₁₊₂**	1.5	800	6×180	99	traces	traces	traces	traces	traces
7[d]	**1-K₁₊₂**	2.0	800	6×180	99	traces	traces	traces	traces	traces
8[d,e]	**1-K₁₊₂**	2.0	800	6×180	100	-	-	-	-	-
9	**1-K₂**	1.5	750	6×180	99	traces	traces	traces	traces	traces
10	**1-Li₂**	2.0	800	6×180	28	38	21	9	4	72
11[f]	**1-Li₂**	2.0	750	6×180	27	37	19	6	4	66
12	**1-Li₂**	2.0	700	6×180	39	32	20	5	4	61

[a] Reaction conditions: **1-M₂** was weighed into ZrO_2-M milling vessels with gas valves and 5 ZrO_2-M balls (Ø = 1 cm) inside a glovebox and the vessels were sealed, 4 bar CO_2 were added outside the glovebox and the mixture was milled at the indicated rotational frequency for 6 x 180 min, with 30 min breaks after each cycle and a renewal of the CO_2 after the first cycle, followed by aqueous work-up with HCl. [b] Identification by NMR and quantification by quantitative NMR with 1,3,5-trimethoxybenzene as internal standard. [c] Milling was paused for 30 min every 3 h (180 min). [d] The spectrum of **1-K₁₊₂** showed the presence of an additional peak which could not be assigned until now (Figure S3). [e] 18-crown-6 (1.0 equiv.) was added. [f] Average over three reactions.

All dialkali metal catecholates were subjected to the mechanochemical carboxylation protocol (Table S9), in most cases prior to their characterization with solid-state NMR, thus before knowing their purity. While with **1-Cs₂**, there seemed to be a slight tendency to form only the monocarboxylated **2a** and **2b** (Table S9, entries 1-5), the conversion was very low in general, independent of the milling frequency. With the first batch of dipotassium catecholate (**1-K₁₊₂**), only traces of the desired carboxylic acids **2a-d** were observed (Table S9, entries 6 and 7). Since the dialkali metal catecholates of the heavier alkali metals naturally had a higher molecular mass, their amount of

substance was varied in the reactions to account for changes due to the volume consumed, but these variations equally showed no effect. The use of the crown ether 18-crown-6 with **1-K$_{1+2}$** did not solve this (Table S9, entry 8) despite the known advantages of forming complexes with potassium ions.[5] Once dipotassium catecholate (**1-K$_2$**) had been synthesized, a test reaction with the salt at optimum conditions only gave traces of the anticipated acids (Table S9, entry 9). Hence, the assumed detrimental effect of the presence of protonated phenolic groups was not confirmed. The reasons for the lack of reactivity could also therefore lie either in the size of the cation or in the presence of excess KOMe that was necessary to ensure complete deprotonation (compare with Table S10). With dilithium catecholate (**1-Li$_2$**), on the other hand, NMR yields of the anticipated carboxylic acids **2a-d** were similar to those obtained with disodium catecholate (**1-Na$_2$**), irrespective of the milling frequency (Table S9, entries 10-12).

3.3.1 Solid-state NMR spectroscopy of dialkali metal catecholates

At this point, the reason behind these differences in reactivity was sought in the characteristic solid-state properties of the dialkali metal catecholates. Due to their enormous sensitivity to ambient air, common analytical methods such as mass spectrometry, infrared spectroscopy or elemental analysis were excluded. However, the rotors for solid-state NMR spectroscopy can be packed under an argon atmosphere inside a glovebox and thus protect the catecholates sufficiently for the duration of the measuring time. For this, ^{1}H-^{13}C cross polarization (CP) and magic angle spinning (MAS), *i.e.* rotation about an axis inclined at the “magic angle” of approximately 54.74° to the external magnetic field, in short, ^{1}H-^{13}C CP-MAS NMR spectroscopy was applied (comparison shown in Figure S1).

In the ^{1}H-^{13}C CP-MAS NMR spectrum of pure catechol (**1**), the three expected signals for the three chemically inequivalent carbon atoms appeared between about 100 ppm and 150 ppm (Figure S1, bottom). The signals with large line widths that appeared for dilithium catecholate (**1-Li$_2$**) could possibly be due to the amorphous nature of the solid, i.e. indicating structural disorder. During the synthesis, the salt accumulated as an oily residue after removal of the solvent, which turned into a foam during drying under vacuum instead of forming a precipitate in methanol like the other salts. Therefore, disodium catecholate (**1-Na$_2$**) as well as dipotassium catecholate (**1-K$_2$**) and dicesium catecholate (**1-Cs$_2$**) each showed much sharper signals. Generally, all four salts showed a set of two signals at 150–170 ppm and 110–120 ppm, where the former corresponds to the two respective quaternary carbon atoms and the latter accommodates the four remaining aromatic carbon atoms, respectively. For all catecholates, one or two signals were also observed at about 50 ppm, indicating the presence of residues of the respective alkali metal methanolate or methanol incorporated in the solid materials.

Since neither **1-Me** nor **1-K$_{1+2}$** underwent carboxylation (see section 3.1 and Table S9), another relevant factor seemed to lie in the protonation state of the catecholate, raising the question whether the complexation of the oxygen atoms with a single alkali metal

cation would be sufficient to activate the aromatic compound for carboxylation. To investigate this hypothesis, monosodium catecholate (**1-Na$_1$**) was synthesized (see section 2.1.2) and characterized with solid-state NMR spectroscopy. According to its ^{1}H-^{13}C CP-MAS NMR spectrum (see Figure S2), **1-Na$_1$** was obtained in a very pure form as expected after precipitating from THF and being washed several times. The signal of the quaternary carbon atoms of **1-Na$_1$** appeared exactly between the corresponding signals of disodium catecholate (**1-Na$_2$**) and catechol (**1**) at about 150 ppm. However, when subjecting this salt to the mechanochemical carboxylation protocol, the ^{1}H NMR spectrum of the crude product showed only catechol (**1**, Scheme S5). The additional proton thus prevented the reaction, reinforcing the finding that the catecholate indeed needs to be deprotonated at both hydroxyl-groups for carboxylation to occur.

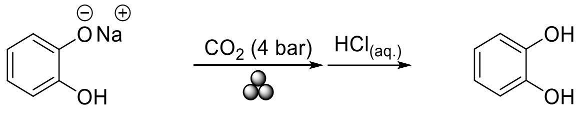

1-Na$_1$ **1**

Scheme S5: Attempted mechanochemical carboxylation of monosodium catecholate (1-Na$_1$).
Reaction conditions: **1-Na$_1$** (1.5 mmol), ZrO_2-M milling vessels with gas valves, 5 ZrO_2-M balls (Ø = 1 cm), 4 bar CO_2, 700 rpm for 6×180 min with 30 min break after each cycle, renewal of CO_2 pressure after the first 180 min.

In the lack of reactivity for **1-Na$_1$** also lies a possible explanation for the missing reactivity for **1-K$_{1+2}$**. As the additional proton prevented the carboxylation for **1-Na$_1$**, the contamination of **1-K$_2$** with **1-K$_1$** could have been one of the reasons for **1-K$_{1+2}$** to also evade carboxylation. However, the preparation of pure **1-K$_2$** was finally achieved with a large excess of KOMe and the mechanochemical Kolbe-Schmitt-type reaction still failed with this fully deprotonated catecholate. Hence, the presence of the additional protons in **1-K$_{1+2}$** seemingly was not the decisive factor. The synthesis of pure **1-K$_1$** ultimately served the purpose of confirming the composition of **1-K$_{1+2}$** through ^{1}H-^{13}C CP-MAS NMR spectroscopy.

3.3.2 Addition of additives and control reactions

Beyond the insights gained through ^{1}H-^{13}C CP-MAS NMR spectroscopy, further attempts were made to optimize the Kolbe-Schmitt-type reaction by using additives (Table S10) while applying the optimum grinding conditions (using 1.5 mmol disodium catecholate **1-Na$_2$**, grinding at 750 rpm for 18 hours with 4 bar CO_2). Similar to the addition of 18-crown-6 to **1-K$_{1+2}$** (Table S9, entry 8), the addition of 15-crown-5 as the more suitable complexing agent for sodium ions equally prevented any carboxylation reactivity (Table S10, entries 1 and 2). Equally, the addition of dimethyl carbonate (DMC) as a liquid CO_2 surrogate (Table S10, entry 4) and the addition of DBU as a captivating agent for CO_2 stopped the carboxylation altogether (Table S10, entry 9). Both the addition of $NaCO_3Me$ (Table S10, entry 3) and Na_2CO_3 (Table S10, entry 5) as solid CO_2 surrogates as used in modifications of the original Kolbe-Schmitt reaction decreased the conversion towards the anticipated carboxylated products **2a-d** with respect to the optimal grinding conditions. Contrary to the assumption that the use of

excess NaOMe might scavenge the protons released during carboxylation (Table S10, entry 6) and thus facilitate an improved reaction outcome, it hindered the reaction from occurring overall, thereby offering an alternative explanation as to why no carboxylation was observed with **1-K2** (which had required an excess of KOMe to be formed in the first place). The alkali metal methanolate could competitively react with CO_2 to form $NaCO_3Me$ which was shown to inhibit the conversion.[6] The tests of degassed Cyrene® and toluene as liquid-assisted grinding agents (LAG agents) showed decreased reactivity for Cyrene® (Table S10, entry 7) and equal reactivity for toluene (Table S10, entry 8) compared to the optimum grinding conditions (Table S8, entry 8). Thus, they were considered superfluous, and their addition was omitted in further investigations.

Table S10: Influence of additives on the mechanochemical carboxylation of disodium catecholate (1-Na2).[a]

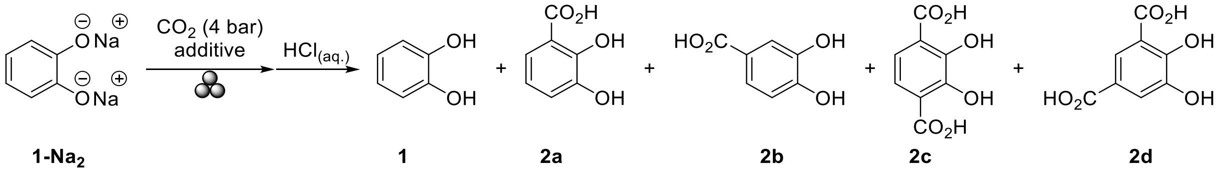

Entry	Additive	Additive [equiv.]	η [µL·mg^{-1}]	1 [%]	2a [%]	2b [%]	2c [%]	2d [%]	Σ(2a-d) [%]
				Yield[b]					
1	15-crown-5	1.0	-	99	-	-	-	-	-
2	15-crown-5	2.0	-	99	-	-	-	-	-
3	$NaCO_3Me$	1.0	-	69	19	10	2	traces	31
4	DMC	1.0	0.55	99	-	-	-	-	-
5	Na_2CO_3	1.05	-	38	17	11	9	1	38
6	NaOMe	1.05	-	98	-	-	-	-	-
7	Cyrene®	0.38	0.25	51	25	16	5	3	49
8	Toluene	0.36	0.25	24	33	24	11	8	76
9	DBU[c]	3.0	2.91	81	-	-	-	-	-

[a] Reaction conditions: **1-M2** was weighed into ZrO_2-M milling vessels with gas valves and 5 ZrO_2-M balls (Ø = 1 cm) inside a glovebox and the vessels were sealed, 4 bar CO_2 were added outside the glovebox and the mixture was milled at the indicated rotational frequency for 6 x 180 min, with 30 min breaks after each cycle and a renewal of the CO_2 after the first cycle, followed by aqueous work-up with HCl. [b] Identification by NMR and quantification by quantitative NMR with 1,3,5-trimethoxybenzene as internal standard. [c] 1,8-Diazabicyclo(5.4.0)undec-7-ene.

3.3.3 Optimized general procedure for mechanochemical carboxylation reactions

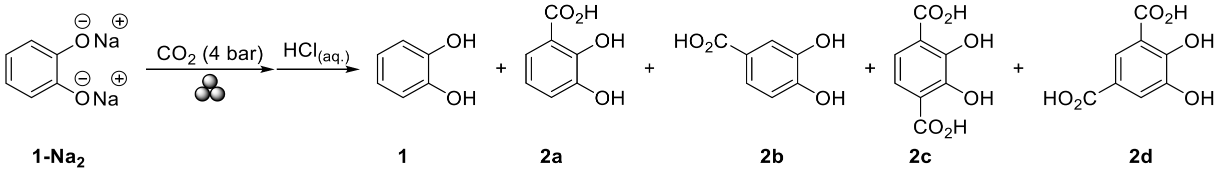

Scheme S6: Mechanochemical carboxylation reaction of disodium catecholate (1-Na2) with CO2.

For these reactions, the Fritsch Pulverisette 7 premium line was used with ZrO_2-M grinding bowls with 20 mL internal volumes and gas inlet/outlet valves as well as five

ZrO_2-M grinding balls (Ø = 10 mm). The grinding accessories were introduced into the glovebox by evacuating and flushing the airlock with argon thrice for at least 10 minutes each cycle. Disodium catecholate (**1-Na$_2$**, 231 mg, 1.5 mmol, 1.0 equiv.) was added to the grinding bowl. The grinding jar was closed, removed from the glovebox, flushed with CO_2, 4 bar CO_2 pressure was applied, and the mixture was ground for 180 min at 750 rpm. The CO_2 pressure was then renewed, and the mixture was ground at 750 rpm for 5×180 min with 4×30 min breaks in between. The pressure was then released, and the gas valves and seals were removed. The inside of the lid, the main sealing ring, the balls, and the inside of the grinding bowl were rinsed with deionized H_2O (approximately 50 mL) until all residues were removed. The gas valves were disassembled separately and thoroughly cleaned together with the valve seals using EtOH and a lint-free paper towel. The grinding jar, lid, balls, and main sealing ring were cleaned with water, little scouring powder and acetone, and dried in air. The reaction mixture was acidified with conc. HCl (approximately 5 mL) to approximately pH 1, placed in a 250 mL separatory funnel and extracted with Et_2O (3×50 mL). The collected organic phases were treated with aq. $NaHCO_3$ (5%, 3×25 mL), dried over $MgSO_4$ and the solvent was removed under reduced pressure. The remaining catechol was thus obtained in 23 ± 5% (37.3 ± 7.8 mg) yield. The aqueous basic phases remaining after extracting the ether phase were acidified with conc. HCl to pH 1, extracted with Et_2O (3×50 mL), the collected organic phases were dried over $MgSO_4$, and the solvent was removed under reduced pressure. 1,3,5-Trimethoxybenzene (TMB, 63.1 mg, 0.375 mmol, 0.25 equiv.) was added as internal standard and after recording a quantitative ^{1}H NMR spectrum of the crude product in CD_3OD, the NMR yield was determined through the integral ratios of distinctive signals of each product (**1**: m at 6.63–6.67 ppm, CH, 2H; **2a**: dd at 7.00 ppm, J = 7.9, 1.5 Hz, CH, 1H; **2b**: d at 6.80 ppm, J = 8.1 Hz, CH, 1H; **2c**: s at 7.36 ppm, CH, 2H; **2d**: d at 7.61 ppm, J = 2.1 Hz, CH, 1H) with the signal for TMB (s at 6.07 ppm, CH, 3H). For the optimal conditions, see Table S8, entry 8.

The crude product mixture was then adsorbed onto Celite and separated by column chromatography (Ø = 2.5 cm, 37 g, Et_2O/*n*-pentane 1:9 → 1:4 with 15 mL acetic acid per 500 mL), leading to the isolation of **2a** and **2b** in 18% and 23% respectively, with large losses due to incomplete separation. Using this procedure, products **2c** and **2d** could not be eluted. Hence, the decision was made to report ^{1}H NMR yields to more accurately represent the true performance of the mechanochemical carboxylation reaction.

Note: While the overall procedure gives reproducible results, a few essential notes should be made. First and foremost, and as stated in previous works with the same setup[4] all valves and insulation seals should be replaced regularly besides being thoroughly cleaned after and investigated with caution prior to each experiment. Second, for the dicatecholate salts, storage under the strict exclusion of oxygen and water is of paramount importance to avoid any discoloration resulting from degradation.

3.3.4 Calculation of the amount of CO_2 added to milling vessels

The ideal gas law was used to calculate the yield, as the behavior of CO_2 under the applied reaction conditions can be adequately described in this way. After converting the equation to volume V, the result is:

$$p \times V = n \times R \times T \therefore n = \frac{p \times V}{R \times T}$$

The volume of the powder used was neglected in the calculations, but the volume of the balls was considered and subtracted from the available volume inside the grinding bowl. A ball diameter of Ø = 10 mm resulted in a radius of r = 5 mm, corresponding to 0.5 cm. From this, the ball volume could be calculated as:

$$V_{ball} = \frac{4}{3} \times \pi \times r^3 = \frac{4}{3} \times \pi \times (0.5\ \text{cm})^3 = 0.524\ \text{cm}^3$$

For the five balls used, the volume was multiplied by five:

$$V_{balls} = 5 \times 0.524\ \text{cm}^3 = 2.618\ \text{cm}^3$$

After subtracting this volume from the inner volume of the grinding bowl and converting the remaining volume into the SI unit m^3, the available inner volume V_{inside} was:

$$V_{inside} = 20\ \text{cm}^3 – 2.618\ \text{cm}^3 = 17.382\ \text{cm}^3 \equiv 1.74 \times 10^{-5} \text{m}^3$$

As the milling vessel was always charged with at least 4 bar of CO_2, and the average room temperature (at which the vessel was pressurized) was around 25 °C, the amount of substance $n(CO_2)$ was obtained by inserting this pressure into the ideal gas law converted at the beginning:

$$n = \frac{\Delta p \times V}{R \times T} = \frac{4\ \text{bar} \times (1.74 \times 10^{-5}\ \text{m}^3)}{8.314\ \text{J} \cdot \text{K}^{-1} \cdot \text{mol}^{-1} \times (25 + 273.15)\ \text{K}} = 2.80\ \text{mmol}$$

This calculation shows that, when neglecting the volume occupied by the powder within the milling vessel, more than 1.0 equivalent of CO_2 (*e.g.* at least 2.80 mmol CO_2 versus 1.5 mmol of disodium catecholate (**1-Na$_2$**) for the optimal conditions) was present in each reaction so that the gas would not become the limiting reagent.

3.3.5 Upscaling procedure for ball-mill derived plasticizer mixture

As outlined in the general procedure (3.3.3), the work-up followed the acid-base extraction method described for phenols and resorcinols by the group of Govender,[7] i.e., initial extraction of catechol under basic conditions, followed by re-acidification to pH 1 and extraction of the carboxylic acids. Finally, a large-scale synthesis of the carboxylic acids **2a-d** was envisioned for the esterification reactions (see section 11). Given the hardware restrictions of ball mills, instead of running one reaction on larger scale, the reaction was run under optimum conditions several times to obtain enough carboxylated material for the plasticizer synthesis and tests. The resulting product mixtures were combined to give a mixture in a relative ratio of 46:31:12:11 (**2a**:**2b**:**2c**:**2d**) as described in section 3.4. Catechol could be recovered in 23 ± 5% (37.3 ± 7.8 mg) yield. Because there were slight deviations in the composition of each individual product mixture after ball milling and the additional washing step, the composition of the “large-scale” batch slightly deviates from the optimal result obtained during optimization (Table S8, entry 8).

3.4 Quantification of the ball mill mixtures for plasticizer candidate synthesis

In section 11, the obtained mixture of carboxylated catechols **2a**-**2d** from the mechanochemical carboxylation reaction is derivatized in families of plasticizer candidates. To this end, the exact starting composition of a crude ball mill-derived mixture (see section 3.3.5) was determined (Table S11).

Experimental procedure:

An aliquot of the ball mill mixture was taken and heated at 80 °C for 18 h under vacuum to remove any remaining water present (from extraction). The ^{1}H NMR spectrum was recorded, showing a relative ratio of **2a**:**2b**:**2c**:**2d** in the mixture of 46:31:12:11.

Table S11: Determination of the relative ratio of the ball mill mixture by ^{1}H NMR spectroscopy (400 MHz, DMSO-*d6*).

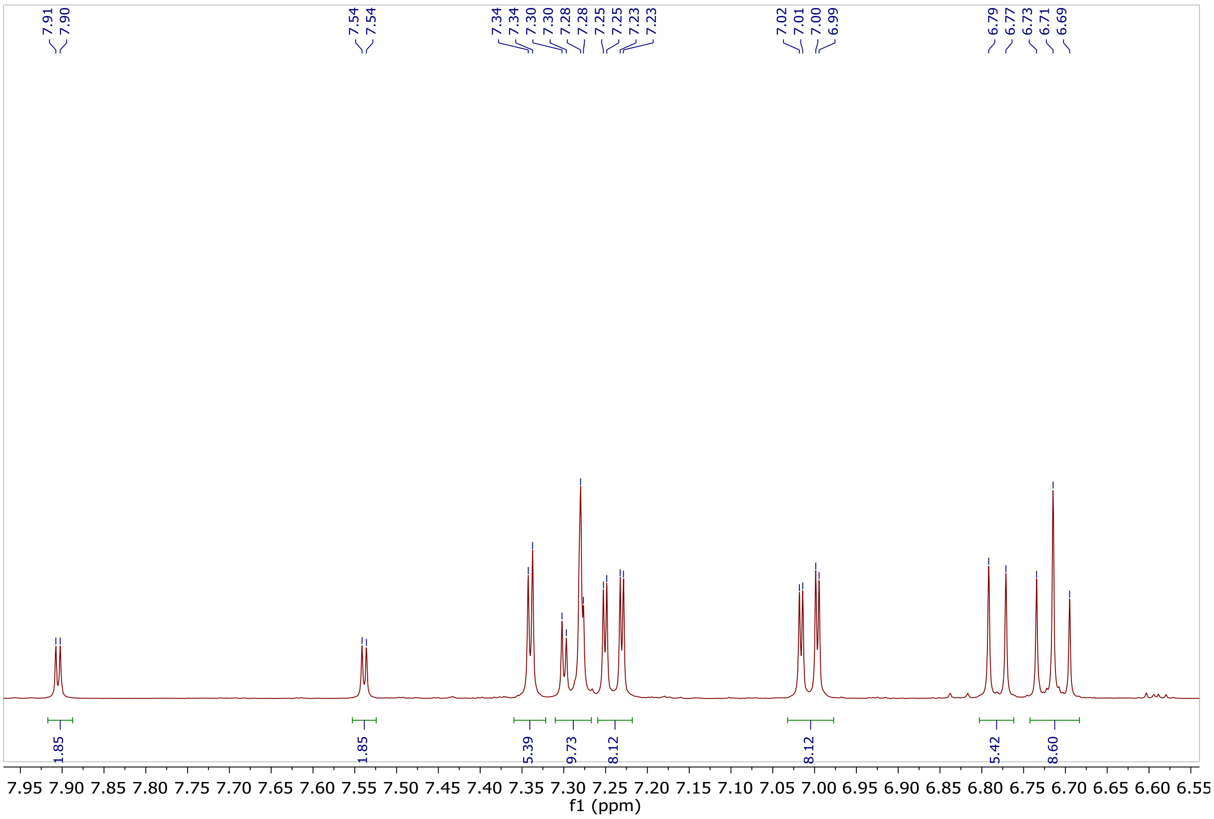

Isomer	Integration	#H-atoms	Relative ratio
**2a**	8.12	1	46%
**2b**	5.39	1	31%
**2c**	4.34[a]	2	12%
**2d**	1.85	1	11%
Total:			100%

[a] Calculated as 9.73 – 5.39 = 4.34.

4. Solution casting of polymers with plasticizers and DSC characterization

4.1 Procedure for PVC samples

Preparation of PVC films and subsequent DSC analysis was performed according to a modified literature procedure.[8] Plasticizers (50 mg, 10 wt%) were accurately weighed in a 5 mL vial. PVC (450 mg, 90 wt%) was accurately weighed in a 25 mL vial. Plasticizers were quantitatively transferred to the 25 mL vial containing the PVC using THF (5 mL). The vial was equipped with a magnetic stirring bar, covered with aluminum foil, and the suspension was stirred at 50 °C until full dissolution. Next, the solution was cast into a glass Petri dish and dried for approximately two hours at room temperature. The obtained PVC film was further dried under vacuum for at least five days to remove residual solvent and then analyzed by DSC. PVC films containing no plasticizers were prepared similarly and used in the control experiment. DSC measurements were performed in open aluminum pans under nitrogen flow, samples equilibrated at 40.0 °C, ramped at 10.0 °C/min to 190.0 °C, remained isothermal at 190.0 °C for 5 minutes (end of heating cycle 1), cooled at 10.0 °C/min to −10.0 °C, remained isothermal at −10.0 °C for 5 minutes (end of cooling cycle 1), and ultimately ramped at 10.0 °C/min to 190.0 °C (end of heating cycle 2). The T_g data from the DSC measurements were taken from the second heating cycle with the step midpoint set at half height. All DSC thermograms can be found in section 14.

4.2 Procedure for PLA samples

Preparation of PLA films and subsequent DSC analysis was performed according to a modified literature procedure.[8] Plasticizers (50 mg, 10 wt%) were accurately weighed in a 5 mL vial. PLA (450 mg, 90 wt%) was accurately weighed in a 25 mL vial.

<u>For compounds soluble in $CHCl_3$:</u> Plasticizers were quantitatively transferred to the 25 mL vial containing the PLA using $CHCl_3$ (5 mL). The vial was equipped with a magnetic stirring bar, covered with aluminum foil, and the suspension was stirred at 50 °C until full dissolution.

<u>For compound (**2a**) insoluble in $CHCl_3$</u>: $CHCl_3$ (4 mL) was added to the 25 mL vial containing the PLA. The vial was equipped with a magnetic stirring bar, covered with aluminum foil, and the suspension was stirred at 50 °C until full dissolution. Plasticizers were dissolved in THF (1 mL) and quantitatively transferred to the PLA solution. The resulting mixture was stirred for an additional 20 minutes.

Next, the solution was cast into a glass Petri dish and dried for approximately two hours at room temperature. The obtained PLA film was further dried under vacuum for at least five days to remove residual solvent and then analyzed by DSC. PLA films containing no plasticizers were prepared similarly and used in the control experiment. DSC measurements were performed in open aluminum pans under nitrogen flow, samples equilibrated at 40.0 °C, ramped at 20.0 °C/min to 230.0 °C, remained isothermal at 230.0 °C for 5 minutes (end of heating cycle 1), cooled at 20.0 °C/min to −10.0 °C, remained isothermal at −10.0 °C for 5 minutes (end of cooling cycle 1), and ultimately ramped at 20.0 °C/min to 230.0 °C (end of heating cycle 2). The T_g data from the DSC measurements were taken from the second heating cycle with the step midpoint set at half height. All DSC thermograms can be found in section 14.

4.3 Photographic report of solution casting and DSC characterization

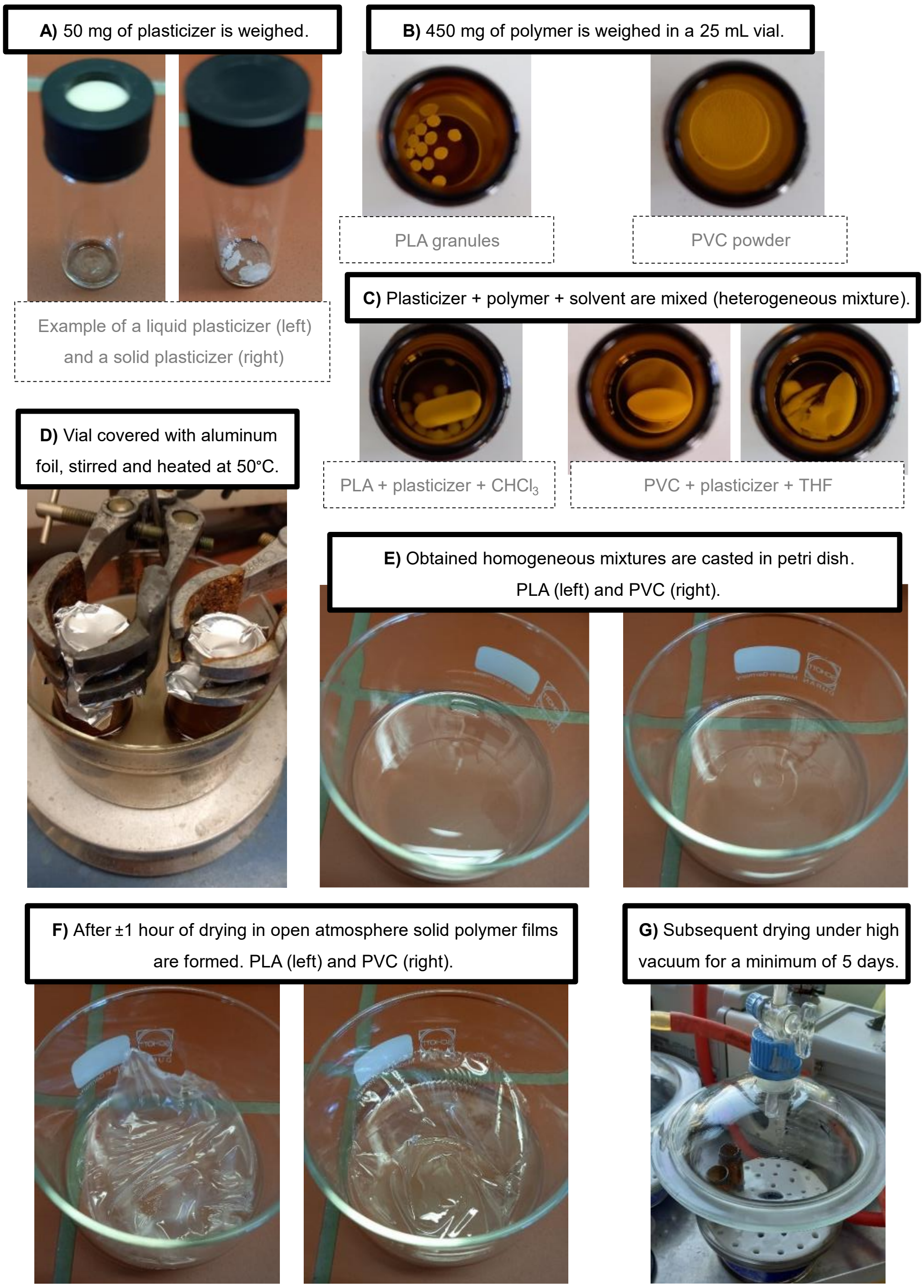

Figure S21: Photographic report of solution casting.

The addition of solvent to a polymer usually causes the polymer to swell, resulting in the polymer sticking at the bottom of the vial. To minimize blending times for PVC, it is beneficial to separate the polymer from the bottom, as illustrated in the second image of the vial in Figure S21, C.

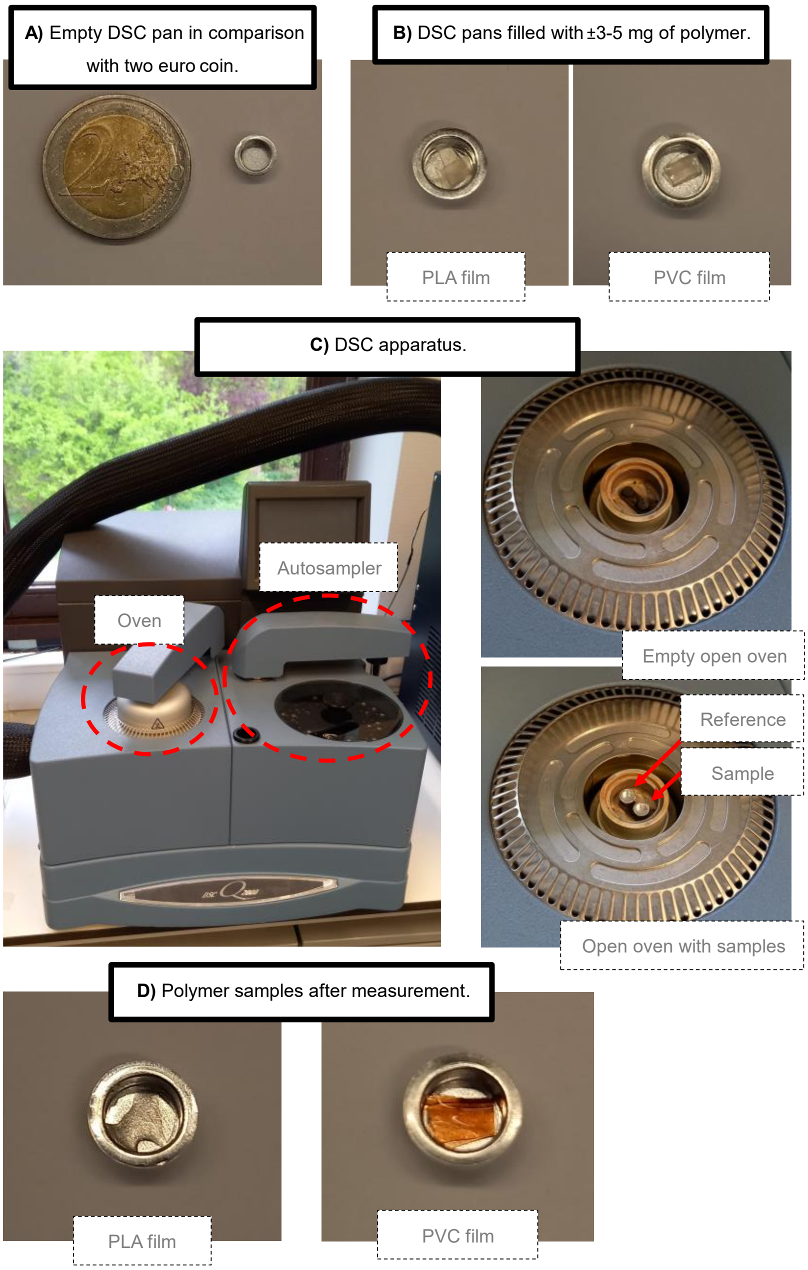

Figure S22: Photographic report of DSC measurements.

PLA samples melt during analysis, resulting in a droplet-like PLA sample. PVC samples begin to degrade upon heating at temperatures starting from 220 °C through dehydrochlorination.[9] Traces of this process cause the samples to develop a dark red to brown color after analysis. However, this discoloration does not significantly affect the T_g results obtained, as the heating cycles (max. temperature of 190 °C) do not nearly approach the degradation temperature.[10]

5. Plasticizer performance of commercial benchmark plasticizers

To accurately compare the performance of the ball mill-derived plasticizer candidates, their glass transition temperature (T_g) as obtained by Differential Scanning Calorimetry (DSC, see section 4) was compared to commercial benchmark plasticizers. The T_g's of the commercial benchmarks were determined first (Figure S23-S24). In PVC, phthalates dominate as plasticizers, so di(2-ethylhexyl) phthalate (DEHP) was selected, as well as di(isononyl) cyclohexane-1,2-dicarboxylate (DINCH), which is a common non-phthalate-based alternative. Both plasticizers lowered the T_g of PVC from 84.2 °C to 58.2–58.5 °C (Figure S23, A). In biorenewable PLA, citrates are often applied as plasticizers, hence triethyl citrate (TEC), tributyl citrate (TBC), and acetylated triethyl 2-acetylcitrate (TEAC), and tributyl 2-acetylcitrate (TBAC) were considered. The largest decrease in T_g was observed for TBAC, lowering the T_g of PLA from 61.4 °C to 54.6 °C (Figure S23, B). Furthermore, dioctyl sebacate (DOS) and epoxidized soybean oil (ESO) were also measured, being even more efficient plasticizers (T_g = 52.5–51.3 °C). However, when DEHP was mixed with PLA and tested, it proved the best performing benchmark plasticizer in PLA and was consequently used to compare the new plasticizer candidates against. Note that since DEHP is a fossil-based plasticizer and toxic, it is usually not used in combination with biorenewable PLA in real applications. In the following sections, DEHP and DINCH will be depicted as representative benchmarks for plasticizing efficiency in PVC, while TBAC and DEHP will be highlighted as representative benchmarks for PLA.

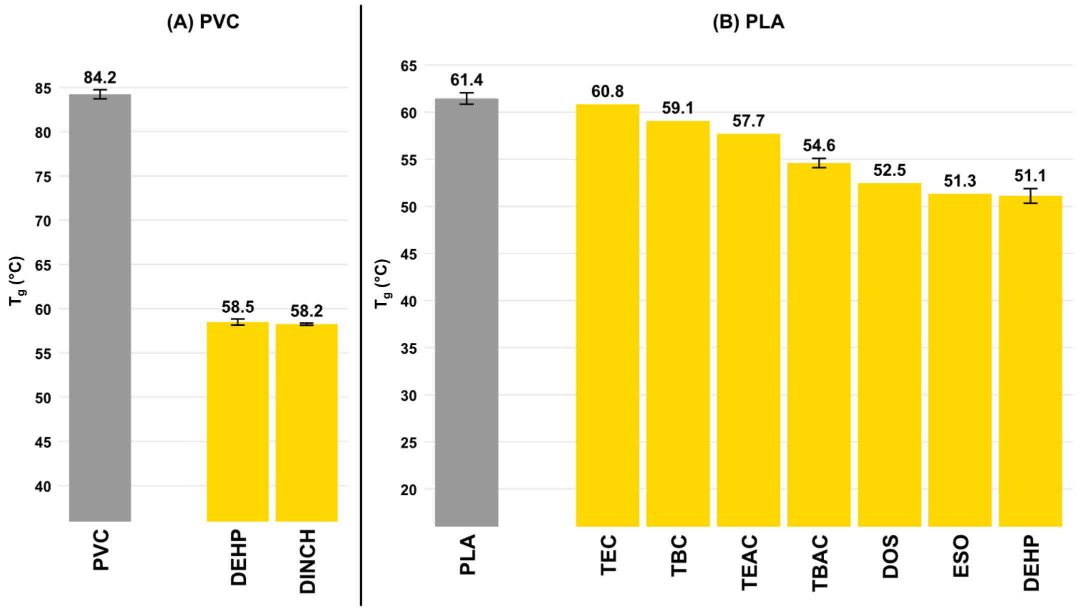

Figure S23: Plasticizer performance of benchmark plasticizers for PVC (A) and PLA (B). Glass transition temperature (T_g) determined using Differential Scanning Calorimetry (DSC) of polymers containing 10 wt% of the respective plasticizing candidate, mixed by solution casting (see section 4). The T_g values for pure polymer, and the selected benchmark plasticizers (DEHP and DINCH in PVC, and DEHP and TBAC in PLA) are shown as the average of four measurements with standard deviation. All other T_g values originate from single measurements.

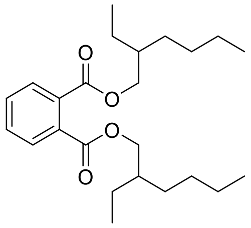

DEHP
(di-2-ethylhexyl phthalate)

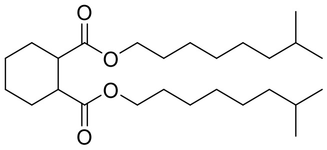

DINCH
(diisononyl cyclohexane-1,2-carboxylate)

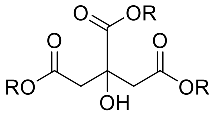

R = Et: **TEC** (triethyl citrate)
R = *n*-Bu: **TBC** (tributyl citrate)

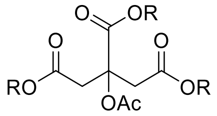

R = Et: **TEAC** (triethyl 2-acetylcitrate)
R = *n*-Bu: **TBAC** (tributyl 2-acetylcitrate)

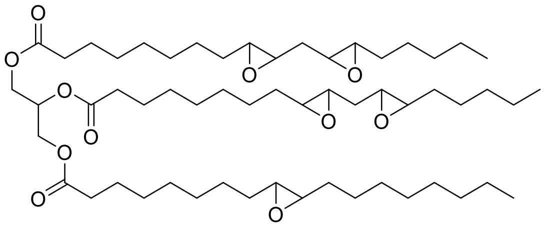

ESO (epoxidized soybean oil)

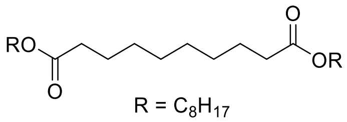

R = C_8H_{17}

DOS (dioctyl sebacate)

Figure S24: Structures of common commercial plasticizers in PVC and PLA applied as benchmarks.

6. Derivatizations on 2,3-dihydroxybenzoic acid (2a) to obtain plasticizing properties

Catechol, retrievable from biomass, was utilized to incorporate CO_2 by the mild, solvent-free mechanochemical method (section 3). Herein, a mixture of 2,3-dihydroxybenzoic acid (**2a**), 3,4-dihydroxybenzoic acid (**2b**), 2,3-dihydroxyterephthalic acid (**2c**), and 4,5-dihydroxyisophthalic acid (**2d**) was obtained. In the following sections, derivatives of these platform molecules and mixtures thereof are evaluated as plasticizers for PVC and PLA. In the first instance, esters derived from major compound **2a** were synthesized to assess whether they possess potential as plasticizers.

6.1 Derivatization of 2,3-dihydroxybenzoic acid (2a) towards plasticizer candidates for PVC and PLA

6.1.1 Fischer esterification

For the esterification of the carboxylic acid groups, biobased alcohols differing in length and branching were selected. Methanol, pentan-1-ol, and 2 ethylhexan-1-ol were used as they are also commonly applied in commercial plasticizers.[11] The oxidation-sensitive catechol moieties[12] required the following measures to be standardly taken during set-up of the reaction: i) reaction solvents of reactions at elevated temperatures were degassed by purging the solvent with argon under ultrasonication in order to remove oxygen, and ii) all reactions were carried out under an inert argon atmosphere.

Methyl ester **3a** was synthesized by a Fischer esterification in an excess of methanol with an equimolar amount of sulfuric acid (Scheme S7, A).[13] The reaction mixture was heated to reflux and stirred overnight, delivering methyl esters **3a** in good yield (89%). In case of the pentyl ester **6a** (Scheme S7, B), *n*-pentanol was used as the solvent at reflux temperature (138 °C) resulting in a yield of 90%.[14] The Fischer esterification with 2-ethylhexan-1-ol used a modified procedure due to the difficult removal of 2-ethylhexan-1-ol (Scheme S7, C).[15] Herein, 1.05 equivalent of 2-ethylhexan-1-ol was reacted with 2,3-dihydroxybenzoic acid (**2a**) in *p*-xylene at reflux to obtain the 2-ethylhexyl ester (**9a**) in good yield (75%). All Fischer esterification reactions were easily performed on multigram-scale. For detailed experimental procedures, see section 12.2.

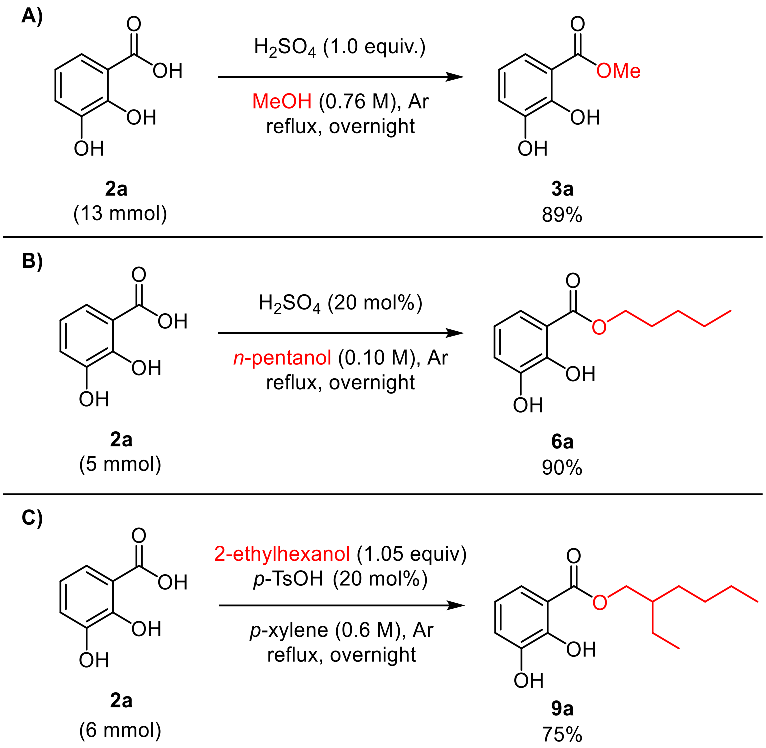

Scheme S7: Fischer esterification reactions of 2a towards 3a, 6a, and 9a with A) MeOH using H_2SO_4, B) *n*-pentanol using catalytic H_2SO_4, and C) 2-ethylhexan-1-ol in *p*-xylene using catalytic *p*-TsOH.

6.1.2 *O*-Acylation of the phenolic hydroxyl groups

With the methyl, pentyl, and 2-ethylhexyl esters **3a**, **6a**, and **9a** in hand, the phenolic hydroxyl groups were esterified with activated acetic acid and levulinic acid as they are common motifs in existing plasticizers.[11] Additionally, these acids can be retrieved from biorenewable sources.[16] The *O*-acetylation reactions were performed using acetyl chloride and triethylamine as a base (Scheme S8). The standard procedure prescribed CH_2Cl_2 as solvent.[11c] The addition of acetyl chloride happened at 0 °C to control the exothermic behavior, after which the reaction mixture was allowed to warm to room temperature and react overnight. After overnight stirring, full conversion was reached for all examples obtaining the acetylated compounds in good to excellent yields (72-91%). For detailed experimental procedures see section 12.2.

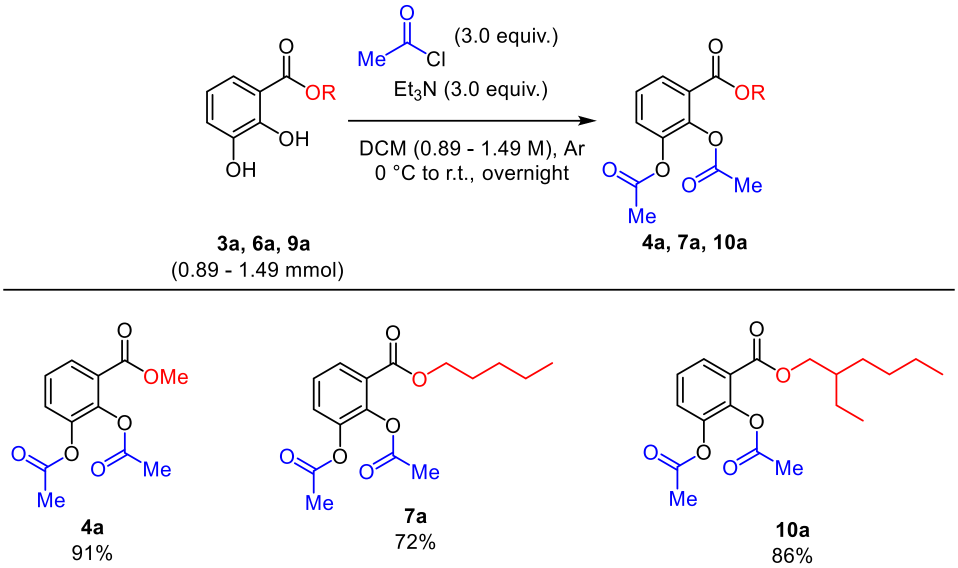

Scheme S8: *O*-Acetylations of phenolic hydroxyl groups of 3a, 6a, and 9a to plasticizer candidates 4a, 7a, and 10a using acetyl chloride. Reactions conducted on 1.49 mmol (**4a**), 0.89 mmol (**7a**) and 0.94 mmol (**10a**) scale in 1.0 mL of CH_2Cl_2.

A Steglich esterification was chosen to couple levulinic acid to the phenolic hydroxyl groups (Scheme S9).[17] *N*-Ethyl-*N'*-(3-dimethylaminopropyl)carbodiimide hydrochloride (EDC•HCl) was chosen as coupling reagent since its urea byproduct can be easily removed by an aqueous acidic extraction. 4-Dimethylaminopyridine (DMAP) was added as an acyl transfer catalyst and CH_2Cl_2 was used as the solvent. After overnight reaction, full conversion was observed by TLC analysis for all examples resulting in good to excellent yields (77-94%). For detailed experimental procedures see section 12.2.

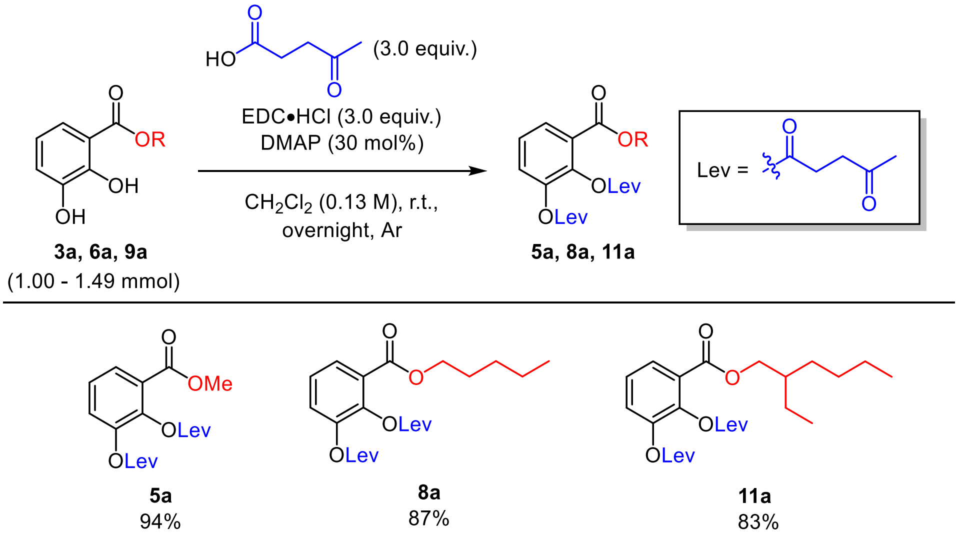

Scheme S9: *O*-Levulinoylations of phenolic hydroxyl groups of 3a, 6a, and 9a to plasticizer candidates 5a, 8a, and 11a using Steglich esterification with levulinic acid. Reactions conducted on 1.49 mmol (5a) and 1.00 mmol (8a, 11a) scale in 11 mL (5a) or 7.5 mL (8a, 11a) of CH_2Cl_2.

6.2 Testing the plasticizer efficiency of 2,3-dihydroxybenzoic acid derivatives 3a-11a in PVC and PLA

With the synthesized plasticizer candidates derived from 2,3-dihydroxybenzoic acid (**2a**) in hand, their plasticizer performance was investigated in PVC and PLA (Figure S25). To this end, the solution casting method was used in which plasticizer candidates (10 wt%) were mixed with the polymers PVC or PLA (90 wt%) by fully dissolving both components in a compatible solvent (see section 4 for details). The non-derivatized 2,3-dihydroxybenzoic acid (**2a**) does not possess a typical plasticizer structure (lacking ester spacer groups).[11b] As expected, no plasticizing effect was observed in PVC and PLA indicating that derivatization is crucial to design potential plasticizers (Figure S25, purple). The carboxylic ester derivatives featuring free hydroxyl groups **3a** and **6a** also do not show considerable plasticizing properties in PVC and PLA (Figure S25, blue). Only the 2-ethylhexyl ester **9a** shows a slight plasticizing effect in both matrices, with the T_g of PVC lowering from 84.2 °C to 79.3 °C and the T_g of PLA lowering from 61.4 °C to 58.7 °C. Possibly, insufficient presence of spacer moieties circumvents efficient plasticization. Nevertheless, the dihydroxybenzoic acid derivatives **3a**, **6a**, and **9a** allowed further functionalization towards potential plasticizers by esterification of the phenolic hydroxyl groups.

The *O*-acetylated derivatives **4a**, **7a**, and **10a** (Figure S25, green) exhibit superior performance relative to the compounds with free hydroxyl groups for the pentyl and 2-ethylhexyl esters. On the other hand, the acetylated methyl ester **4a** only shows a marginal effect both in PVC and PLA. Nevertheless, in both polymer matrices the *O*-levulinoylated compounds **5a**, **8a** and **11a** (Figure S25, red) show even more enhanced performances. The *O*-acylations introduce additional ester functionalities, which are known to be beneficial in terms of plasticizing properties.[11b] On top of that, levulinoylation offers a longer carbon chain spacer (with a ketone moiety) compared to the acetyl derivatives explaining their superiority. Upon comparing the different *O*-levulinoylated esters **5a**, **8a**, and **11a**, methyl esters **5a** display the lowest performance again, while the longer chain pentyl ester **8a** and 2-ethylhexyl ester **11a** both deliver the best results. They even surpass the benchmarks in PLA, while this is not the case for PVC.

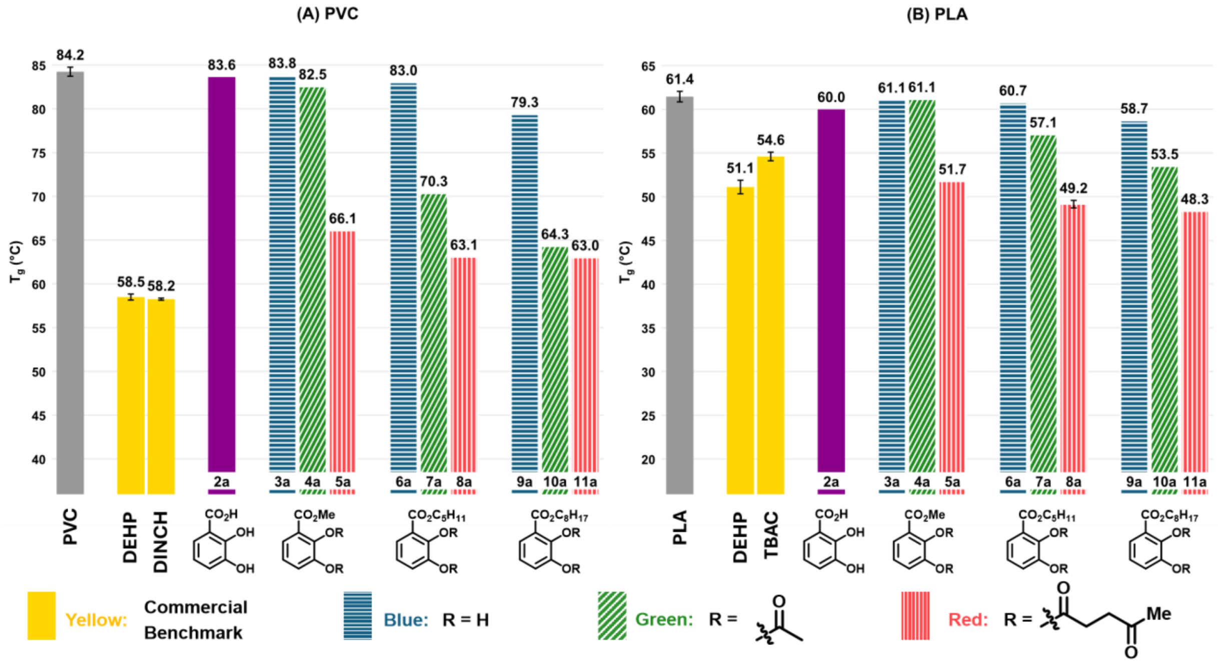

Figure S25: Plasticizing efficiency of 2,3-dihydroxybenzoic acid (2a)-derived plasticizer candidates (3a-11a) in PVC (A) and PLA (B). Glass transition temperature (T_g) determined using Differential Scanning Calorimetry (DSC) of polymers containing 10 wt% of the respective plasticizing candidate, mixed by solution casting (see section 4). T_g values for pure polymer, benchmark plasticizers and **8a** in PLA are shown as the average of four measurements with standard deviation. All other T_g values originate from single measurements.

7. Derivatization of 3,4-dihydroxybenzoic acid (2b), 2,3-dihydroxyterephthalic acid (2c) and 4,5-dihydroxyisophthalic acid (2d) to obtain plasticizing properties.

The *O*-levulinoylated pentyl ester **8a** and *O*-levulinoylated 2-ethylhexyl ester **11a** derived from **2a** were both identified as promising plasticizer candidates for PVC and PLA. Next, we wondered whether the other components (**2b**-**2d**) of the mechanochemical reaction can be derivatized analogously to potent plasticizers. To this end, the other members of the family of *O*-levulinoylated pentyl esters **8a**-**8d** and the family of *O*-levulinoylated 2-ethylhexyl esters **11a**-**11d** were synthesized.

7.1 Synthesis of *O*-levulinoylated pentyl esters 8a-8d and *O*-levulinoylated 2-ethylhexyl esters 11a-11d

First, **2b**-**2c** were converted into **6b**-**6c** and **9b**-**9c** by Fischer esterification similarly to the procedure outlined in Scheme S7 for major component carboxylated catechol **2a** (Scheme S10, A and B). Since 4,5-dihydroxyisophthalic acid (**2d**) is not commercially available, it was synthesized from 5-hydroxyisophthalic acid by a successive electrophilic bromination and Cu-catalyzed hydroxylation and immediately subjected to Fischer esterification with *n*-pentanol towards **6d** or 2-ethylhexanol towards **9d** without isolation of the carboxylic acid intermediates (Scheme S10, C).

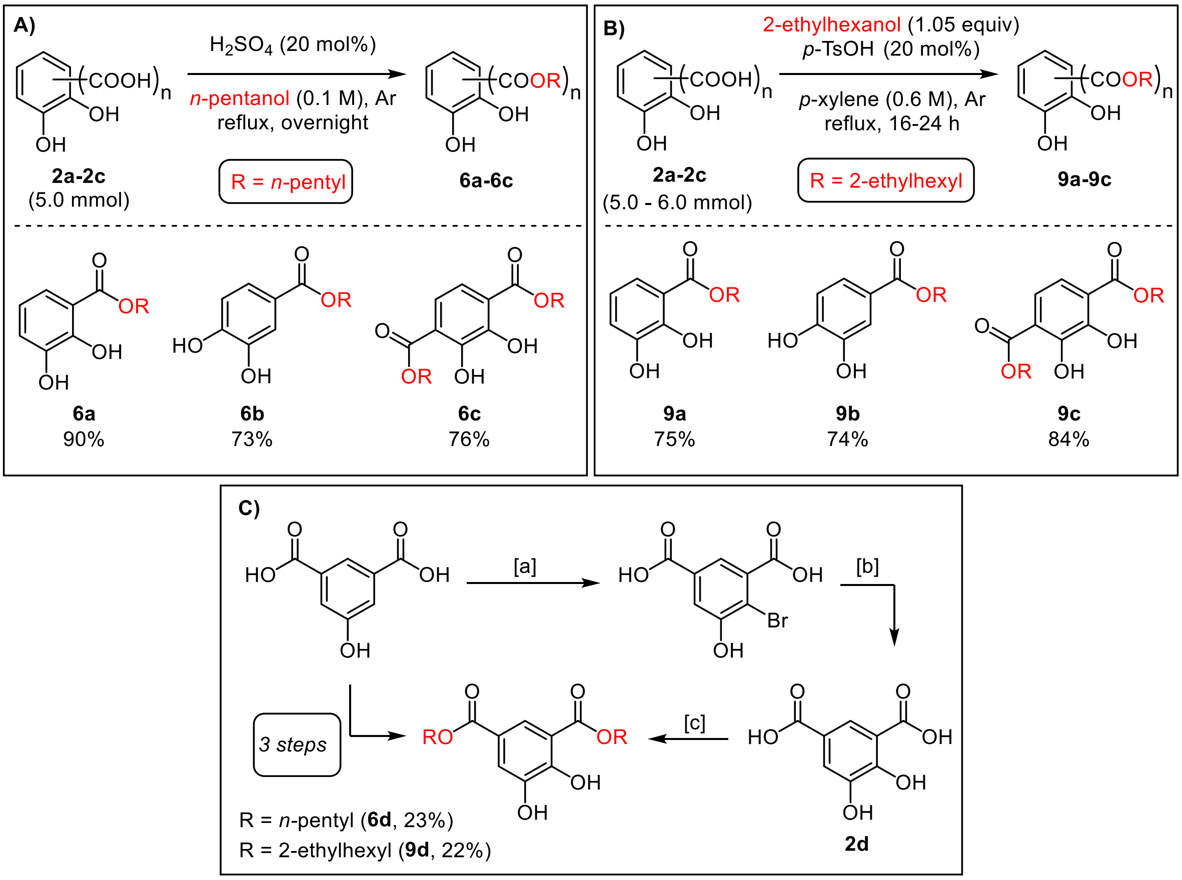

Scheme S10: Fischer esterification reactions of 2b-2d with A) *n*-pentanol using H_2SO_4 as catalyst, and B) 2-ethylhexan-1-ol in *p*-xylene using *p*-TsOH as catalyst. C) Synthesis of 4,5-dihydroxyisophthalic acid derivatives from 5-hydroxyisophthalic acid. [a] NBS (1.05 equiv.), conc. H_2SO_4, 50°C, 2 h; [b] Na_2CO_3,

TMEDA, CuBr, H_2O, 85 °C, 18 h, Ar; [c] for 6d: *n*-pentanol, H_2SO_4, 20 h, reflux, Ar; for 9d: 2-ethylhexanol, *p*-TsOH, *p*-xylene, reflux, 24 h, Ar. Overall yields are shown of one-pot syntheses from 5-hydroxyisophthalic acid with successive electrophilic bromination, Cu-catalyzed debrominative hydroxylation and Fischer esterification protocol. For synthetic details, see section 12.3.

Next, the synthesis of *O*-levulinoylated pentyl esters **8b-8d** and *O*-levulinoylated 2-ethylhexyl esters **11b-11d** originating from pentyl esters **6b-6d** and 2-ethylhexyl esters **9b-9d** was conducted by Steglich esterification similarly to Scheme S9 (Scheme S11). Due to solubility issues, the esterification of **6b** towards **8b** was conducted in THF instead of CH_2Cl_2, which proved to be a viable alternative. After overnight reaction, full conversion was observed by TLC analysis for all examples resulting in good to excellent yields (69-93%). For detailed experimental procedures see sections 12.4.

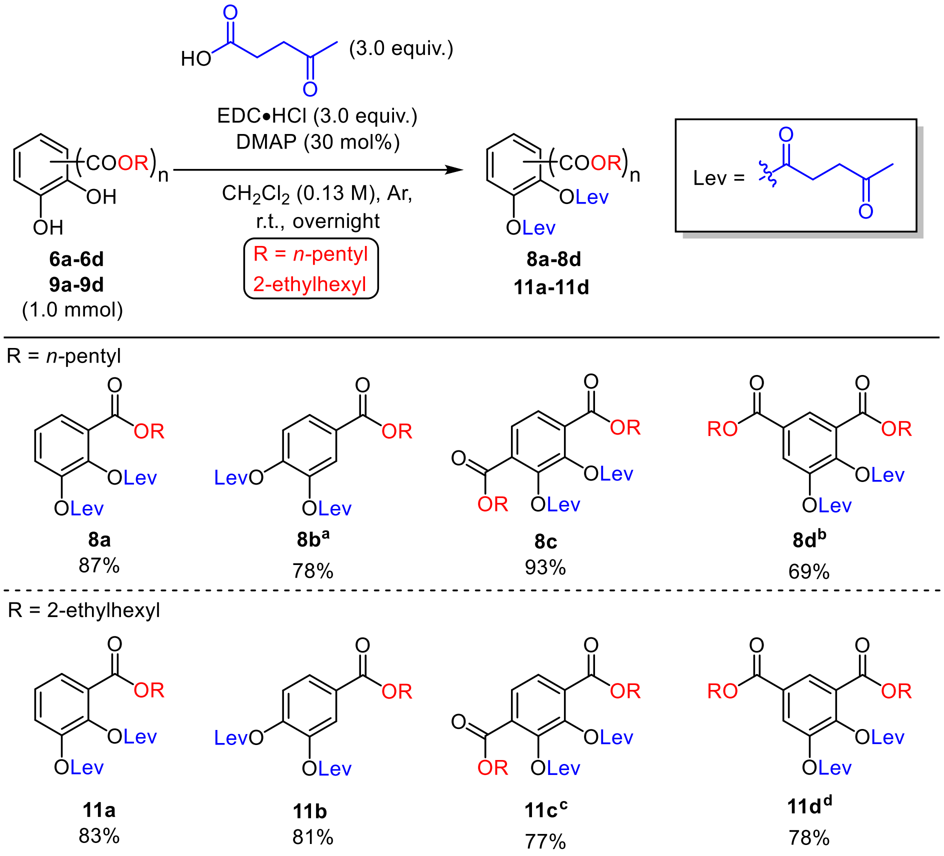

Scheme S11: *O*-Levulinoylations of the phenolic hydroxyl groups of pentyl esters 6a-6d to plasticizer candidates 8a-8d using Steglich esterification with levulinic acid. [a] Reaction conducted on 0.89 mmol scale in THF (0.13 M); [b] Reaction conducted on 0.62 mmol scale; [c] Reaction conducted on 0.80 mmol scale using CH_2Cl_2 (0.11 M); [d] Reaction conducted on 0.95 mmol scale using CH_2Cl_2 (0.11 M). For synthetic details, see section 12.4.

7.2 Testing of the plasticizing efficiency of *O*-levulinoylated pentyl esters 8a-8d and *O*-levulinoylated 2-ethylhexyl esters 11a-11d in PVC and PLA

The *O*-levulinoylated pentyl (**8b**, **8c**, **8d**) and *O*-levulinoylated 2-ethylhexyl esters (**11b**, **11c**, **11d**) were each tested separately as plasticizers in PVC and PLA (Figure S26). Surprisingly, the plasticizing properties for these other compounds are largely independent of the regioisomers and very similar to these of the major components **8a** and **11a** for both PVC and PLA. This behavior is very appealing towards the selection of a compound mixture as obtained from the mechanochemical ball milling synthesis, as this requires a set of good performing derivatives of 2,3- and 3,4-dihydroxybenzoic acid, 2,3-dihydroxyterephthalic acid and 4,5-dihydroxyisophthalic acid (**2a-2d**). In PVC, the *O*-levulinoylated pentyl esters (**8a-8d**) and the *O*-levulinoylated 2-ethylhexyl esters (**11a-11d**) each show good plasticizer potential, with T_g values between 63.0–65.1 °C (vs. 58.2 °C for DINCH benchmark). In PLA, the *O*-levulinoylated pentyl (**8a-8d**) and *O*-levulinoylated 2-ethylhexyl esters (**11a-11d**) even outperformed the benchmarks, with T_g values between 47.9–50.6 °C (vs. 51.1 °C for DEHP benchmark). Due to the high boiling point of 2-ethylhexan-1-ol, its removal after Fischer esterification was tedious and energy-intensive. For this reason, and because the T_g values of pentyl vs. ethylhexyl esters do not differ significantly, we opted to continue with the levulinoylated pentyl esters (**8a-8d**) for PLA.

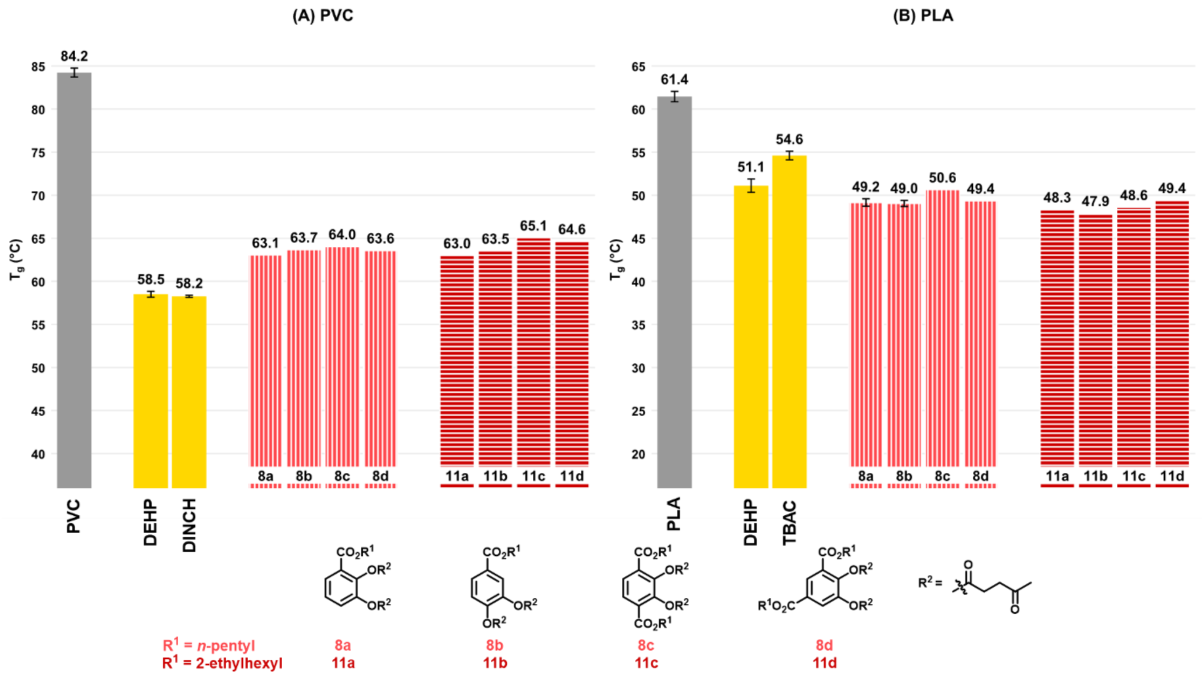

Figure S26: Plasticizing efficiency of *O*-levulinoylated pentyl esters 8a-8d and *O*-levulinoylated 2-ethylhexyl esters 11a-11d plasticizer candidates in PVC (A) and PLA (B). Glass transition temperature (T_g) determined using Differential Scanning Calorimetry (DSC) of polymers containing 10 wt% of the respective plasticizing candidate, mixed by solution casting (see section 4). T_g values for pure polymer, benchmark plasticizers and the best performing individual plasticizers **8a**, **8b** in PLA are shown as the average of four measurements with standard deviation. All other T_g values originate from single measurements.

8. Exchanging *O*-levulinoylation by *O*-pentanoylation: comparing *O*-pentanoylated pentyl esters 12a-12d with *O*-levulinoylated pentyl esters 8a-8d

With the commercial benchmarks hitherto not matched in PVC, the search towards improved plasticizers was continued with the exchange of the levulinoyl group by the less polar pentanoyl group for the best performing candidate family (**8a-8d**) found. We reasoned that PVC, being a more apolar polymer than PLA, would benefit from a more apolar plasticizer.

8.1 Synthesis of *O*-pentanoylated pentyl esters 12a-12d

Pentyl esters (**6a-6d**) were *O*-pentanoylated using pentanoyl chloride with triethylamine as the base (Scheme S12), similarly to the conditions for the *O*-acetylation in Scheme S8. Once more, the esterification of **6b** towards **12b** was conducted in THF due to limited solubility in CH_2Cl_2. Full conversion was obtained for all three compounds with excellent yields ranging from 90-96%. For detailed experimental procedures see sections 12.5.

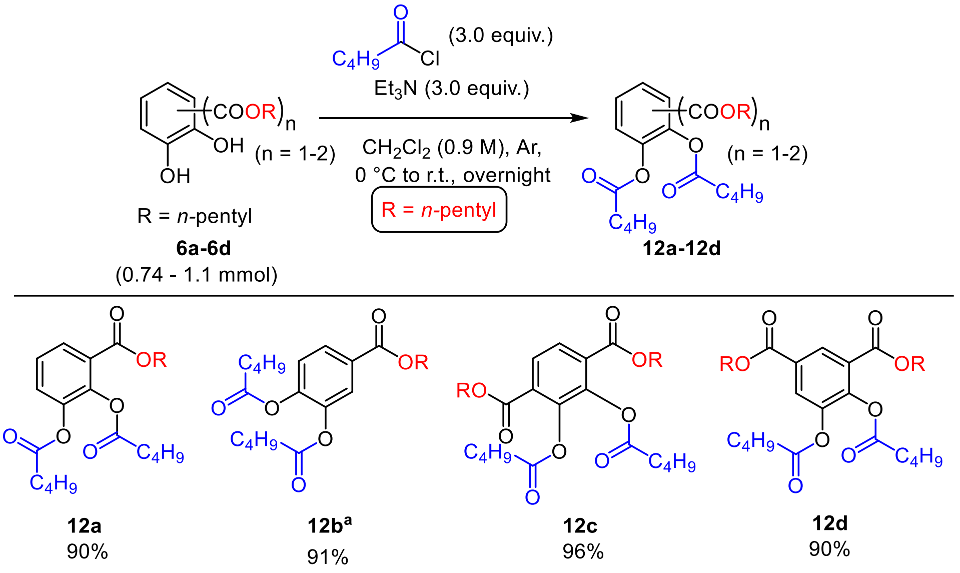

Scheme S12: *O*-Pentanoylations of the phenolic hydroxyl groups of pentyl esters 6a-6d to plasticizer candidates 12a-12d using pentanoyl chloride. [a] Reaction conducted on 0.53 mmol scale in THF (0.4 M). For synthetic details, see section 12.5.

8.2 Testing the plasticizing efficiency of *O*-pentanoylated pentyl esters 12a-12d in PVC and PLA and comparing them to *O*-levulinoylated pentyl esters 8a-8d

The *O*-pentanoylated derivatives (**12a-12d**) generally performed better compared to their *O*-levulinoylated analogues with T_g values between 58.7–62.4 °C (vs. 58.2 °C for DINCH benchmark). Gratifyingly, the 3,4-regioisomer **12b** was now found to be competitive with the commercial benchmarks (Figure S27, A). To allow a proper comparison, *O*-pentanoylated derivatives **12a-12d** were also tested in PLA (Figure S27, B). Here, the relative performance compared to the *O*-levulinoylated derivatives **8a-8d** is dependent on the exact derivative. With T_g values between 48.0–52.7 °C (vs. 51.1 °C for DEHP benchmark), the *O*-pentanoylated derivatives **12a-12d** do not show

an overall beneficial effect over the *O*-levulinoylated derivatives **8a-8d**, which are still preferred in PLA especially since the major compounds perform better in their *O*-levulinoylated form (**8a** and **8b** *vs.* **12a** and **12b**). The difference in polarity between the *O*-levulinoylated and *O*-pentanoylated compounds could explain their varying behavior in PVC and PLA.

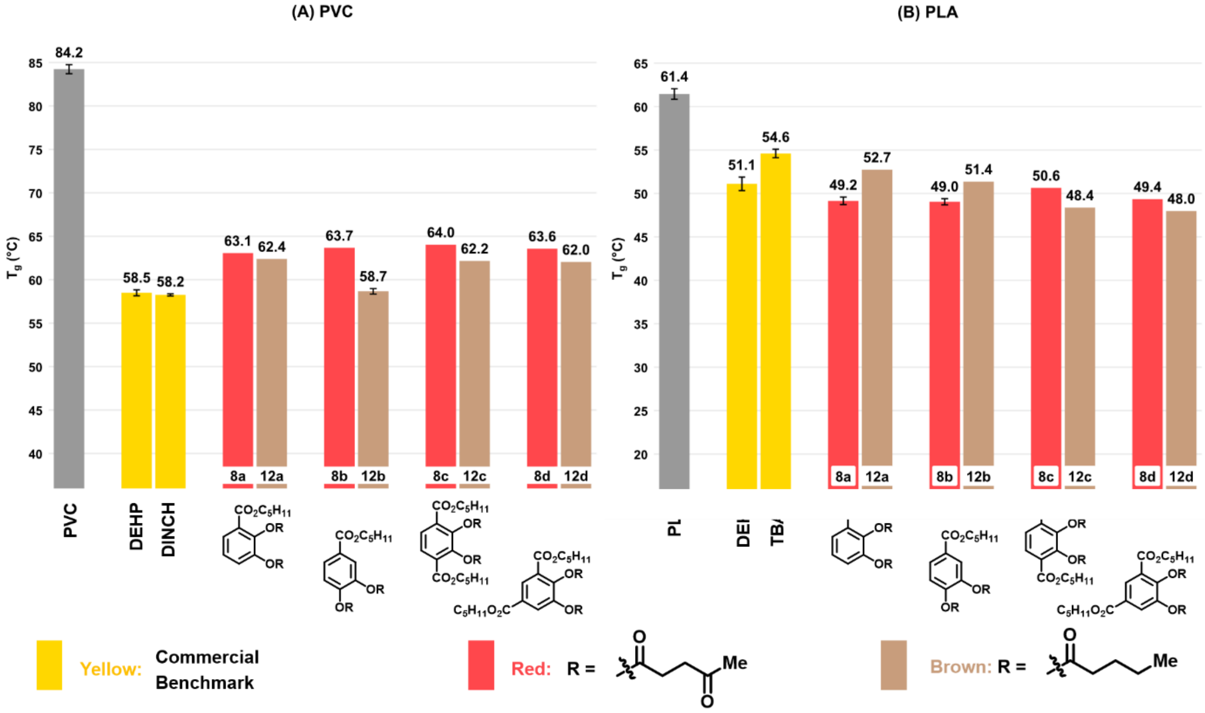

Figure S27: Comparison of plasticizing efficiency of *O*-levulinoylated pentyl esters (8a-8d) with *O*-pentanoylated pentyl esters (12a-12d) in PVC (A) and PLA (B). Glass transition temperature (T_g) determined using Differential Scanning Calorimetry (DSC) of polymers containing 10 wt% of the respective plasticizing candidate, mixed by solution casting (see section 4). T_g values for pure polymer, benchmark plasticizers and the best performing individual plasticizers (**12b** in PVC and **8a**, **8b** in PLA) are shown as the average of four measurements with standard deviation. All other T_g values originate from single measurements.

9. Optimization of synthesis of *O*-levulinoylated pentyl esters 8a-8d and *O*-pentanoylated pentyl esters 12a-12d

Before derivatizing a real ball mill-derived mixture to the set of candidate *O*-levulinoylated pentyl esters (**8a-8d**) and *O*-pentanoylated pentyl esters (**12a-12d**), the synthetic route was revised with attention to increasing the *green* potential of the reactions used by, for example, eliminating the use of chlorinated solvents.[18] Since the Fischer esterification of **2a-2d** to **6a-6d** was conducted in *green* pentanol as solvent and reactant, we mostly focused on the *O*-levulinoylation (section 9.1) and *O*-pentanoylation (section 9.2) of the catecholic hydroxyl groups.

9.1 *O*-Levulinoylation reaction

The *O*-levulinoylations of the catecholic hydroxyl groups of compounds **3a**, **6a**, and **9a** towards **5a**, **8a**, and **11a**, respectively, by Steglich esterification was conducted in CH_2Cl_2 (Scheme S9 and S11). Alternative conditions were explored aiming for a *greener* synthesis.

9.1.1 Fischer esterification

Our first attempts were based on a reported Fischer esterification of phenolic hydroxyl groups.[19] As such, pentyl 2,3-dihydroxybenzoate (**6a**) was chosen as model substrate (Table S12). First, the reaction was conducted in the melt (*m.p.* levulinic acid = 33 °C)[20] under open atmosphere (entry 1), allowing the evaporation of water, or in a closed vial in the presence of molecular sieves (entry 2). Partial hydrolysis of the pentyl ester was observed without any formation of phenolic levulinoylation. Furthermore, the mixture severely discolored indicating degradation of levulinic acid.[21] Hence, the reaction mixture was diluted using toluene at a lower temperature. This led to 17% of monolevulinoylated **8e** without hydrolysis of the pentyl ester. Unfortunately, low conversion was observed. The limited stability of levulinic acid at high temperatures in acidic medium prohibits raising the reaction temperature to increase the reaction kinetics.

Table S12: Fischer esterification of pentyl 2,3-dihydroxybenzoate (6a) using levulinic acid.

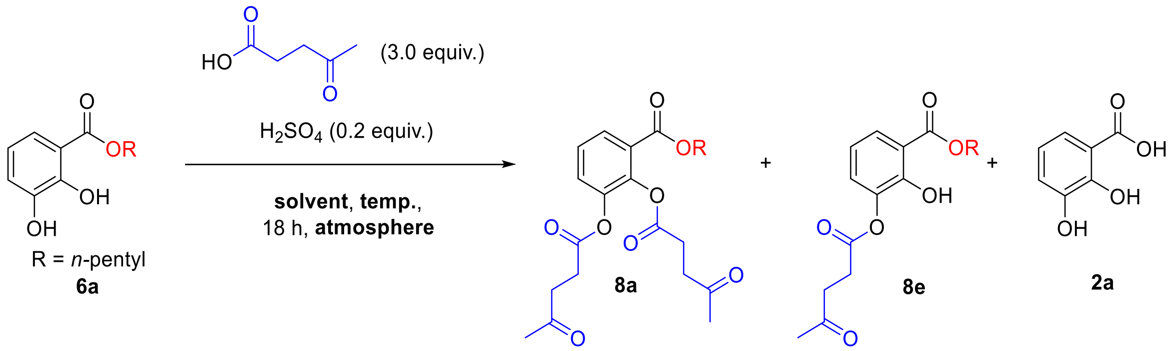

Entry	Solvent	Temp.	Atmosphere	Yield 8a	Yield 8e	Yield 2a	Yield 6a	ELN code
1[a]	/	130 °C	Open	0%	0%	19%	81%	AGO-002

2[a,b]	/	130 °C	Ar	0%	0%	33%	67%	AGO-003
3[c]	Toluene	110 °C	Ar	0%	17%	0%	83%	AGO-005

[a] 1 mmol scale; [b] 3Å molecular sieves were placed at top of the closed vial on cotton wool; [c] 2 mmol scale, reactive distillation set-up with 3Å molecular sieves using 20 mL of toluene.

9.1.2 Steglich esterification with greener solvents

Next, the hazardous CH_2Cl_2 used in the Steglich esterification (see Scheme S9 and S11) was substituted by the *greener* solvents methyl ethyl ketone and ethyl acetate (Table S13).[18] Interestingly, both gave a mixture of mono- and dilevulinoylation at 3.0 equivalents of EDC•HCl, with MEK delivering relatively more **8a** over **8e**. Nevertheless, compared to the result in CH_2Cl_2 or THF (see Scheme S9 and S11), these solvents proved suboptimal.

Table S13: Steglich esterification of pentyl 2,3-dihydroxybenzoic acid (6a) using levulinic acid.

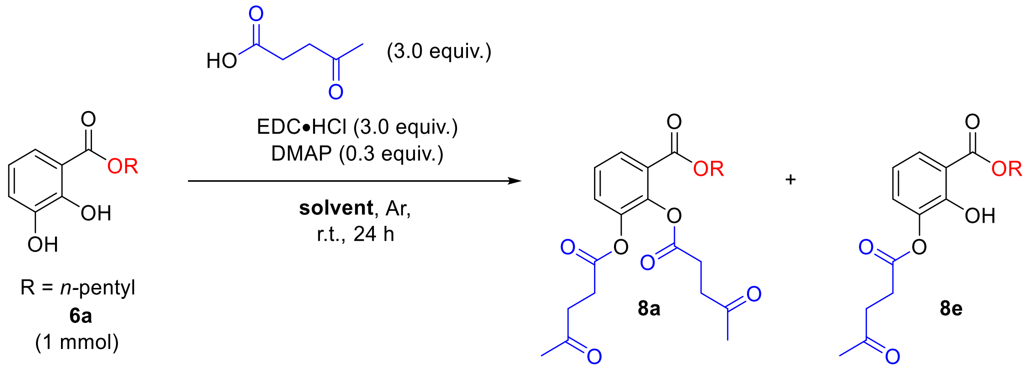

Entry	Solvent	Yield 8a	Yield 8e	ELN code
1	DMC	65%	35%	AGO-009
2	EtOAc	47%	53%	AGO-010
3	MEK	76%	24%	AGO-011

9.1.3 *O*-Acylation with (pseudo)levulinoyl chloride (13)

As a third possible pathway, we attempted a synthesis of levulinoyl chloride by mixing levulinic acid and thionyl chloride (1.1 equiv.) for 3 h at room temperature and concentrating the mixture under reduced pressure with the temperature never exceeding 40°C (Scheme S13). Interestingly, a cyclized ‘pseudo’ acyl chloride was formed, which has earlier been observed for γ-keto acids and γ-keto acyl halides.[22] The purity of the resulting yellow liquid was determined using quantitative ^{1}H NMR spectroscopy as 91% (Figure S28). The product was stable for at least two weeks upon storage in a sealed vial under argon atmosphere at –20 °C.

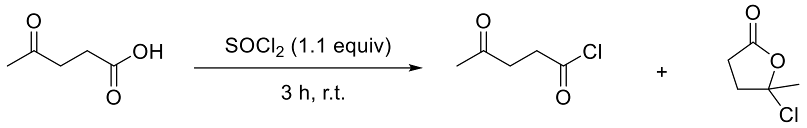

Scheme S13: Synthesis of pseudo-levulinoyl chloride (13) by deoxychlorination of levulinic acid.

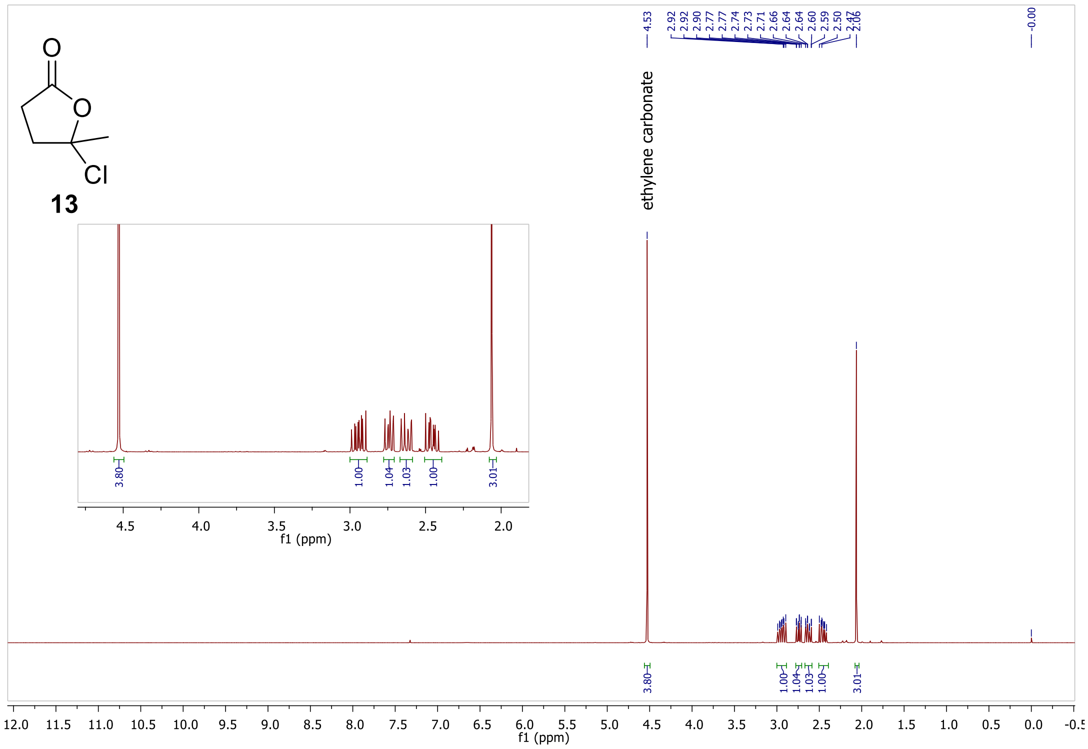

Figure S28: ^{1}H NMR (400 MHz, $CDCl_3$) spectrum of pseudo-levulinoyl chloride (13), admixed with ethylene carbonate as internal standard [AGO-084].

Subsequently, the *O*-levulinoylation of pentyl 2,3-dihydroxybenzoate (**6a**) was explored using pseudo-levulinoyl chloride (**13**) in the presence of Et_3N in EtOAc (Table S14, entry 1). The desired product **8a** was obtained in 59% yield with 22% of **8e** along with 9% of undesired **8f**. Neither the addition of catalytic DMAP[23] (entry 2), nor increasing the temperature to 50°C (entry 3) led to an improvement. Gratifyingly, replacing Et_3N by greener K_2CO_3 delivered a comparable result (entry 4). Other solvents were found to underperform (entries 5). To improve stirring, the reaction was diluted (entry 6), yet this resulted in a slightly lower yield of **8a**. Under these conditions, Na_2CO_3 proved inferior to K_2CO_3 (entry 7). Grinding K_2CO_3 drastically improved the yield of **8a** (entry 8), allowing to reduce the reaction time to 4 h (entry 9). However, this result could not be reproduced (entry 10). We hypothesized that the very hygroscopic ground K_2CO_3 rapidly adsorbs water from the environment affecting the reaction with the water-sensitive pseudo-levulinoyl chloride (**13**). To confirm this, ground K_2CO_3 was flame-dried under vacuum before the reaction. Surprisingly, only 2% of the desired disubstituted **8a** was observed (entry 11). Therefore, the amount of water was screened, resulting in greatly varying yields of **8a** and **8e** (entries 12-16). Eventually, the addition of 0.5 equiv. of water proved optimal (entry 13), delivering **8a** in 81% with 8% of **8e** and 8% of **8f**.

Subsequently, the amount of K_2CO_3 was increased to 3.75 equiv., which required an increase in the amount of EtOAc to allow proper stirring. The amount of water was screened again (entries 17-20), with 1.00 equiv. of water now being optimal. Finally, the ground and flame-dried base was pretreated with 1 hour of ultrasonication to achieve even finer particles (entries 21-22). A slight improvement was noticed, and this result was proven to be reproducible. Unfortunately, full conversion to the desired di-substituted product **8a** seemed difficult by this procedure, with mainly the formation of **8f** being hard to avoid. Removal of the undesired compounds **8e** and **8f** by basic extraction proved unsuccessful.

Table S14: *O*-Levulinoylations of pentyl 2,3-dihydroxybenzoate (6a) using pseudo-levulinoyl chloride (13).

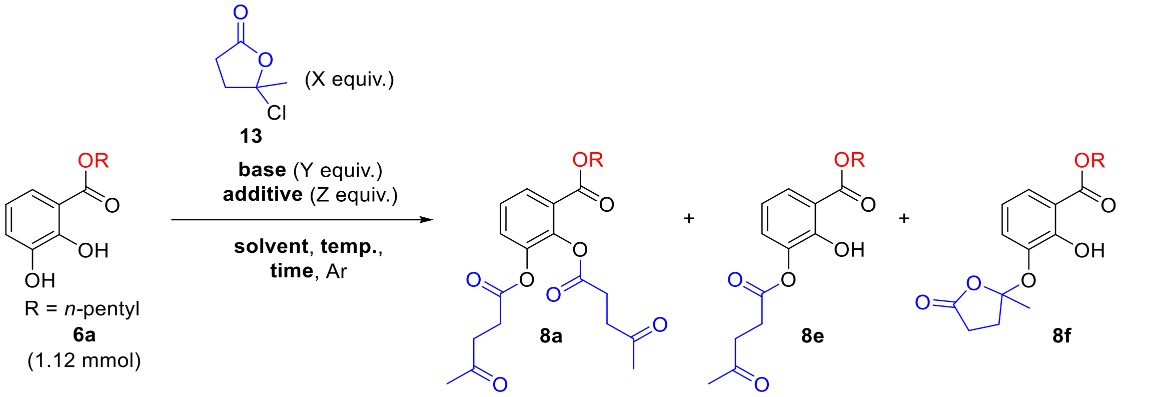

Entry	13 (equiv.)	Base (equiv.)[a]	Additive (equiv.)	Solvent	Molarity (M)	Temp.	Time (h)	Yield 8a (%)[b]	Yield 8e (%)[b]	Yield 8f (%)[b]	S.M. (%)[b]	Mass balance	ELN code
1	3.0	Et_3N (3.0)	/	EtOAc	0.88	r.t.	18	59	22	9	3	93	AGO-017
2	3.0	Et_3N (3.0)	DMAP (0.05)	EtOAc	0.88	r.t.	24	36	50	1	4	91	AGO-019
3	3.0	Et_3N (3.0)	/	EtOAc	0.88	50 °C	24	28	57	4	5	94	AGO-022
4[c]	3.0	K_2CO_3 (3.0)	/	EtOAc	0.88	r.t.	24	51	37	9	0	97	AGO-036
5	3.3	K_2CO_3 (3.0)	/	MeCN	0.88	r.t.	24	20	51	18	0	89	AGO-041
6	2.9	K_2CO_3 (3.0)	/	EtOAc	0.44	r.t.	24	40	57	7	0	104	AGO-044
7	3.0	Na_2CO_3 (3.0)	/	EtOAc	0.44	r.t.	24	4	69	16	6	95	AGO-054
8	3.0	K_2CO_3 (gr) (3.0)	/	EtOAc	0.44	r.t.	24	84	6	9	0	99	AGO-062
9	3.0	K_2CO_3 (gr) (3.0)	/	EtOAc	0.44	r.t.	4	78	12	9	0	99	AGO-063
10	3.3	K_2CO_3 (gr) (3.0)	/	EtOAc	0.44	r.t.	4	63	25	8	0	96	AGO-075
11	3.4	K_2CO_3 (gr+dr) (3.0)	/	EtOAc	0.44	r.t.	4	2	59	29	10	100	AGO-076

12	3.1	K_2CO_3 (gr+dr) (3.0)	H_2O (0.25)	EtOAc	0.44	r.t.	4	31	60	4	0	95	AGO-081
13	3.2	K_2CO_3 (gr+dr) (3.0)	H_2O (0.5)	EtOAc	0.44	r.t.	4	81	8	8	0	97	AGO-090
14	3.1	K_2CO_3 (gr+dr) (3.0)	H_2O (1.0)	EtOAc	0.44	r.t.	4	66	21	10	0	97	AGO-087
15	3.4	K_2CO_3 (gr+dr) (3.0)	H_2O (1.5)	EtOAc	0.44	r.t.	4	50	36	11	0	97	AGO-088
16	3.3	K_2CO_3 (gr+dr) (3.0)	H_2O (2.5)	EtOAc	0.44	r.t.	4	29	59	8	0	96	AGO-086
17	3.7	K_2CO_3 (gr+dr) (3.75)	H_2O (0.5)	EtOAc	0.35	r.t.	4	75	15	6	0	96	AGO-107
18	3.4	K_2CO_3 (gr+dr) (3.75)	H_2O (1.0)	EtOAc	0.35	r.t.	4	86	1	9	0	96	AGO-092
19	3.4	K_2CO_3 (gr+dr) (3.75)	H_2O (1.5)	EtOAc	0.35	r.t.	4	80	6	11	0	97	AGO-093
20	3.2	K_2CO_3 (gr+dr) (3.75)	H_2O (2.0)	EtOAc	0.35	r.t.	4	59	28	11	0	98	AGO-094
**21**	**3.7**	**K_2CO_3 (gr+dr+ultra) (3.75)**	**H_2O (1.0)**	**EtOAc**	**0.35**	**r.t.**	**4**	**88**	**1**	**9**	**0**	**98**	**AGO-108**
22	3.6	K_2CO_3 (gr+dr+ultra) (3.75)	H_2O (1.0)	EtOAc	0.35	r.t.	4	86	4	8	0	98	AGO-109

[a] Abbreviations: gr = ground, dr = dried, ultra = ultrasonicated. [b] Yields determined using quantitative ^{1}H NMR spectroscopy using 1,3,5-trimethoxybenzene as internal standard. [c] Reaction conducted on 0.508 mmol scale.

9.2 *O*-Pentanoylation reaction

The *O*-pentanoylation of pentyl 2,3-dihydroxybenzoate (**6a**) towards **12a** was initially conducted using pentanoyl chloride (3.0 equiv.) and Et_3N (3.0 equiv.) in CH_2Cl_2 (see section 8.1). In an attempt to improve the *green* aspects of this reaction, hazardous CH_2Cl_2 was exchanged for *green* EtOAc as a solvent, which resulted in *O*-pentanoylated product **12a** in 98% yield (Table S15, entry 1). Gratifyingly, toxic Et_3N could be replaced by benign K_2CO_3, using the final conditions for *O*-levulinoylation (section 9.1.2, Table S14, entry 22). Without ultrasonication of ground and dried K_2CO_3, still 6% of monopentanoylated **12e** was detected (entry 2) in the crude NMR, while when using ultrasonication, reproducible quantitative yields of **12a** were obtained in 4 h using 1.0 equiv. of water as an additive (entry 3-4). The low quantities of monopentanoylated **12e** obtained prevented its isolation.

Table S15: *O*-Pentanoylations of pentyl 2,3-dihydroxybenzoate (6a) using pentanoyl chloride.

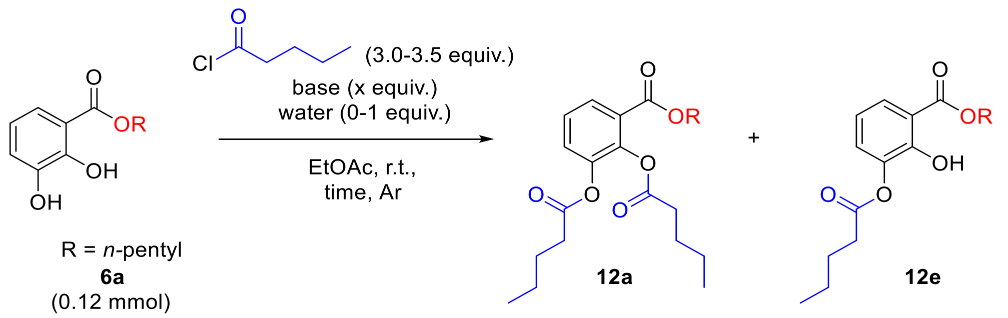

Entry	Pentanoyl chloride (equiv.)	Base (equiv.)[a]	Additive (equiv.)	Molarity (M)	Time (h)	Yield 12a (%)[b]	Yield 12e (%)[b]	ELN code
1	3.0	Et_3N (3.0 equiv.)	/	0.88	24	98	0	AGO-013
2	3.5	K_2CO_3 (gr+dr) (3.75 equiv.)	H_2O (1.0 equiv.)	0.35	4	95	6	AGO-098
3	3.5	K_2CO_3 (gr+dr+ultra) (3.75 equiv.)	H_2O (1.0 equiv.)	0.35	4	100	0	AGO-102
**4**	**3.5**	**K_2CO_3 (gr+dr+ultra) (3.75 equiv.)**	**H_2O (1.0 equiv.)**	**0.35**	**4**	**100**	**0**	**AGO-103**

[a] Abbreviations: gr = ground, dr = dried, ultra = ultrasonicated. [b] Yields determined using quantitative ^1H NMR spectroscopy using 1,3,5-trimethoxybenzene as internal standard.

10. Derivatization of model mixture (2a-2d) towards plasticizer candidates and testing

The mechanochemical synthesis directly delivers a mixture of carboxylated catechols **2a-2d**. It would therefore be highly practical if this mixture as a whole could be derivatized into the family of *O*-levulinoylated pentyl esters (**8a-8d**) identified as an optimal plasticizer in PLA, and the family of *O*-pentanoylated pentyl esters (**12a**-**12d**), identified as an optimal plasticizer in PVC, as this would render the challenging separation and isolation of carboxylic acids **2a-2d** unnecessary. Of course, this is only relevant if the mixture of plasticizer candidates performs similarly in terms of plasticizing properties compared to the compounds individually.[8, 11d] To this end, a model mixture of the obtained mono- and dicarboxylated catechols (**2a**:**2b**:**2c**:**2d**) was made in the ratios similar to those obtained by the mechanochemical procedure (45:32:13:10), and derivatized to both **8a**-**8d** for application in PLA and **12a**-**12d** for application in PVC. For synthetic details and the determination of the relative ratios, see section 12.8.

10.1 Derivatization of model mixture (2a-2d) towards mixture of *O*-pentanoylated pentyl esters (12a-12d) and *O*-levulinoylated pentyl esters (8a-8d)

A model mixture of **2a**-**2d** with ratios 45:32:13:10 (**2a**:**2b**:**2c**:**2d**) was subjected to a Fischer esterification using an excess of *n*-pentanol. The resulting mixture of pentyl esters **6a**-**6d** was obtained in quantitative yield with an (almost) unchanged ratio of 47:31:12:10 (**6a**:**6b**:**6c**:**6d**) in a quantitative mass-based yield (for details, see section 12.8). Next, the crude mixture of **6a**-**6d** was further derivatized to **12a**-**12d** using the optimal procedure for major compound **12a** as described in Table S7. Gratifyingly, the resulting mixture of **12a**-**12d** was obtained with a ratio of 47:32:11:10 (**12a**:**12b**:**12c**:**12d**) in 79% mass-based yield (for details, see section 12.8). A quick filtration over a silica plug was necessary to remove the last inorganic impurities, which slightly influenced the performance of this mixture as plasticizer (T_g = 61.4 °C instead of 63.6 °C before filtration, recorded in PVC).

When the mixture of pentyl esters **6a**-**6d** was *O*-levulinoylated using pseudo-levulinoyl chloride **13**, multiple unexpected signals appeared in the ^{1}H NMR spectrum, indicating undesired monolevulinoylation and/or the presence of acetal adducts (see Table S14). Hence, this route was ultimately abandoned and Steglich esterification using EDC•HCl was revisited. Unfortunately, Steglich esterification using 3 equiv. of levulinoyl chloride and 3 equiv. of EDC•HCl still showed monolevulinoylation (Table S16, entry 1). Upon reapplying the reaction conditions on the resulting mixture of mono- and dilevulinoylated products, full conversion to the dilevulinoylated products **8a**-**8d** was observed (entry 2) in a ratio of 47:30:13:10 (**8a**:**8b**:**8c**:**8d**) and a mass-based yield of 71 %. Once again, filtration over a silica plug was necessary to remove the last inorganic impurities (T_g = 49.7 °C instead of 51.5 °C before filtration, recorded in PLA).

For practical reasons, a single-step protocol for the *O*-acylation of **6a**-**6d** to **8a**-**8d** was pursued. Increasing the temperature to 40 °C did not solve the incomplete conversion (entry 3). To allow dilevulinoylation in a single step, 5 equiv. of EDC•HCl and levulinic acid had to be used, generating the desired mixture of plasticizers **8a**-**8d** in high yield

with a 47:31:12:10 (**8a**:**8b**:**8c**:**8d**) (entry 4). These reaction conditions will be used for the derivatization of the final mixture.

Table S16: Optimization of the *O*-levulinoylation of a mixture of 6a-6d to a mixture of 8a-8d by Steglich esterification with levulinic acid.

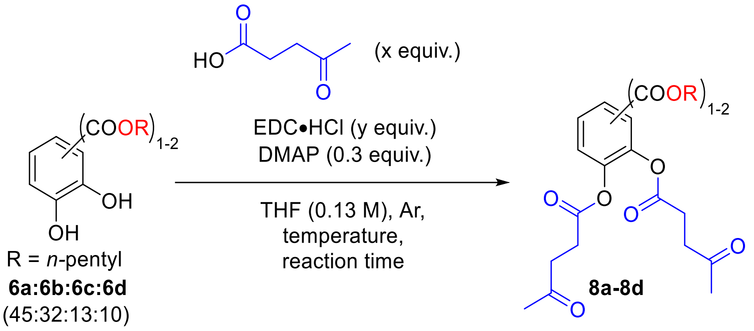

Entry	Scale	Reaction time (h)	Levulinic acid (equiv.)	EDC•HCl (equiv.)	Temperature (°C)	Yield (%)[a]	ELN-code
1	0.94 mmol	23 h	3.00	3.00	RT	Incomplete conversion	AGO-2024-0006
2[b]	0.94 mmol	2 × 23 h	3.00 + 3.00	3.00 + 3.00	RT	78 (71)[c]	AGO-2024-0006
3	0.77 mmol	21 h	3.00	3.00	40	Incomplete conversion	AGO-2024-0008
4	1.00 mmol	20 h	5.00	5.00	RT	92[d]	AGO-2024-0009

[a] Mass-based yield calculated as the mass of the fully converted ^{1}H NMR pure product mixture divided by the theoretical mass obtainable; [b] Result of entry 1 subjected to identical conditions; [c] Mass-based yield after filtration over silica plug (4 cm). Ratios of product mixture 47:30:13:10 (**8a**:**8b**:**8c**:**8d**); [d] Ratio of product mixture 47:31:12:10 (**8a**:**8b**:**8c**:**8d**).

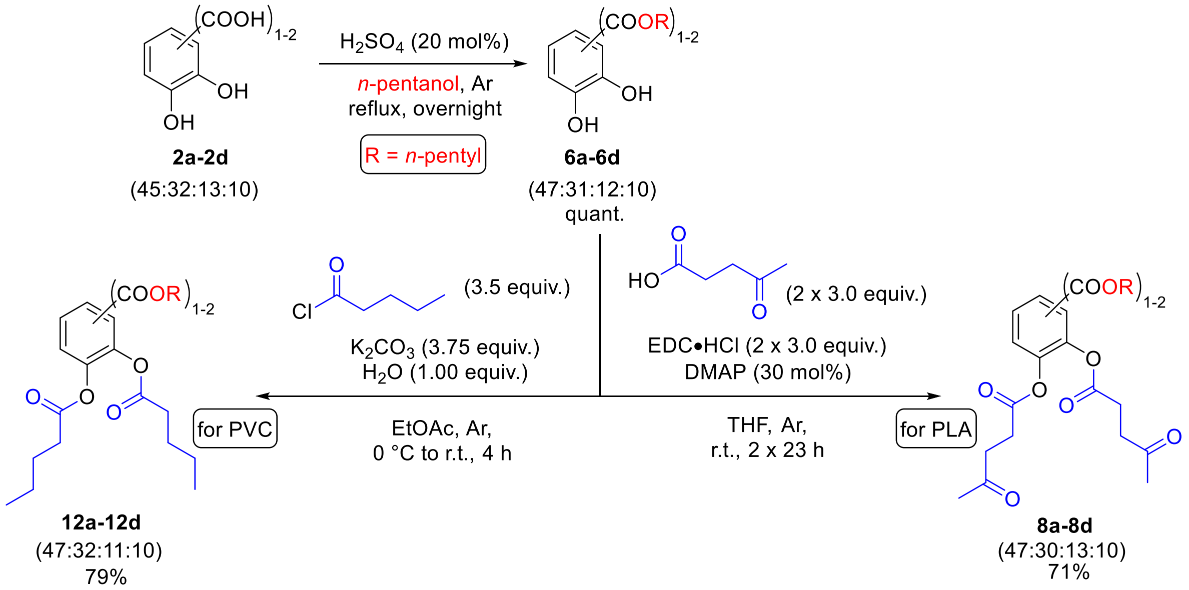

Scheme S14: Derivatizations of mixture of 2a-2d, mimicking a ball mill-derived mixture, towards mixture of *O*-pentanoylated pentyl esters 12a-12d and *O*-levulinoylated pentyl esters 8a-8d. For synthetic details, see section 12.8.

10.2 Comparison of the plasticizer efficiency of mixtures of *O*-pentanoylated pentyl esters (12a-12d) and *O*-levulinoylated pentyl esters (8a-8d) with each individual pure component

Next, the plasticizer performance of the synthesized mixture of *O*-pentanoylated pentyl esters (**12a-12d**) and that of the mixture of *O*-levulinoylated pentyl esters (**8a-8d**) was compared to each of their respective pure components (Figure S29). Gratifyingly, the plasticizer performance of the mixture of **12a-12d** is similar (T_g = 61.4 °C vs. 58.7–62.4 °C) to the weighted average of that of the pure plasticizers **12a-12d** (Figure S29, A) in PVC. The same behavior is observed for the mixture of **8a-8d** (T_g = 49.7 °C vs. 49.0–50.6 °C), which surpasses the benchmarks just like each of the respective components **8a-8d** (Figure S29, B). Gratifyingly, the mixtures perform similar to the best compounds individually, meaning that the challenging separation of the carboxylic acids (**2a-2d**) can be avoided.

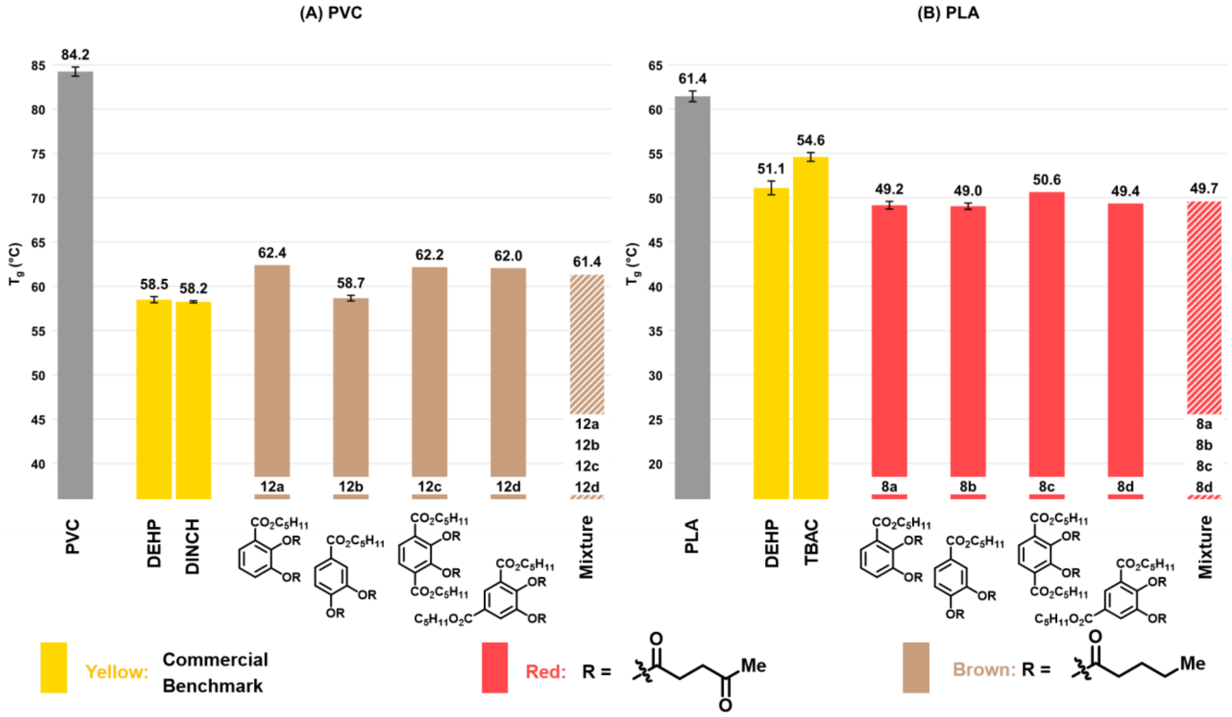

Figure S29: Plasticizing efficiency of (A) mixture of *O*-pentanoylated pentyl esters (12a-12d) compared to their individual performance in PVC (A) and (B) mixture of *O*-levulinoylated pentyl esters (8a-8d) compared to their individual performance in PLA. Glass transition temperature (T_g) determined using Differential Scanning Calorimetry (DSC) of polymers containing 10 wt% of the respective plasticizing candidate, mixed by solution casting (see section 4). T_g values for pure polymer, benchmark plasticizers and the best performing individual plasticizers (**12b** in PVC and **8a**, **8b** in PLA) are shown as the average of four measurements with standard deviation. For mixtures **12a-12d** in PVC and **8a-8d** in PLA, measurements were conducted twice and the average is reported. All other T_g values originate from single measurements.

10.3 Evaluation of the plasticizer efficiency at different concentrations

To demonstrate the dynamic range of our plasticizers, mixtures **12a-12d** and **8a-8d** were respectively mixed with PVC and PLA in a loading of both 5 wt% and 20 wt%. This enabled evaluation of how sensitively the glass transition temperature (T_g) responds to plasticizer concentration. As expected, the T_g values at 5 wt% fall between those of the unplasticized polymer and the corresponding 10 wt% loadings for both PVC [T_g (°C): 61.4 < 71.4 < 84.2] and PLA [T_g (°C): 49.7 < 56.1 < 61.4]. At 5 wt% in PVC, mixture **12a-12d** performs slightly below the benchmark plasticizers, giving T_g = 71.4 °C compared with T_g = 69.4–70.0 °C for DEHP and DINCH. In contrast, in PLA, mixture **8a-8d** (T_g = 56.1 °C) surpasses the benchmarks (57.2–58.0 °C for DEHP and TBAC). This is consistent with their relative behavior at 10 wt%.

Importantly, both mixtures induce a further decrease in T_g at 20 wt% loading. In PLA at 20 wt%, mixture **8a-8d** (T_g = 37.2 °C) again outperforms the benchmarks (41.3–43.8 °C for DEHP and TBAC), while in PVC the mixture **12a-12d** (T_g = 34.6 °C) delivers T_g values closely aligned to those of DEHP and DINCH (34.4–36.0 °C).

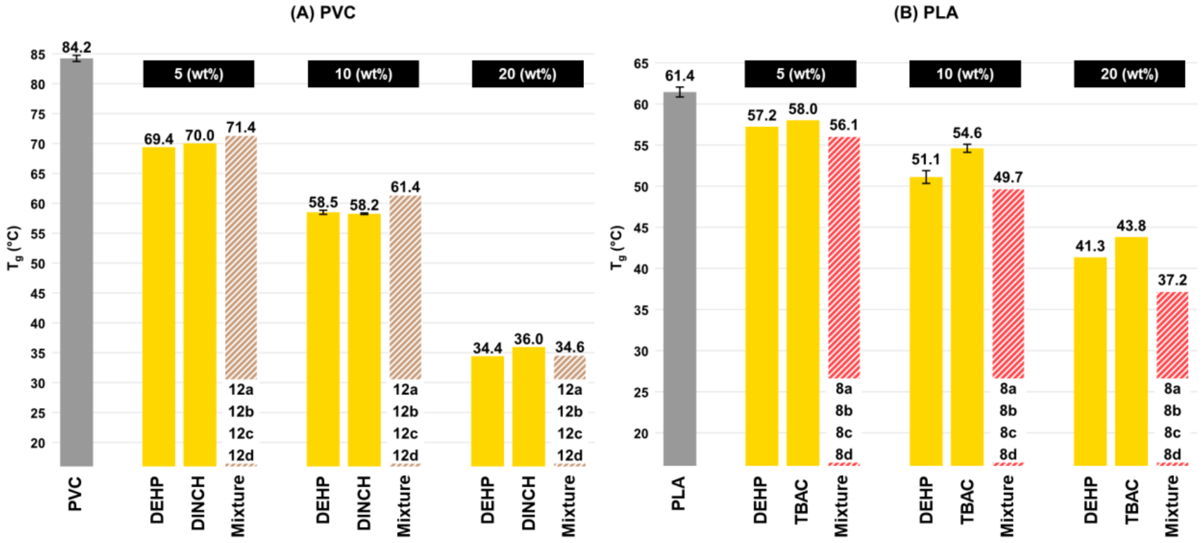

Figure S30: Effect of plasticizer loading on plasticizing efficiency of (A) mixture of *O*-pentanoylated pentyl esters (12a-12d) in PVC and (B) mixture of *O*-levulinoylated pentyl esters (8a-8d) in PLA. Glass transition temperature (T_g) determined using Differential Scanning Calorimetry (DSC) of polymers containing 5-20 wt% of the respective commercial benchmark plasticizer or plasticizer mixture, mixed by solution casting (see section 4). T_g values for pure polymer and benchmark plasticizers at a 10 wt% loading are shown as the average of four measurements with standard deviation. For mixtures **12a-12d** in PVC and **8a-8d** in PLA at the 10 wt% loading, measurements were conducted twice and the average is reported. All other T_g values originate from single measurements.

11. Derivatization and plasticizer performance of ball mill-derived plasticizer mixtures

Finally, a ball mill-derived mixture of **2a-2d** with ratios 46:31:12:11 (**2a**:**2b**:**2c**:**2d**) (see section 3.3) was derivatized to the family of *O*-levulinoylated pentyl esters (**8a-8d**), identified as an optimal plasticizer in PLA and the family of *O*-pentanoylated pentyl esters (**12a-12d**), identified as an optimal plasticizer in PVC. To this end, the synthetic procedures as described in section 10 were applied (Scheme S15). Once again, mixtures of **8a-8d** and **12a-12d** were obtained in two steps with almost identical ratios of their respective components compared to the starting mixture with high yields, and without requiring column chromatography. For synthetic details and the determination of the relative ratios, see section 12.9.

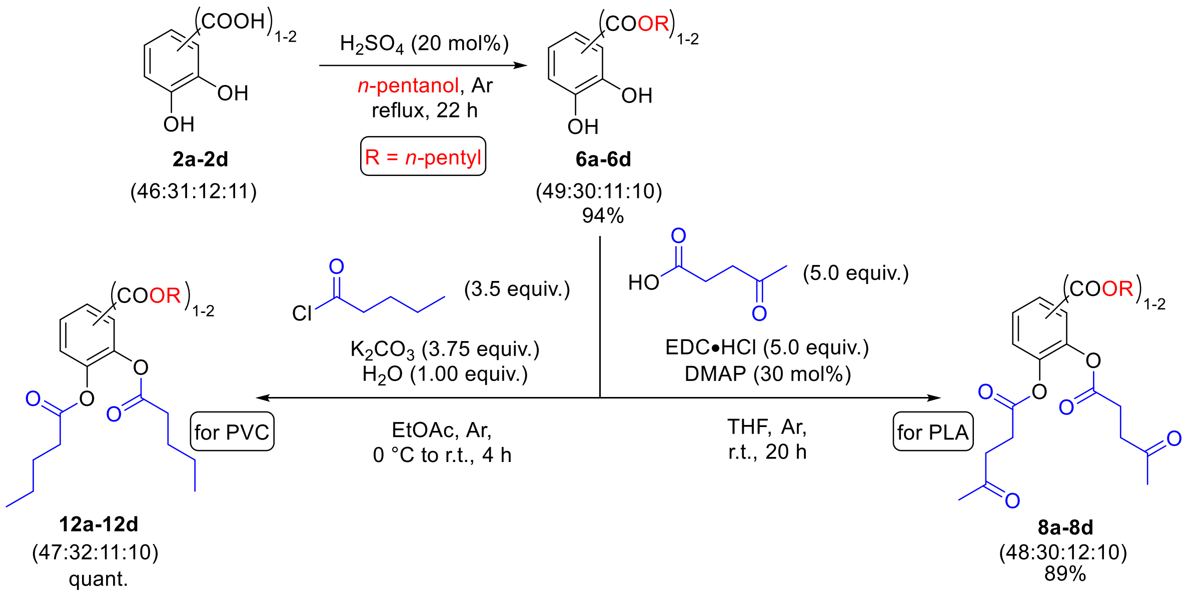

Scheme S15: Derivatizations of a ball mill-derived mixture of carboxylated catechols 2a-2d, towards mixture of *O*-pentanoylated pentyl esters 12a-12d and *O*-levulinoylated pentyl esters 8a-8d. For synthetic details, see section 12.9.

In PVC, the ball mill-derived mixture of *O*-pentanoylated pentyl esters (**12a-12d**) resulted in a T_g of (61.8 ± 0.8) °C, roughly corresponding to the weighted average of the T_g's of the individual compounds (Figure S31, A). In PLA, the ball mill-derived mixture of *O*-levulinoylated pentyl esters (**8a-8d**) resulted in a T_g of (49.6 ± 0.5) °C, which is comparable with each of the individual components and outperforms the commercial benchmarks in PLA (Figure S31, B). Gratifyingly, the mixtures perform similarly to the individual compounds, meaning that the challenging separation of the carboxylic acids (**2a-2d**) can be avoided.

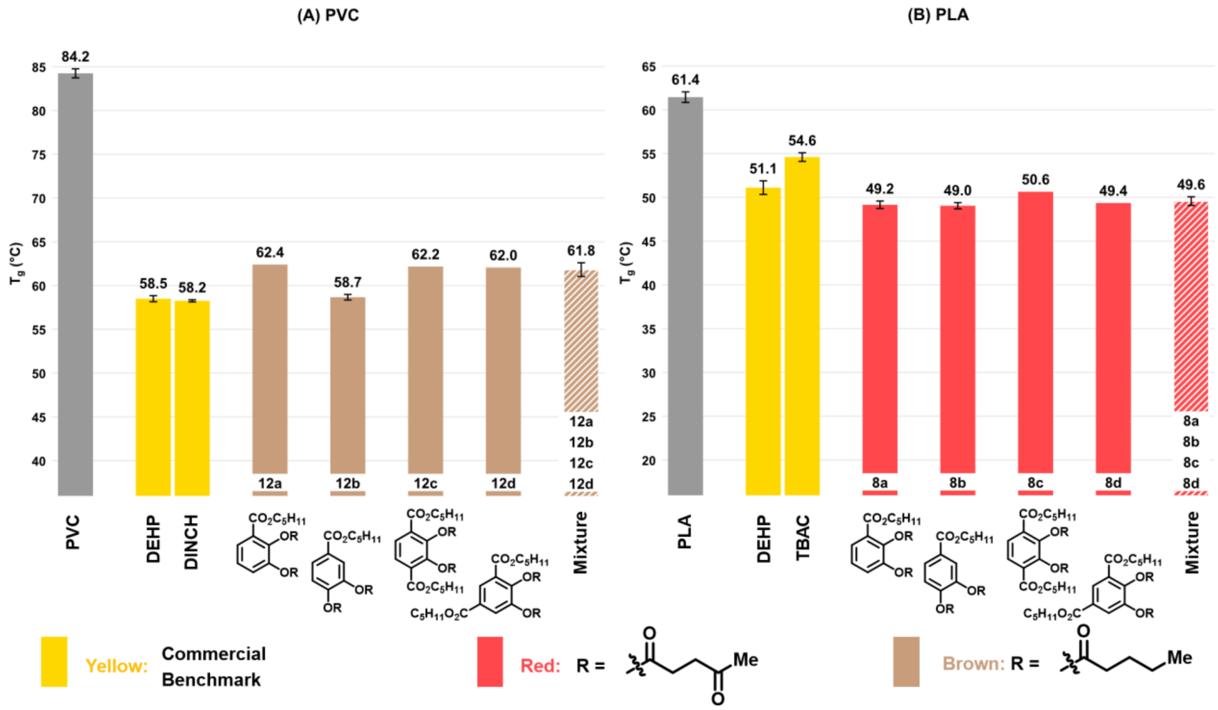

Figure S31: Plasticizing efficiency of (A) *O*-pentanoylated pentyl esters (12a-12d) in PVC and (B) *O*-levulinoylated pentyl esters (8a-8d) in PLA and application of ball mill-derived mixture. Glass transition temperature (T_g) determined using Differential Scanning Calorimetry (DSC) of polymers containing 10 wt% of the respective plasticizing candidate, mixed by solution casting (see section 4). T_g values for pure polymer, benchmark plasticizers, the best performing individual plasticizers (**12b** in PVC and **8a**, **8b** in PLA) and the ball mill-derived mixtures **12a**-**12d** in PVC and **8a**-**8d** in PLA are shown as the average of four measurements with standard deviation. All other T_g values originate from single measurements.

12. Synthetic protocols and characterization of plasticizer candidates

12.1 General procedures

These procedures were used to synthesize the individual components for evaluating their plasticizing effects. For synthetic details regarding the plasticizing mixtures, see section 12.8-12.9.

General procedure A (esterification with pentan-1-ol)

The reaction was performed according to a modified literature procedure.[14] First, approximately 55 mL of pentan-1-ol was degassed to avoid oxidation of the catechol materials by sparging with argon under ultrasonication for 15 minutes. The suitable hydroxybenzoic acid (5.00 mmol, 1.00 equiv.) was dissolved in degassed pentan-1-ol (50 mL) and sulfuric acid (0.06 mL, 1.00 mmol, 20 mol%) was added. The reaction mixture was stirred overnight at reflux temperature under an argon atmosphere. By TLC analysis a full conversion was demonstrated. The mixture was concentrated under reduced pressure and then water (50 mL) was added. The obtained aqueous phase was extracted with MTBE (3 × 40 mL). The organic phases were combined and washed with a saturated, aqueous $NaHCO_3$ solution (2 × 30 mL) and brine (1 × 30 mL). The organic phase was dried over anhydrous Na_2SO_4, filtered, and then concentrated under reduced pressure. The resulting crude residue was purified by automated flash chromatography to obtain the corresponding pure pentyl ester.

General procedure B (*O*-acylation with acetyl chloride or pentanoyl chloride)

The reaction was performed according to a modified literature procedure.[11c, 24] The suitable dihydroxybenzoate (1.00 equiv.) and triethylamine (3.00 equiv.) were dissolved in CH_2Cl_2 (0.67 – 1.25 mL/mmol) or THF (1.68 – 2.80 mL/mmol). The mixture was stirred in an ice bath. Under an argon atmosphere, the respective acyl chloride (3.00 equiv.) was added dropwise into the reaction mixture over a time span of 20 minutes. Subsequently, the reaction mixture was allowed to warm to room temperature and was stirred overnight. The reaction was terminated after full conversion as demonstrated by TLC analysis. The reaction mixture was quenched with H_2O (15 mL), EtOAc (15 mL) was added, and the phases were separated. The organic phase was washed with distilled H_2O (1 × 15 mL) and a saturated, aqueous $NaHCO_3$ solution (2 × 15 mL). Then the mixture was dried over anhydrous Na_2SO_4, filtered, and concentrated under reduced pressure. The resulting crude residue was purified by automated flash chromatography to obtain the corresponding pure acylated derivative.

General procedure C (*O*-levulinoylation)

The reaction was performed according to a modified literature procedure.[17, 25] Levulinic acid (3.00 equiv.), EDC•HCl (3.00 equiv.) and DMAP (30 mol%) were dissolved in CH_2Cl_2 (7.24 – 9.38 mL/mmol) or THF (5.38 – 7.85 mL/mmol). The suitable dihydroxybenzoate (1.00 equiv.) was added and the reaction mixture was stirred overnight at room temperature under an argon atmosphere. The reaction was terminated after full conversion as demonstrated by TLC analysis. The solvent was removed under reduced pressure and the residue was dissolved in EtOAc (30 mL). The obtained organic phase was subsequently washed with an aqueous 1 M HCl solution (2 × 30 mL) and a saturated, aqueous $NaHCO_3$ solution (2 × 30 mL). The organic layer was dried over anhydrous Na_2SO_4, filtered, and concentrated under

reduced pressure. The resulting crude residue was purified by automated flash chromatography to obtain the corresponding pure levulinoylated derivative.

12.2 Experimental protocols and characterization of plasticizer candidates 3a-11a

Methyl 2,3-dihydroxybenzoate (3a) [DDV-AG-001]

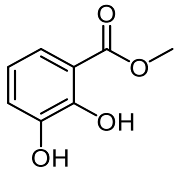

3a

A modified literature procedure was followed.[13] First, approximately 20 mL of MeOH was degassed to avoid oxidation of the catechol materials by sparging with argon under ultrasonication for 15 minutes. A solution of 2,3-dihydroxybenzoic acid (**3a**) (2.00 g, 13.00 mmol, 1.00 equiv.) in degassed MeOH (17 mL) and sulfuric acid (0.71 mL, 13.00 mmol, 1.00 equiv.) was refluxed for 22 hours under an argon atmosphere. After the reaction time, although the presence of starting material was still detected by TLC analysis, the work-up was nevertheless started. The solvent was removed under reduced pressure and the residue was dissolved in EtOAc (70 mL). The obtained organic phase was washed with a saturated, aqueous solution of $NaHCO_3$ (2 × 40 mL) and brine (1 × 30 mL), dried over anhydrous Na_2SO_4, and filtered. The solvent was removed under reduced pressure to afford methyl 2,3-dihydroxybenzoate (**39**) as an off-white solid in 89% yield (1.95 g, 11.62 mmol). Spectroscopic data is in accordance with the literature.[26]

Off-white solid, R_f = 0.73 (EtOAc/heptane 50:50), m.p. 78.5 °C. ^{1}H NMR (400 MHz, $CDCl_3$) δ_H: 10.89 (s, 1H), 7.35 (dd, *J* = 8.1, 1.4 Hz, 1H), 7.10 (dd, *J* = 7.9, 1.3 Hz, 1H), 6.78 (t, *J* = 8.0 Hz, 1H), 5.79 (s, 1H), 3.94 (s, 3H) ppm. ^{13}C{^{1}H} NMR (101 MHz, $CDCl_3$) δ_C: 170.9 (C), 149.0 (C), 145.2 (C), 120.7 (CH), 120.0 (CH), 119.3 (CH), 112.5 (C), 52.5 (CH_3) ppm.

Pentyl 2,3-dihydroxybenzoate (6a) [DDV-AG-006]

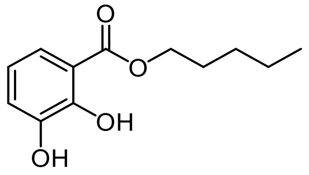

6a

The *title compound* **6a** was synthesized using 2,3-dihydroxybenzoic acid (**2a**) (0.77 g, 5.00 mmol, 1.00 equiv.), following General procedure A. The resulting crude residue was purified by automated flash chromatography (Büchi Pure C-850 Flashprep, 80 g silica cartridge, 40 mL/min flowrate, eluent: 100% heptane to 80% heptane/20% EtOAc over 50 min) to obtain pentyl 2,3-dihydroxybenzoate (**42**) as a colorless oil in 90% yield (1.01 g, 4.49 mmol). Spectroscopic data is in accordance with the literature.[14]

Colorless oil, R_f = 0.66 (EtOAc/heptane 30:70). ^{1}H NMR (400 MHz, $CDCl_3$) δ_H: 10.98 (s, 1H), 7.37 (dd, *J* = 8.1, 1.5 Hz, 1H), 7.10 (dd, *J* = 7.9, 1.5 Hz, 1H), 6.79 (t, *J* = 8.0 Hz, 1H), 5.64 (s, 1H), 4.35 (t, *J* = 6.7 Hz, 2H), 1.83 – 1.74 (m, 2H), 1.47 – 1.35 (m, 4H), 0.94 (t, *J* = 7.1 Hz, 3H) ppm. ^{13}C{^{1}H} NMR (101 MHz, $CDCl_3$) δ_C: 170.5 (C), 148.9 (C), 145.1 (C), 120.6 (CH), 119.7 (CH), 119.1 (CH), 112.7 (C), 65.7 (CH_2), 28.3 (CH_2), 28.1 (CH_2), 22.3 (CH_2), 14.0 (CH_3) ppm.

2-Ethylhexyl 2,3-dihydroxybenzoate (9a) [DDV-AG-005]

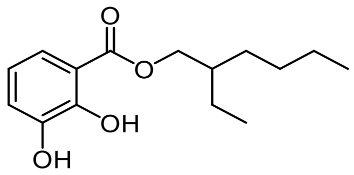

9a

The reaction was performed according to a modified literature procedure.[15] First, approximately 15 mL of *p*-xylene was degassed to avoid oxidation of the catechol materials by sparging with argon under ultrasonication for 15 minutes. 2-Ethylhexan-1-ol (0.82 g, 6.30 mmol, 1.05 equiv.), 2,3-dihydroxybenzoic acid (**2a**) (0.93 g, 6.00 mmol, 1.00 equiv.) and *p*-toluenesulfonic acid (0.23 g, 1.20 mmol, 20 mol%) were added to *p*-xylene (10 mL). The reaction was stirred at reflux temperature under an argon atmosphere for 19 hours. After the reaction time, although the presence of starting material was still detected by TLC analysis, the work-up was nevertheless started. The reaction mixture was dissolved in MTBE (20 mL). The obtained organic phase was subsequently washed with a saturated, aqueous $NaHCO_3$ solution (2 × 20 mL) and brine (1 × 20 mL), dried over anhydrous Na_2SO_4 and filtered. The mixture was concentrated under reduced pressure and purified by automated flash chromatography (Büchi Pure C-850 Flashprep, 80 g silica cartridge, 40 mL/min flowrate, eluent: 100% heptane to 80% heptane/20% EtOAc over 50 min, 80% heptane/20% EtOAc isocratic over 10 min) to obtain 2-ethylhexyl 2,3-dihydroxybenzoate (**9a**) as a yellow oil in 75% yield (1.20 g, 4.50 mmol). No spectroscopic data was available in literature.

Yellow oil, R_f = 0.67 (EtOAc/heptane 30:70). ^{1}H NMR (400 MHz, $CDCl_3$) δ_H: 10.99 (d, *J* = 0.5 Hz, 1H), 7.36 (dd, *J* = 8.1, 1.5 Hz, 1H), 7.10 (ddd, *J* = 7.9, 1.5, 0.5 Hz, 1H), 6.80 (t, *J* = 8.0 Hz, 1H), 5.64 (s, 1H), 4.32 – 4.23 (m, 2H), 1.79 – 1.68 (m, 1H), 1.50 – 1.29 (m, 8H), 0.95 (t, *J* = 7.5 Hz, 3H), 0.93 – 0.89 (m, 3H) ppm. ^{13}C{^{1}H} NMR (101 MHz, $CDCl_3$) δ_C: 170.5 (C), 148.9 (C), 145.1 (C), 120.5 (CH), 119.7 (CH), 119.2 (CH), 112.8 (C), 67.9 (CH_2), 38.9 (CH), 30.5 (CH_2), 29.0 (CH_2), 24.0 (CH_2), 23.0 (CH_2), 14.0 (CH_3), 11.1 (CH_3) ppm.

Methyl 2,3-bis(acetyloxy)benzoate (4a) [DDV-AG-026]

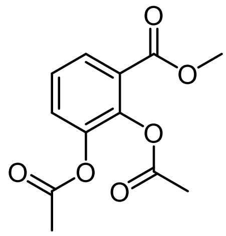

4a

The *title compound* **4a** was synthesized using methyl 2,3-dihydroxybenzoate (**3a**) (250 mg, 1.49 mmol, 1.00 equiv.), triethylamine (451 mg, 4.46 mmol, 3.00 equiv.), acetyl chloride (0.32 mL, 4.46 mmol, 3.00 equiv.) and CH_2Cl_2 (1 mL), following General procedure B. The resulting crude residue was purified by automated flash chromatography (Büchi Pure C-850 Flashprep, 40 g silica cartridge, 40 mL/min flowrate, eluent: 100% heptane to 80% heptane/20% EtOAc over 50 min) to obtain methyl 2,3-bis(acetyloxy)benzoate (**4a**) as a white solid in 91% yield (343 mg, 1.36 mmol). No spectroscopic data was available in literature.

White solid, R_f = 0.60 (EtOAc/heptane 50:50), m.p. 56.5 °C. ^{1}H NMR (400 MHz, $CDCl_3$) δ_H: 7.89 (dd, *J* = 7.8, 1.7 Hz, 1H), 7.38 (dd, *J* = 8.1, 1.7 Hz, 1H), 7.31 (t, *J* = 8.0 Hz, 1H), 3.87 (s, 3H), 2.34 (s, 3H), 2.31 (s, 3H) ppm. ^{13}C{^{1}H} NMR (101 MHz, $CDCl_3$) δ_C: 168.5 (C), 168.3 (C), 164.5 (C), 143.7 (C), 142.9 (C), 129.0 (CH), 127.9 (CH), 126.2 (CH), 125.0 (C), 52.5 (CH_3), 20.7 (CH_3), 20.7 (CH_3) ppm. HRMS (ESI) for $C_{12}H_{12}O_6Na$ $[M+Na]^+$, calcd 275.0526, found 275.0533.

Pentyl 2,3-bis(acetyloxy)benzoate (7a) [DDV-AG-010]

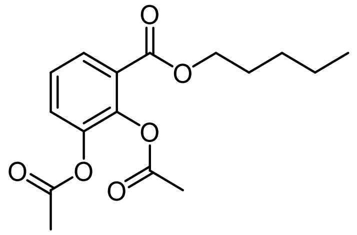

7a

The *title compound* **7a** was synthesized using pentyl 2,3-dihydroxybenzoate (**6a**) (200 mg, 0.89 mmol, 1.00 equiv.), triethylamine (271 mg, 2.68 mmol, 3.00 equiv.), acetyl chloride (0.19 mL, 2.68 mmol, 3.00 equiv.) and CH_2Cl_2 (1 mL), following General procedure B. The resulting crude residue was purified by automated flash chromatography (Büchi Pure C-850 Flashprep, 12 g silica cartridge, 30 mL/min flowrate, eluent: 100% heptane to 80% heptane/20% EtOAc over 50 min) to obtain pentyl 2,3-bis(acetyloxy)benzoate (**51**) as a colorless oil in 72% yield (199 mg, 0.64 mmol). No spectroscopic data was available in literature.

Colorless oil, R_f = 0.48 (EtOAc/heptane 30:70). ^{1}H NMR (400 MHz, $CDCl_3$) δ_H: 7.89 (dd, *J* = 7.8, 1.8 Hz, 1H), 7.37 (dd, *J* = 8.1, 1.8 Hz, 1H), 7.30 (t, *J* = 7.9 Hz, 1H), 4.26 (t, *J* = 6.8 Hz, 2H), 2.33 (s, 3H), 2.30 (s, 3H), 1.73 (quint, *J* = 7.0 Hz, 2H), 1.44 – 1.33 (m, 4H), 0.92 (t, *J* = 7.1 Hz, 3H) ppm. ^{13}C{^{1}H} NMR (101 MHz, $CDCl_3$) δ_C: 168.3 (C), 168.2 (C), 164.0 (C), 143.6 (C), 142.7 (C), 128.9 (CH), 127.6 (CH), 126.0 (CH), 125.3 (C), 66.5 (CH_2), 28.3 (CH_2), 28.1 (CH_2), 22.3 (CH_2), 20.6 (CH_3), 20.6 (CH_3), 13.9 (CH_3) ppm. HRMS (ESI) for $C_{16}H_{20}O_6Na$ $[M+Na]^+$, calcd 331.1152, found 331.1161.

2-Ethylhexyl 2,3-bis(acetyloxy)benzoate (10a) [DDV-AG-016]

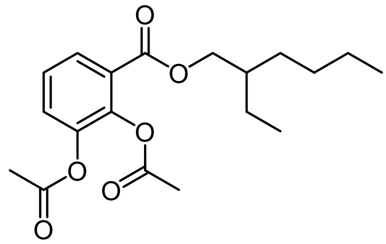

10a

The *title compound* **10a** was synthesized using 2-ethylhexyl 2,3-dihydroxybenzoate (**9a**) (250 mg, 0.94 mmol, 1.00 equiv.), triethylamine (285 mg, 2.82 mmol, 3.00 equiv.), acetyl chloride (0.20 mL, 2.82 mmol, 3.00 equiv.) and CH_2Cl_2 (1 mL), following General procedure B. The resulting crude residue was purified by automated flash chromatography (Büchi Pure C-850 Flashprep, 40 g silica cartridge, 35 mL/min flowrate, eluent: 100% heptane to 80% heptane/20% EtOAc over 50 min) to obtain 2-ethylhexyl 2,3-bis(acetyloxy)benzoate (**10a**) as a colorless oil in 86% yield (284 mg, 0.81 mmol). No spectroscopic data was available in literature.

Colorless oil, R_f = 0.52 (EtOAc/heptane 30:70). ^{1}H NMR (400 MHz, $CDCl_3$) δ_H: 7.87 (dd, *J* = 7.8, 1.8 Hz, 1H), 7.37 (dd, *J* = 8.1, 1.8 Hz, 1H), 7.31 (t, *J* = 7.9 Hz, 1H), 4.23 – 4.14 (m, 2H), 2.34 (s, 3H), 2.30 (s, 3H), 1.73 – 1.63 (m, 1H), 1.47 – 1.27 (m, 8H), 0.95 – 0.88 (m, 6H) ppm. ^{13}C{^{1}H} NMR (101 MHz, $CDCl_3$) δ_C: 168.4 (C), 168.2 (C), 163.9 (C), 143.6 (C), 142.8 (C), 128.7 (CH), 127.6 (CH), 126.0 (CH), 125.3 (C), 67.8 (CH_2), 38.9 (CH), 30.4 (CH_2), 28.9 (CH_2), 23.8 (CH_2), 23.0 (CH_2), 20.6 (CH_3), 20.6 (CH_3), 14.0 (CH_3), 11.0 (CH_3) ppm. HRMS (ESI) for $C_{19}H_{26}O_6Na$ [M+Na]$^+$, calcd 373.1622, found 373.1630.

Methyl 2,3-bis[(4-oxopentanoyl)oxy]benzoate (5a) [DDV-AG-032]

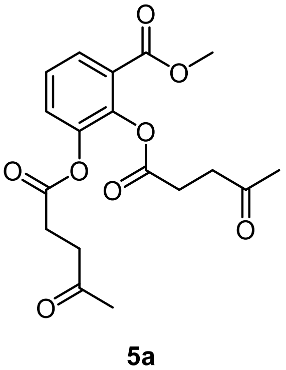

5a

The *title compound* **5a** was synthesized using methyl 2,3-dihydroxybenzoate (**3a**) (250 mg, 1.49 mmol, 1.00 equiv.), levulinic acid (518 mg, 4.46 mmol, 3.00 equiv.), EDC•HCl (855 mg, 4.46 mmol, 3.00 equiv.), DMAP (54.5 mg, 0.45 mmol, 30 mol%), and CH_2Cl_2 (11 mL), following General procedure C. The resulting crude residue was purified by automated flash chromatography (Büchi Pure C-850 Flashprep, 12 g silica cartridge, 30 mL/min flowrate, eluent: 100% heptane to 50% heptane/50% EtOAc over 40 min, 50% heptane/50% EtOAc isocratic over 20 min) to obtain methyl 2,3-bis[(4-oxopentanoyl)oxy]benzoate (**5a**) as a colorless oil in 94% yield (509 mg, 1.40 mmol). No spectroscopic data was available in literature.

Colorless oil, R_f = 0.21 (EtOAc/heptane 50:50). ^1H NMR (400 MHz, $CDCl_3$) δ_H: 7.86 (dd, J = 7.8, 1.7 Hz, 1H), 7.36 (dd, J = 8.1, 1.8 Hz, 1H), 7.29 (t, J = 6.9 Hz, 1H), 3.86 (s, 3H), 2.96 – 2.91 (m, 4H), 2.90 – 2.85 (m, 4H), 2.21 (s, 6H) ppm. $^{13}C\{^1H\}$ NMR (101 MHz, $CDCl_3$) δ_C: 206.3 (C)*, 170.6 (C), 170.5 (C), 164.4 (C), 143.8 (C), 142.8 (C), 128.9 (CH), 127.8 (CH), 126.1 (CH), 124.8 (C), 52.4 (CH_3), 37.8 (CH_2), 37.7 (CH_2), 29.9 (CH_3), 29.8 (CH_3), 27.8 (CH_2), 27.7 (CH_2) ppm. HRMS (ESI) for $C_{18}H_{20}O_8Na$ $[M+Na]^+$, calcd 387.1050, found 387.1064.

**One quaternary carbon signal is missing. The signal at 206.3 ppm assumably belongs to the carbonyl carbon signals of both ketone functionalities.*

Pentyl 2,3-bis[(4-oxopentanoyl)oxy]benzoate (8a) [DDV-AG-012]

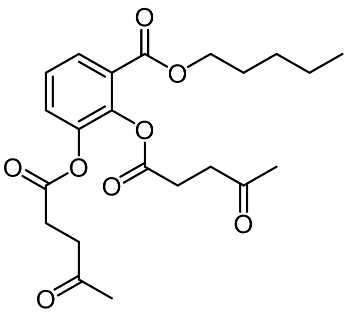

8a

The *title compound* **8a** was synthesized using pentyl 2,3-dihydroxybenzoate (**6a**) (224 mg, 1.00 mmol, 1.00 equiv.), levulinic acid (348 mg, 3.00 mmol, 3.00 equiv.), EDC•HCl (575 mg, 3.00 mmol, 3.00 equiv.), DMAP (36.0 mg, 0.30 mmol, 30 mol%) and CH_2Cl_2 (7.5 mL), following General procedure C. The resulting crude residue was purified by automated flash chromatography (Büchi Pure C-850 Flashprep, 12 g silica cartridge, 30 mL/min flowrate, eluent: 100% heptane to 70% heptane/30% EtOAc over 50 min, 70% heptane/30% EtOAc isocratic over 15 min) to obtain pentyl 2,3-bis[(4-oxopentanoyl)oxy]benzoate (**8a**) as a colorless oil in 87% yield (365 mg, 0.87 mmol). No spectroscopic data was available in literature.

Colorless oil, R_f = 0.08 (EtOAc/heptane 30:70). ^1H NMR (400 MHz, $CDCl_3$) δ_H: 7.86 (dd, J = 7.8, 1.8 Hz, 1H), 7.35 (dd, J = 8.1, 1.8 Hz, 1H), 7.29 (t, J = 7.6 Hz, 1H), 4.25 (t, J = 6.8, 2H), 2.97 – 2.91 (m, 4H), 2.90 – 2.85 (m, 4H), 2.21 (s, 3H), 2.20 (s, 3H), 1.75 – 1.69 (m, 2H), 1.43 – 1.34 (m, 4H), 0.94 – 0.90 (m, 3H) ppm. $^{13}C\{^1H\}$ NMR (101 MHz, $CDCl_3$) δ_C: 206.3 (C), 206.3 (C), 170.6 (C), 170.5 (C), 163.9 (C), 143.7 (C), 142.8 (C), 128.8 (CH), 127.7 (CH), 126.1 (CH), 125.1 (C), 65.5 (CH_2), 37.8 (CH_2), 37.7 (CH_2), 29.9 (CH_3), 29.8 (CH_3), 28.3 (CH_2), 28.1 (CH_2), 27.8 (CH_2), 27.8 (CH_2), 22.3 (CH_2), 14.0 (CH_3) ppm. HRMS (ESI) for $C_{22}H_{28}O_8Na$ $[M+Na]^+$, calcd 443.1676, found 443.1694.

2-Ethylhexyl 2,3-bis[(4-oxopentanoyl)oxy]benzoate (11a) [DDV-AG-020]

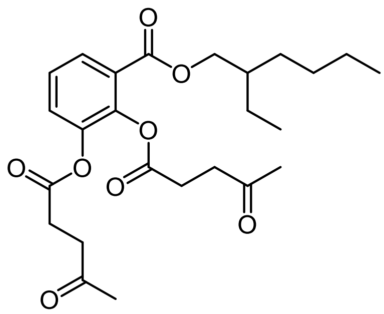

11a

The *title compound* **11a** was synthesized using 2-ethylhexyl 2,3-dihydroxybenzoate (**9a**) (266 mg, 1.00 mmol, 1.00 equiv.), levulinic acid (348 mg, 3.00 mmol, 3.00 equiv.), EDC•HCl (575 mg, 3.00 mmol, 3.00 equiv.), DMAP (37.0 mg, 0.30 mmol, 30 mol%) and CH_2Cl_2 (7.5 mL), following General procedure C. The resulting crude residue was purified by automated flash chromatography (Büchi Pure C-850 Flashprep, 40 g silica cartridge, 40 mL/min flowrate, eluent: 100% heptane to 60% heptane/40% EtOAc over 50 min) to obtain 2-ethylhexyl 2,3-bis[(4-oxopentanoyl)oxy]benzoate (**11a**) as a colorless oil in 83% yield (382 mg, 0.83 mmol). No spectroscopic data was available in literature.

Colorless oil, R_f = 0.11 (EtOAc/heptane 30:70). ^{1}H NMR (400 MHz, $CDCl_3$) δ_H: 7.84 (dd, *J* = 7.8, 1.7 Hz, 1H), 7.36 (dd, *J* = 8.1, 1.7 Hz, 1H), 7.29 (t, *J* = 8.0 Hz, 1H), 4.21 – 4.12 (m, 2H), 2.97 – 2.92 (m, 4H), 2.91 – 2.86 (m, 4H), 2.21 (s, 3H), 2.21 (s, 3H), 1.71 – 1.64 (m, 1H), 1.47 – 1.28 (m, 8H), 0.95 – 0.88 (m, 6H) ppm. ^{13}C{^{1}H} NMR (101 MHz, $CDCl_3$) δ_C: 206.3 (C), 206.3 (C), 170.6 (C), 170.5 (C), 163.8 (C), 143.8 (C), 143.0 (C), 128.6 (CH), 127.7 (CH), 126.1 (CH), 125.0 (C), 67.7 (CH_2), 38.8 (CH), 37.8 (CH_2), 37.7 (CH_2), 30.4 (CH_2), 29.8 (CH_3), 29.8 (CH_3), 28.9 (CH_2), 27.8 (CH_2), 27.8 (CH_2), 23.8 (CH_2), 23.0 (CH_2), 14.0 (CH_3), 11.0 (CH_3) ppm. HRMS (ESI) for $C_{25}H_{34}O_8Na$ $[M+Na]^+$, calcd 485.2146, found 485.2153.

12.3 Experimental protocols and characterization of plasticizer candidates 6b-6d and 9b-9d

Pentyl 3,4-dihydroxybenzoate (6b) [DDV-AG-007]

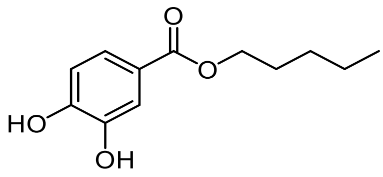

6b

The *title compound* **6b** was synthesized using 3,4-dihydroxybenzoic acid (**2b**) (0.77 g, 5.00 mmol, 1.00 equiv.), following General procedure A. The resulting crude residue was purified by automated flash chromatography (Büchi Pure C-850 Flashprep, 80 g silica cartridge, 40 mL/min flowrate, eluent: 100% heptane to 80% heptane/20% EtOAc over 50 min, 80% heptane/20% EtOAc isocratic over 20 min) to obtain pentyl 3,4-dihydroxybenzoate (**6b**) as a white solid in 73% yield (0.81 g, 3.63 mmol). Spectroscopic data is in accordance with literature.[27]

White solid, R_f = 0.30 (EtOAc/heptane 30:70), m.p. 97.5 °C. ^{1}H NMR (400 MHz, DMSO-d_6) δ_H: 9.51 (br s, 2H), 7.37 (d, J = 2.1 Hz, 1H), 7.31 (dd, J = 8.3, 2.1 Hz, 1H), 6.81 (d, J = 8.3 Hz, 1H), 4.17 (t, J = 6.6 Hz, 2H), 1.65 (quint, J = 6.8 Hz, 2H), 1.39 – 1.27 (m, 4H), 0.88 (t, J = 7.1 Hz, 3H) ppm. ^{13}C{^{1}H} NMR (101 MHz, DMSO-d_6) δ_C: 165.7 (C), 150.4 (C), 145.1 (C), 121.7 (CH), 120.8 (C), 116.3 (CH), 115.3 (CH), 64.0 (CH_2), 28.0 (CH_2), 27.7 (CH_2), 21.8 (CH_2), 13.8 (CH_3) ppm.

Dipentyl 2,3-dihydroxybenzene-1,4-dicarboxylate (6c) [DDV-AG-015]

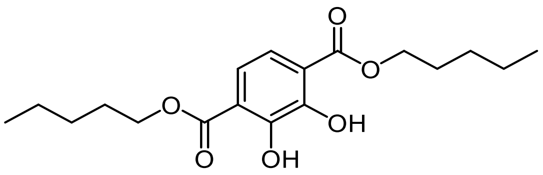

6c

The *title compound* **6c** was synthesized using 2,3-dihydroxybenzene-1,4-dicarboxylic acid (**2c**) (0.99 g, 5.00 mmol, 1.00 equiv.), following General procedure A with a slightly modified work-up. After a first washing step with a saturated, aqueous $NaHCO_3$ solution (1 × 30 mL), deprotonation of the product and subsequent migration to the aqueous phase was observed. Therefore, the aqueous phase was acidified, and a back-extraction was performed. A second washing with $NaHCO_3$ was not executed, instead distilled H_2O (1 × 30 mL) was used. No further changes were made to the general work-up procedure. After purification by automated flash chromatography (Büchi Pure C-850 Flashprep, 80 g silica cartridge, 40 mL/min flowrate, eluent: 100% heptane to 80% heptane/20% EtOAc over 50 min) dipentyl 2,3-dihydroxybenzene-1,4-dicarboxylate (**6c**) was obtained as a white solid in 76% yield (1.29 g, 3.81 mmol). No spectroscopic data was available in literature.

White solid, R_f = 0.74 (EtOAc/heptane 30:70), m.p. 48.5 °C. ^{1}H NMR (400 MHz, $CDCl_3$) δ_H: 10.99 (s, 2H), 7.32 (s, 2H), 4.37 (t, J = 6.7 Hz, 4H), 1.84 – 1.76 (m, 4H), 1.47 – 1.34 (m, 8H), 0.94 (t, J = 7.1 Hz, 6H) ppm. ^{13}C{^{1}H} NMR (101 MHz, $CDCl_3$) δ_C: 169.8 (C), 151.7 (C), 118.2 (CH), 116.1 (C), 66.1 (CH_2), 28.2 (CH_2), 28.1 (CH_2), 22.3 (CH_2), 14.0 (CH_3) ppm. HRMS (ESI) for $C_{18}H_{26}O_6Na$ $[M+Na]^+$, calcd 361.1622, found 361.1624.

Dipentyl 4,5-dihydroxyisophthalate (6d) [AGO-132-133-134]

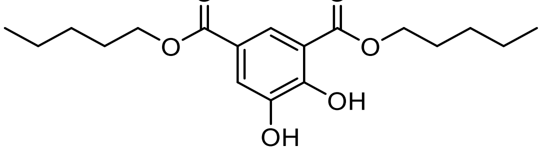

6d

Modified literature procedures were followed.[14, 28] 5-Hydroxyisophthalic acid (500 mg, 2.75 mmol) and concentrated sulfuric acid (6 mL) were added together and stirred at 0 °C. Subsequently, *N*-bromosuccinimide (513 mg, 2.88 mmol) was slowly added under vigorous stirring. Then, the resulting reaction mixture was heated to 50 °C and stirred for 2 h. After the reaction time, the solution was cooled to 0 °C and water (20 mL) was added. The aqueous phase was extracted with EtOAc (2 × 20 mL). The

organic phases were combined, dried over anhydrous Na_2SO_4, filtered, and evaporated under reduced pressure. The resulting crude product (726 mg), containing 4-bromo-5-hydroxyisophthalic acid as the major compound, was then dissolved in water (2.7 mL) and sodium carbonate (796 mg, 7.51 mmol) was slowly added. The resulting mixture was flushed with argon and stirred at 85 °C for 1.5 h. Meanwhile, *N*,*N*,*N*',*N*'-tetramethylethylenediamine (21.3 mg, 0.184 mmol) and copper(I) bromide (12.4 mg, 0.086 mmol) were dissolved in water (0.35 mL) and stirred at room temperature for 1 h under argon atmosphere. Subsequently, the second solution was added to the solution containing the substrate, the resulting mixture flushed with argon and stirred at 85 °C for 18 h. After the reaction time, the solution was cooled to room temperature and slowly acidified with an aqueous 1 M HCl solution until pH 1. The products were extracted with 2-MeTHF (2 × 20 mL). The organic phase was dried over Na_2SO_4, filtered, and evaporated under reduced pressure. The resulting crude product (442 mg), containing 4,5-dihydroxyisophthalic acid (**2d**) as the major compound, was directly used for esterification with pentan-1-ol. First, approximately 25 mL of *n*-pentanol was degassed to avoid oxidation of the catechol materials by sparging with argon under ultrasonication for 15 minutes. The crude starting material (442 mg) was dissolved in degassed pentan-1-ol (22 mL) and sulfuric acid (0.025 mL, 0.446 mmol) was added. The reaction mixture was stirred for 20 h at reflux temperature (138 °C) under an argon atmosphere. By TLC analysis a full conversion was demonstrated. The mixture was concentrated under reduced pressure and then water (20 mL) was added. The obtained aqueous phase was extracted with MTBE (3 × 20 mL). The organic phases were combined and washed with water (2 × 50 mL) and brine (1 × 50 mL). The organic phase was dried over anhydrous Na_2SO_4, filtered, and then concentrated under reduced pressure. The resulting crude residue was purified by automated flash chromatography (Büchi Pure C-850 Flashprep, 40 g silica cartridge, 35 mL/min flowrate, eluent: 100% heptane to 90% heptane/10% EtOAc over 40 min) to obtain dipentyl 4,5-dihydroxyisophthalate (**6d**) as an off-white solid in 23% overall yield (211 mg, 0.622 mmol). No spectroscopic data was available in literature.

Off-white solid, R_f = 0.58 (EtOAc/heptane 30:70), m.p. 47.0 °C. ^{1}H NMR (400 MHz, $CDCl_3$) δ_H: 11.47 (br s, 1H), 8.14 (d, *J* = 2.0 Hz, 1H), 7.75 (d, *J* = 2.0 Hz, 1H), 5.70 (br s, 1H), 4.38 (t, *J* = 6.7 Hz, 2H), 4.30 (t, *J* = 6.7 Hz, 2H), 1.85 – 1.72 (m, 4H), 1.47 – 1.35 (m, 8H), 0.97 – 0.91 (m, 6H) ppm. ^{13}C{^{1}H} NMR (101 MHz, $CDCl_3$) δ_C: 170.1 (C), 165.7 (C), 152.6 (C), 144.9 (C), 123.2 (CH), 121.9 (C), 120.0 (CH), 112.3 (C), 66.2 (CH_2), 65.3 (CH_2), 28.4 (CH_2), 28.2 (CH_2), 28.2 (CH_2), 28.1 (CH_2), 22.4 (CH_2), 22.3 (CH_2), 14.0 (CH_3), 13.9 (CH_3) ppm.

2-Ethylhexyl 3,4-dihydroxybenzoate (9b) [DDV-AG-009]

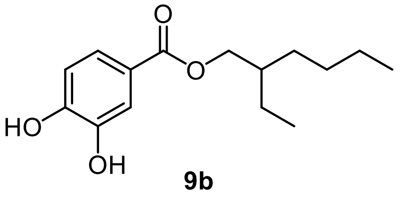

9b

The reaction was performed according to a modified literature procedure.[15] First, approximately 15 mL of *p*-xylene was degassed to avoid oxidation of the catechol materials by sparging with argon under ultrasonication for 15 minutes. 2-Ethylhexan-

1-ol (0.82 g, 6.30 mmol, 1.05 equiv.), 3,4-dihydroxybenzoic acid (**2b**) (0.93 g, 6.00 mmol, 1.00 equiv.) and *p*-toluenesulfonic acid (0.23 g, 1.20 mmol, 20 mol%) were added to *p*-xylene (10 mL). The reaction was stirred at reflux temperature under an argon atmosphere for 16 hours. After the reaction time, although the presence of starting material was still detected by TLC analysis, the work-up was nevertheless started. The reaction mixture was dissolved in MTBE (20 mL). The obtained organic phase was subsequently washed with a saturated, aqueous $NaHCO_3$ solution (2 × 20 mL) and brine (1 × 20 mL), dried over anhydrous Na_2SO_4, and filtered. The mixture was concentrated under reduced pressure and purified by automated flash chromatography (Büchi Pure C-850 Flashprep, 80 g silica cartridge, 40 mL/min flowrate, eluent: 100% heptane to 80% heptane/20% EtOAc over 50 min, 80% heptane/20% EtOAc isocratic over 20 min) to obtain 2-ethylhexyl 3,4-dihydroxybenzoate (**9b**) as a white solid in 74% yield (1.18 g, 4.42 mmol). Spectroscopic data is in accordance with literature.[29]

White solid, R_f = 0.37 (EtOAc/heptane 30:70), m.p. 76.3 °C. ^{1}H NMR (400 MHz, $CDCl_3$) δ_H: 7.71 (d, *J* = 2.0 Hz, 1H), 7.55 (dd, *J* = 8.3, 2.0 Hz, 1H), 6.92 (d, *J* = 8.3, 1H), 6.59 (br s, 2H), 4.26 – 4.17 (m, 2H), 1.74 – 1.64 (m, 1H), 1.49 – 1.26 (m, 8H), 0.95 – 0.86 (m, 6H) ppm. ^{13}C{^{1}H} NMR (101 MHz, $CDCl_3$) δ_C: 167.7 (C), 149.1 (C), 143.3 (C), 123.7 (CH), 122.6 (C), 116.8 (CH), 114.9 (CH), 67.7 (CH_2), 39.0 (CH), 30.6 (CH_2), 29.0 (CH_2), 24.0 (CH_2), 23.0 (CH_2), 14.0 (CH_3), 11.1 (CH_3) ppm.

Bis(2-ethylhexyl) 2,3-dihydroxybenzene-1,4-dicarboxylate (9c) [DDV-AG-018]

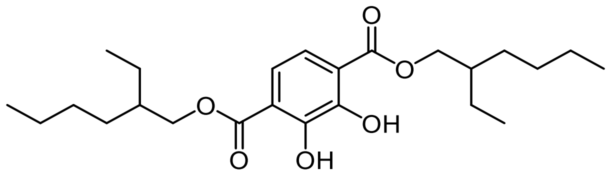

9c

The reaction was performed according to a modified literature procedure.[15] First, approximately 15 mL of *p*-xylene was degassed to avoid oxidation of the catechol materials by sparging with argon under ultrasonication for 15 minutes. 2-Ethylhexan-1-ol (2.60 g, 20.00 mmol, 4.00 equiv.), 2,3-dihydroxybenzene-1,4-dicarboxylic acid (**2c**) (0.99 g, 5.00 mmol, 1.00 equiv.) and *p*-toluenesulfonic acid (0.19 g, 1.00 mmol, 20 mol%) were added to *p*-xylene (8 mL). The reaction was stirred at reflux temperature under an argon atmosphere for 24 hours. After the reaction time, although the presence of starting material was still detected by TLC analysis, the work-up was nevertheless started. The reaction mixture was dissolved in MTBE (20 mL). The obtained organic phase was subsequently washed with distilled H_2O (2 × 20 mL) and brine (1 × 20 mL), dried over anhydrous Na_2SO_4, and filtered. The mixture was concentrated under reduced pressure and purified by automated flash chromatography (Büchi Pure C-850 Flashprep, 80 g silica cartridge, 40 mL/min flowrate, eluent: 100% heptane to 80% heptane/20% EtOAc over 50 min) to obtain bis(2-ethylhexyl) 2,3-dihydroxybenzene-1,4-dicarboxylate (**9c**) as a yellow solid in 84% yield (1.77 g, 4.18 mmol). No spectroscopic data was available in literature.

Yellow solid, R_f = 0.83 (EtOAc/heptane 30:70), m.p. 39.5 °C. ^{1}H NMR (400 MHz, $CDCl_3$) δ_H: 10.99 (s, 2H), 7.32 (s, 2H), 4.34 – 4.26 (m, 4H), 1.80 – 1.69 (m, 2H), 1.50

– 1.29 (m, 16 H), 0.95 (t, *J* = 7.5 Hz, 6H), 0.93 – 0.89 (m, 6H) ppm. $^{13}C\{^1H\}$ NMR (101 MHz, $CDCl_3$) δ_C: 169.9 (C), 151.7 (C), 118.2 (CH), 116.2 (C), 68.3 (CH_2), 38.9 (CH), 30.5 (CH_2), 29.0 (CH_2), 24.0 (CH_2), 23.0 (CH_2), 14.0 (CH_3), 11.1 (CH_3) ppm. HRMS (ESI) for $C_{24}H_{38}O_6Na$ $[M+Na]^+$, calcd 445.2561, found 445.2574.

Bis(2-ethylhexyl) 4,5-dihydroxyisophthalate (9d) [AGO-136-137-138]

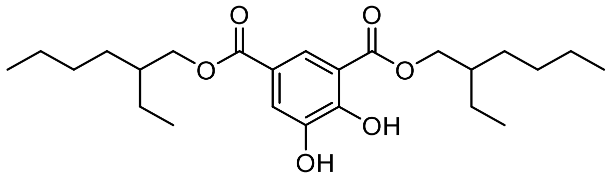

9d

Modified literature procedures were followed.[15, 28] 5-Hydroxyisophthalic acid (1.00 g, 5.49 mmol) and concentrated sulfuric acid (12 mL) were added together and stirred at 0 °C. Subsequently, *N*-bromosuccinimide (1.026 g, 5.77 mmol) was slowly added under vigorous stirring. The resulting reaction mixture was heated to 50 °C and stirred for 2 h. After the reaction time, the solution was cooled to 0 °C and water (40 mL) was added. The aqueous phase was extracted with EtOAc (2 × 40 mL). The organic phases were combined, dried over anhydrous Na_2SO_4, filtered, and evaporated under reduced pressure. The resulting crude product (1.60 g), containing 4-bromo-5-hydroxyisophthalic acid as the major compound, was then dissolved in water (6 mL) and sodium carbonate (1.754 g, 16.55 mmol) was slowly added. The resulting mixture was flushed with argon and stirred at 85 °C for 1.5 h. Meanwhile, *N*,*N*,*N'*,*N'*-tetramethylethylenediamine (47.0 mg, 0.405 mmol) and copper(I) bromide (27.0 mg, 0.190 mmol) were dissolved in water (0.75 mL) and stirred at room temperature for 1 h under argon atmosphere. Subsequently, the second solution was added to the solution containing the substrate, the resulting mixture flushed with argon and stirred at 85 °C for 18 h. After the reaction time, the solution was cooled to room temperature and slowly acidified with an aqueous 1 M HCl solution until pH 1. The products were extracted with 2-MeTHF (2 × 40 mL). The organic phase was dried over Na_2SO_4, filtered, and evaporated under reduced pressure. The resulting crude product (881 mg), containing 4,5-dihydroxyisophthalic acid (**2d**) as the major compound, was directly used for the esterification with 2-ethylhexan-1-ol. First, approximately 15 mL of *p*-xylene was degassed to avoid oxidation of the catechol materials by sparging with argon under ultrasonication for 15 minutes. The crude starting material (881 mg), 2-ethylhexan-1-ol (3.48 g, 26.7 mmol), and *p*-toluenesulfonic acid (0.153 g, 0.890 mmol) were added to *p*-xylene (7.2 mL). The reaction was stirred at reflux temperature under an argon atmosphere for 24 h. After the reaction time, the reaction mixture was dissolved in MTBE (40 mL). The obtained organic phase was subsequently washed with distilled H_2O (2 × 40 mL) and brine (1 × 40 mL), dried over anhydrous Na_2SO_4, filtered, and concentrated under reduced pressure. The resulting crude was purified by automated flash chromatography (Büchi Pure C-850 Flashprep, 80 g silica cartridge, 40 mL/min flowrate, eluent: 100% heptane to 90% heptane/10% EtOAc over 50 min) to obtain bis(2-ethylhexyl) 4,5-dihydroxyisophthalate (**9d**) as a brown oil in 22% overall yield (499 mg, 1.181 mmol). No spectroscopic data was available in literature.

Brown oil, R_f = 0.66 (EtOAc/heptane 30:70). ^{1}H NMR (400 MHz, $CDCl_3$) δ_H: 8.13 (d, *J* = 2.0 Hz, 1H), 7.75 (d, *J* = 2.0 Hz, 1H), 4.36 – 4.17 (m, 4H), 1.78 – 1.66 (m, 2H), 1.52 – 1.29 (m, 16H), 0.99 – 0.87 (m, 12H) ppm. $^{13}C\{^1H\}$ NMR (101 MHz, $CDCl_3$) δ_C: 170.2 (C), 165.7 (C), 152.6 (C), 145.0 (C), 123.1 (CH), 122.0 (C), 120.1 (CH), 112.4 (C), 68.3 (CH_2), 67.4 (CH_2), 39.0 (CH), 38.9 (CH), 30.6 (CH_2), 30.5 (CH_2), 29.1 (CH_2), 29.0 (CH_2), 24.1 (CH_2), 23.9 (CH_2), 23.0 (CH_2), 22.9 (CH_2), 14.1 (CH_3), 14.0 (CH_3), 11.1 (CH_3), 11.1 (CH_3) ppm.

12.4 Experimental protocols and characterization of plasticizer candidates 8b-8d and 11b-11d

Pentyl 3,4-bis[(4-oxopentanoyl)oxy]benzoate (8b) [DDV-AG-014]

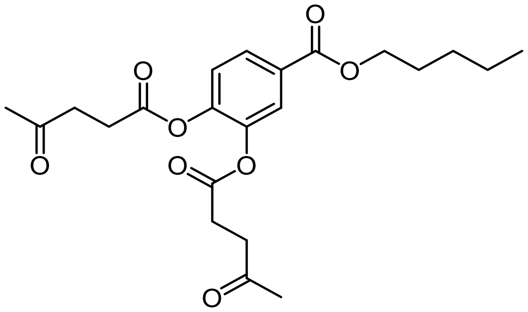

8b

The *title compound* **8b** was synthesized using pentyl 3,4-dihydroxybenzoate (**6b**) (200 mg, 0.89 mmol, 1.00 equiv.), levulinic acid (311 mg, 2.68 mmol, 3.00 equiv.), EDC•HCl (513 mg, 2.68 mmol, 3.00 equiv.), DMAP (32.7 mg, 0.27 mmol, 30 mol%) and THF (7 mL), following General procedure C. The resulting crude residue was purified by automated flash chromatography (Büchi Pure C-850 Flashprep, 12 g silica cartridge, 30 mL/min flowrate, eluent: 100% heptane to 70% heptane/30% EtOAc over 50 min, 70% heptane/30% EtOAc isocratic over 20 min) to obtain pentyl 3,4-bis[(4-oxopentanoyl)oxy]benzoate (**8b**) as a colorless oil in 78% yield (293 mg, 0.70 mmol). No spectroscopic data was available in literature.

Colorless oil, R_f = 0.08 (EtOAc/heptane 30:70). ^{1}H NMR (400 MHz, $CDCl_3$) δ_H: 7.93 (dd, *J* = 8.5, 2.0 Hz, 1H), 7.83 (d, *J* = 2.0 Hz, 1H), 7.25 (d, *J* = 8.5 Hz, 1H), 4.30 (t, *J* = 6.7 Hz, 2H), 2.92 – 2.84 (m, 8H), 2.21 (s, 6H), 1.75 (quint, *J* = 6.9 Hz, 2H), 1.43 – 1.35 (m, 4H), 0.95 – 0.90 (m, 3H) ppm. $^{13}C\{^1H\}$ NMR (101 MHz, $CDCl_3$) δ_C: 206.1 (C), 206.1 (C), 170.3 (C), 170.1 (C), 165.1 (C), 146.1 (C), 142.1 (C), 129.2 (C), 128.1 (CH), 124.9 (CH), 123.5 (CH), 65.5 (CH_2), 37.7 (CH_2), 37.7 (CH_2), 29.8 (CH_3), 29.8 (CH_3), 28.4 (CH_2), 28.1 (CH_2), 27.8 (CH_2), 27.7 (CH_2), 22.3 (CH_2), 14.0 (CH_3) ppm. HRMS (ESI) for $C_{22}H_{28}O_8Na$ $[M+Na]^+$, calcd 443.1676, found 443.1692.

Dipentyl 2,3-bis[(4-oxopentanoyl)oxy]benzene-1,4-dicarboxylate (8c) [DDV-AG-022]

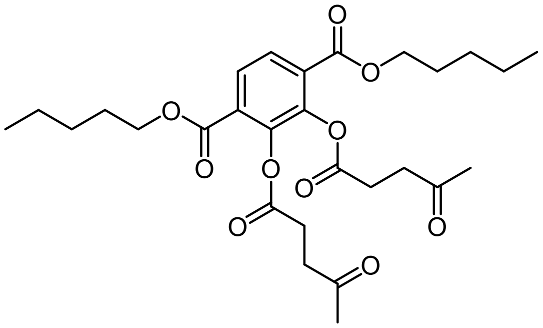

8c

The *title compound* **8c** was synthesized using dipentyl 2,3-dihydroxybenzene-1,4-dicarboxylate (**6c**) (338 mg, 1.00 mmol, 1.00 equiv.), levulinic acid (348 mg, 3.00 mmol, 3.00 equiv.), EDC•HCl (575 mg, 3.00 mmol, 3.00 equiv.), DMAP (37.0 mg, 0.30 mmol, 30 mol%) and CH_2Cl_2 (7.5 mL), following General procedure C. The resulting crude residue was purified by automated flash chromatography (Büchi Pure C-850 Flashprep, 40 g silica cartridge, 40 mL/min flowrate, eluent: 100% heptane to 60% heptane/40% EtOAc over 50 min) to obtain dipentyl 2,3-bis[(4-oxopentanoyl)oxy]benzene-1,4-dicarboxylate (**8c**) as a colorless oil in 93% yield (498 mg, 0.93 mmol). No spectroscopic data was available in literature.

Colorless oil, R_f = 0.16 (EtOAc/heptane 30:70). ^{1}H NMR (400 MHz, $CDCl_3$) δ_H: 7.89 (s, 2H), 4.28 (t, *J* = 6.8 Hz, 4H), 3.01 (t, *J* = 6.2 Hz, 4H), 2.90 (t, *J* = 6.3 Hz, 4H), 2.21 (s, 6H), 1.78 – 1.69 (m, 4H), 1.42 – 1.34 (m, 8H), 0.95 – 0.90 (m, 6H) ppm. $^{13}C\{^1H\}$ NMR (101 MHz, $CDCl_3$) δ_C: 206.1 (C), 170.5 (C), 163.3 (C), 144.1 (C), 128.4 (C), 128.0 (CH), 65.9 (CH_2), 37.7 (CH_2), 29.8 (CH_3), 28.3 (CH_2), 28.1 (CH_2), 27.8 (CH_2), 22.3 (CH_2), 14.0 (CH_3) ppm. HRMS (ESI) for $C_{28}H_{38}O_{10}Na$ $[M+Na]^+$, calcd 557.2357, found 557.2364.

Dipentyl 4,5-bis((4-oxopentanoyl)oxy)isophthalate (8d) [AGO-135]

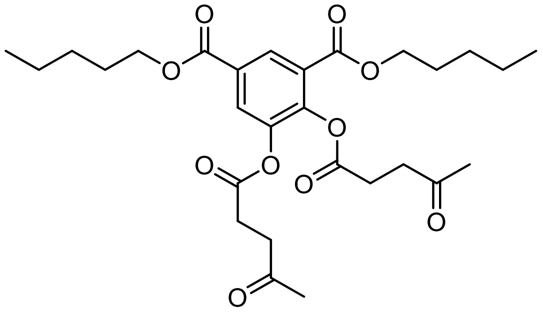

8d

The *title compound* **8d** was synthesized using dipentyl 4,5-dihydroxyisophthalate (**6d**) (211 mg, 0.624 mmol, 1.00 equiv.), levulinic acid (217 mg, 1.87 mmol, 3.00 equiv.), EDC•HCl (359 mg, 1.87 mmol, 3.00 equiv.), DMAP (22.9 mg, 0.187 mmol, 30 mol%) and CH_2Cl_2 (4.7 mL), following General procedure C. The resulting crude residue was purified by automated flash chromatography (Büchi Pure C-850 Flashprep, 40 g silica cartridge, 40 mL/min flowrate, eluent: 100% heptane to 60% heptane/40% EtOAc over 50 min) to obtain dipentyl 4,5-bis((4-oxopentanoyl)oxy)isophthalate (**8d**) as a colorless

oil in 69% yield (228 mg, 0.427 mmol). No spectroscopic data was available in literature.

Colorless oil, R_f = 0.18 (EtOAc/heptane 30:70). ^{1}H NMR (400 MHz, $CDCl_3$) δ_H: 8.52 (d, *J* = 2.1 Hz, 1H), 7.99 (d, *J* = 2.1 Hz, 1H), 4.33 (t, *J* = 6.7 Hz, 2H), 4.28 (t, *J* = 6.8 Hz, 2H), 2.99 – 2.94 (m, 4H), 2.92 – 2.86 (m, 4H), 2.22 (s, 3H), 2.21 (s, 3H), 1.82 – 1.70 (m, 4H), 1.43 – 1.34 (m, 8H), 0.96 – 0.89 (m, 6H) ppm. ^{13}C{^{1}H} NMR (101 MHz, $CDCl_3$) δ_C: 206.3 (C), 206.2 (C), 170.5 (C), 170.2 (C), 164.6 (C), 163.4 (C), 146.5 (C), 143.9 (C), 130.2 (CH), 128.9 (C), 128.6 (CH), 125.4 (C), 66.0 (CH_2), 65.9 (CH_2), 37.9 (CH_2), 37.8 (CH_2), 29.9 (CH_3), 29.9 (CH_3), 28.5 (CH_2), 28.4 (CH_2), 28.2 (CH_2), 28.2 (CH_2), 27.9 (CH_2), 27.8 (CH_2), 22.4 (CH_2), 22.4 (CH_2), 14.1 (CH_3), 14.0 (CH_3) ppm. HRMS (ESI) for $C_{28}H_{38}O_{10}Na$ $[M+Na]^+$, calcd 557.2357, found 557.2357.

2-Ethylhexyl 3,4-bis[(4-oxopentanoyl)oxy]benzoate (11b) [DDV-AG-021]

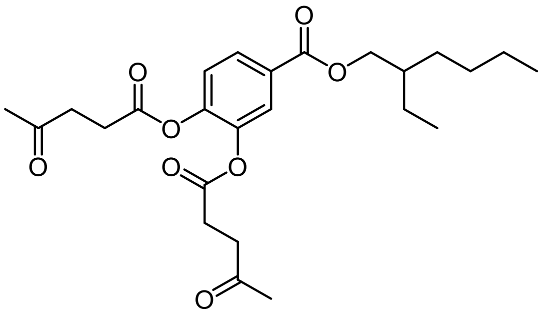

11b

The *title compound* **11b** was synthesized using 2-ethylhexyl 3,4-dihydroxybenzoate (**9b**) (266 mg, 1.00 mmol, 1.00 equiv.), levulinic acid (348 mg, 3.00 mmol, 3.00 equiv.), EDC•HCl (575 mg, 3.00 mmol, 3.00 equiv.), DMAP (37.0 mg, 0.30 mmol, 30 mol%) and CH_2Cl_2 (7.5 mL), following General procedure C. The resulting crude residue was purified by automated flash chromatography (Büchi Pure C-850 Flashprep, 40 g silica cartridge, 40 mL/min flowrate, eluent: 100% heptane to 60% heptane/40% EtOAc over 50 min) to obtain 2-ethylhexyl 3,4-bis[(4-oxopentanoyl)oxy]benzoate (**11b**) as a yellow oil in 81% yield (374 mg, 0.81 mmol). No spectroscopic data was available in literature.

Yellow oil, R_f = 0.09 (EtOAc/heptane 30:70). ^{1}H NMR (400 MHz, $CDCl_3$) δ_H: 7.92 (dd, *J* = 8.5, 2.0 Hz, 1H), 7.82 (d, *J* = 2.0 Hz, 1H), 7.25 (d, *J* = 8.5 Hz, 1H), 4.27 – 4.18 (m, 2H), 2.93 – 2.84 (m, 8H), 2.21 (s, 6H), 1.75 – 1.65 (m, 1H), 1.48 – 1.29 (m, 8H), 0.96 – 0.88 (m, 6H) ppm. ^{13}C{^{1}H} NMR (101 MHz, $CDCl_3$) δ_C: 206.1 (C), 206.1 (C), 170.3 (C), 170.1 (C), 165.2 (C), 146.1 (C), 142.2 (C), 129.2 (C), 128.1 (CH), 124.9 (CH), 123.5 (CH), 67.7 (CH_2), 38.9 (CH), 37.7 (CH_2), 37.7 (CH_2), 30.6 (CH_2), 29.8 (CH_3), 29.8 (CH_3), 29.0 (CH_2), 27.8 (CH_2), 27.7 (CH_2), 24.0 (CH_2), 23.0 (CH_2), 14.0 (CH_3), 11.1 (CH_3) ppm. HRMS (ESI) for $C_{25}H_{34}O_8Na$ $[M+Na]^+$, calcd 485.2146, found 485.2149.

Bis(2-ethylhexyl) 2,3-bis[(4-oxopentanoyl)oxy]benzene-1,4-dicarboxylate (11c) [DDV-AG-023]

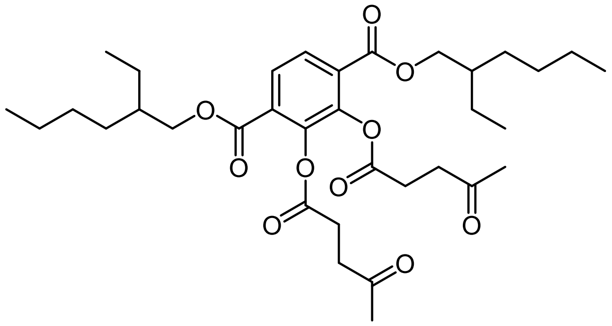

11c

The *title compound* **11c** was synthesized using bis(2-ethylhexyl) 2,3-dihydroxybenzene-1,4-dicarboxylate (**9c**) (338 mg, 0.80 mmol, 1.00 equiv.), levulinic acid (279 mg, 2.40 mmol, 3.00 equiv.), EDC•HCl (460 mg, 2.40 mmol, 3.00 equiv.), DMAP (29.0 mg, 0.24 mmol, 30 mol%) and CH_2Cl_2 (7.5 mL), following General procedure C. The resulting crude residue was purified by automated flash chromatography (Büchi Pure C-850 Flashprep, 40 g silica cartridge, 40 mL/min flowrate, eluent: 100% heptane to 70% heptane/30% EtOAc over 50 min) to obtain bis(2-ethylhexyl) 2,3-bis[(4-oxopentanoyl)oxy]benzene-1,4-dicarboxylate (**11c**) as a colorless oil in 77% yield (379 mg, 0.61 mmol). No spectroscopic data was available in literature.

Colorless oil, R_f = 0.26 (EtOAc/heptane 30:70). ^{1}H NMR (400 MHz, $CDCl_3$) δ_H: 7.87 (s, 2H), 4.24 – 4.15 (m, 4H), 3.01 (t, *J* = 6.3 Hz, 4H), 2.91 (t, *J* = 6.5 Hz, 4H), 2.21 (s, 6H), 1.72 – 1.65 (m, 2H), 1.46 – 1.28 (m, 16H), 0.95 – 0.89 (m, 12H) ppm. ^{13}C{^{1}H} NMR (101 MHz, $CDCl_3$) δ_C: 206.1 (C), 170.5 (C), 163.2 (C), 144.2 (C), 128.3 (C), 127.8 (CH), 68.1 (CH_2), 38.8 (CH), 37.8 (CH_2), 30.4 (CH_2), 29.8 (CH_3), 28.9 (CH_2), 27.8 (CH_2), 23.8 (CH_2), 23.0 (CH_2), 14.0 (CH_3), 11.0 (CH_3) ppm. HRMS (ESI) for $C_{34}H_{50}O_{10}Na$ [M+Na]$^+$, calcd 641.3296, found 641.3314.

Bis(2-ethylhexyl) 4,5-bis[(4-oxopentanoyl)oxy]isophthalate (11d) [AGO-139]

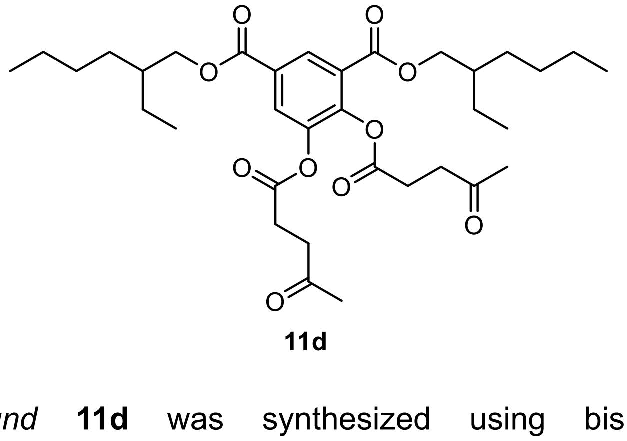

11d

The *title compound* **11d** was synthesized using bis(2-ethylhexyl) 4,5-dihydroxyisophthalate (**9d**) (400 mg, 0.947 mmol, 1.00 equiv.), levulinic acid (330 mg, 2.84 mmol, 3.00 equiv.), EDC•HCl (544 mg, 2.84 mmol, 3.00 equiv.), DMAP (34.7 mg, 0.284 mmol, 30 mol%) and CH_2Cl_2 (8.8 mL), following General procedure C. The resulting crude residue was purified by automated flash chromatography (Büchi Pure

C-850 Flashprep, 40 g silica cartridge, 35 mL/min flowrate, eluent: 100% heptane to 65% heptane/35% EtOAc over 50 min) to obtain bis(2-ethylhexyl) 4,5-bis[(4-oxopentanoyl)oxy]isophthalate (**11d**) as a colorless oil in 78% yield (455 mg, 0.735 mmol). No spectroscopic data was available in literature.

Colorless oil, R_f = 0.28 (EtOAc/heptane 30:70). ^{1}H NMR (400 MHz, $CDCl_3$) δ_H: 8.51 (d, *J* = 2.1 Hz, 1H), 7.99 (d, *J* = 2.1 Hz, 1H), 4.31 – 4.15 (m, 4H), 3.01 – 2.95 (m, 4H), 2.93 – 2.87 (m, 4H), 2.22 (s, 3H), 2.21 (s, 3H), 1.76 – 1.65 (m, 2H), 1.49 – 1.27 (m, 16 H), 0.97 – 0.87 (m, 12H) ppm. ^{13}C{^{1}H} NMR (101 MHz, $CDCl_3$) δ_C: 206.3 (C), 206.2 (C), 170.6 (C), 170.3 (C), 164.6 (C), 163.2 (C), 146.7 (C), 144.0 (C), 130.0 (CH), 128.8 (C), 128.6 (CH), 125.2 (C), 68.0 (CH_2), 68.0 (CH_2), 39.0 (CH), 38.9 (CH), 37.8 (CH_2), 37.8 (CH_2), 30.6 (CH_2), 30.5 (CH_2), 29.9 (CH_3), 29.9 (CH_3), 29.1 (CH_2), 29.0 (CH_2), 27.9 (CH_2), 27.8 (CH_2), 24.0 (CH_2), 23.9 (CH_2), 23.1* (CH_2), 14.1* (CH_3), 11.2 (CH_3), 11.1 (CH_3) ppm. *Signal is assumably the result of two overlapping signals. HRMS (ESI) for $C_{34}H_{50}O_{10}Na$ [M+Na]$^+$, calcd 641.3296, found 641.3309.

12.5 Experimental protocols and characterization of plasticizer candidates 12a-12d

Pentyl 2,3-bis(pentanoyloxy)benzoate (12a) [DDV-AG-036]

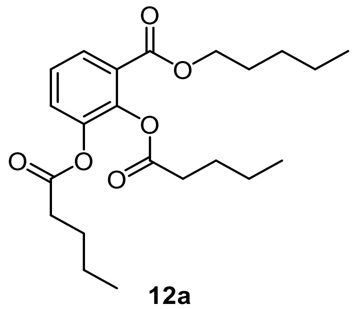

12a

The *title compound* **12a** was synthesized using pentyl 2,3-dihydroxybenzoate (**6a**) (250 mg, 1.12 mmol, 1.00 equiv.), triethylamine (338 mg, 3.34 mmol, 3.00 equiv.), pentanoyl chloride (0.40 mL, 3.34 mmol, 3.00 equiv.) and CH_2Cl_2 (1.25 mL), following General procedure B. The resulting crude residue was purified by automated flash chromatography (Büchi Pure C-850 Flashprep, 40 g silica cartridge, 40 mL/min flowrate, eluent: 100% heptane to 85% heptane/15% EtOAc over 50 min) to obtain pentyl 2,3-bis(pentanoyloxy)benzoate (**12a**) as a colorless oil in 90% yield (394 mg, 1.00 mmol). No spectroscopic data was available in literature.

Colorless oil, R_f = 0.74 (EtOAc/heptane 30:70). ^{1}H NMR (400 MHz, $CDCl_3$) δ_H: 7.86 (dd, *J* = 7.8, 1.8 Hz, 1H), 7.35 (dd, *J* = 8.1, 1.8 Hz, 1H), 7.29 (t, *J* = 7.9 Hz, 1H), 4.25 (t, *J* = 6.8 Hz, 2H), 2.64 – 2.58 (m, 2H), 2.57 – 2.53 (m, 2H), 1.77 – 1.68 (m, 6H), 1.50 – 1.34 (m, 8H), 1.00 – 0.94 (m, 6H), 0.94 – 0.90 (m, 3H) ppm. ^{13}C{^{1}H} NMR (101 MHz, $CDCl_3$) δ_C: 171.1 (C), 171.1 (C), 164.0 (C), 143.6 (C), 142.8 (C), 128.7 (CH), 127.6 (CH), 125.9 (CH), 125.4 (C), 65.5 (CH_2), 33.8 (CH_2), 33.7 (CH_2), 28.4 (CH_2), 28.1 (CH_2), 27.0 (CH_2), 26.7 (CH_2), 22.4 (CH_2), 22.3 (CH_2), 22.3 (CH_2), 13.9 (CH_3), 13.8 (CH_3), 13.7 (CH_3) ppm. HRMS (ESI) for $C_{22}H_{32}O_6Na$ [M+Na]$^+$, calcd 415.2091, found 415.2102.

Pentyl 3,4-bis(pentanoyloxy)benzoate (12b) [DDV-AG-038]

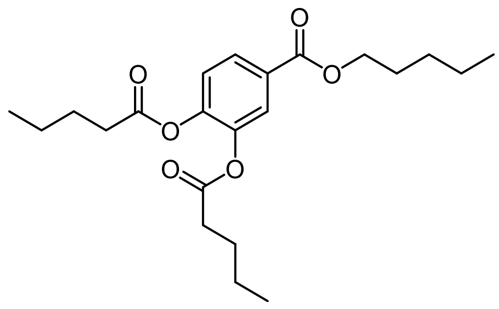

12b

The *title compound* **12b** was synthesized using pentyl 3,4-dihydroxybenzoate (**6b**) (180 mg, 0.80 mmol, 1.00 equiv.), triethylamine (244 mg, 2.41 mmol, 3.00 equiv.), pentanoyl chloride (0.29 mL, 2.41 mmol, 3.00 equiv.) and THF (2.2 mL), following General procedure B. The resulting crude residue was purified by automated flash chromatography (Büchi Pure C-850 Flashprep, 12 g silica cartridge, 30 mL/min flowrate, eluent: 100% heptane to 90% heptane/10% EtOAc over 50 min) to obtain pentyl 3,4-bis(pentanoyloxy)benzoate (**12b**) as a colorless oil in 91% yield (287 mg, 0.73 mmol). No spectroscopic data was available in literature.

Colorless oil, R_f = 0.72 (EtOAc/heptane 30:70). ^{1}H NMR (400 MHz, $CDCl_3$) δ_H: 7.94 (dd, *J* = 8.5, 2.0 Hz, 1H), 7.84 (d, *J* = 2.0 Hz, 1H), 7.26 (d, *J* = 8.5 Hz, 1H), 4.30 (t, *J* = 6.7 Hz, 2H), 2.58 – 2.52 (m, 4H), 1.79 – 1.68 (m, 6H), 1.50 – 1.35 (m, 8H), 0.99 – 0.90 (m, 9H) ppm. ^{13}C{^{1}H} NMR (101 MHz, $CDCl_3$) δ_C: 170.9 (C), 170.6 (C), 165.2 (C), 146.1 (C), 142.1 (C), 129.0 (C), 127.9 (CH), 125.0 (CH), 123.5 (CH), 65.5 (CH_2), 33.8 (CH_2), 33.7 (CH_2), 28.4 (CH_2), 28.2 (CH_2), 27.0 (CH_2), 26.9 (CH_2), 22.4 (CH_2), 22.3 (CH_2), 22.2 (CH_2), 14.0 (CH_3), 13.7 (CH_3), 13.7 (CH_3) ppm. HRMS (ESI) for $C_{22}H_{32}O_6Na$ [M+Na]$^+$, calcd 415.2091, found 415.2100.

Dipentyl 2,3-bis(pentanoyloxy)benzene-1,4-dicarboxylate (12c) [DDV-AG-037]

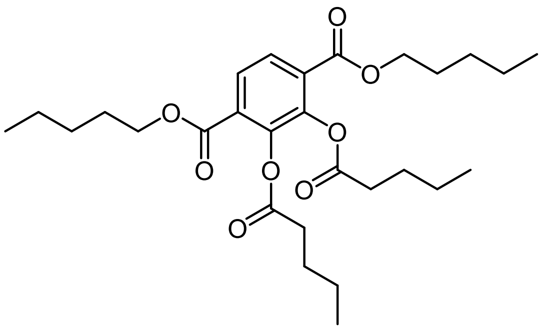

12c

The *title compound* **12c** was synthesized using dipentyl 2,3-dihydroxybenzene-1,4-dicarboxylate (**6c**) (250 mg, 0.74 mmol, 1.00 equiv.), triethylamine (224 mg, 2.22 mmol, 3.00 equiv.), pentanoyl chloride (0.26 mL, 2.22 mmol, 3.00 equiv.) and CH_2Cl_2 (0.8 mL), following General procedure B. The resulting crude residue was purified by automated flash chromatography (Büchi Pure C-850 Flashprep, 12 g silica cartridge, 30 mL/min flowrate, eluent: 100% heptane to 90% heptane/10% EtOAc over 50 min) to obtain dipentyl 2,3-bis(pentanoyloxy)benzene-1,4-dicarboxylate (**12c**) as a

colorless oil in 96% yield (360 mg, 0.71 mmol). No spectroscopic data was available in literature.

Colorless oil, R_f = 0.74 (EtOAc/heptane 30:70). ^{1}H NMR (400 MHz, $CDCl_3$) δ_H: 7.89 (s, 2H), 4.27 (t, *J* = 6.8 Hz, 4H), 2.61 (t, *J* = 7.6 Hz, 4H), 1.79 – 1.69 (m, 8H), 1.50 – 1.34 (m, 12H), 0.97 (t, *J* = 7.4 Hz, 6H), 0.95 – 0.90 (m, 6H) ppm. ^{13}C{^{1}H} NMR (101 MHz, $CDCl_3$) δ_C: 171.0 (C), 163.4 (C), 144.0 (C), 128.5 (C), 127.8 (CH), 65.8 (CH_2), 33.7 (CH_2), 28.3 (CH_2), 28.1 (CH_2), 26.7 (CH_2), 22.3 (CH_2), 22.3 (CH_2), 13.9 (CH_3), 13.7 (CH_3) ppm. HRMS (ESI) for $C_{28}H_{42}O_8Na$ [M+Na]$^+$, calcd 529.2772, found 529.2776.

Dipentyl 4,5-bis(pentanoyloxy)isophthalate (12d) [AGO-131]

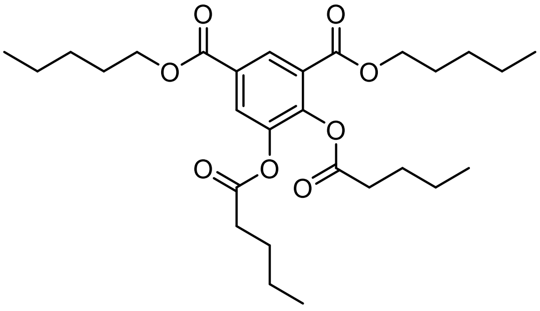

12d

The *title compound* **12d** was synthesized using dipentyl 4,5-dihydroxyisophthalate (**6d**) (180 mg, 0.532 mmol, 1.00 equiv.), triethylamine (161 mg, 1.60 mmol, 3.00 equiv.), pentanoyl chloride (0.19 mL, 1.60 mmol, 3.00 equiv.) and CH_2Cl_2 (0.6 mL), following General procedure B. The resulting crude residue was purified by automated flash chromatography (Büchi Pure C-850 Flashprep, 40 g silica cartridge, 35 mL/min flowrate, eluent: 100% heptane to 90% heptane/10% EtOAc over 50 min) to obtain dipentyl 4,5-bis(pentanoyloxy)isophthalate (**12d**) as a colorless oil in 90% yield (242 mg, 0.478 mmol). No spectroscopic data was available in literature.

Colorless oil, R_f = 0.73 (EtOAc/heptane 30:70). ^{1}H NMR (400 MHz, $CDCl_3$) δ_H: 8.52 (d, *J* = 2.1 Hz, 1H), 7.99 (d, *J* = 2.0 Hz, 1H), 4.33 (t, *J* = 6.7 Hz, 2H), 4.28 (t, *J* = 6.8 Hz, 2H), 2.62 (t, *J* = 7.6 Hz, 2H), 2.57 (t, *J* = 7.5 Hz, 2H), 1.81 – 1.69 (m, 8H), 1.50 – 1.36 (m, 12H), 1.00 – 0.89 (m, 12H) ppm. ^{13}C{^{1}H} NMR (101 MHz, $CDCl_3$) δ_C: 171.0 (C), 170.8 (C), 164.7 (C), 163.5 (C), 146.5 (C), 143.8 (C), 130.1 (CH), 128.7 (C), 128.5 (CH), 125.7 (C), 65.9 (CH_2), 65.9 (CH_2), 33.8 (CH_2), 33.8 (CH_2), 28.5 (CH_2), 28.4 (CH_2), 28.2 (CH_2), 28.2 (CH_2), 27.1 (CH_2), 26.7 (CH_2), 22.5* (CH_2), 22.4 (CH_2), 22.4 (CH_2), 14.1 (CH_3), 14.1 (CH_3), 13.8 (CH_3), 13.8 (CH_3) ppm. *Signal is assumably the result of two overlapping signals. HRMS (ESI) for $C_{28}H_{42}O_8Na$ [M+Na]$^+$, calcd 529.2772, found 529.2776.

12.6 Characterization of isolated byproducts

Pentyl 2-hydroxy-3-((4-oxopentanoyl)oxy)benzoate (8e) [AGO-076]

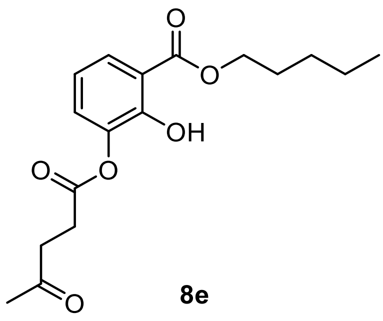

8e

The *title compound* **8e** was isolated as a byproduct from the crude reaction mixture in Table S14, entry 11.

Colorless oil, R_f = 0.43 (EtOAc/heptane 30:70). ^{1}H NMR (400 MHz, $CDCl_3$) δ_H: 10.98 (s, 1H), 7.73 (dd, *J* = 8.1, 1.6 Hz, 1H), 7.25 (dd, *J* = 8.0, 1.5 Hz, 1H), 6.86 (t, *J* = 8.0 Hz, 1H), 4.34 (t, *J* = 6.7 Hz, 2H), 2.93 – 2.86 (m, 4H), 2.22 (s, 3H), 1.82 – 1.73 (m, 2H), 1.46 – 1.35 (m, 4H), 0.93 (t, *J* = 7.1 Hz, 3H) ppm. ^{13}C{^{1}H} NMR (101 MHz, $CDCl_3$) δ_C: 206.2 (C), 170.8 (C), 170.0 (C), 153.6 (C), 139.2 (C), 128.6 (CH), 127.3 (CH), 118.5 (CH), 114.2 (C), 65.9 (CH_2), 38.0 (CH_2), 29.9 (CH_3), 28.2 (CH_2), 28.1 (CH_2), 27.8 (CH_2), 22.3 (CH_2), 13.9 (CH_3) ppm. TLC-MS (ESI) for $C_{17}H_{22}O_6$ $[M+Na]^+$ calcd 345.13, found 345.58.

Pentyl 2-hydroxy-3-((2-methyl-5-oxotetrahydrofuran-2-yl)oxy)benzoate (8f) [AGO-076]

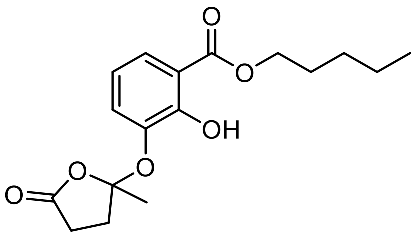

8f

The *title compound* **8f** was isolated as a byproduct from the crude reaction mixture in Table S14, entry 11.

Colorless oil, R_f = 0.51 (EtOAc/heptane 30:70). ^{1}H NMR (400 MHz, $CDCl_3$) δ_H: 11.05 (d, *J* = 0.5 Hz, 1H), 7.67 (dd, *J* = 8.1, 1.6 Hz, 1H), 7.38 (ddd, *J* = 7.9, 1.6, 0.4 Hz, 1H), 6.82 (t, *J* = 8.0 Hz, 1H), 4.35 (t, *J* = 6.7 Hz, 2H), 2.83 (ddd, *J* = 17.4, 10.0, 9.4 Hz, 1H), 2.70 (ddd, *J* = 13.2, 9.3, 2.5 Hz, 1H), 2.57 (ddd, *J* = 17.4, 9.5, 2.5 Hz, 1H), 2.30 (dt, *J* = 13.2, 9.7 Hz), 1.83 – 1.74 (m, 2H), 1.67 (s, 3H), 1.47 – 1.35 (m, 4H), 0.94 (t, *J* = 7.1 Hz, 3H) ppm. ^{13}C{^{1}H} NMR (101 MHz, $CDCl_3$) δ_C: 176.1 (C), 170.3 (C), 155.6 (C), 141.4 (C), 130.5 (CH), 126.3 (CH), 118.6 (CH), 114.0 (C), 111.1 (C), 65.9 (CH_2), 34.6 (CH_2), 28.9 (CH_2), 28.2 (CH_2), 28.1 (CH_2), 23.3 (CH_3), 22.3 (CH_2), 13.9 (CH_3) ppm. TLC-MS (ESI) for $C_{17}H_{22}O_6$ $[M+Na]^+$, calcd 345.13, found 345.46.

12.7 Experimental protocols for synthesis and characterization of reactants

5-Chloro-5-methyldihydrofuran-2(3*H*)-one (13) [AGO-2024-0004]

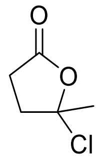

13

A round-bottom flask equipped with a magnetic stirring bar was charged with levulinic acid (2.32 g, 20.0 mmol, 1.0 equiv.) and closed with a septum pierced with a needle to avoid overpressure. Thionyl chloride (1.61 mL, 22.0 mmol, 1.1 equiv.) was slowly added over a period of 15 minutes, and the resulting mixture was stirred for 3 h. After the reaction time, the excess of thionyl chloride was removed by rotary evaporation. The water bath of the rotavapor was set at 40 °C while a pressure of 500 mbar was imposed for 5 min and eventually a pressure of 20 mbar for 10 min. Eventually, the *title compound* was obtained as a yellow liquid with 91 wt% purity in quantitative yield. Spectroscopic data is in accordance with literature.[22a]

Yellow liquid. ^{1}H NMR (400 MHz, $CDCl_3$) δ_H: 2.95 (ddd, *J* = 17.7, 11.7, 8.6 Hz, 1H), 2.74 (ddd, *J* = 13.5, 8.6, 1.1 Hz, 1H), 2.62 (ddd, *J* = 17.7, 8.6, 1.1 Hz, 1H), 2.43 (ddd, *J* = 13.5, 11.7, 8.6 Hz, 1H), 2.06 (s, 3H) ppm. ^{13}C{^{1}H} NMR (101 MHz, $CDCl_3$) δ_C: 174.5 (C), 104.2 (C), 39.9 (CH_2), 31.1 (CH_3), 28.2 (CH_2) ppm.

4,5-Dihydroxyisophthalic acid (2d) [AGO-125]

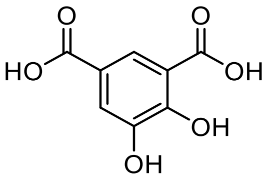

2d

Modified literature procedures were followed.[30] Dipentyl 4,5-dihydroxyisophthalate (**6d**) (518 mg, 1.53 mmol, 1.00 equiv.) was dissolved in an aqueous 2 M NaOH solution (2.30 mL). The mixture was stirred overnight at 85 °C under an argon atmosphere. The mixture was dissolved in MTBE (30 mL) and extracted with aq. NaOH (1 M, 3 × 20 mL). The combined aqueous phases were acidified using conc. HCl until pH = 1 and subsequently extracted with freshly distilled 2-MeTHF (3 × 20 mL). The resulting organic phase was subsequently dried over Na_2SO_4, filtered, and concentrated under reduced pressure. Next, *n*-pentane was added, and the mixture was ultrasonicated and decanted, twice. The resulting product was dried under reduced pressure, obtaining 4,5-dihydroxyisophthalic acid (**2d**) as a brown solid in 70% yield (254 mg, 83 wt%, 1.06 mmol). Spectroscopic data are reported in DMSO-d_6/methanol-d_4.[31]

Brown solid, m.p. 290 °C (decomp.). ^{1}H NMR (400 MHz, DMSO-d_6) δ_H: 7.89 (d, *J* = 2.1 Hz, 1H), 7.53 (d, *J* = 2.1 Hz, 1H) ppm. ^{13}C{^{1}H} NMR (101 MHz, DMSO-d_6) δ_C:

171.9 (C), 166.5 (C), 154.1 (C), 146.0 (C), 122.3 (CH), 121.1 (C), 120.2 (CH), 113.0 (C) ppm. LRMS (ASAP) for $C_8H_6O_6$ $[M+H]^+$, calc 199.0, found 199.2.

12.8 Experimental protocols for synthesis and characterization of mixtures of 8a-8d and 12a-12d starting from model mixtures of 2a-2d

This section uses a model mixture made to resemble the ball mill mixture composition and the results are applied on the actual ball mill mixture in the next section.

Synthesis of mixture of pentyl esters 6a-6d from model mixture 2a-2d

The composition of a ball mill-derived mixture was mimicked by mixing 2,3-dihydroxybenzoic acid (**2a**, 100 wt% purity, 13.87 mg, 9.00 mmol), 3,4-dihydroxybenzoic acid (**2b**, 95 wt% purity, 10.87 mg, 6.70 mmol), 2,3-dihydroxyterephthalic acid (**2c**, 92 wt% purity, 5.61 mg, 2.60 mmol) and 4,5-dihydroxyisophthalic acid (**2d**, 83 wt% purity, 5.11 mg, 2.14 mmol), for a total mass of 35.46 mg. Upon analysis of the mixture by ^{1}H NMR spectroscopy, the relative molar ratios of the **2a**-**2d** are almost identical to those obtained from a real ball mill mixture (see section 3.3). Hence, this mixture was used to explore the derivatization towards plasticizer families **8a-8d** and **12a-12d**.

Table S17: Determination of the relative ratio of the model ball mill mixture by ^{1}H NMR spectroscopy.

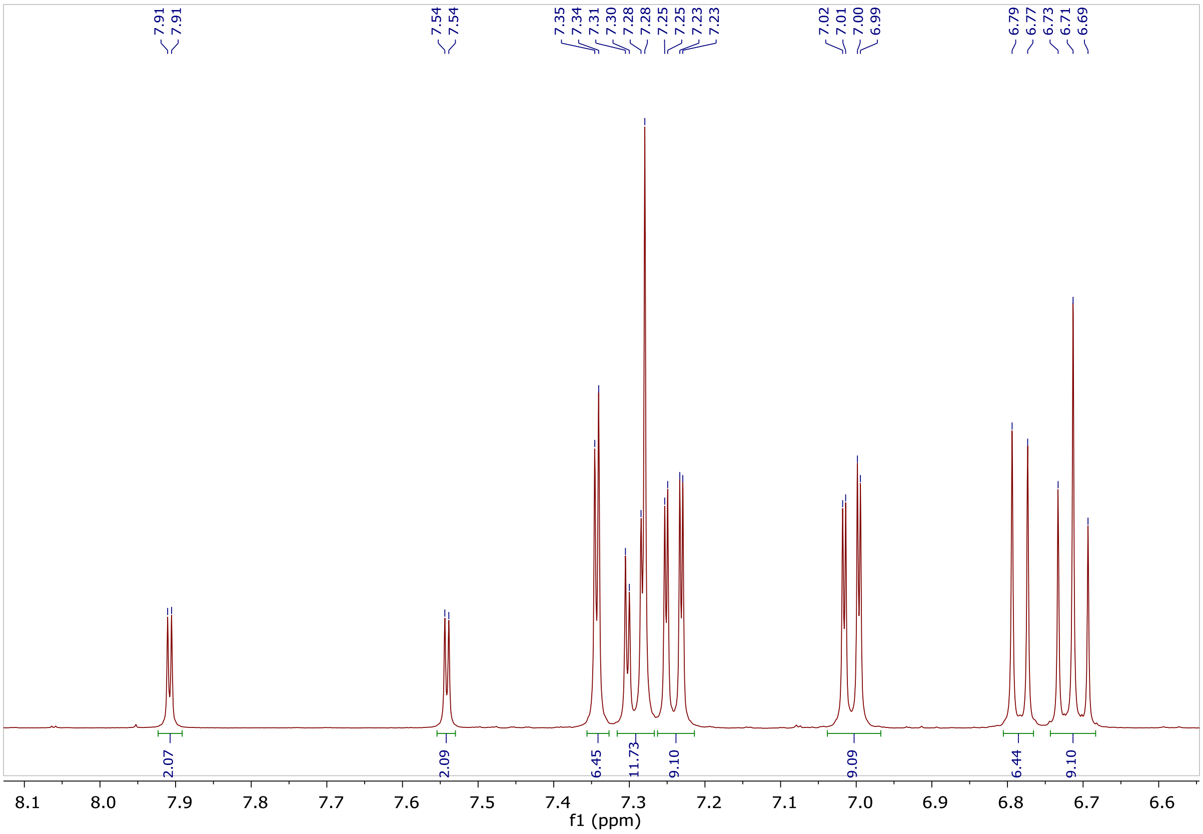

Isomer	Integration	#H-atoms	Relative ratio
**2a**	9.10	1	45%
**2b**	6.44	1	32%
**2c**	5.29	2	13%
**2d**	2.09	1	10%
**Total:**			100%

Fischer esterification of model ball mill mixture of 2a-2d towards 6a-6d [AGO-2024-0002]

The reaction was performed according to a modified literature procedure.[14] First, approximately 35 mL of pentan-1-ol was degassed to avoid oxidation of the catechol moieties by sparging with argon under ultrasonication for 15 minutes. The model ball mill mixture **2a**-**2d** [520.5 mg, 3.0 mmol (sum of isomers), ratio = 45:32:13:10 (**2a**:**2b**:**2c**:**2d**)] was dissolved in degassed pentan-1-ol (30 mL, 0.10 M) and sulfuric acid (33 µL, 0.60 mmol, 20 mol%) was added. The reaction mixture was stirred for 20 h at reflux temperature (138 °C) under an argon atmosphere. After the reaction time, the mixture was concentrated under reduced pressure and water (30 mL) was added. The obtained aqueous phase was extracted with MTBE (3 × 30 mL). The organic phases were combined and washed with a saturated, aqueous $NaHCO_3$ solution (2 × 30 mL) and brine (1 × 30 mL). The organic phase was dried over anhydrous Na_2SO_4, filtered, and then concentrated under reduced pressure. A dark brown oil (0.763 g, quant. mass-based yield) was obtained, containing **6a**, **6b**, **6c** and **6d** in the relative molar ratios 47:31:12:10 (**6a**:**6b**:**6c**:**6d**) (Table S18).

Table S18: Determination of the relative ratio of the mixture of pentyl esters 6a-6d derived from a model ball mill mixture by ^{1}H NMR spectroscopy.

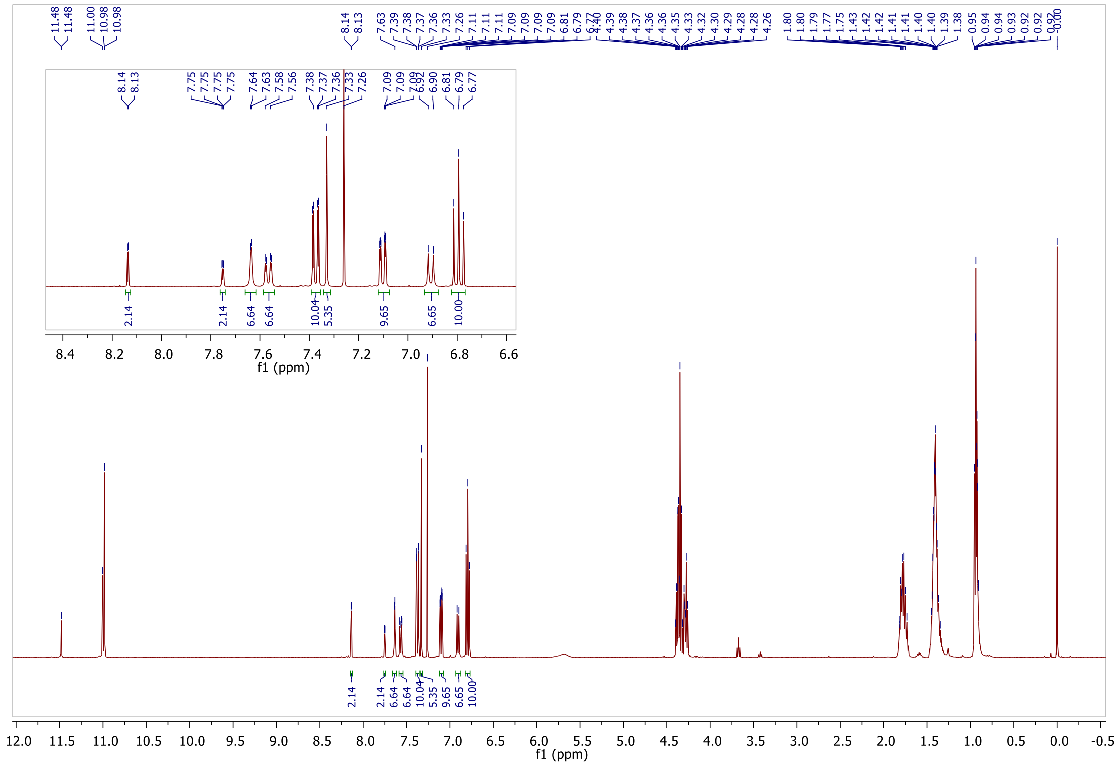

Isomer	Integration	#H-atoms	Relative ratio
**6a**	10.00	1	47%
**6b**	6.65	1	31%
**6c**	5.35	2	12%
**6d**	2.14	1	10%
Total:			100%

***O*-Acylation of pentyl esters (6a-6d) towards mixture of *O*-pentanoylated pentyl esters (12a-12d) [AGO-2024-0003 and DDV-2025-0247]**
[AGO-2024-0003]
Approximately 700 mg of potassium carbonate was ground with pestle and mortar. The ground potassium carbonate (518 mg, 3.75 mmol, 3.75 equiv.) was transferred into a 25 mL round-bottom flask and flame-dried under vacuum. The flask was kept under argon and sealed with a septum. A model ball mill-derived mixture of **6a**-**6d** [250 mg, 1.00 mmol, (sum of isomers), ratios = 47:31:12:10 (**6a**:**6b**:**6c**:**6d**)] was charged in a separate vial and quantitatively transferred to the round-bottom flask using EtOAc (2.85 mL, 0.35 M). The resulting suspension was ultrasonicated for 1 h. Thereafter, water (18.00 µL, 1.00 mmol, 1.00 equiv.) was added under vigorous stirring and the suspension was cooled using an ice bath. Subsequently, pentanoyl chloride (0.42 mL, 3.50 mmol, 3.50 equiv.) was slowly added into the reaction mixture over 15 minutes by syringe. The resulting reaction mixture was stirred for 4 h at 20 °C under an argon atmosphere. After the reaction time, the mixture was filtered, and the filtrate diluted with EtOAc (50 mL) and washed with an aqueous, saturated $NaHCO_3$ solution (1 × 40 mL), and brine (1 × 40 mL). The organic phase was dried over anhydrous Na_2SO_4, filtered and concentrated under reduced pressure. The resulting mixture (496.3 mg) still contained inorganic salts, as its ^{1}H NMR spectrum looked clean but was heavier than the theoretical mass (419.4 mg).

Hence, the mixture was redissolved in EtOAc (40 mL) and washed with distilled water (40 mL). Subsequently, the organic phase was dried with anhydrous Na_2SO_4, filtered and concentrated under reduced pressure. The resulting mixture of **12a**-**12d** was ^{1}H NMR pure (376.3 mg, 90% mass-based yield) and used as plasticizer in PVC. However, the T_g of the resulting polymer was higher than expected (T_g = 63.6 °C, average of two measurements). The mixture (197.4 mg) was redissolved and filtered over a short (4 cm) silica plug using EtOAc as eluent. The filtrate was concentrated under reduced pressure.

^{1}H NMR analysis of the resulting mixture (173.4 mg, 88% mass-based recovery) showed a complete conversion towards **12a**-**12d** in a ratio of 47:32:11:10 (**12a**:**12b**:**12c**:**12d**, see Table S19), respectively, indicating an overall mass-based yield of 79% (0.90 × 0.88). The T_g measured using the mixture after filtration over silica was T_g = 61.4 °C, an average of two measurements.

Table S19: Determination of the relative ratio of the mixture of *O*-pentanoylated pentyl esters 12a-12d derived from a model ball mill mixture by ^{1}H NMR spectroscopy.

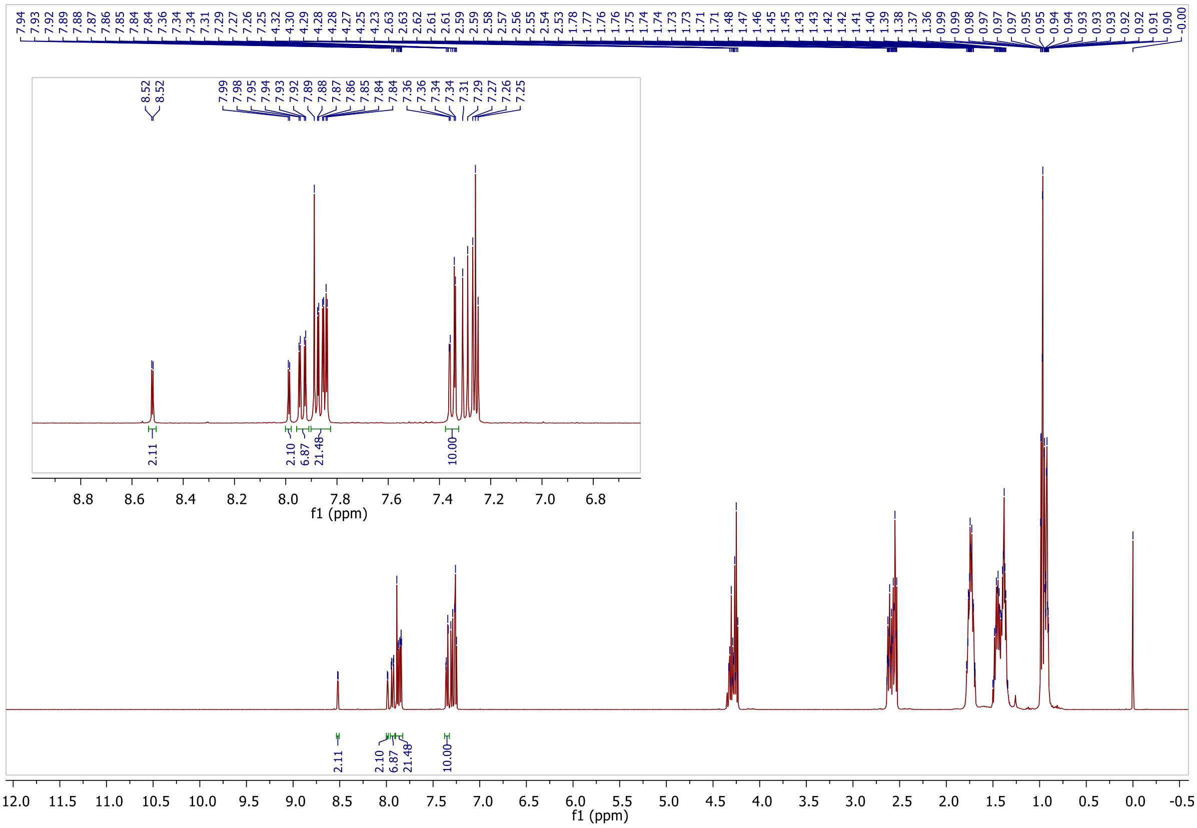

Isomer	Integration	#H-atoms	Relative ratio
**12a**	10.00	1	47%
**12b**	6.87	1	32%
**12c**	4.61[a]	2	11%
**12d**	2.10	1	10%
Total:			100%

[a] Calculated as 21.48 – 10.00 – 6.87 = 4.61

_O_-Acylation of pentyl esters (6a-6d) towards mixture of _O_-levulinoylated pentyl esters (8a-8d) [AGO-2024-0006 and AGO-2024-0009]
[AGO-2024-0006]
A round-bottom flask was charged with levulinic acid (330 mg, 2.84 mmol, 3.00 equiv.), EDC•HCl (544 mg, 2.84 mmol, 3.00 equiv.) and DMAP (34 mg, 0.28 mmol, 30 mol%). Using THF (7.0 mL, 0.13 M), a model ball mill-derived mixture of **6a**-**6d** [236 mg, 0.94 mmol, (sum of isomers), ratios = 47:31:12:10 (**6a**:**6b**:**6c**:**6d**)] was quantitatively added and the reaction mixture was stirred for 23 h at room temperature under an argon atmosphere. After the reaction time, the solvent was removed under reduced pressure and the residue was dissolved in EtOAc (30 mL). The organic phase was washed with aqueous HCl (1 M, 2 × 30 mL) and a saturated aqueous $NaHCO_3$ (2 × 30 mL). The organic layer was dried over anhydrous Na_2SO_4, filtered and concentrated under reduced pressure.

^{1}H NMR analysis of the resulting mixture (346.5 mg, 82% mass-based yield) showed an incomplete conversion. Hence, the reaction mixture was resubjected to the same reaction conditions and work-up, *i.e.* levulinic acid (330 mg, 2.84 mmol, 3.00 equiv.), EDC•HCl (544 mg, 2.84 mmol, 3.00 equiv.), DMAP (34 mg, 0.28 mmol, 30 mol%) in THF (7.0 mL, 0.13 M). After identical work-up, the resulting mixture of **8a**-**8d** was ^{1}H NMR pure (326.5 mg, 78% mass-based yield) and used as plasticizer in PLA. However, the T_g of the resulting polymer was higher than expected (T_g = 51.5 °C, average of two measurements). The mixture (108.9 mg) was redissolved and filtered over a short (4 cm) silica plug using EtOAc as eluent. The filtrate was concentrated under reduced pressure.

^{1}H NMR analysis of the resulting mixture (99.6 mg, 91% mass-based recovery) showed a complete conversion towards **8a**-**8d** in a ratio of 47:30:13:10 (**8a**:**8b**:**8c**:**8d**, see Table S20), respectively, indicating an overall mass-based yield of 71% (= 0.78 × 0.91). The T_g measured using the mixture after filtration over silica was T_g = 49.7 °C, an average of two measurements.

Table S20: Determination of the relative ratio of the mixture of *O*-levulinoylated pentyl esters 8a-8d derived from a model ball mill mixture by ^1H NMR spectroscopy (1).

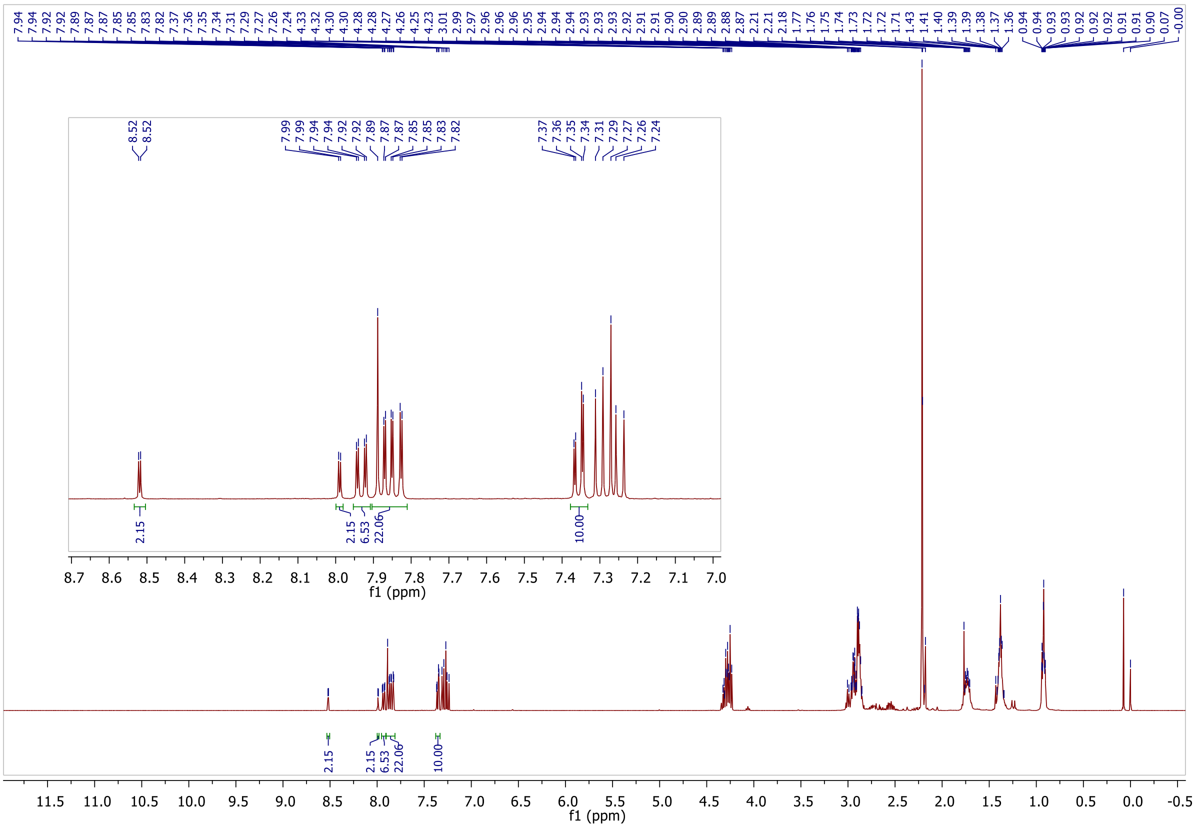

Isomer	Integration	#H-atoms	Relative ratio
**8a**	10.00	1	47%
**8b**	6.53	1	30%
**8c**	5.53[a]	2	13%
**8d**	2.15	1	10%
Total:			100%

[a] Calculated as 22.06 – 10.00 – 6.53 = 5.53

[AGO-2024-0009]

This mixture was used to determine concentration-dependent T_g reduction in PLA (Figure S30).

A round-bottom flask was charged with levulinic acid (581 mg, 5.00 mmol, 5.00 equiv.), EDC•HCl (958 mg, 5.00 mmol, 5.00 equiv.) and DMAP (37 mg, 0.30 mmol, 30 mol%).. Using THF (7.5 mL, 0.13 M), a model ball mill-derived mixture of **6a**-**6d** [250 mg, 1.00 mmol, (sum of isomers) ratios = 47:31:12:10 (**6a**:**6b**:**6c**:**6d**)] was quantitatively added and the reaction mixture was stirred for 20 h at room temperature under an argon atmosphere. After the reaction time, the solvent was removed under reduced pressure and the residue was dissolved in EtOAc (30 mL). The organic phase was washed with aqueous HCl (1 M, 2 × 30 mL) and a saturated aqueous $NaHCO_3$ (2 × 30 mL). The organic layer was dried over anhydrous Na_2SO_4, filtered and concentrated under reduced pressure.

^{1}H NMR analysis of the resulting mixture (411.4 mg, 92% mass-based yield) showed a complete conversion towards **8a**-**8d** in a ratio of 47:31:12:10 (**8a**:**8b**:**8c**:**8d**, see Table S21).

Table S21: Determination of the relative ratio of the mixture of *O*-levulinoylated pentyl esters 8a-8d derived from a model ball mill mixture by ^{1}H NMR spectroscopy (2).

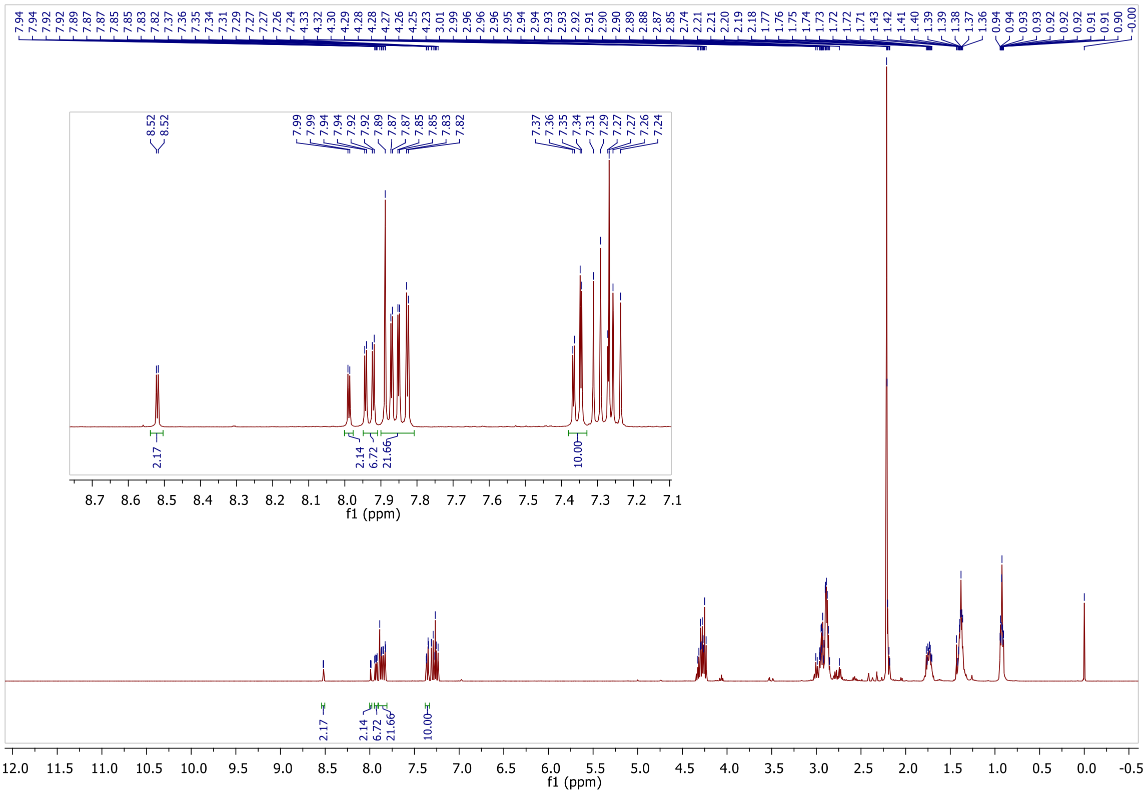

Isomer	Integration	#H-atoms	Relative ratio
**8a**	10.00	1	47%
**8b**	6.72	1	31%
**8c**	4.94[a]	2	12%
**8d**	2.14	1	10%
Total:			100%

[a] Calculated as 21.66 – 10.00 – 6.72 = 4.94

12.9 Experimental protocols for synthesis and characterization of mixtures of 8a-8d and 12a-12d starting from ball mill-derived mixture of 2a-2d

The results of this section are graphically shown in Figure 6 of the manuscript.

Synthesis of mixture of pentyl esters 6a-6d from ball mill mixture 2a-2d [AGO-2024-0010]

First, approximately 35 mL of pentan-1-ol was degassed to avoid oxidation of the catechol moieties by sparging with argon under ultrasonication for 15 min. Next, the ball mill-derived mixture of **2a**-**2d** [554.4 mg, 3.37 mmol, (sum of isomers), ratios = 46:31:12:11 (**2a**:**2b**:**2c**:**2d**)] was dissolved in degassed pentan-1-ol (34 mL, 0.10 M). Sulfuric acid (96 wt%, 69 mg, 37 µL, 0.674 mmol, 20 mol%) was added and the mixture was stirred for 22 h at reflux temperature (138 °C) under an argon atmosphere. After the reaction time, the mixture was concentrated under reduced pressure and water (40 mL) was added. The obtained aqueous phase was extracted with MTBE (3 × 40 mL). The combined organic phases were washed with distilled water (2 × 40 mL) and brine (1 × 40 mL). The organic phase was dried over anhydrous Na_2SO_4, filtered and concentrated under reduced pressure.

^{1}H NMR analysis of the resulting mixture (795.9 mg, 94% mass-based yield) showed a complete conversion towards **6a**-**6d** in a ratio of 49:30:11:10 (**6a**:**6b**:**6c**:**6d**).

Table S22: Determination of the relative ratio of the mixture of pentyl esters 6a-6d derived from a ball mill mixture by ^{1}H NMR spectroscopy.

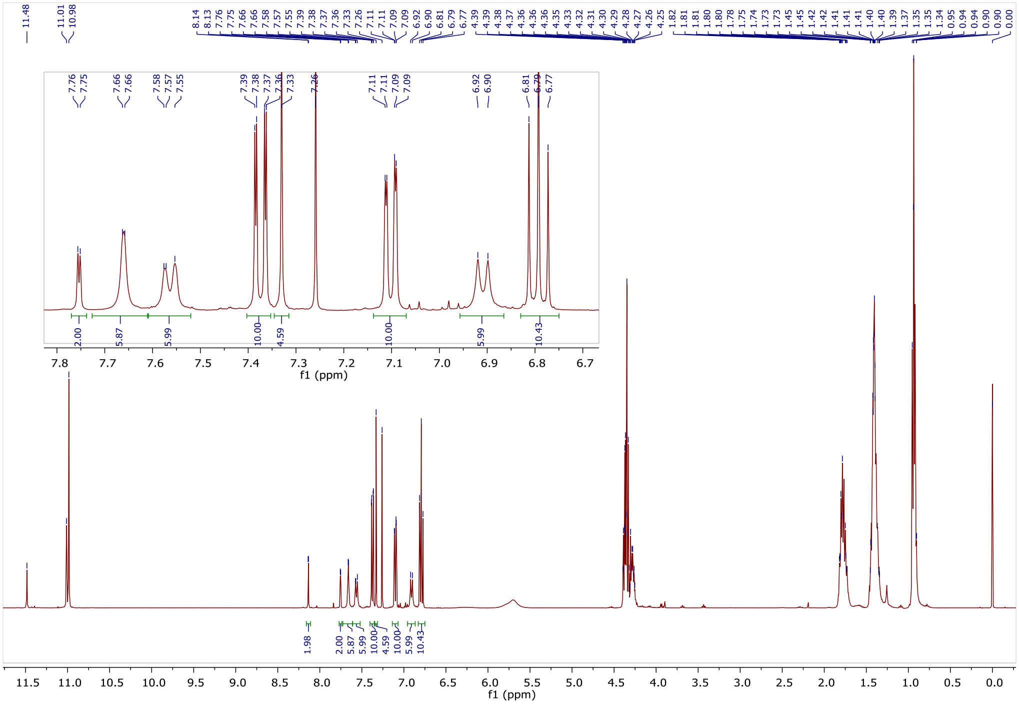

Isomer	Integration	#H-atoms	Relative ratio
**6a**	10.00	1	49%
**6b**	5.99	1	30%
**6c**	4.59	2	11%
**6d**	1.98	1	10%
Total:			100%

***O*-Acylation of ball mill-derived mixture of pentyl esters (6a-6d) towards mixture of *O*-pentanoylated pentyl esters (12a-12d) [AGO-2024-0012]**

Approximately 700 mg of potassium carbonate was ground with pestle and mortar. The ground potassium carbonate (560 mg, 4.05 mmol, 3.75 equiv.) was transferred into a 25 mL round-bottom flask and flame-dried under vacuum. The flask was kept under argon and sealed with a septum. The ball mill-derived mixture of **6a**-**6d** [272 mg, 1.08 mmol, (sum of isomers), ratios = 49:30:11:10 (**6a**:**6b**:**6c**:**6d**)], was charged in a separate vial and quantitatively transferred to the round-bottom flask using EtOAc (3.10 mL, 0.35 M). The resulting suspension was ultrasonicated for 1 h. Thereafter, water (19.5 µL, 1.08 mmol, 1.00 equiv.) was added under vigorous stirring and the suspension was cooled using an ice bath. Subsequently, pentanoyl chloride (456 mg, 0.45 mL, 3.78 mmol, 3.50 equiv.) was slowly added into the reaction mixture over 15 minutes by syringe. The resulting reaction mixture was stirred for 4 h at 20 °C under an argon atmosphere. After the reaction time, the mixture was filtered and the filtrate diluted with EtOAc (50 mL) and washed with water (1 × 40 mL), an aqueous, saturated $NaHCO_3$ solution (1 × 40 mL) and brine (1 × 40 mL). The organic phase was dried over anhydrous Na_2SO_4, filtered and concentrated under reduced pressure. Finally, the mixture was filtered over a silica plug (4 cm) using EtOAc as eluent. The filtrate was concentrated under reduced pressure, yielding a mixture (575.2 mg) heavier than the theoretical mass (452.9 mg). ^1H NMR analysis showed the presence of pentanoic acid.

Hence, the mixture was redissolved in EtOAc (40 mL) and washed with aqueous saturated $NaHCO_3$ (2 × 40 mL). The organic phase was once more filtered over a silica plug (4 cm) using EtOAc as eluent and dried with anhydrous Na_2SO_4, filtered and concentrated under reduced pressure.

^1H NMR analysis of the resulting mixture (474.0 mg, quant. mass-based yield) showed no remaining pentanoic acid and a complete conversion towards **12a**-**12d** in a ratio of 47:32:11:10 (**12a**:**12b**:**12c**:**12d**).

Table S23: Determination of the relative ratio of the mixture of *O*-pentanoylated pentyl esters 12a-12d derived from a ball mill-derived mixture by ^{1}H NMR spectroscopy.

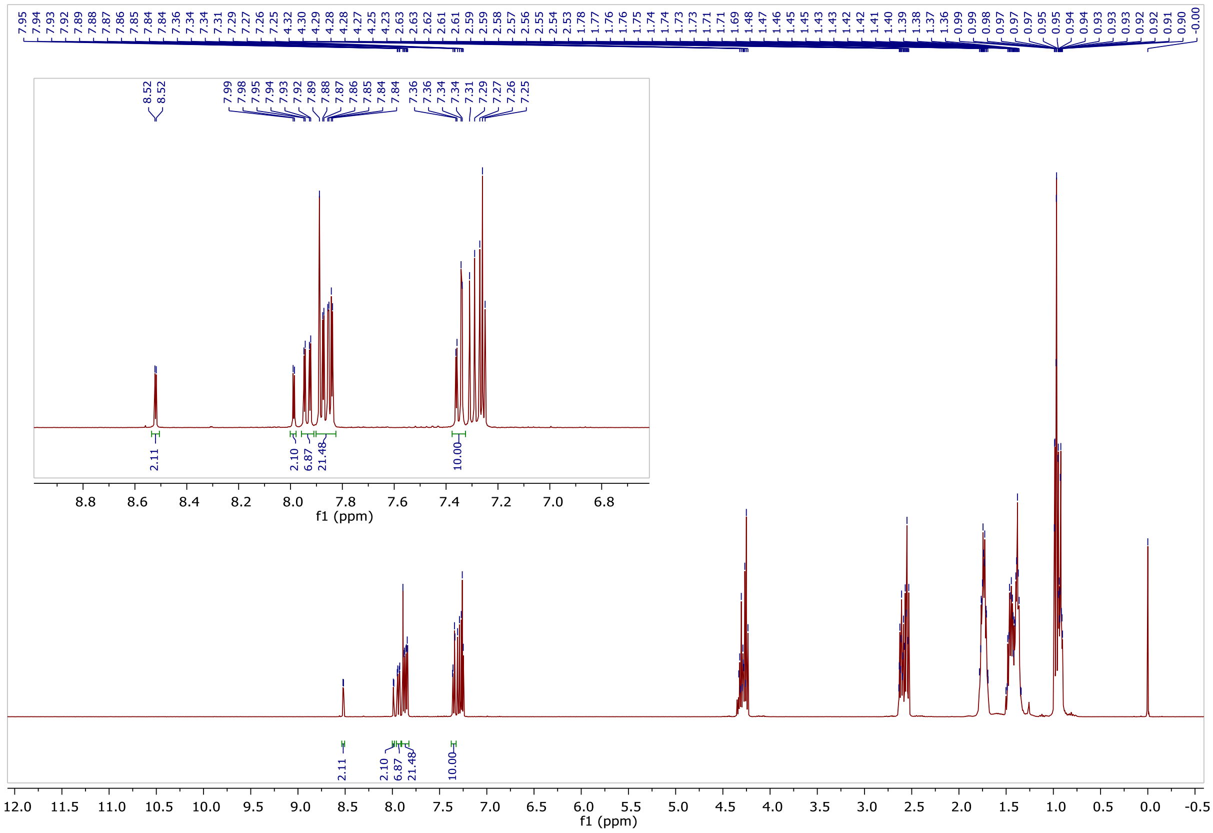

Isomer	Integration	#H-atoms	Relative ratio
**12a**	10.00	1	47%
**12b**	6.87	1	32%
**12c**	4.61^a	2	11%
**12d**	2.10	1	10%
Total:			100%

^{a} Calculated as 21.48 – 10.00 – 6.87 = 4.61.

O-Acylation of ball mill-derived mixture of pentyl esters (6a-6d) towards mixture of *O*-levulinoylated pentyl esters (8a-8d) [AGO-2024-0011]

A round-bottom flask was charged with levulinic acid (525 mg, 4.53 mmol, 5.00 equiv.), EDC•HCl (867 mg, 4.53 mmol, 5.00 equiv.), DMAP (33 mg, 0.27 mmol, 30 mol%) and THF (6.8 mL, 0.13 M). The ball mill-derived mixture of **6a-6d** [227.4 mg, 0.905 mmol (sum of isomers), ratio = 49:30:11:10 (**6a**:**6b**:**6c**:**6d**)] was added and the reaction mixture was stirred for 20 h at room temperature under an argon atmosphere. After the reaction time, the solvent was removed under reduced pressure and the residue was dissolved in EtOAc (30 mL). The obtained organic phase was washed with aqueous HCl (1 M, 3 × 30 mL) and a saturated aqueous $NaHCO_3$ (3 × 30 mL). The organic layer was dried over anhydrous Na_2SO_4, filtered and concentrated under reduced pressure. Finally, the mixture was filtered over a silica plug (4 cm) twice using EtOAc as eluent. The filtrate was concentrated under reduced pressure.

^{1}H NMR analysis of the resulting mixture (359.3 mg, 89% mass-based yield) showed no pentanoic acid and a complete conversion towards **8a-8d** in a ratio of 48:30:12:10 (**8a**:**8b**:**8c**:**8d**).

Table S24: Determination of the relative ratio and purity of the mixture of *O*-levulinoylated pentyl esters 8a-8d derived from a ball mill-derived mixture by quantitative ^{1}H NMR spectroscopy.

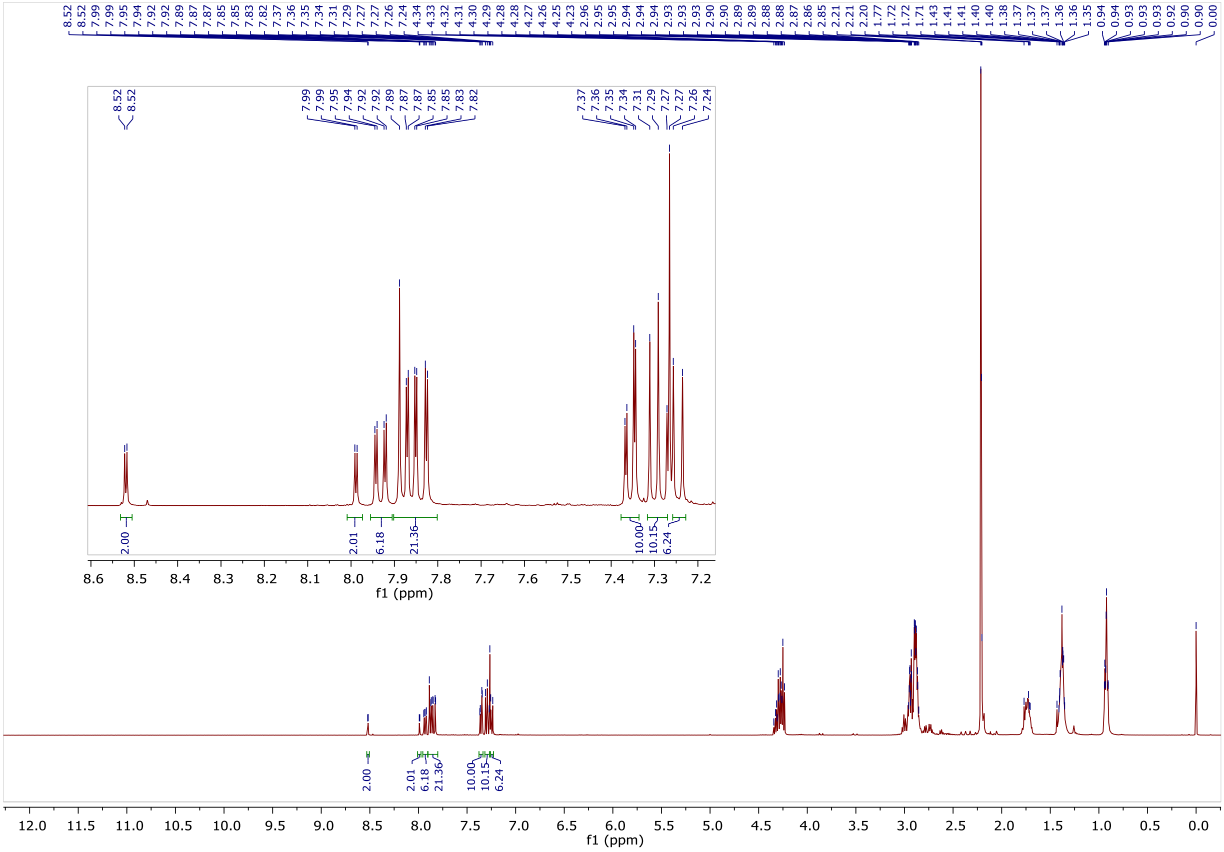

Isomer	Integration	#H-atoms	Relative ratio
**12a**	10.00	1	48
**12b**	6.18	1	30
**12c**	5.18[a]	2	12
**12d**	2.00	1	10
Total:			100

[a] Calculated as 21.36 – 10.00 – 6.18 = 5.18.

13. NMR spectra of plasticizer candidates

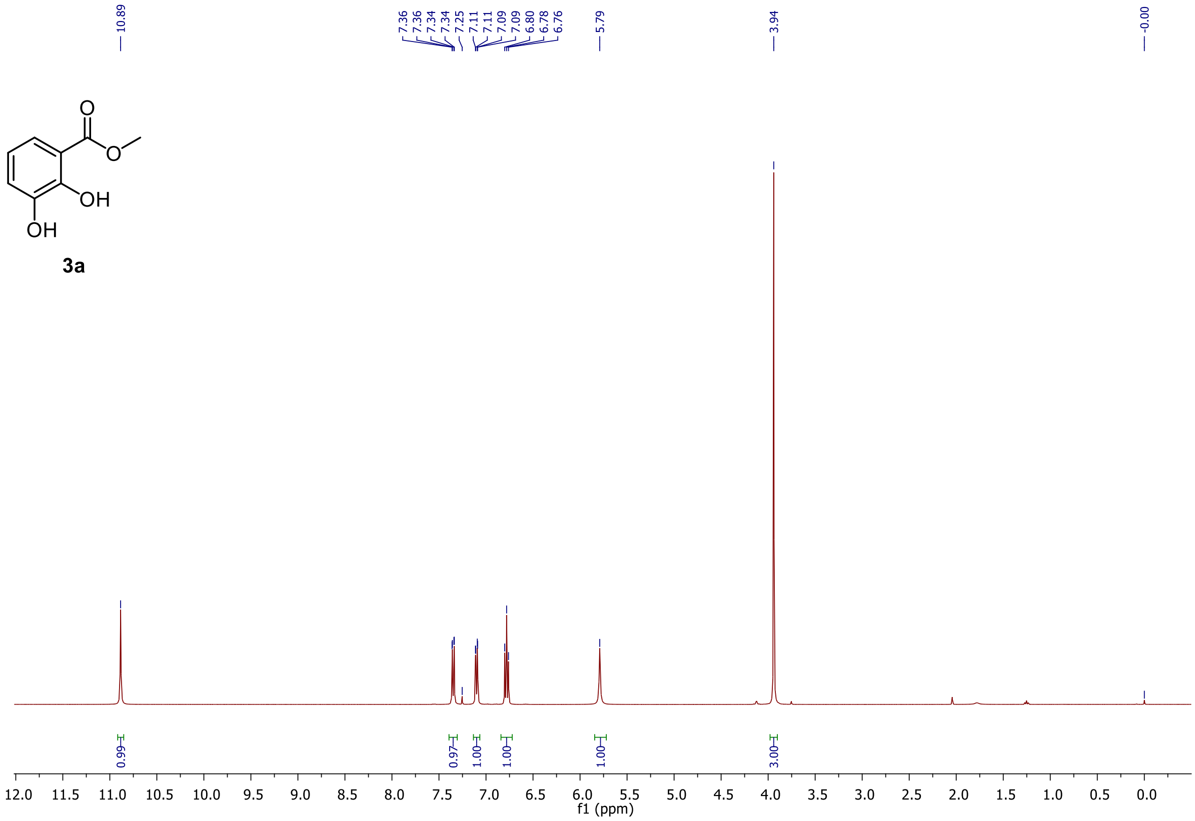

Figure S32: ^{1}H NMR (400 MHz, $CDCl_3$) spectrum of methyl 2,3-dihydroxybenzoate (3a) [DDV-AG-001].

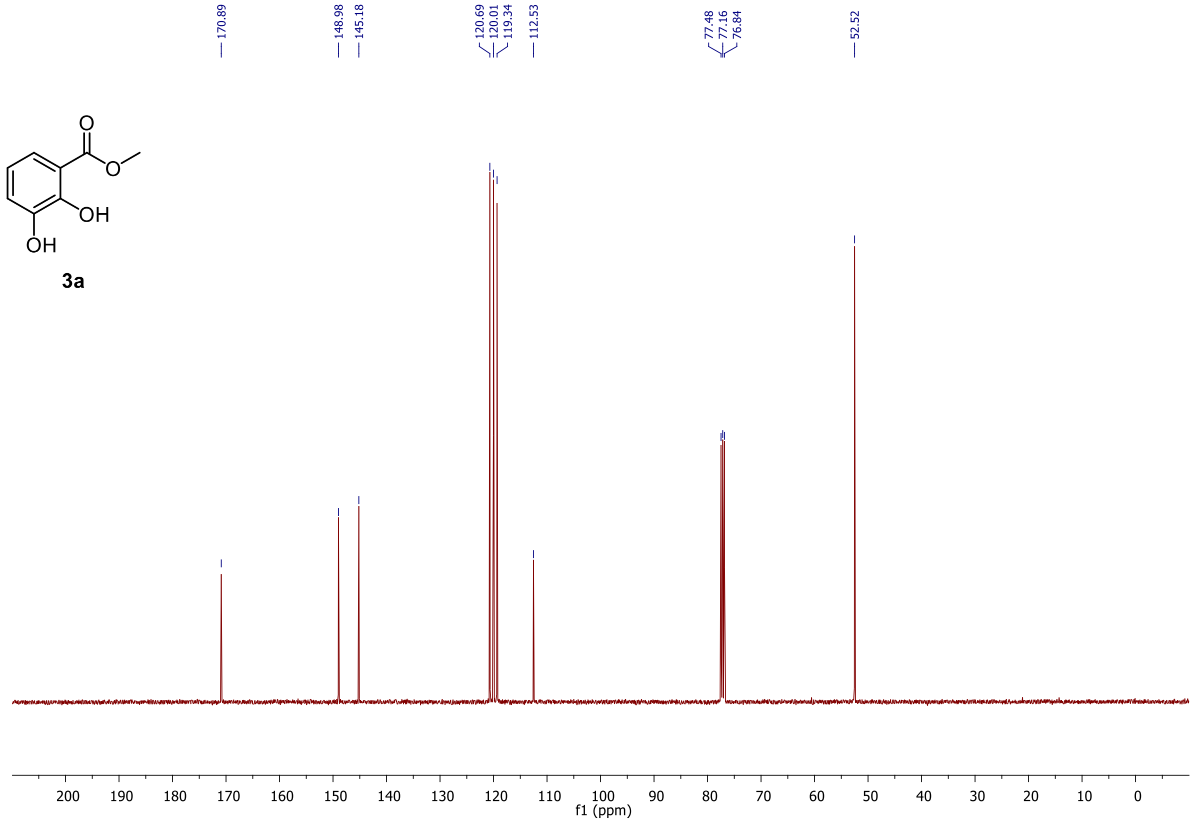

Figure S33: $^{13}C\{^{1}H\}$ NMR (101 MHz, $CDCl_3$) spectrum of methyl 2,3-dihydroxybenzoate (3a) [DDV-AG-001].

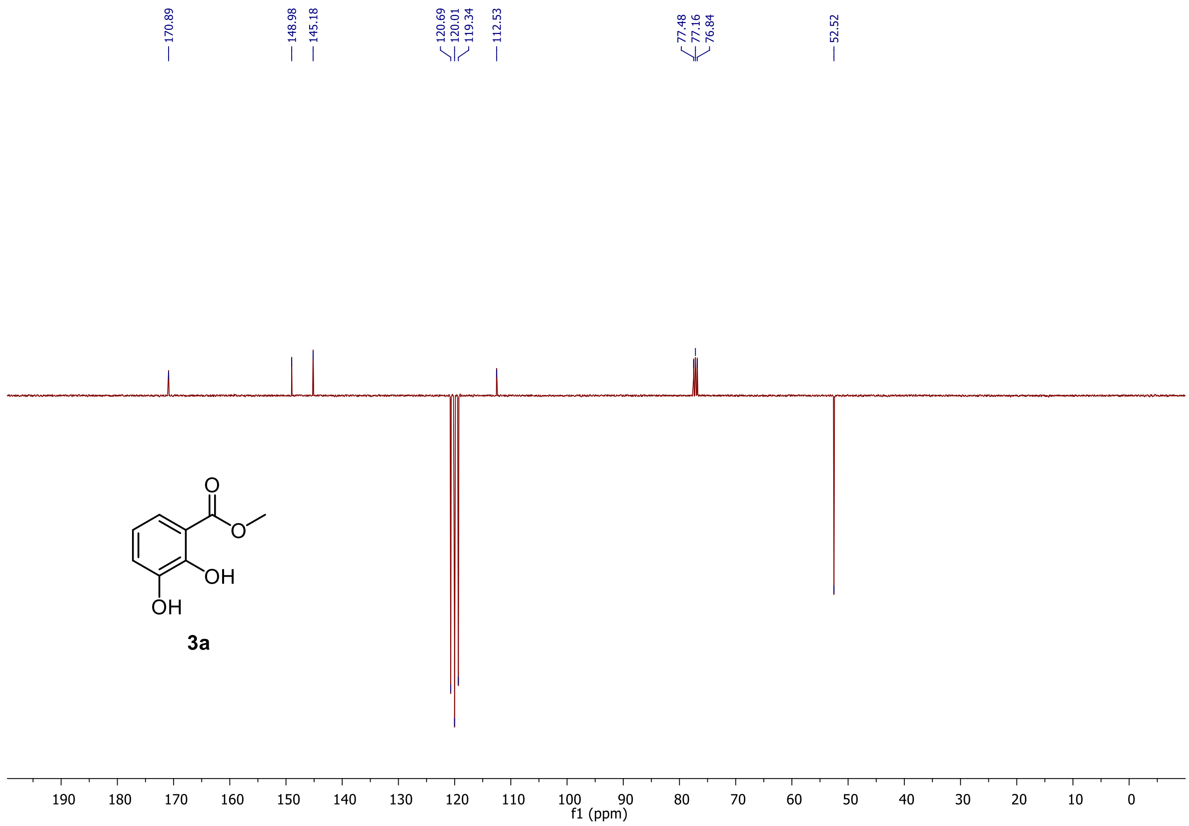

Figure S34: ^{13}C APT NMR (101 MHz, $CDCl_3$) spectrum of methyl 2,3-dihydroxybenzoate (3a) [DDV-AG-001].

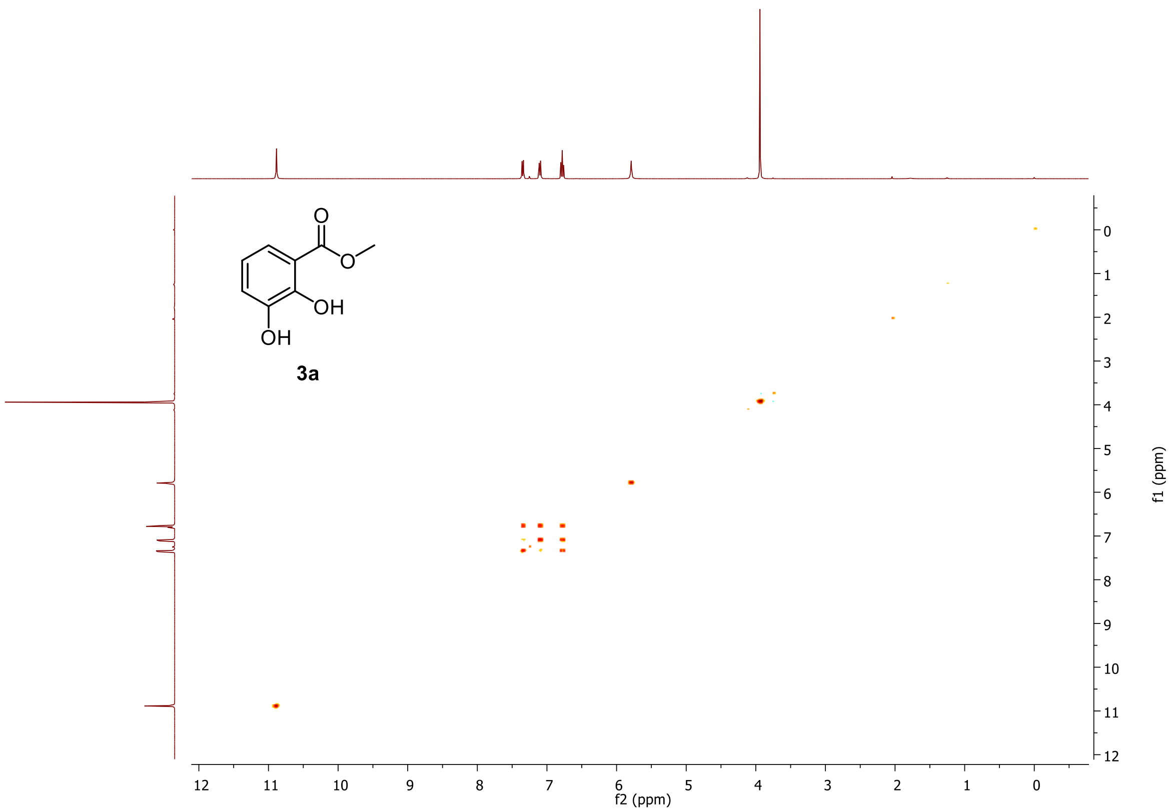

Figure S35: ^{1}H COSY NMR (400 MHz, $CDCl_3$) spectrum of methyl 2,3-dihydroxybenzoate (3a) [DDV-AG-001].

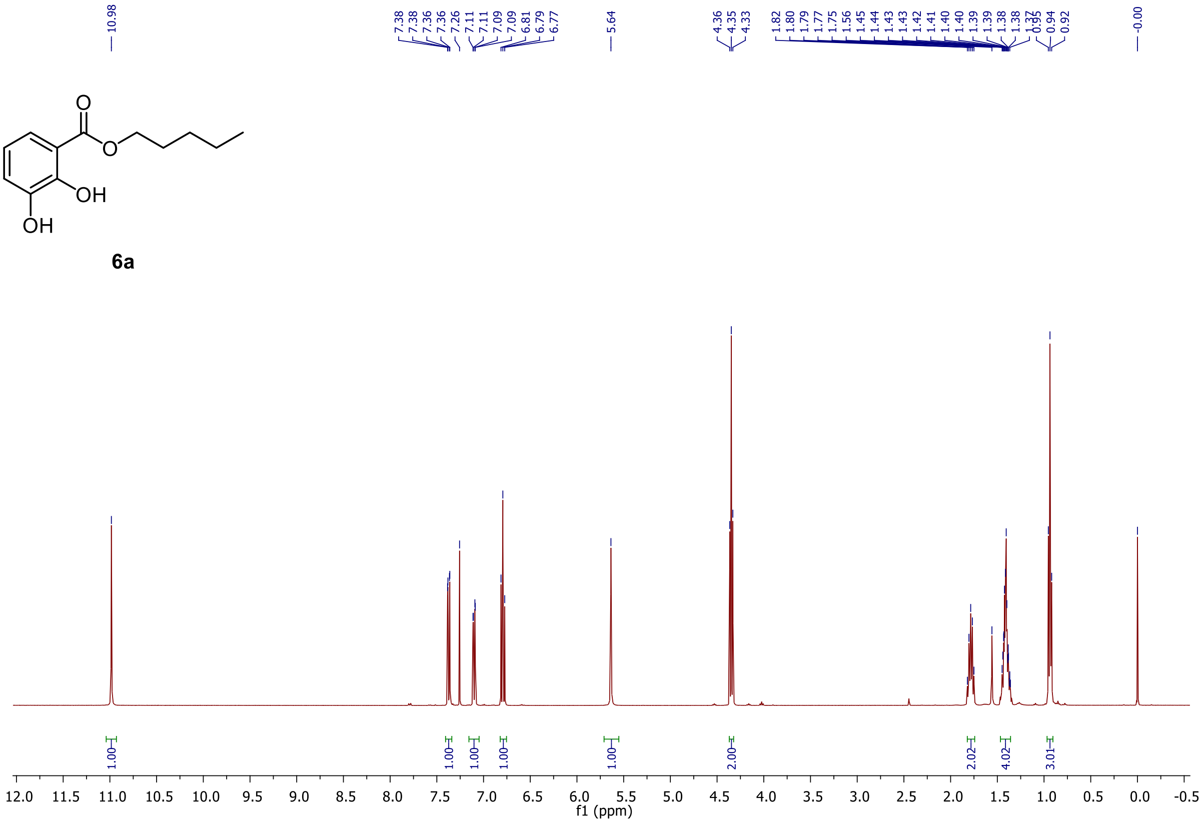

Figure S36: ^{1}H NMR (400 MHz, $CDCl_3$) spectrum of pentyl 2,3-dihydroxybenzoate (6a) [DDV-AG-004].

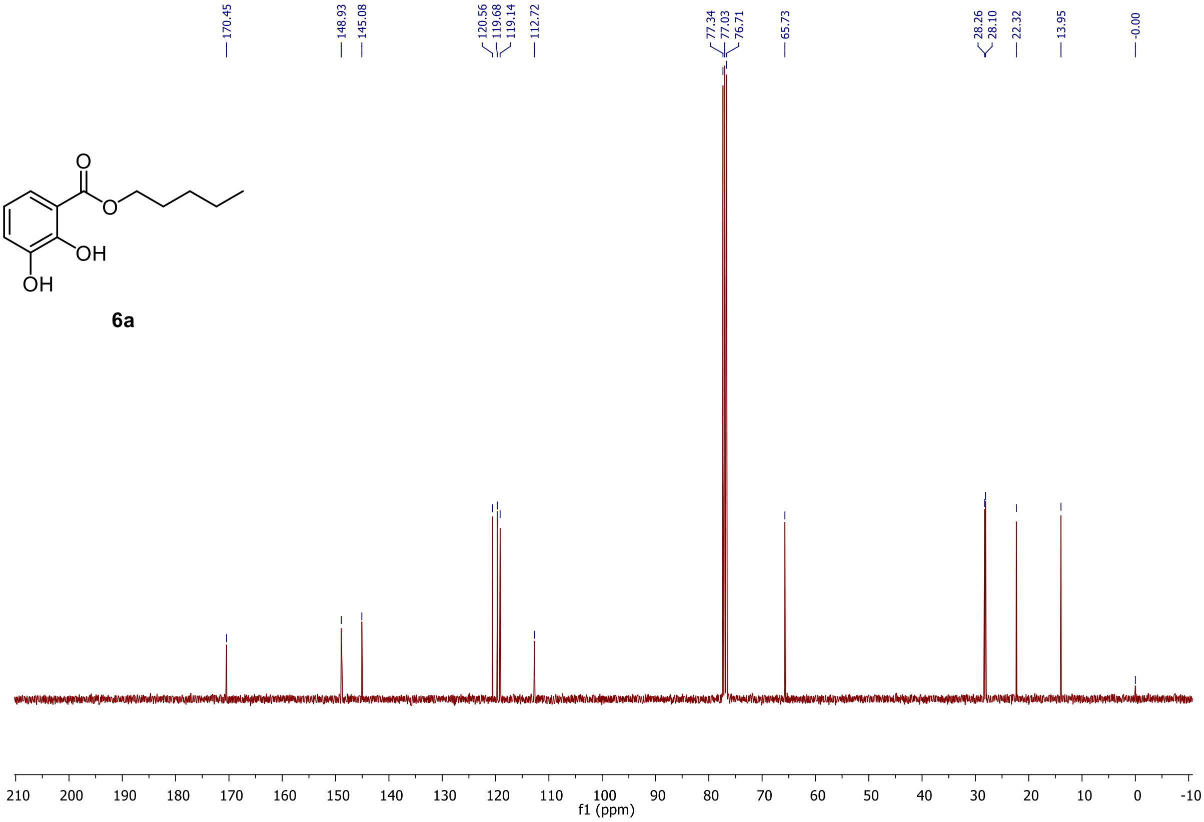

Figure S37: $^{13}C\{^{1}H\}$ NMR (101 MHz, $CDCl_3$) spectrum of pentyl 2,3-dihydroxybenzoate (6a) [DDV-AG-004].

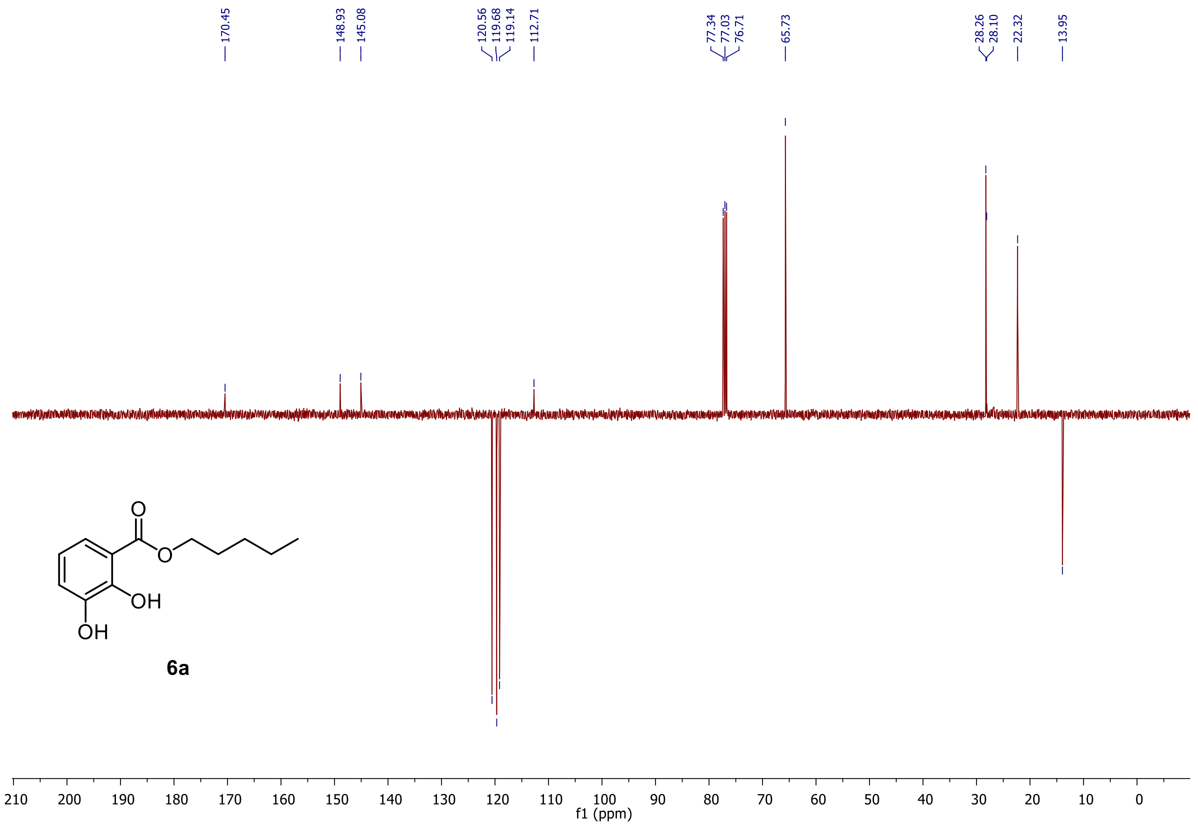

Figure S38: ^{13}C APT NMR (101 MHz, CDCl$_3$) spectrum of pentyl 2,3-dihydroxybenzoate (6a) [DDV-AG-004].

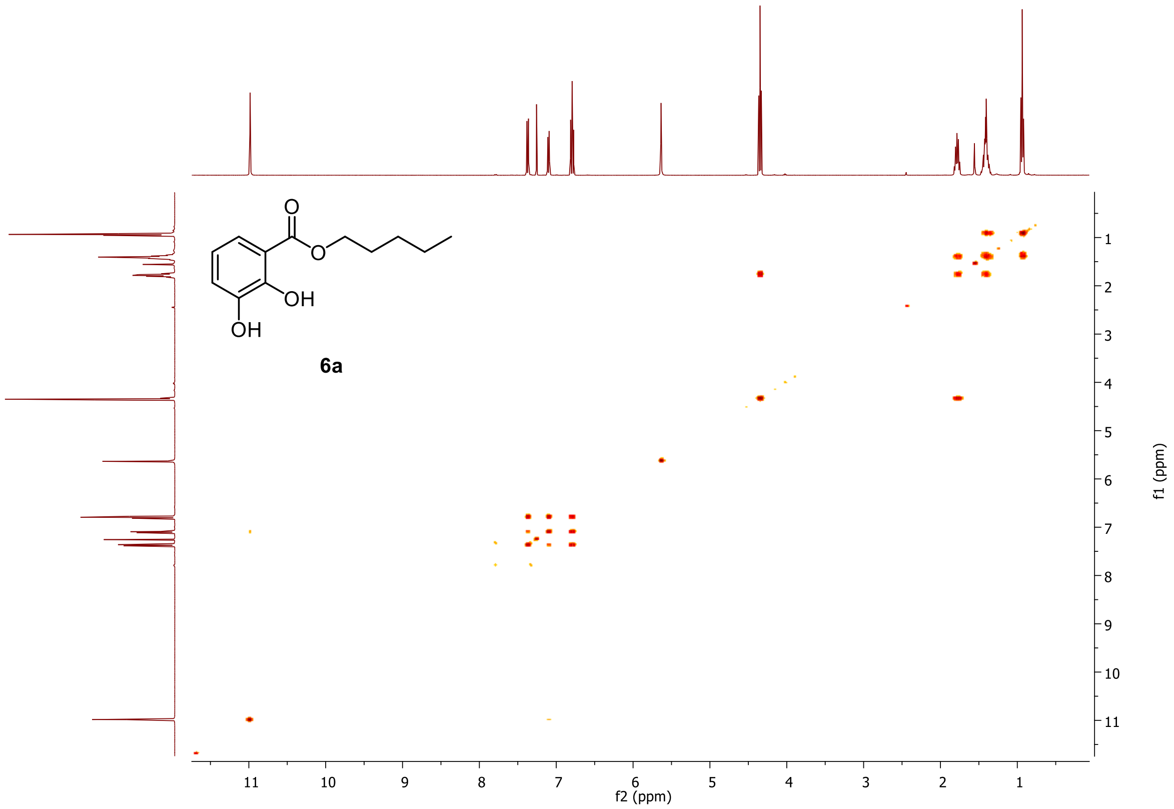

Figure S39: ^{1}H COSY NMR (400 MHz, CDCl$_3$) spectrum of pentyl 2,3-dihydroxybenzoate (6a) [DDV-AG-004].

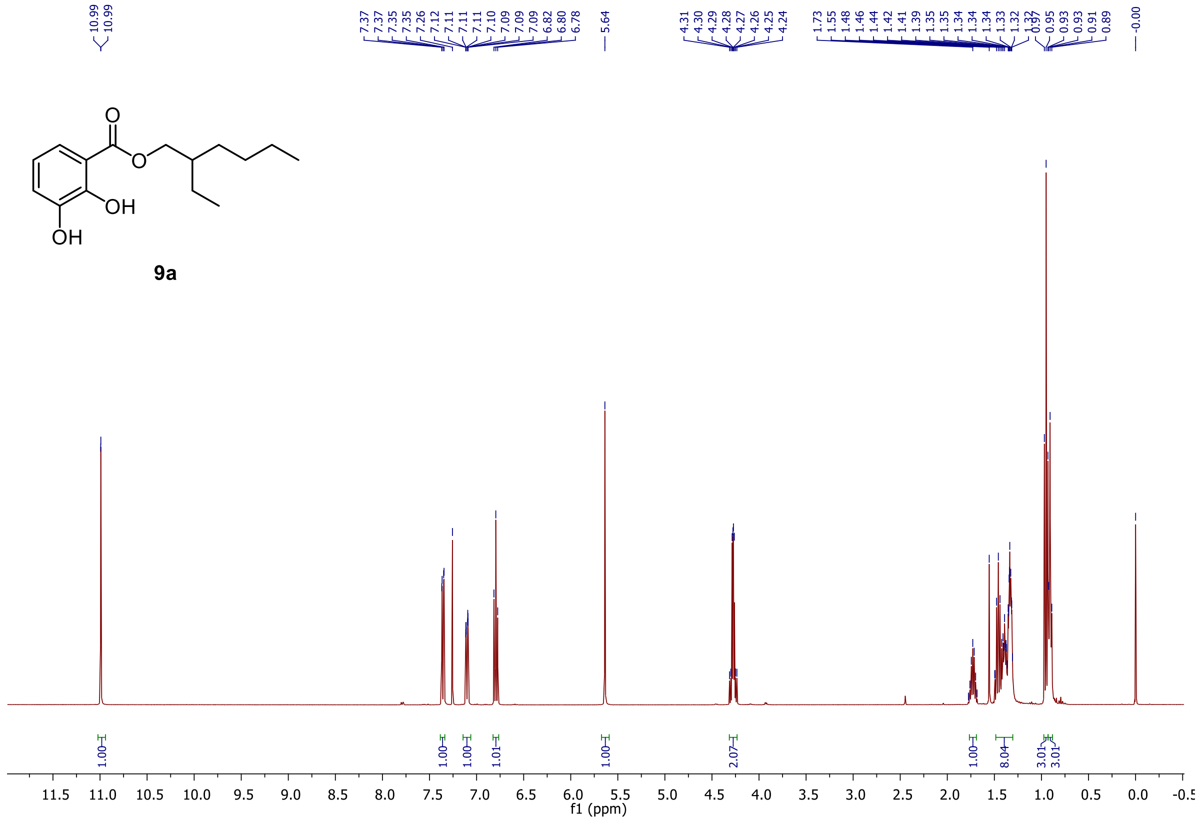

Figure S40: ^{1}H NMR (400 MHz, $CDCl_3$) spectrum of 2-ethylhexyl 2,3-dihydroxybenzoate (9a) [DDV-AG-005].

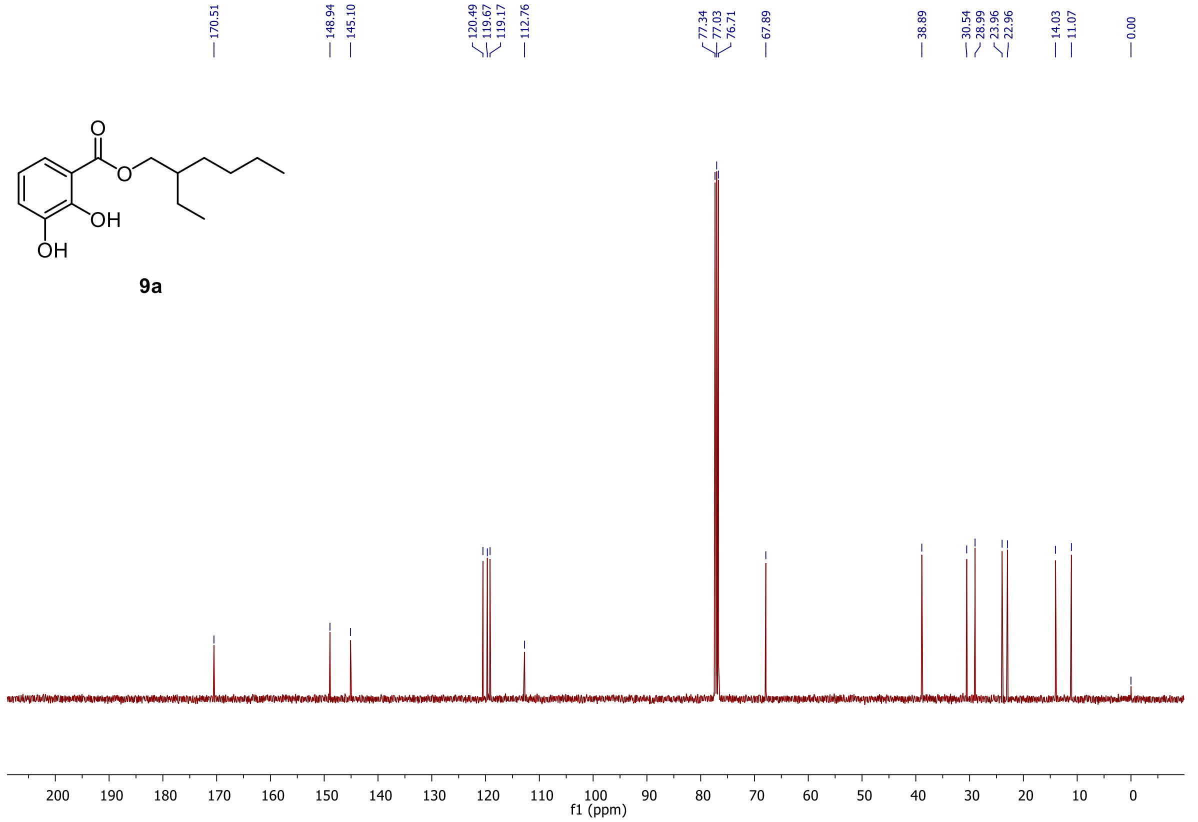

Figure S41: $^{13}C\{^{1}H\}$ NMR (101 MHz, $CDCl_3$) spectrum of 2-ethylhexyl 2,3-dihydroxybenzoate (9a) [DDV-AG-005].

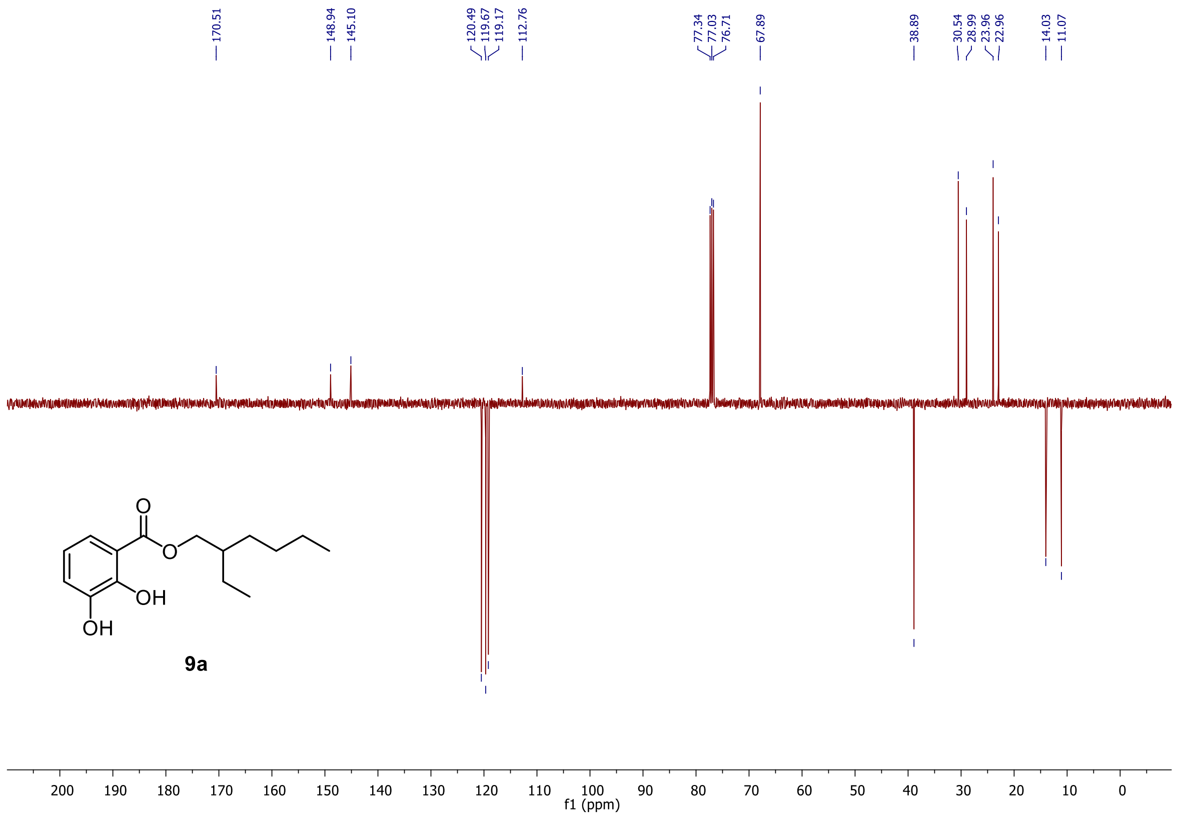

Figure S42: ^{13}C APT NMR (101 MHz, $CDCl_3$) spectrum of 2-ethylhexyl 2,3-dihydroxybenzoate (9a) [DDV-AG-005].

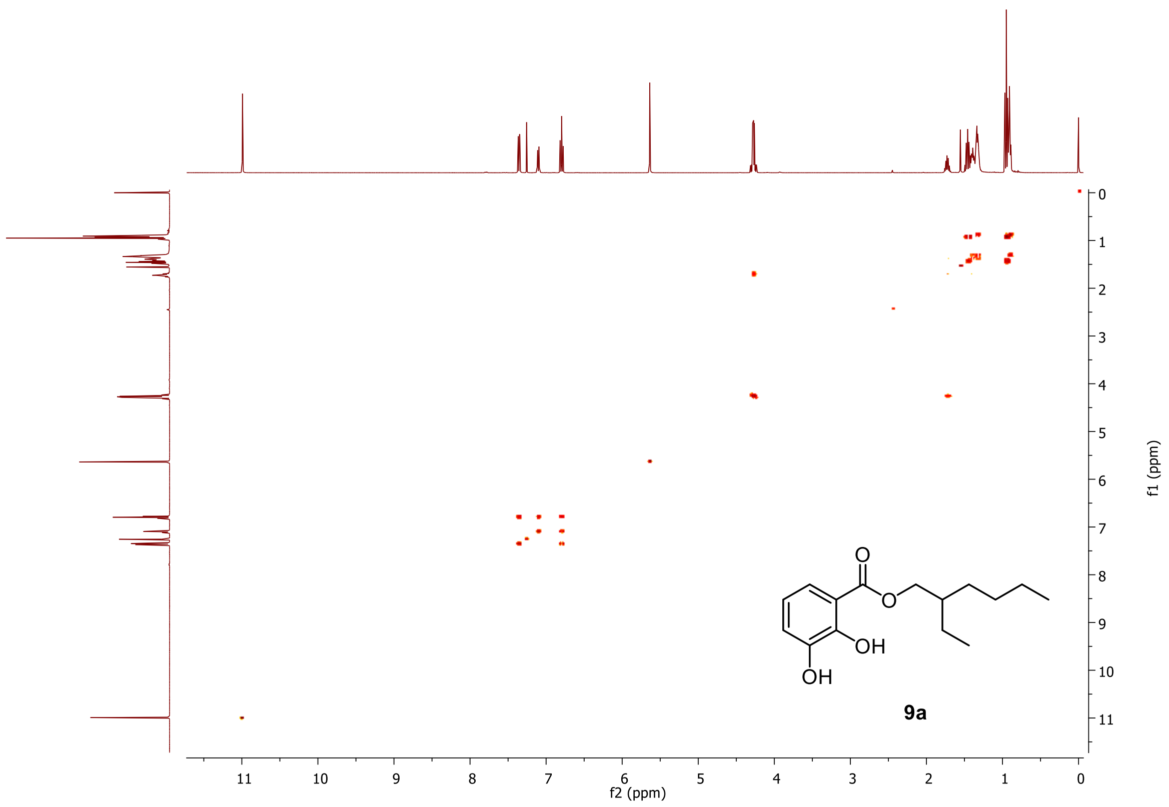

Figure S43: ^{1}H COSY NMR (400 MHz, $CDCl_3$) spectrum of 2-ethylhexyl 2,3-dihydroxybenzoate (9a) [DDV-AG-005].

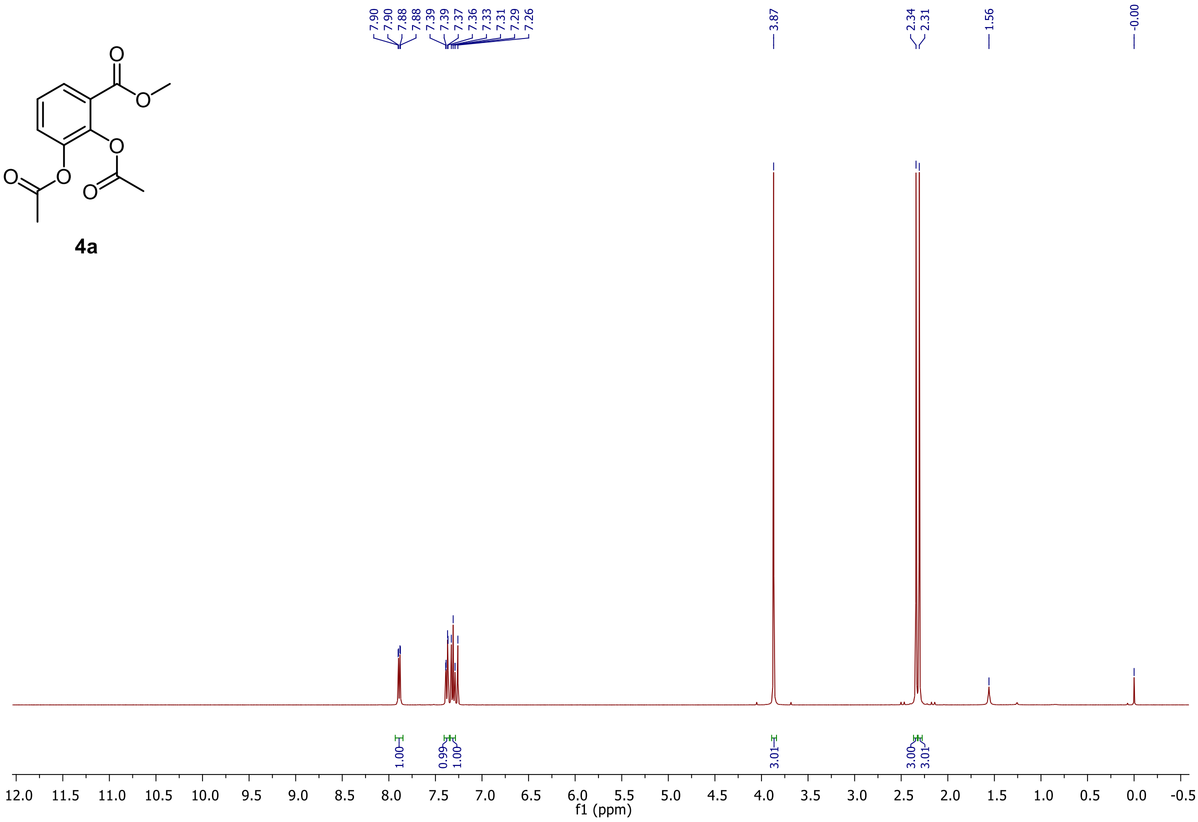

Figure S44: ¹H NMR (400 MHz, CDCl₃) spectrum of methyl 2,3-bis(acetyloxy)benzoate (4a) [DDV-AG-026].

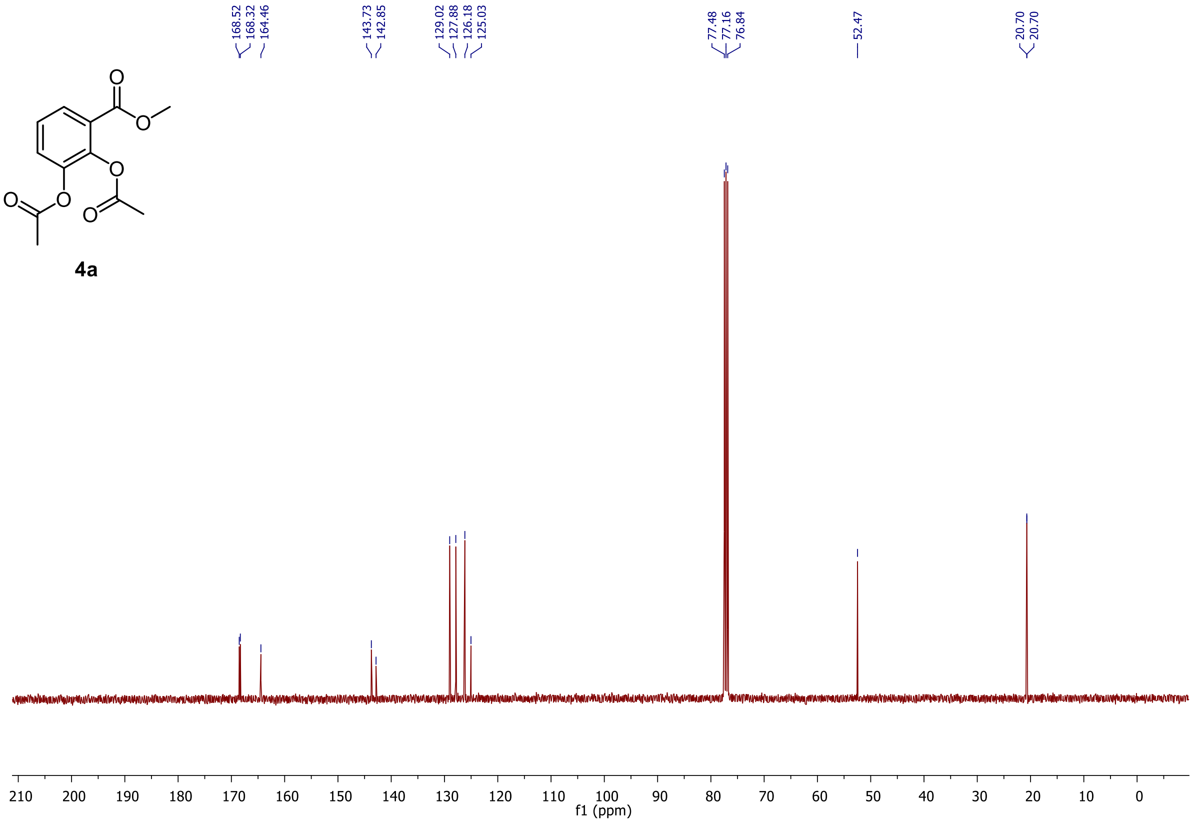

Figure S45: ¹³C{¹H} NMR (101 MHz, CDCl₃) spectrum of methyl 2,3-bis(acetyloxy)benzoate (4a) [DDV-AG-026].

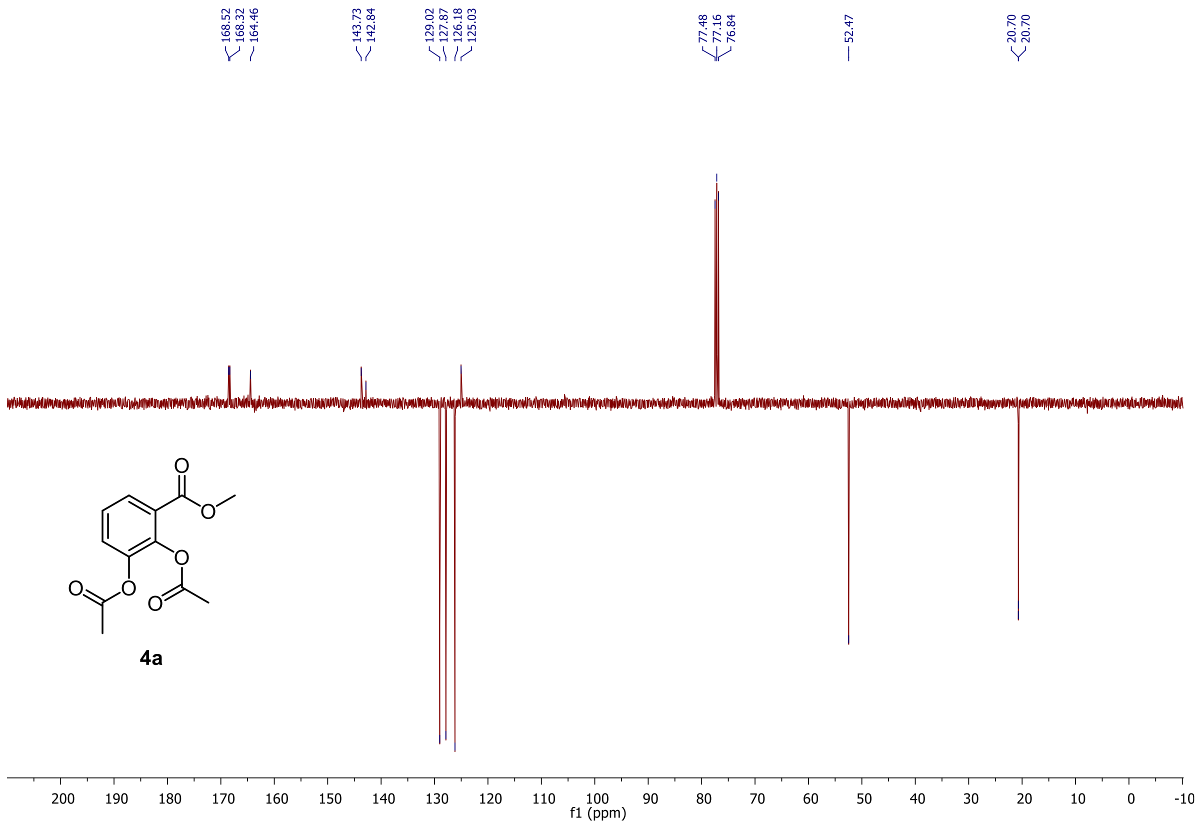

Figure S46: ^{13}C APT NMR (101 MHz, CDCl$_3$) spectrum of methyl 2,3-bis(acetyloxy)benzoate (4a) [DDV-AG-026].

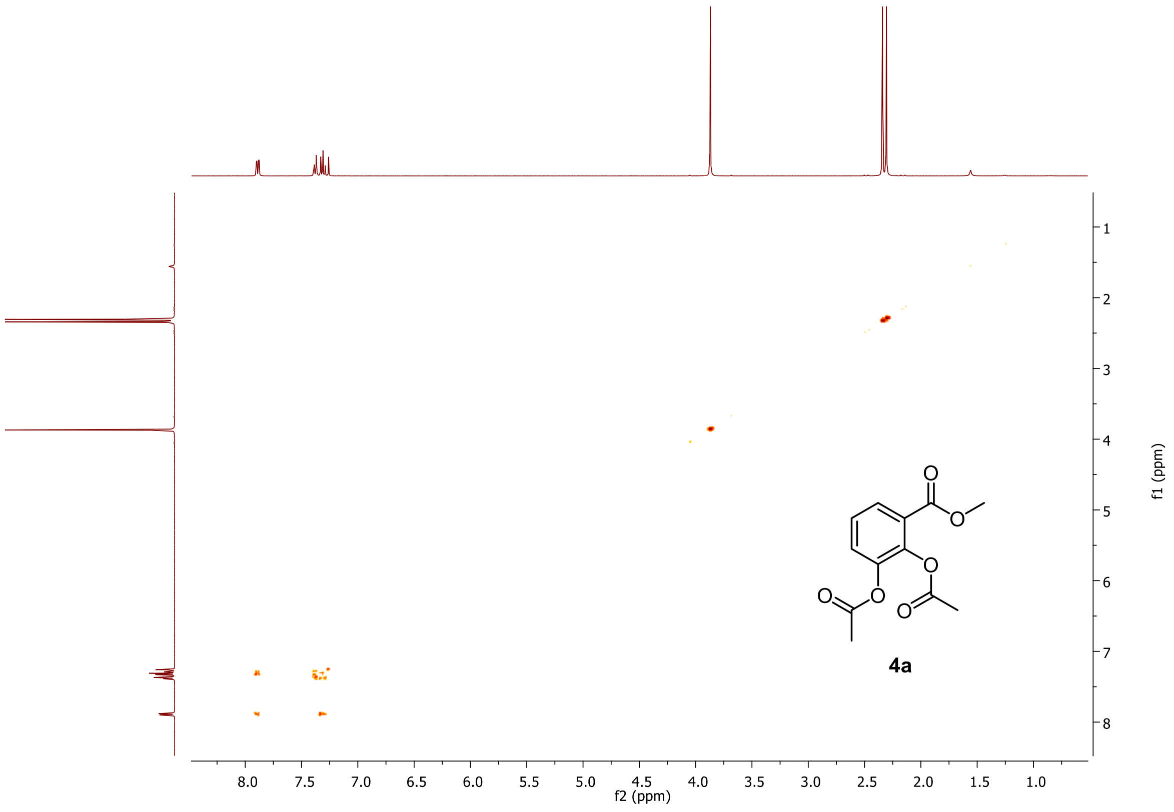

Figure S47: ^{1}H COSY NMR (400 MHz, CDCl$_3$) spectrum of methyl 2,3-bis(acetyloxy)benzoate (4a) [DDV-AG-026].

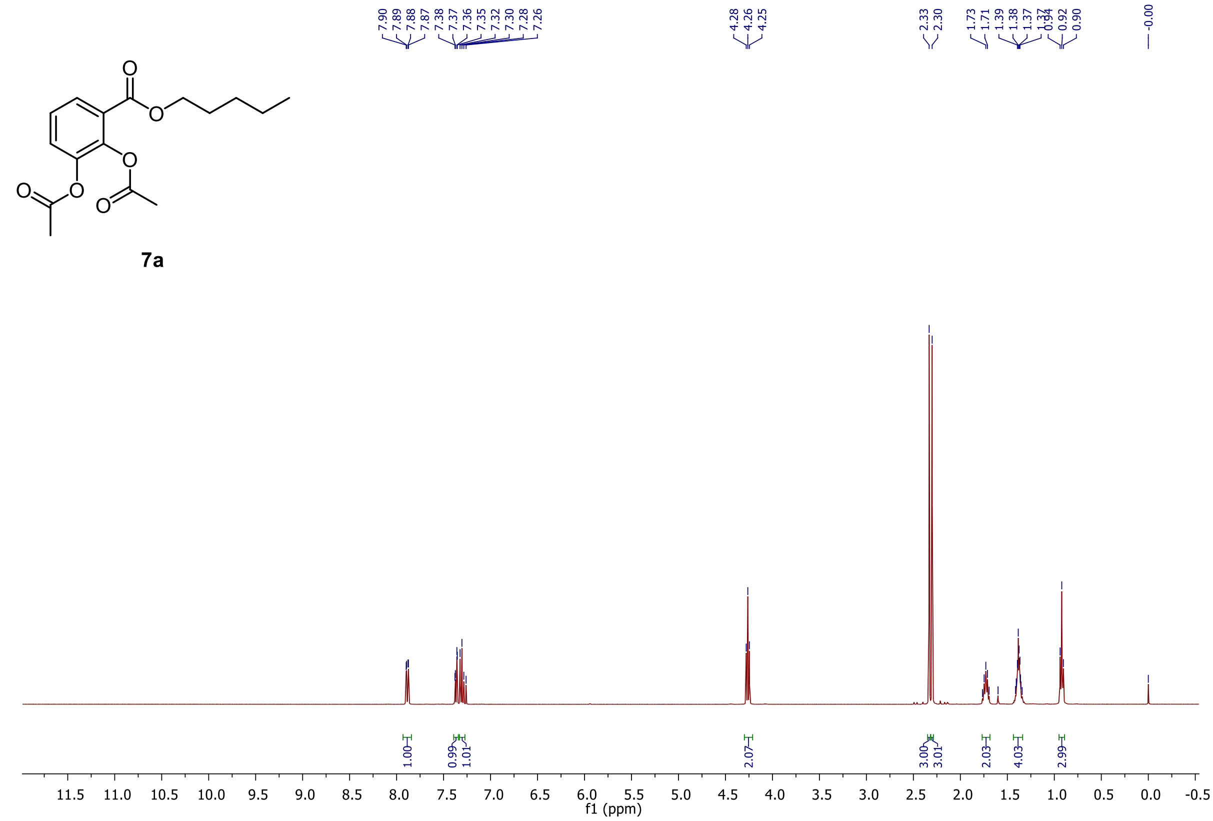

Figure S48: ^{1}H NMR (400 MHz, $CDCl_3$) spectrum of pentyl 2,3-bis(acetyloxy)benzoate (7a) [DDV-AG-010].

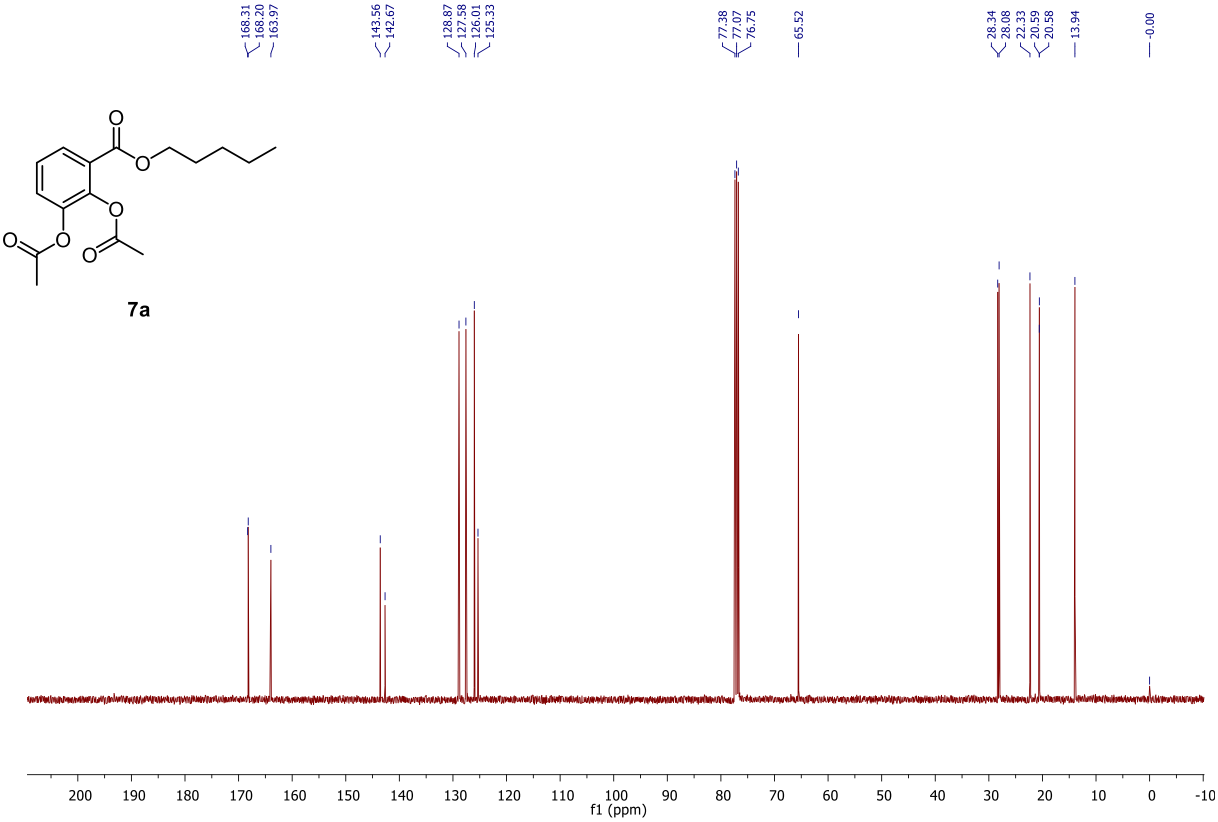

Figure S49: $^{13}C\{^{1}H\}$ NMR (101 MHz, $CDCl_3$) spectrum of pentyl 2,3-bis(acetyloxy)benzoate (7a) [DDV-AG-010].

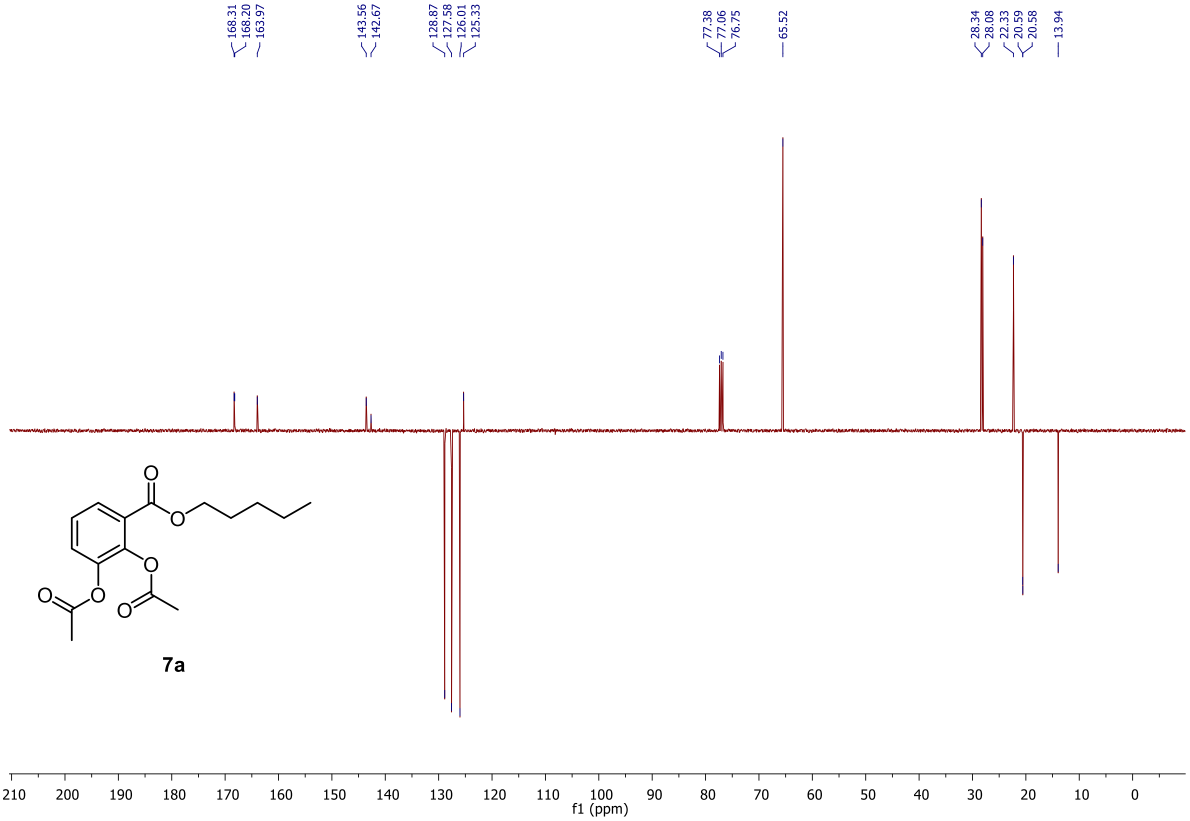

Figure S50: ^{13}C APT NMR (101 MHz, $CDCl_3$) spectrum of pentyl 2,3-bis(acetyloxy)benzoate (7a) [DDV-AG-010].

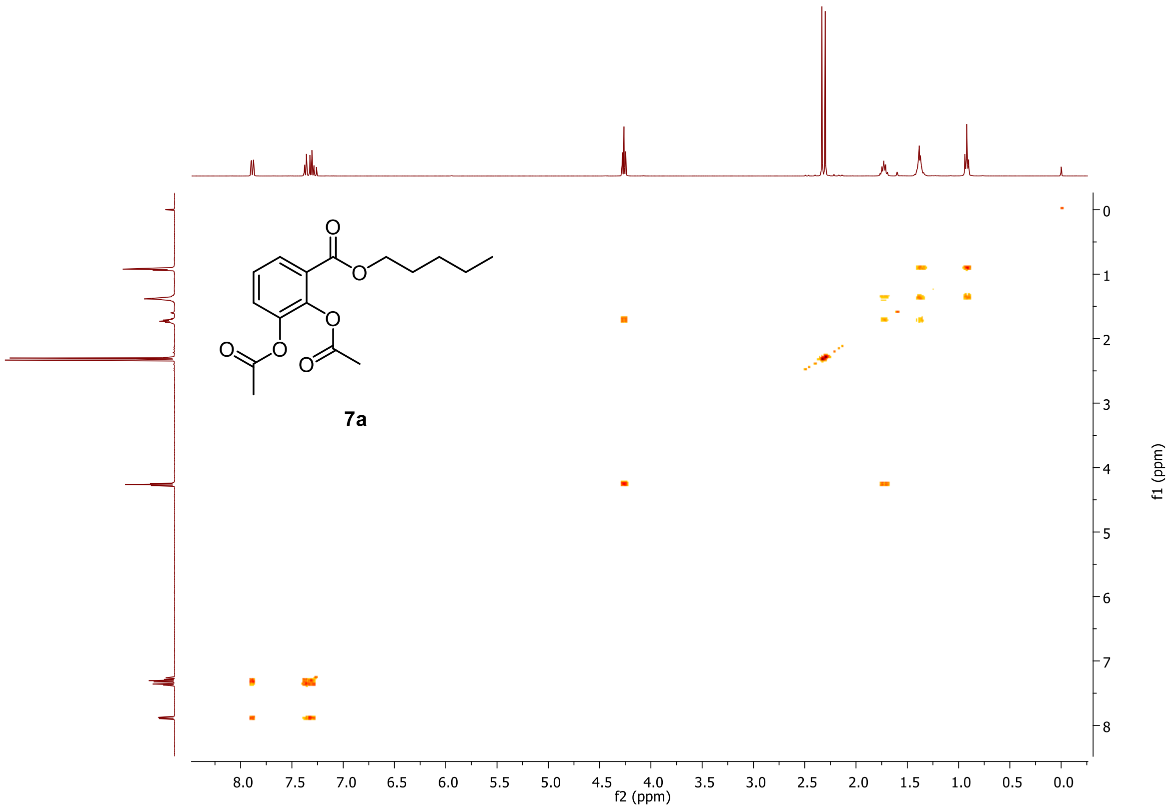

Figure S51: ^{1}H COSY NMR (400 MHz, $CDCl_3$) spectrum of pentyl 2,3-bis(acetyloxy)benzoate (7a) [DDV-AG-010].

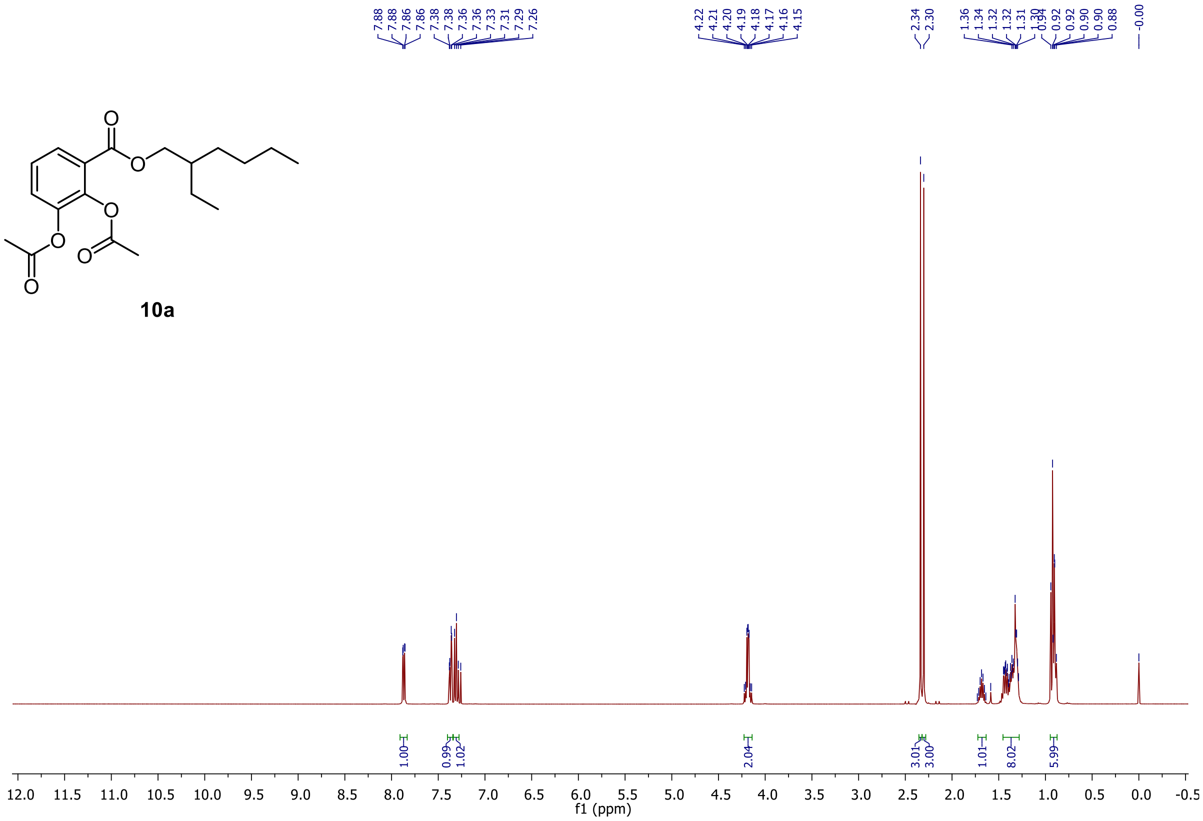

Figure S52: ^{1}H NMR (400 MHz, CDCl$_3$) spectrum of 2-ethylhexyl 2,3-bis(acetyloxy)benzoate (10a) [DDV-AG-016].

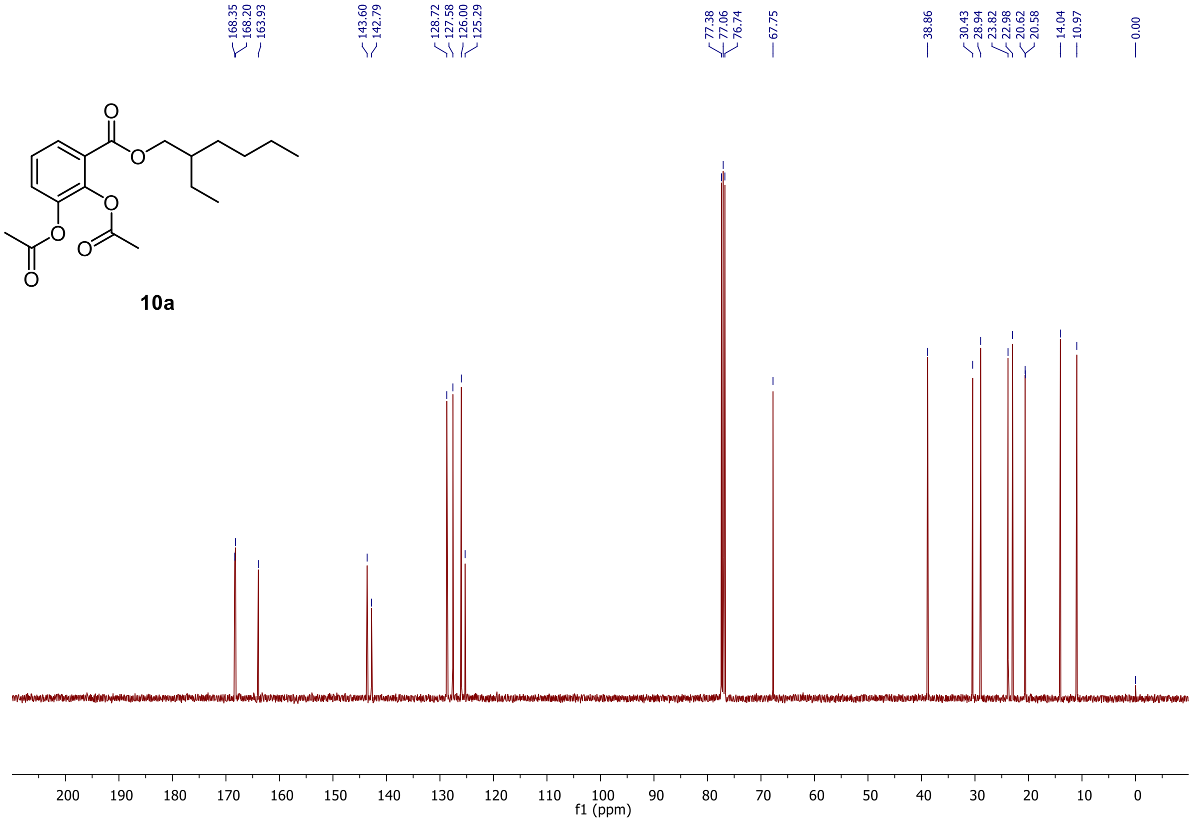

Figure S53: ^{13}C{^{1}H} NMR (101 MHz, CDCl$_3$) spectrum of 2-ethylhexyl 2,3-bis(acetyloxy)benzoate (10a) [DDV-AG-016].

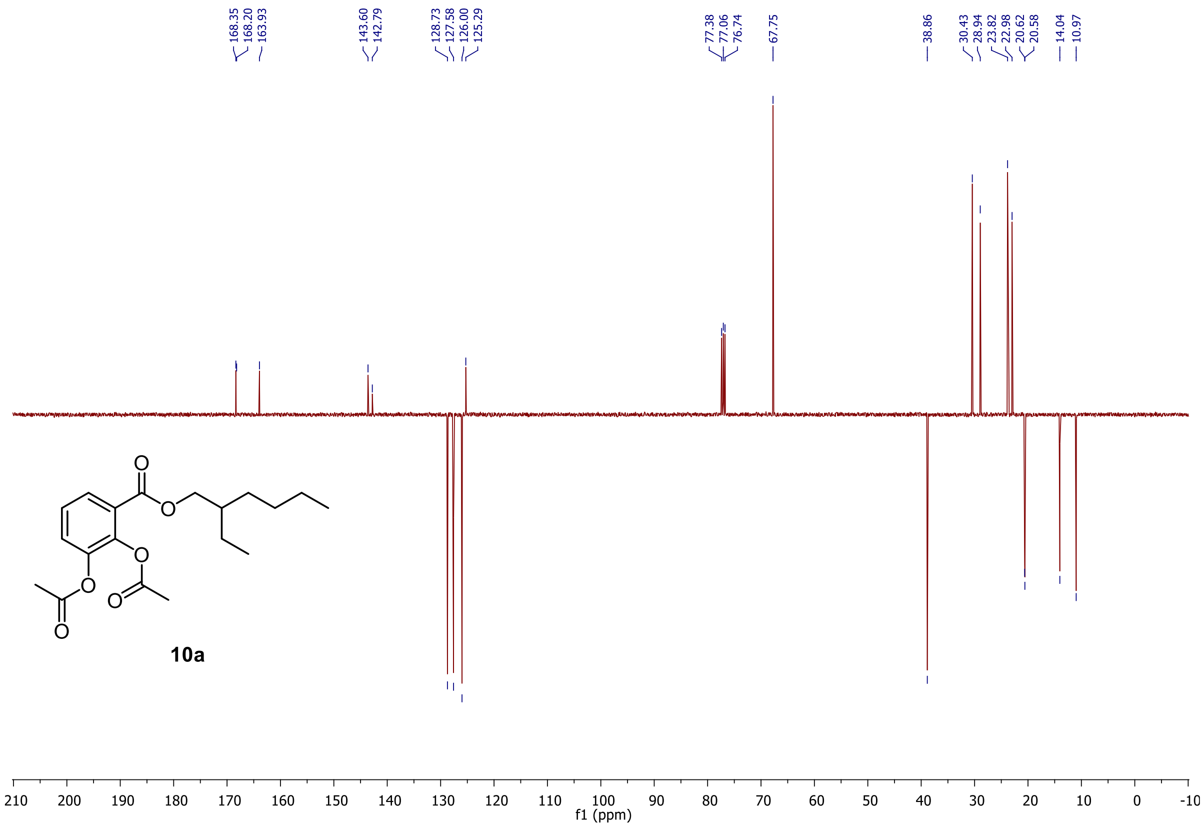

Figure S54: ^{13}C APT NMR (101 MHz, $CDCl_3$) spectrum of 2-ethylhexyl 2,3-bis(acetyloxy)benzoate (10a) [DDV-AG-016].

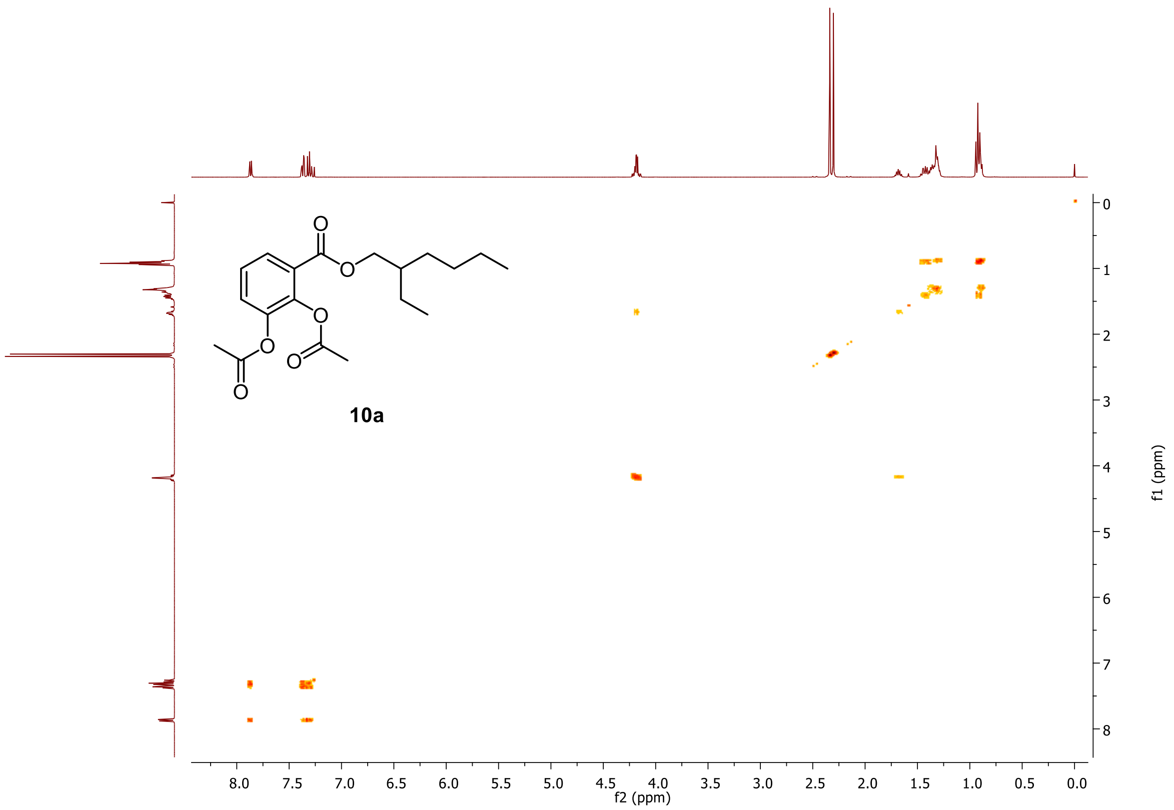

Figure S55: ^{1}H COSY NMR (400 MHz, $CDCl_3$) spectrum of 2-ethylhexyl 2,3-bis(acetyloxy)benzoate (10a) [DDV-AG-016].

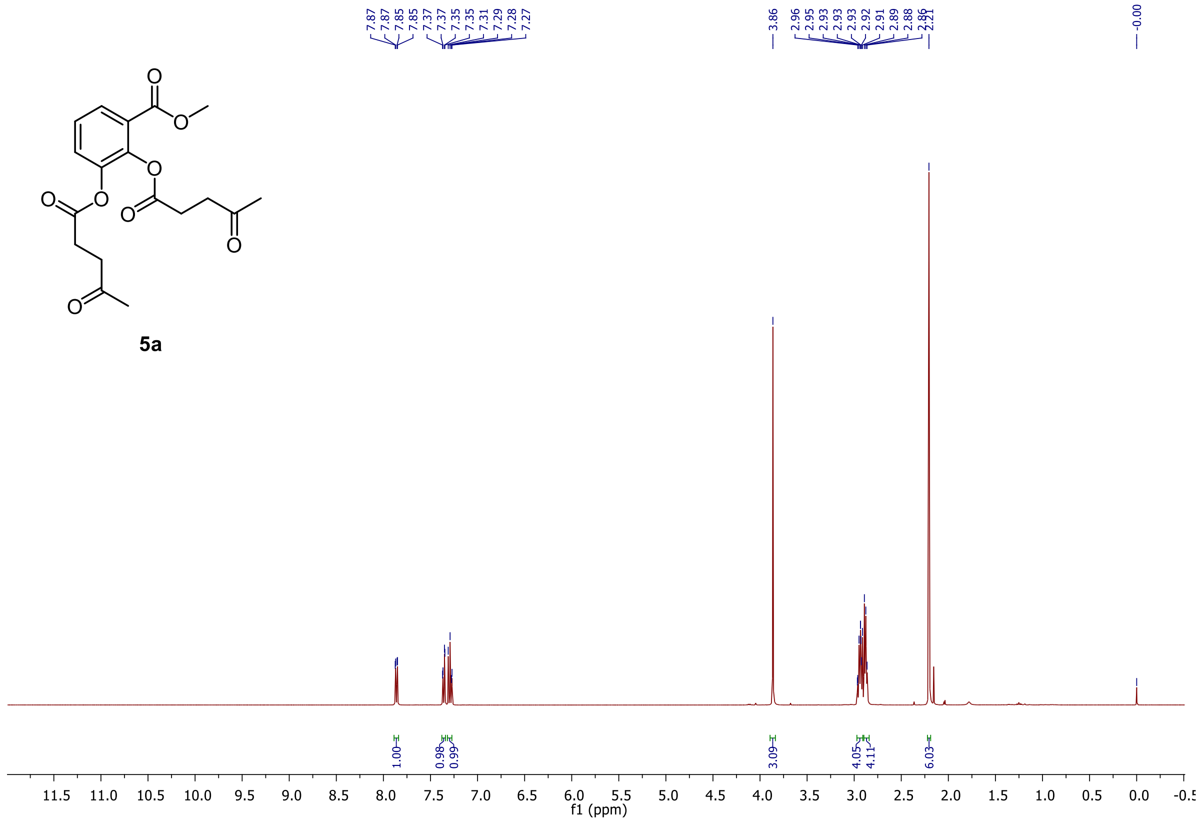

Figure S56: ^{1}H NMR (400 MHz, CDCl$_3$) spectrum of methyl 2,3-bis[(4-oxopentanoyl)oxy]benzoate (5a) [DDV-AG-032].

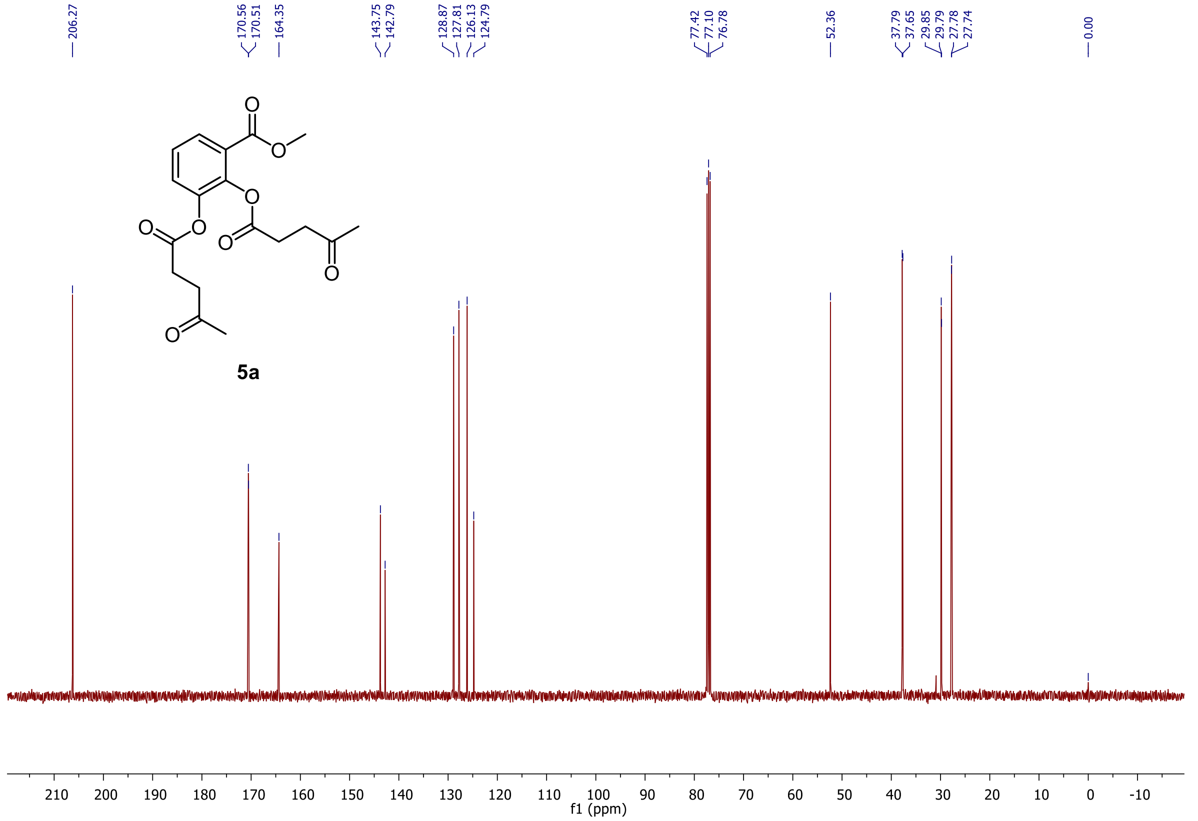

Figure S57: ^{13}C{^{1}H} NMR (101 MHz, CDCl$_3$) spectrum of methyl 2,3-bis[(4-oxopentanoyl)oxy]benzoate (5a) [DDV-AG-032].

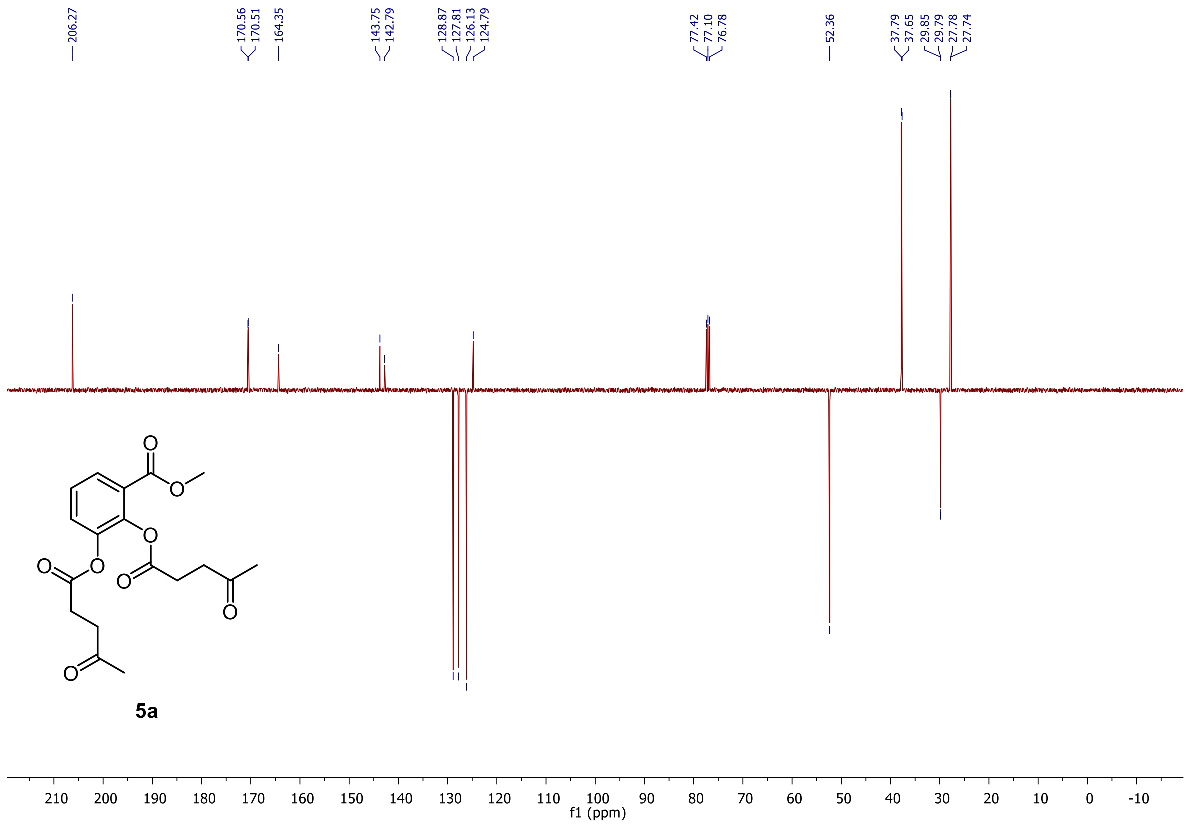

Figure S58: ^{13}C APT NMR (101 MHz, CDCl$_3$) spectrum of methyl 2,3-bis[(4-oxopentanoyl)oxy]benzoate (5a) [DDV-AG-032].

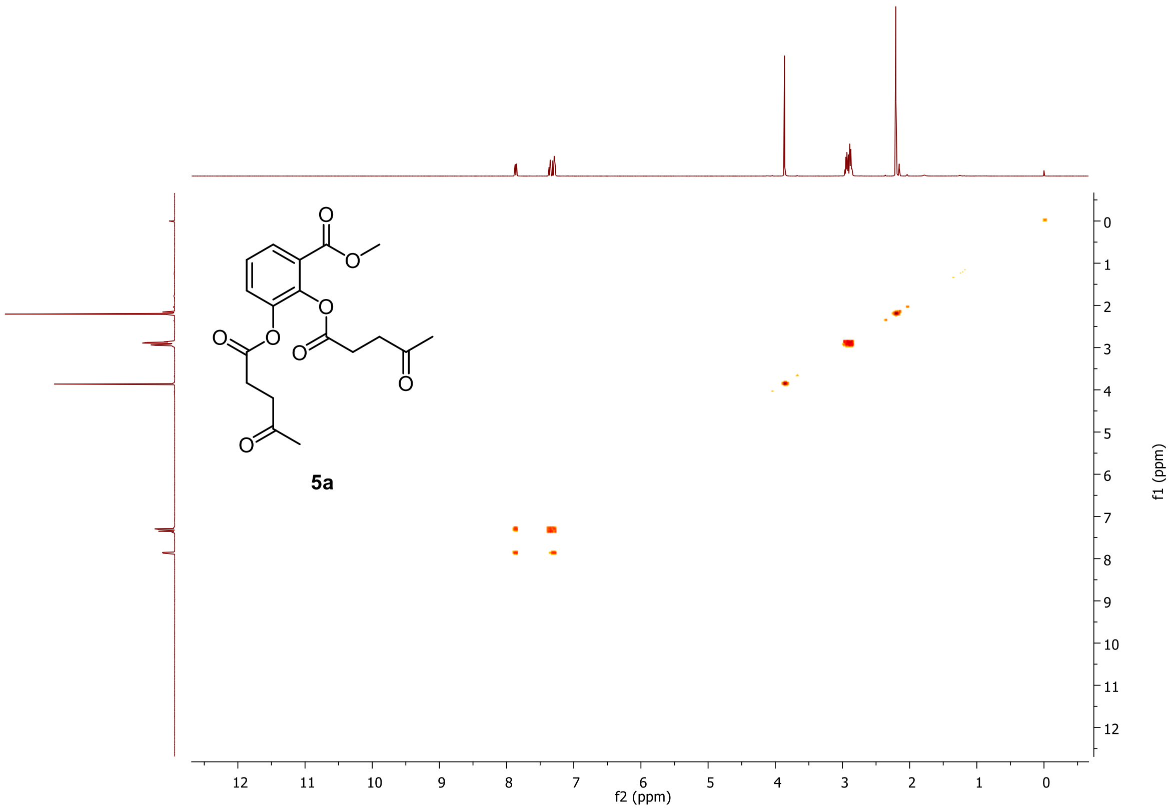

Figure S59: ^{1}H COSY NMR (400 MHz, CDCl$_3$) spectrum of methyl 2,3-bis[(4-oxopentanoyl)oxy]benzoate (5a) [DDV-AG-032].

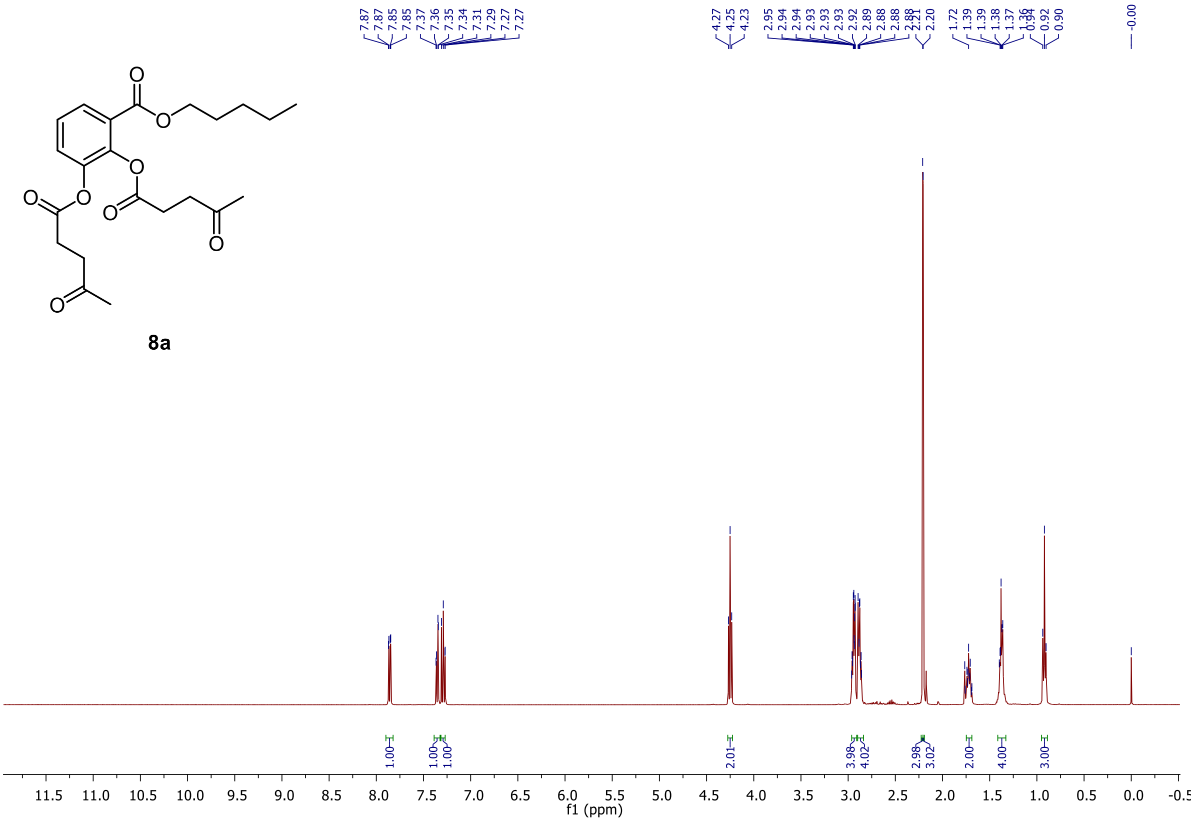

Figure S60: ^{1}H NMR (400 MHz, $CDCl_3$) spectrum of pentyl 2,3-bis[(4-oxopentanoyl)oxy]benzoate (8a) [DDV-AG-012].

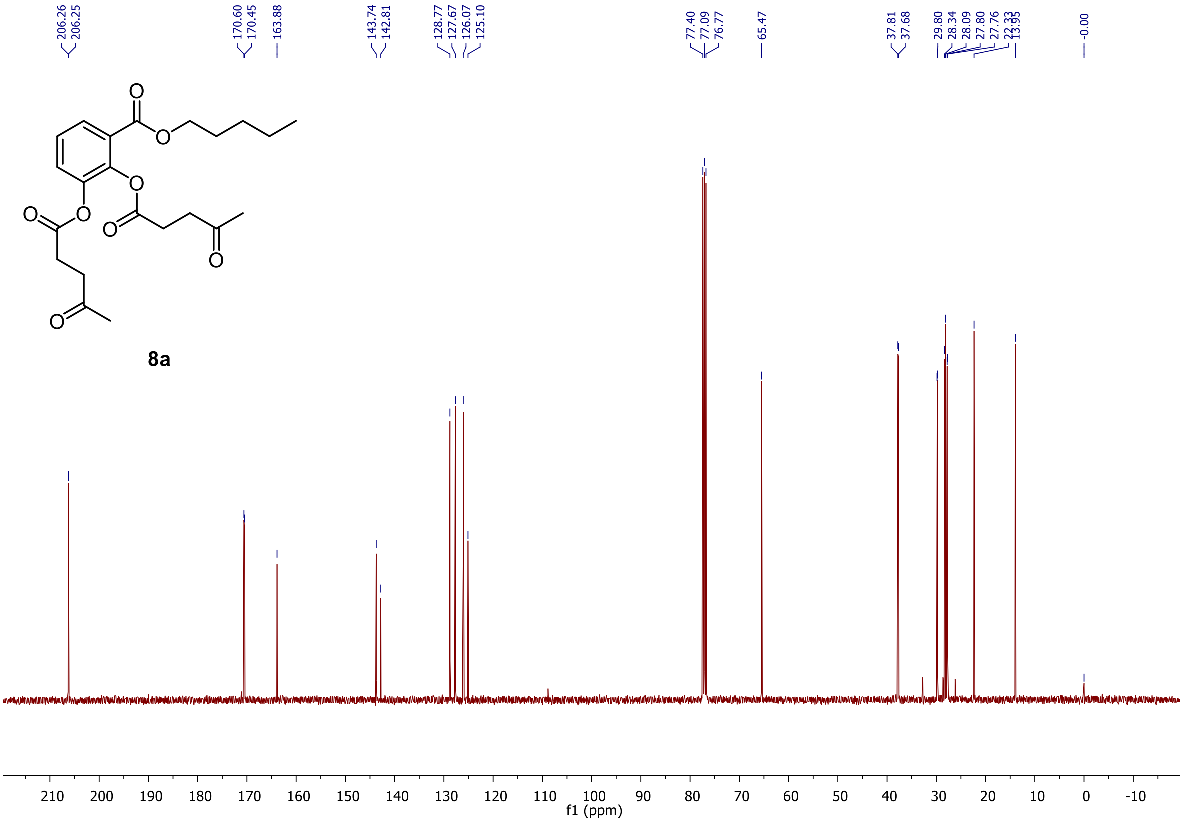

Figure S61: $^{13}C\{^{1}H\}$ NMR (101 MHz, $CDCl_3$) spectrum of pentyl 2,3-bis[(4-oxopentanoyl)oxy]benzoate (8a) [DDV-AG-012].

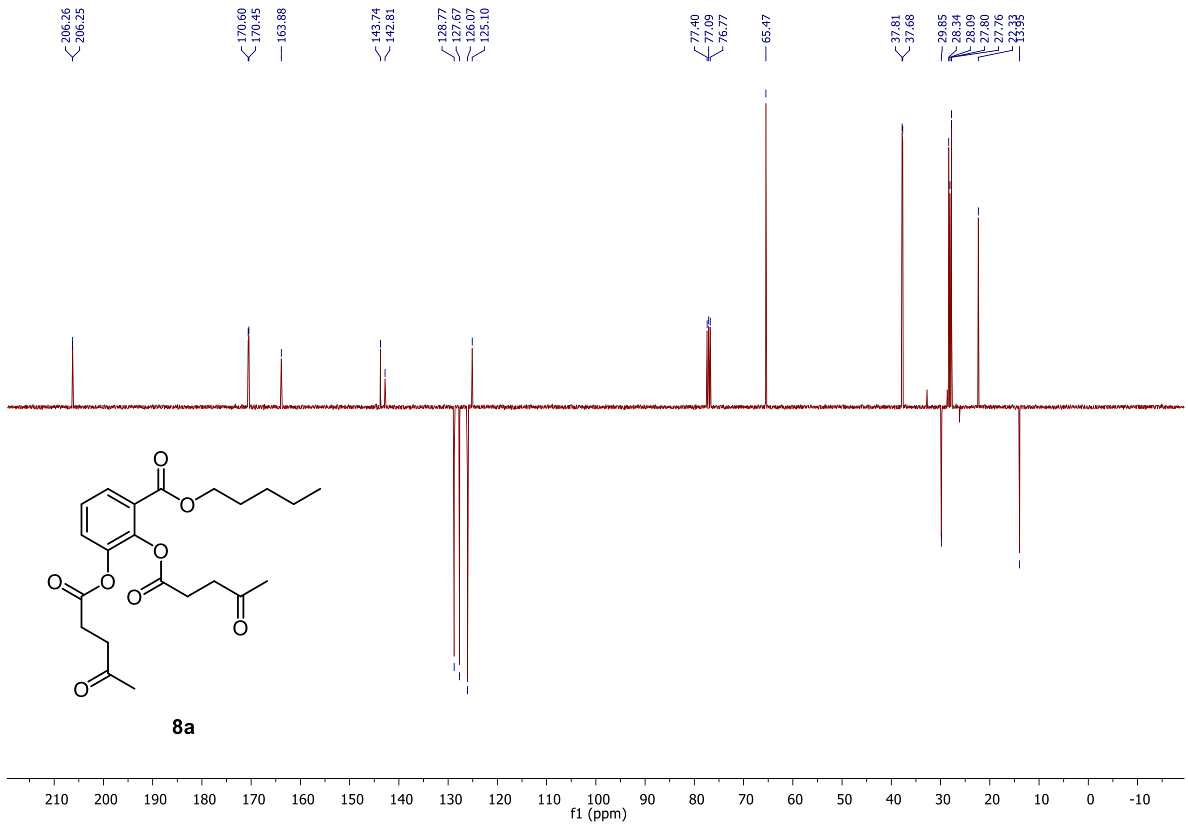

Figure S62: ^{13}C APT NMR (101 MHz, $CDCl_3$) spectrum of pentyl 2,3-bis[(4-oxopentanoyl)oxy]benzoate (8a) [DDV-AG-012].

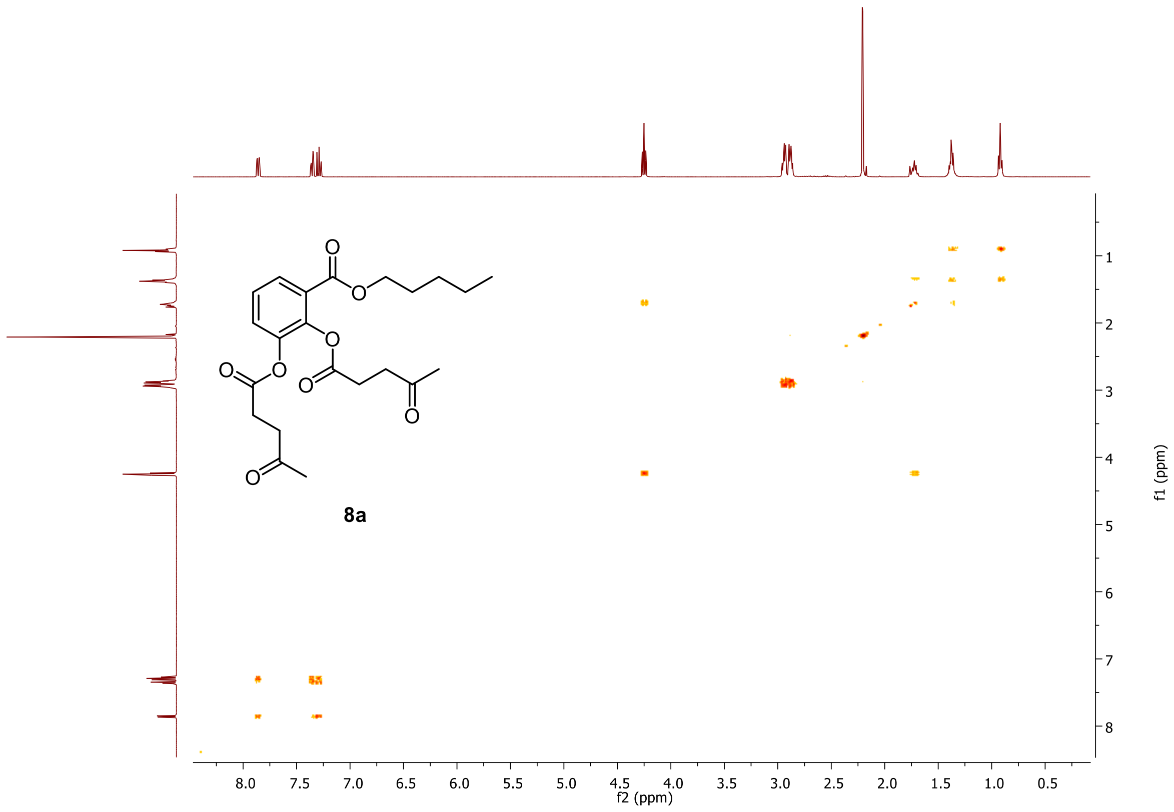

Figure S63: ^{1}H COSY NMR (400 MHz, $CDCl_3$) spectrum of pentyl 2,3-bis[(4-oxopentanoyl)oxy]benzoate (8a) [DDV-AG-012].

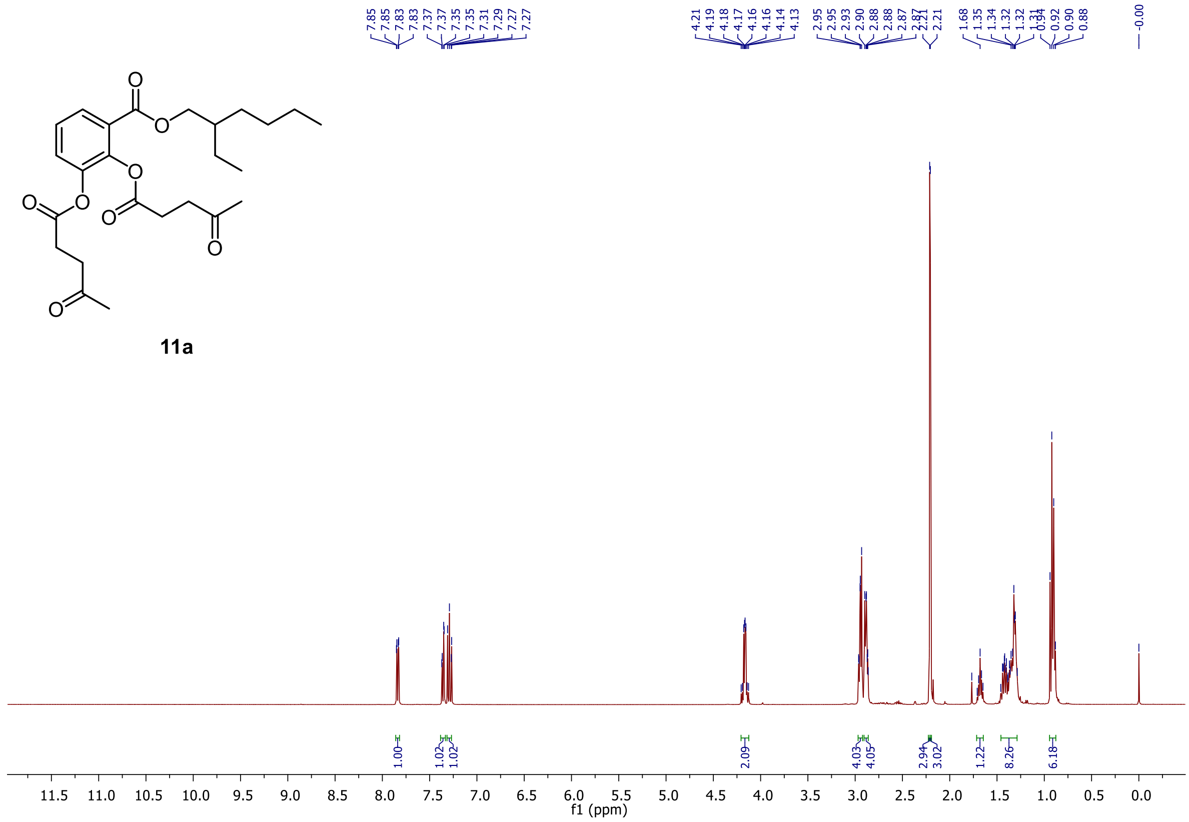

Figure S64: ^{1}H NMR (400 MHz, $CDCl_3$) spectrum of 2-ethylhexyl 2,3-bis[(4-oxopentanoyl)oxy]benzoate (11a) [DDV-AG-020].

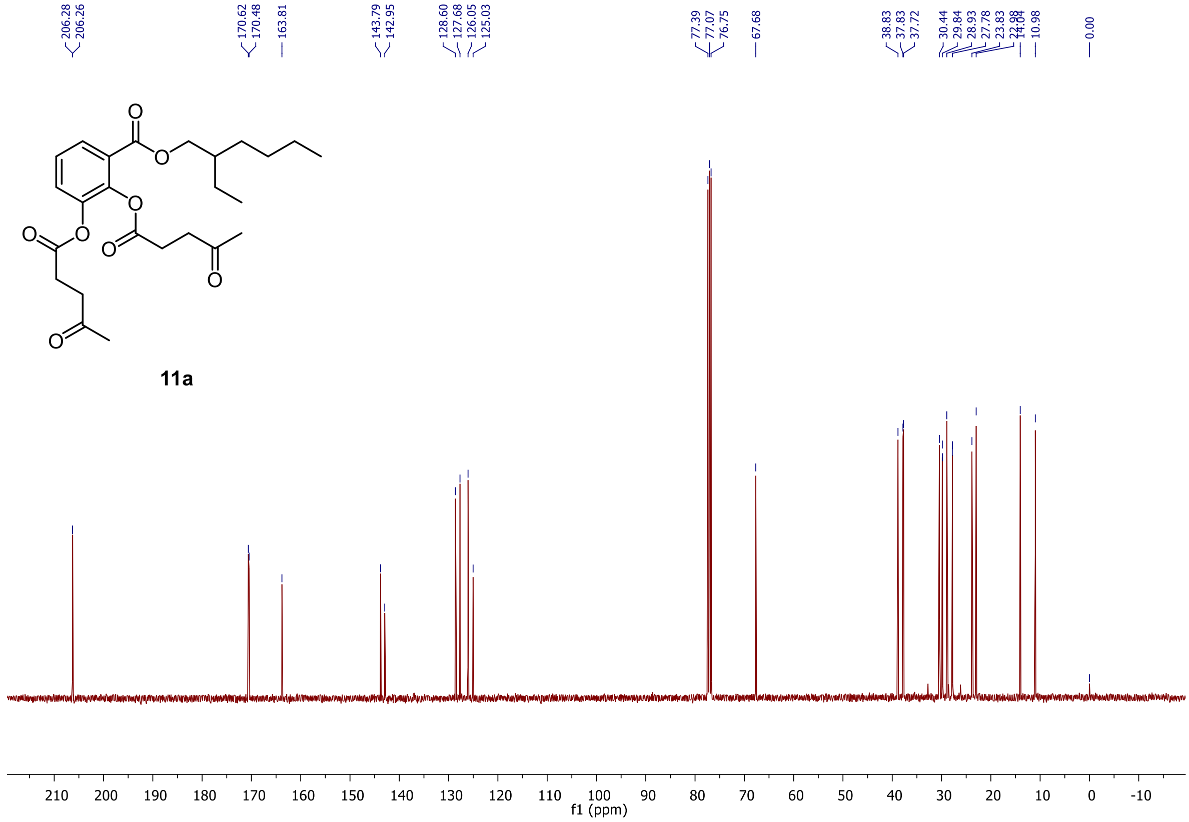

Figure S65: ^{13}C{^{1}H} NMR (101 MHz, $CDCl_3$) spectrum of 2-ethylhexyl 2,3-bis[(4-oxopentanoyl)oxy]benzoate (11a) [DDV-AG-020].

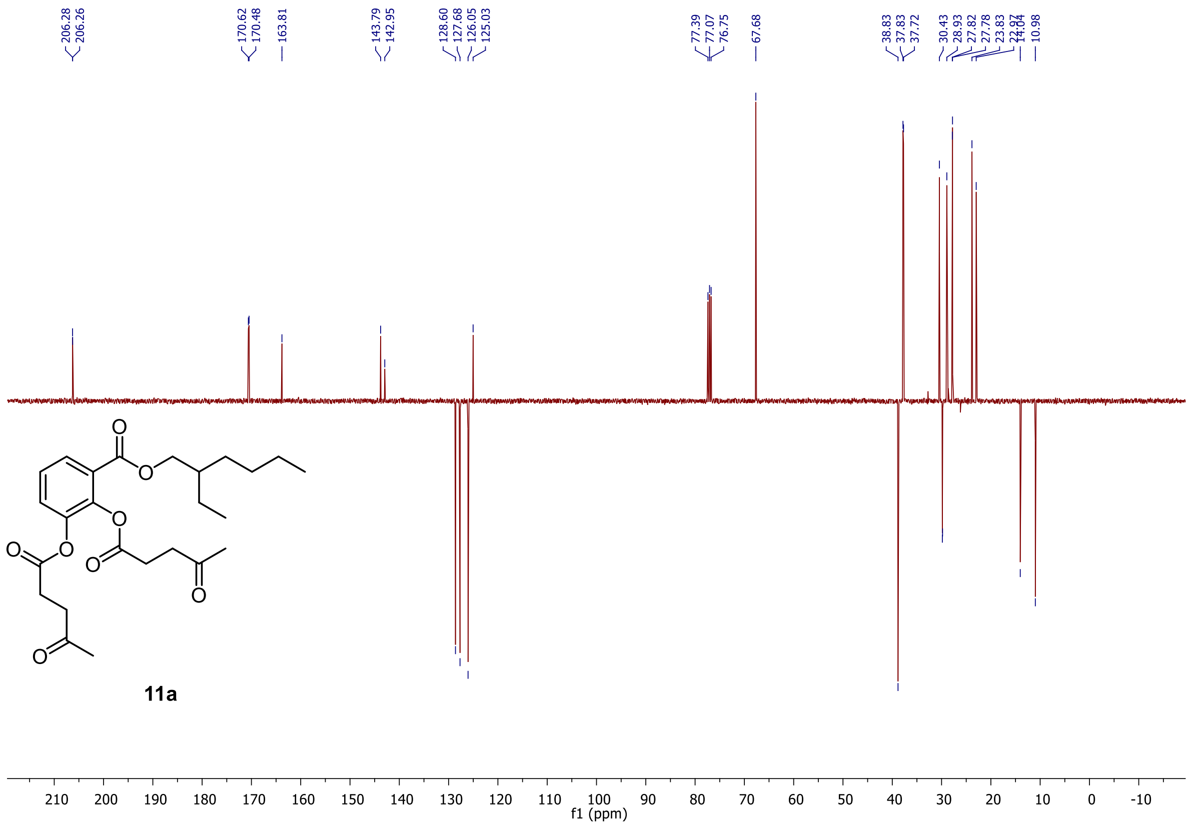

Figure S66: ^{13}C APT NMR (101 MHz, $CDCl_3$) spectrum of 2-ethylhexyl 2,3-bis[(4-oxopentanoyl)oxy] benzoate (11a) [DDV-AG-020].

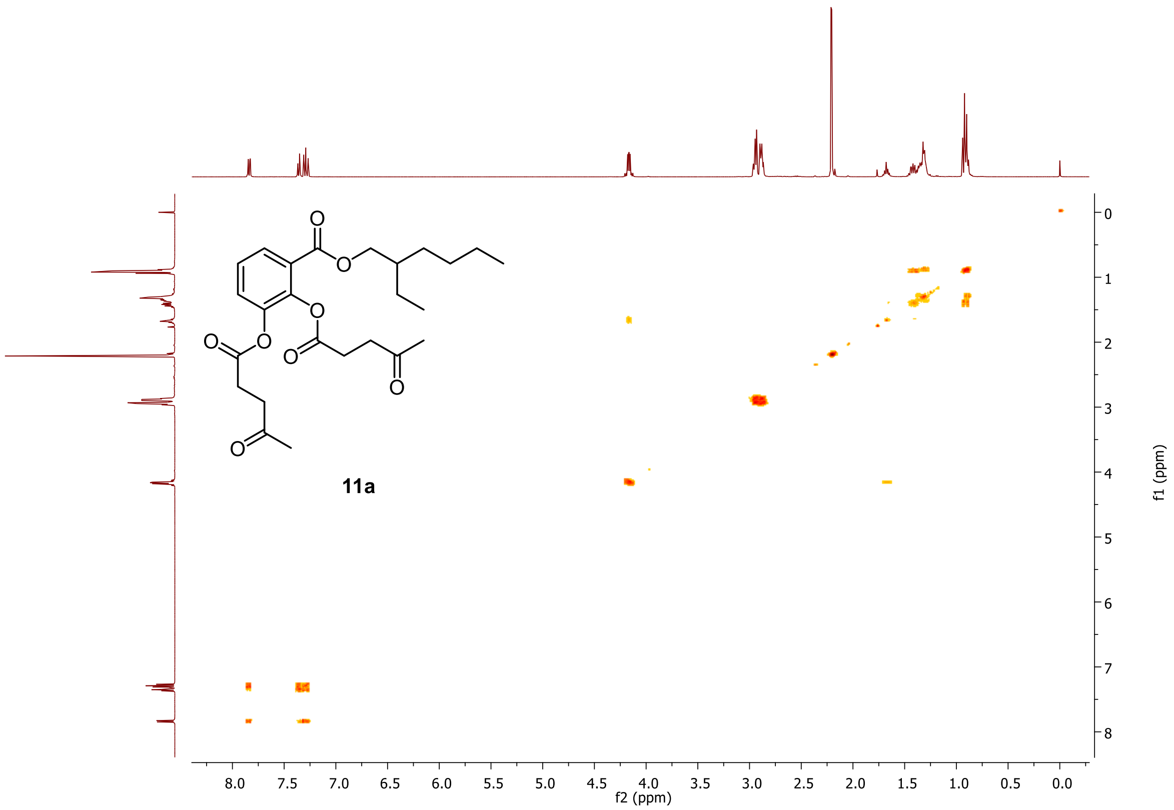

Figure S67: ^{1}H COSY NMR (400 MHz, $CDCl_3$) spectrum of 2-ethylhexyl 2,3-bis[(4-oxopentanoyl)oxy] benzoate (11a) [DDV-AG-020].

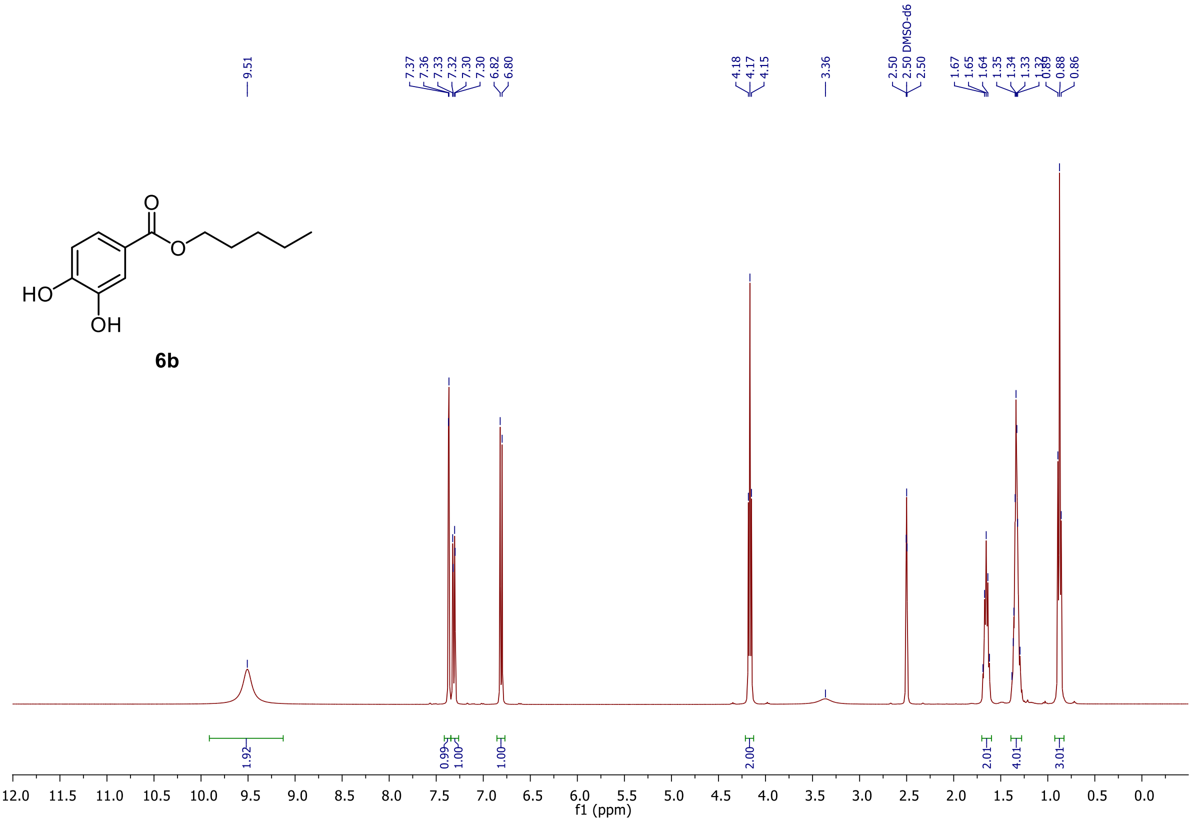

Figure S68: ^{1}H NMR (400 MHz, DMSO-d_6) spectrum of pentyl 3,4-dihydroxybenzoate (6b) [DDV-AG-007].

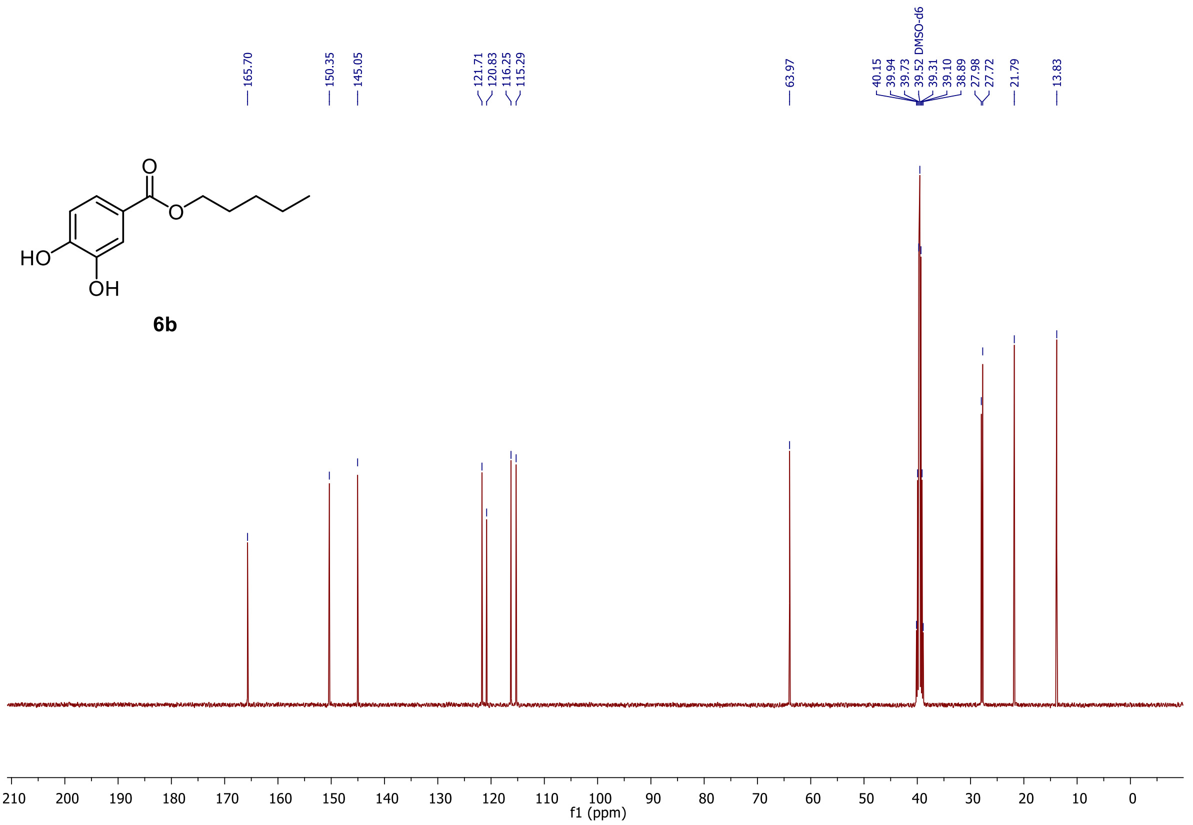

Figure S69: $^{13}C\{^{1}H\}$ NMR (101 MHz, DMSO-d_6) spectrum of pentyl 3,4-dihydroxybenzoate (6b) [DDV-AG-007].

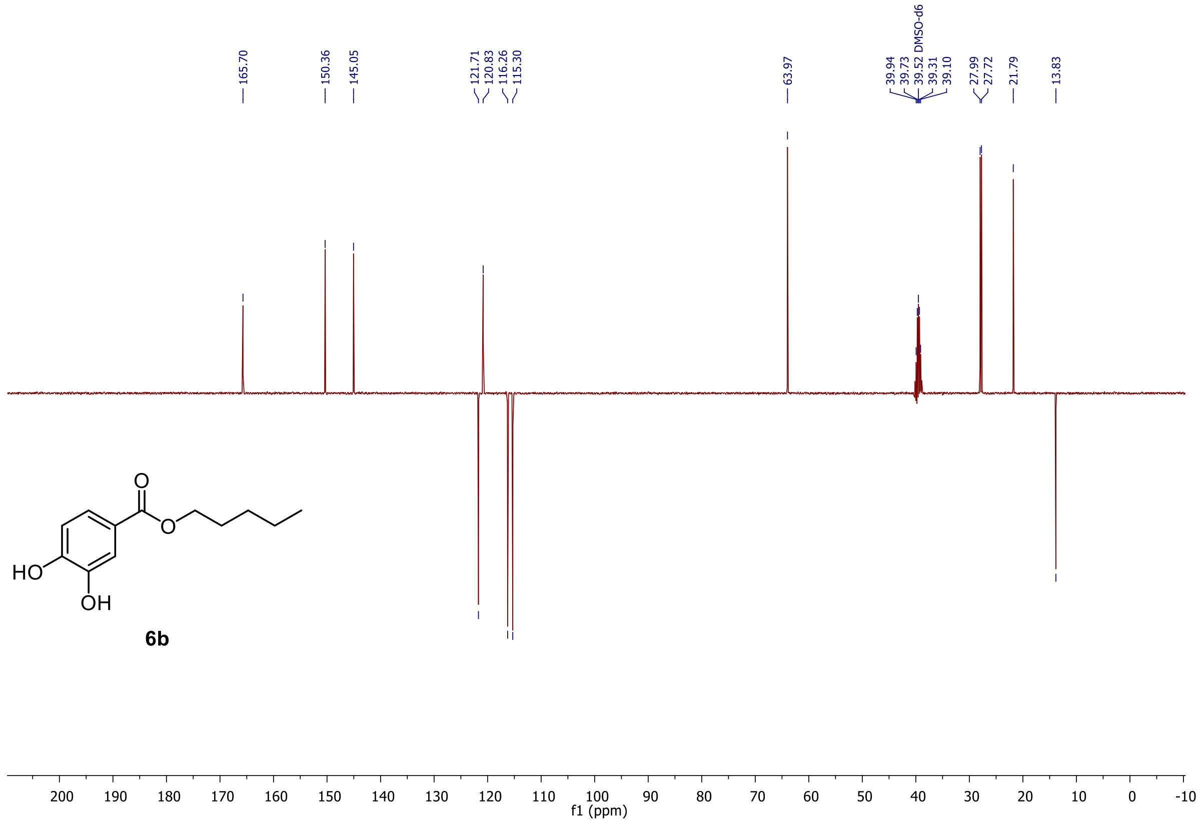

Figure S70: ^{13}C APT NMR (101 MHz, DMSO-d_6) spectrum of pentyl 3,4-dihydroxybenzoate (6b) [DDV-AG-007].

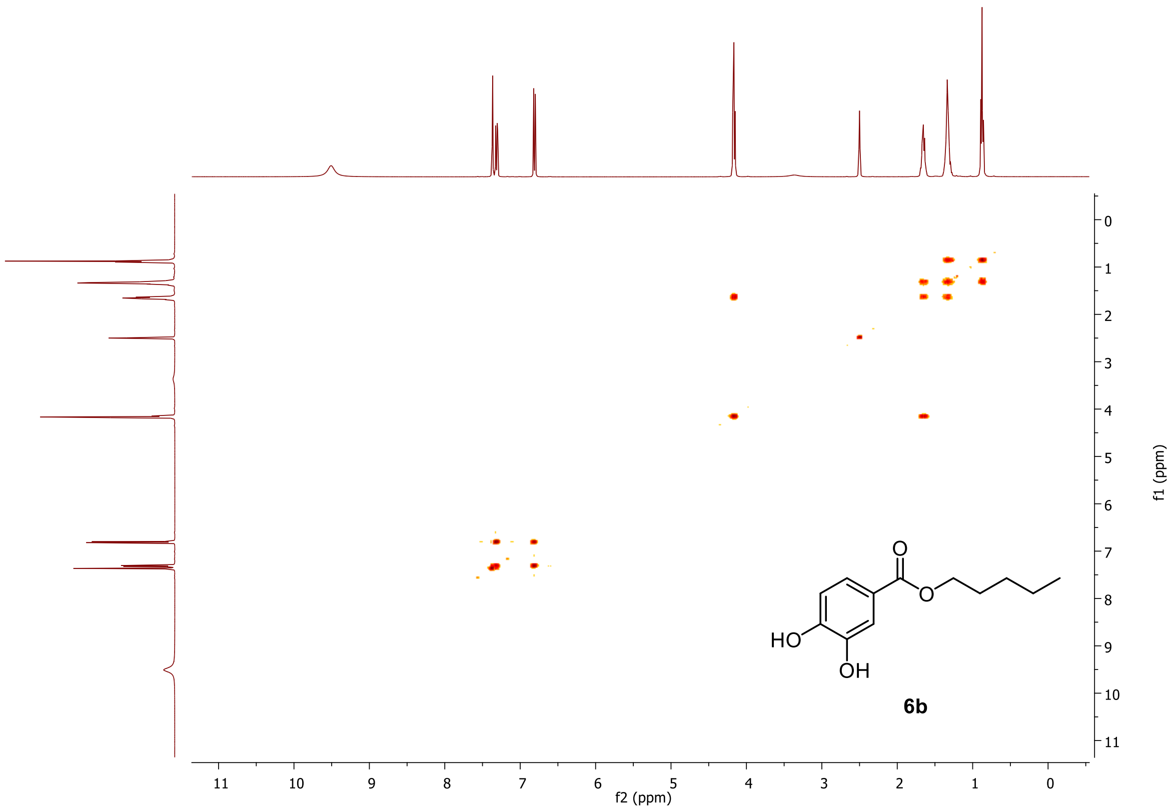

Figure S71: ^{1}H COSY NMR (400 MHz, DMSO-d_6) spectrum of pentyl 3,4-dihydroxybenzoate (6b) [DDV-AG-007].

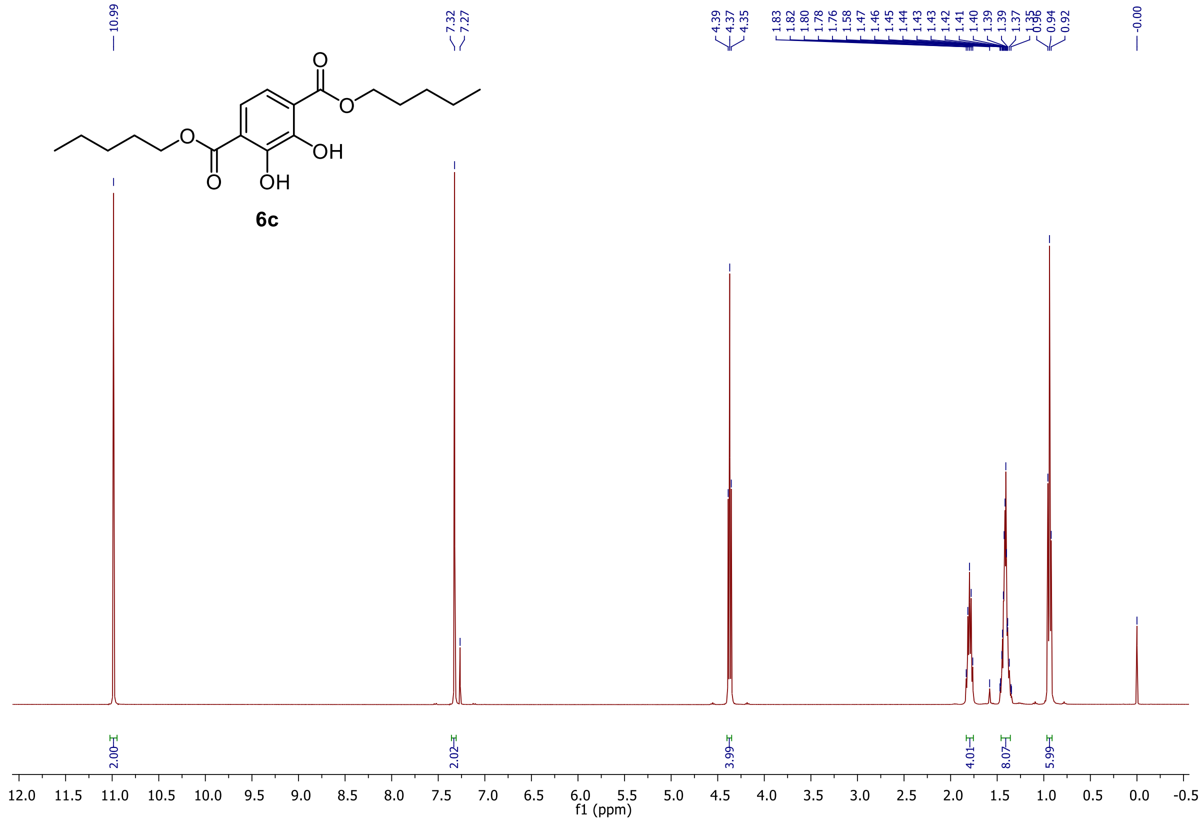

Figure S72: ^{1}H NMR (400 MHz, $CDCl_3$) spectrum of dipentyl 2,3-dihydroxybenzene-1,4-dicarboxylate (6c) [DDV-AG-015].

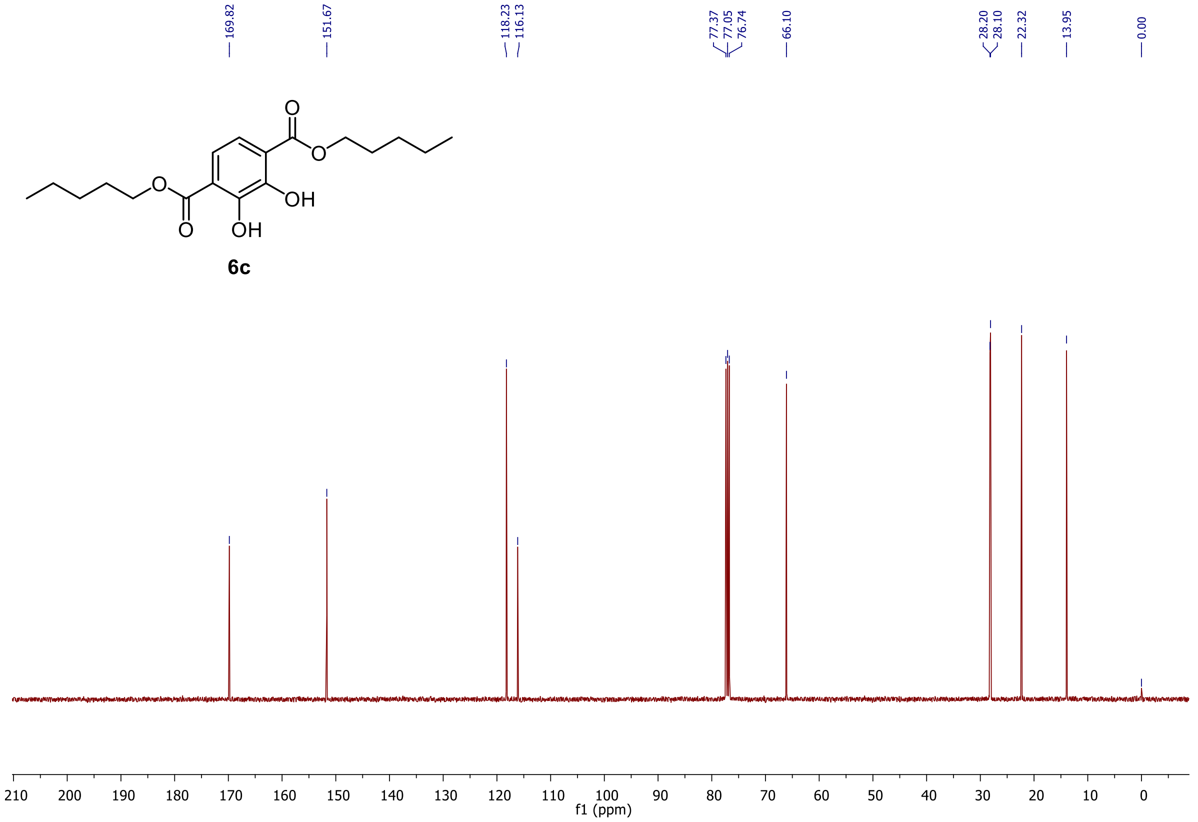

Figure S73: $^{13}C\{^{1}H\}$ NMR (101 MHz, $CDCl_3$) spectrum of dipentyl 2,3-dihydroxybenzene-1,4-dicarboxylate (6c) [DDV-AG-015].

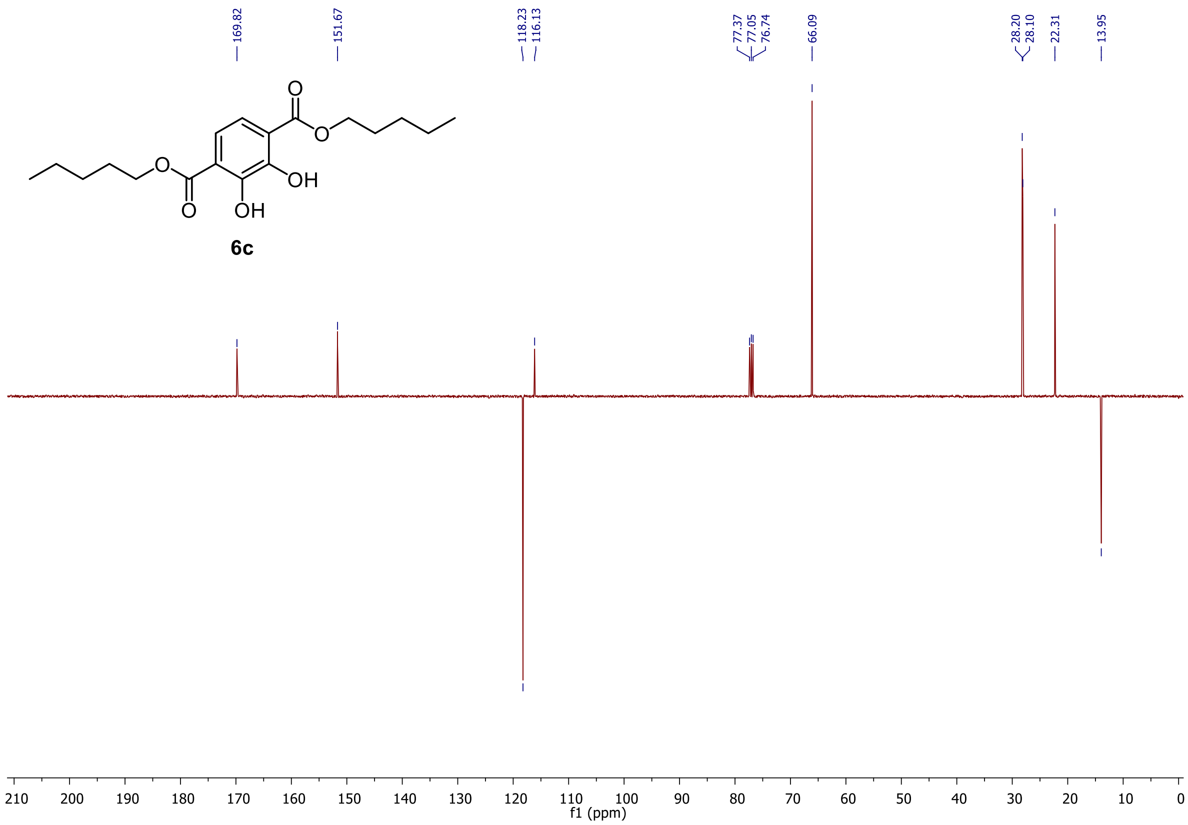

Figure S74: ^{13}C APT NMR (101 MHz, CDCl$_3$) spectrum of dipentyl 2,3-dihydroxybenzene-1,4-dicarboxylate (6c) [DDV-AG-015].

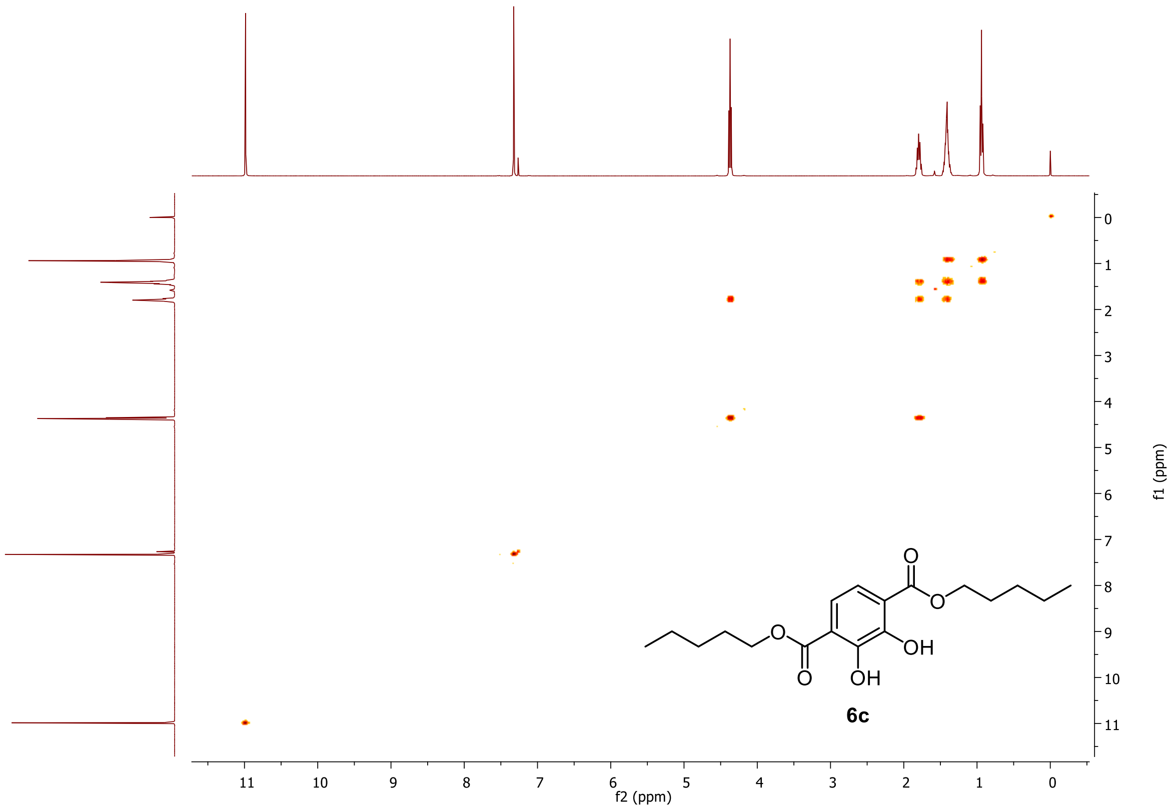

Figure S75: ^{1}H COSY NMR (400 MHz, CDCl$_3$) spectrum of dipentyl 2,3-dihydroxybenzene-1,4-dicarboxylate (6c) [DDV-AG-015].

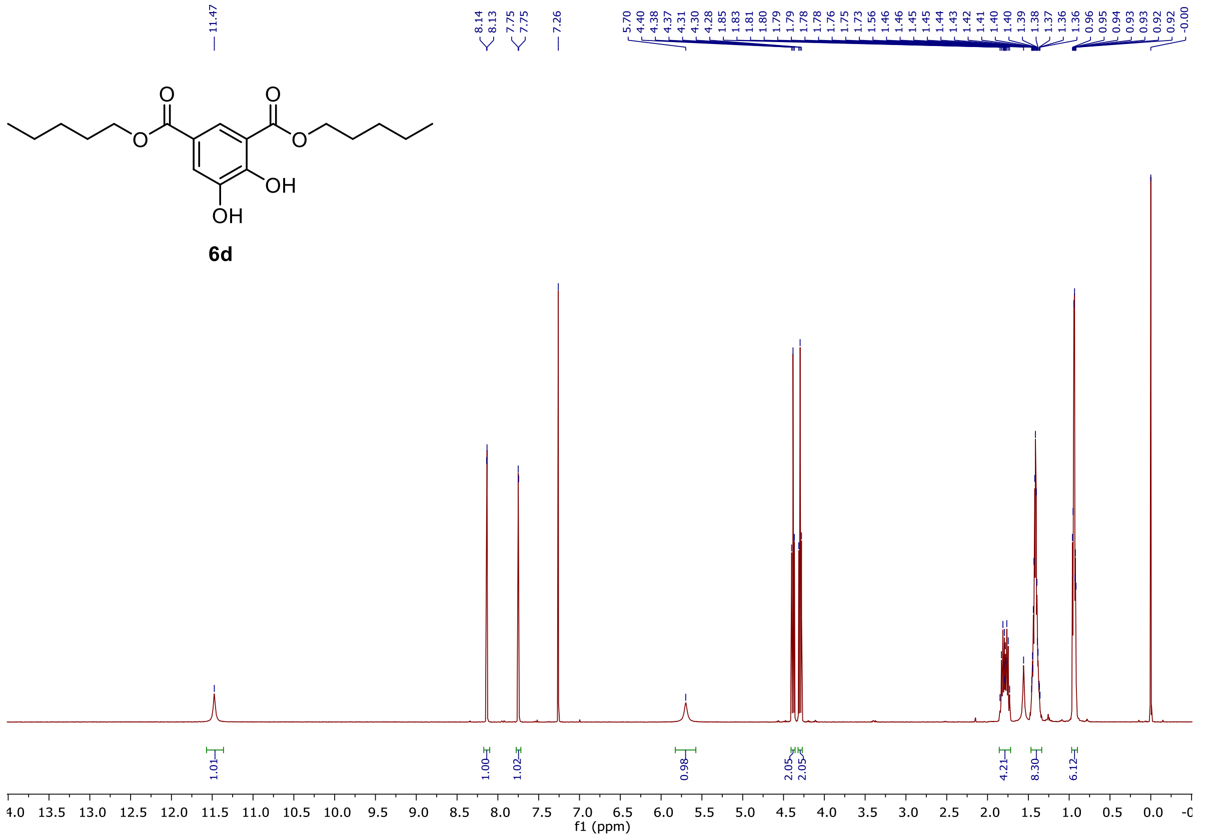

Figure S76: ^{1}H NMR (400 MHz, $CDCl_3$) spectrum of bis(2-ethylhexyl) 4,5-dihydroxybenzene-1,3-dicarboxylate (6d) [AGO-132-133-134].

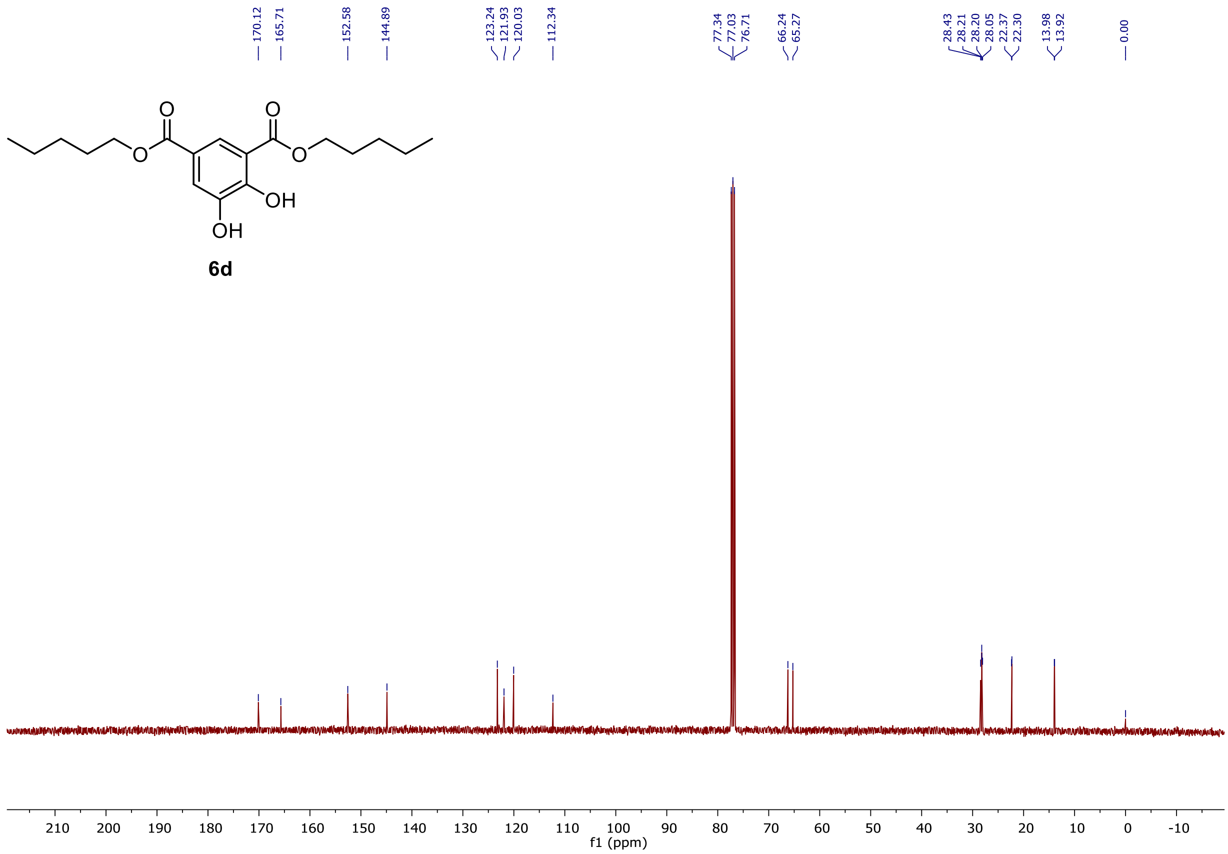

Figure S77: ^{13}C{^{1}H} NMR (101 MHz, $CDCl_3$) spectrum of bis(2-ethylhexyl) 4,5-dihydroxybenzene-1,3-dicarboxylate (6d) [AGO-132-133-134].

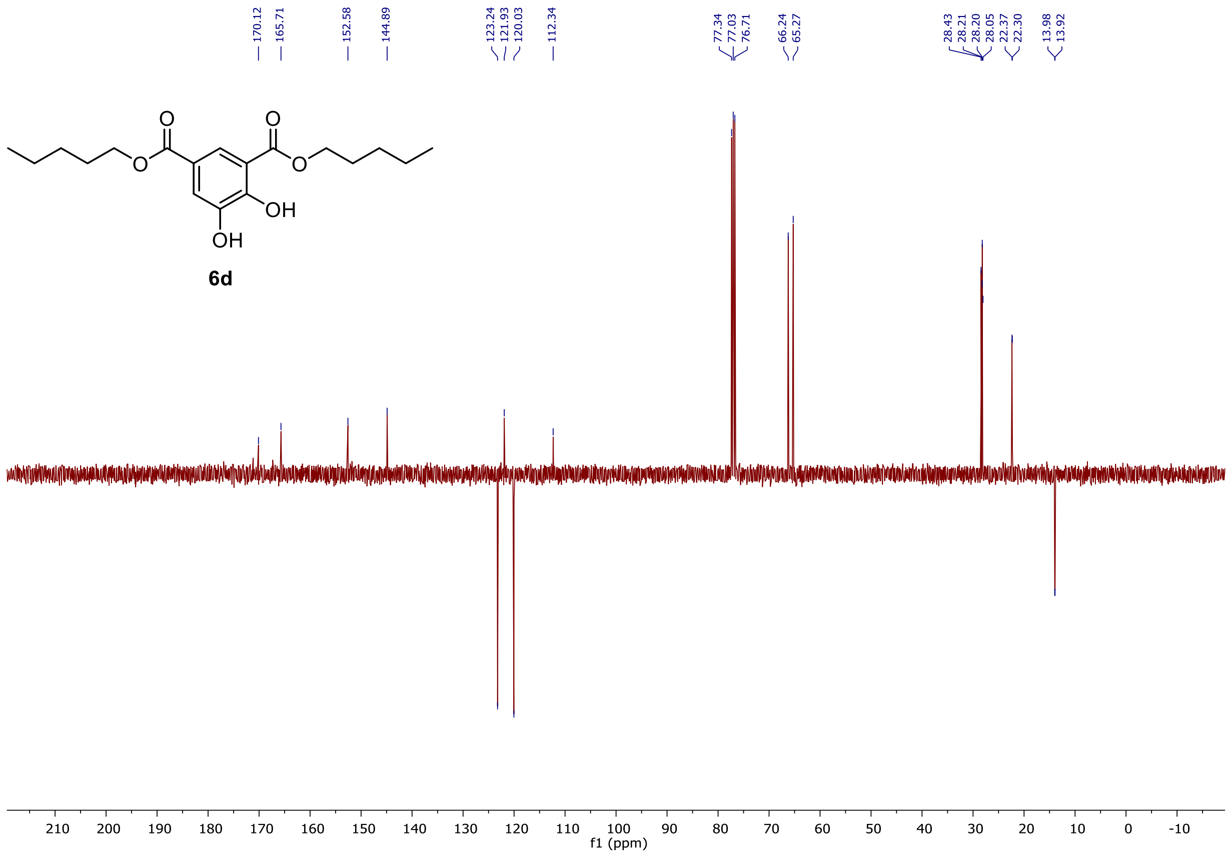

Figure S78: ^{13}C APT NMR (101 MHz, $CDCl_3$) spectrum of bis(2-ethylhexyl) 4,5-dihydroxybenzene-1,3-dicarboxylate (6d) [AGO-132-133-134].

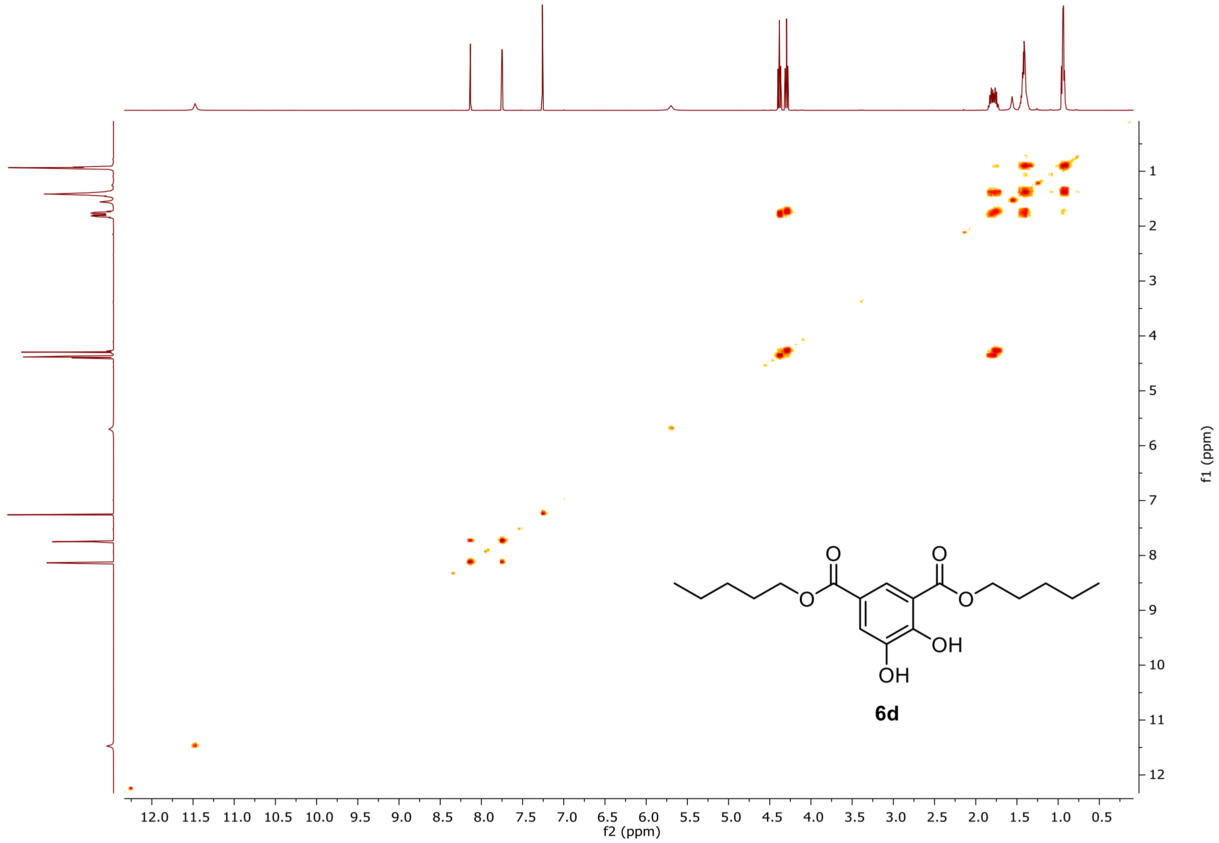

Figure S79: ^{1}H COSY NMR (400 MHz, $CDCl_3$) spectrum of bis(2-ethylhexyl) 4,5-dihydroxybenzene-1,3-dicarboxylate (6d) [AGO-132-133-134].

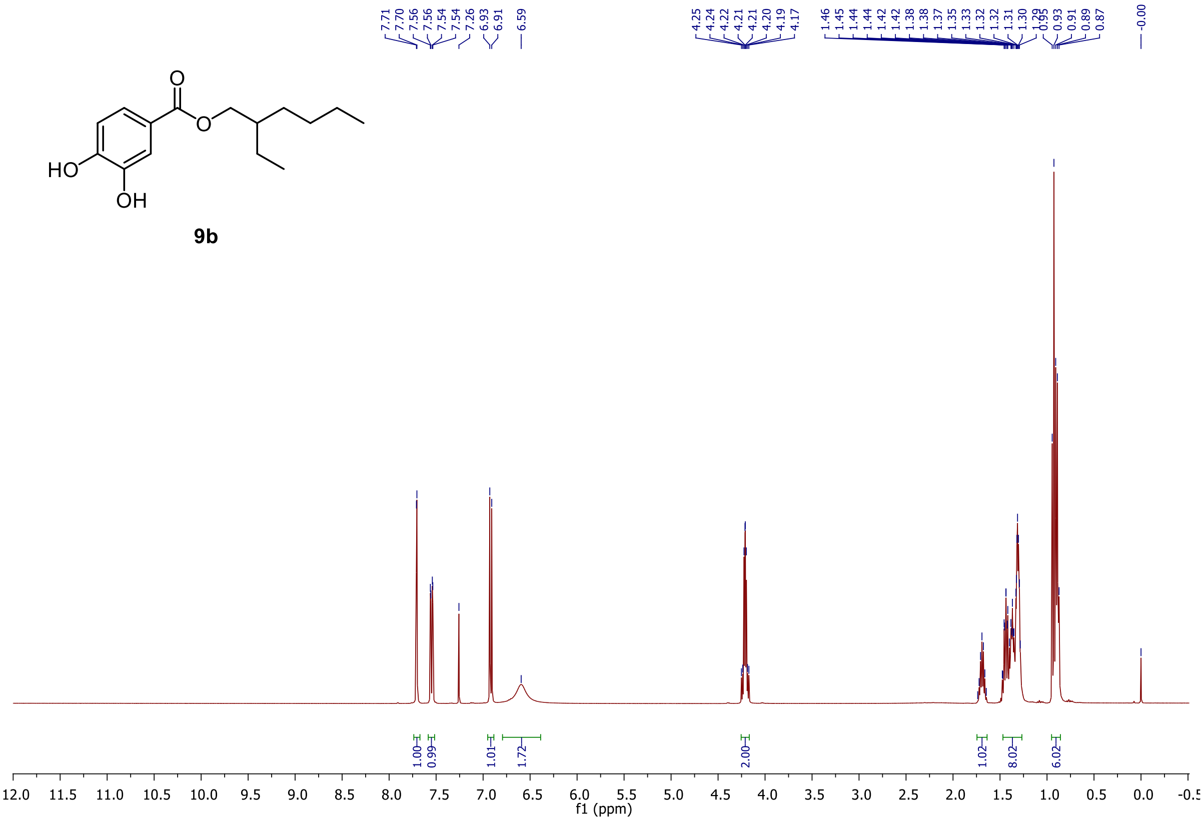

Figure S80: ^{1}H NMR (400 MHz, $CDCl_3$) spectrum of 2-ethylhexyl 3,4-dihydroxybenzoate (9b) [DDV-AG-009].

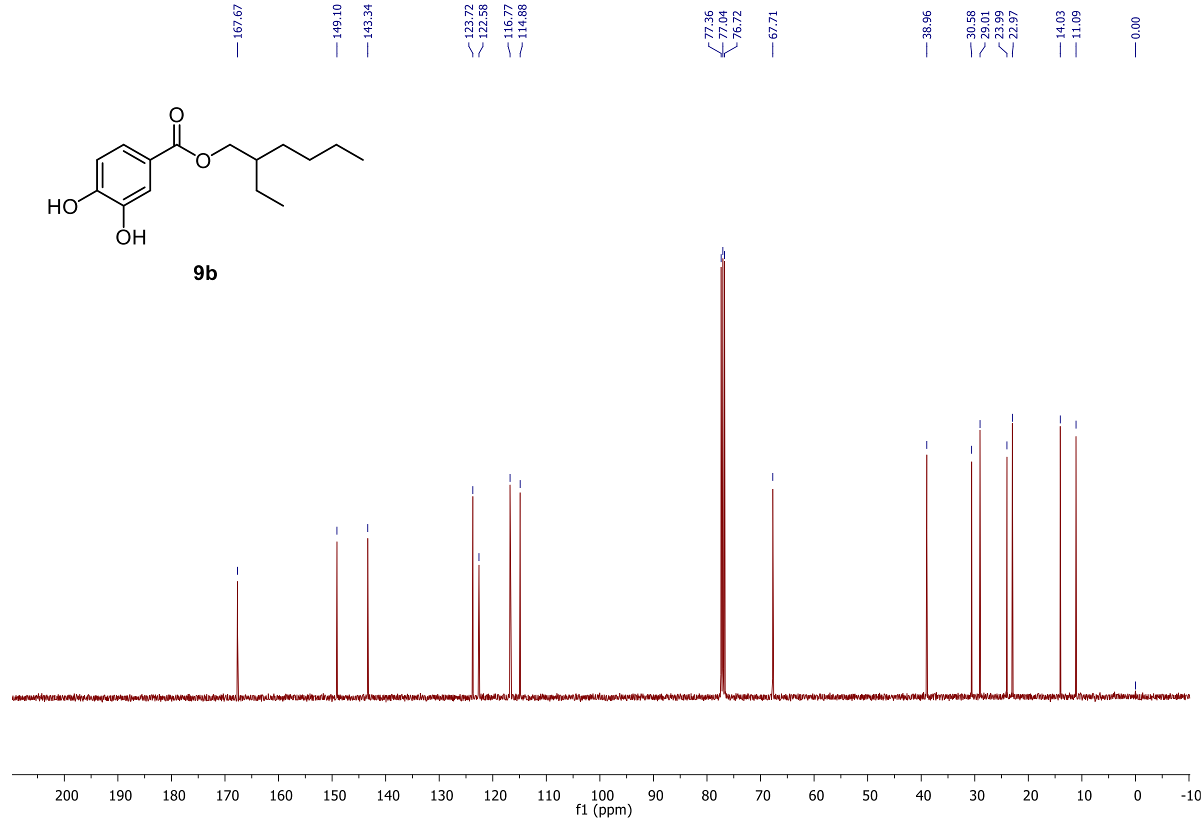

Figure S81: $^{13}C\{^{1}H\}$ NMR (101 MHz, $CDCl_3$) spectrum of 2-ethylhexyl 3,4-dihydroxybenzoate (9b) [DDV-AG-009].

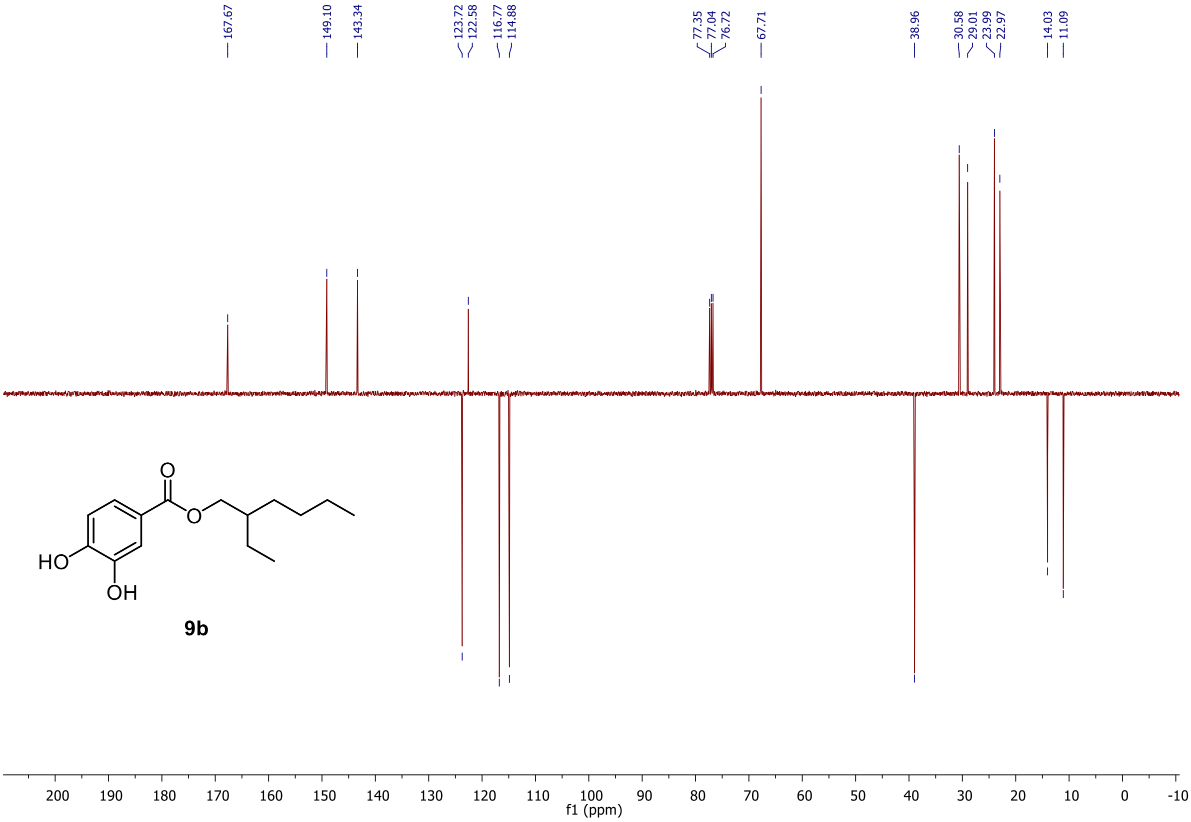

Figure S82: ^{13}C APT NMR (101 MHz, CDCl$_3$) spectrum of 2-ethylhexyl 3,4-dihydroxybenzoate (9b) [DDV-AG-009].

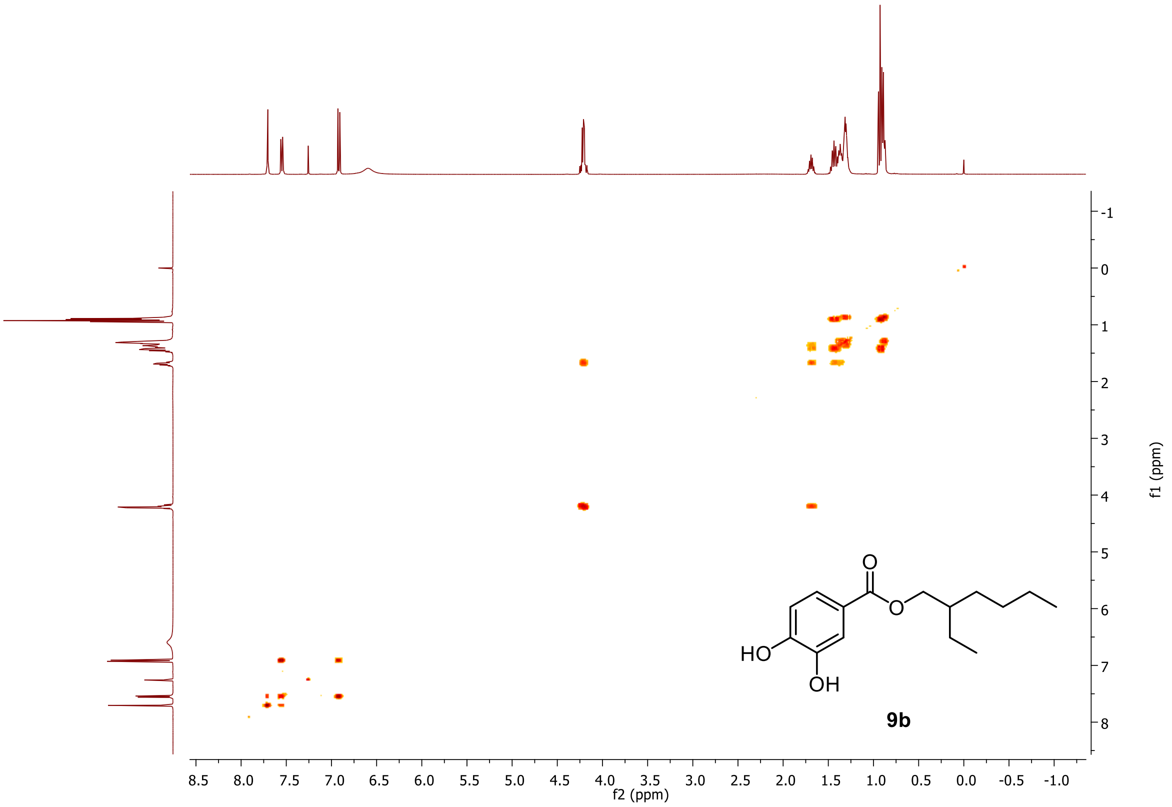

Figure S83: ^{1}H COSY NMR (400 MHz, CDCl$_3$) spectrum of 2-ethylhexyl 3,4-dihydroxybenzoate (9b) [DDV-AG-009].

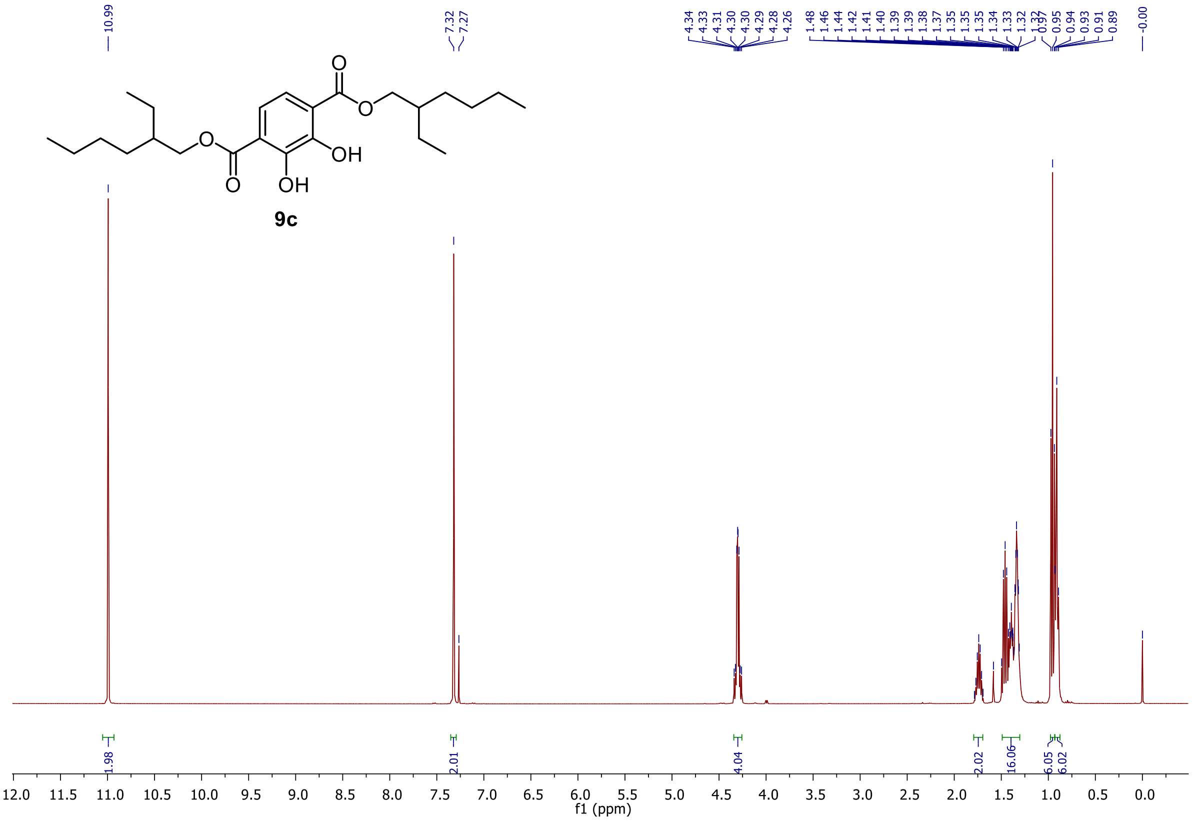

Figure S84: ^{1}H NMR (400 MHz, $CDCl_3$) spectrum of bis(2-ethylhexyl) 2,3-dihydroxybenzene-1,4-dicarboxylate (9c) [DDV-AG-018].

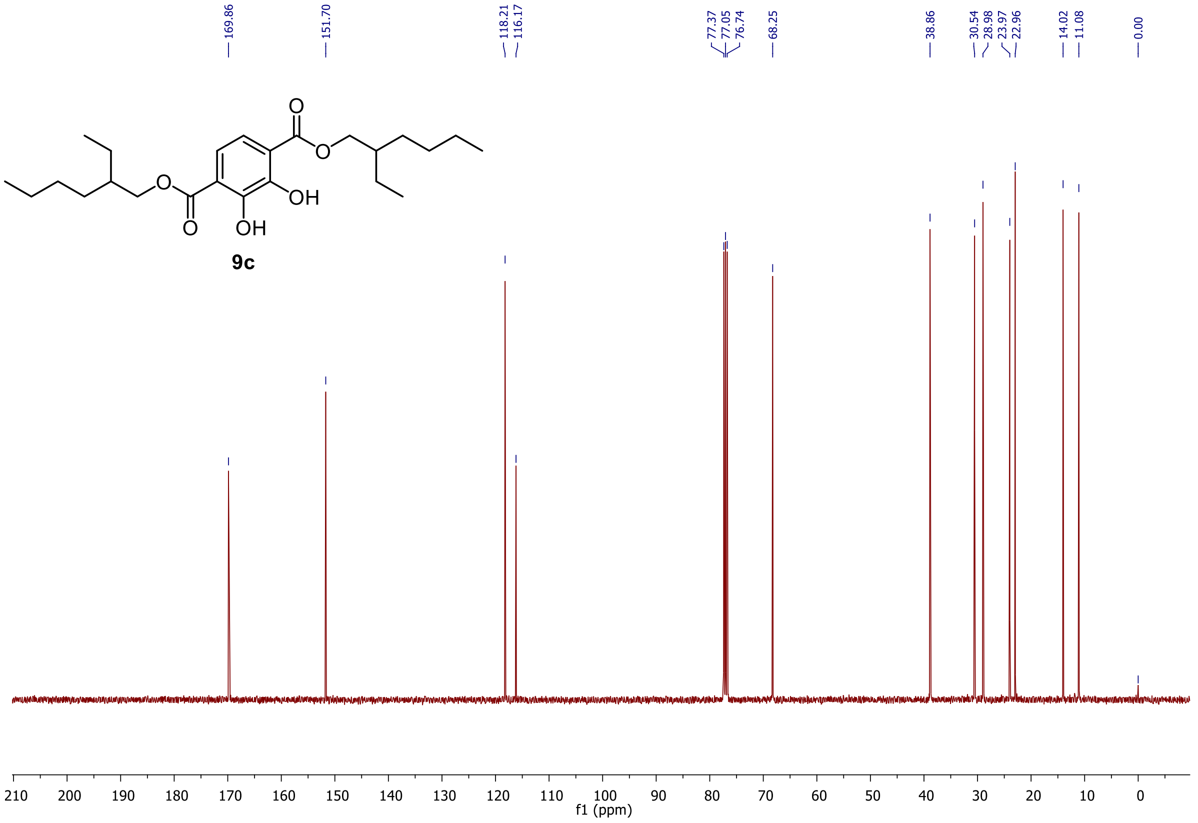

Figure S85: $^{13}C\{^1H\}$ NMR (101 MHz, $CDCl_3$) spectrum of bis(2-ethylhexyl) 2,3-dihydroxybenzene-1,4-dicarboxylate (9c) [DDV-AG-018].

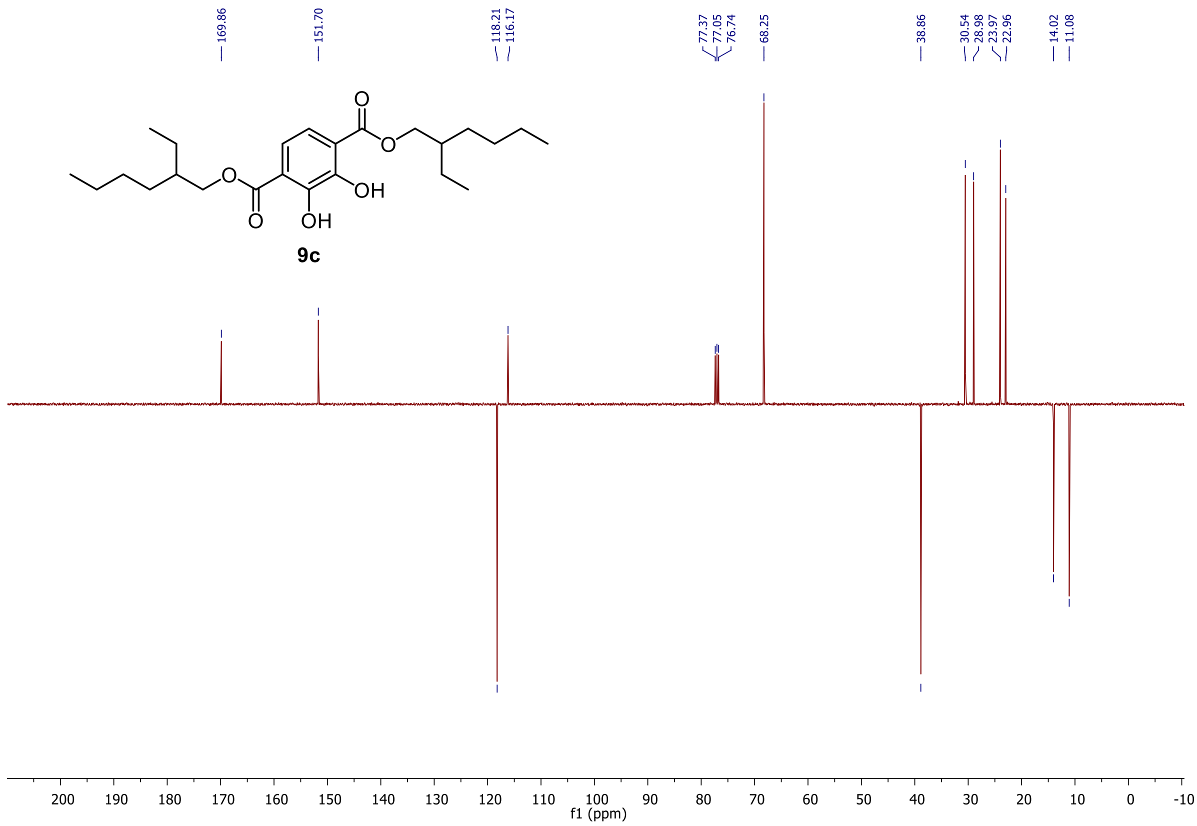

Figure S86: ^13^C APT NMR (101 MHz, CDCl3) spectrum of bis(2-ethylhexyl) 2,3-dihydroxybenzene-1,4-dicarboxylate (9c) [DDV-AG-018].

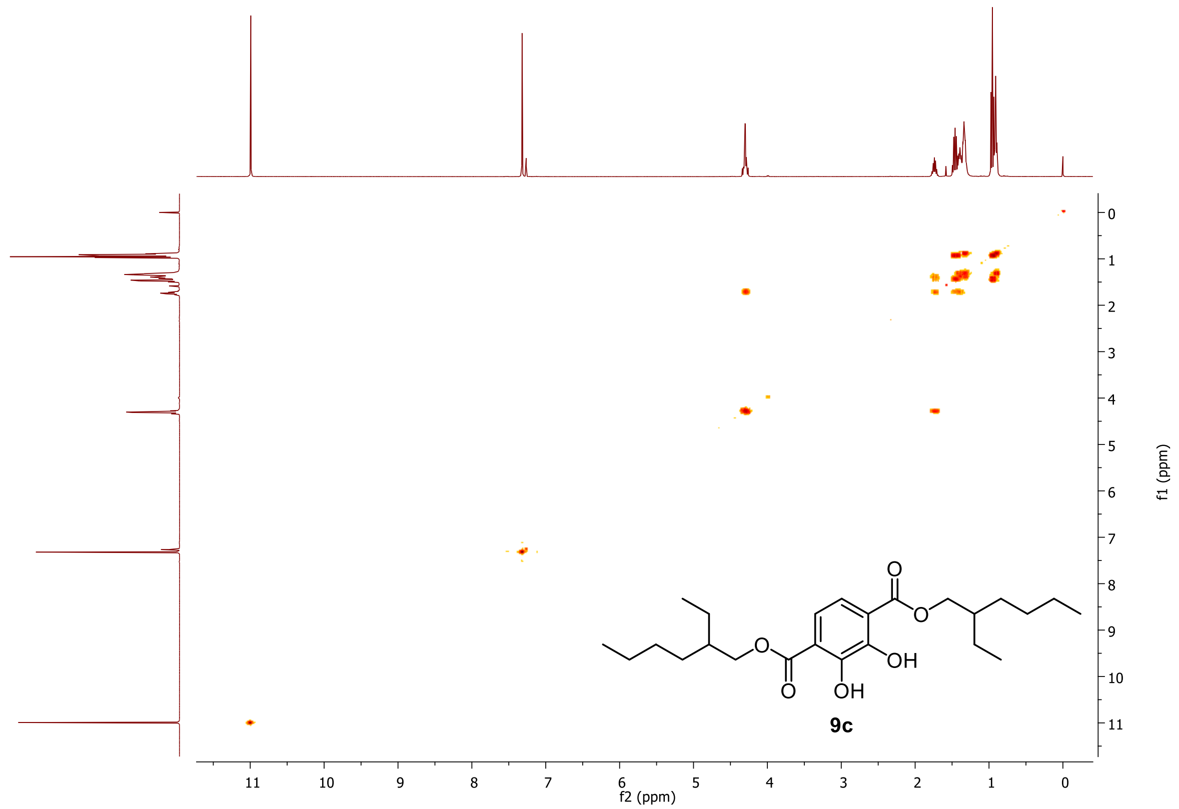

Figure S87: ^1^H COSY NMR (400 MHz, CDCl3) spectrum of bis(2-ethylhexyl) 2,3-dihydroxybenzene-1,4-dicarboxylate (9c) [DDV-AG-018].

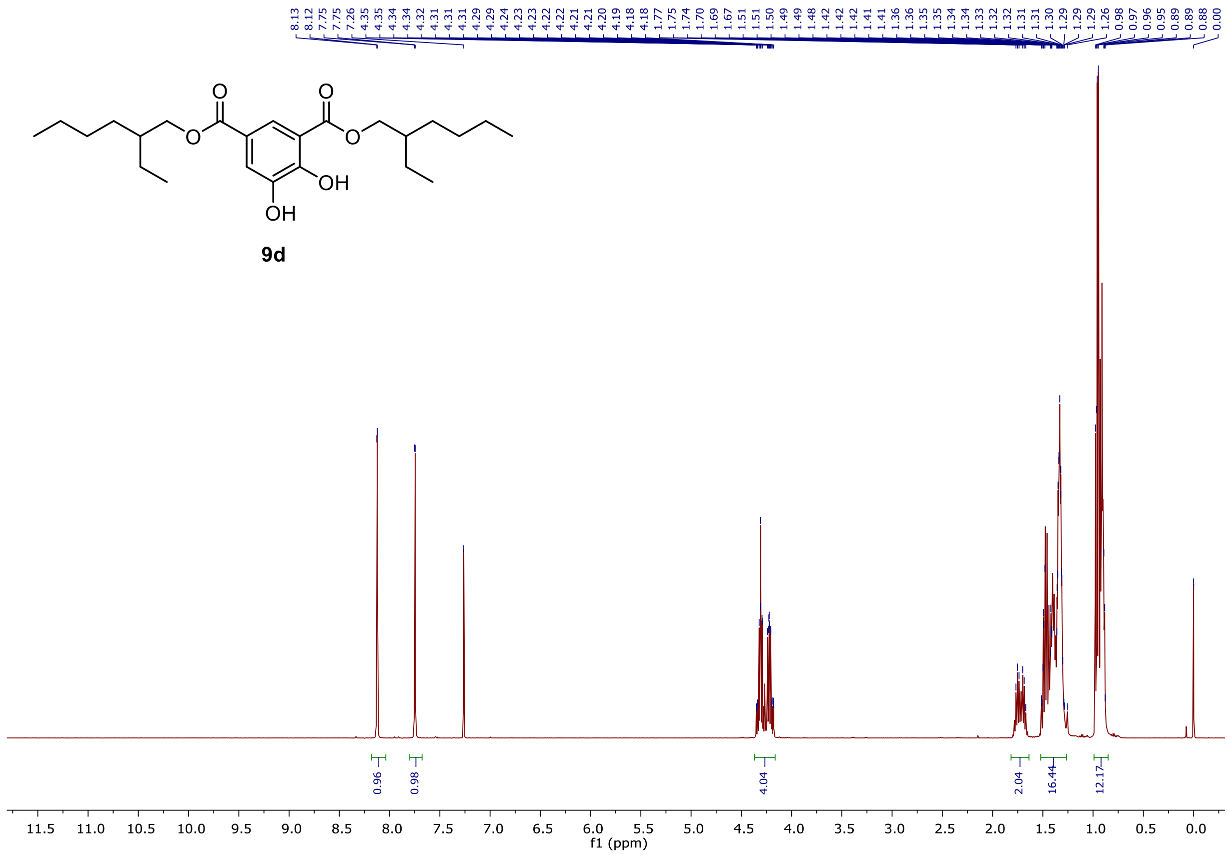

Figure S88: ^{1}H NMR (400 MHz, $CDCl_3$) spectrum of bis(2-ethylhexyl) 4,5-dihydroxybenzene-1,3-dicarboxylate (9d) [AGO-136-137-138].

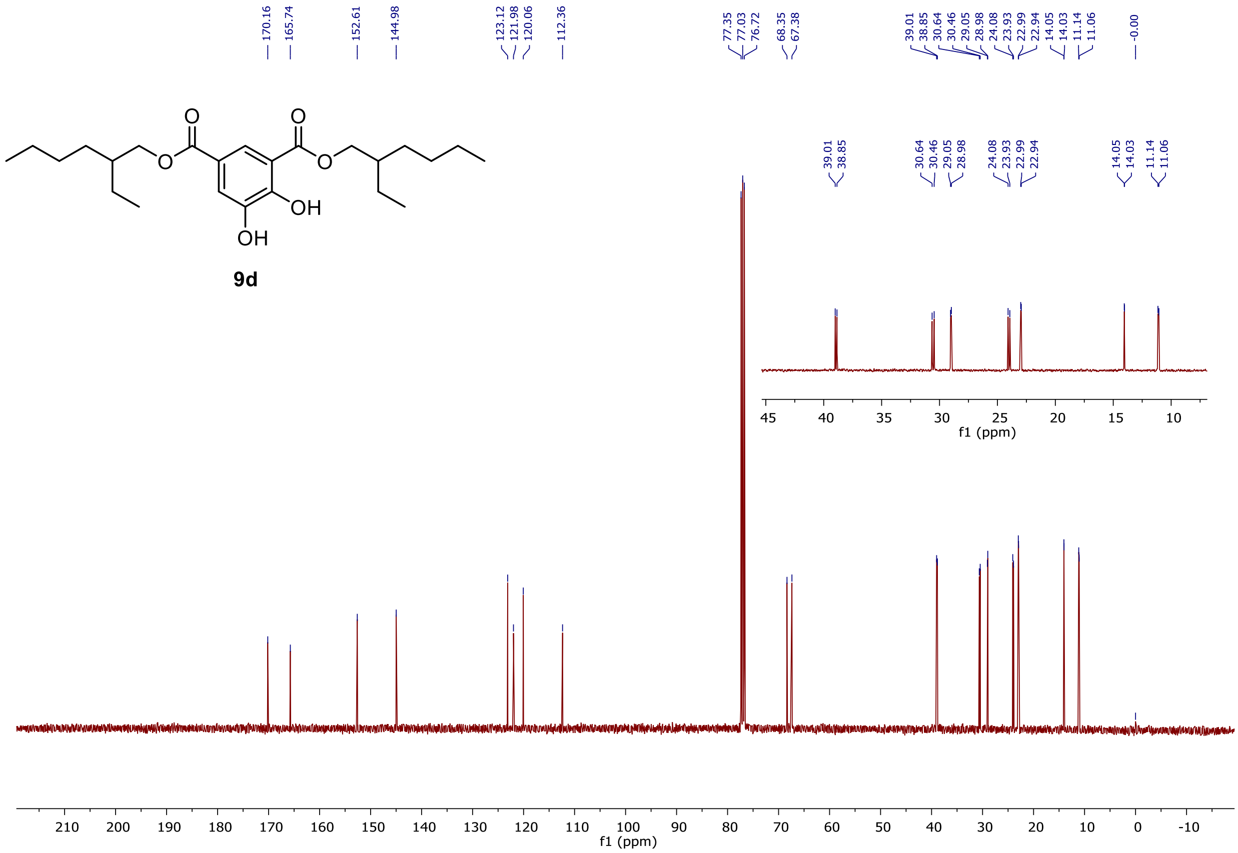

Figure S89: $^{13}C\{^1H\}$ NMR (101 MHz, $CDCl_3$) spectrum of bis(2-ethylhexyl) 4,5-dihydroxybenzene-1,3-dicarboxylate (9d) [AGO-136-137-138].

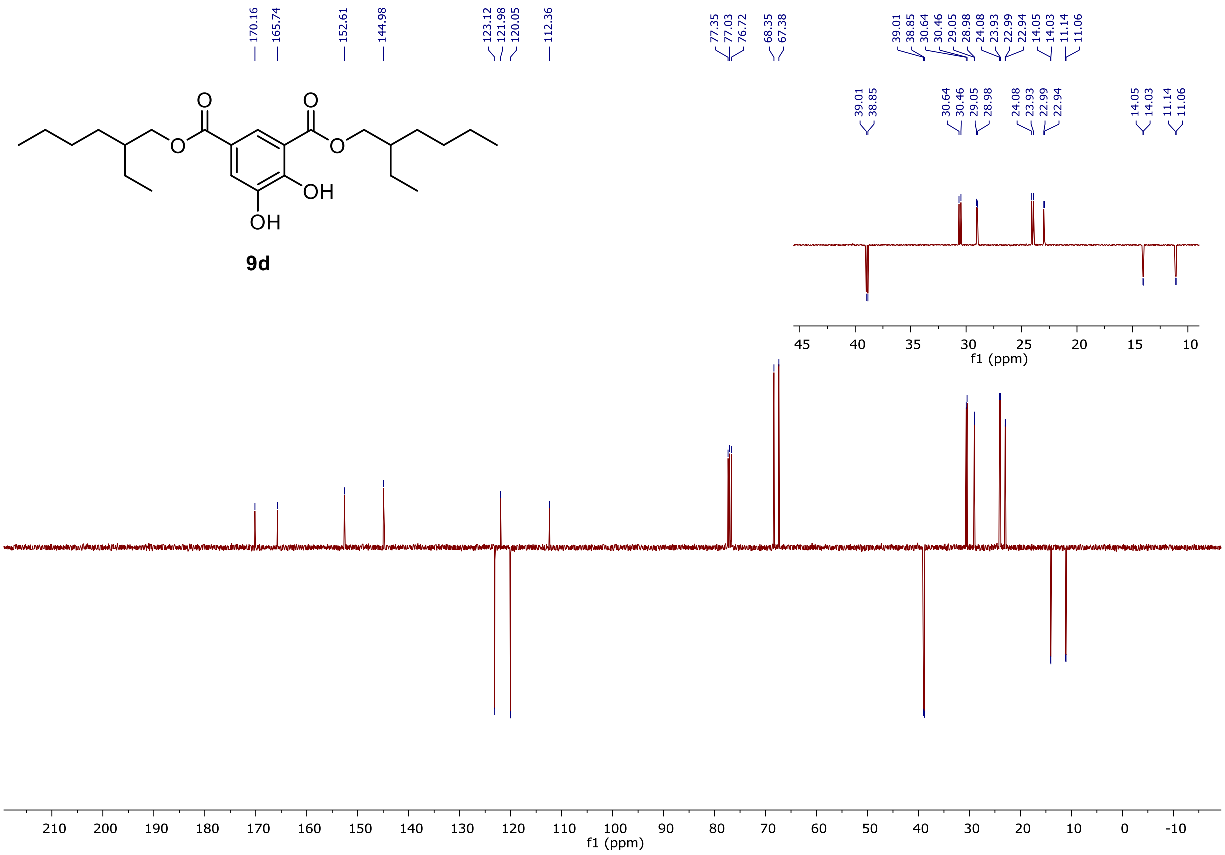

Figure S90: ^{13}C APT NMR (101 MHz, $CDCl_3$) spectrum of bis(2-ethylhexyl) 4,5-dihydroxybenzene-1,3-dicarboxylate (9d) [AGO-136-137-138].

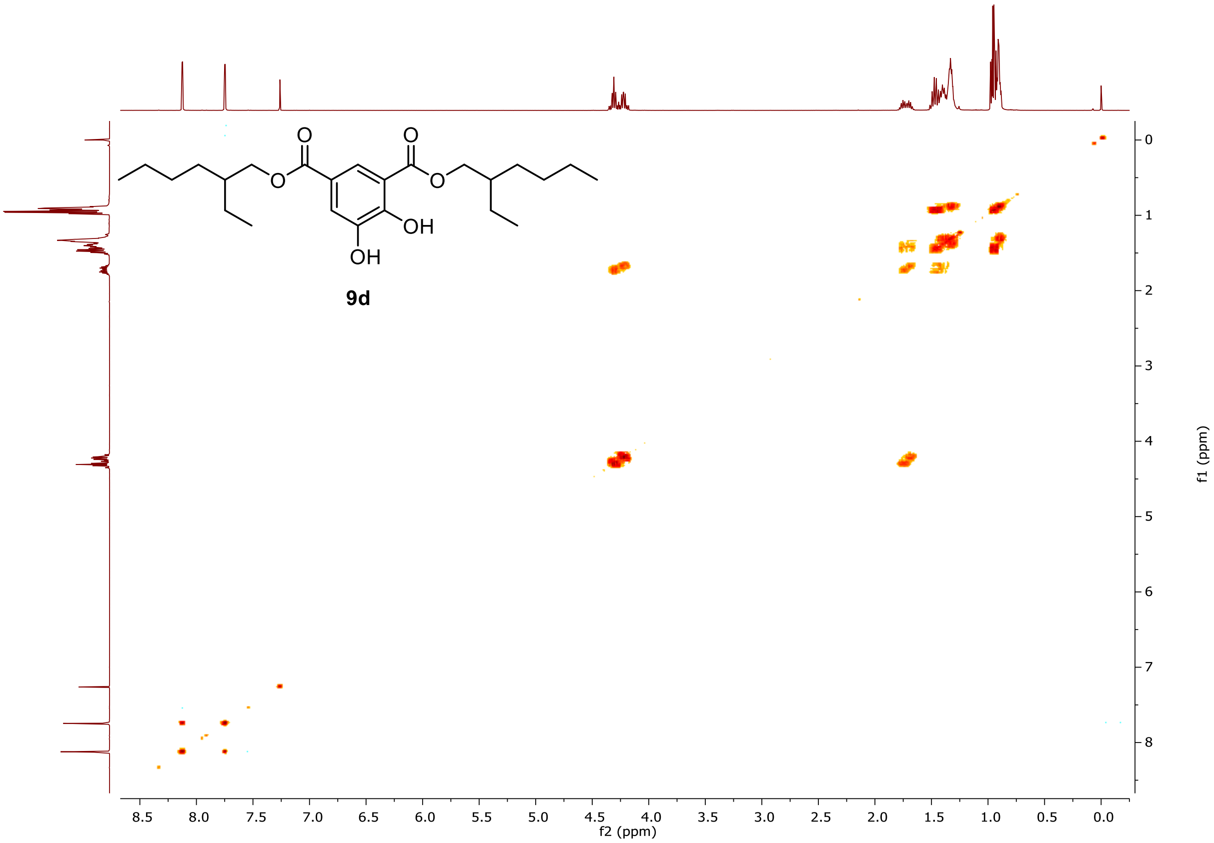

Figure S91: ^{1}H COSY NMR (400 MHz, $CDCl_3$) spectrum of bis(2-ethylhexyl) 4,5-dihydroxybenzene-1,3-dicarboxylate (9d) [AGO-136-137-138].

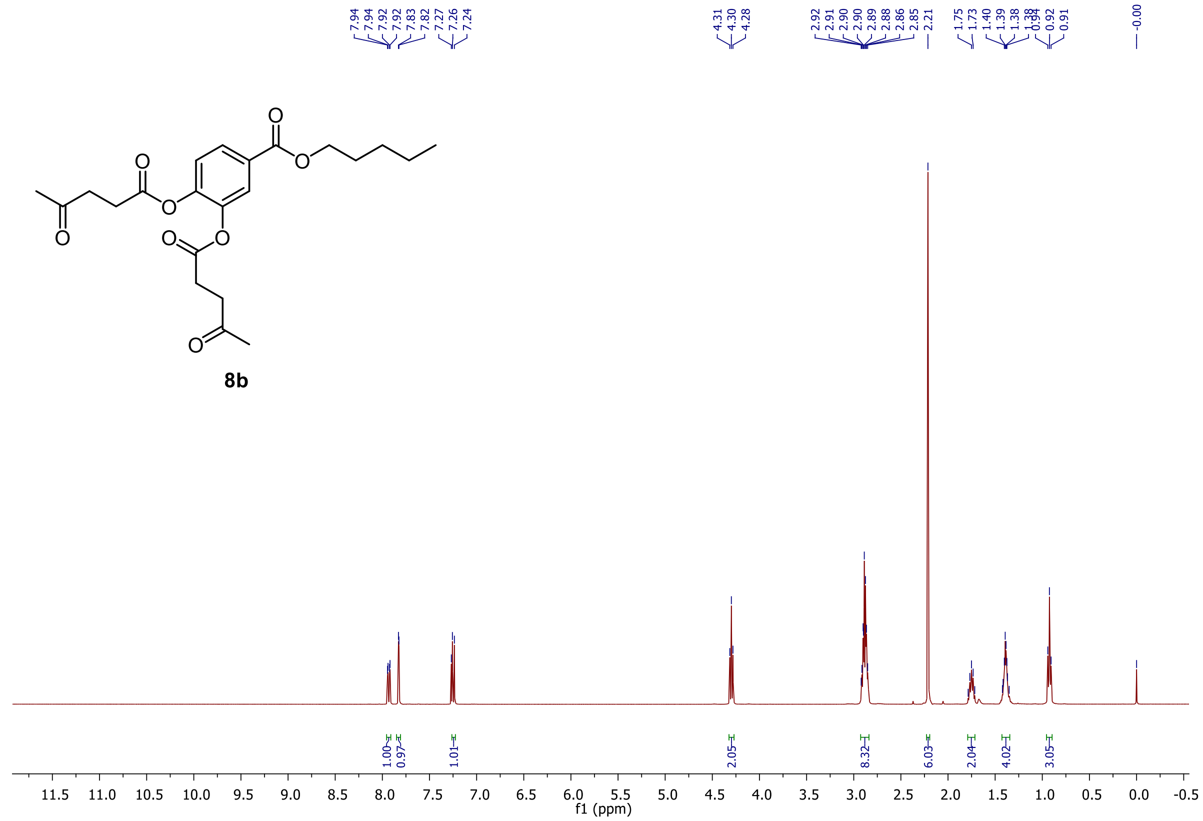

Figure S92: ^{1}H NMR (400 MHz, CDCl$_3$) spectrum of pentyl 3,4-bis[(4-oxopentanoyl)oxy]benzoate (8b) [DDV-AG-014].

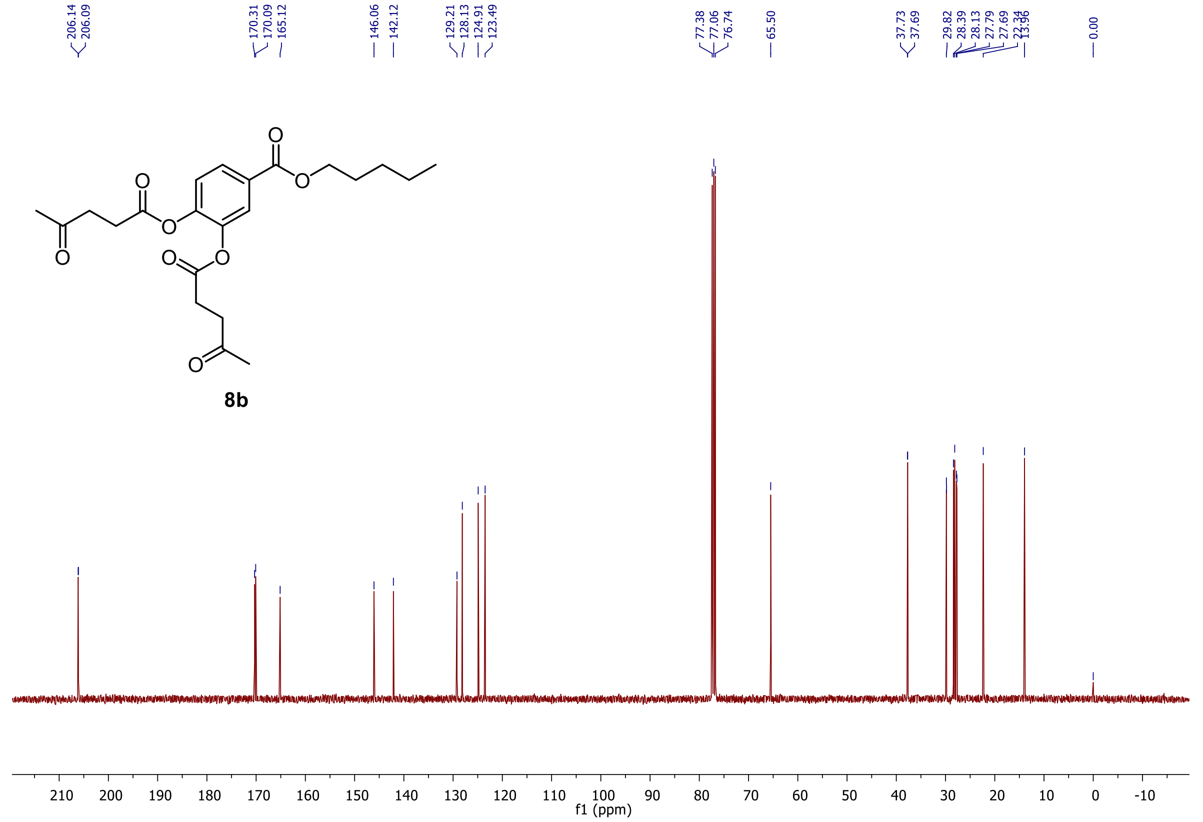

Figure S93: ^{13}C{^{1}H} NMR (101 MHz, CDCl$_3$) spectrum of pentyl 3,4-bis[(4-oxopentanoyl)oxy]benzoate (8b) [DDV-AG-014].

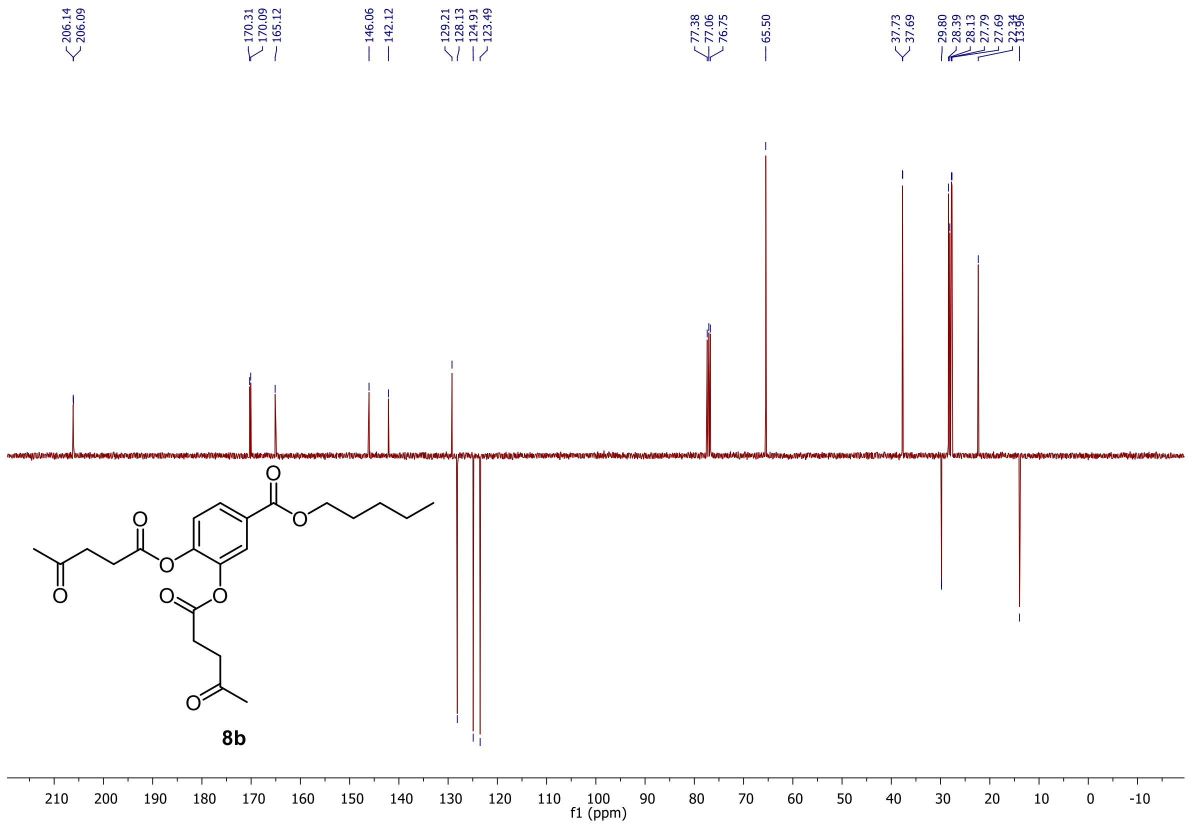

Figure S94: ^{13}C APT NMR (101 MHz, CDCl$_3$) spectrum of pentyl 3,4-bis[(4-oxopentanoyl)oxy]benzoate (8b) [DDV-AG-014].

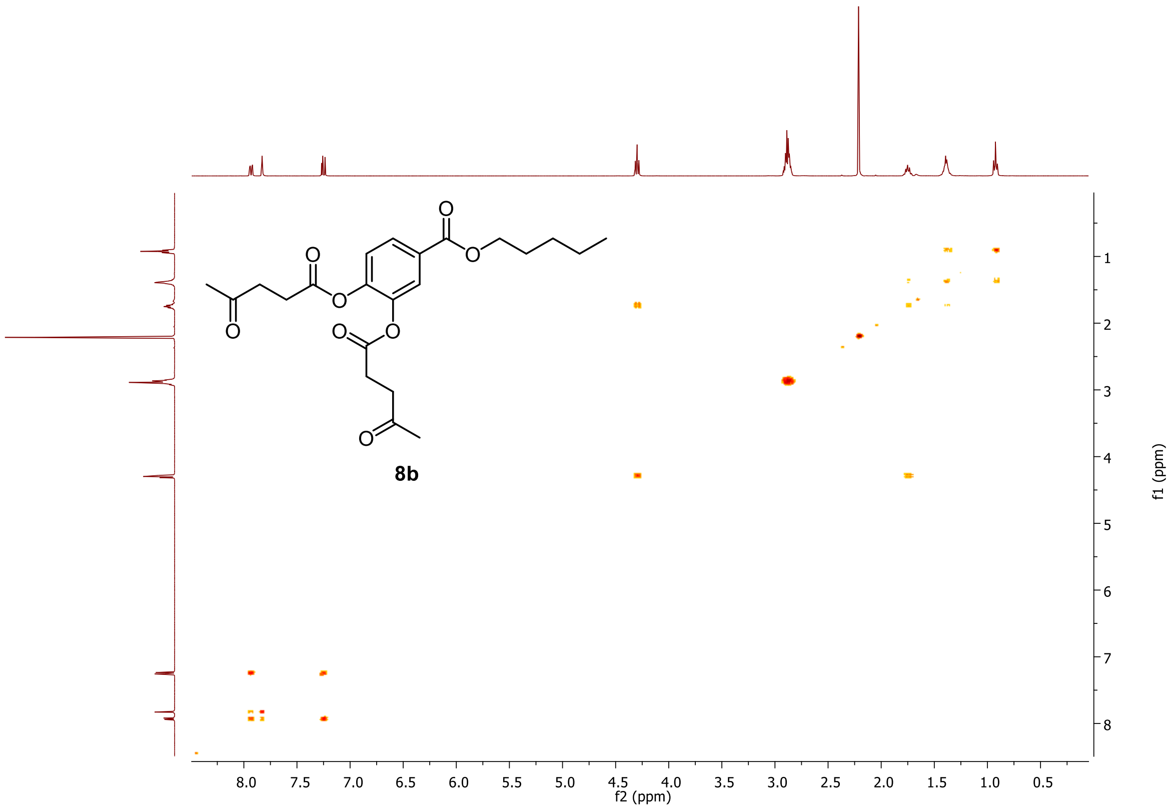

Figure S95: ^{1}H COSY NMR (400 MHz, CDCl$_3$) spectrum of pentyl 3,4-bis[(4-oxopentanoyl)oxy]benzoate (8b) [DDV-AG-014].

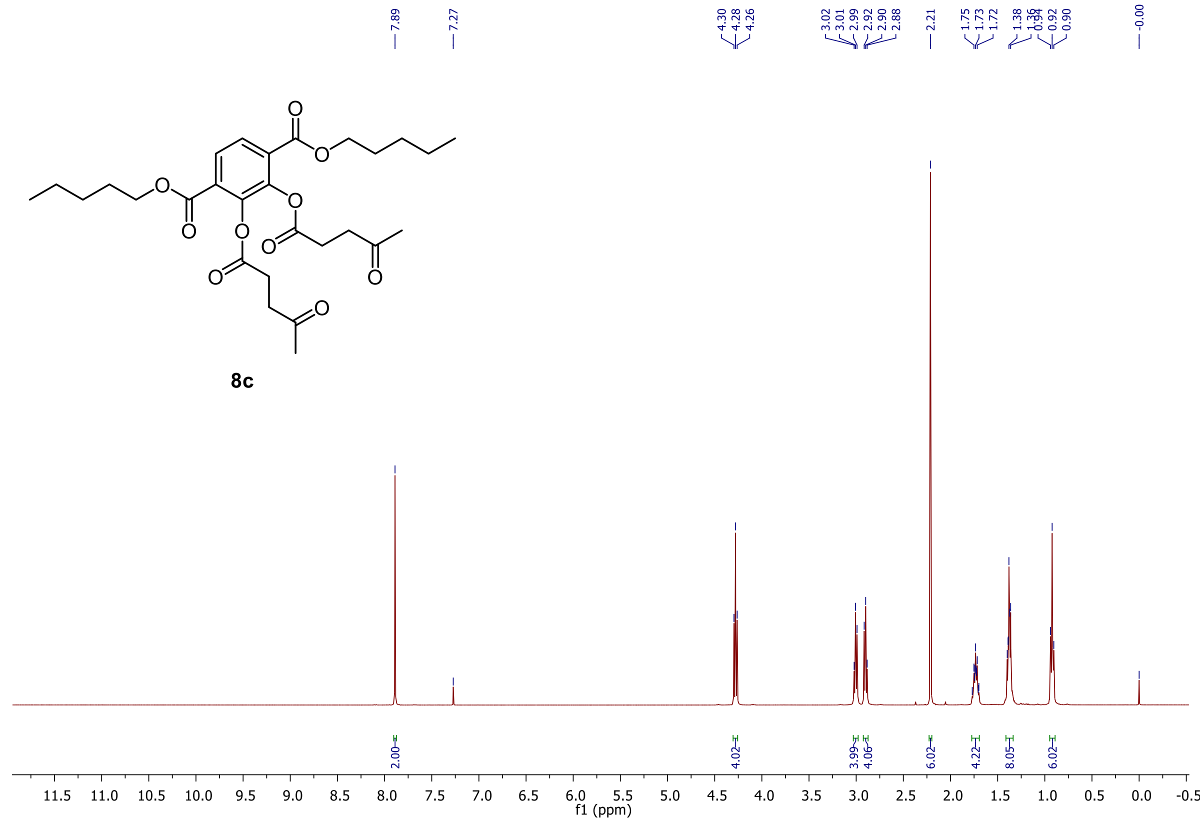

Figure S96: ^{1}H NMR (400 MHz, $CDCl_3$) spectrum of dipentyl 2,3-bis[(4-oxopentanoyl)oxy]benzene-1,4-dicarboxylate (8c) [DDV-AG-022].

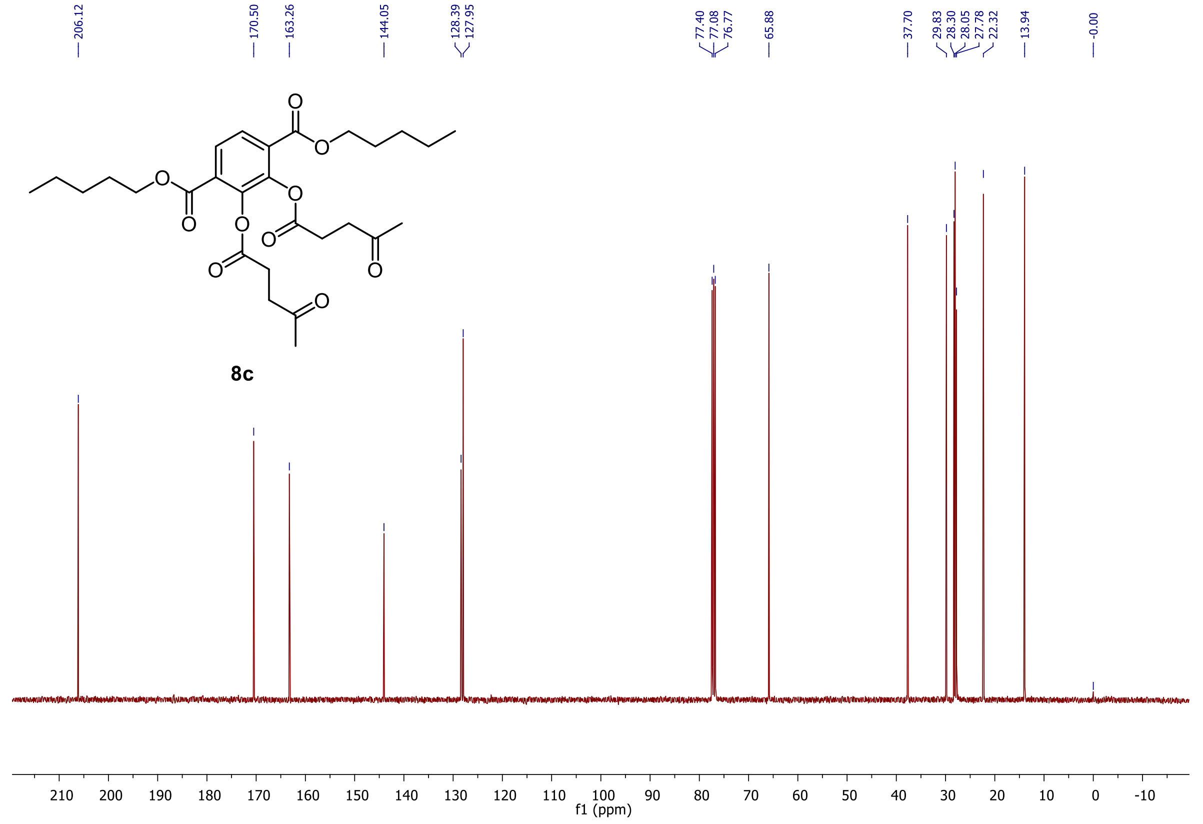

Figure S97: $^{13}C\{^{1}H\}$ NMR (101 MHz, $CDCl_3$) spectrum of dipentyl 2,3-bis[(4-oxopentanoyl)oxy]benzene-1,4-dicarboxylate (8c) [DDV-AG-022].

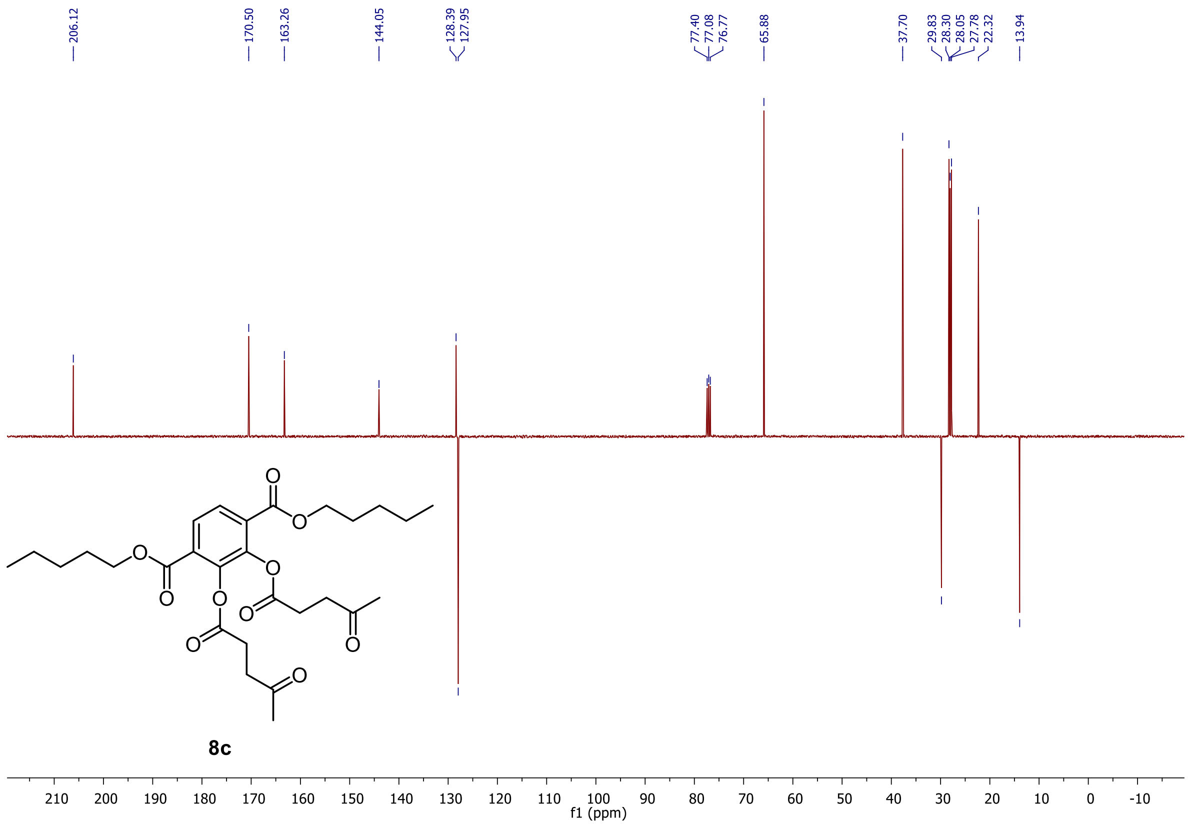

Figure S98: ^{13}C APT NMR (101 MHz, $CDCl_3$) spectrum of dipentyl 2,3-bis[(4-oxopentanoyl)oxy]benzene-1,4-dicarboxylate (8c) [DDV-AG-022].

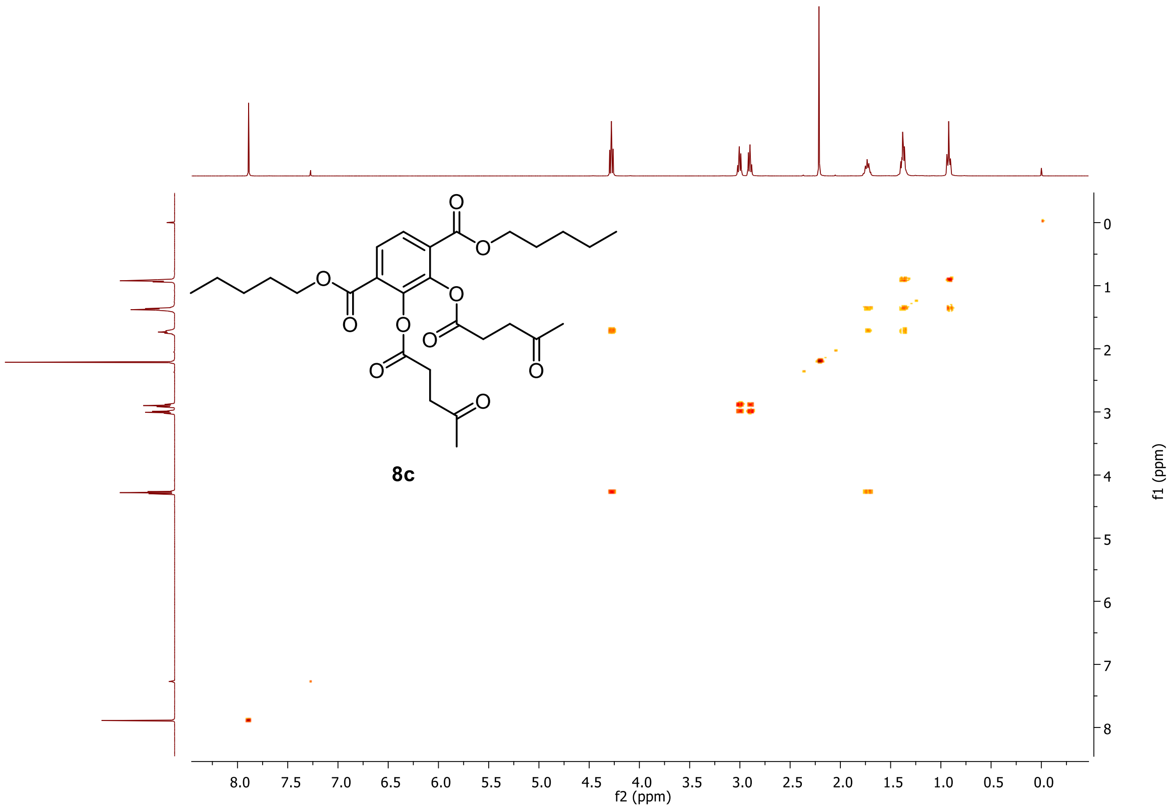

Figure S99: ^{1}H COSY NMR (400 MHz, $CDCl_3$) spectrum of dipentyl 2,3-bis[(4-oxopentanoyl)oxy]benzene-1,4-dicarboxylate (8c) [DDV-AG-022].

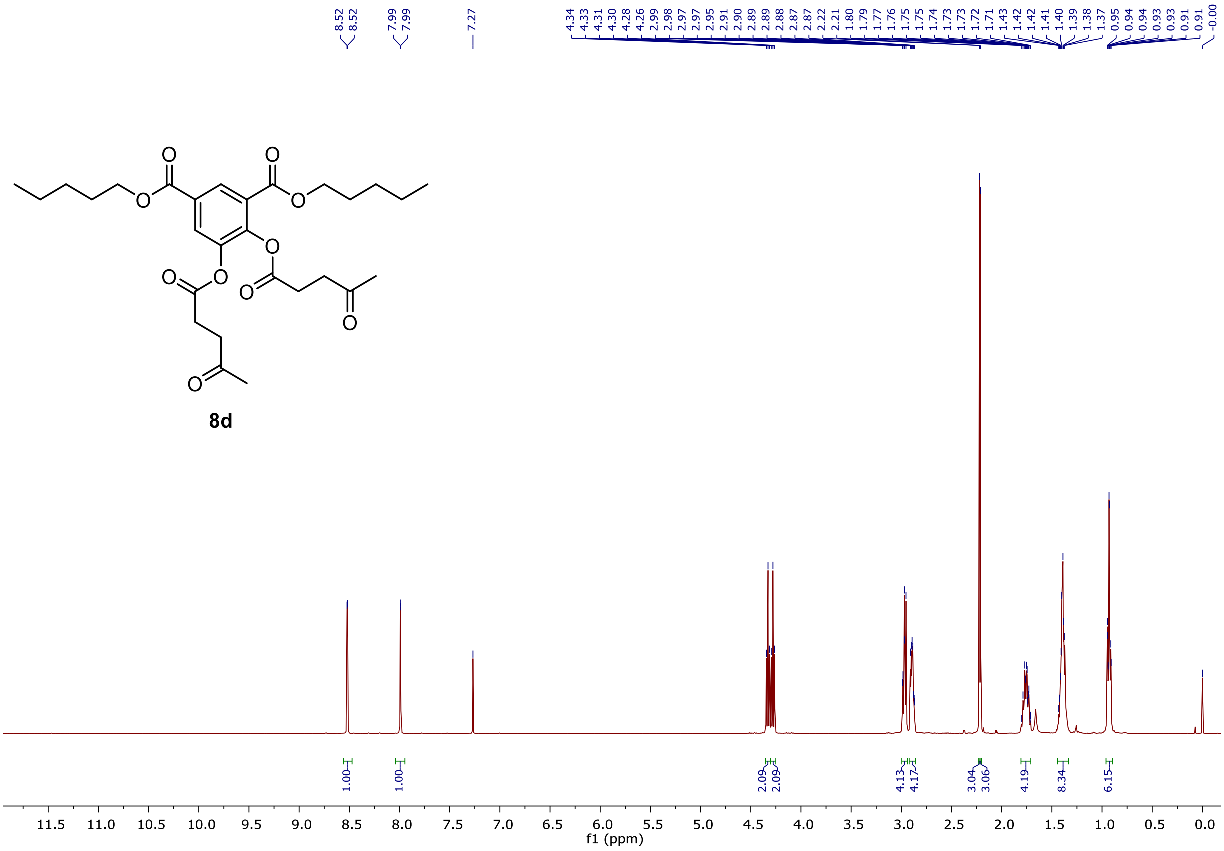

Figure S100: ¹H NMR (400 MHz, CDCl₃) spectrum of dipentyl 4,5-bis[(4-oxopentanoyl)oxy]benzene-1,3-dicarboxylate (8d) [AGO-135].

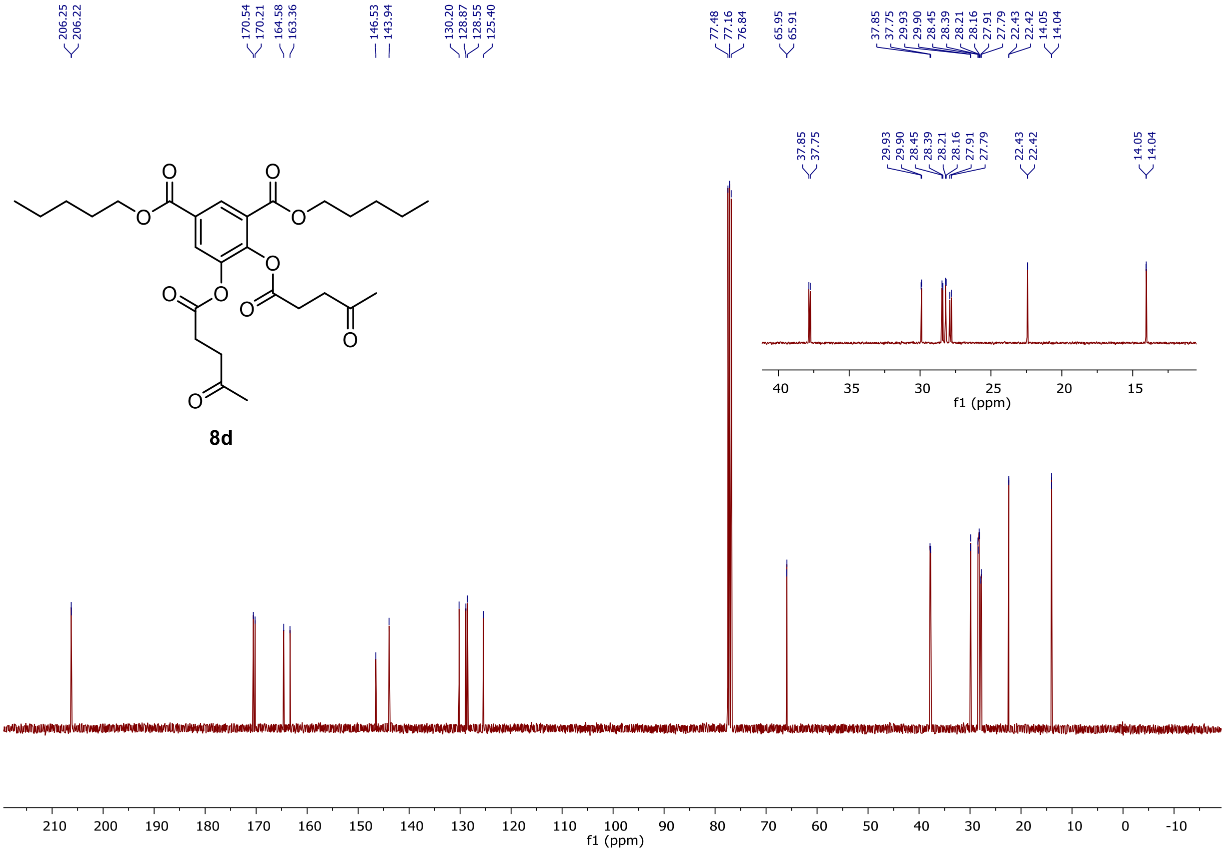

Figure S101: $^{13}C\{^{1}H\}$ NMR (101 MHz, CDCl₃) spectrum of dipentyl 4,5-bis[(4-oxopentanoyl)oxy]benzene-1,3-dicarboxylate (8d) [AGO-135].

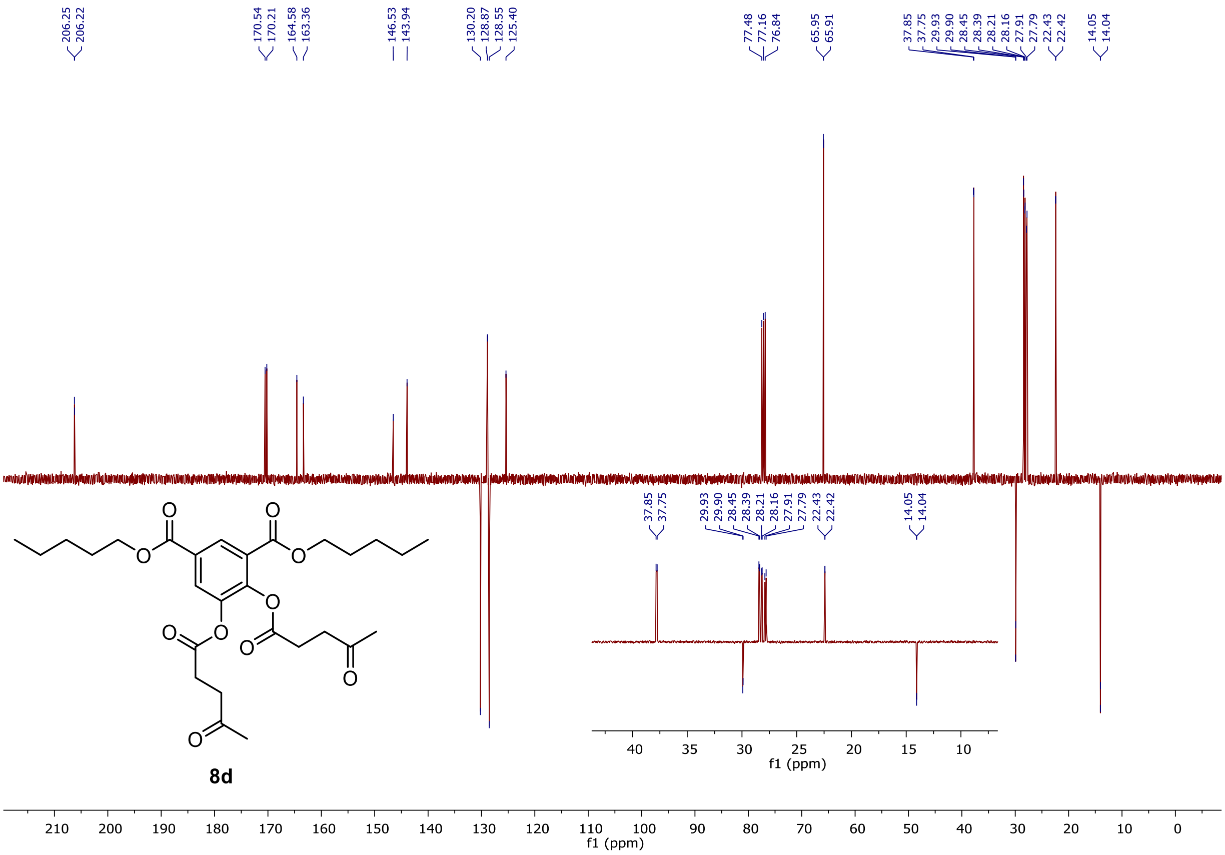

Figure S102: ^{13}C APT NMR (101 MHz, $CDCl_3$) spectrum of dipentyl 4,5-bis[(4-oxopentanoyl)oxy]benzene-1,3-dicarboxylate (8d) [AGO-135].

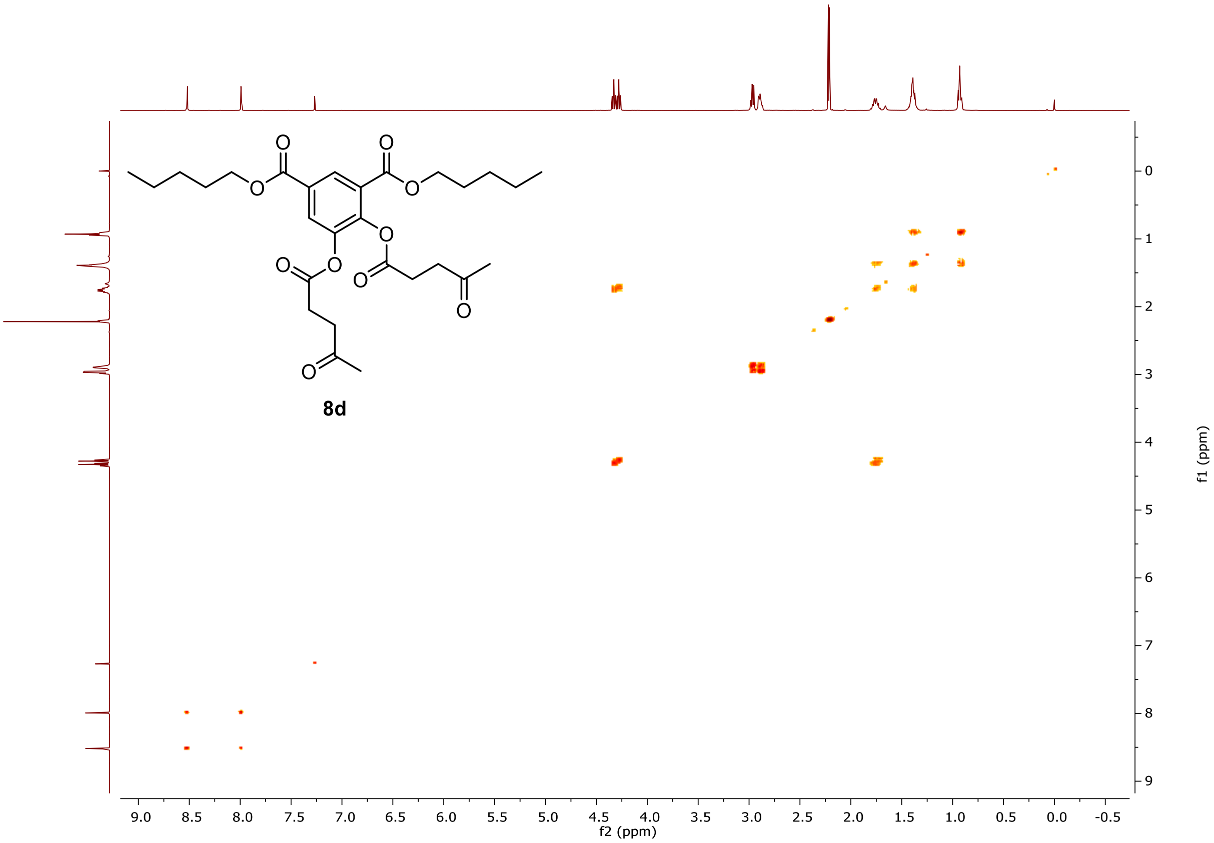

Figure S103: ^{1}H COSY NMR (400 MHz, $CDCl_3$) spectrum of dipentyl 4,5-bis[(4-oxopentanoyl)oxy]benzene-1,3-dicarboxylate (8d) [AGO-135].

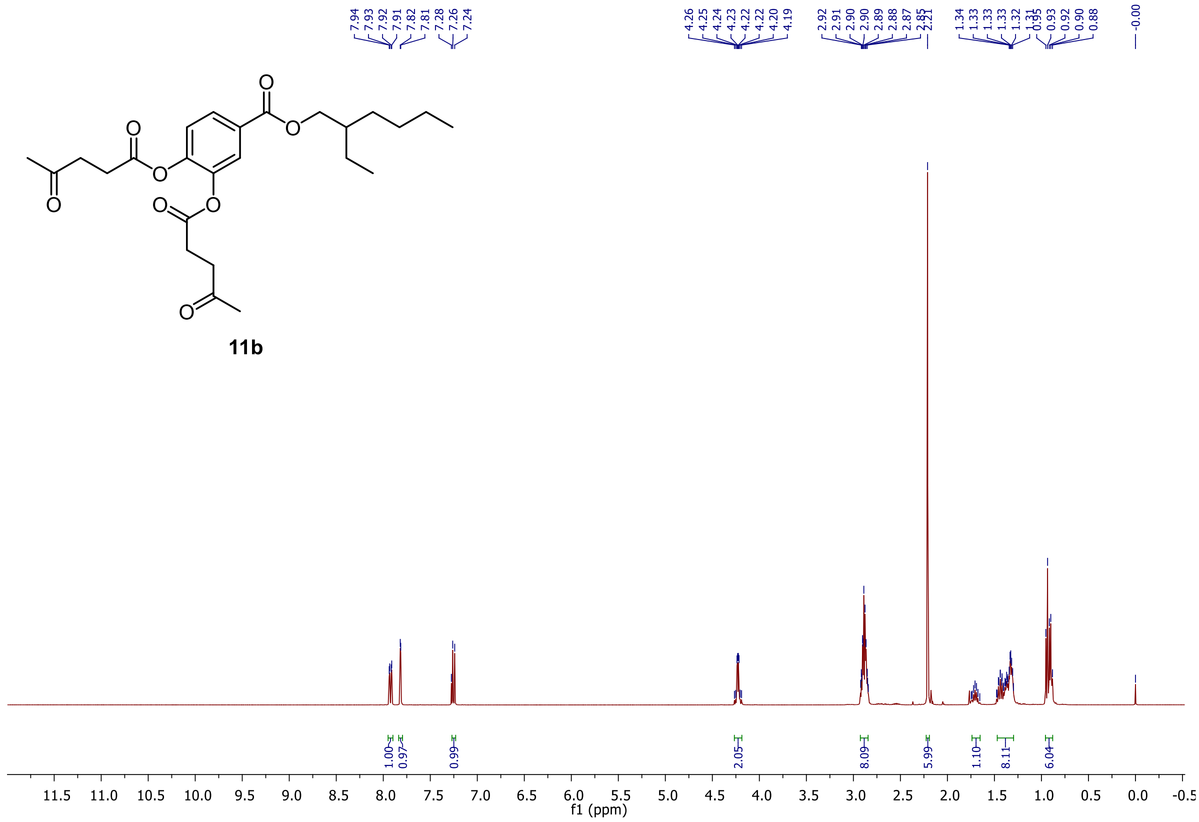

Figure S104: ^{1}H NMR (400 MHz, $CDCl_3$) spectrum of 2-ethylhexyl 3,4-bis[(4-oxopentanoyl)oxy]benzoate (11b) [DDV-AG-021].

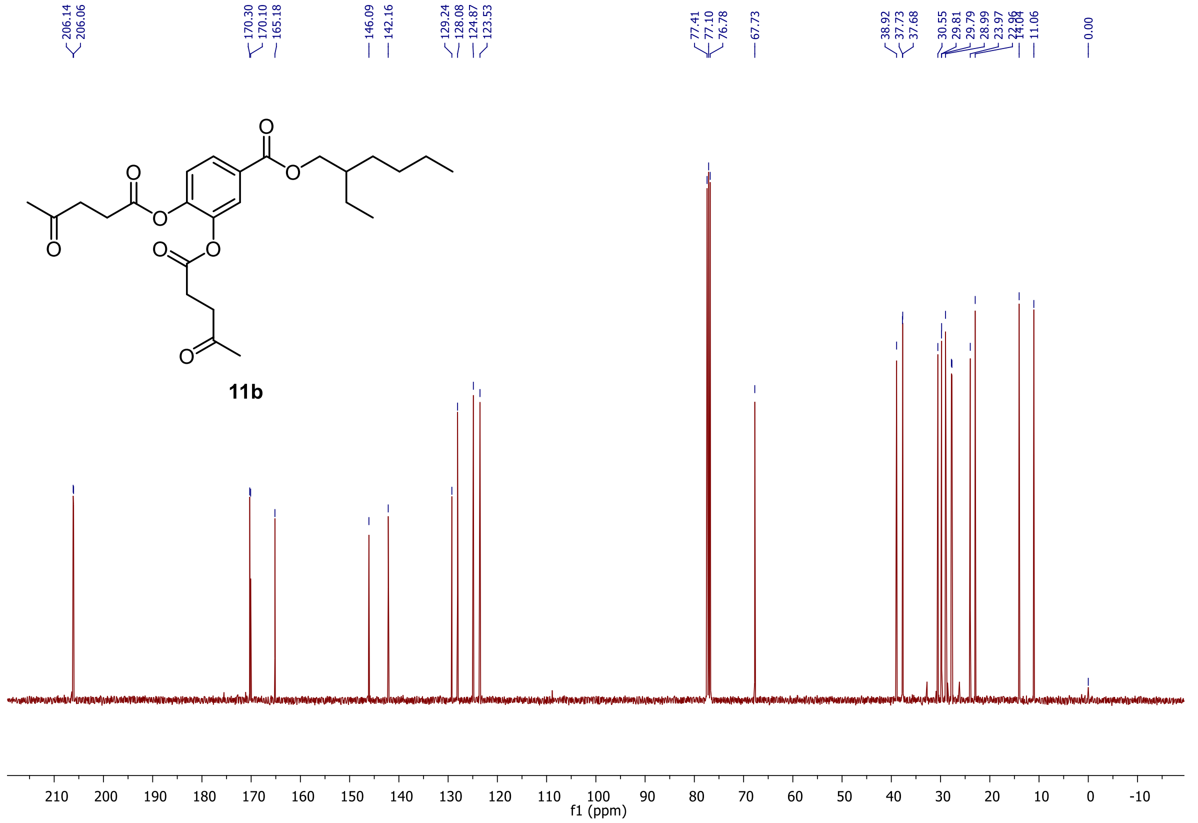

Figure S105: $^{13}C\{^{1}H\}$ NMR (101 MHz, $CDCl_3$) spectrum of 2-ethylhexyl 3,4-bis[(4-oxopentanoyl)oxy]benzoate (11b) [DDV-AG-021].

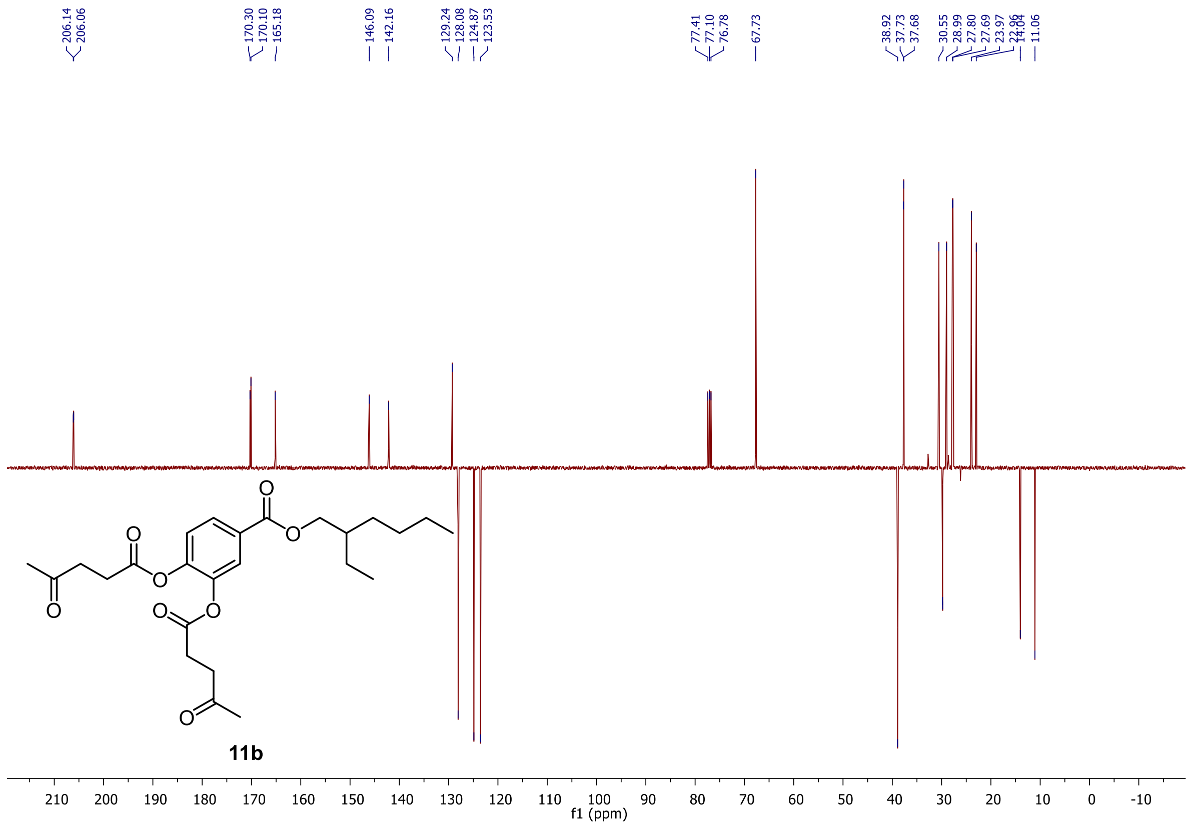

Figure S106: ^{13}C APT NMR (101 MHz, $CDCl_3$) spectrum of 2-ethylhexyl 3,4-bis[(4-oxopentanoyl)oxy]benzoate (11b) [DDV-AG-021].

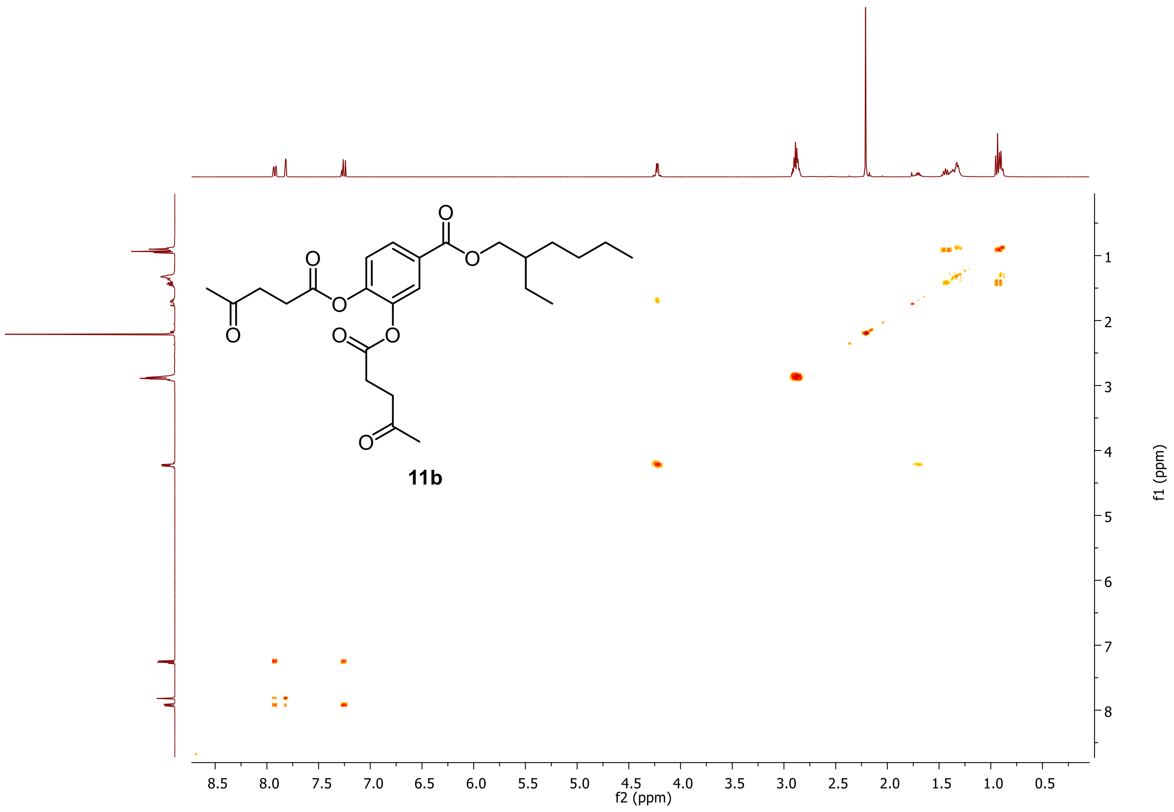

Figure S107: ^1H COSY NMR (400 MHz, $CDCl_3$) spectrum of 2-ethylhexyl 3,4-bis[(4-oxopentanoyl)oxy]benzoate (11b) [DDV-AG-021].

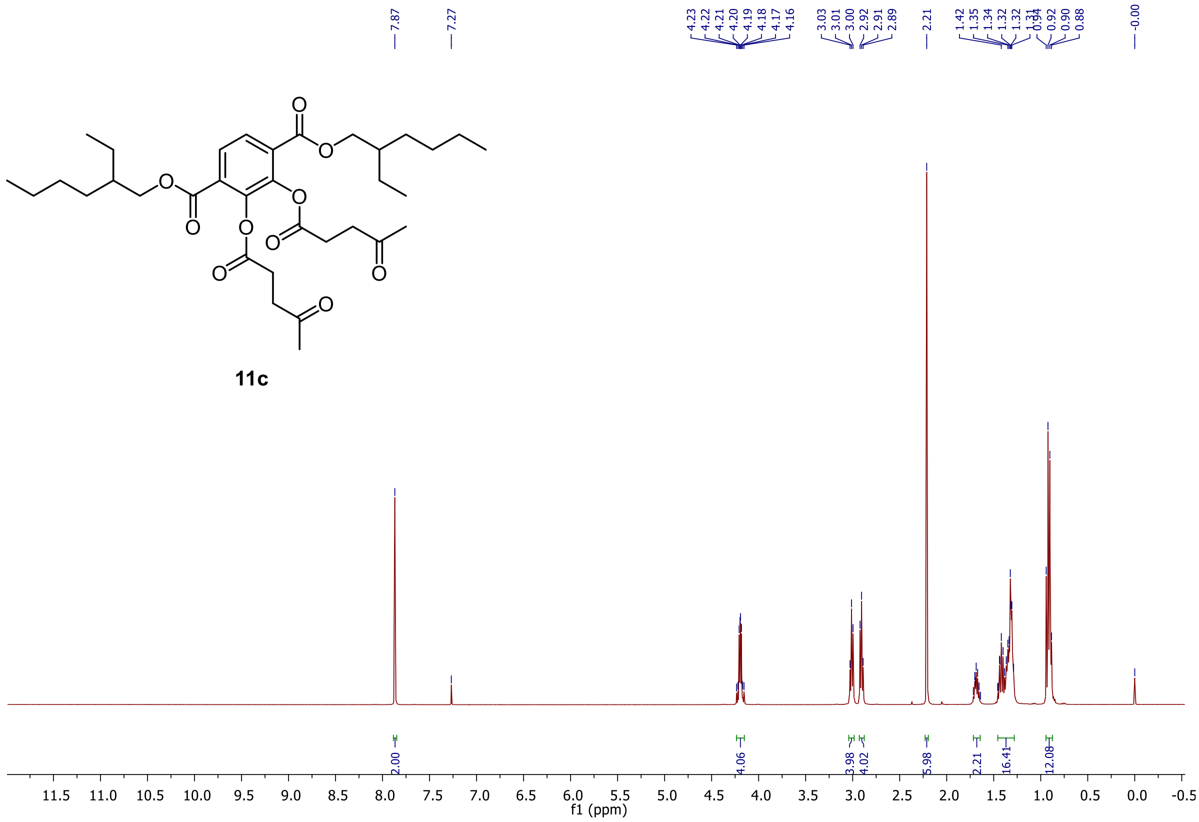

Figure S108: ^{1}H NMR (400 MHz, $CDCl_3$) spectrum of bis(2-ethylhexyl) 2,3-bis[(4-oxopentanoyl)oxy]benzene-1,4-dicarboxylate (11c) [DDV-AG-023].

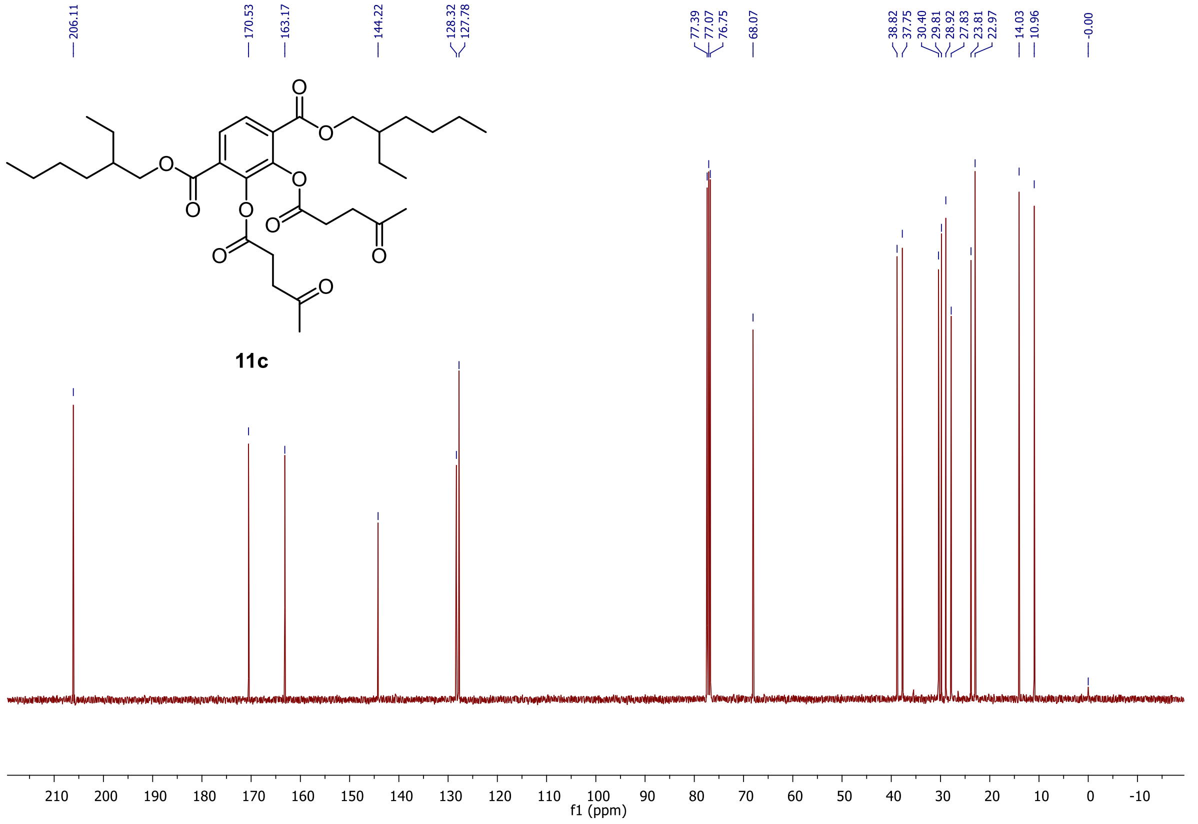

Figure S109: $^{13}C\{^{1}H\}$ NMR (101 MHz, $CDCl_3$) spectrum of bis(2-ethylhexyl) 2,3-bis[(4-oxopentanoyl)oxy]benzene-1,4-dicarboxylate (11c) [DDV-AG-023].

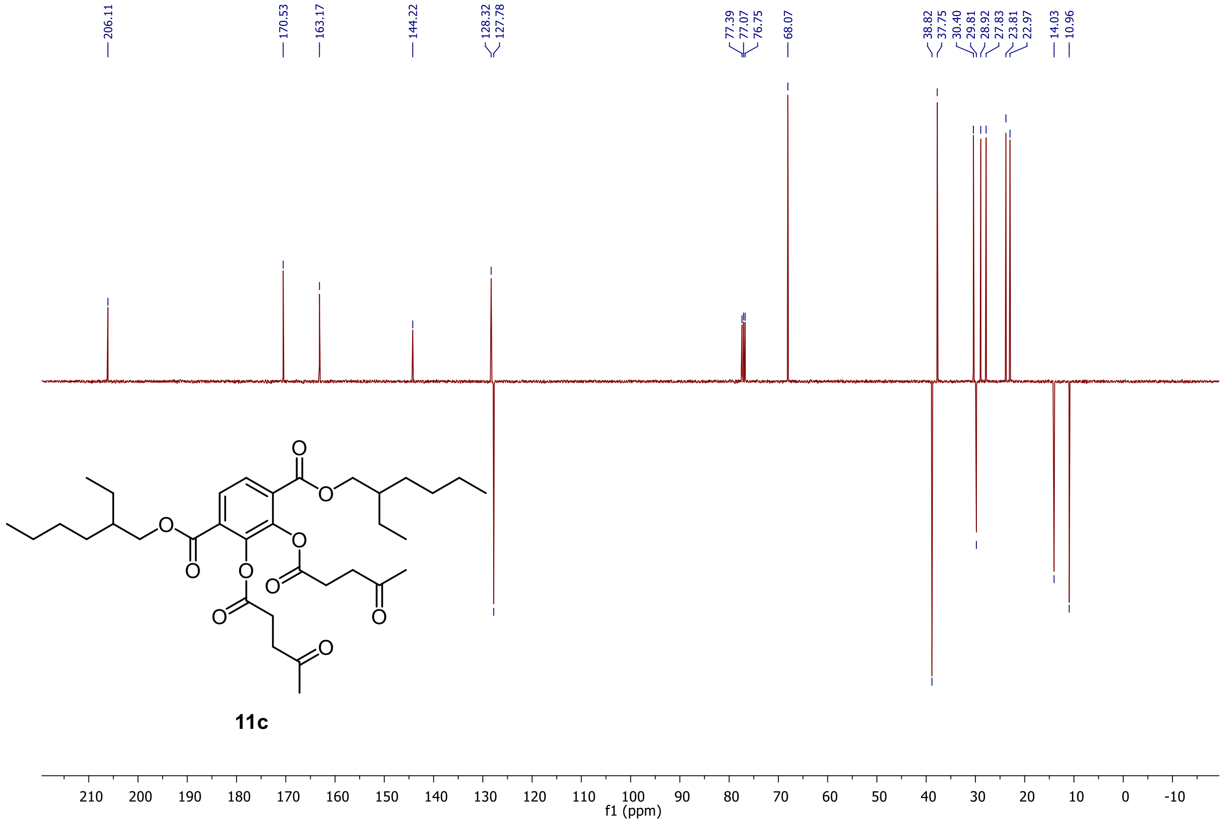

Figure S110: ^{13}C **APT NMR (101 MHz, $CDCl_3$) spectrum of bis(2-ethylhexyl) 2,3-bis[(4-oxopentanoyl)oxy]benzene-1,4-dicarboxylate (11c) [DDV-AG-023].**

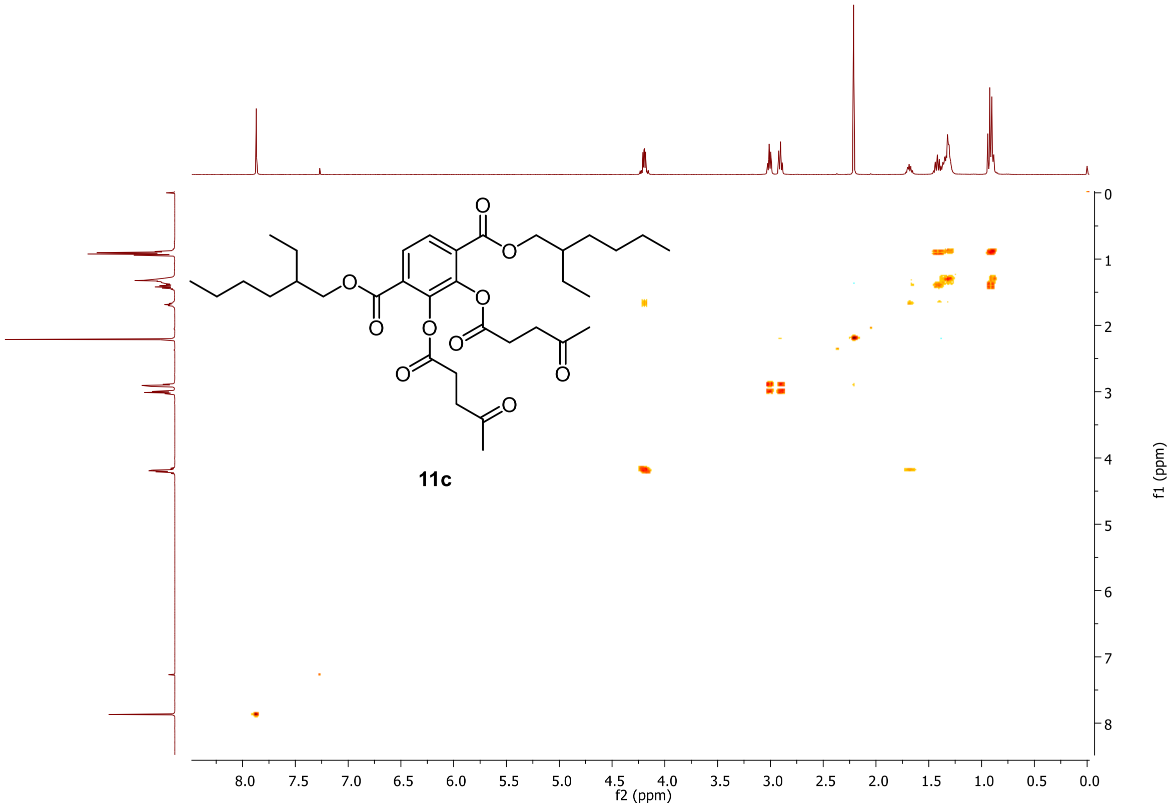

Figure S111: ^{1}H **COSY NMR (400 MHz, $CDCl_3$) spectrum of bis(2-ethylhexyl) 2,3-bis[(4-oxopentanoyl)oxy]benzene-1,4-dicarboxylate (11c) [DDV-AG-023].**

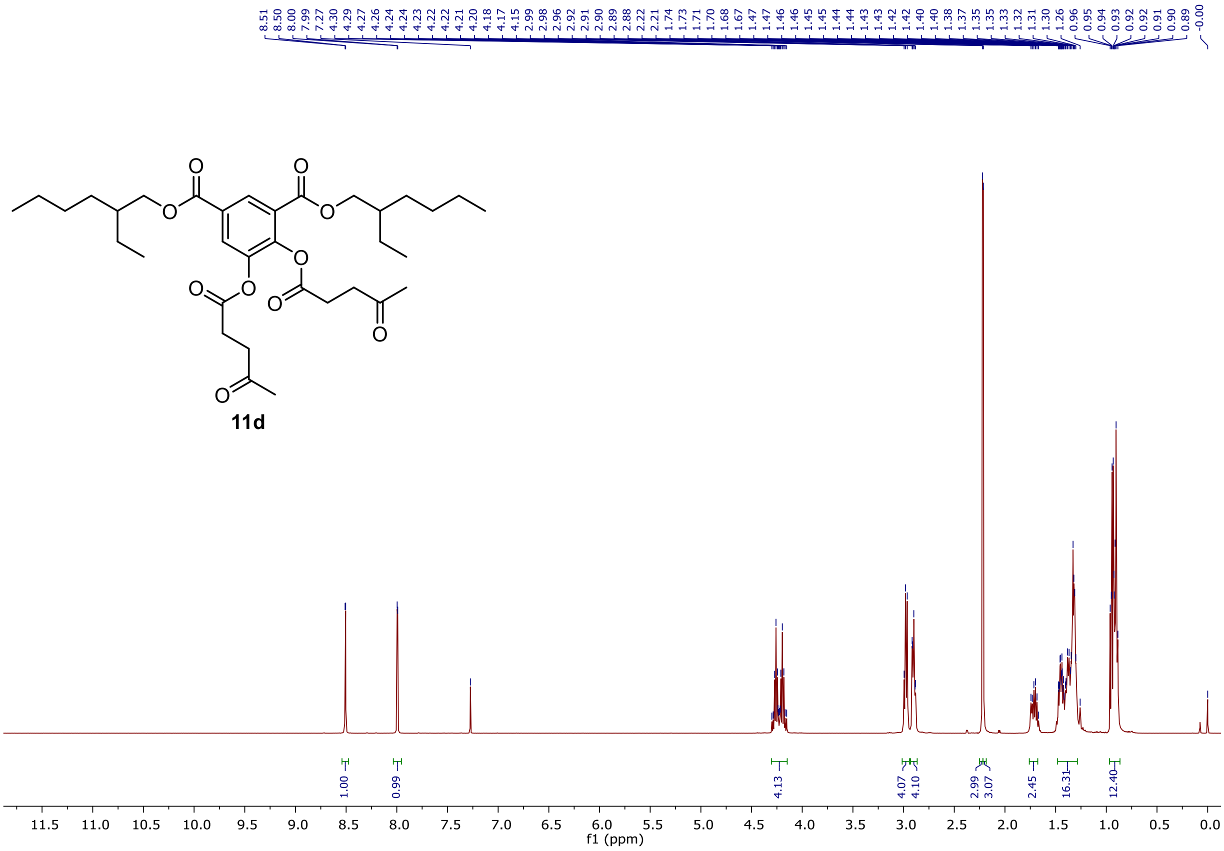

Figure S112: ^{1}H NMR (400 MHz, $CDCl_3$) spectrum of bis(2-ethylhexyl) 4,5-bis[(4-oxopentanoyl)oxy]benzene-1,3-dicarboxylate (11d) [AGO-139].

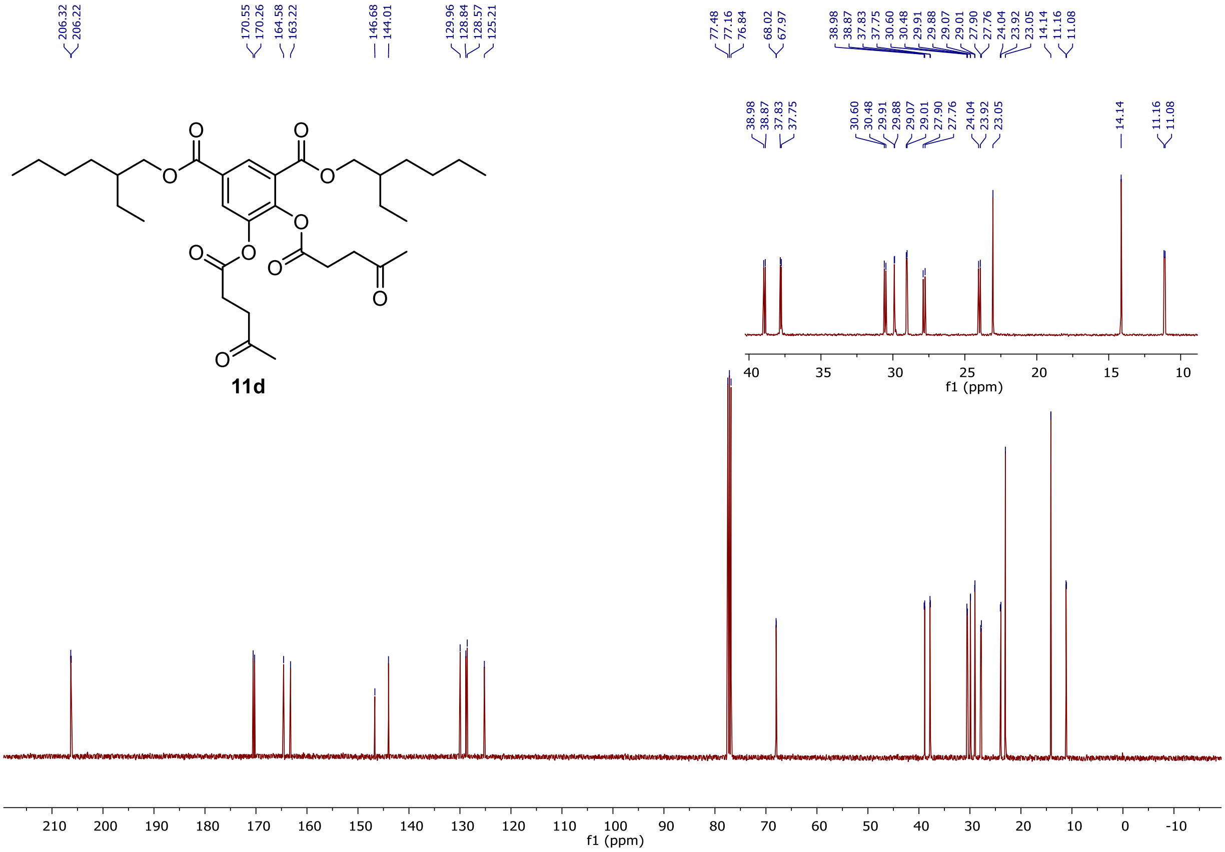

Figure S113: $^{13}C\{^{1}H\}$ NMR (400 MHz, $CDCl_3$) spectrum of bis(2-ethylhexyl) 4,5-bis[(4-oxopentanoyl)oxy]benzene-1,3-dicarboxylate (11d) [AGO-139].

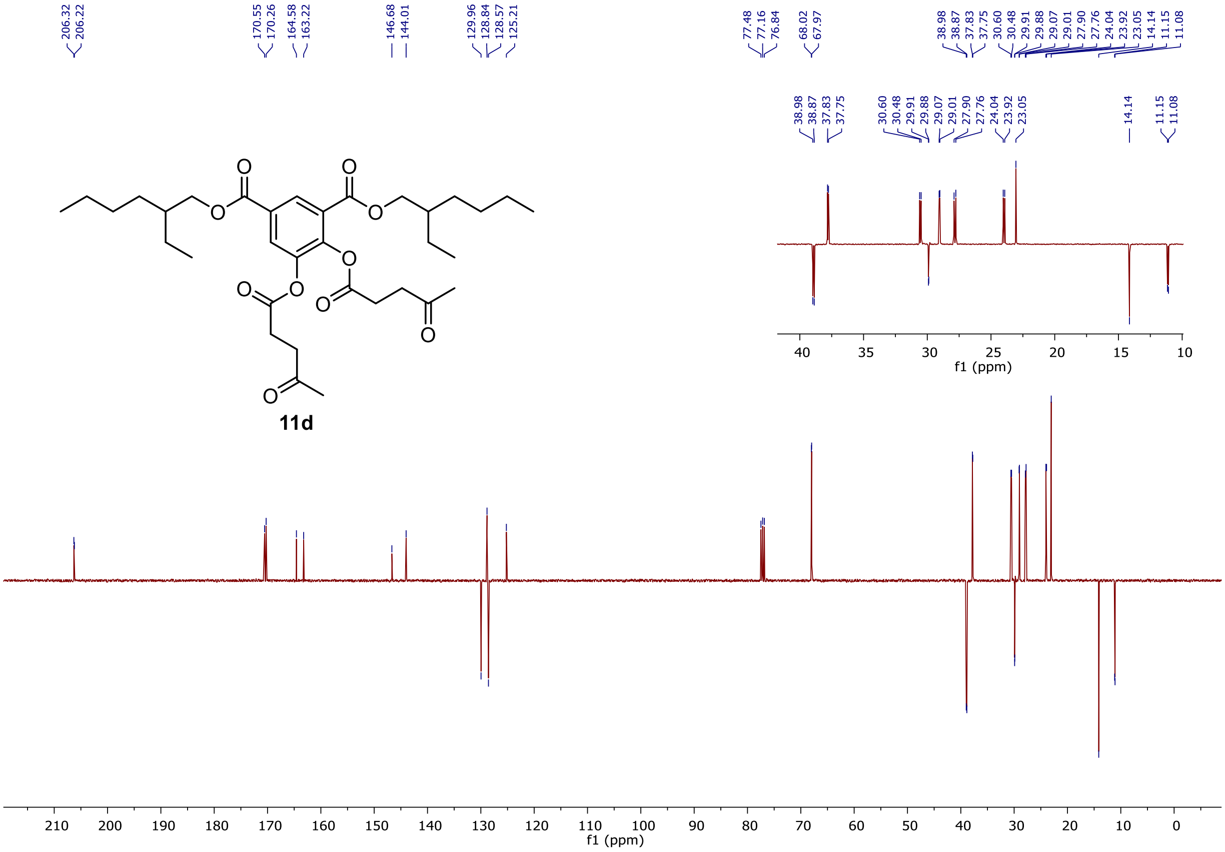

Figure S114: ^{13}C APT NMR (101 MHz, $CDCl_3$) spectrum of bis(2-ethylhexyl) 4,5-bis[(4-oxopentanoyl)oxy]benzene-1,3-dicarboxylate (11d) [AGO-139].

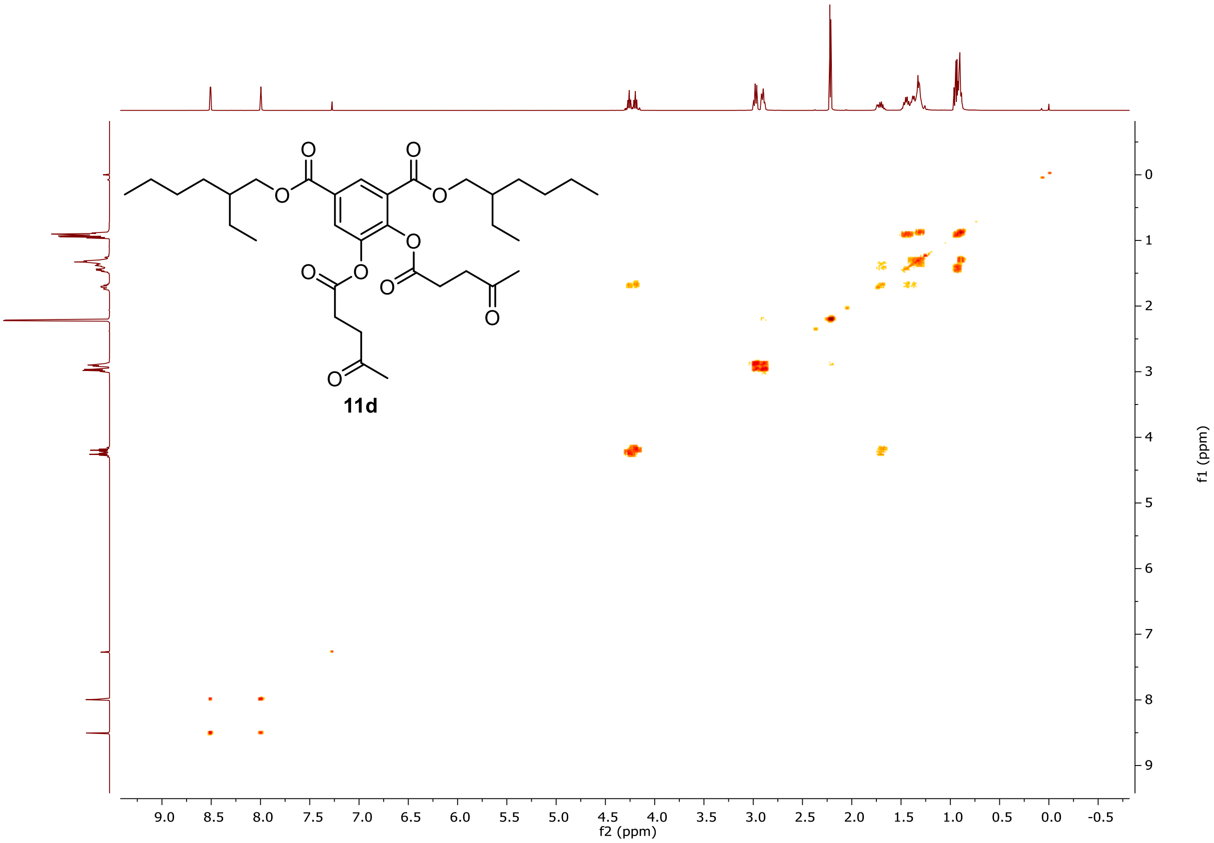

Figure S115: ^{1}H COSY NMR (400 MHz, $CDCl_3$) spectrum of bis(2-ethylhexyl) 4,5-bis[(4-oxopentanoyl)oxy]benzene-1,3-dicarboxylate (11d) [AGO-139].

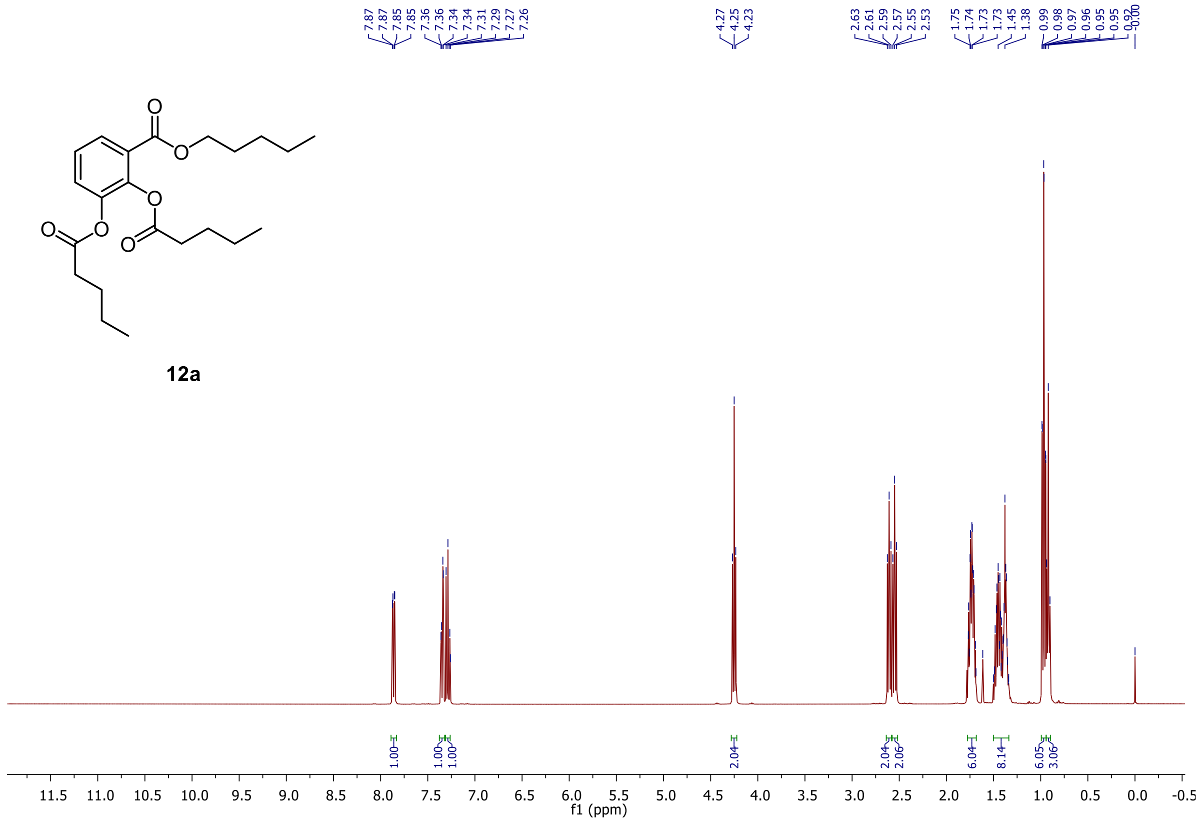

Figure S116: ^{1}**H NMR (400 MHz, CDCl$_3$) spectrum of pentyl 2,3-bis(pentanoyloxy)benzoate (12a) [DDV-AG-036].**

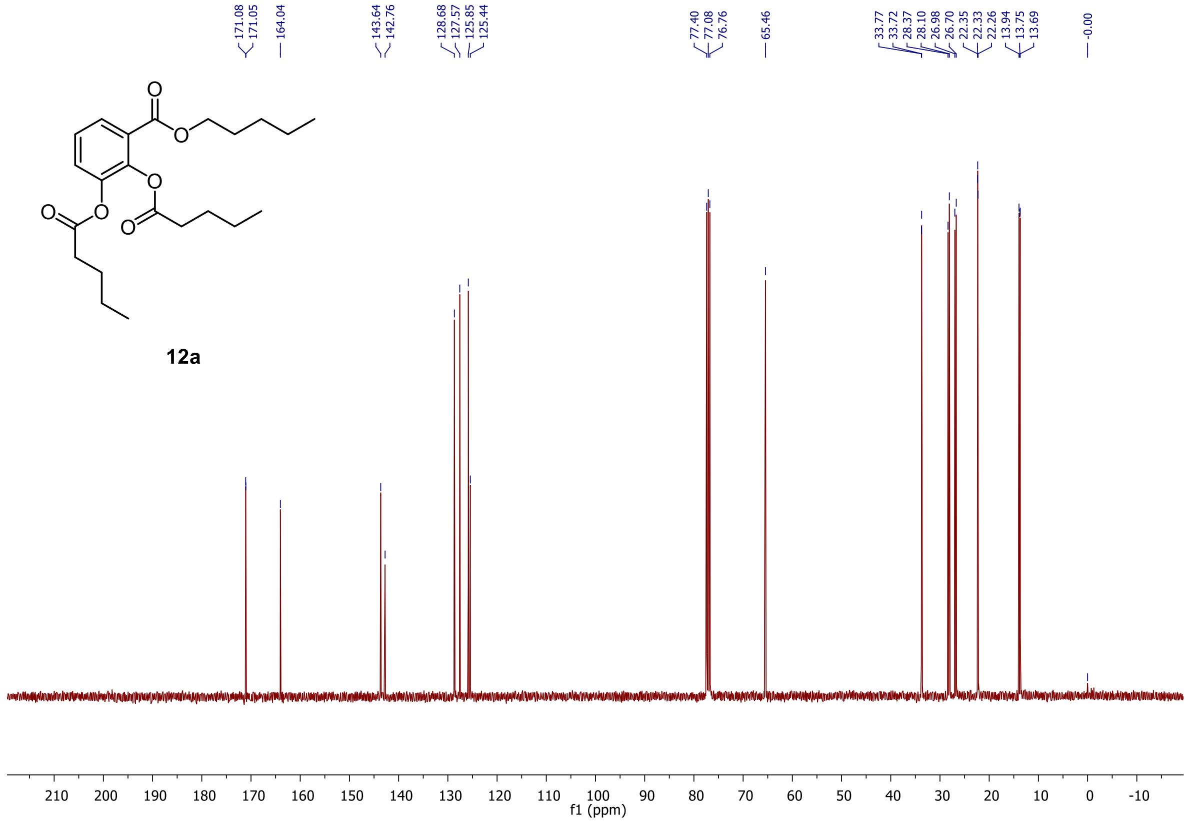

Figure S117: ^{13}**C{^{1}H} NMR (101 MHz, CDCl$_3$) spectrum of pentyl 2,3-bis(pentanoyloxy)benzoate (12a) [DDV-AG-036].**

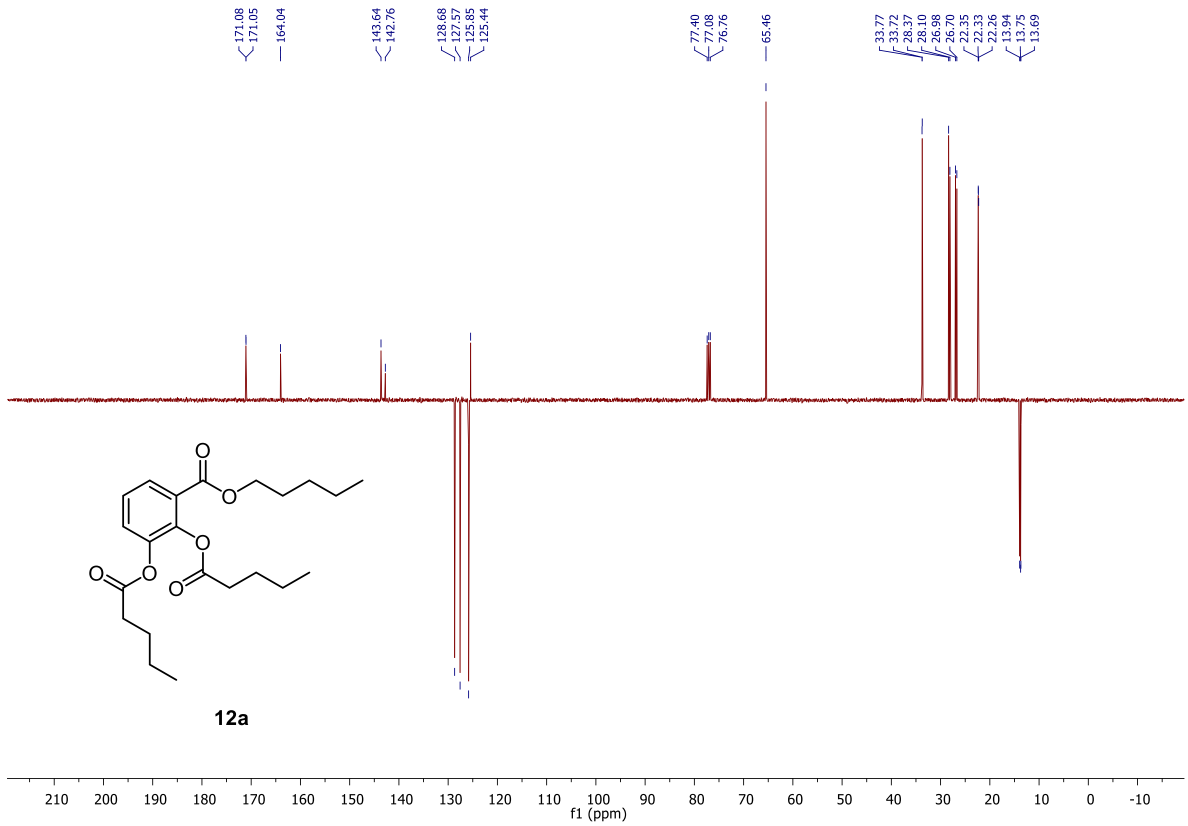

Figure S118: ^{13}C APT NMR (101 MHz, CDCl$_3$) spectrum of pentyl 2,3-bis(pentanoyloxy)benzoate (12a) [DDV-AG-036].

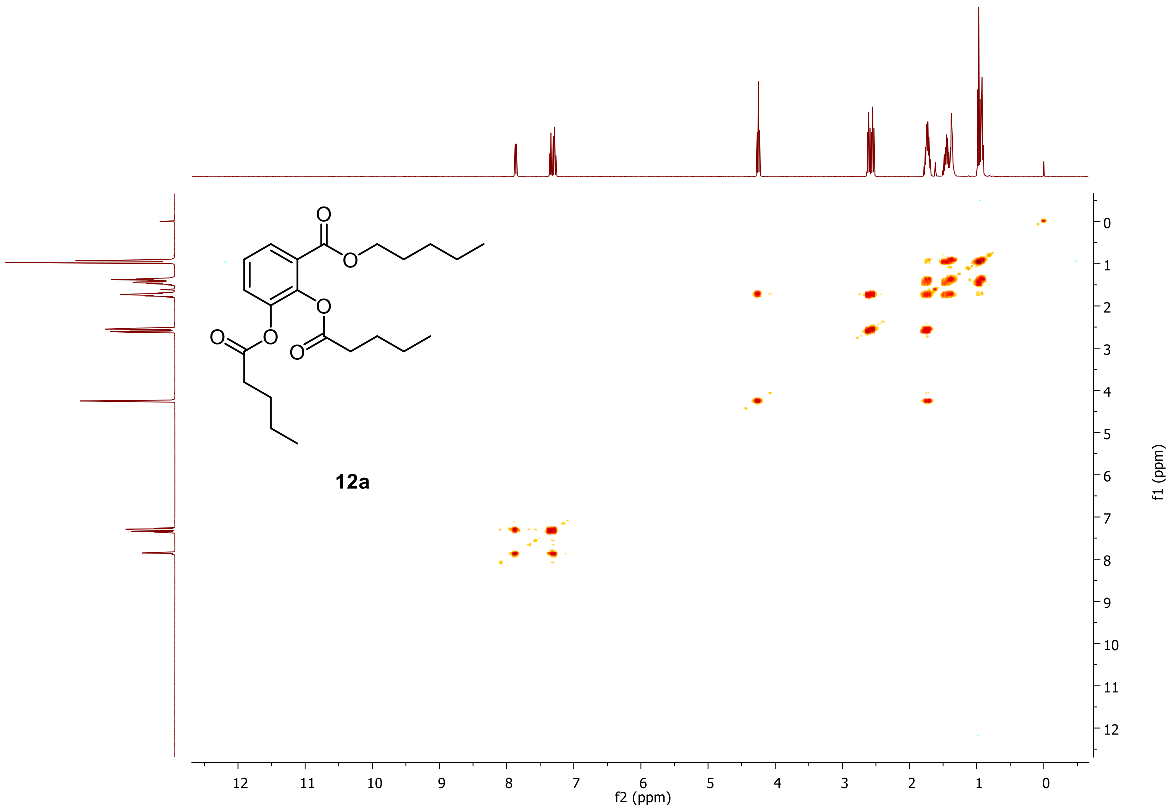

Figure S119: ^{1}H COSY NMR (400 MHz, CDCl$_3$) spectrum of pentyl 2,3-bis(pentanoyloxy)benzoate (12a) [DDV-AG-036].

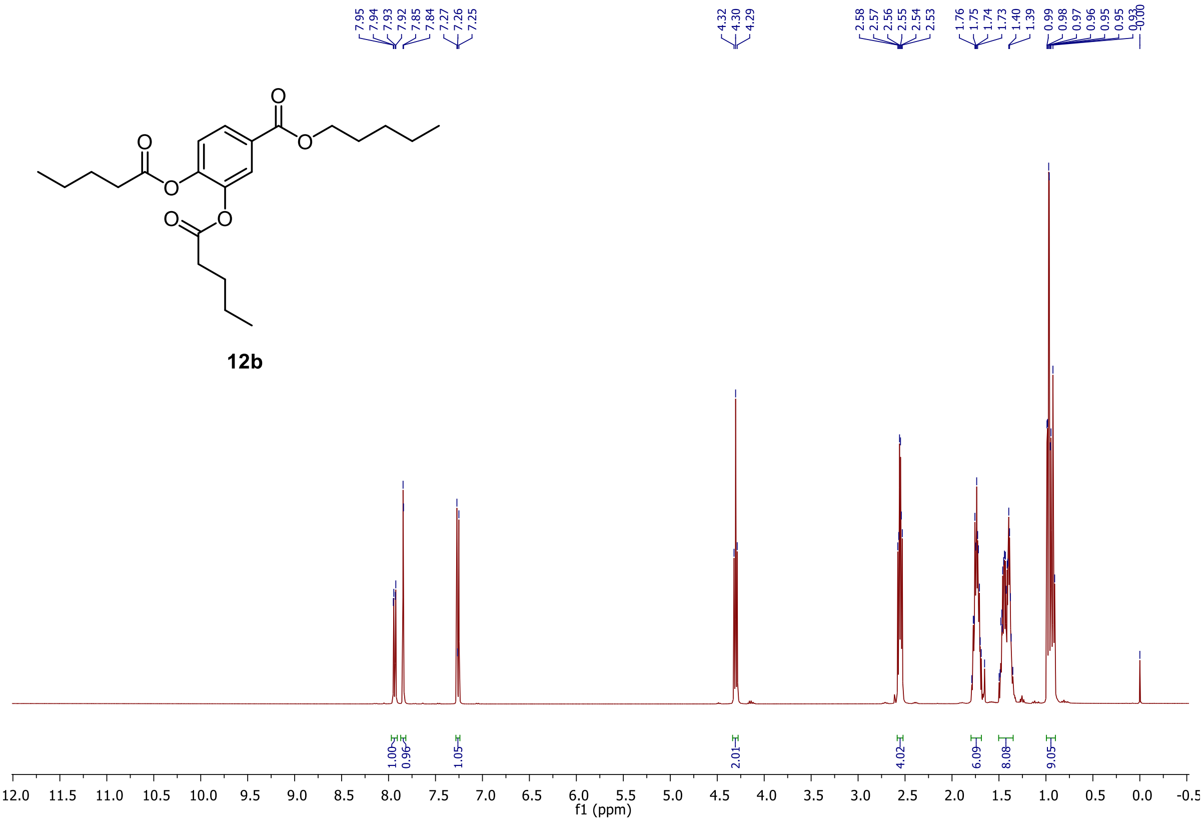

Figure S120: ^{1}H NMR (400 MHz, CDCl$_3$) spectrum of pentyl 3,4-bis(pentanoyloxy)benzoate (12b) [DDV-AG-038].

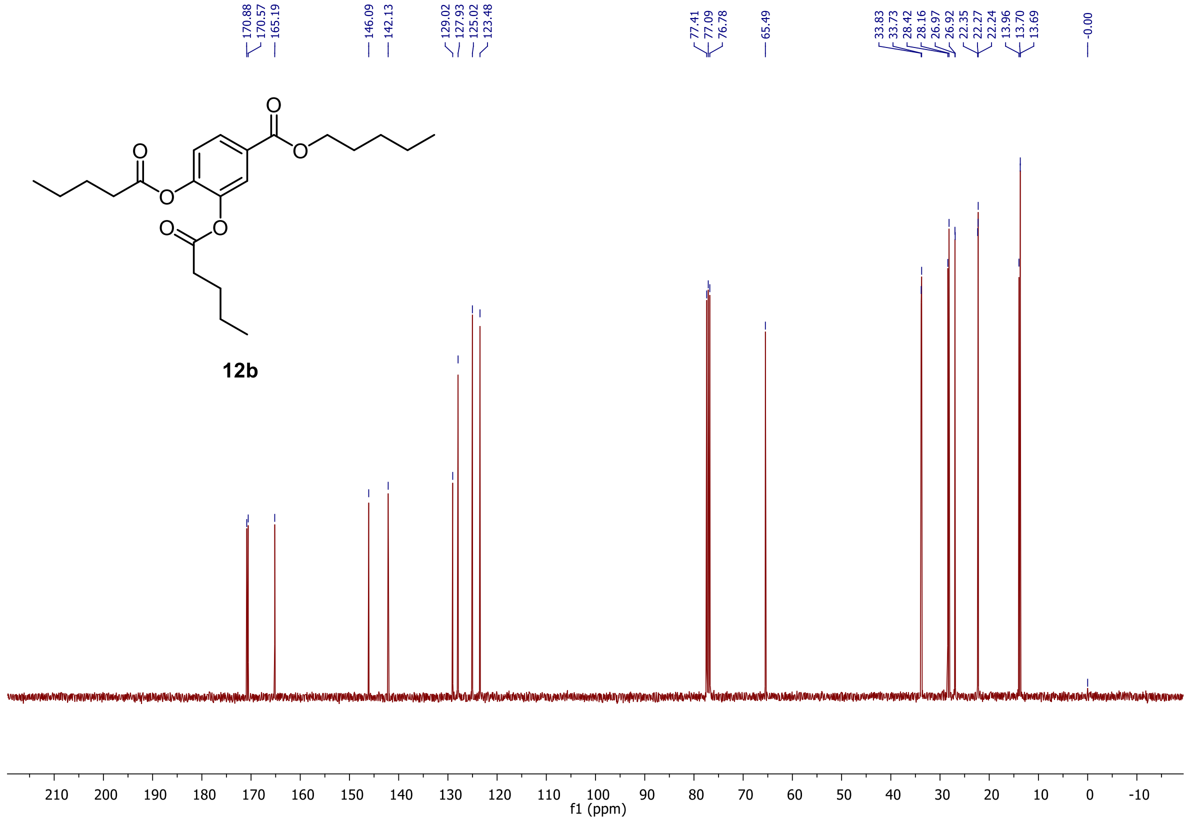

Figure S121: ^{13}C{^{1}H} NMR (101 MHz, CDCl$_3$) spectrum of pentyl 3,4-bis(pentanoyloxy)benzoate (12b) [DDV-AG-038].

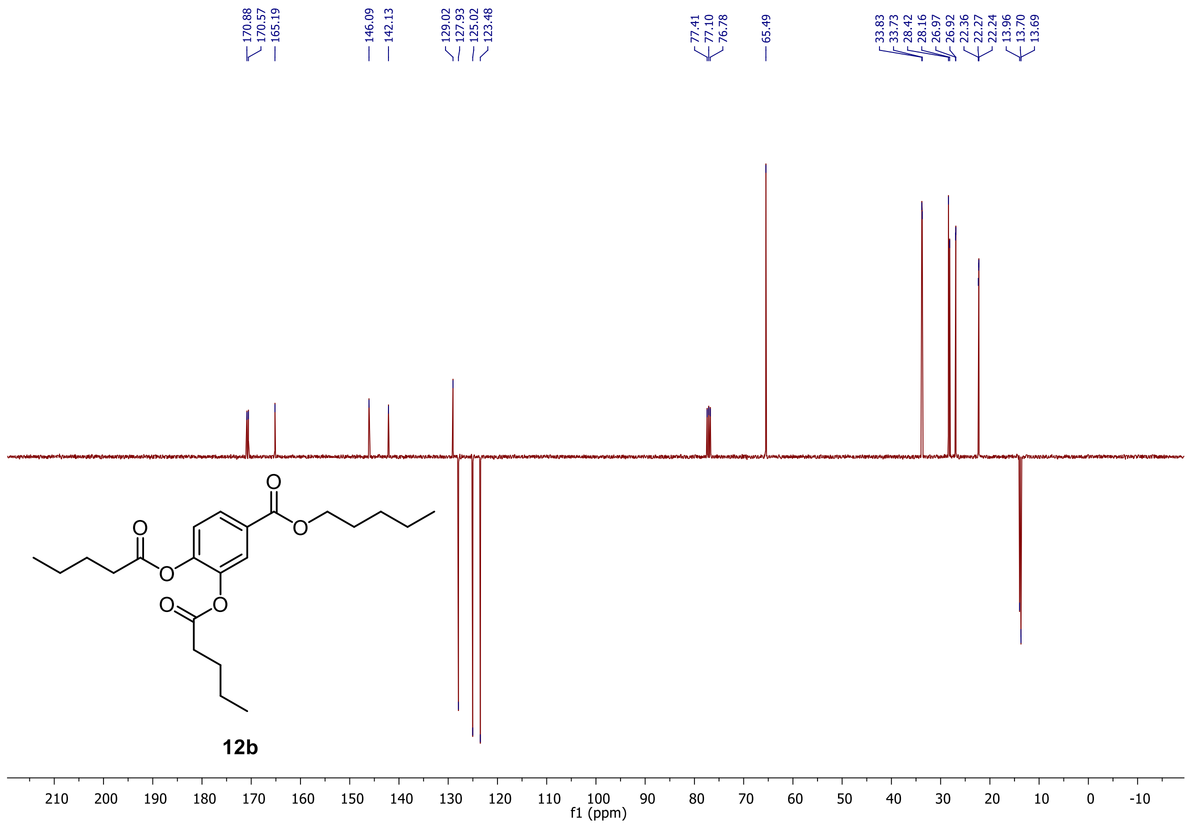

Figure S122: ^{13}C APT NMR (101 MHz, $CDCl_3$) spectrum of pentyl 3,4-bis(pentanoyloxy)benzoate (12b) [DDV-AG-038].

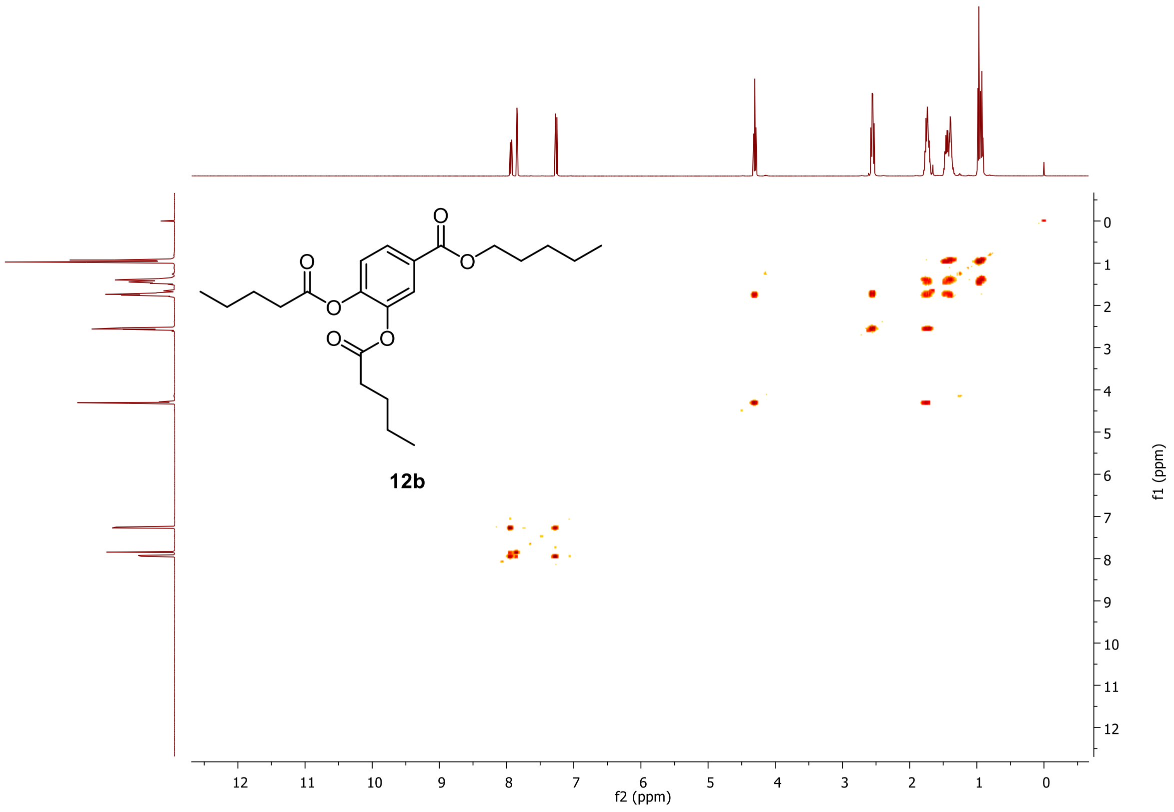

Figure S123: ^{1}H COSY NMR (400 MHz, $CDCl_3$) spectrum of pentyl 3,4-bis(pentanoyloxy)benzoate (12b) [DDV-AG-038].

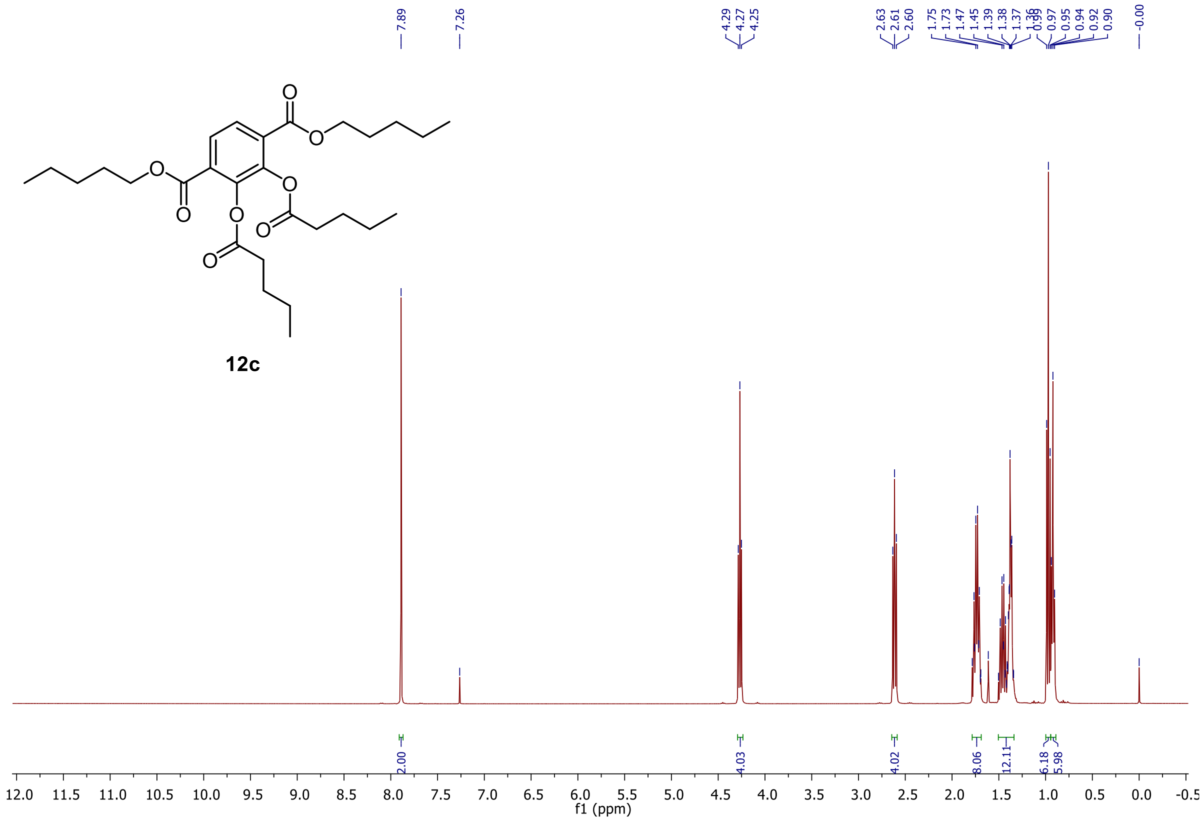

Figure S124: ^{1}H NMR (400 MHz, $CDCl_3$) spectrum of dipentyl 2,3-bis(pentanoyloxy)benzene-1,4-dicarboxylate (12c) [DDV-AG-037].

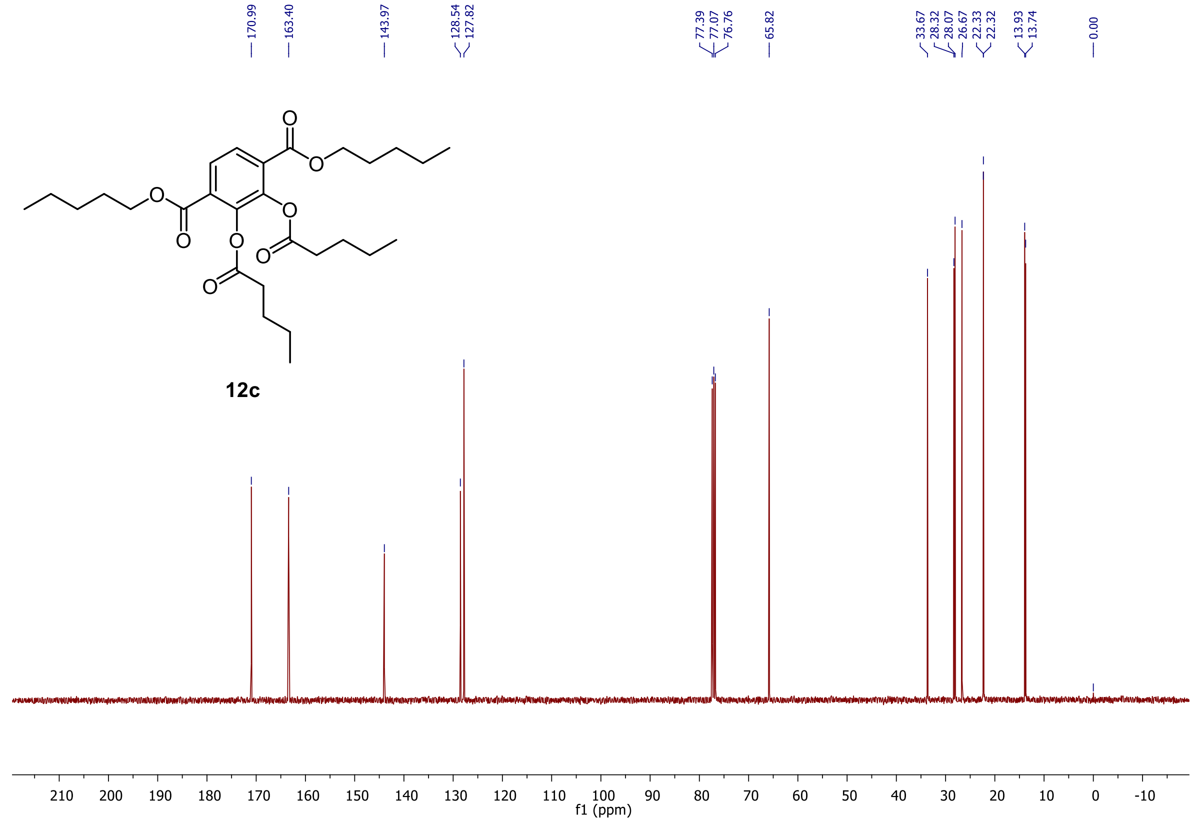

Figure S125: $^{13}C\{^{1}H\}$ NMR (101 MHz, $CDCl_3$) spectrum of dipentyl 2,3-bis(pentanoyloxy)benzene-1,4-dicarboxylate (12c) [DDV-AG-037].

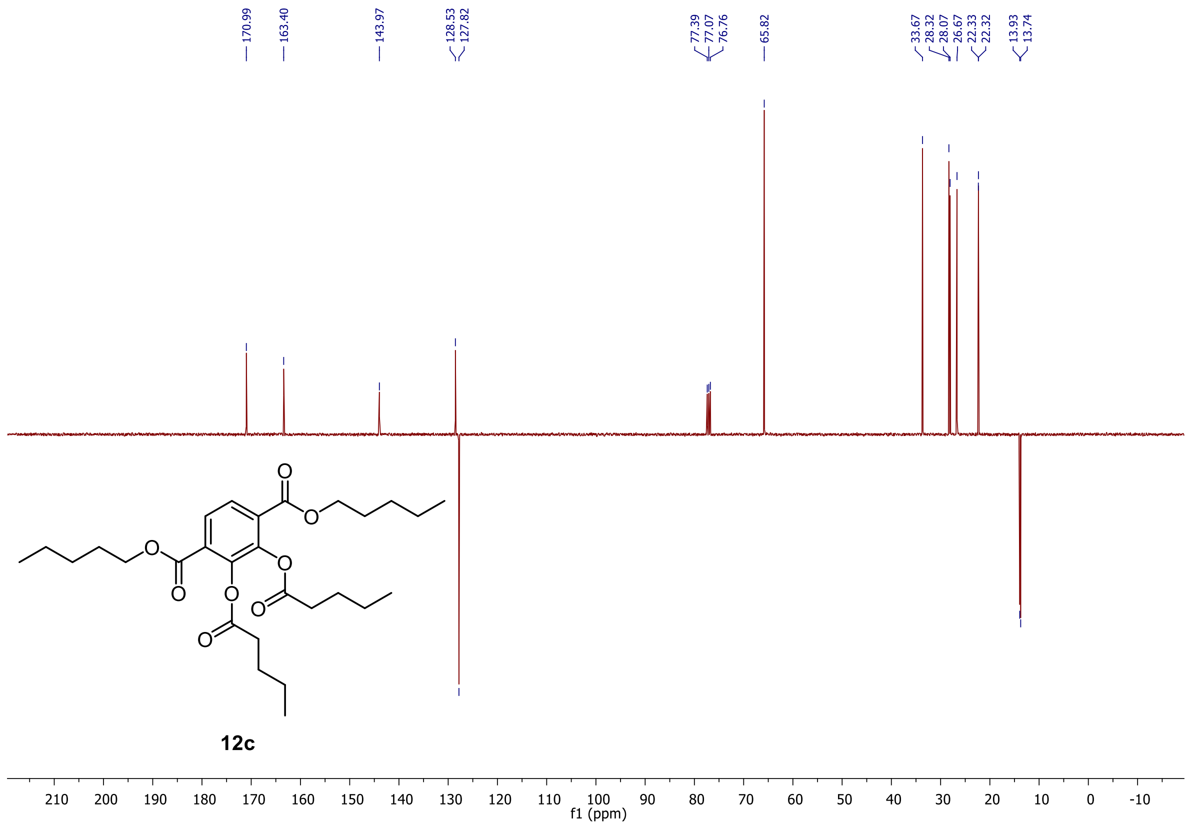

Figure S126: ^{13}C APT NMR (101 MHz, $CDCl_3$) spectrum of dipentyl 2,3-bis(pentanoyloxy)benzene-1,4-dicarboxylate (12c) [DDV-AG-037].

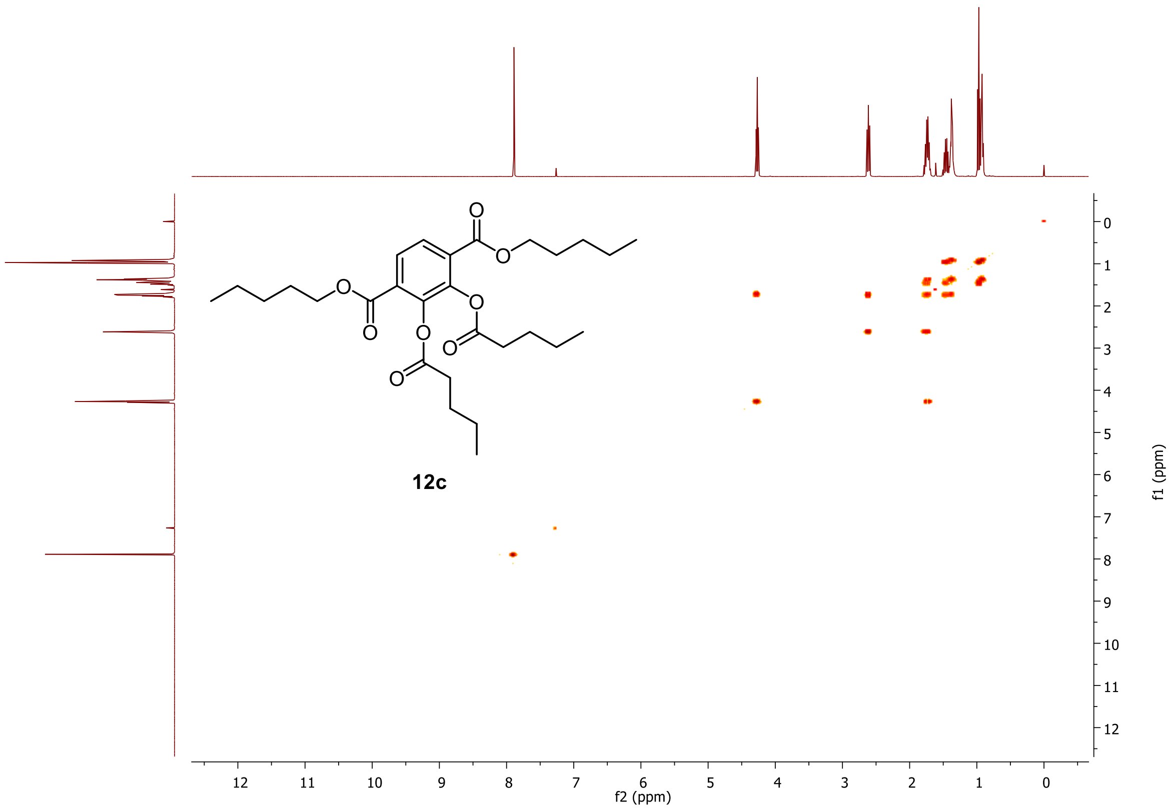

Figure S127: ^{1}H COSY NMR (400 MHz, $CDCl_3$) spectrum of dipentyl 2,3-bis(pentanoyloxy)benzene-1,4-dicarboxylate (12c) [DDV-AG-037].

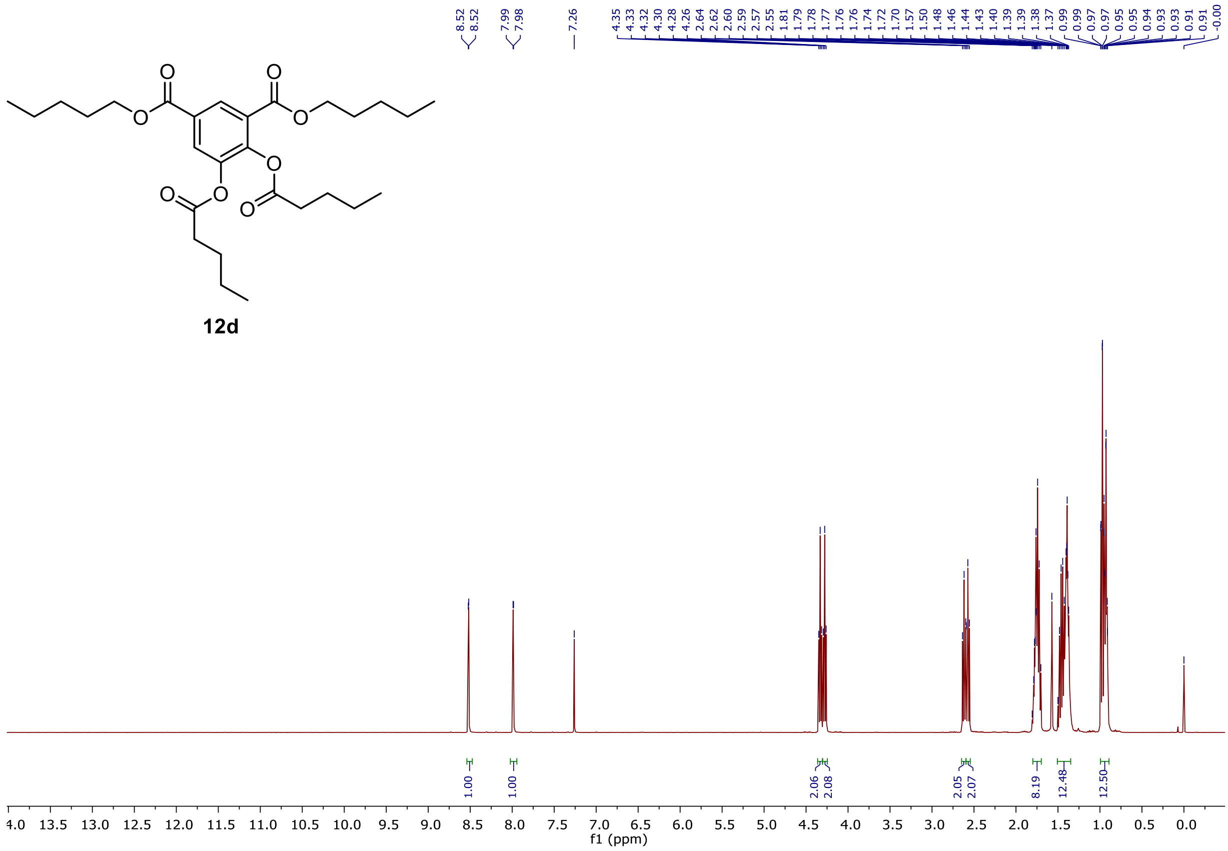

Figure S128: ^{1}H NMR (400 MHz, $CDCl_3$) spectrum of bis(2-ethylhexyl) 4,5-bis[(4-oxopentanoyl)oxy]benzene-1,3-dicarboxylate (12d) [AGO-131].

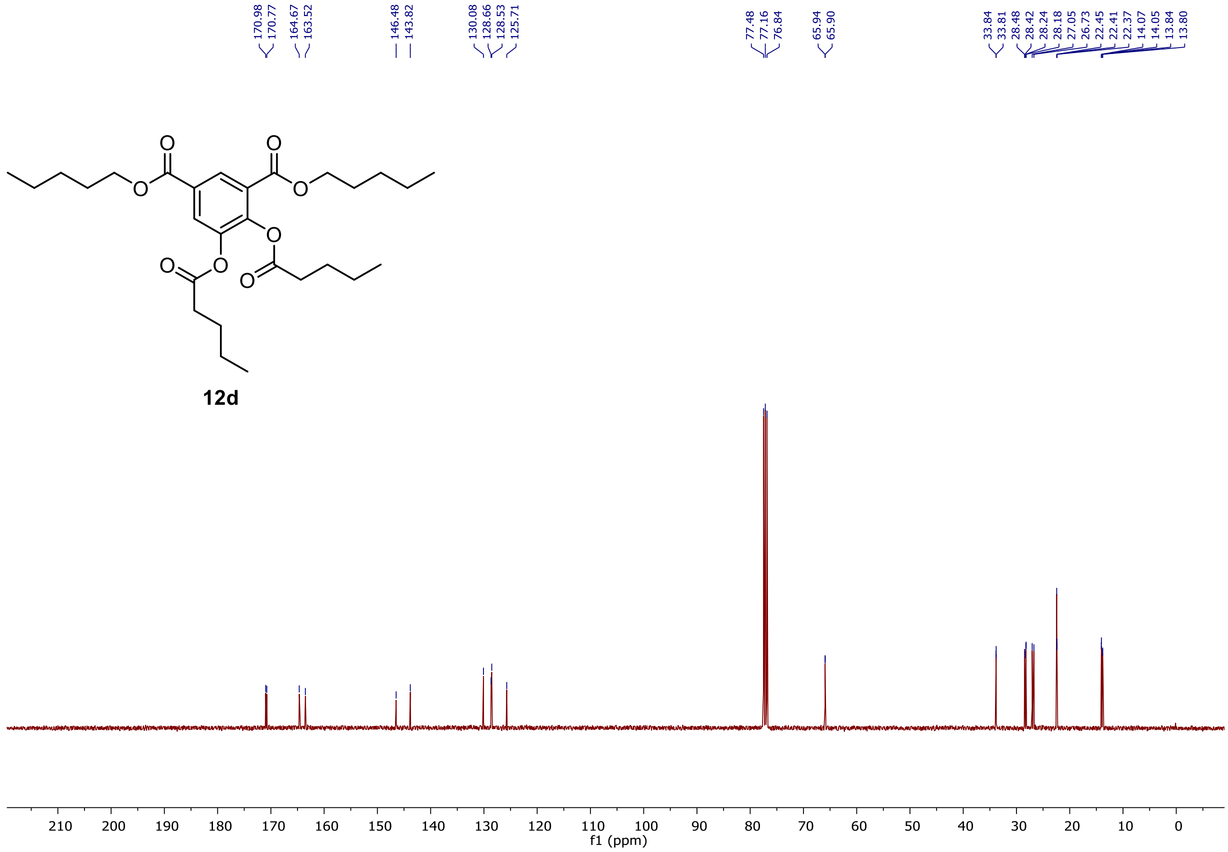

Figure S129: $^{13}C\{^1H\}$ NMR (101 MHz, $CDCl_3$) spectrum of bis(2-ethylhexyl) 4,5-bis[(4-oxopentanoyl)oxy]benzene-1,3-dicarboxylate (12d) [AGO-131].

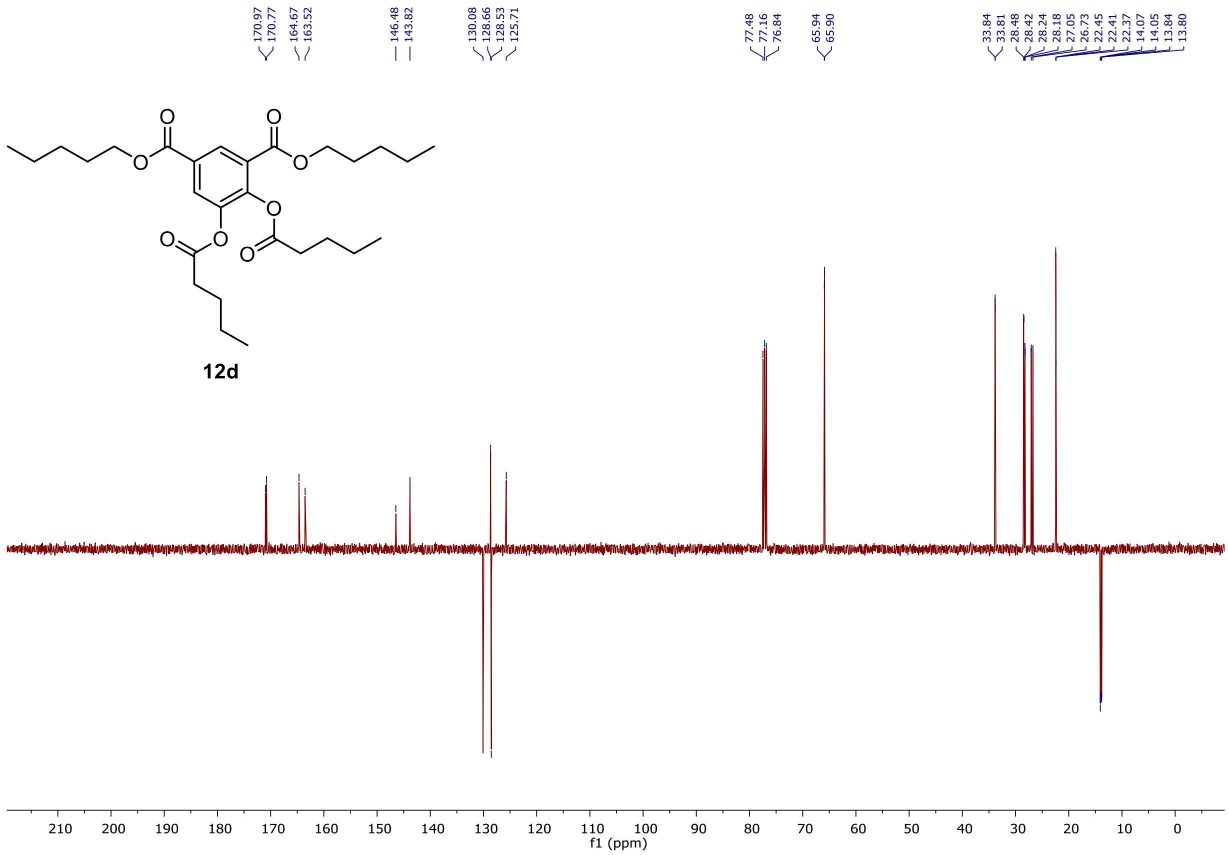

Figure S130: ^{13}C APT NMR (101 MHz, $CDCl_3$) spectrum of bis(2-ethylhexyl) 4,5-bis[(4-oxopentanoyl)oxy]benzene-1,3-dicarboxylate (12d) [AGO-131].

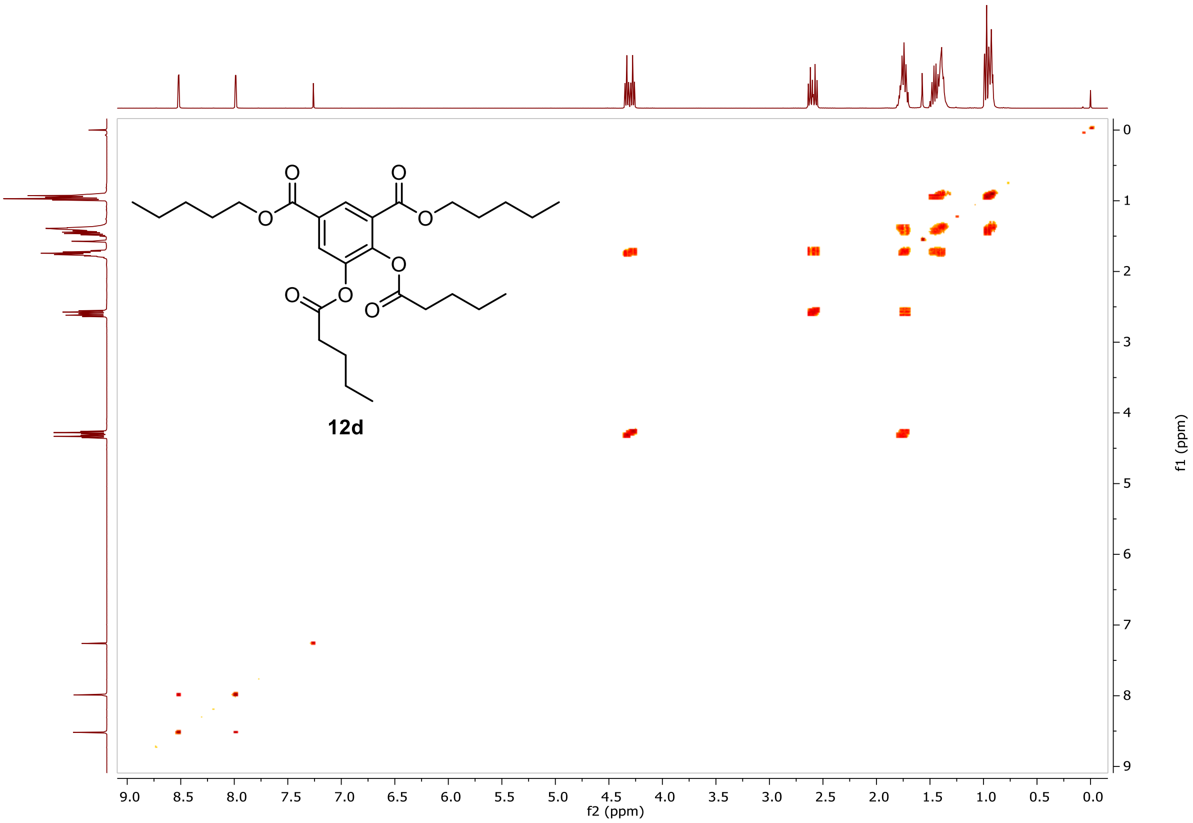

Figure S131: ^{1}H COSY NMR (400 MHz, $CDCl_3$) spectrum of bis(2-ethylhexyl) 4,5-bis[(4-oxopentanoyl)oxy]benzene-1,3-dicarboxylate (12d) [AGO-131].

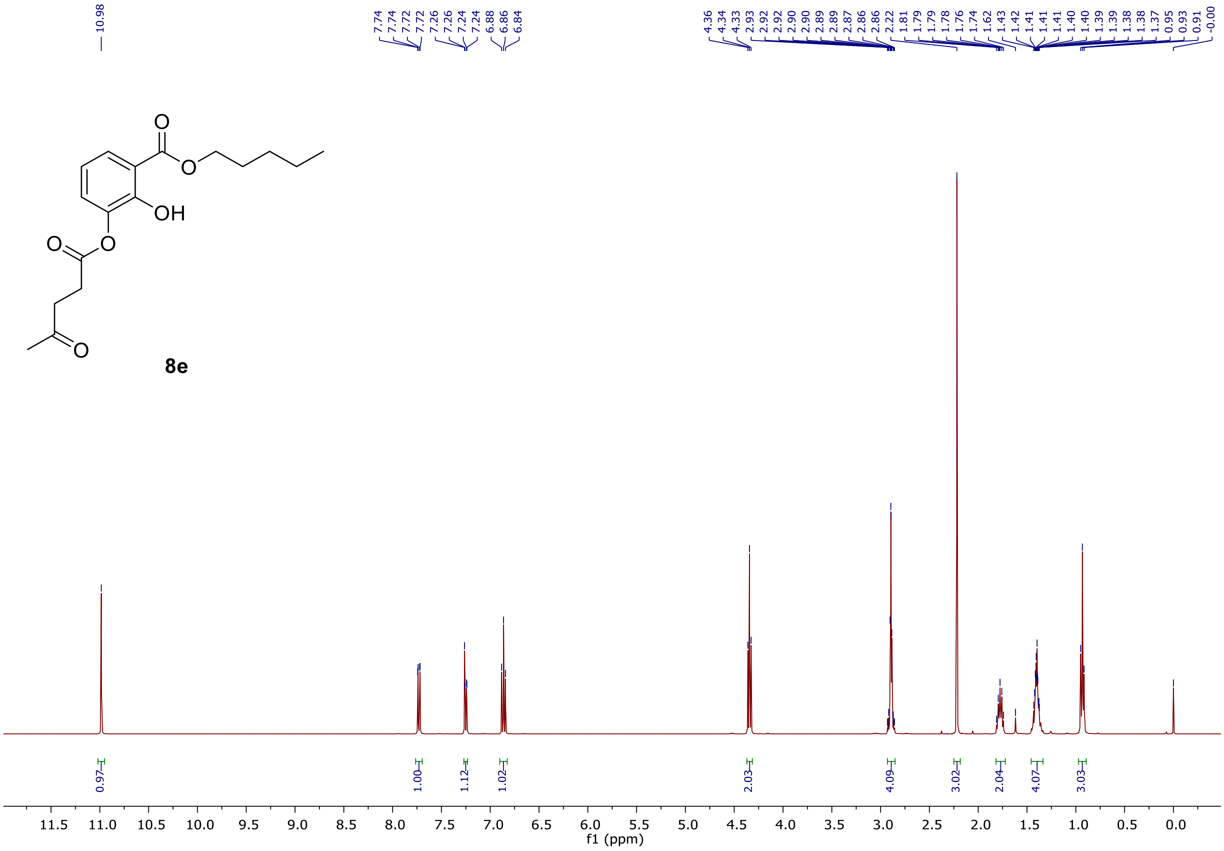

Figure S132: ^{1}H NMR (400 MHz, $CDCl_3$) spectrum of pentyl 2-hydroxy-3-((4-oxopentanoyl)oxy)benzoate (8e) [AGO-076].

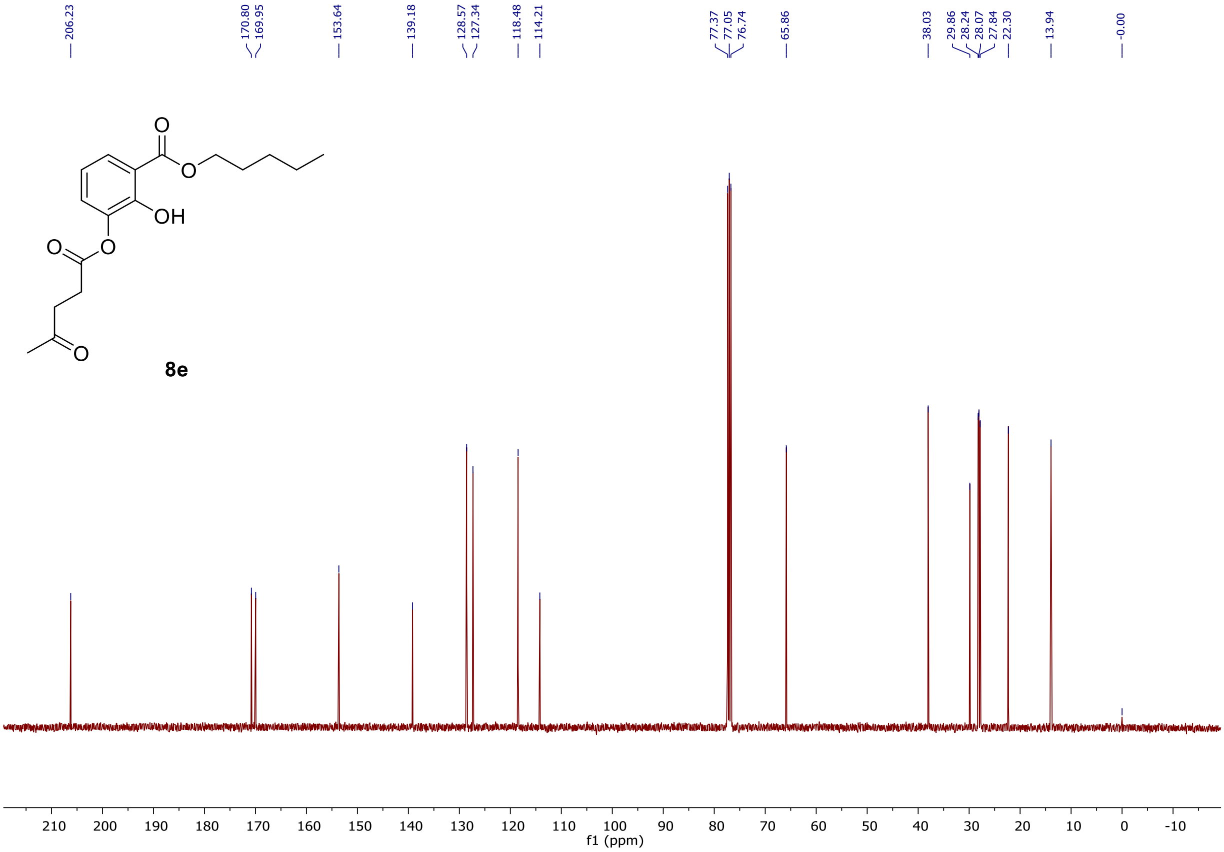

Figure S133: $^{13}C\{^{1}H\}$ NMR (101 MHz, $CDCl_3$) spectrum of pentyl 2-hydroxy-3-((4-oxopentanoyl)oxy)benzoate (8e) [AGO-076].

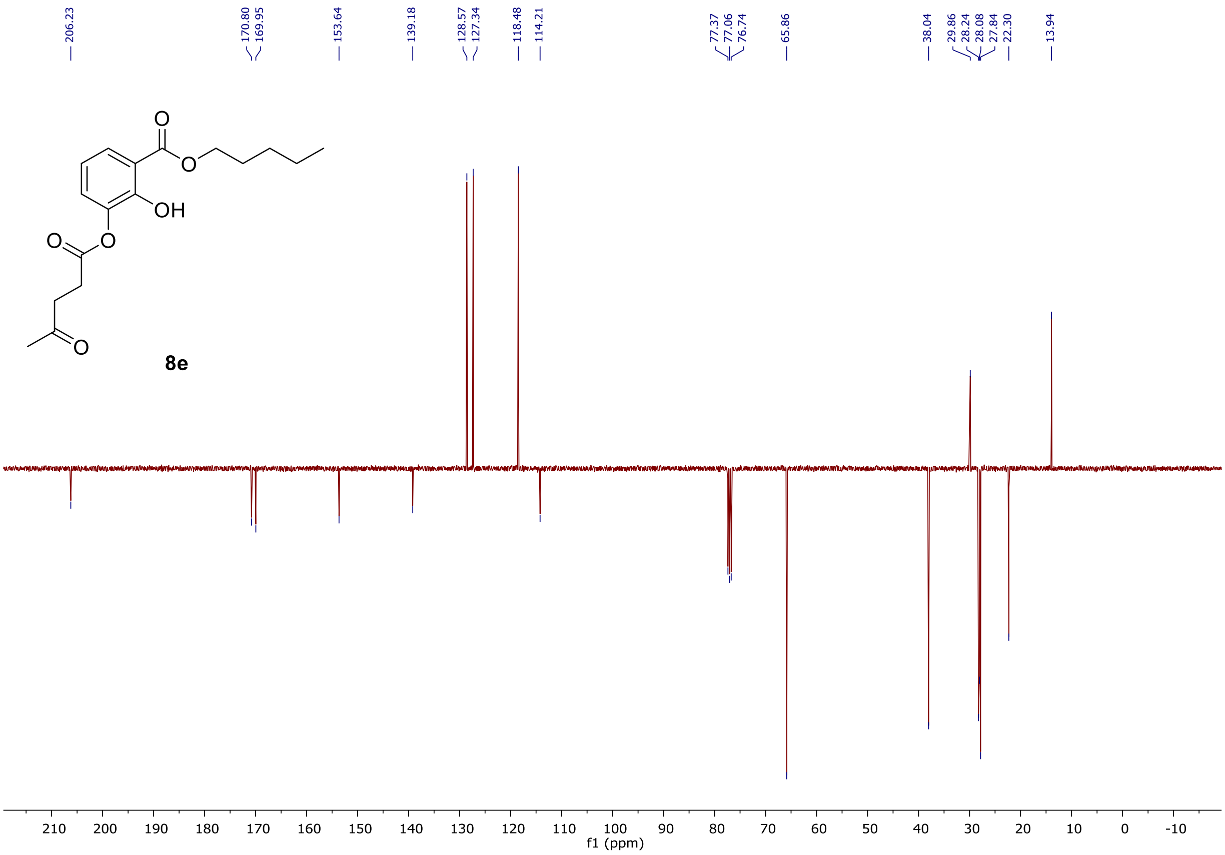

Figure S134: ^{13}C APT NMR (101 MHz, $CDCl_3$) spectrum of pentyl 2-hydroxy-3-((4-oxopentanoyl)oxy)benzoate (8e) [AGO-076].

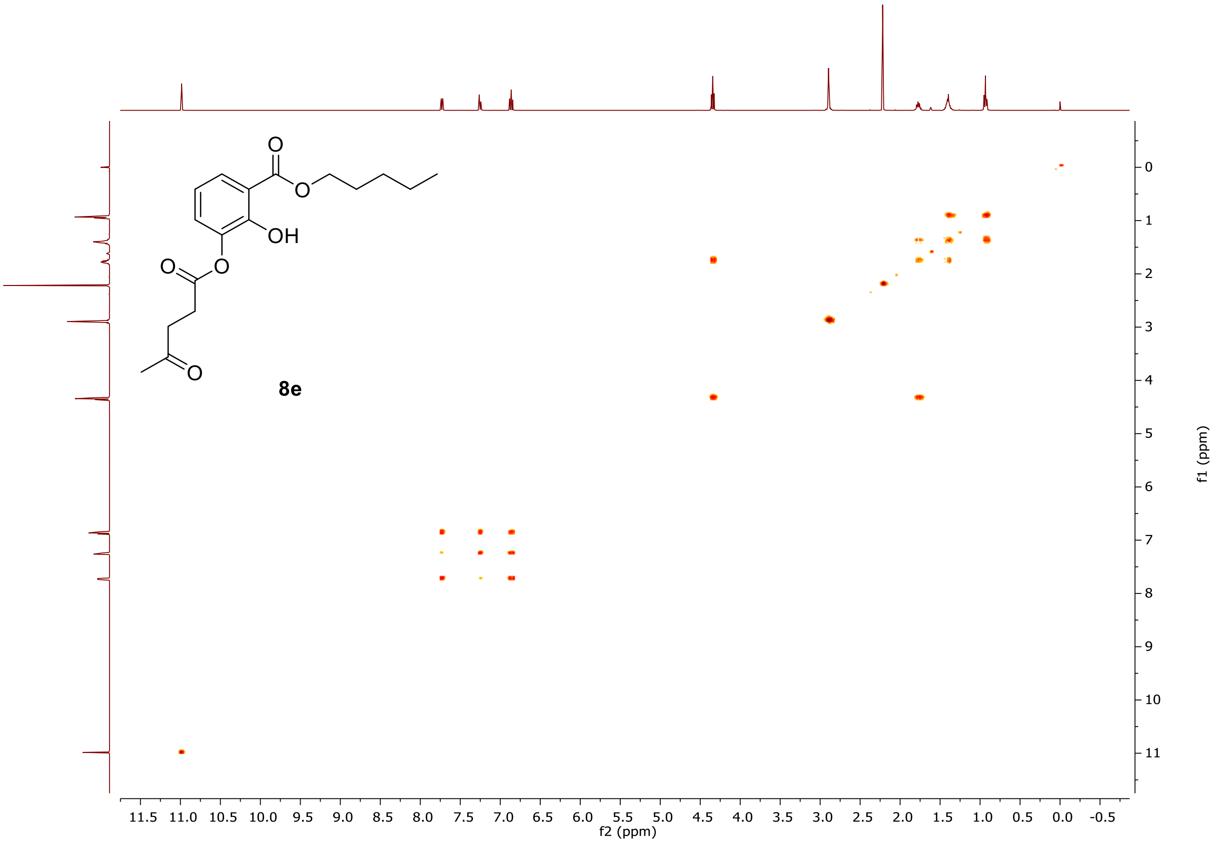

Figure S135: ^{1}H COSY NMR (400 MHz, $CDCl_3$) spectrum of pentyl 2-hydroxy-3-((4-oxopentanoyl)oxy)benzoate (8e) [AGO-076].

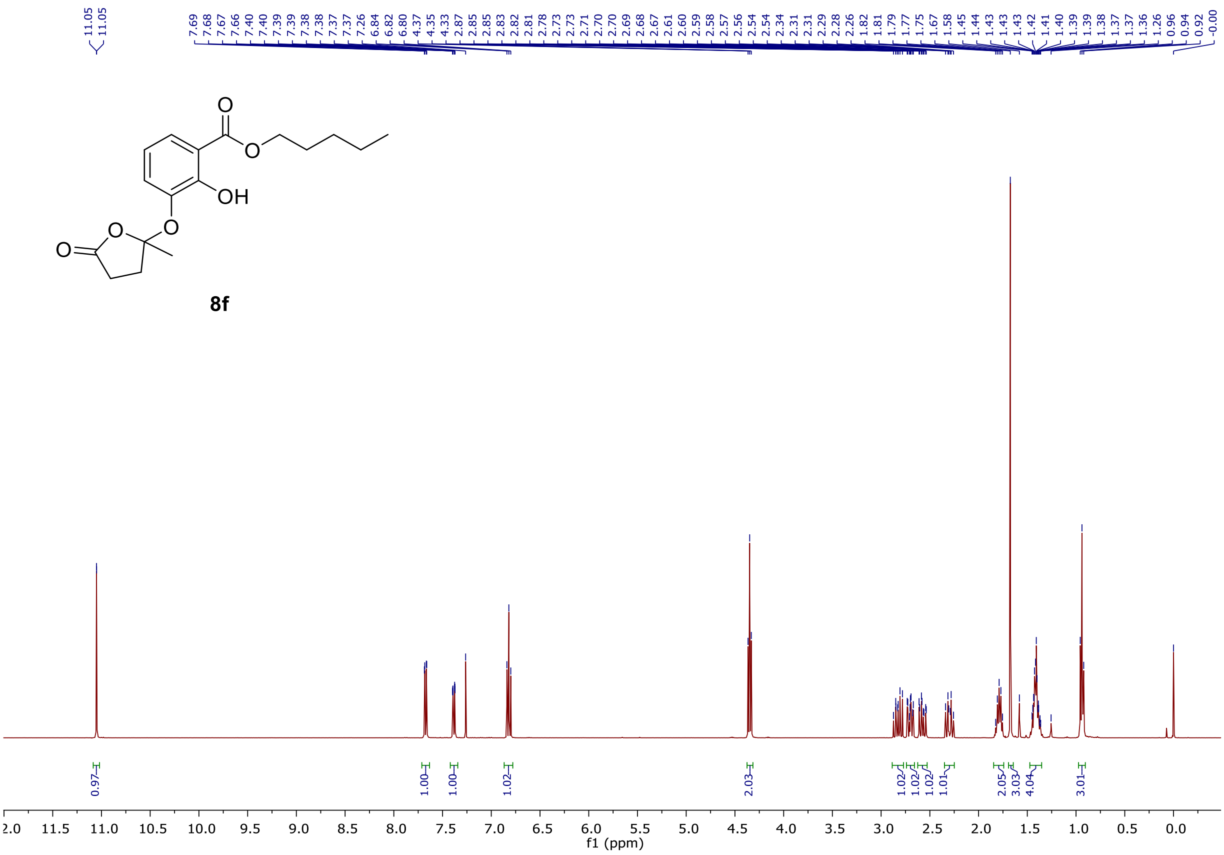

Figure S136: ^{1}H NMR (400 MHz, $CDCl_3$) spectrum of pentyl 2-hydroxy-3-((2-methyl-5-oxotetrahydrofuran-2-yl)oxy)benzoate (8f) [AGO-076].

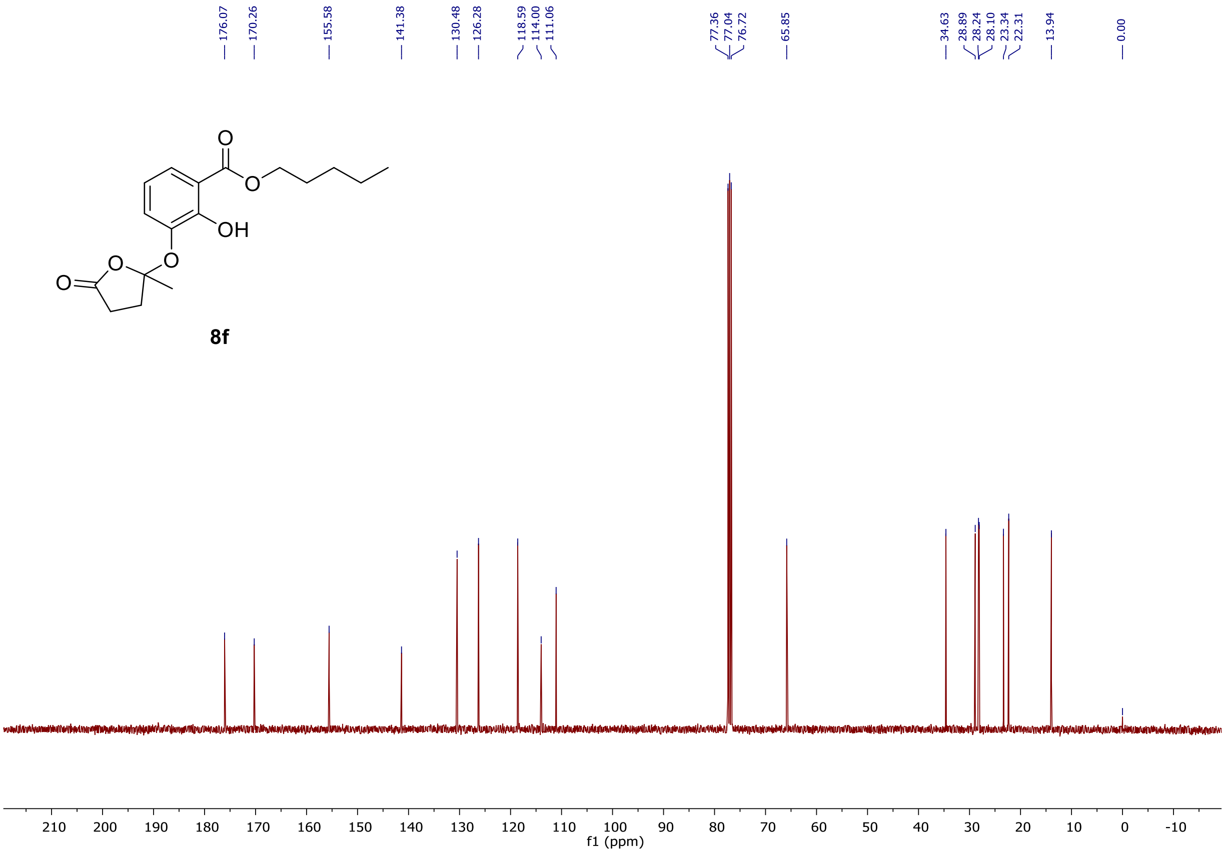

Figure S137: ^{13}C{^{1}H} NMR (101 MHz, $CDCl_3$) spectrum of pentyl 2-hydroxy-3-((2-methyl-5-oxotetrahydrofuran-2-yl)oxy)benzoate (8f) [AGO-076].

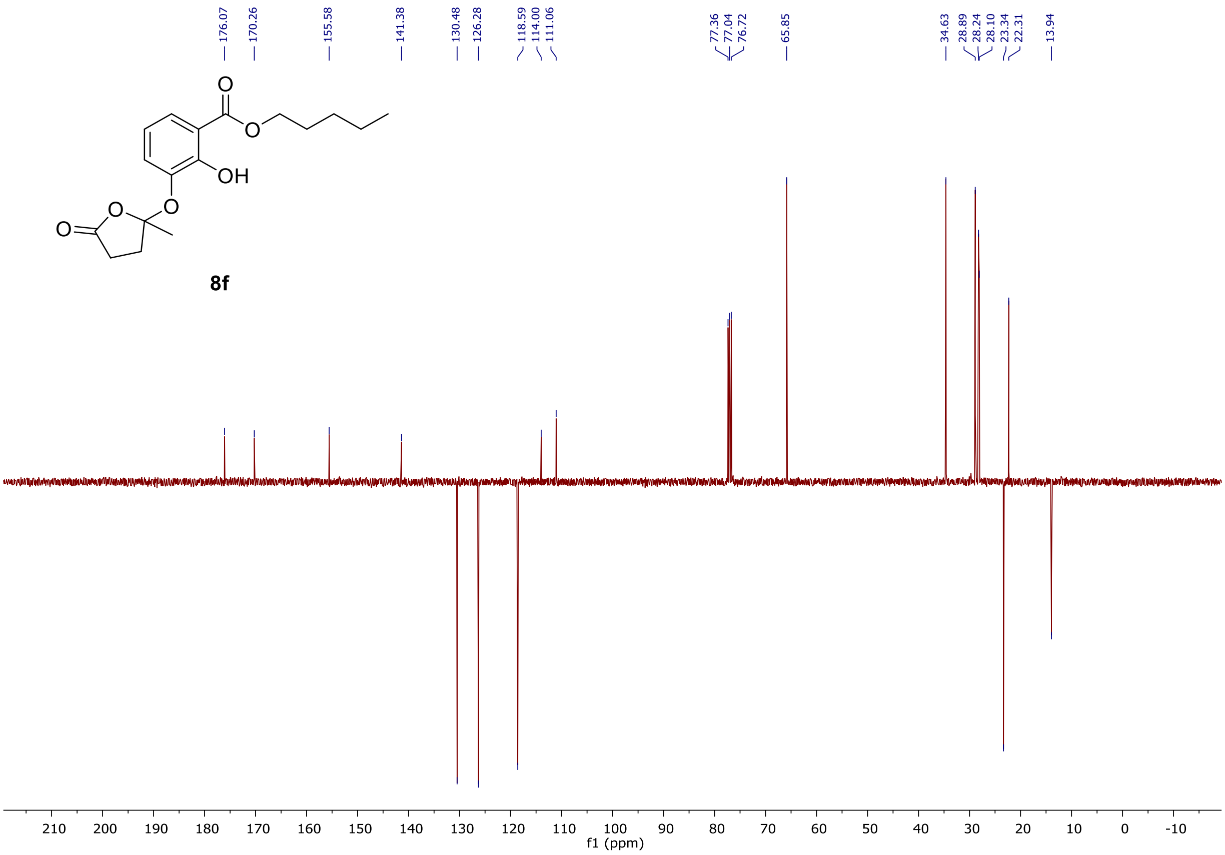

Figure S138: ^{13}C APT NMR (101 MHz, $CDCl_3$) spectrum of pentyl 2-hydroxy-3-((2-methyl-5-oxotetrahydrofuran-2-yl)oxy)benzoate (8f) [AGO-076].

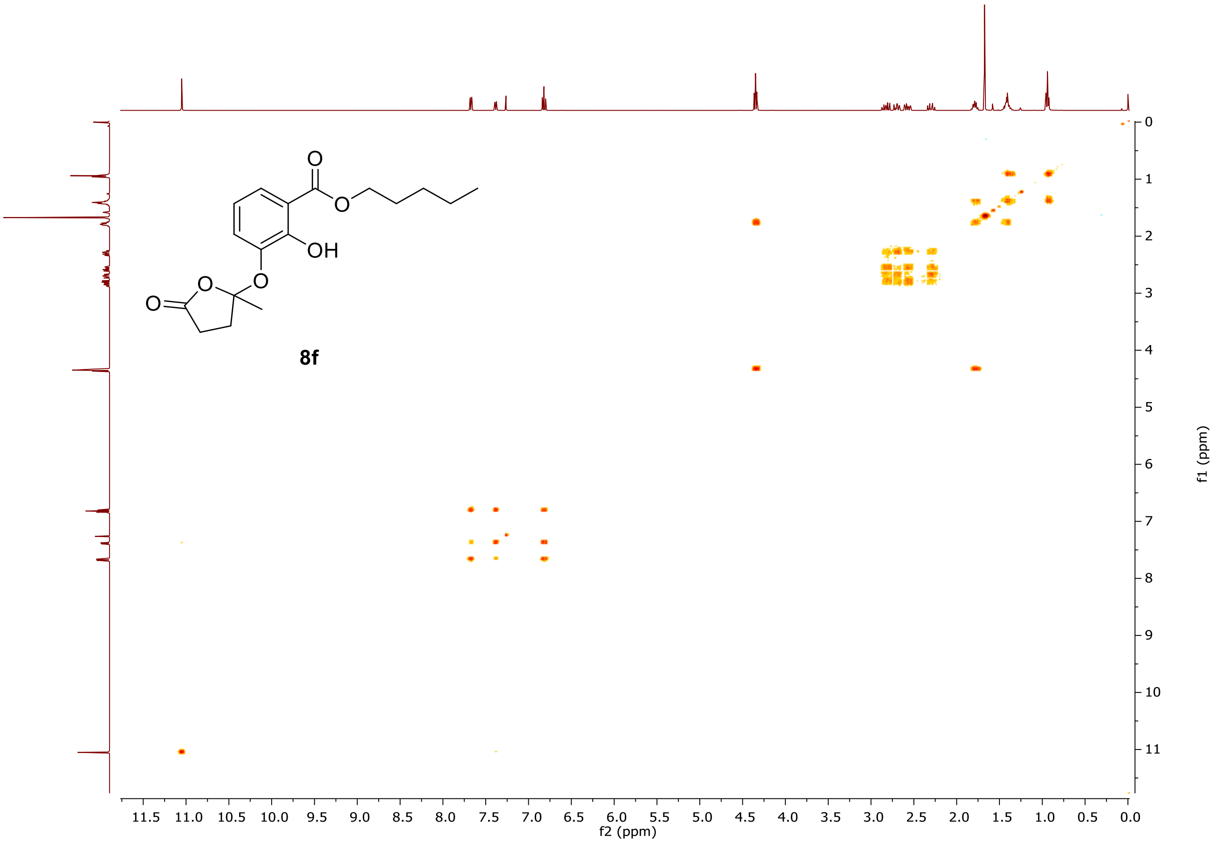

Figure S139: ^{1}H COSY NMR (400 MHz, $CDCl_3$) spectrum of pentyl 2-hydroxy-3-((2-methyl-5-oxotetrahydrofuran-2-yl)oxy)benzoate (8f) [AGO-076].

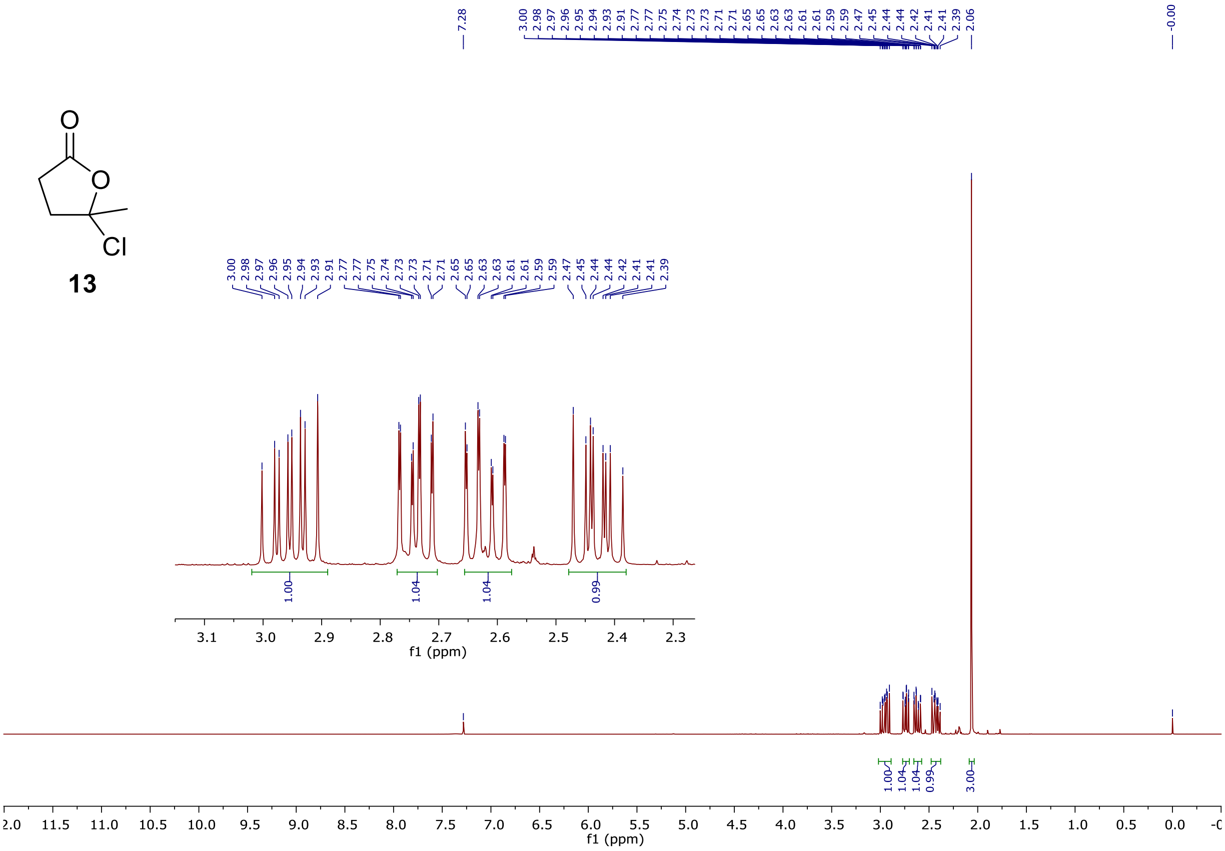

Figure S140: ^{1}H NMR (400 MHz, $CDCl_3$) spectrum of 5-chloro-5-methyldihydrofuran-2(3H)-one (13) [AGO-2024-0004].

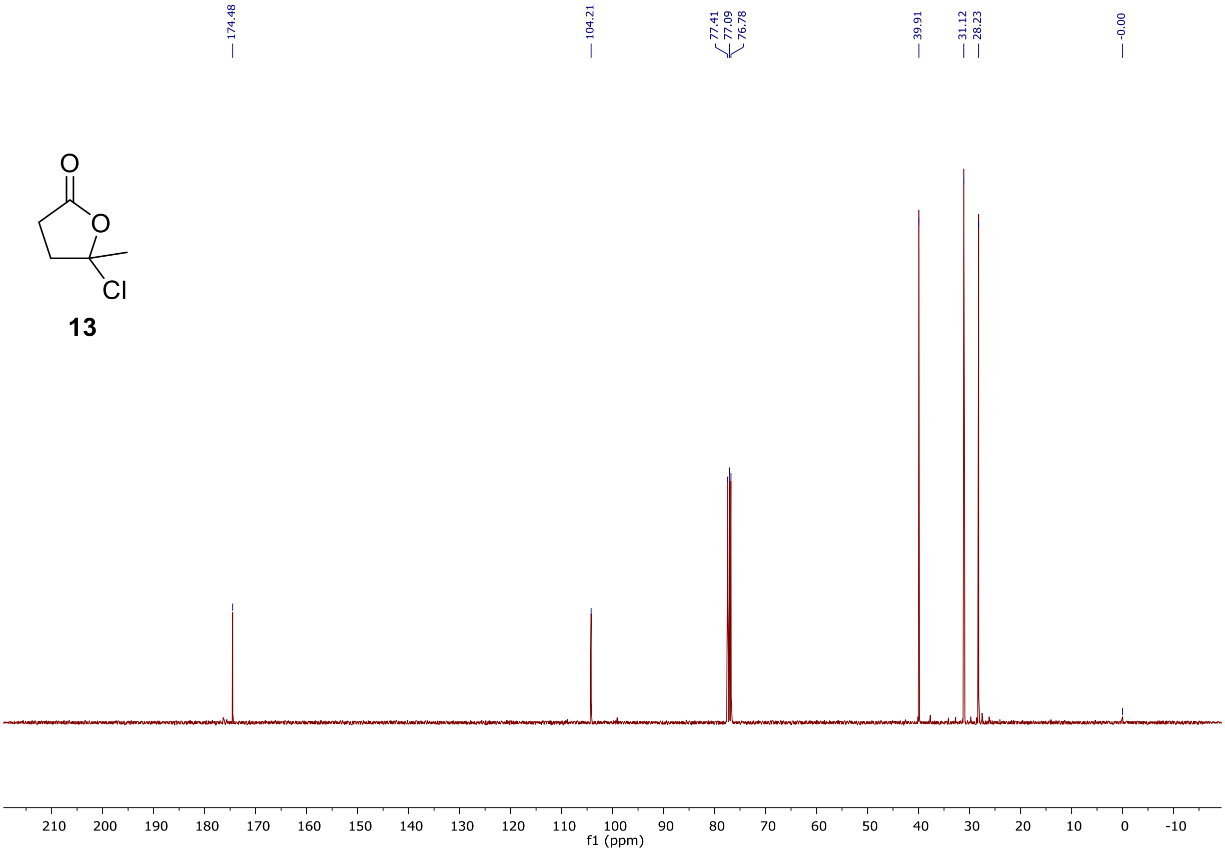

Figure S141: $^{13}C\{^{1}H\}$ NMR (101 MHz, $CDCl_3$) spectrum of 5-chloro-5-methyldihydrofuran-2(3H)-one (13) [AGO-2024-0004].

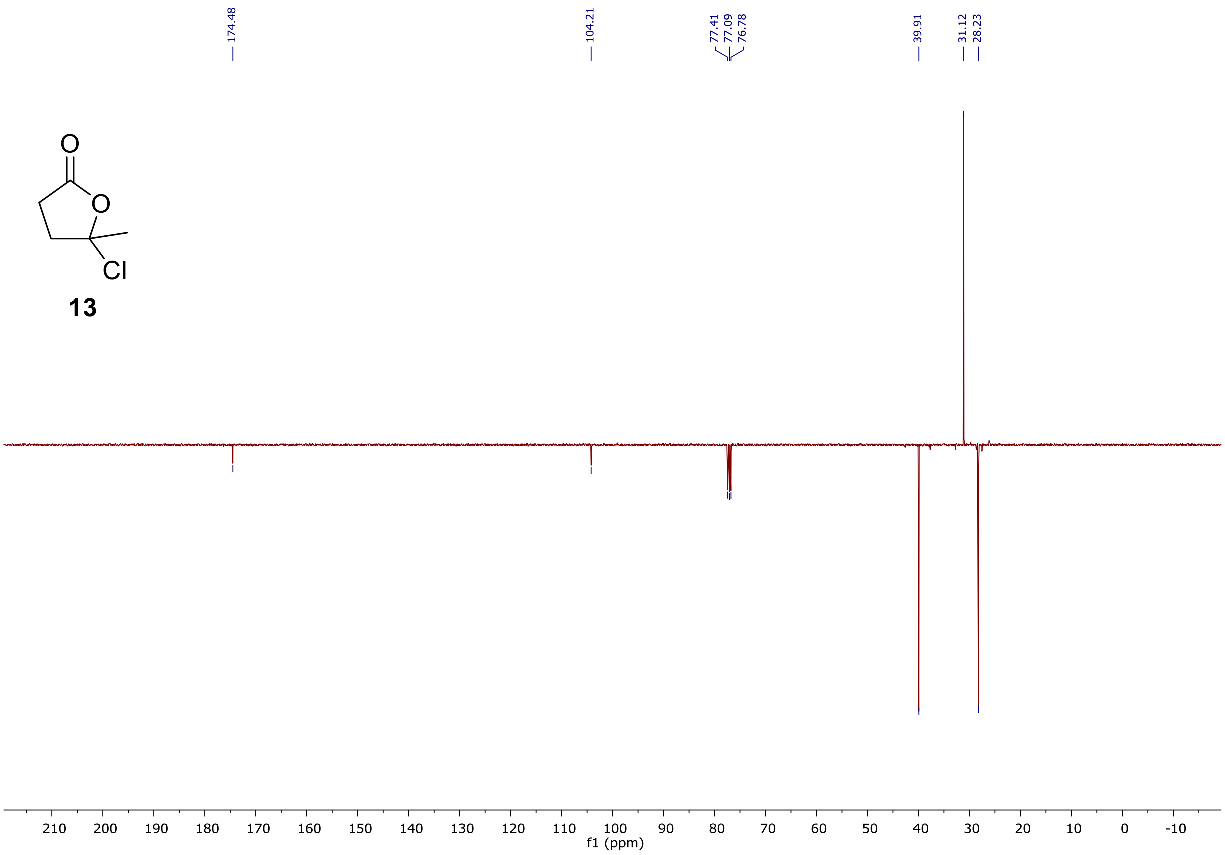

Figure S142: ^{13}C APT NMR (101 MHz, $CDCl_3$) spectrum of 5-chloro-5-methyldihydrofuran-2(3H)-one (13) [AGO-2024-0004].

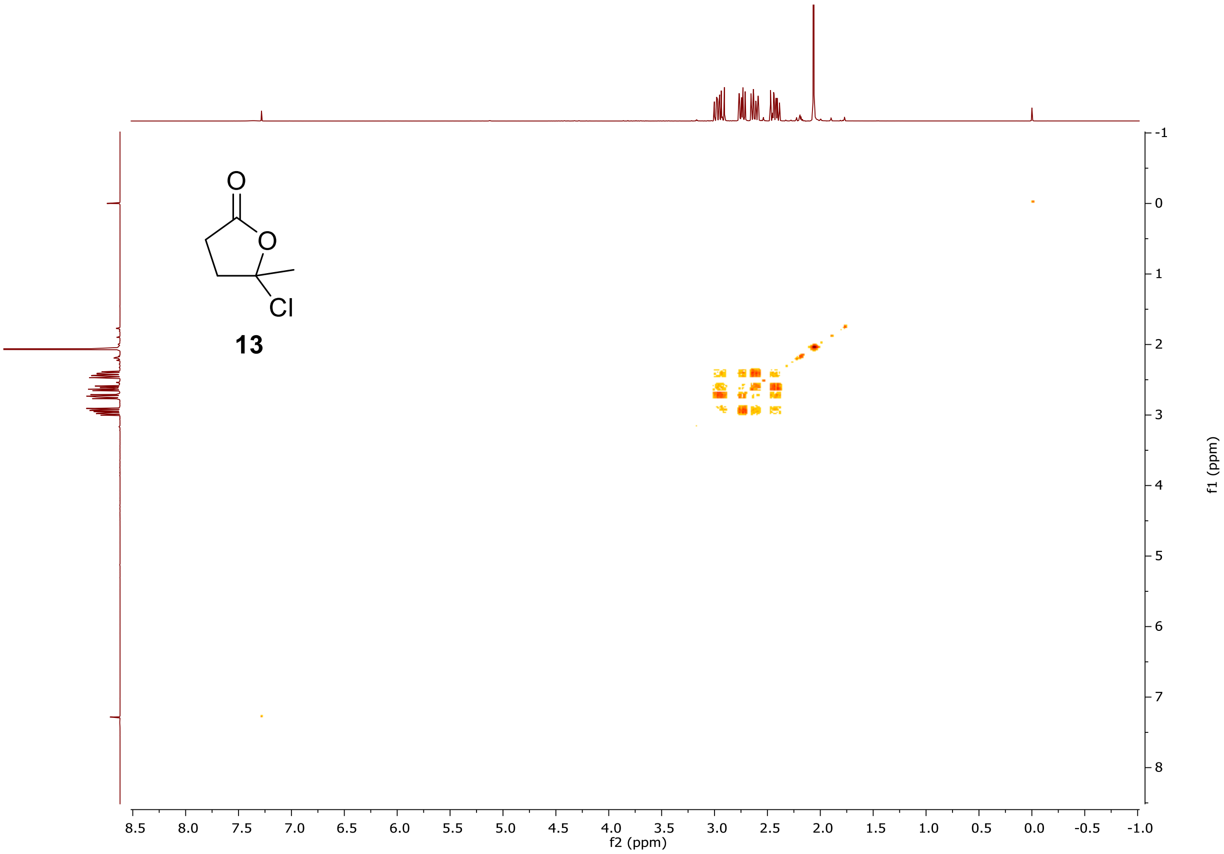

Figure S143: ^{1}H COSY NMR (400 MHz, $CDCl_3$) spectrum of 5-chloro-5-methyldihydrofuran-2(3H)-one (13) [AGO-2024-0004].

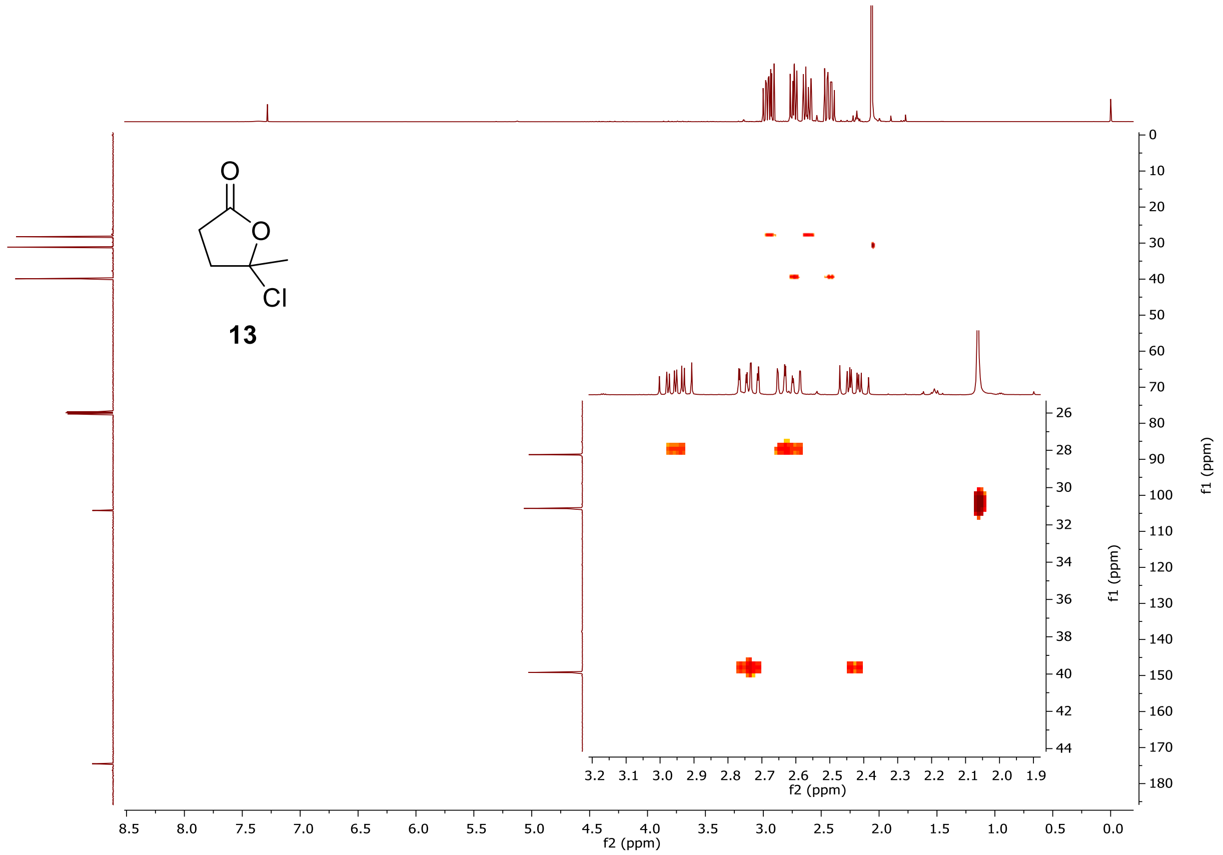

Figure S144: $^{1}H/^{13}C$ HSQC NMR ($CDCl_3$) spectrum of 5-chloro-5-methyldihydrofuran-2(3H)-one (13) [AGO-2024-0004].

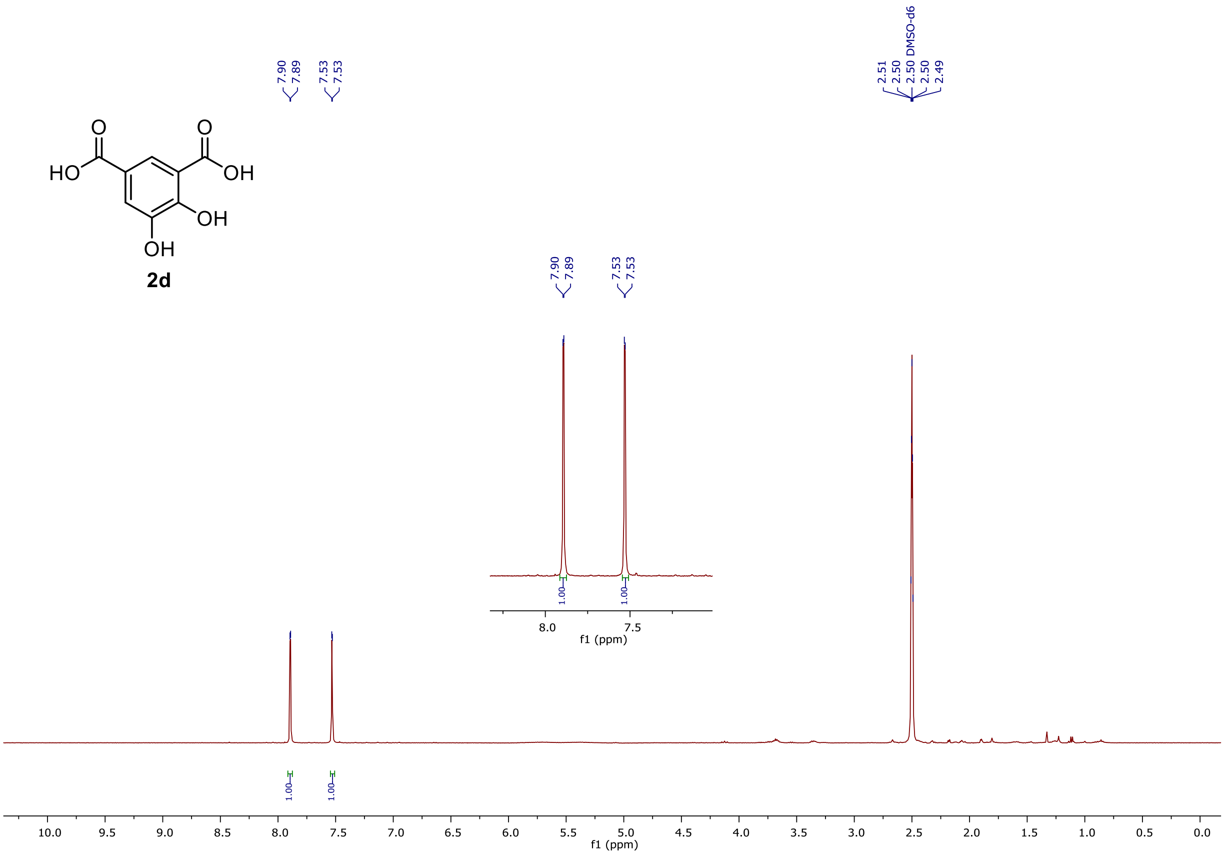

Figure S145: ^{1}H NMR (400 MHz, DMSO-*d*$_6$) spectrum of 4,5-dihydroxybenzene-1,3-dicarboxylic acid (2d) [AGO-125].

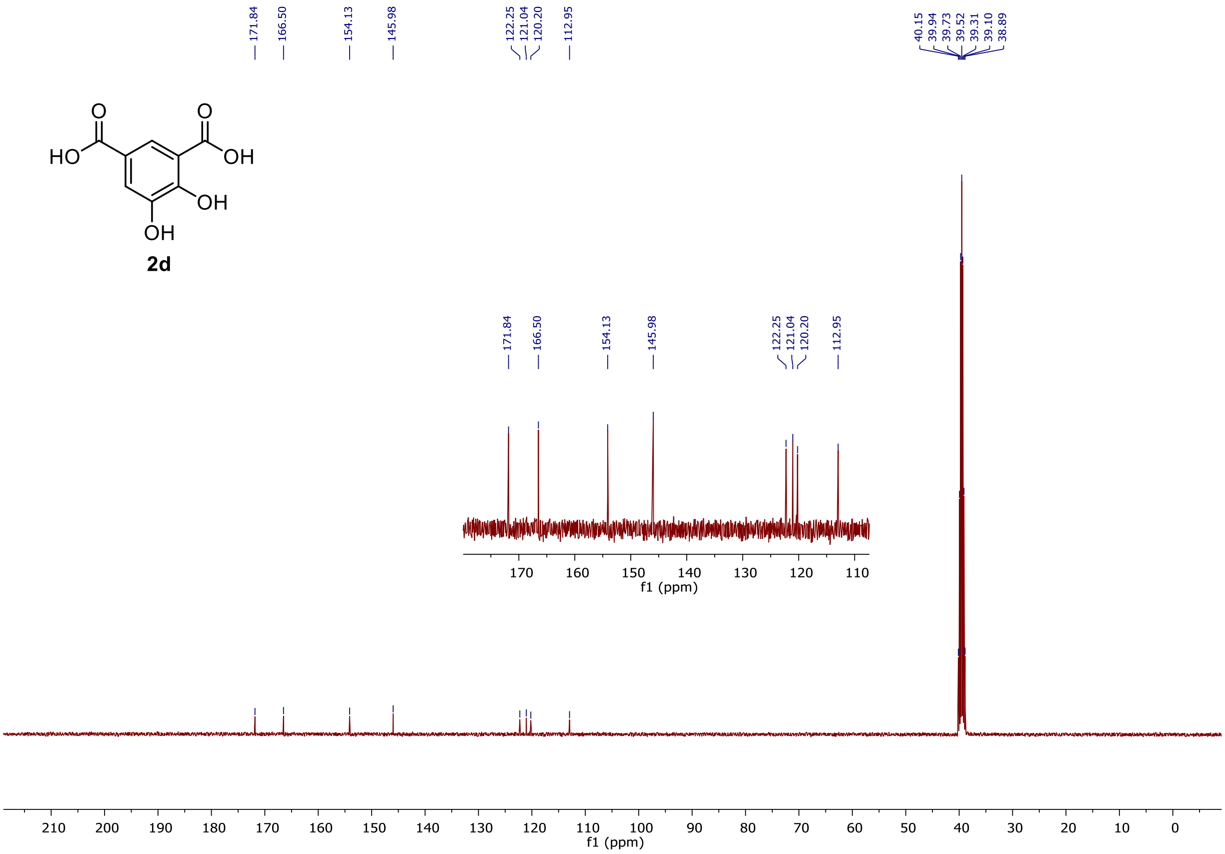

Figure S146: $^{13}C\{^1H\}$ NMR (101 MHz, DMSO-d_6) spectrum of 4,5-dihydroxybenzene-1,3-dicarboxylic acid (2d) [AGO-125].

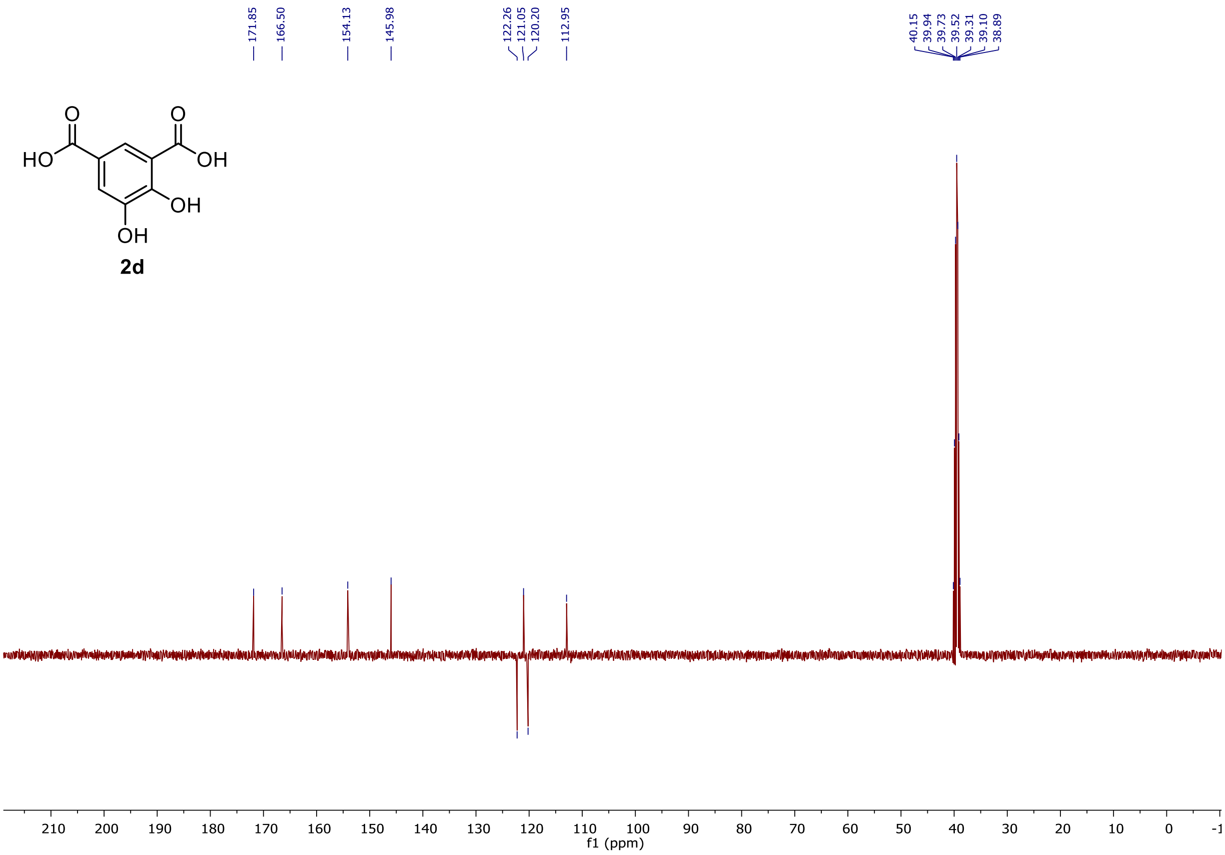

Figure S147: ^{13}C APT NMR (101 MHz, DMSO-d_6) spectrum of 4,5-dihydroxybenzene-1,3-dicarboxylic acid (2d) [AGO-125].

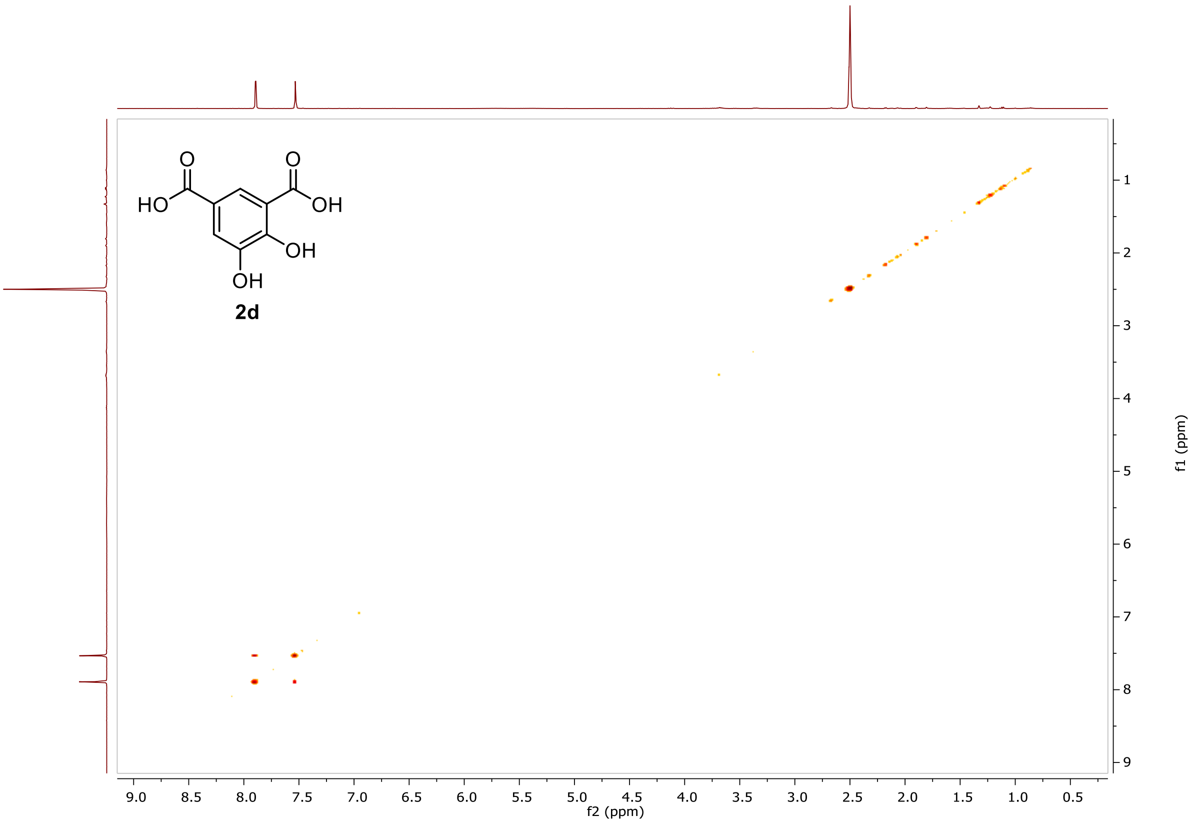

Figure S148: ^{1}H COSY NMR (400 MHz, DMSO-d_6) spectrum of 4,5-dihydroxybenzene-1,3-dicarboxylic acid (2d) [AGO-125].

14. DSC thermograms

14.1 DSC thermograms in PVC

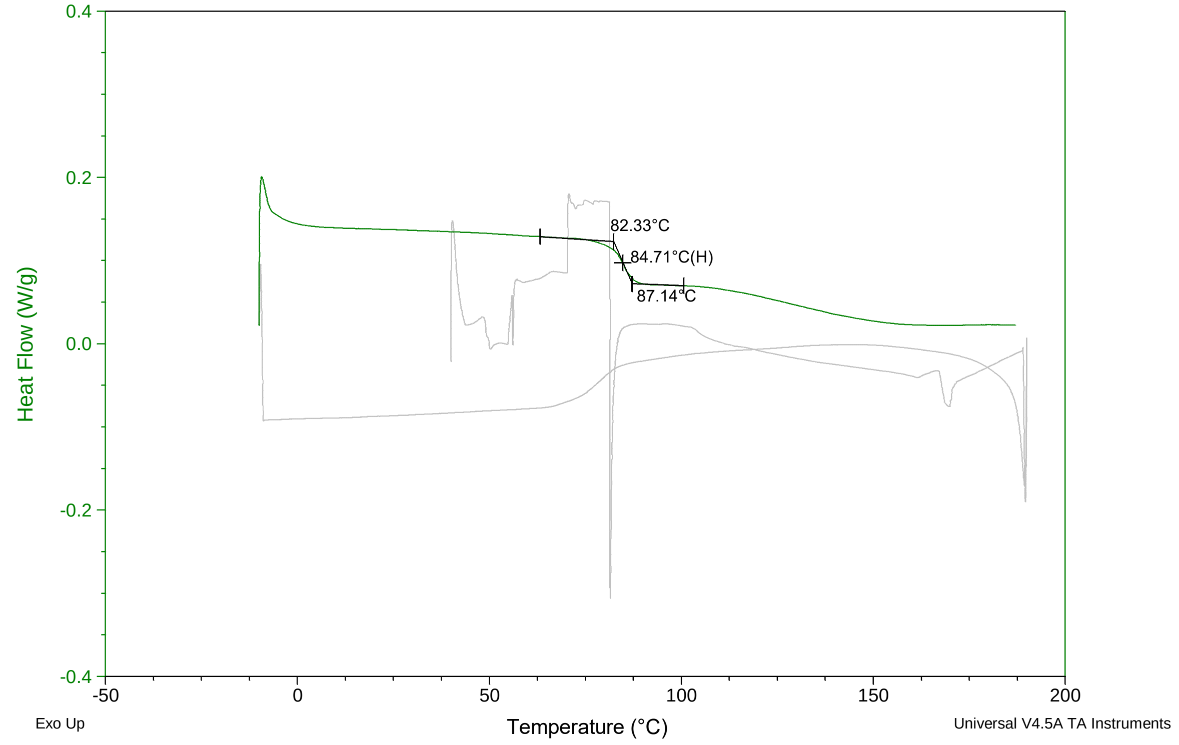

Figure S149: DSC thermogram of unplasticized PVC with 2nd heating cycle in green.

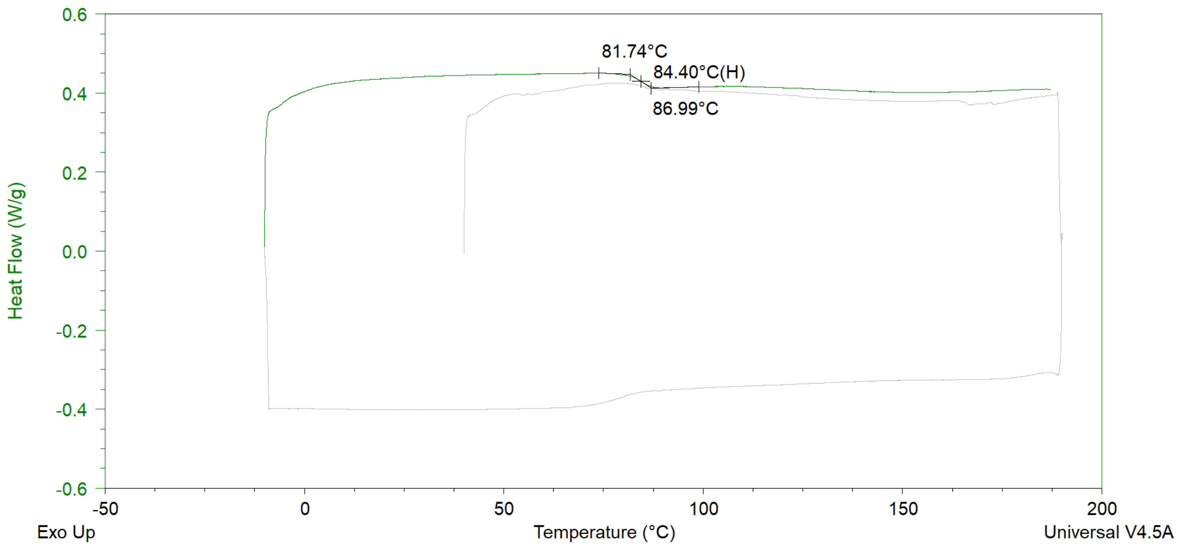

Figure S150: DSC thermogram of unplasticized PVC with 2nd heating cycle in green (2nd measurement).

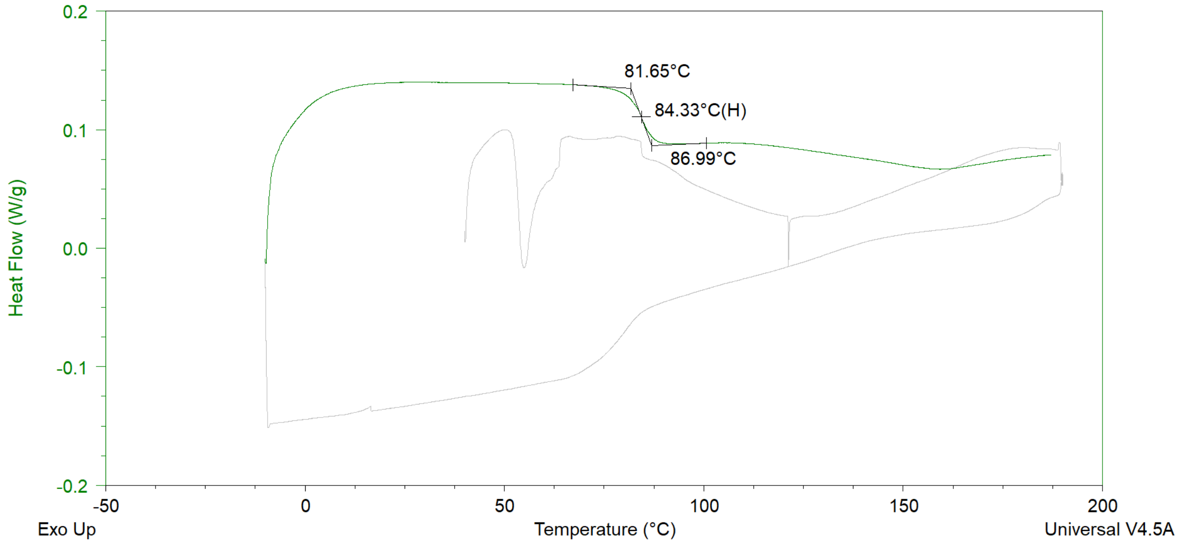

Figure S151: DSC thermogram of unplasticized PVC with 2nd heating cycle in green (3rd measurement).

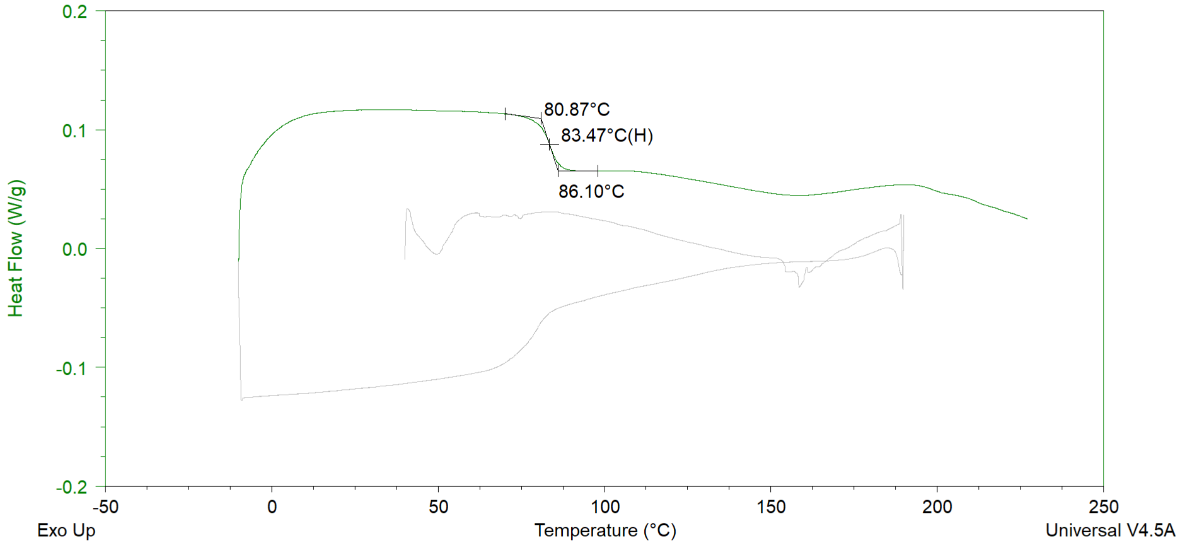

Figure S152: DSC thermogram of unplasticized PVC with 2nd heating cycle in green (4th measurement).

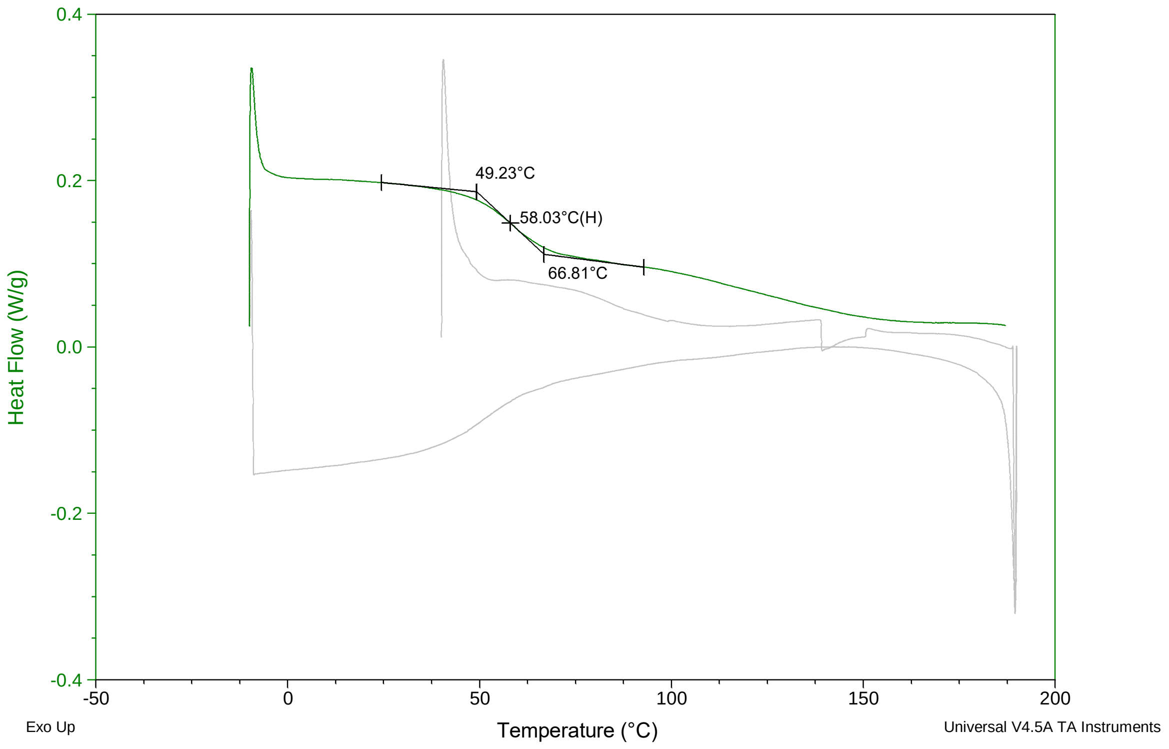

Figure S153: DSC thermograms of DEHP (10 wt%) in PVC with 2nd heating cycle in green.

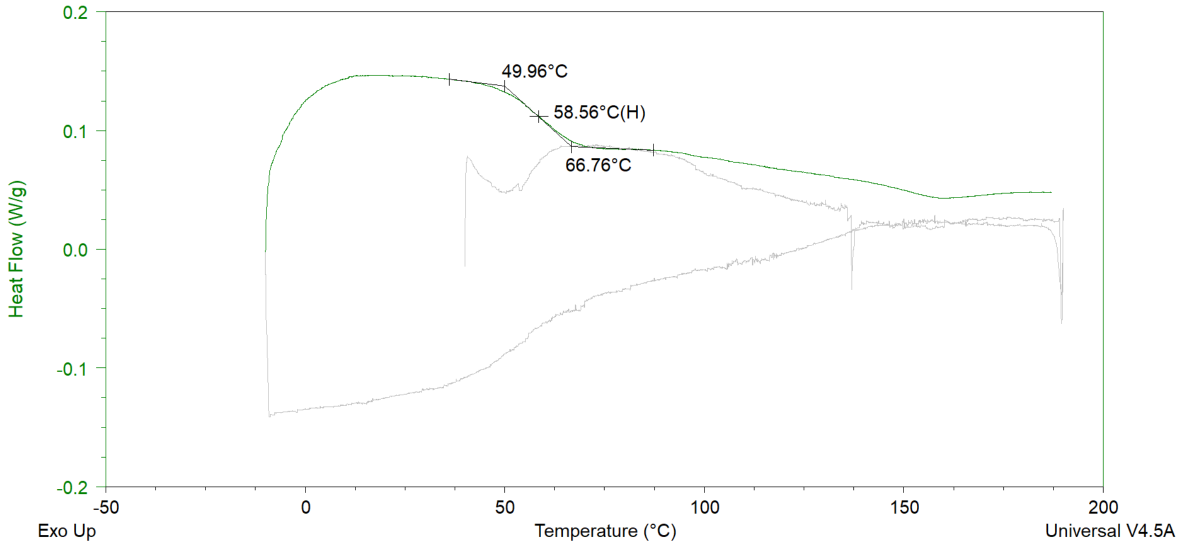

Figure S154: DSC thermograms of DEHP (10 wt%) in PVC with 2nd heating cycle in green (2nd measurement).

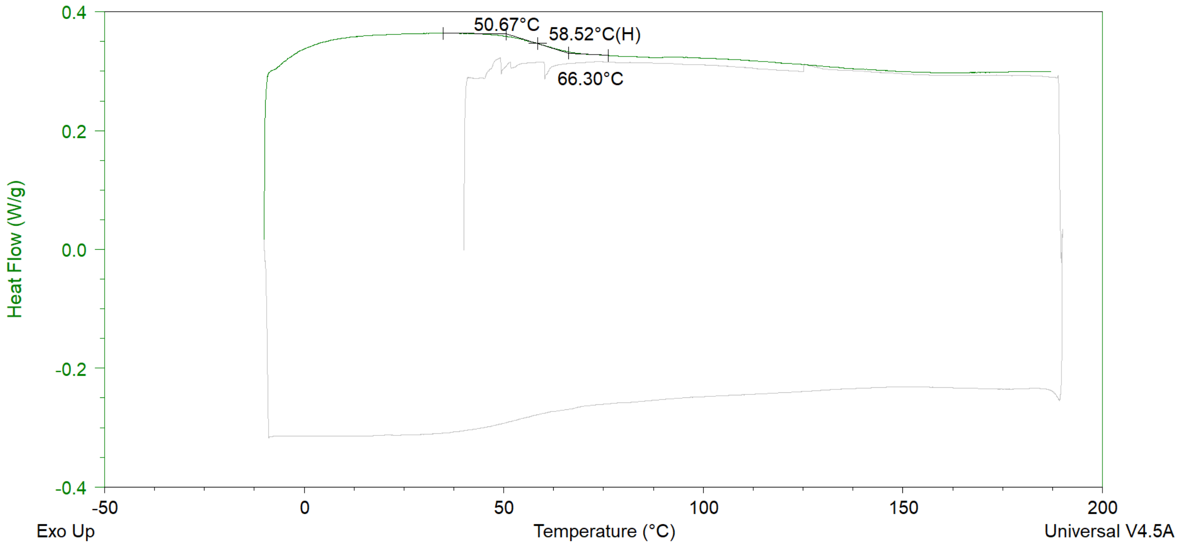

Figure S155: DSC thermograms of DEHP (10 wt%) in PVC with 2nd heating cycle in green (3rd measurement).

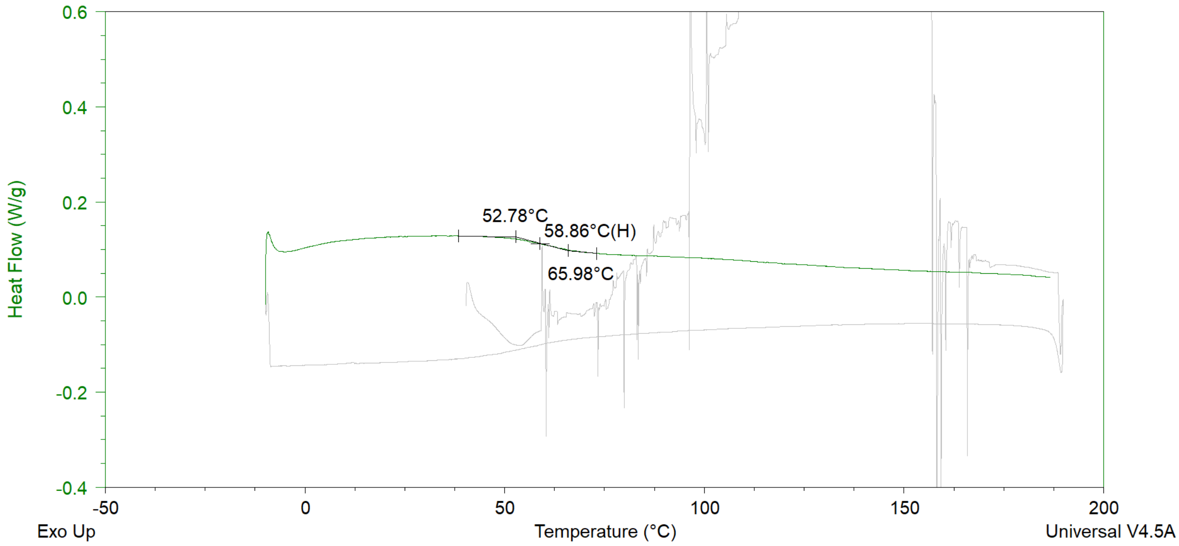

Figure S156: DSC thermograms of DEHP (10 wt%) in PVC with 2nd heating cycle in green (4th measurement).

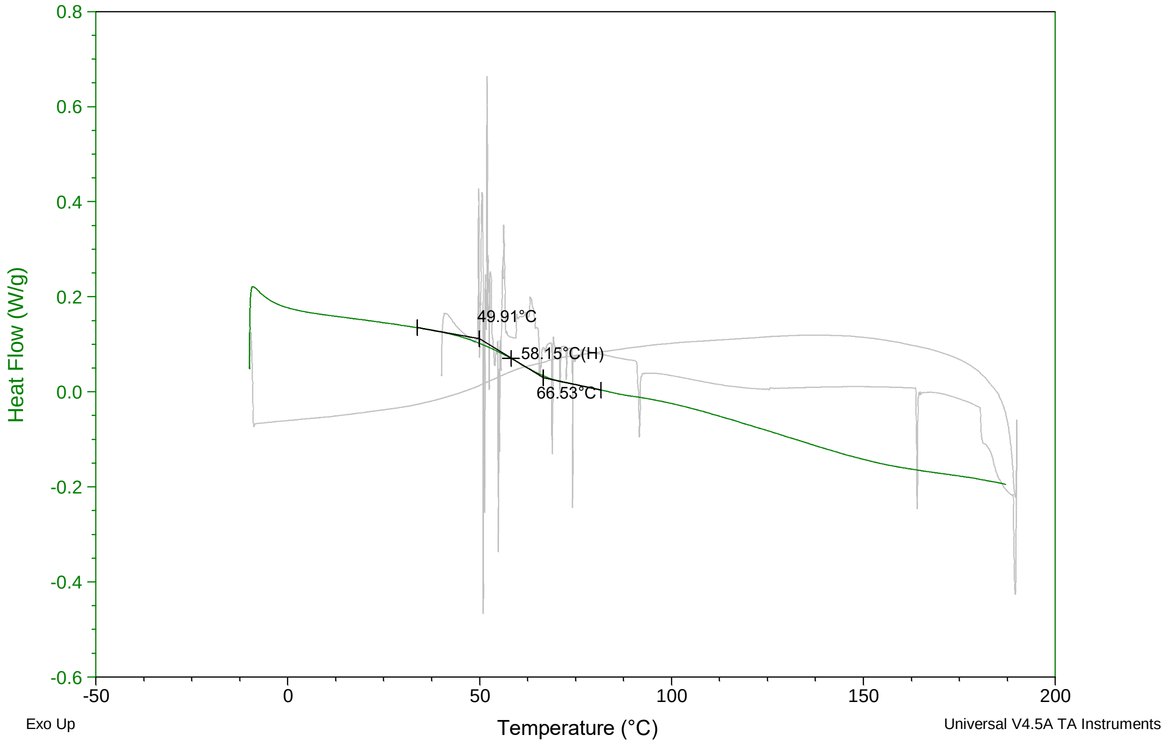

Figure S157: DSC thermograms of DINCH (10 wt%) in PVC with 2nd heating cycle in green.

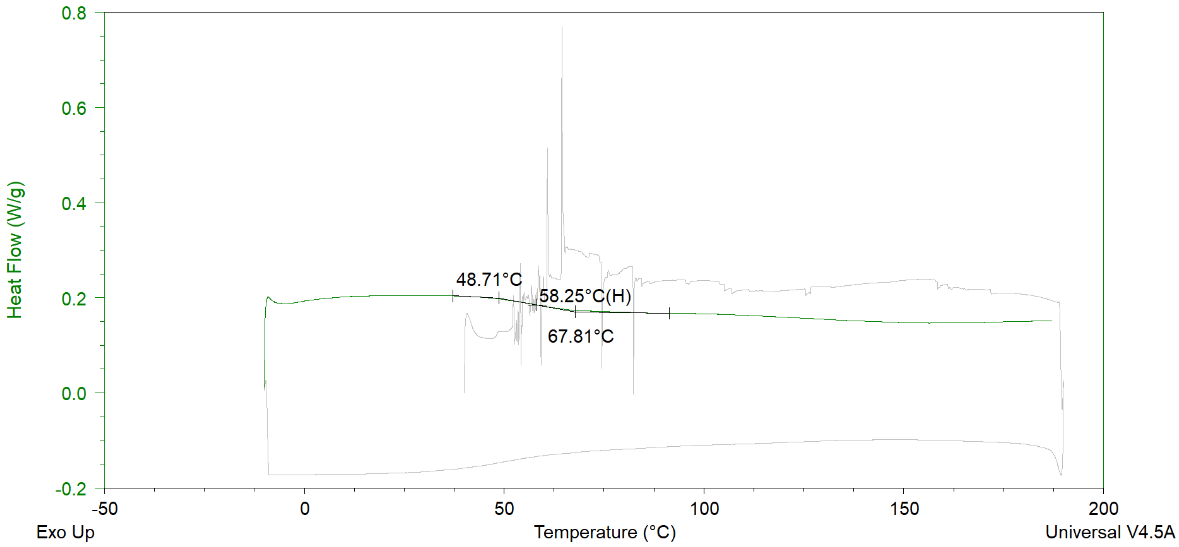

Figure S158: DSC thermograms of DINCH (10 wt%) in PVC with 2nd heating cycle in green (2nd measurement).

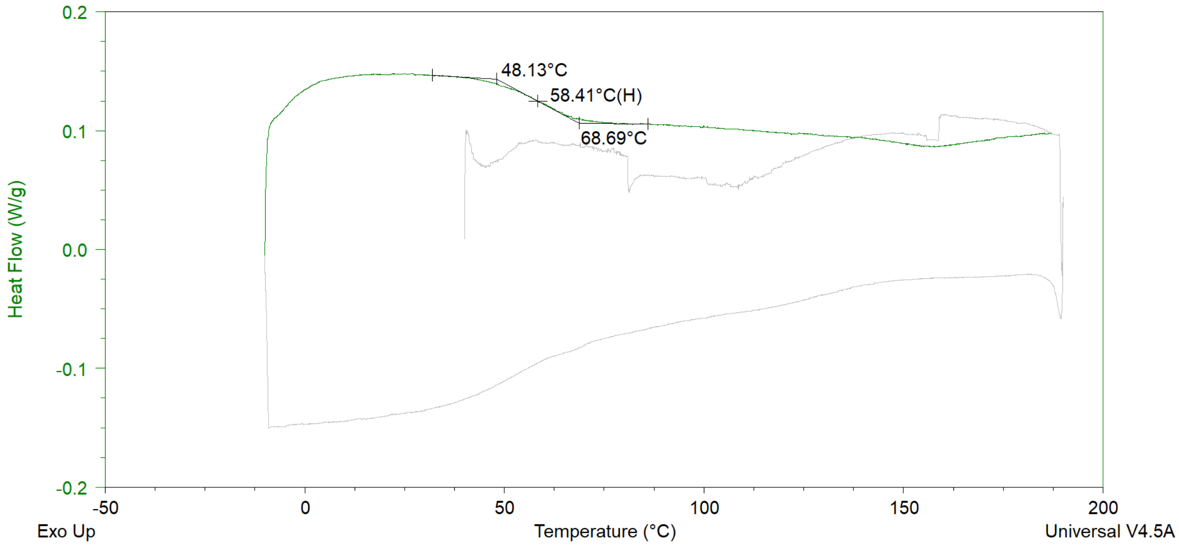

Figure S159: DSC thermograms of DINCH (10 wt%) in PVC with 2nd heating cycle in green (3rd measurement).

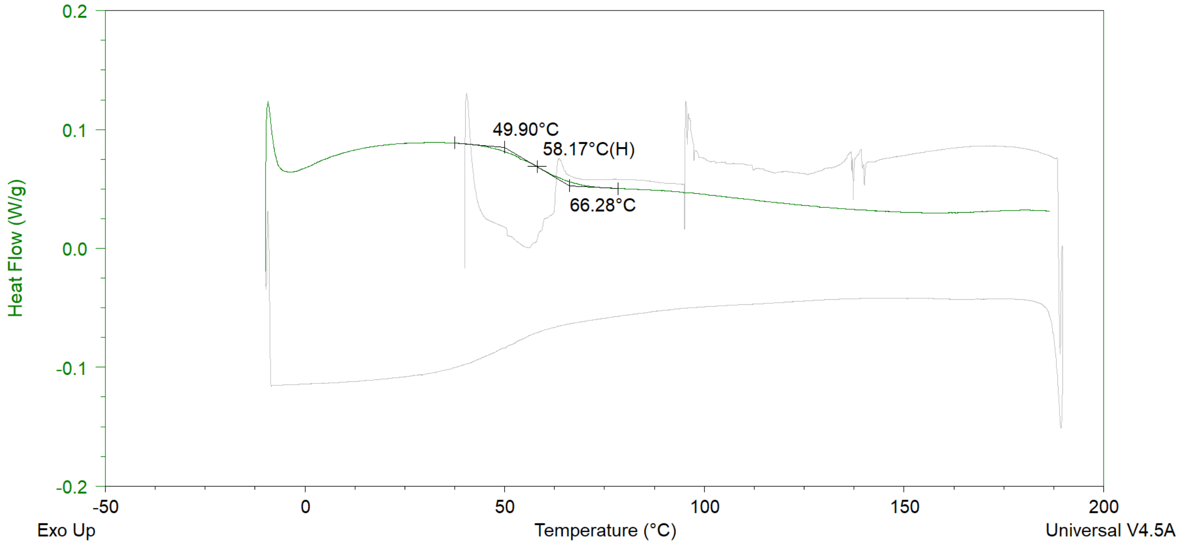

Figure S160: DSC thermograms of DINCH (10 wt%) in PVC with 2nd heating cycle in green (4th measurement).

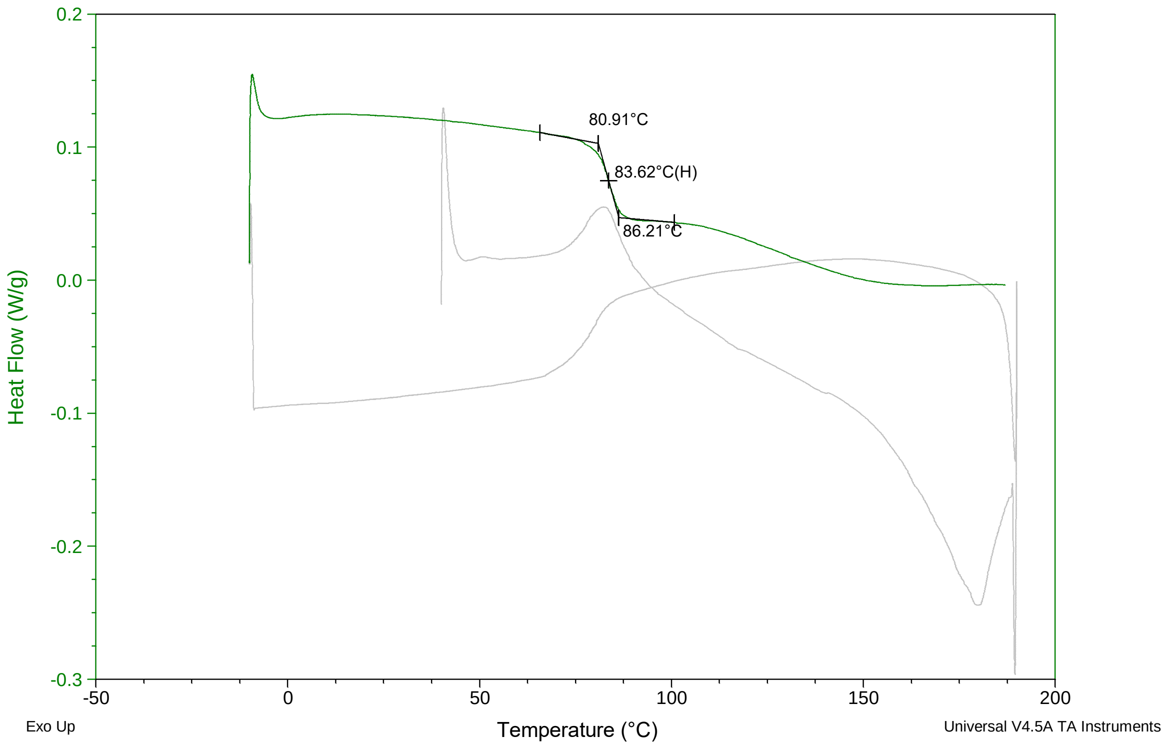

Figure S161: DSC thermograms of 2a (10 wt%) in PVC with 2nd heating cycle in green.

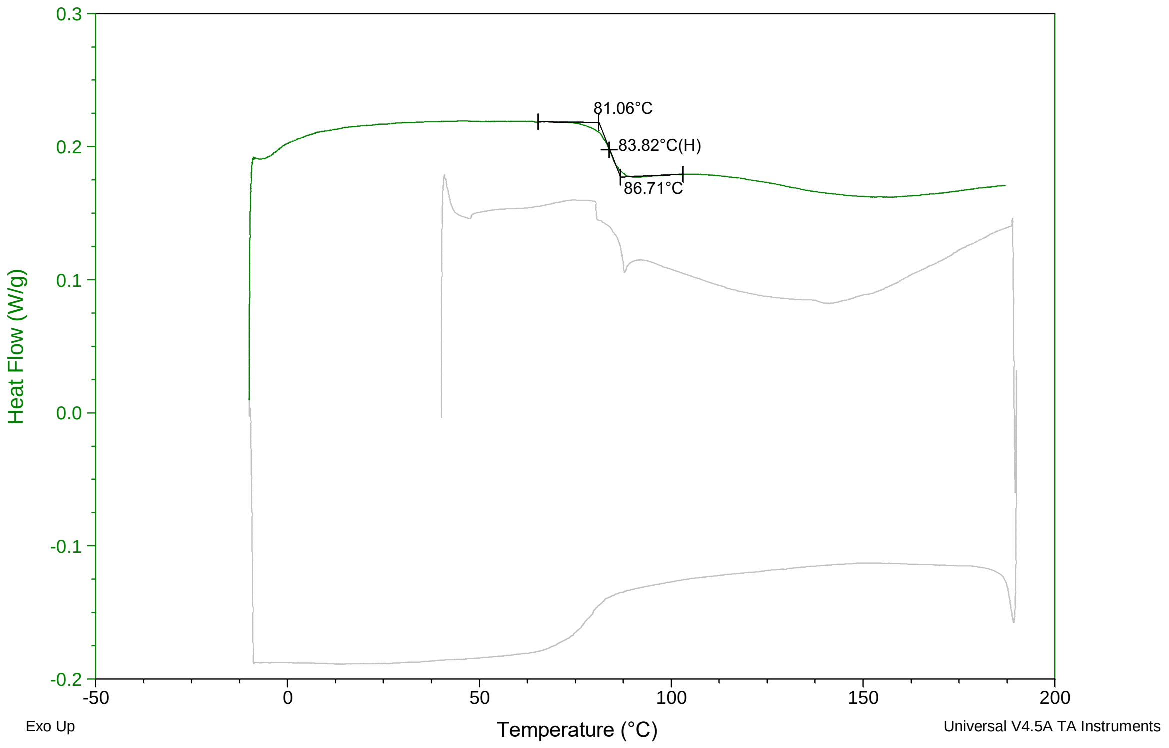

Figure S162: DSC thermograms of 3a (10 wt%) in PVC with 2nd heating cycle in green.

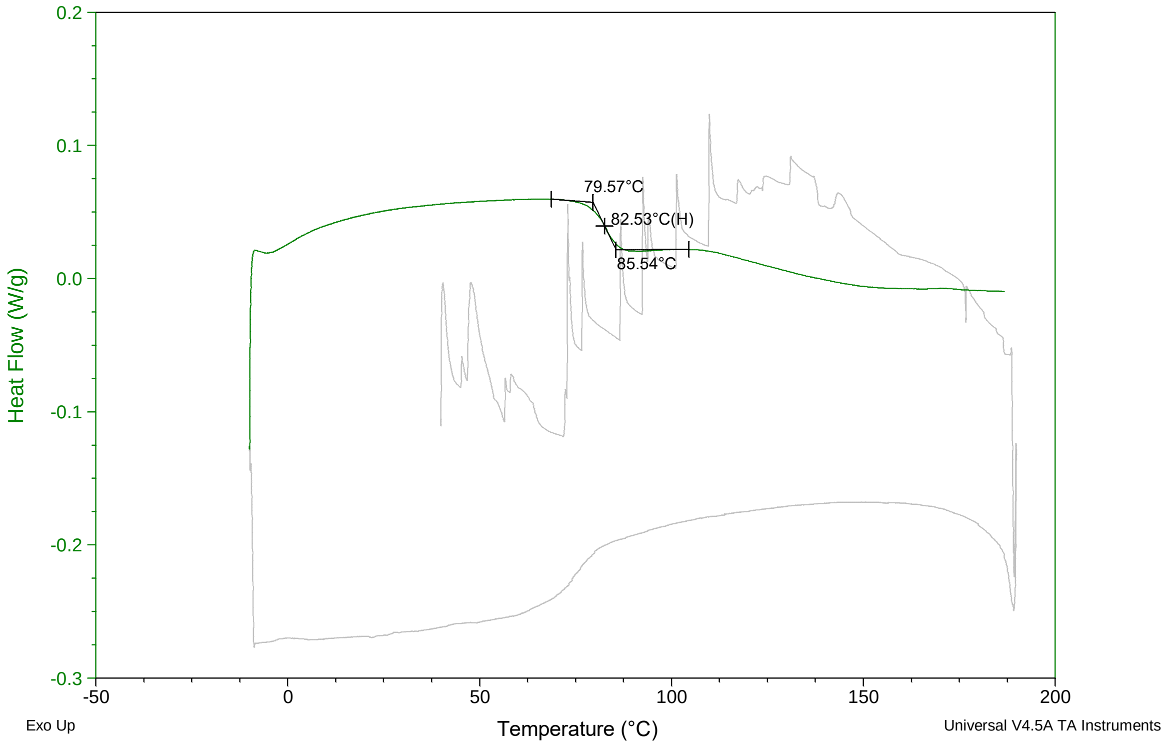

Figure S163: DSC thermograms of 4a (10 wt%) in PVC with 2nd heating cycle in green.

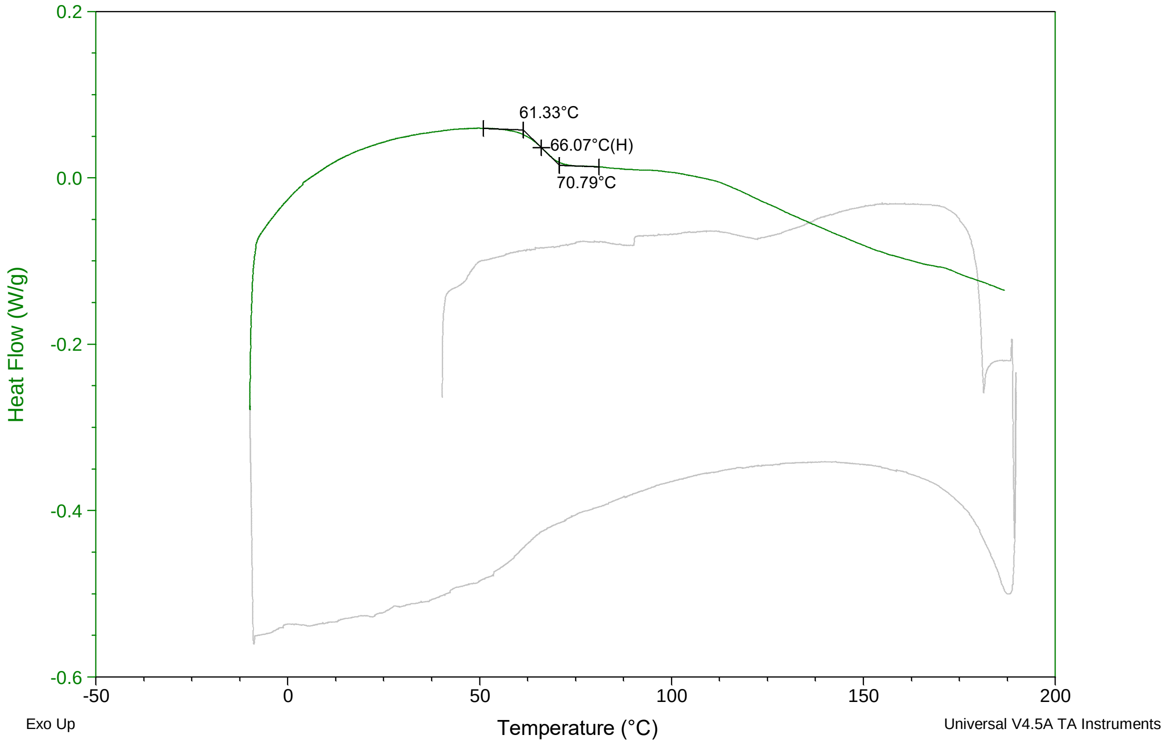

Figure S164: DSC thermograms of 5a (10 wt%) in PVC with 2nd heating cycle in green.

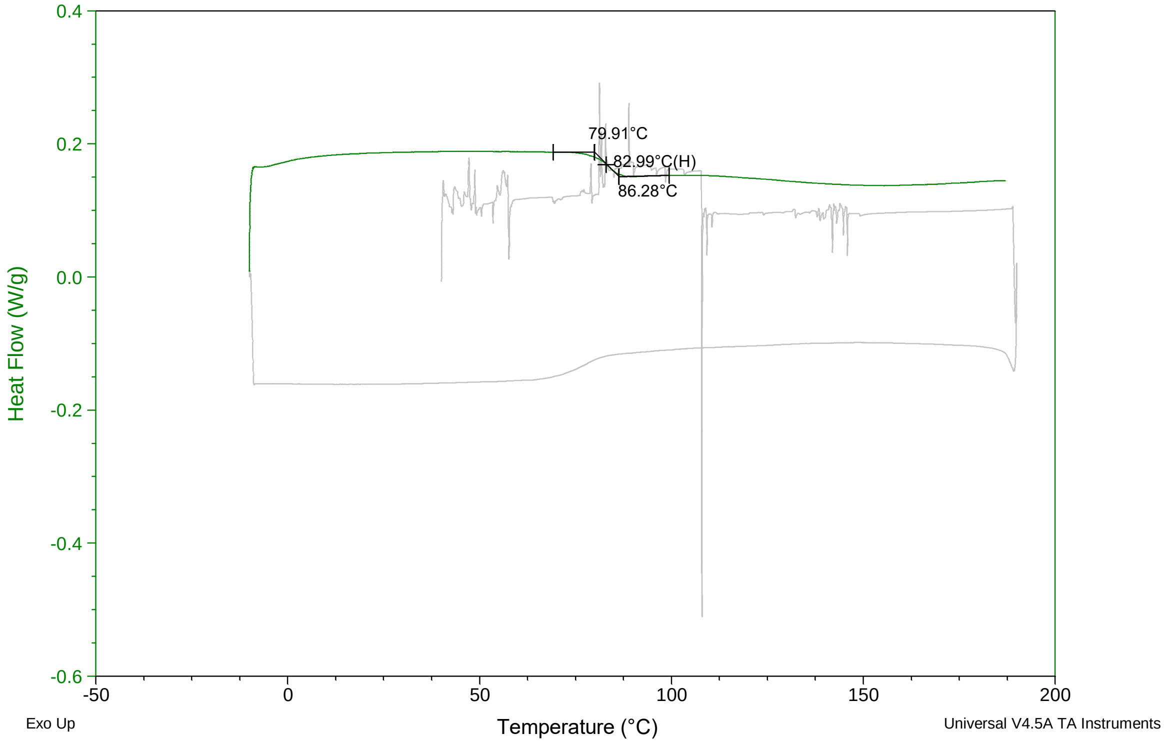

Figure S165: DSC thermograms of 6a (10 wt%) in PVC with 2nd heating cycle in green.

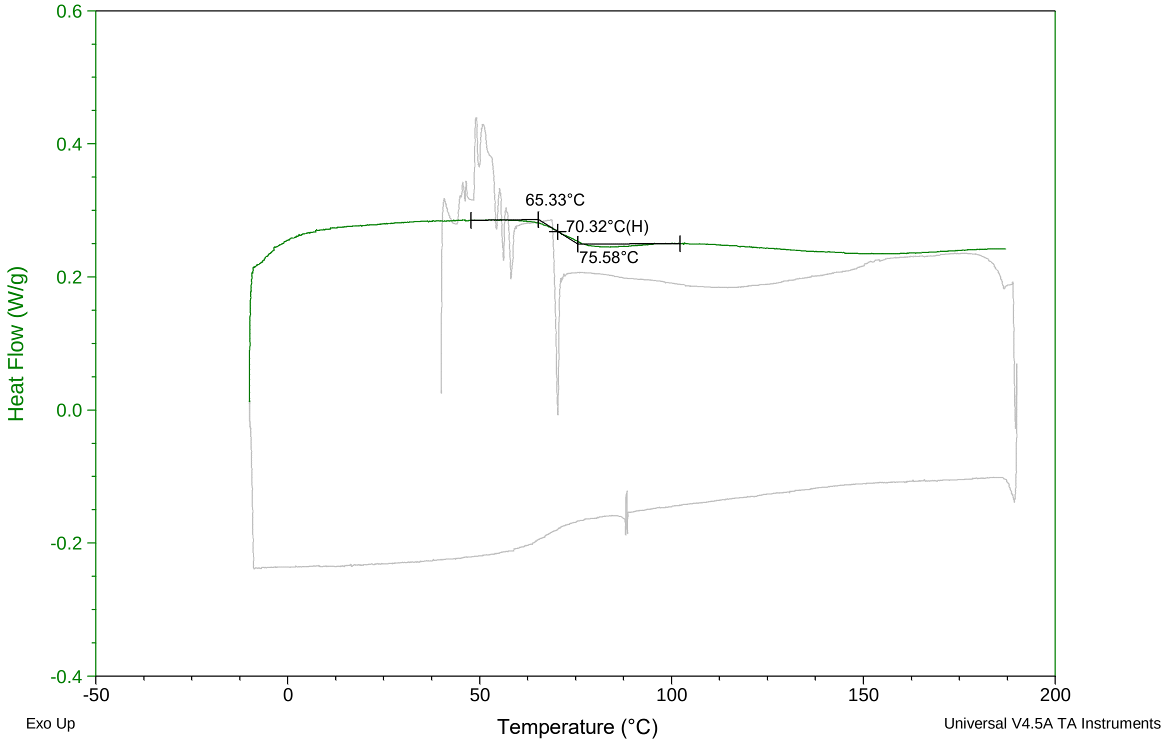

Figure S166: DSC thermograms of 7a (10 wt%) in PVC with 2nd heating cycle in green.

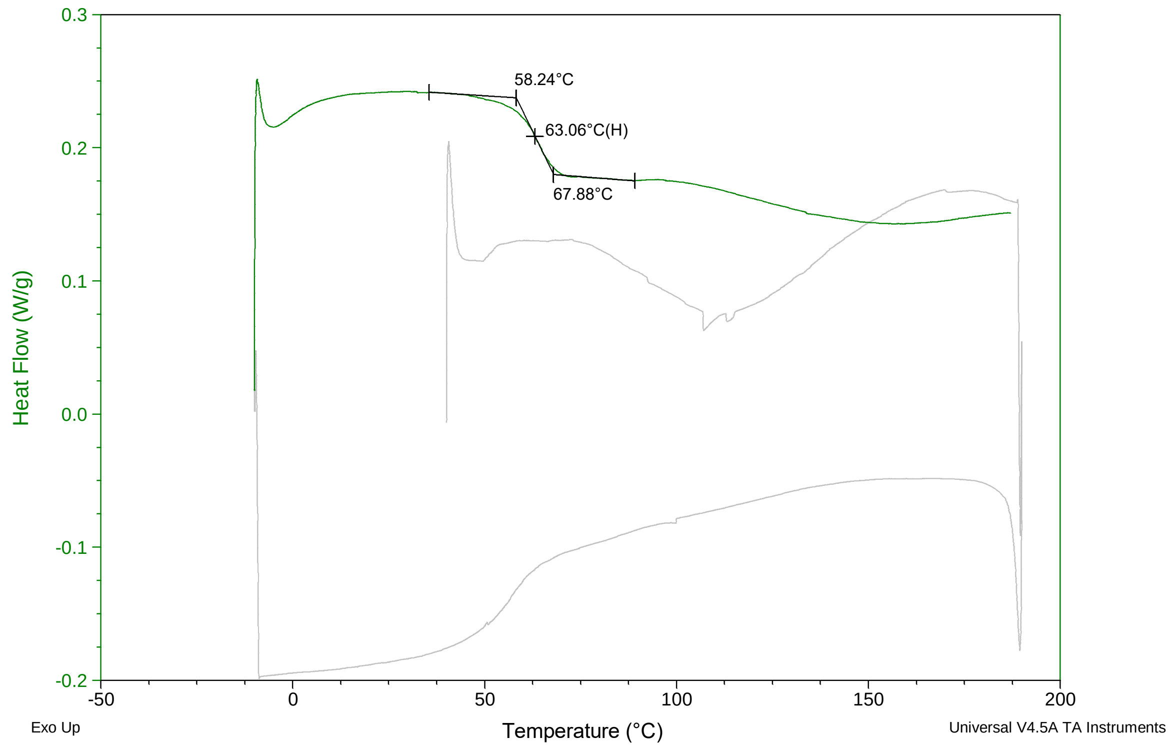

Figure S167: DSC thermograms of 8a (10 wt%) in PVC with 2nd heating cycle in green.

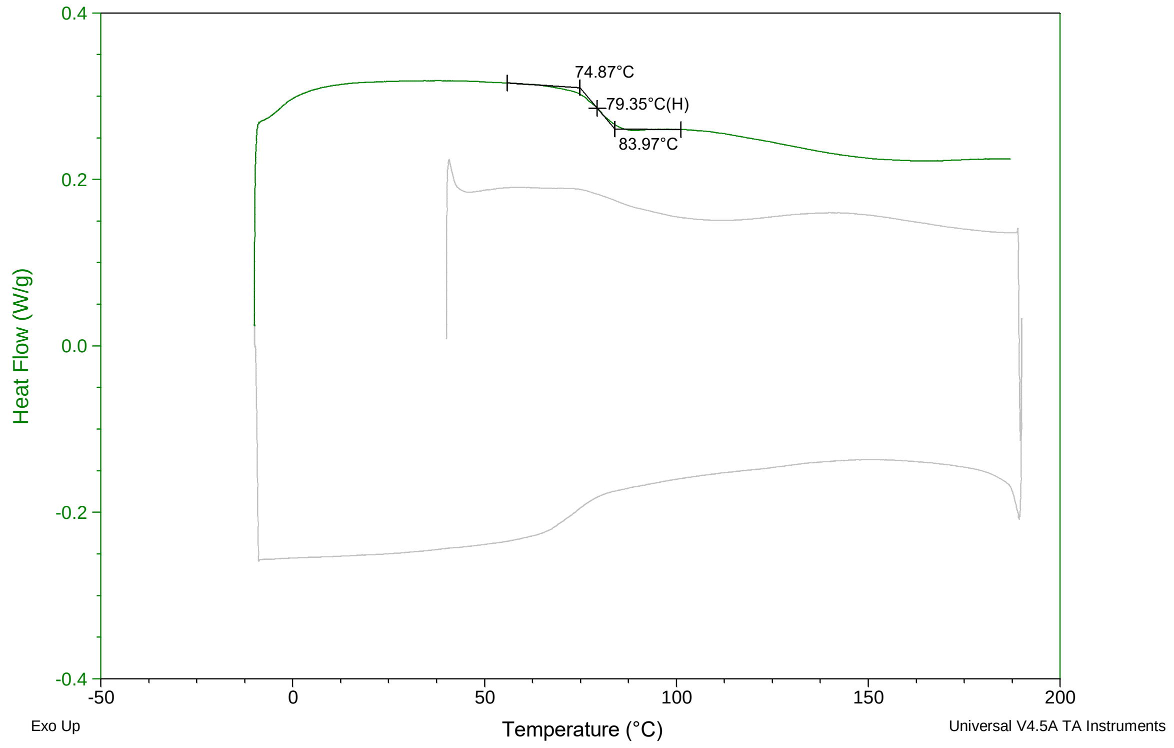

Figure S168: DSC thermogram of 9a (10 wt%) in PVC with 2nd heating cycle in green.

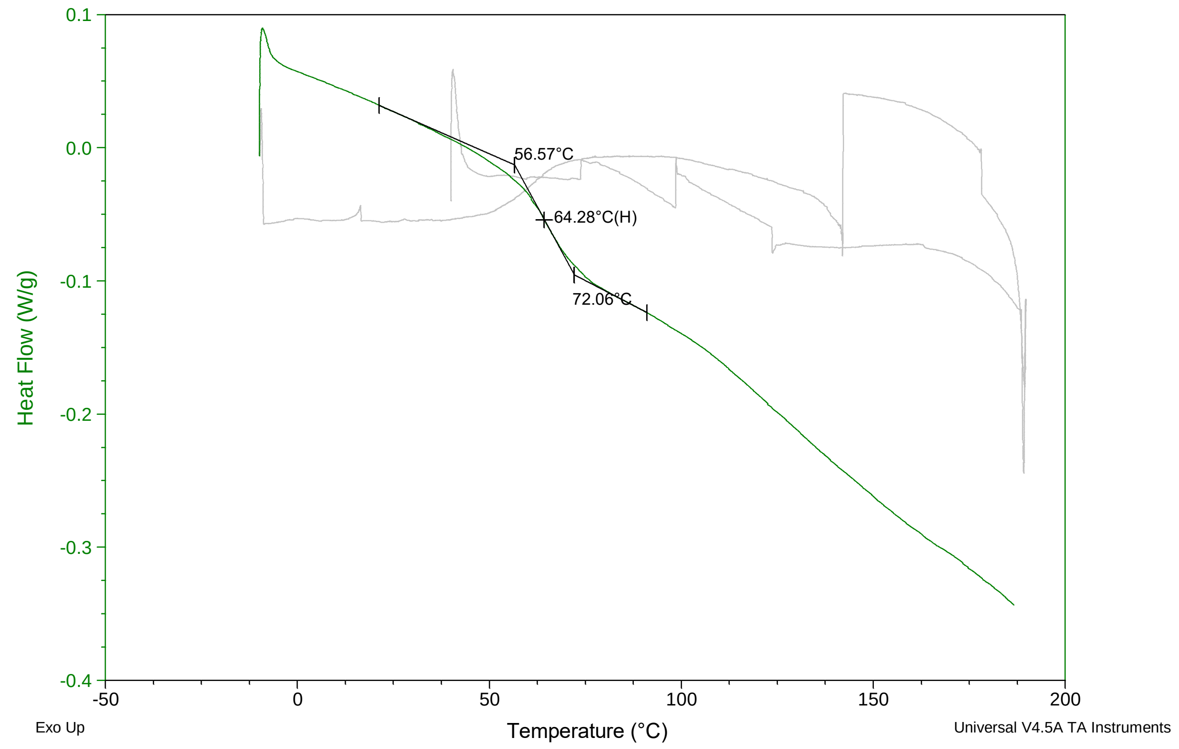

Figure S169: DSC thermogram of 10a (10 wt%) in PVC with 2nd heating cycle in green.

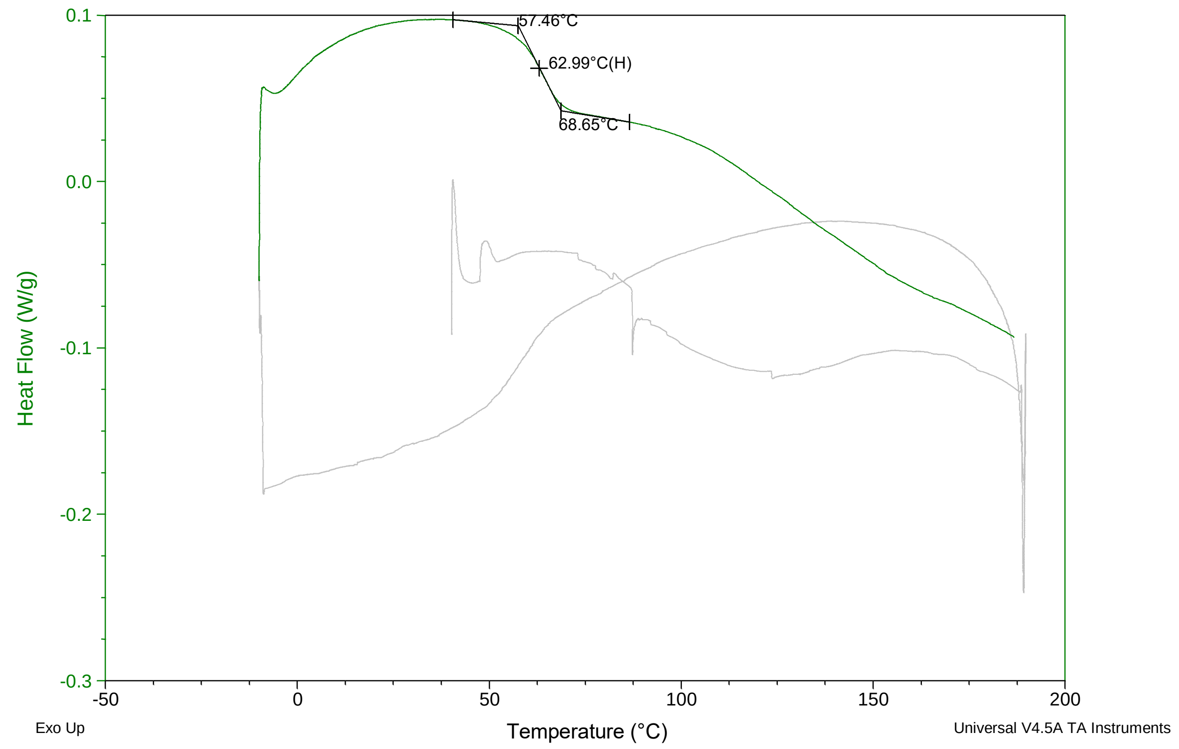

Figure S170: DSC thermogram of 11a (10 wt%) in PVC with 2nd heating cycle in green.

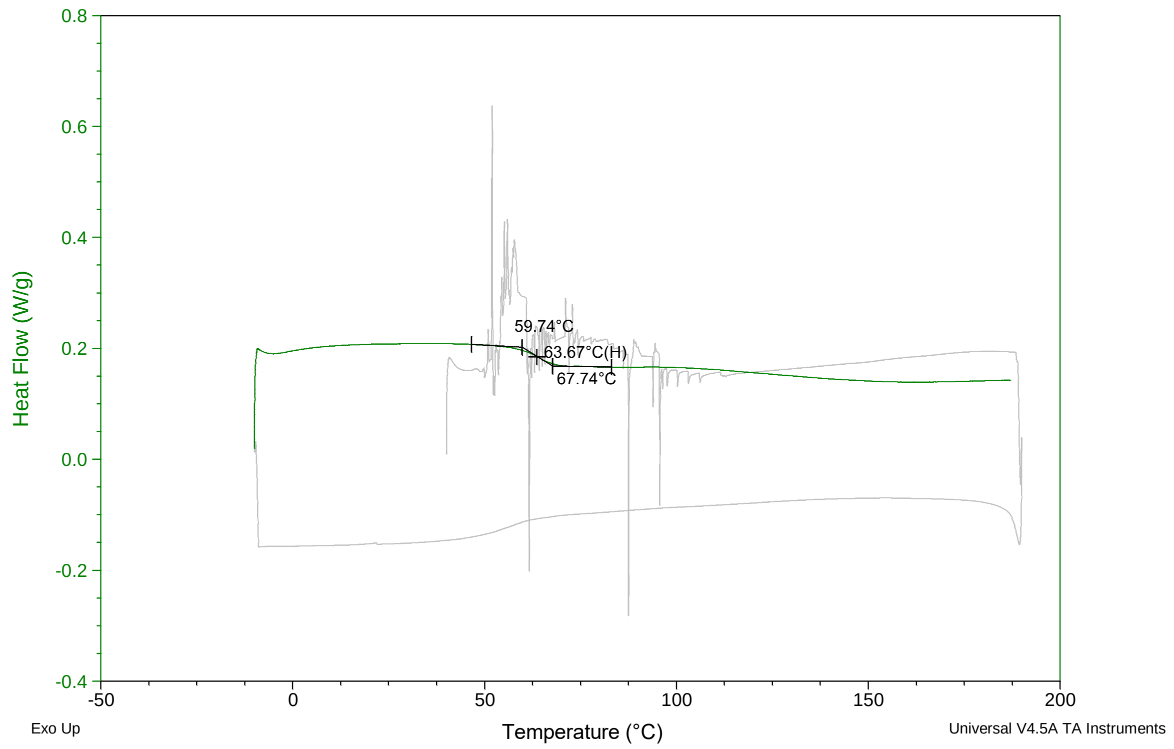

Figure S171: DSC thermogram of 8b (10 wt%) in PVC with 2nd heating cycle in green.

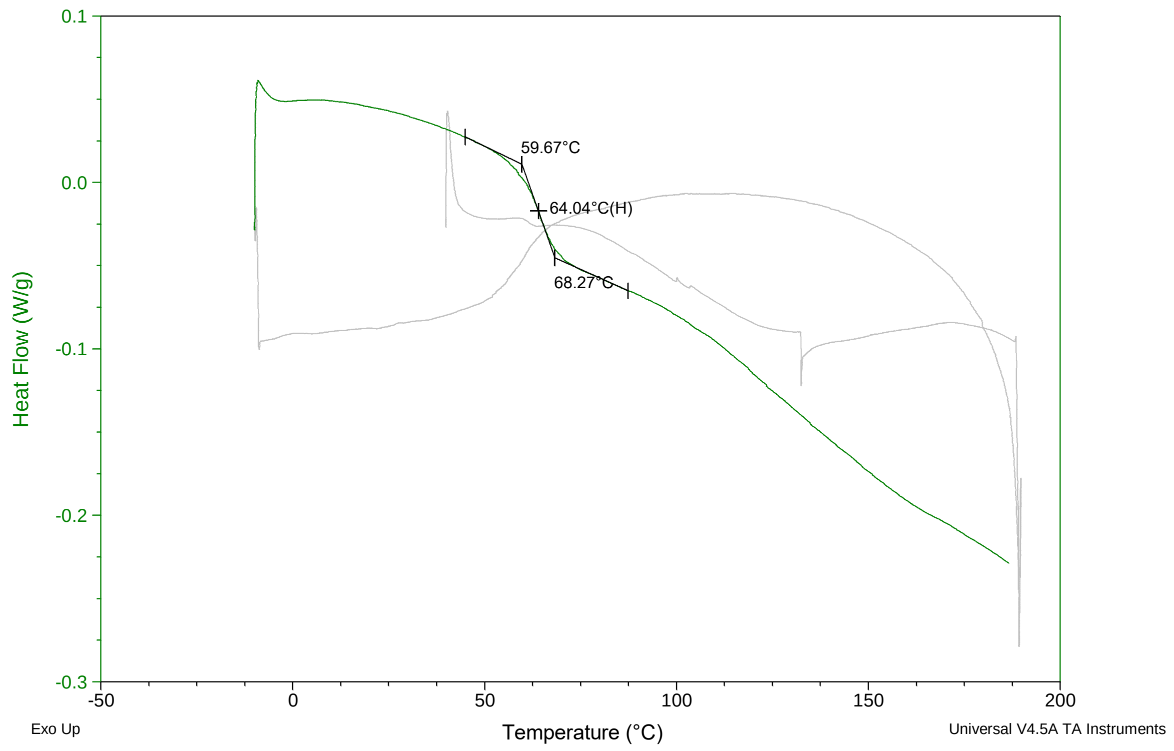

Figure S172: DSC thermogram of 8c (10 wt%) in PVC with 2nd heating cycle in green.

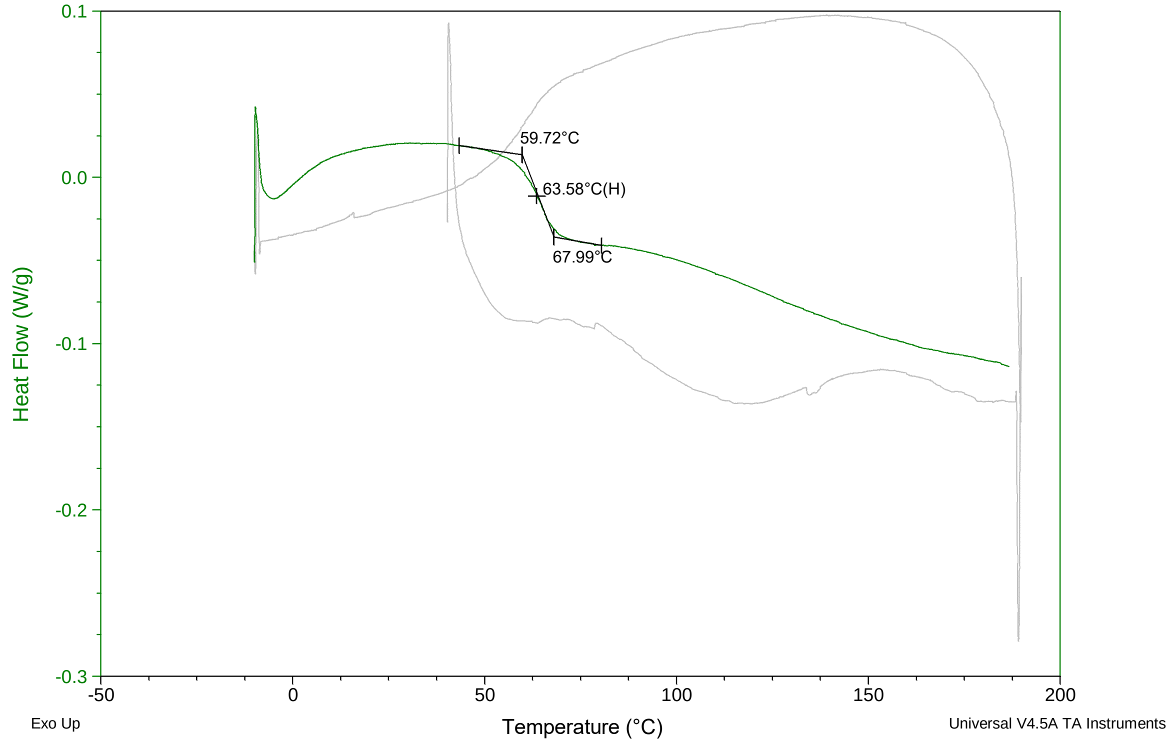

Figure S173: DSC thermogram of 8d (10 wt%) in PVC with 2nd heating cycle in green.

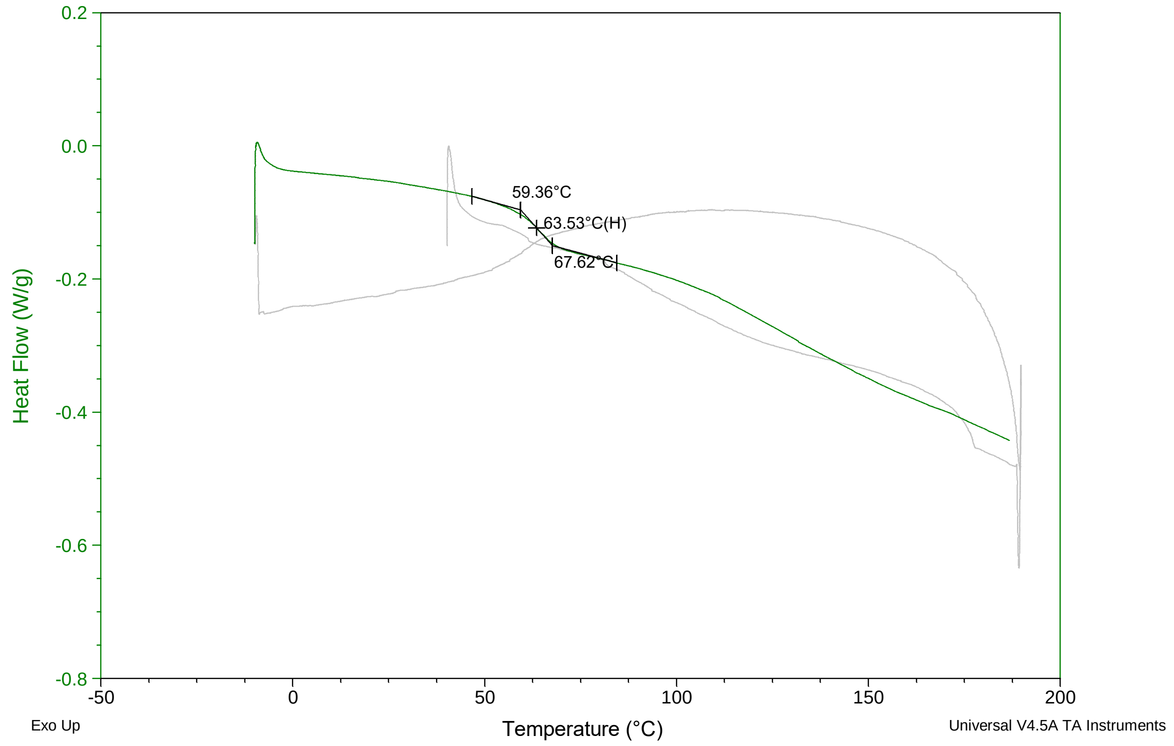

Figure S174: DSC thermogram of 11b (10 wt%) in PVC with 2nd heating cycle in green.

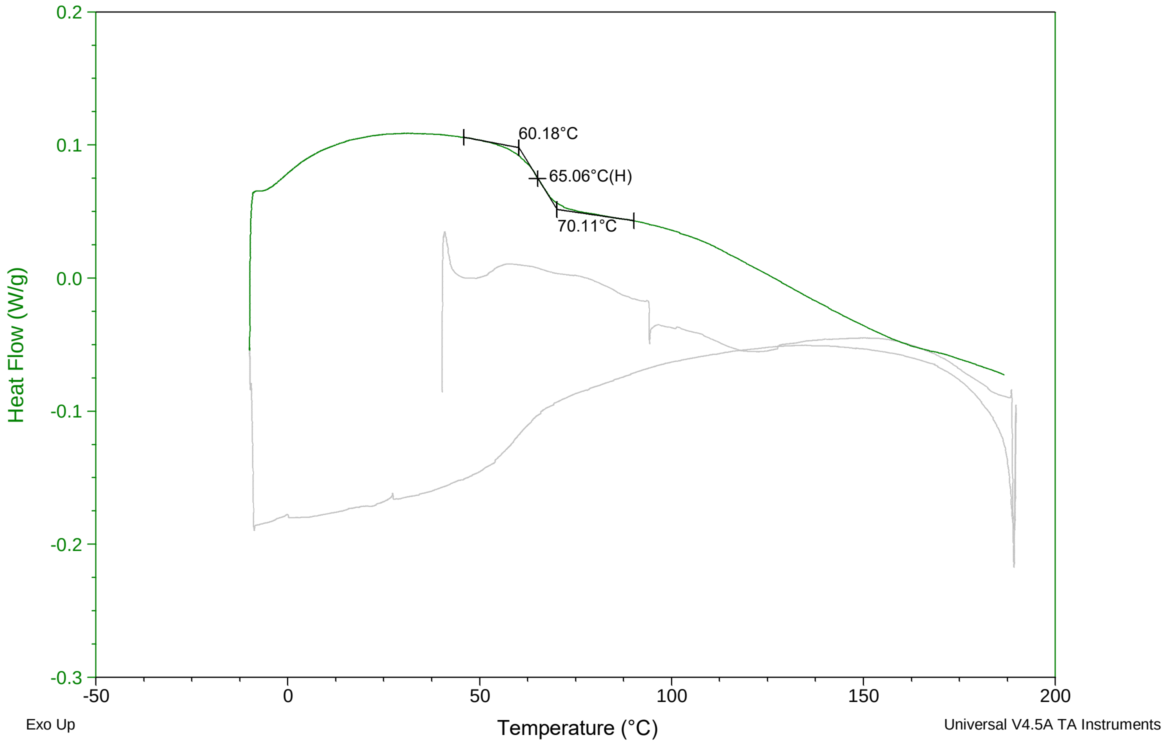

Figure S175: DSC thermogram of 11c (10 wt%) in PVC with 2nd heating cycle in green.

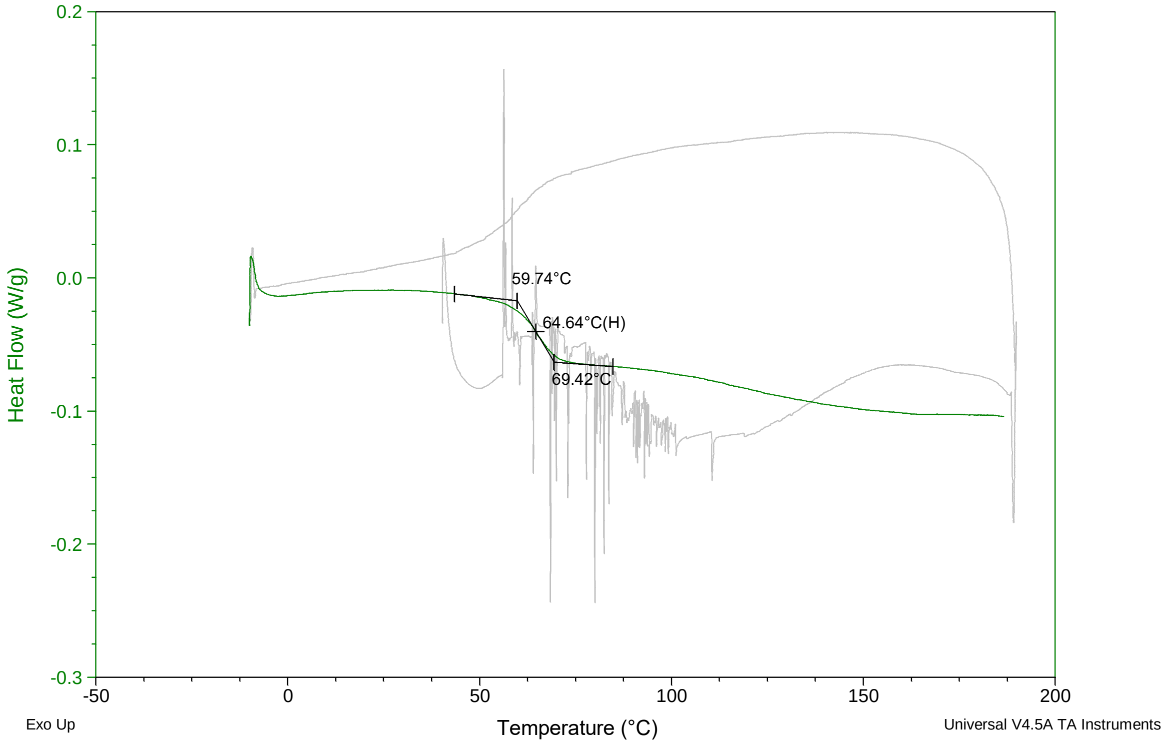

Figure S176: DSC thermogram of 11d (10 wt%) in PVC with 2nd heating cycle in green.

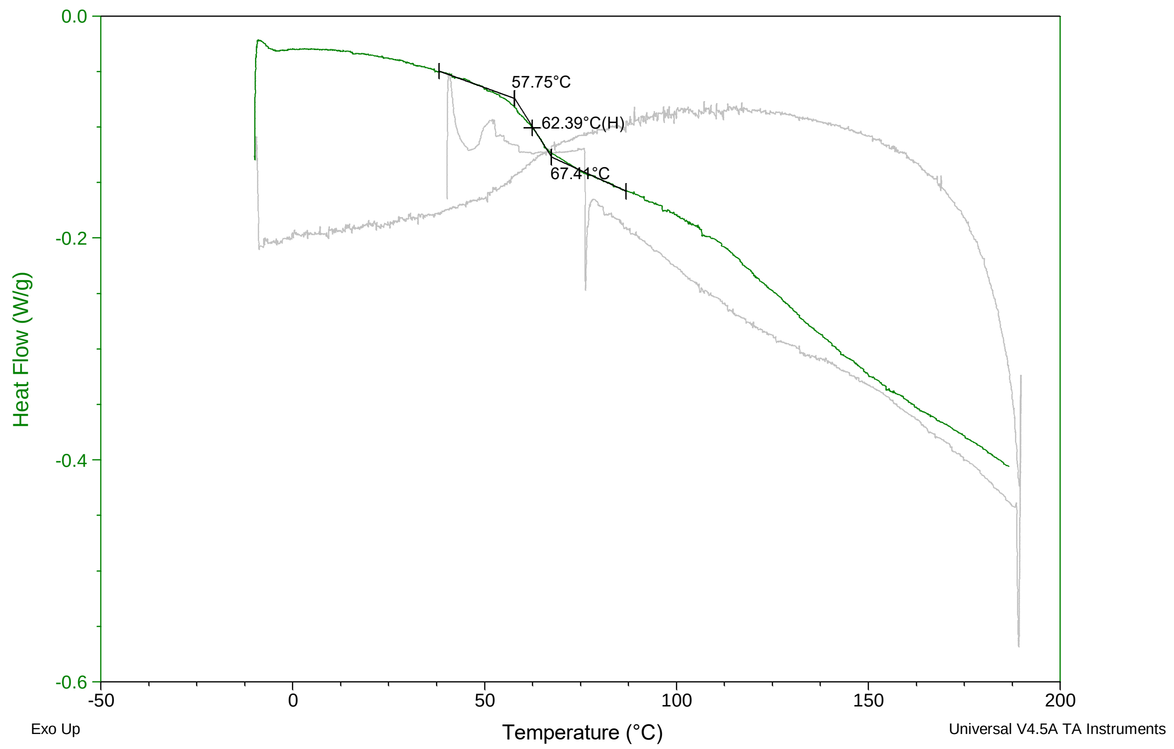

Figure S177: DSC thermogram of 12a (10 wt%) in PVC with 2nd heating cycle in green.

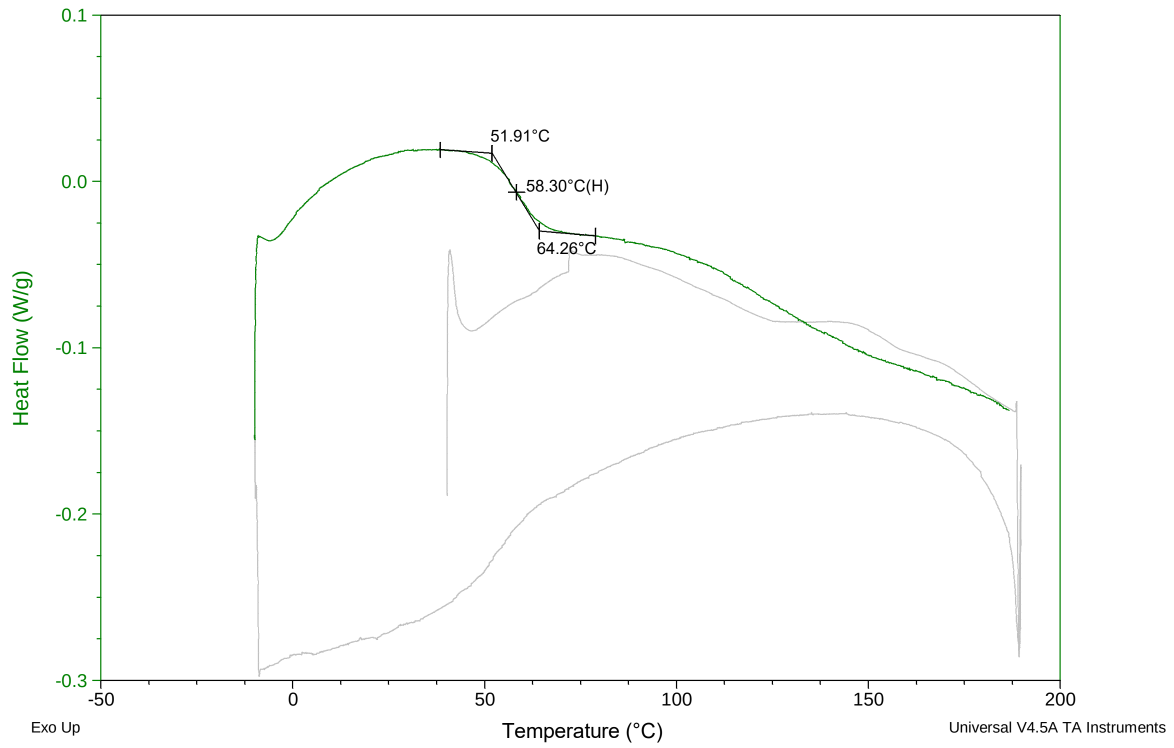

Figure S178: DSC thermogram of 12b (10 wt%) in PVC with 2nd heating cycle in green.

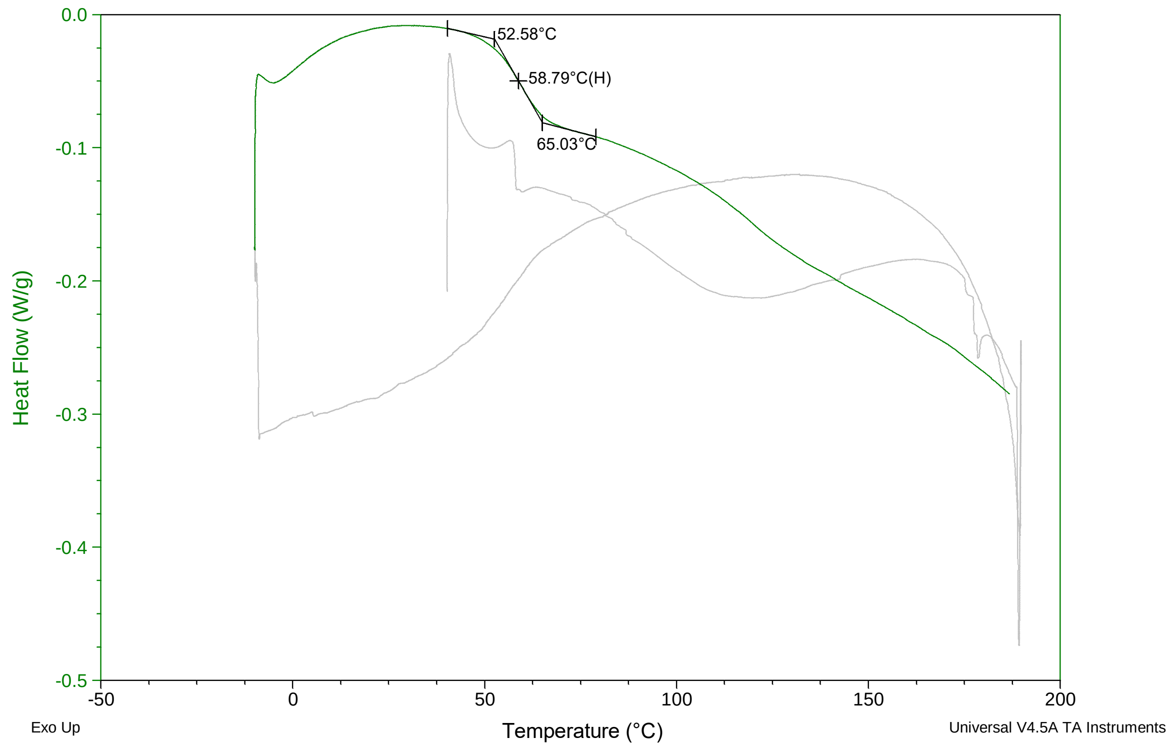

Figure S179: DSC thermogram of 12b (10 wt%) in PVC with 2nd heating cycle in green (2nd measurement).

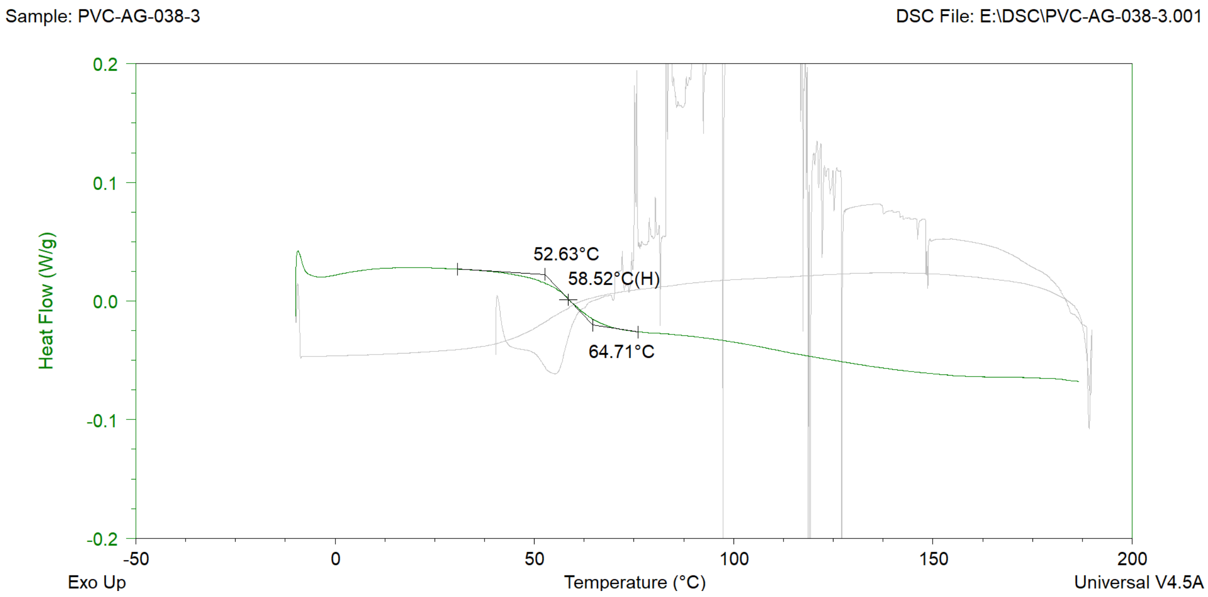

Figure S180: DSC thermogram of 12b (10 wt%) in PVC with 2nd heating cycle in green (3rd measurement).

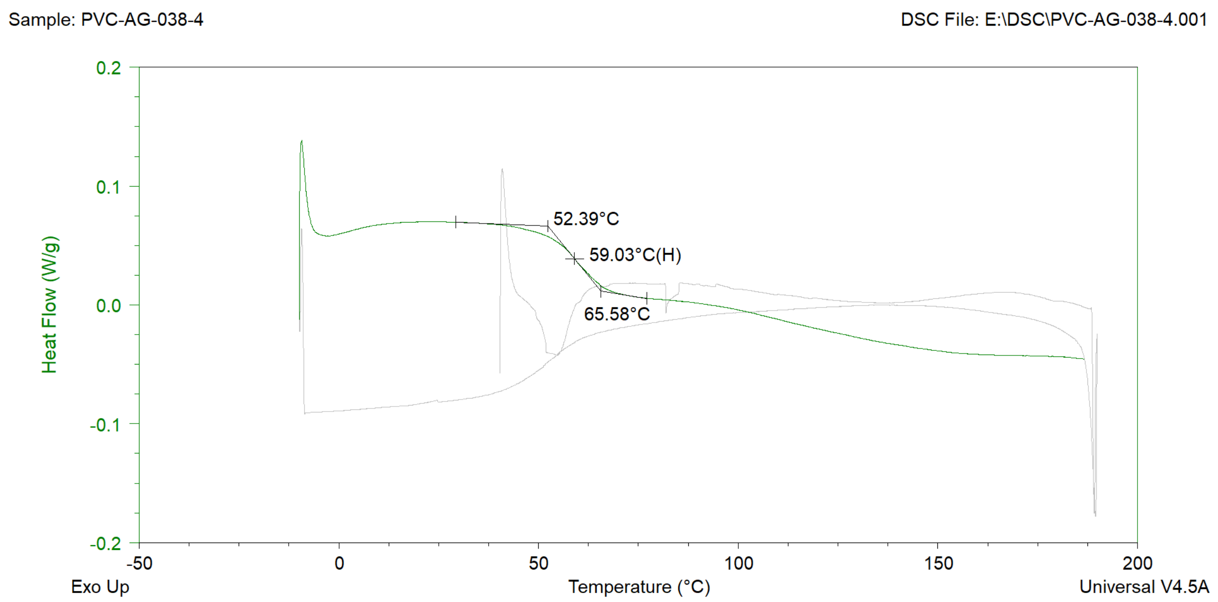

Figure S181: DSC thermogram of 12b (10 wt%) in PVC with 2nd heating cycle in green (4th measurement).

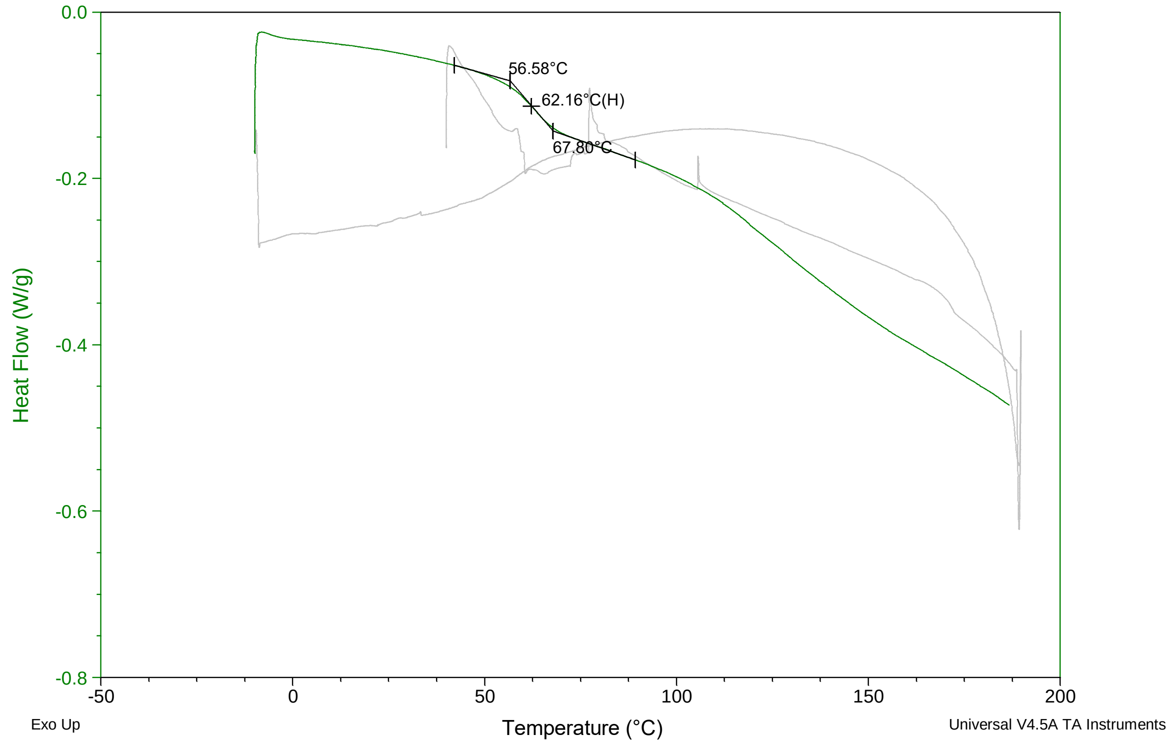

Figure S182: DSC thermogram of 12c (10 wt%) in PVC with 2nd heating cycle in green.

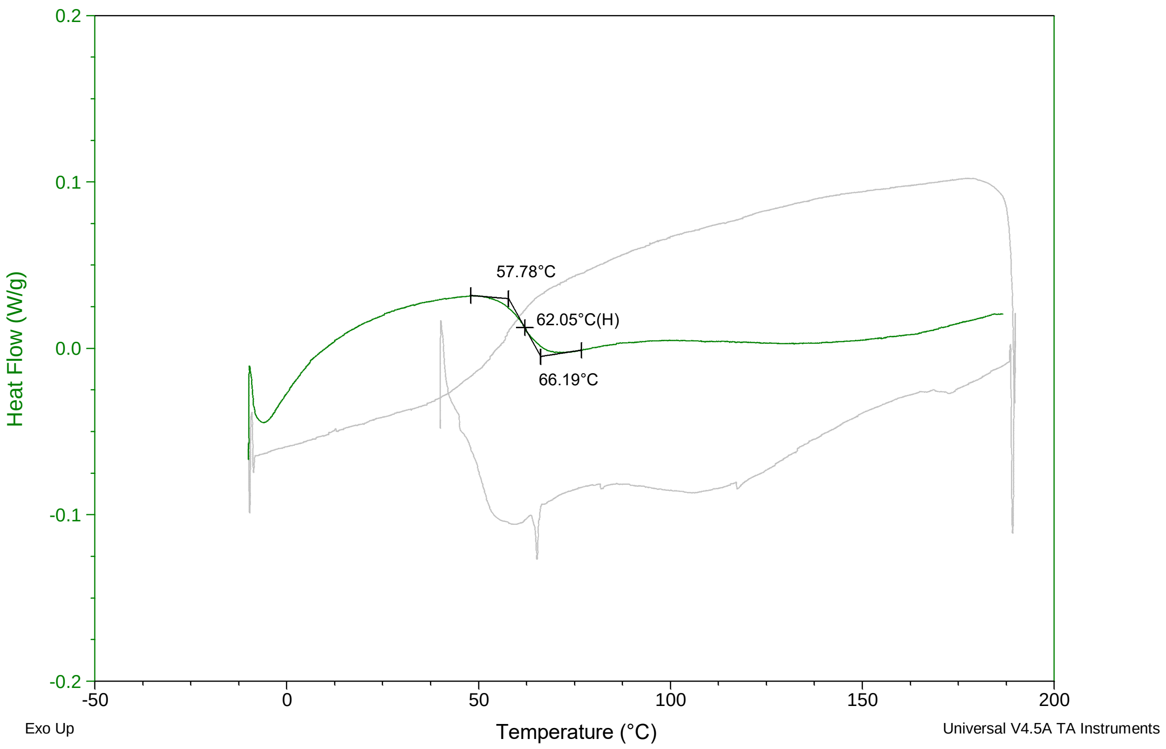

Figure S183: DSC thermogram of 12d (10 wt%) in PVC with 2nd heating cycle in green.

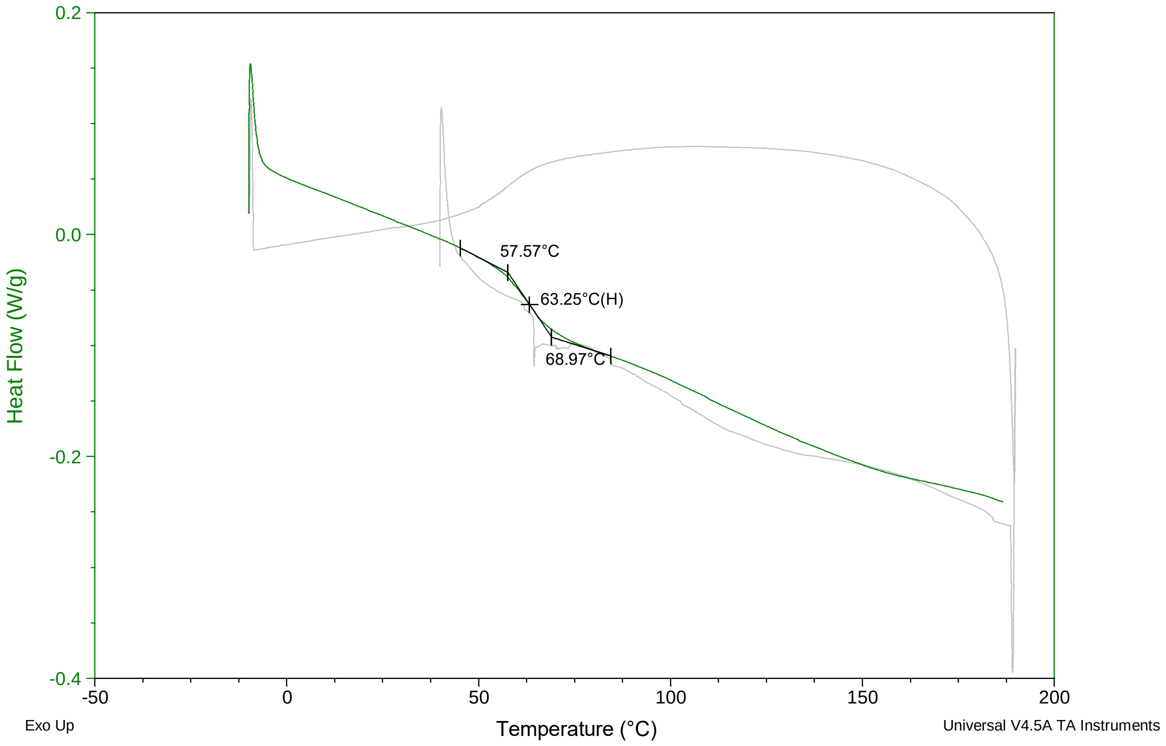

Figure S184: DSC thermogram of model mixture of 12a-12d [AGO-2024-0003 before filtration over silica] (10 wt%) in PVC with 2nd heating cycle in green.

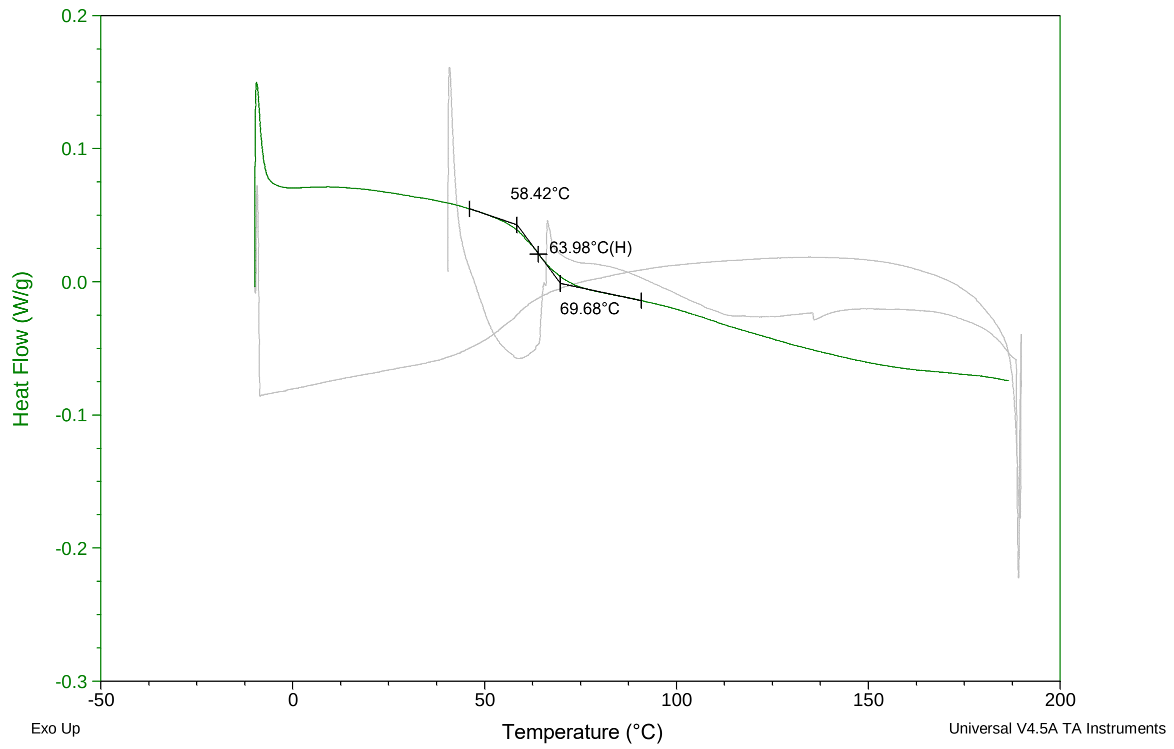

Figure S185: DSC thermogram of model mixture of 12a-12d [AGO-2024-0003 before filtration over silica] (10 wt%) in PVC with 2nd heating cycle in green (2nd measurement).

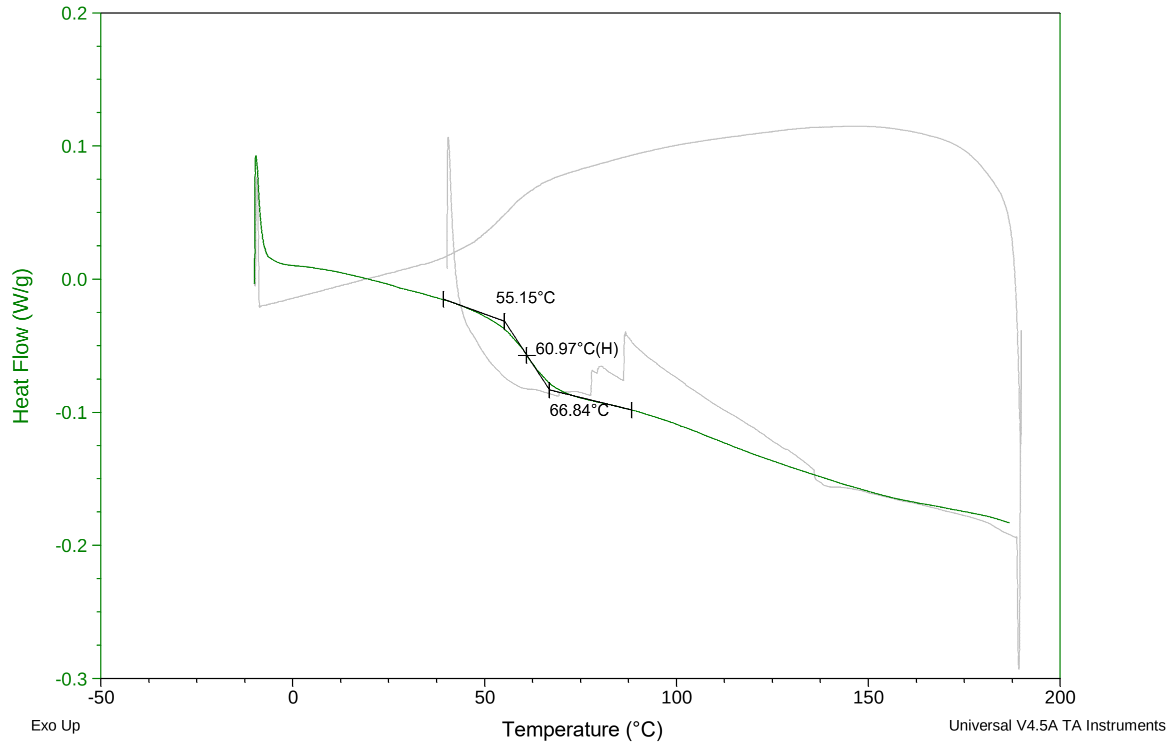

Figure S186: DSC thermogram of model mixture of 12a-12d [AGO-2024-0003 after filtration over silica] (10 wt%) in PVC with 2nd heating cycle in green.

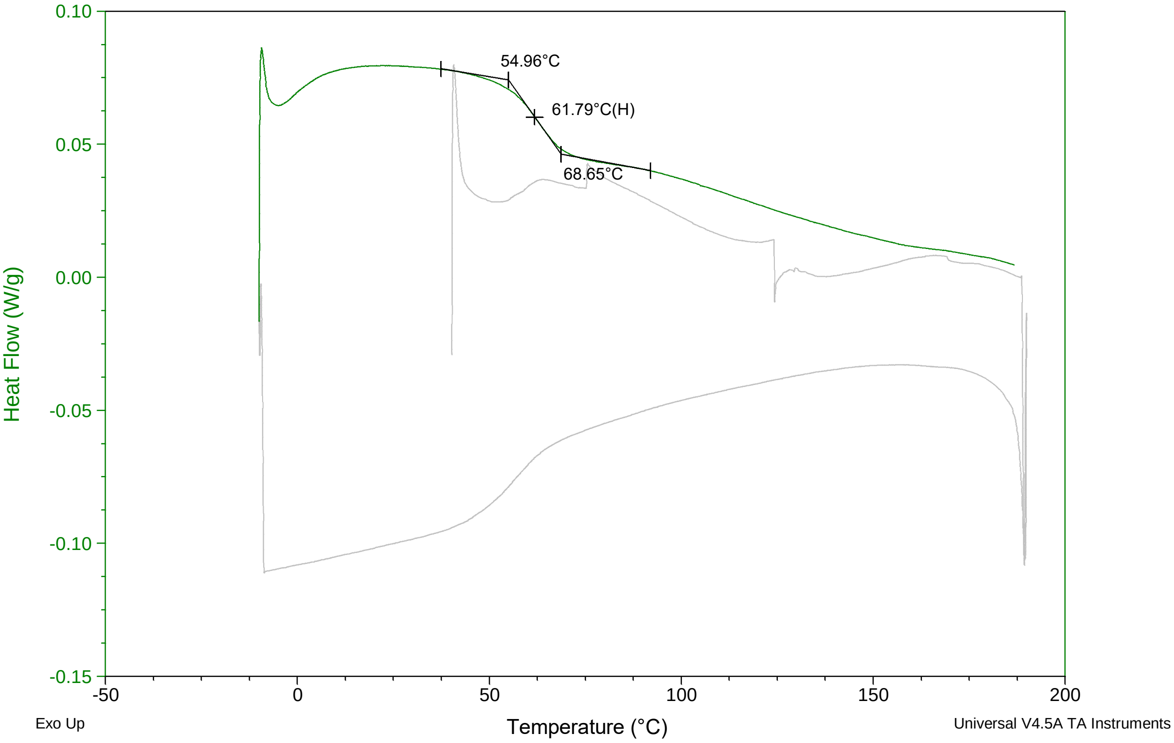

Figure S187: DSC thermogram of model mixture of 12a-12d [AGO-2024-0003 after filtration over silica] (10 wt%) in PVC with 2nd heating cycle in green (2nd measurement).

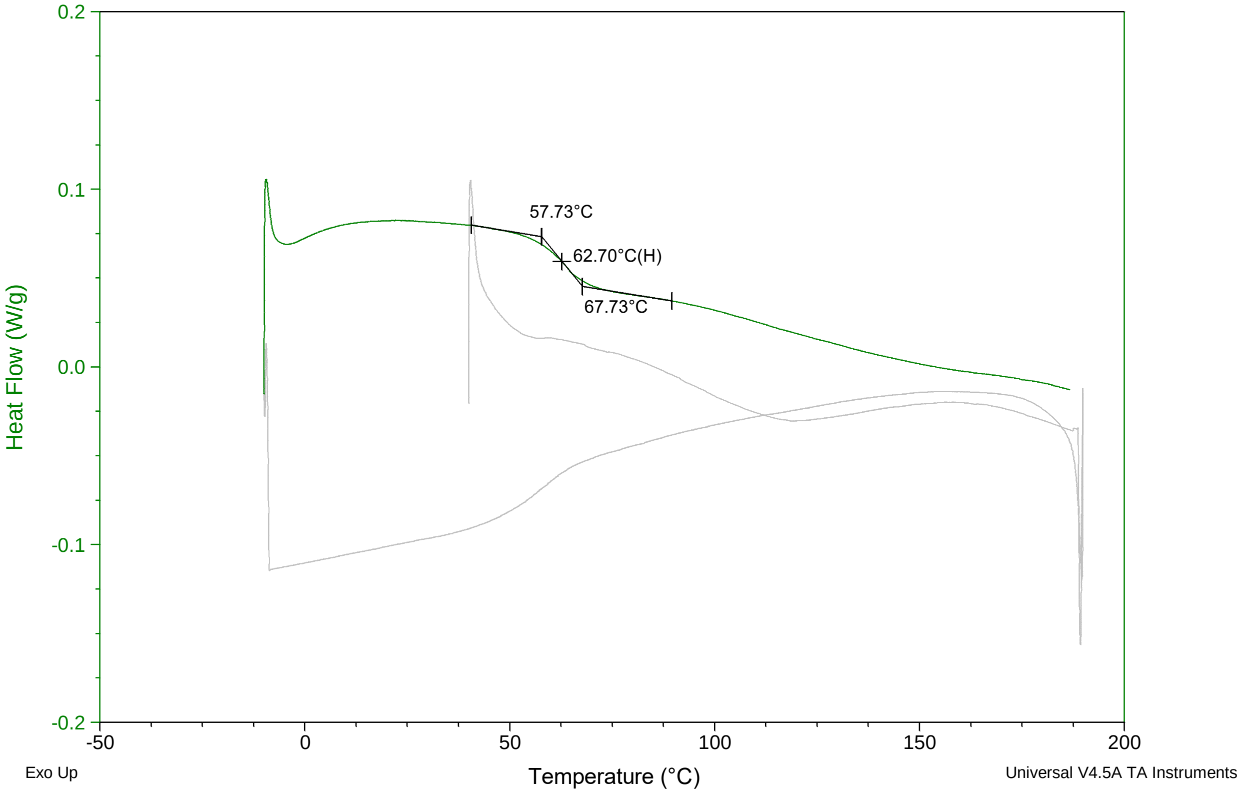

Figure S188: DSC thermogram of ball mill-derived mixture of 12a-12d [AGO-2024-0012] (10 wt%) in PVC with 2nd heating cycle in green.

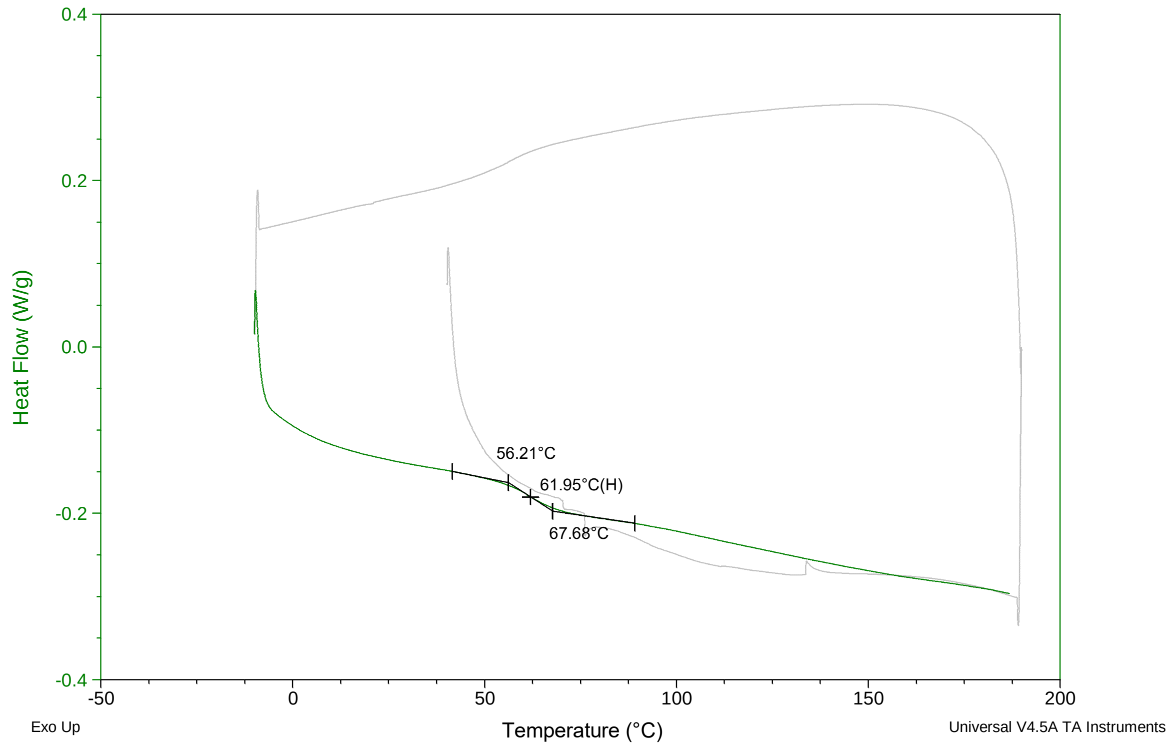

Figure S189: DSC thermogram of ball mill-derived mixture of 12a-12d [AGO-2024-0012] (10 wt%) in PVC with 2nd heating cycle in green (2nd measurement).

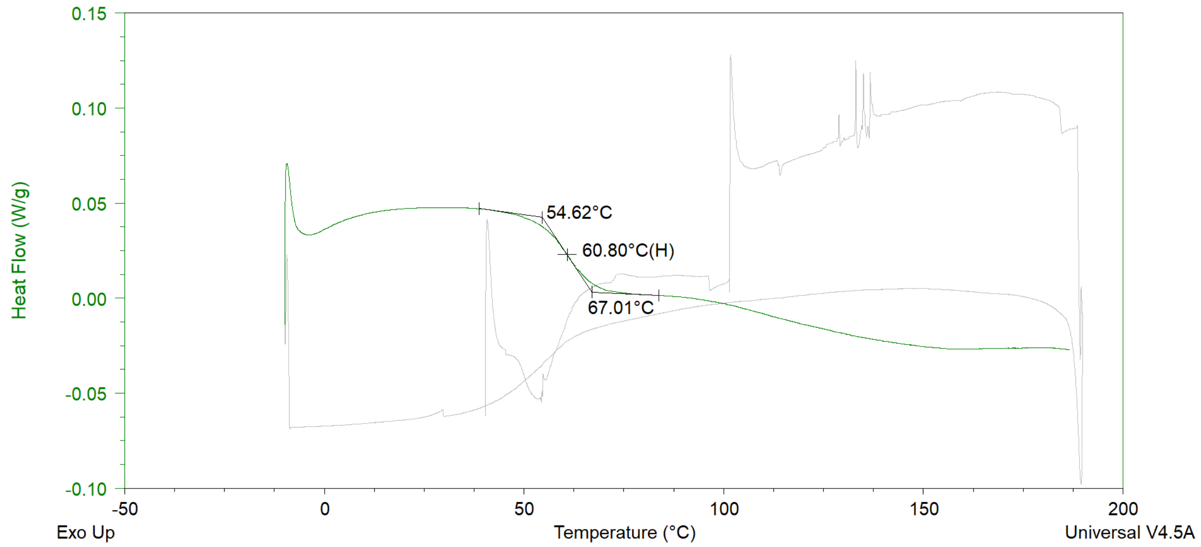

Figure S190: DSC thermogram of ball mill-derived mixture of 12a-12d [AGO-2024-0012] (10 wt%) in PVC with 2nd heating cycle in green (3rd measurement).

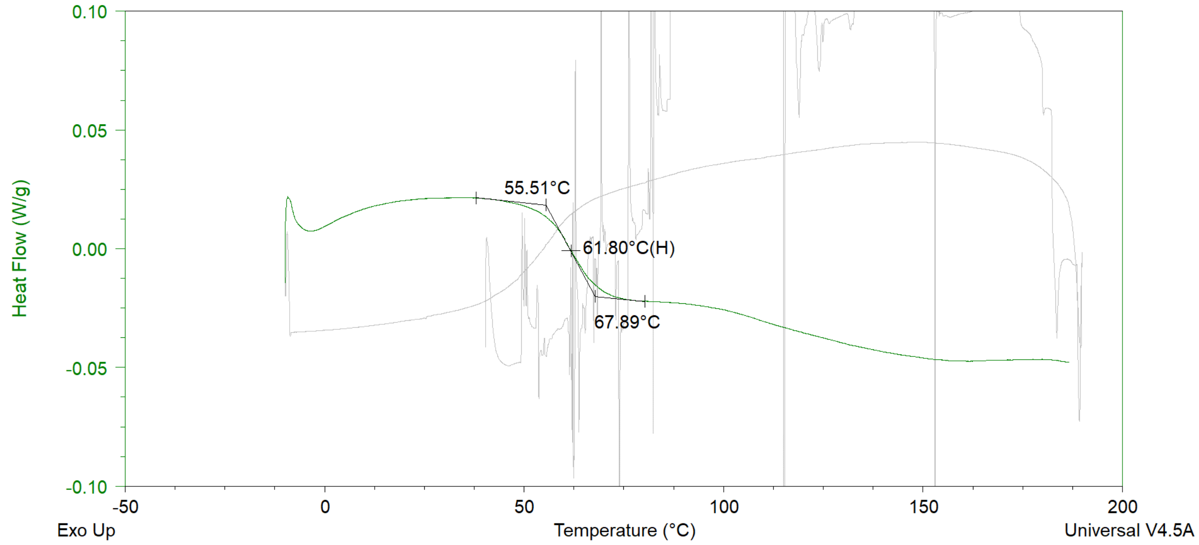

Figure S191: DSC thermogram of ball mill-derived mixture of 12a-12d [AGO-2024-0012] (10 wt%) in PVC with 2nd heating cycle in green (4th measurement).

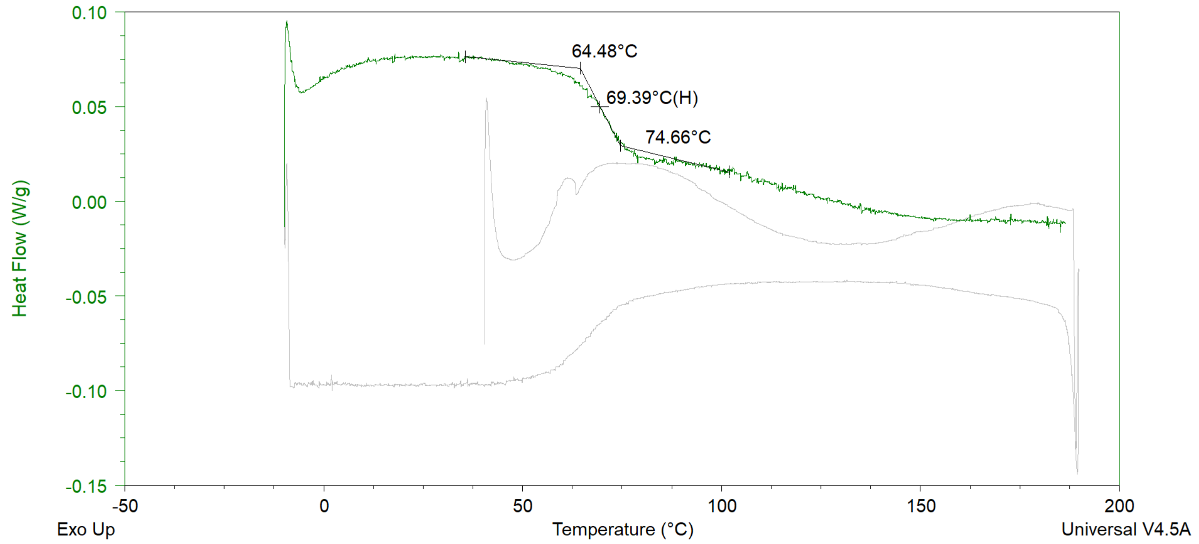

Figure S192: DSC thermogram of DEHP (5 wt%) in PVC with 2nd heating cycle in green.

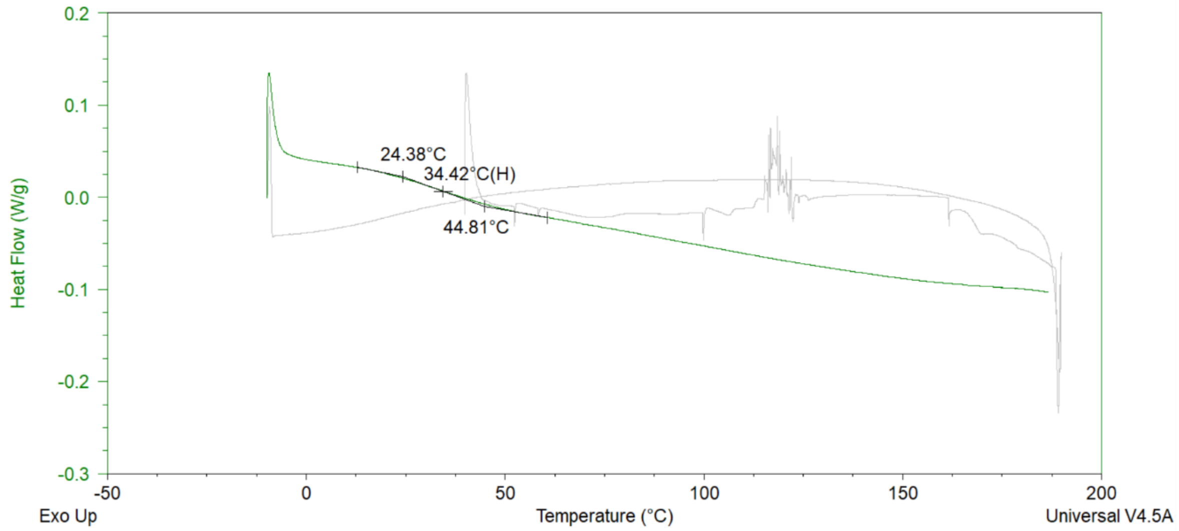

Figure S193: DSC thermogram of DEHP (20 wt%) in PVC with 2nd heating cycle in green.

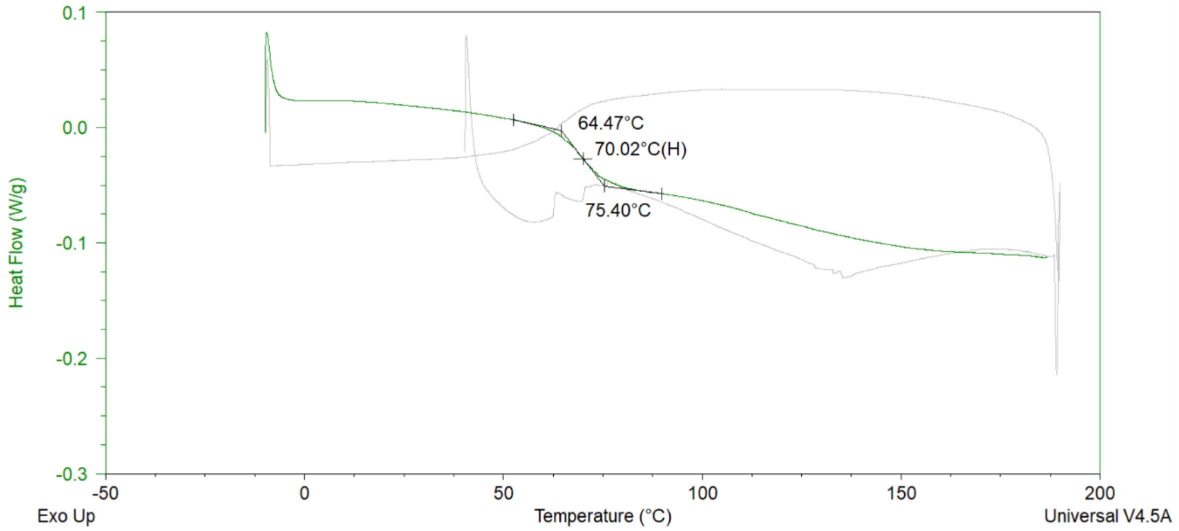

Figure S194: DSC thermogram of DINCH (5 wt%) in PVC with 2nd heating cycle in green.

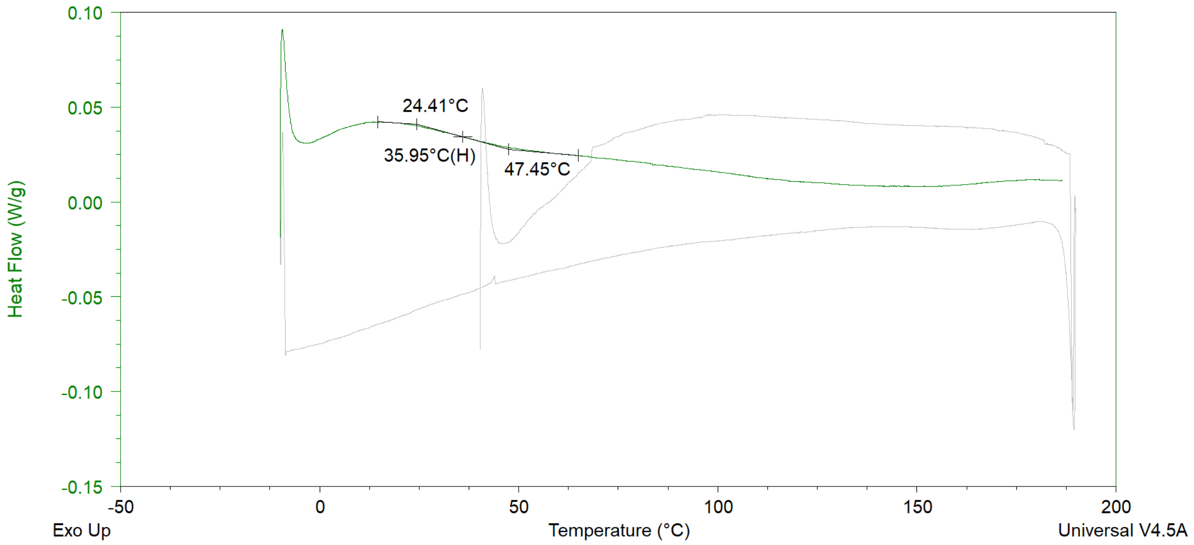

Figure S195: DSC thermogram of DINCH (20 wt%) in PVC with 2nd heating cycle in green.

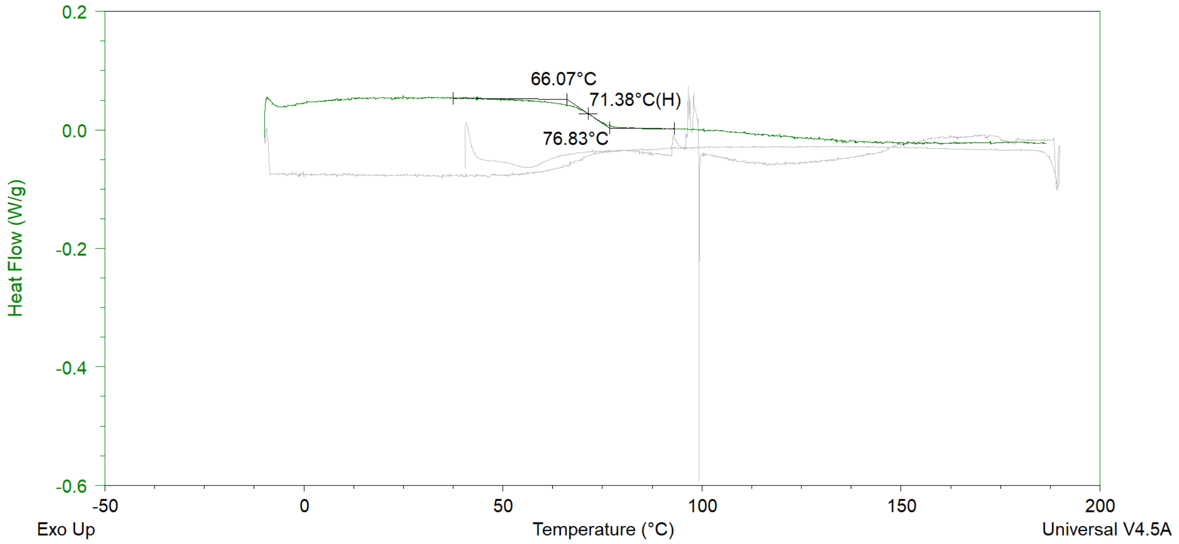

Figure S196: DSC thermogram of model mixture of 12a-12d [DDV-2025-0247 after filtration over silica] (5 wt%) in PVC with 2nd heating cycle in green.

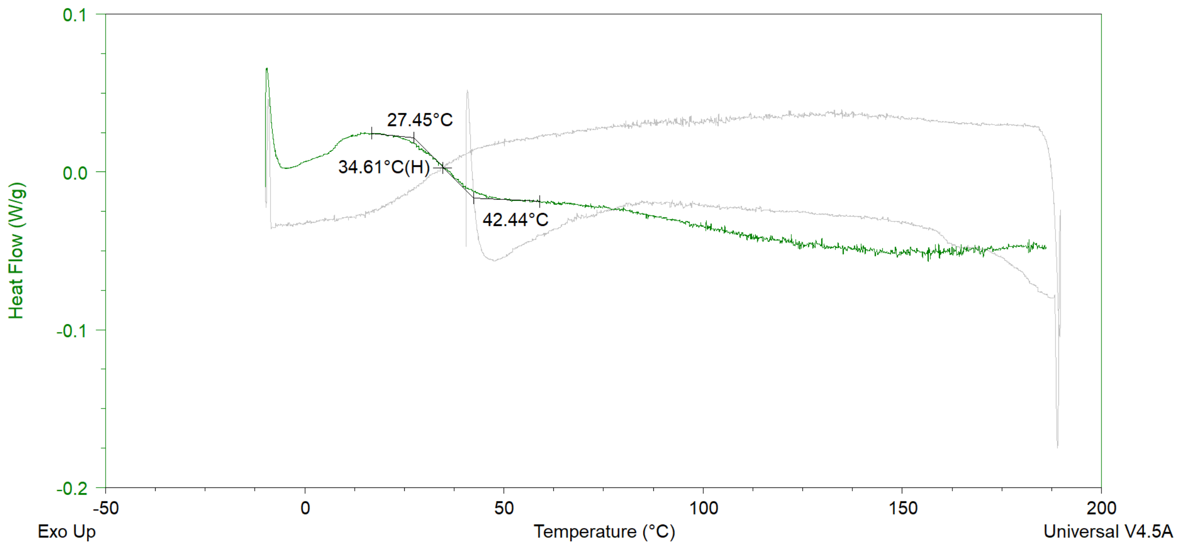

Figure S197: DSC thermogram of model mixture of 12a-12d [DDV-2025-0247 after filtration over silica] (20 wt%) in PVC with 2nd heating cycle in green.

14.2 DSC thermograms in PLA

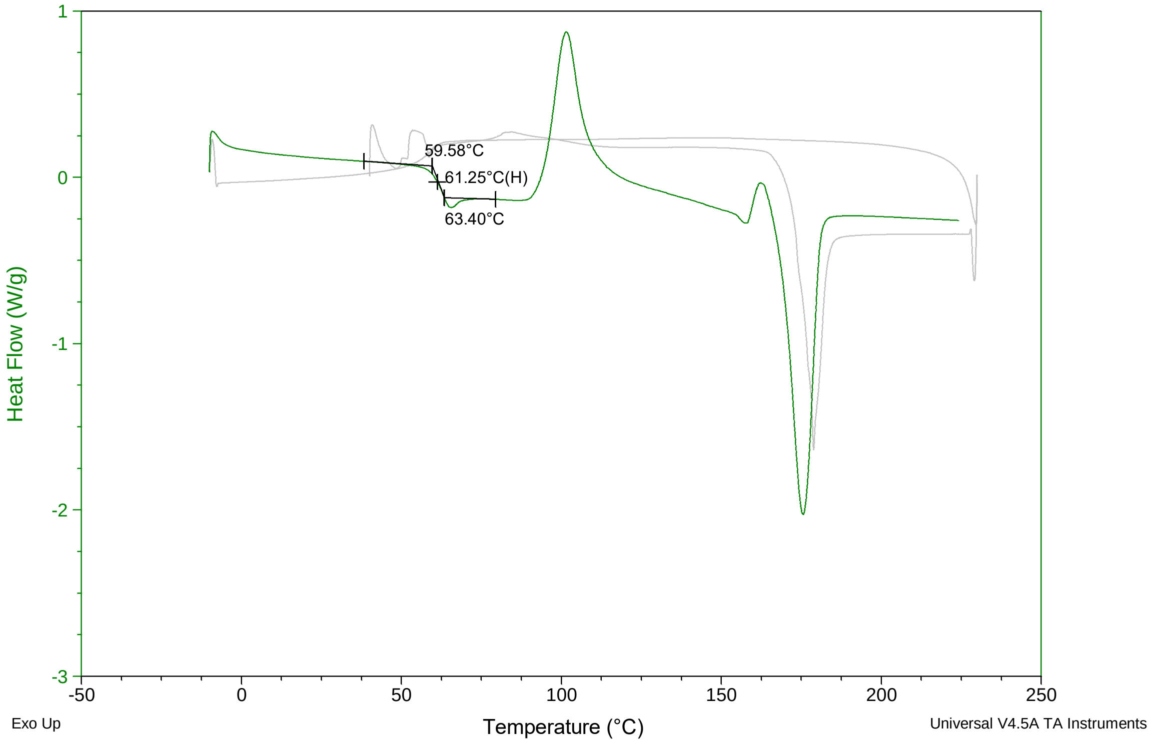

Figure S198: DSC thermogram of unplasticized PLA with 2nd heating cycle in green.

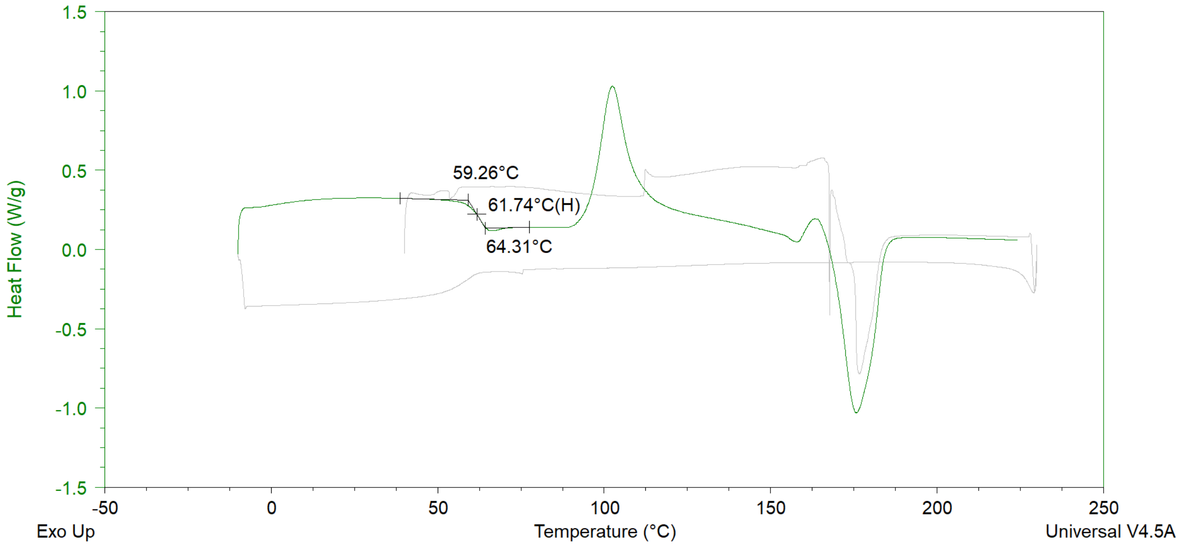

Figure S199: DSC thermogram of unplasticized PLA with 2nd heating cycle in green (2nd measurement).

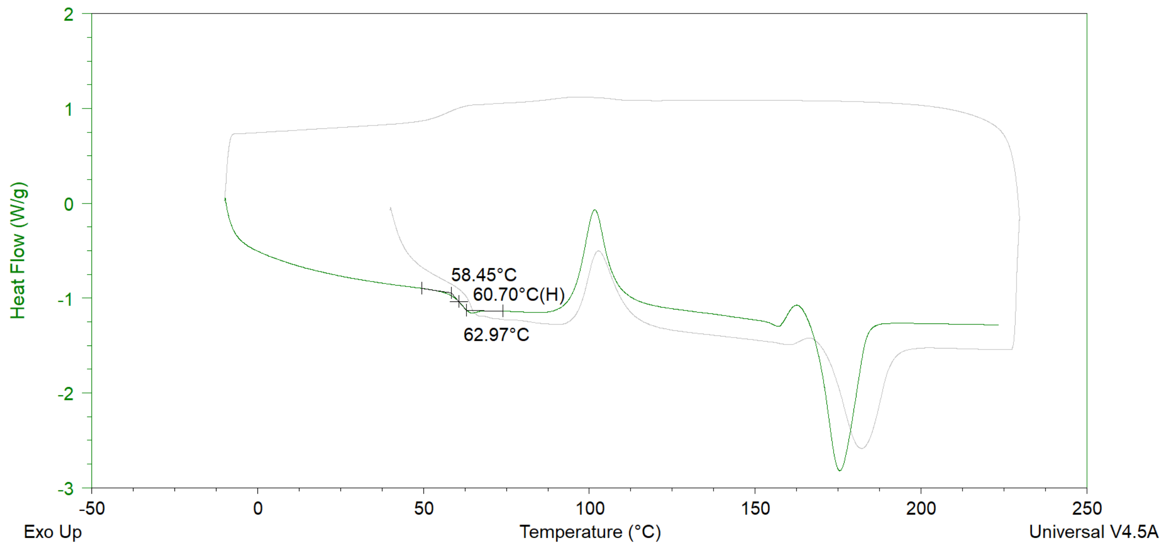

Figure S200: DSC thermogram of unplasticized PLA with 2nd heating cycle in green (3rd measurement).

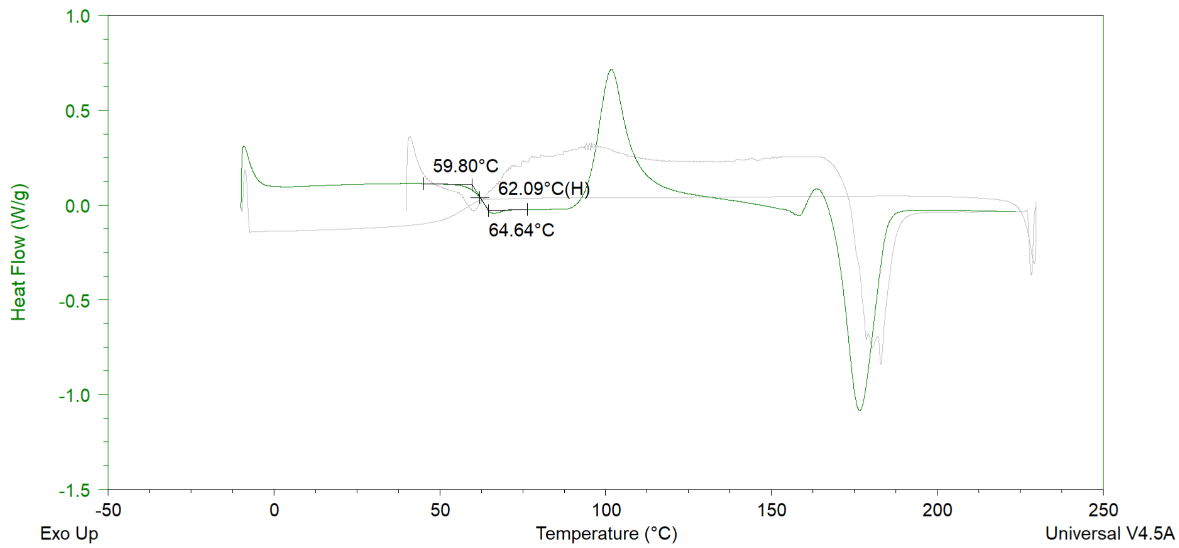

Figure S201: DSC thermogram of unplasticized PLA with 2nd heating cycle in green (4th measurement).

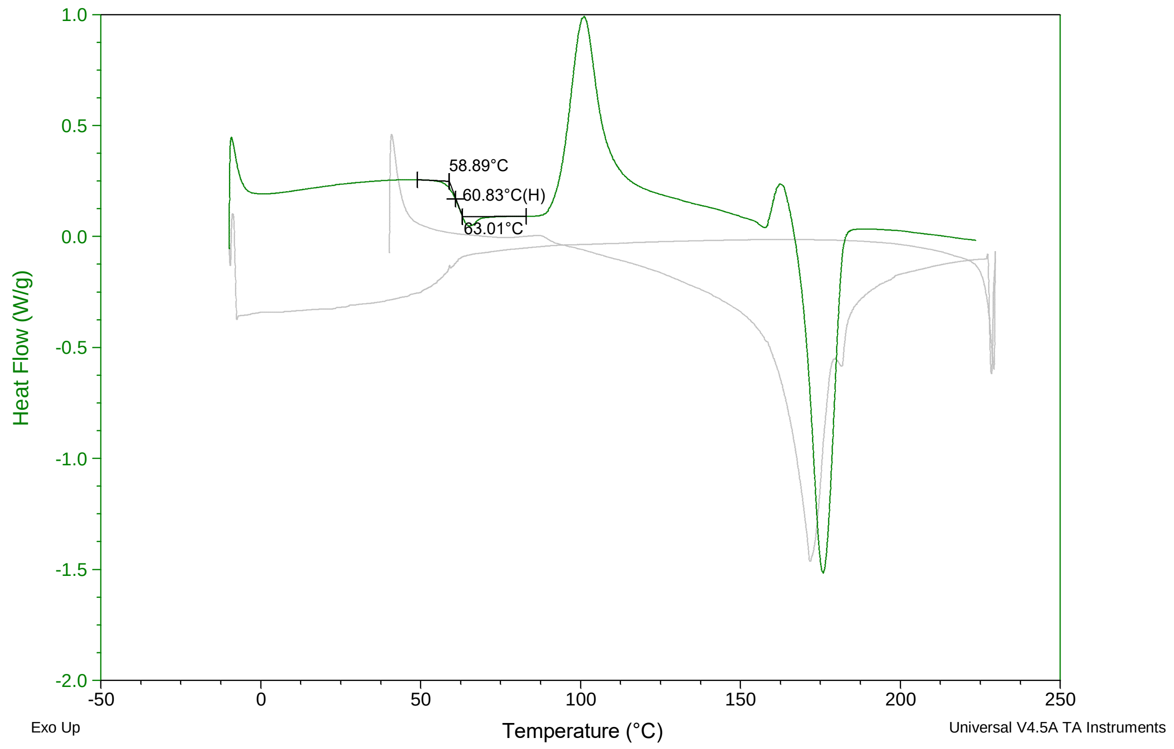

Figure S202: DSC thermogram of TEC (10 wt%) in PLA with 2nd heating cycle in green.

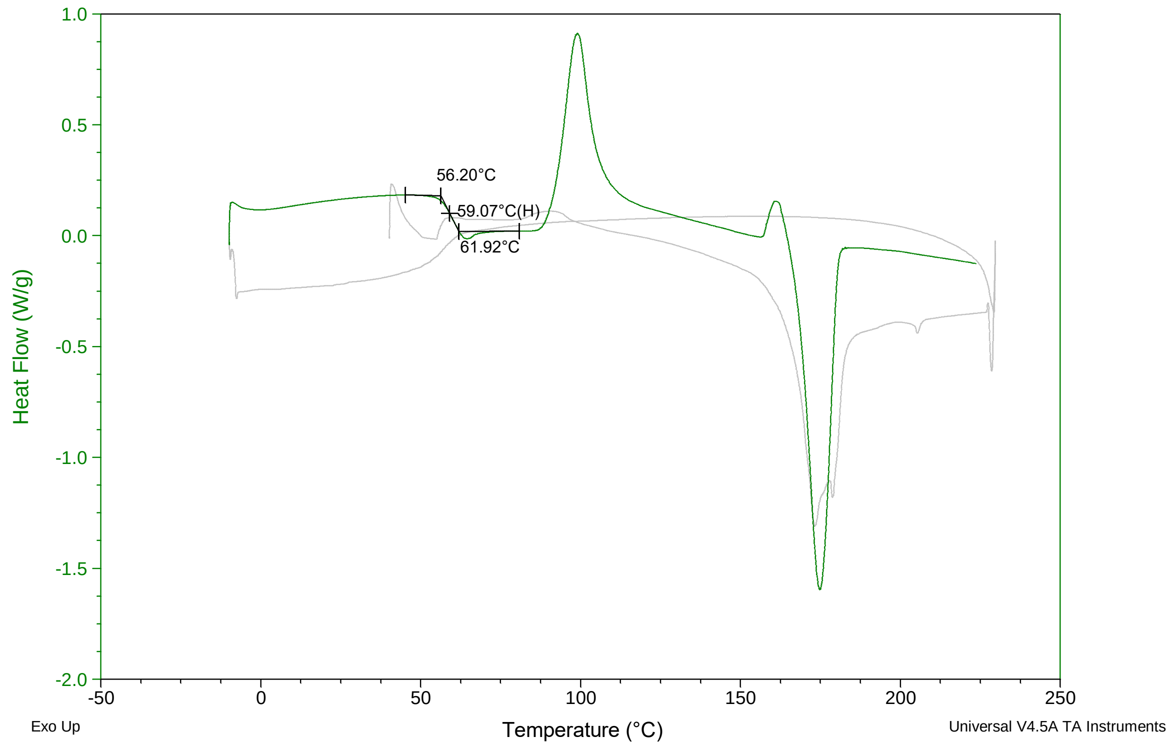

Figure S203: DSC thermogram of TBC (10 wt%) in PLA with 2nd heating cycle in green.

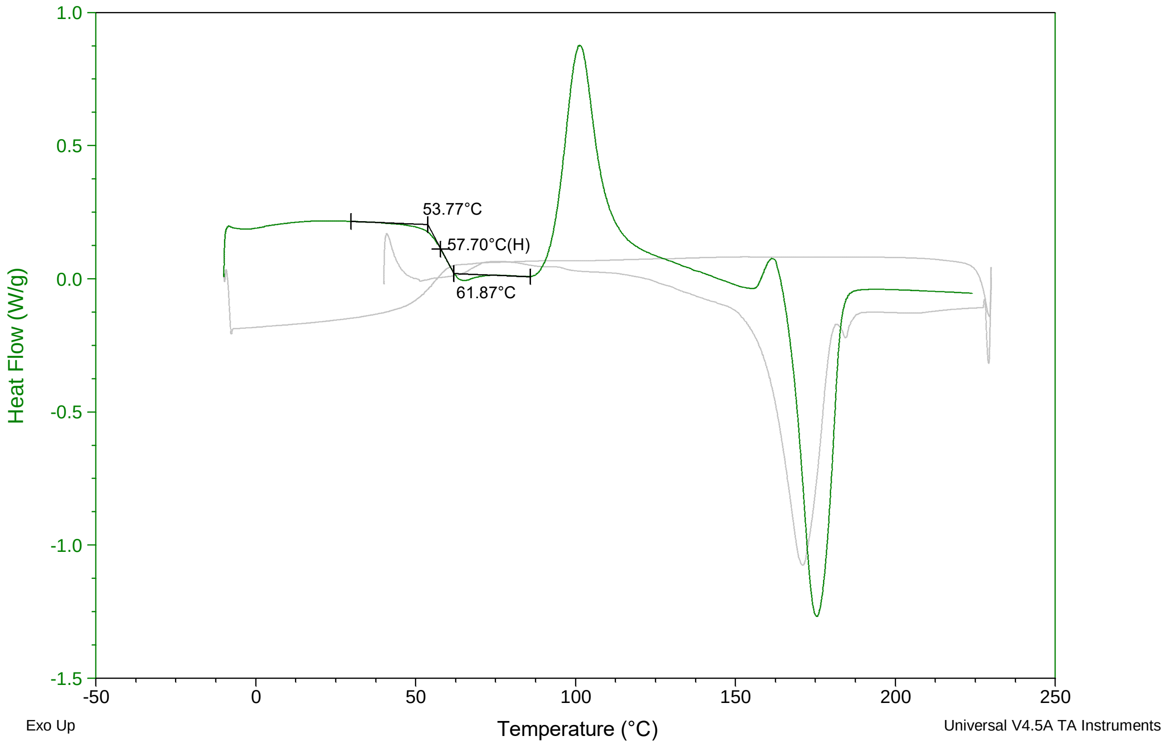

Figure S204: DSC thermogram of TEAC (10 wt%) in PLA with 2nd heating cycle in green.

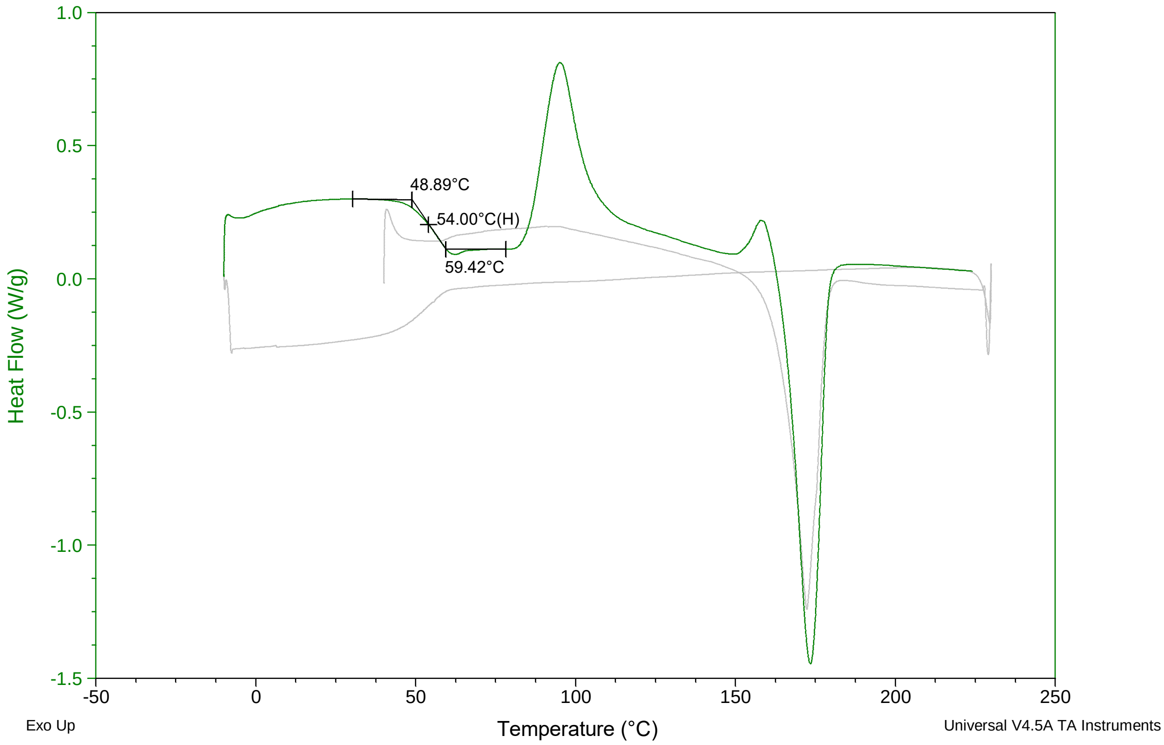

Figure S205: DSC thermogram of TBAC (10 wt%) in PLA with 2nd heating cycle in green.

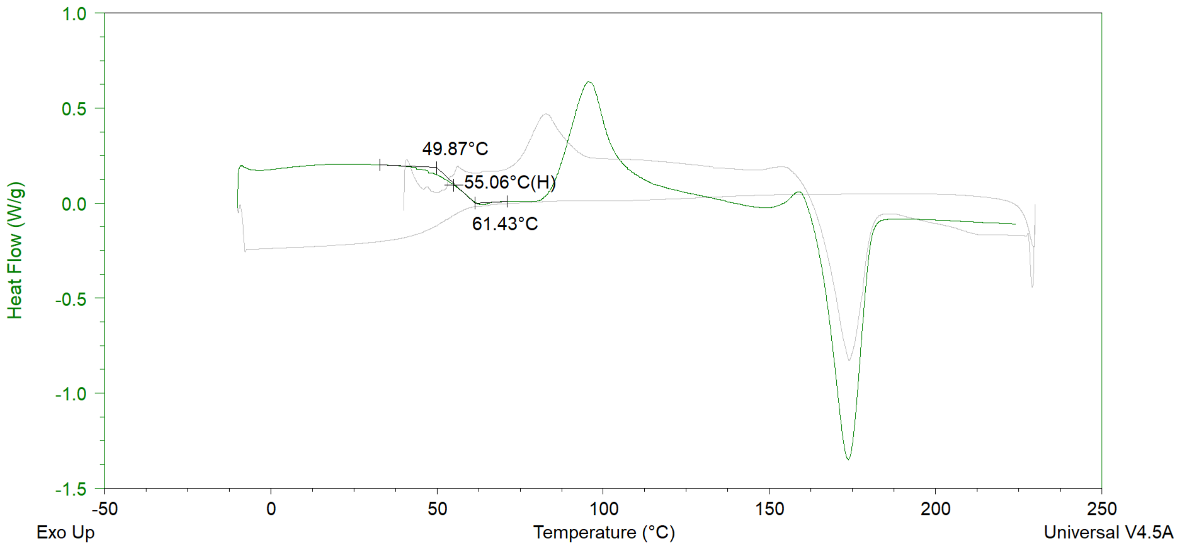

Figure S206: DSC thermogram of TBAC (10 wt%) in PLA with 2nd heating cycle in green (2nd measurement).

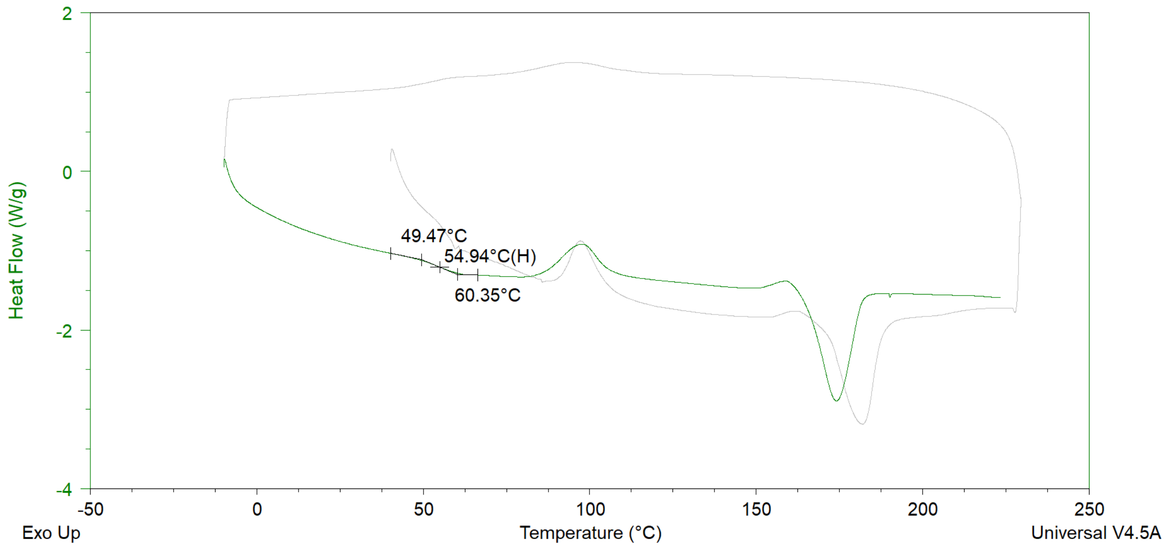

Figure S207: DSC thermogram of TBAC (10 wt%) in PLA with 2nd heating cycle in green (3rd measurement).

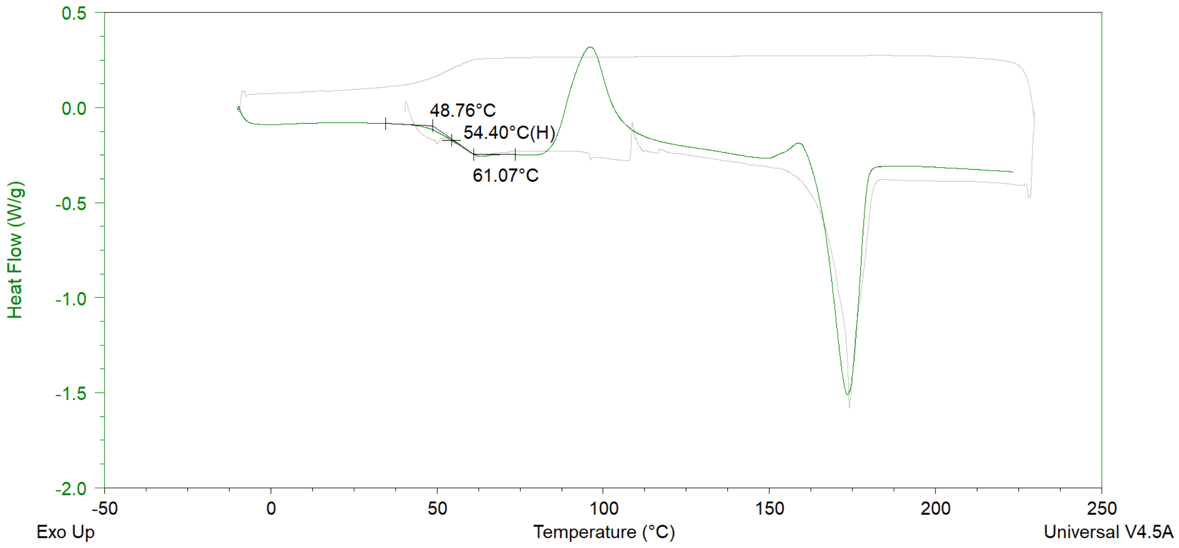

Figure S208: DSC thermogram of TBAC (10 wt%) in PLA with 2nd heating cycle in green (4th measurement).

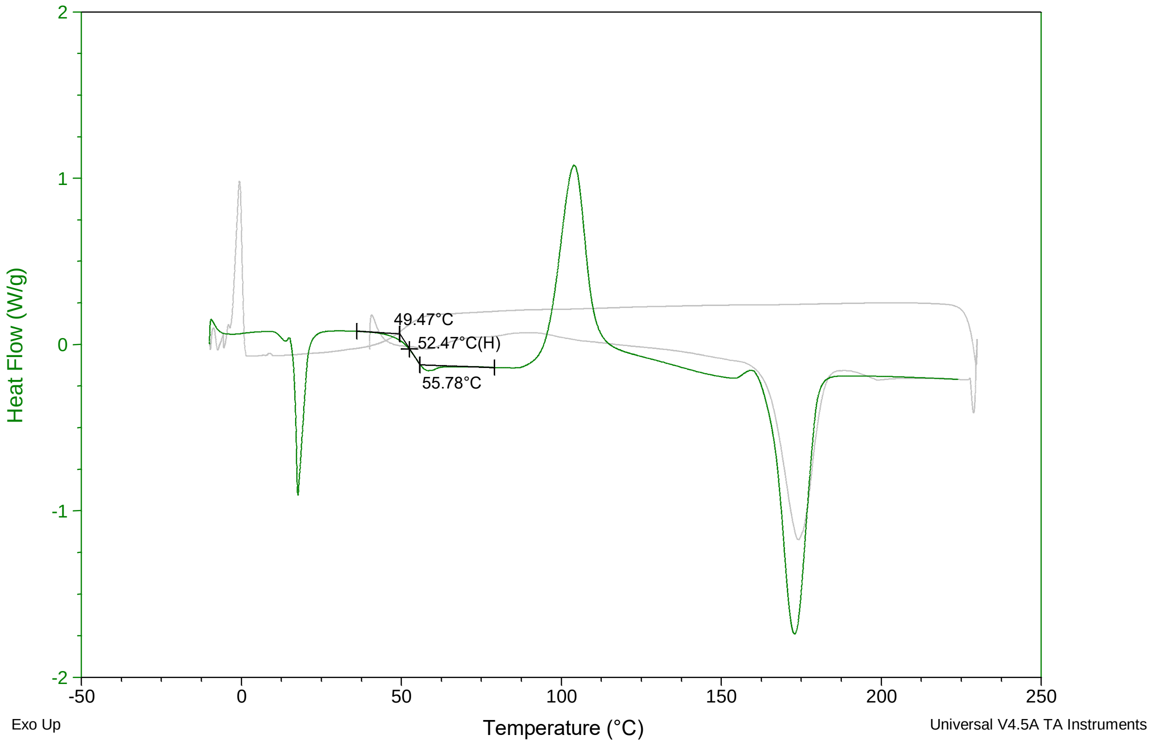

Figure S209: DSC thermogram of DOS (10 wt%) in PLA with 2nd heating cycle in green.

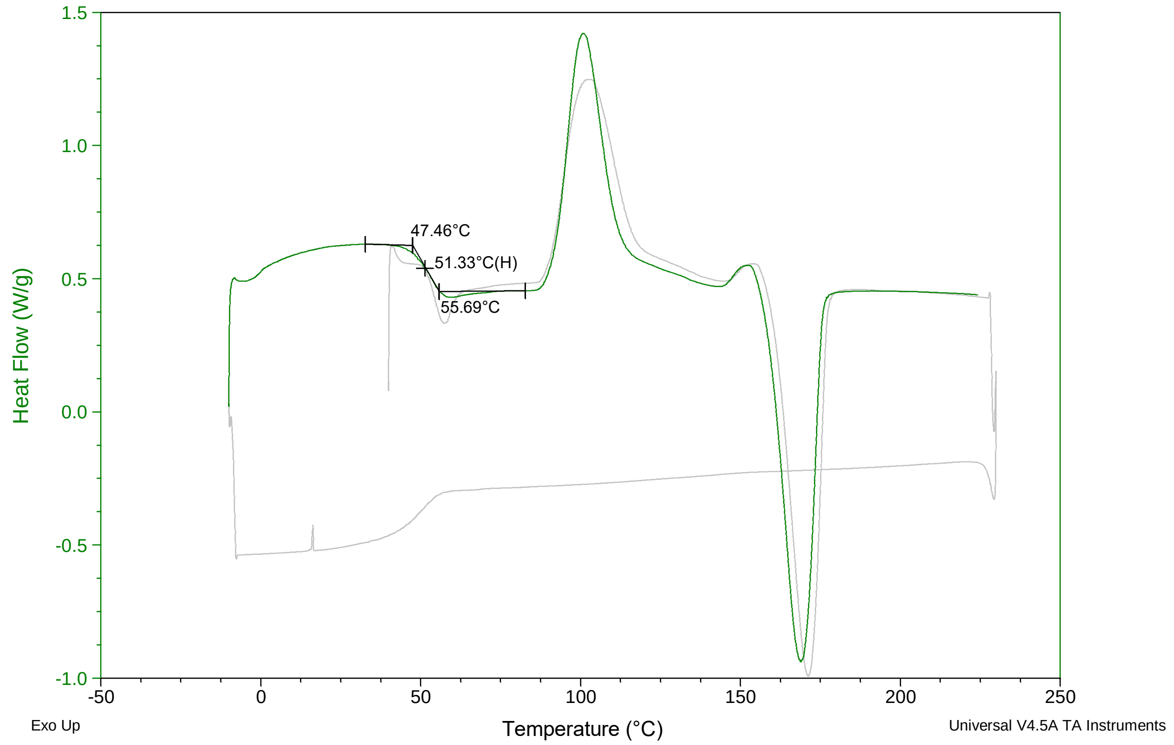

Figure S210: DSC thermogram of ESO (10 wt%) in PLA with 2nd heating cycle in green.

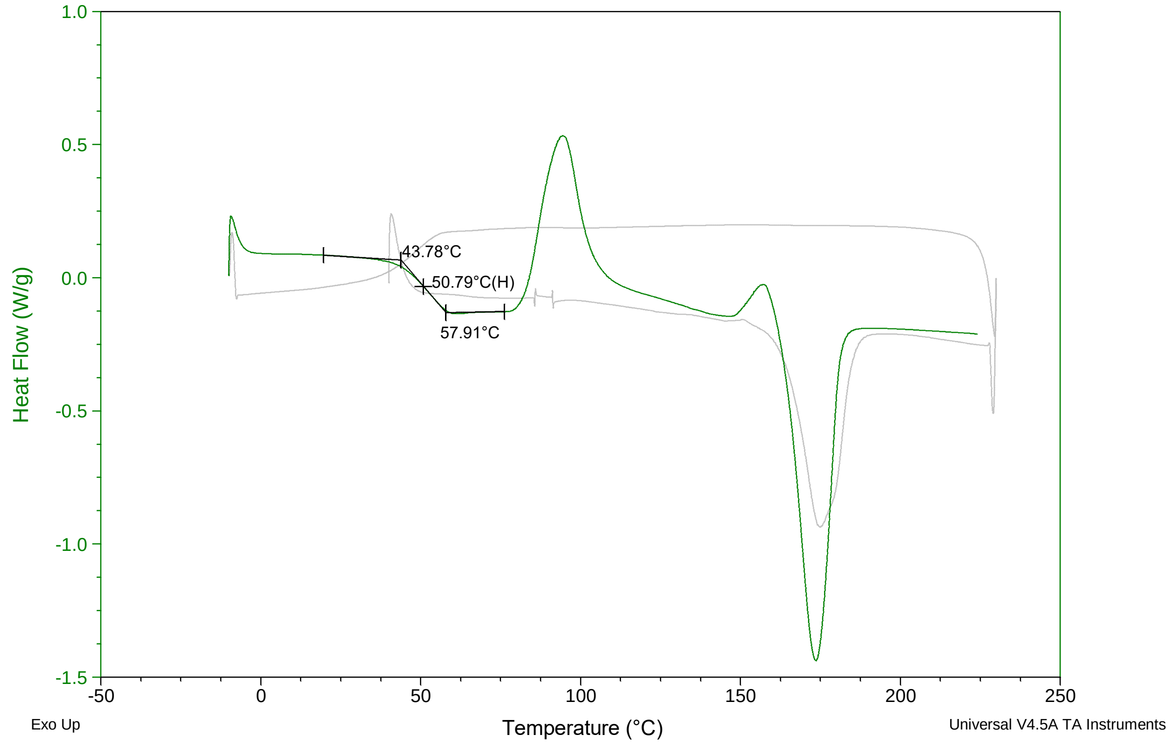

Figure S211: DSC thermogram of DEHP (10 wt%) in PLA with 2nd heating cycle in green.

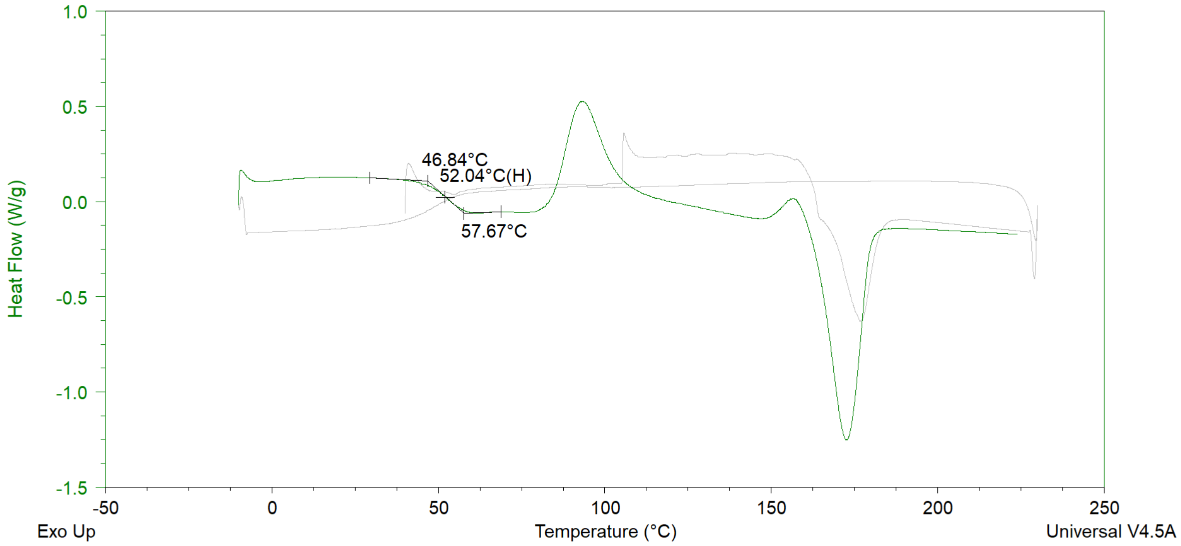

Figure S212: DSC thermogram of DEHP (10 wt%) in PLA with 2nd heating cycle in green (2nd measurement).

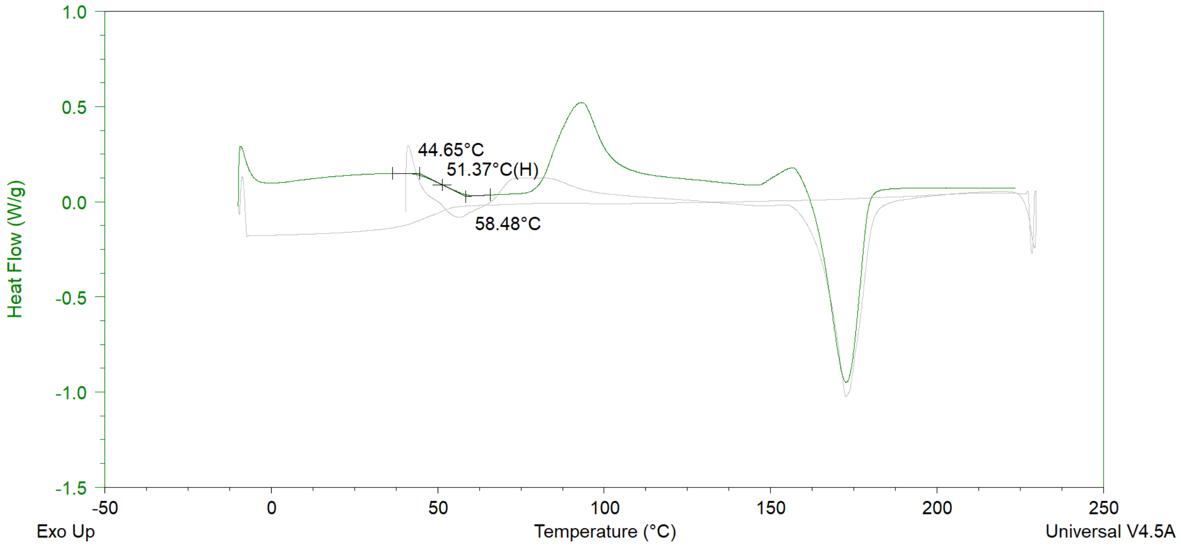

Figure S213: DSC thermogram of DEHP (10 wt%) in PLA with 2nd heating cycle in green (3th measurement).

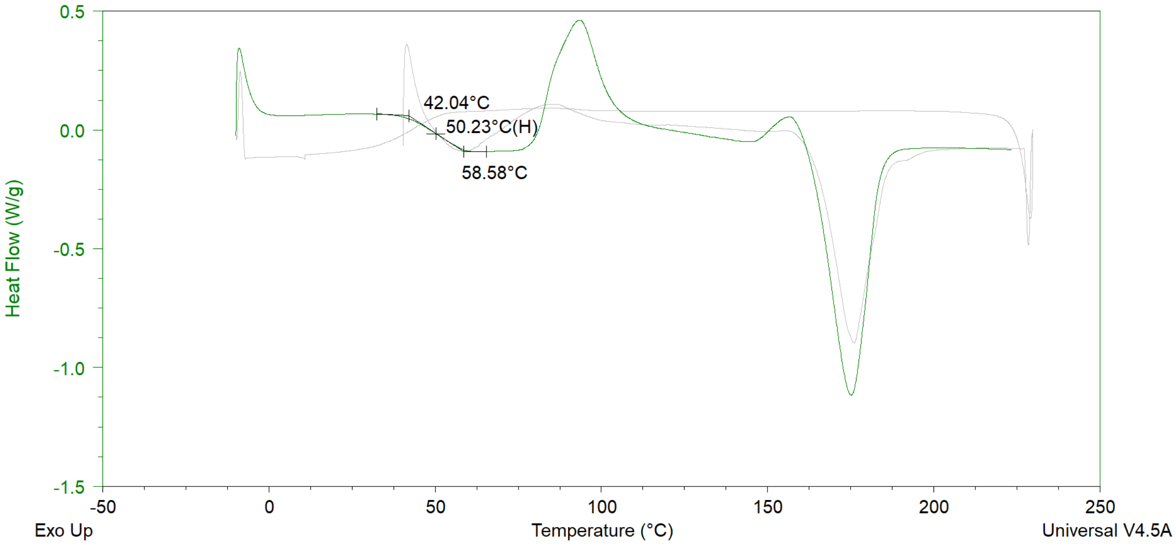

Figure S214: DSC thermogram of DEHP (10 wt%) in PLA with 2nd heating cycle in green (4th measurement).

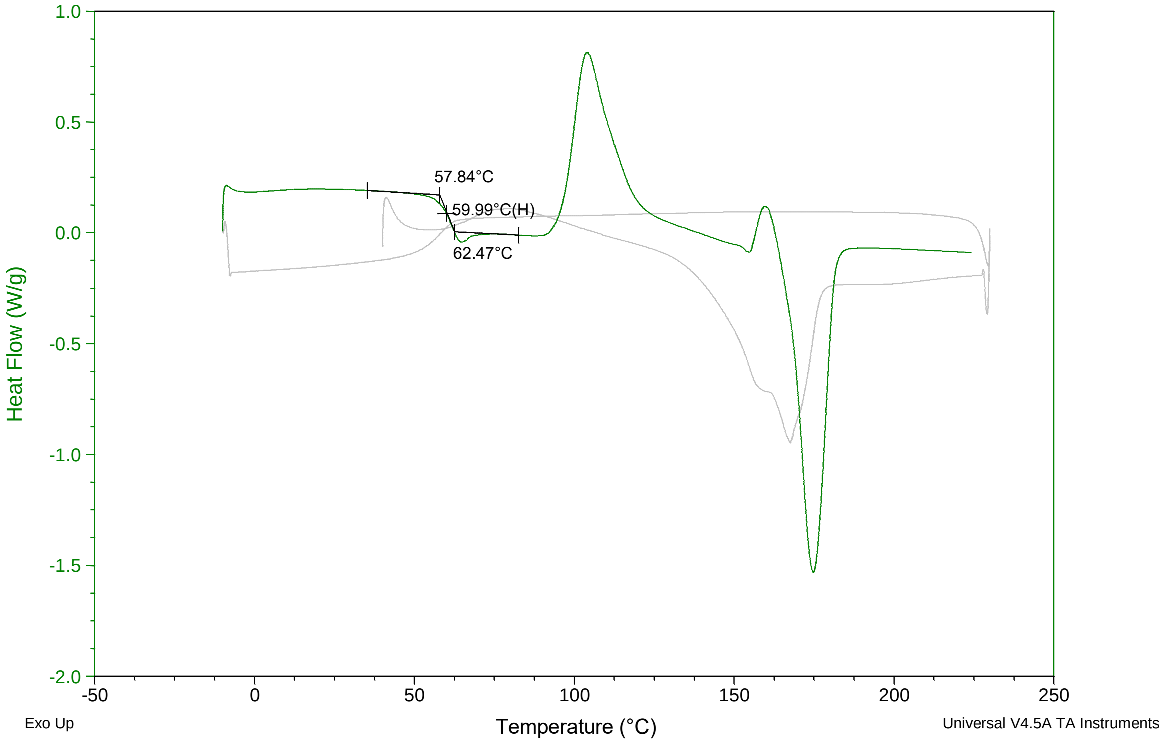

Figure S215: DSC thermogram of 2a (10 wt%) in PLA with 2nd heating cycle in green.

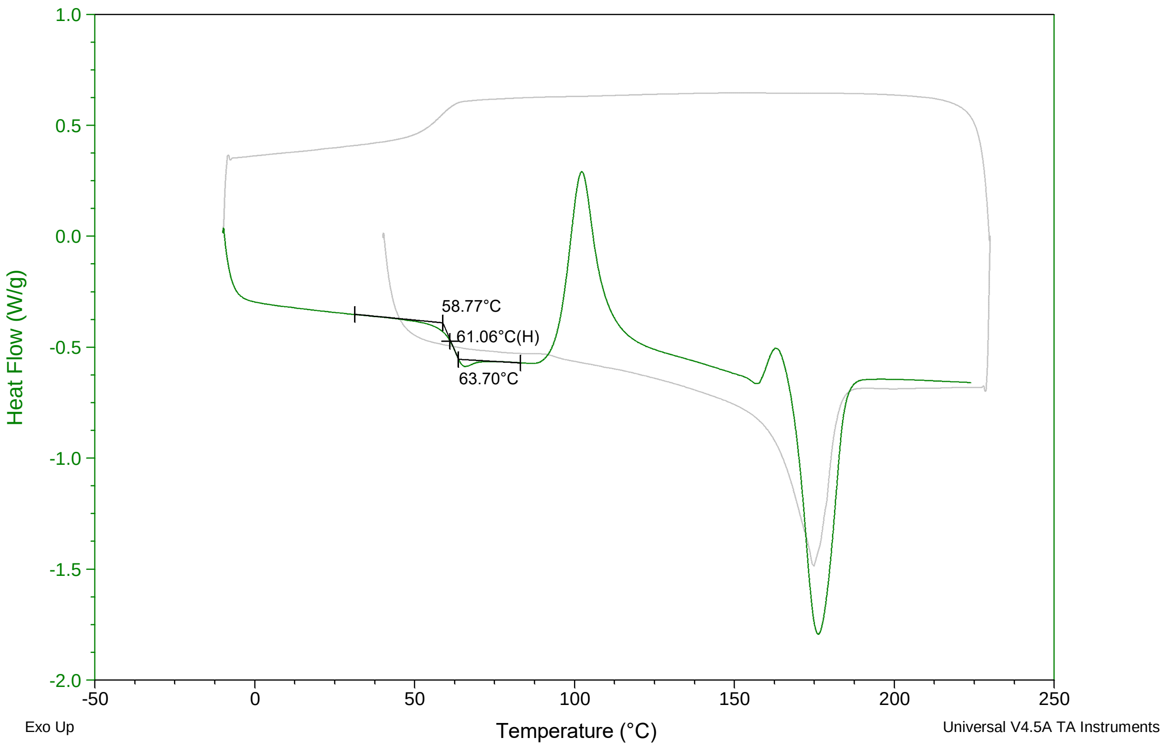

Figure S216: DSC thermogram of 3a (10 wt%) in PLA with 2nd heating cycle in green.

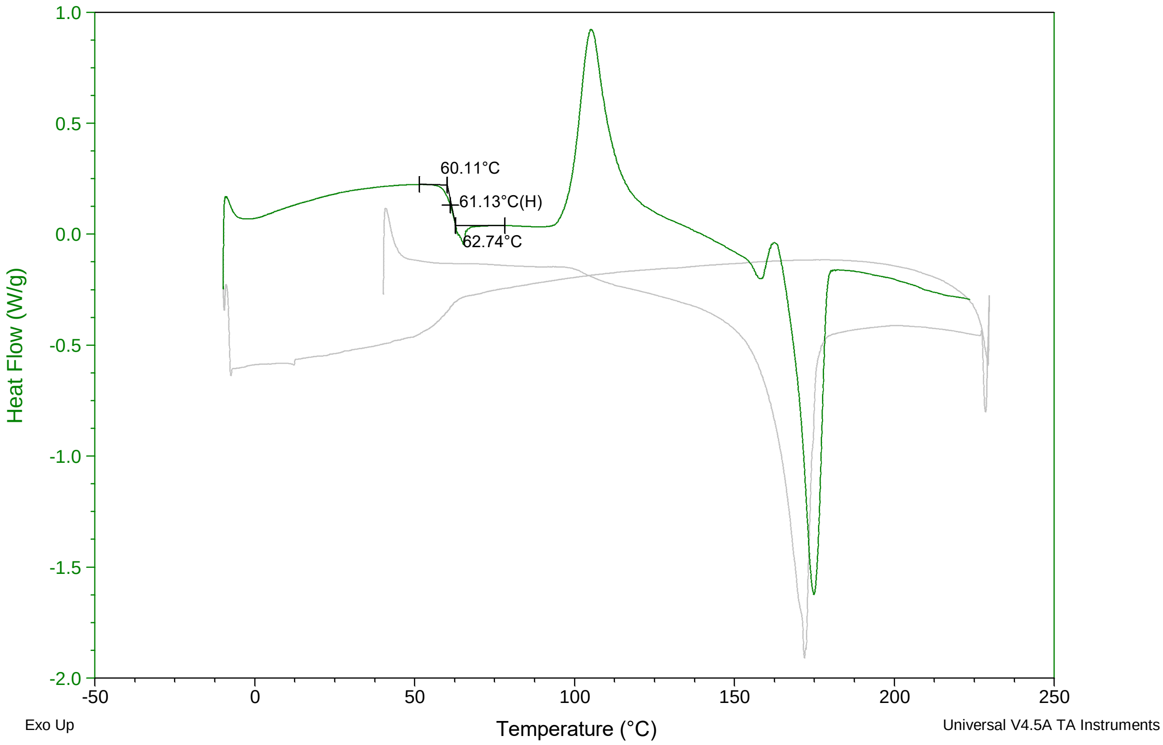

Figure S217: DSC thermogram of 4a (10 wt%) in PLA with 2nd heating cycle in green.

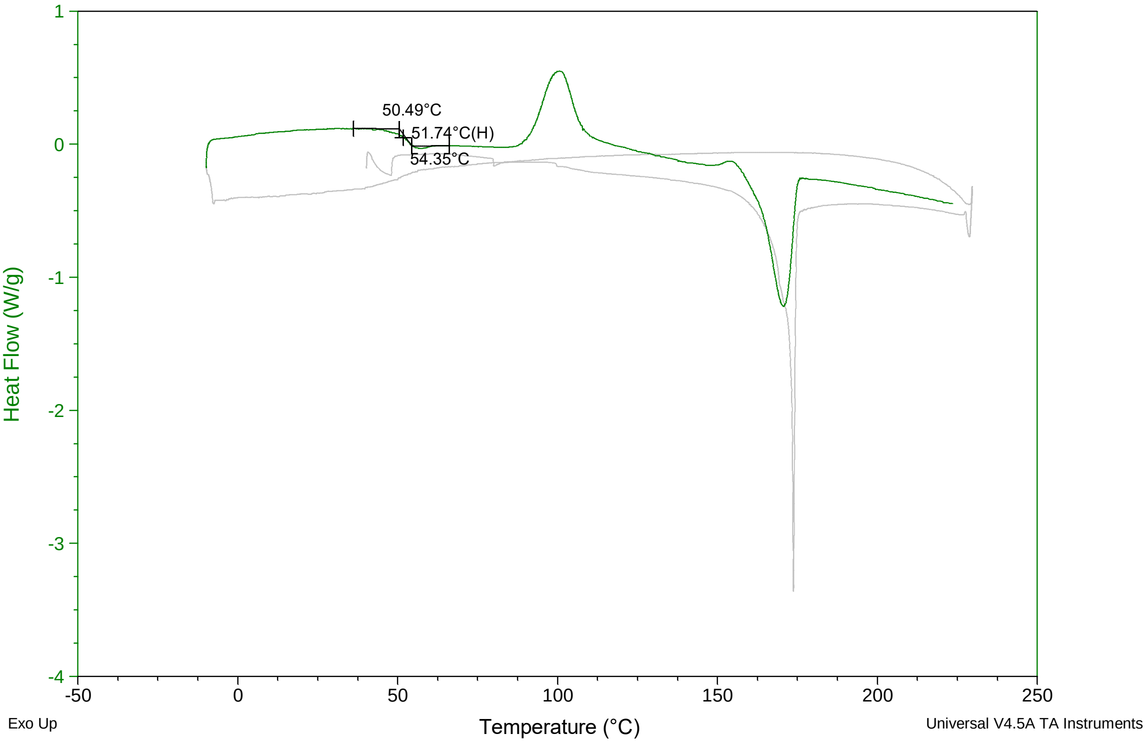

Figure S218: DSC thermogram of 5a (10 wt%) in PLA with 2nd heating cycle in green.

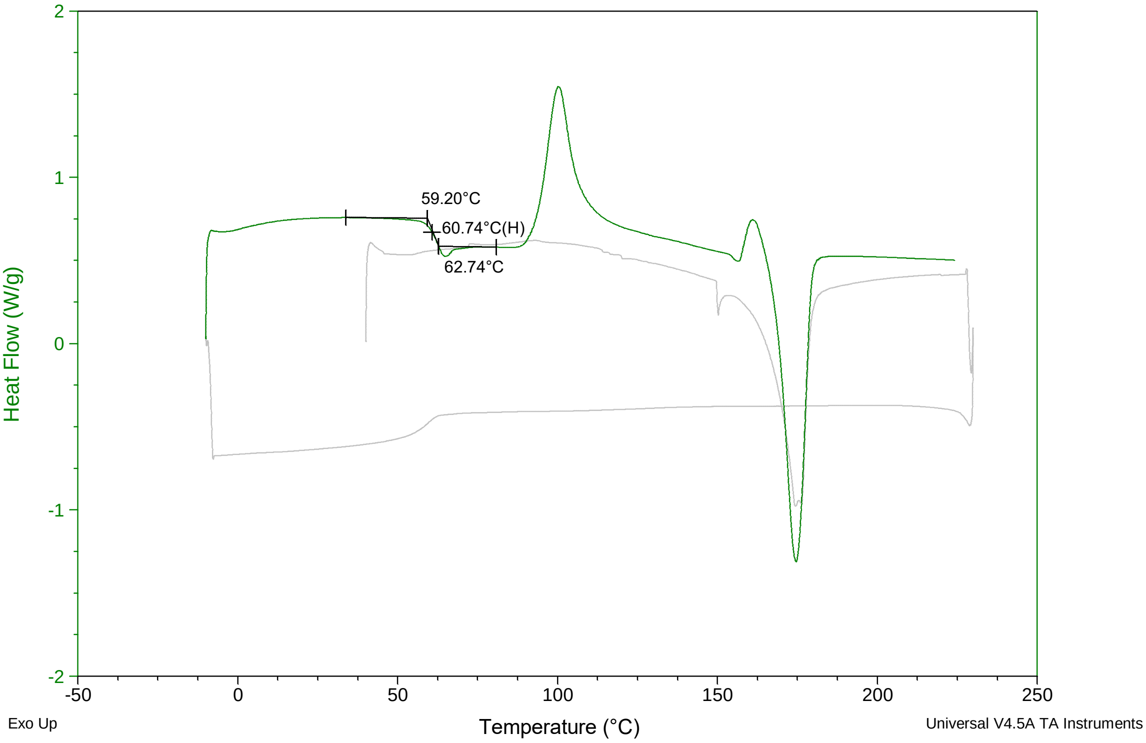

Figure S219: DSC thermogram of 6a (10 wt%) in PLA with 2nd heating cycle in green.

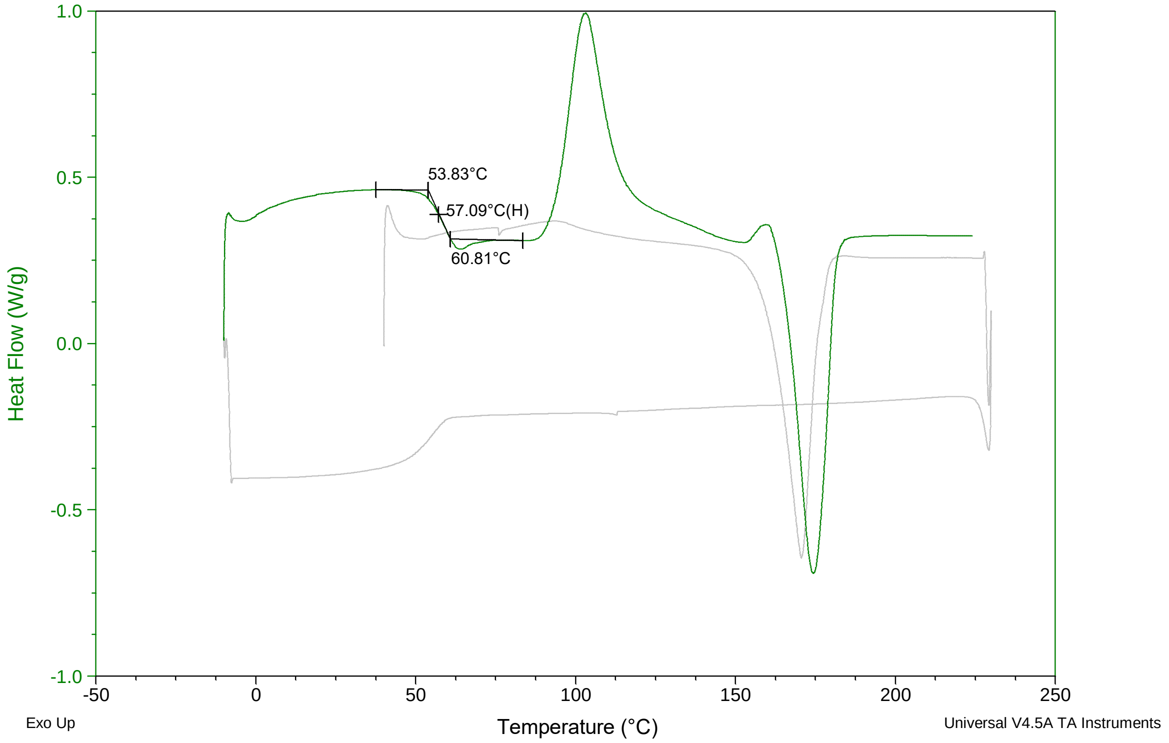

Figure S220: DSC thermogram of 7a (10 wt%) in PLA with 2nd heating cycle in green.

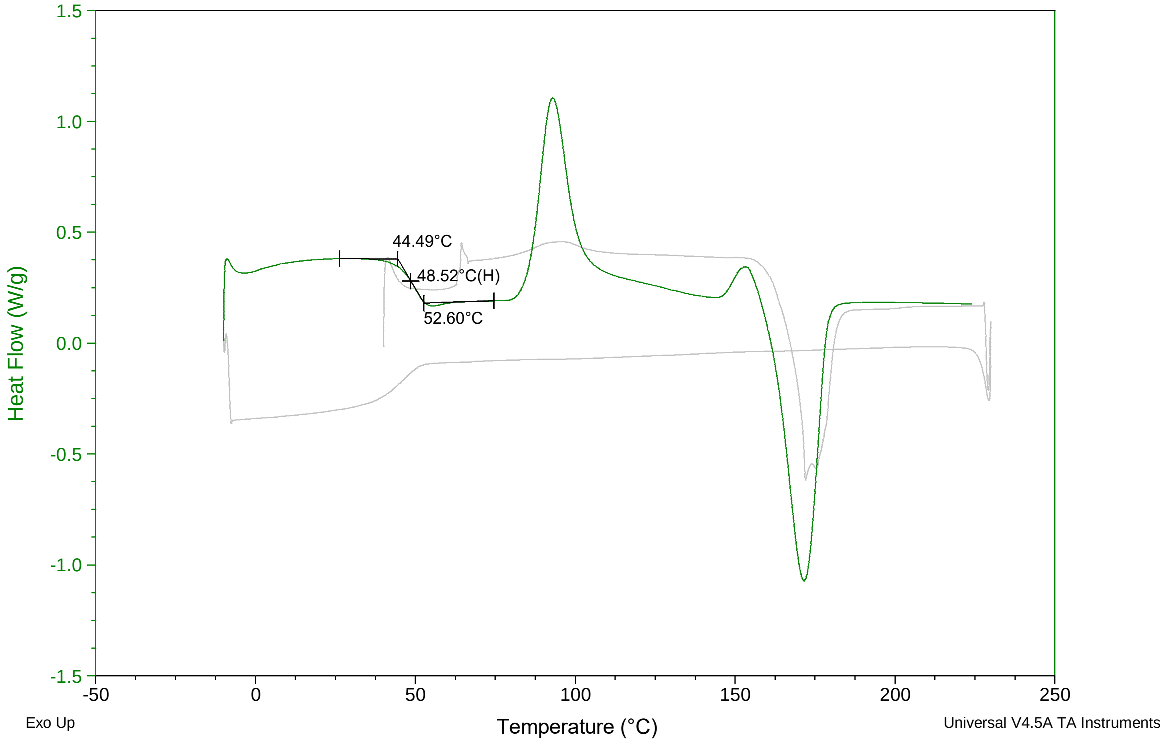

Figure S221: DSC thermogram of 8a (10 wt%) in PLA with 2nd heating cycle in green.

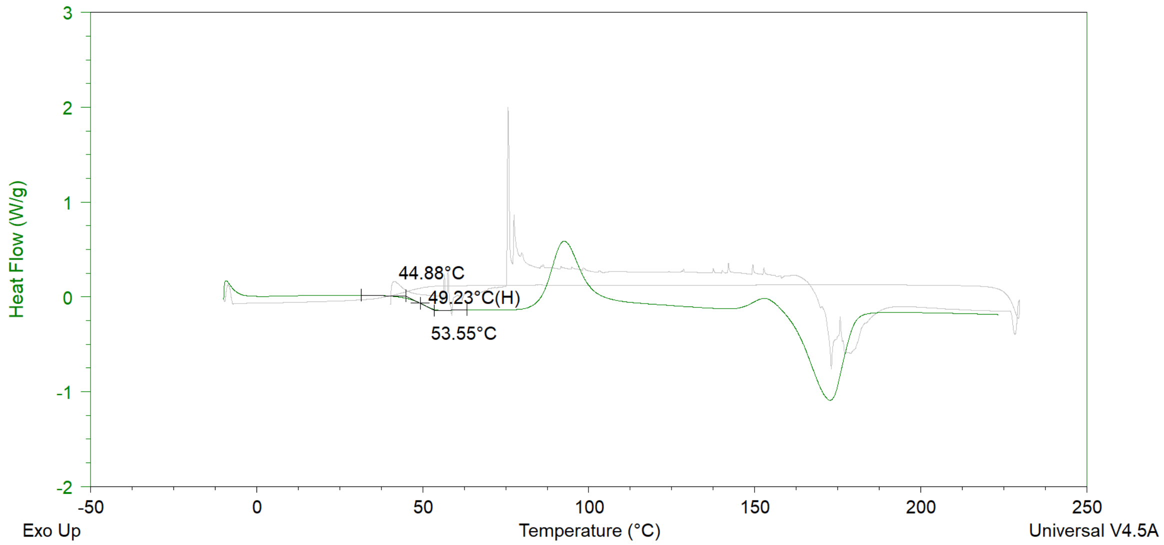

Figure S222: DSC thermogram of 8a (10 wt%) in PLA with 2nd heating cycle in green (2nd measurement).

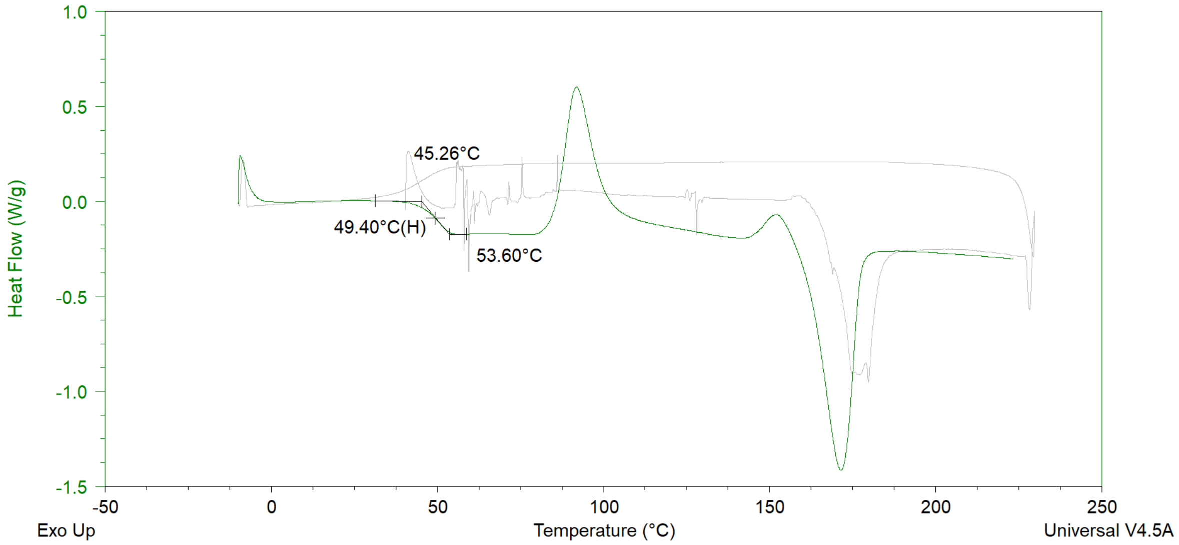

Figure S223: DSC thermogram of 8a (10 wt%) in PLA with 2nd heating cycle in green (3rd measurement).

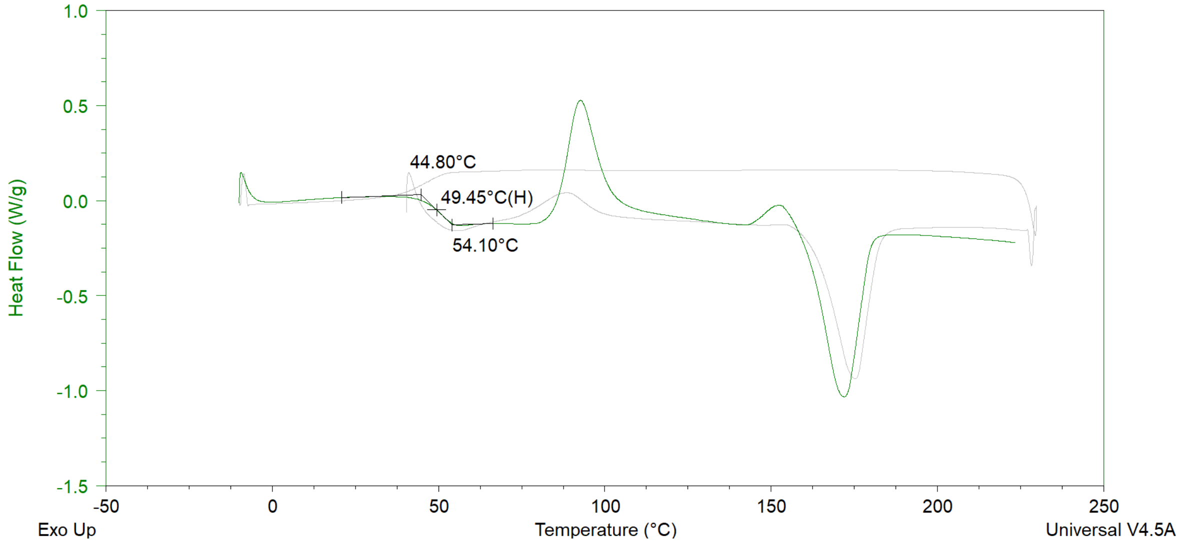

Figure S224: DSC thermogram of 8a (10 wt%) in PLA with 2nd heating cycle in green (4th measurement).

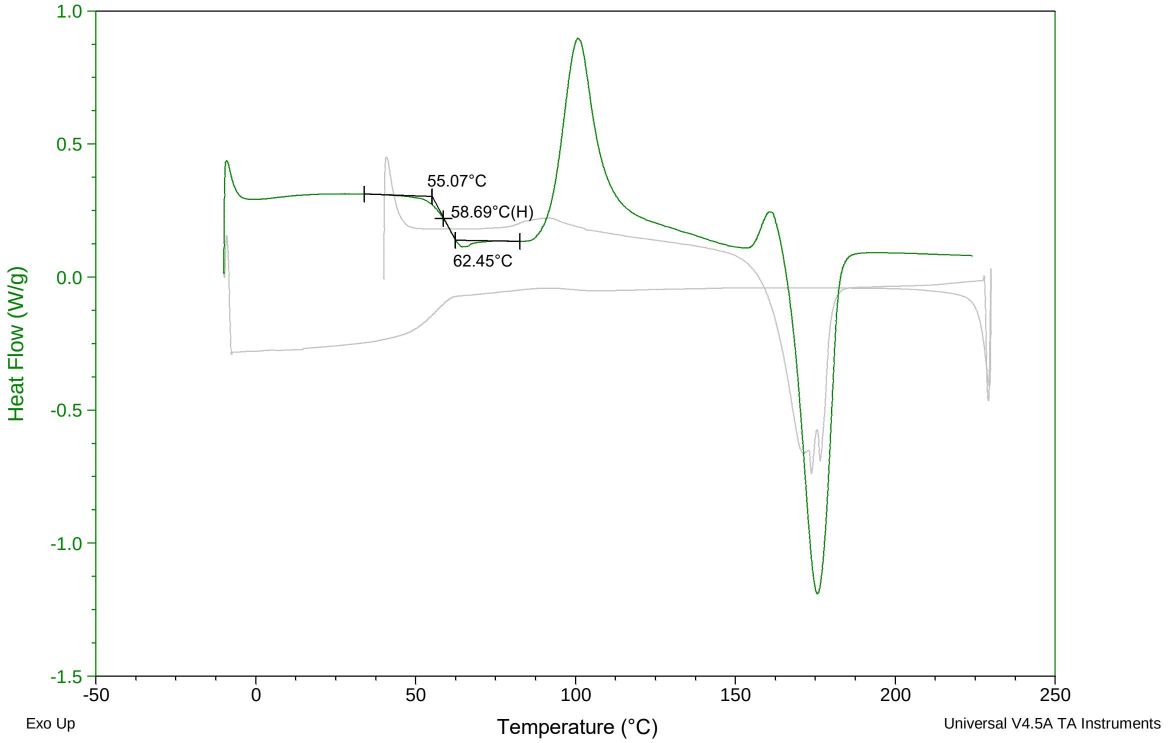

Figure S225: DSC thermogram of 9a (10 wt%) in PLA with 2nd heating cycle in green.

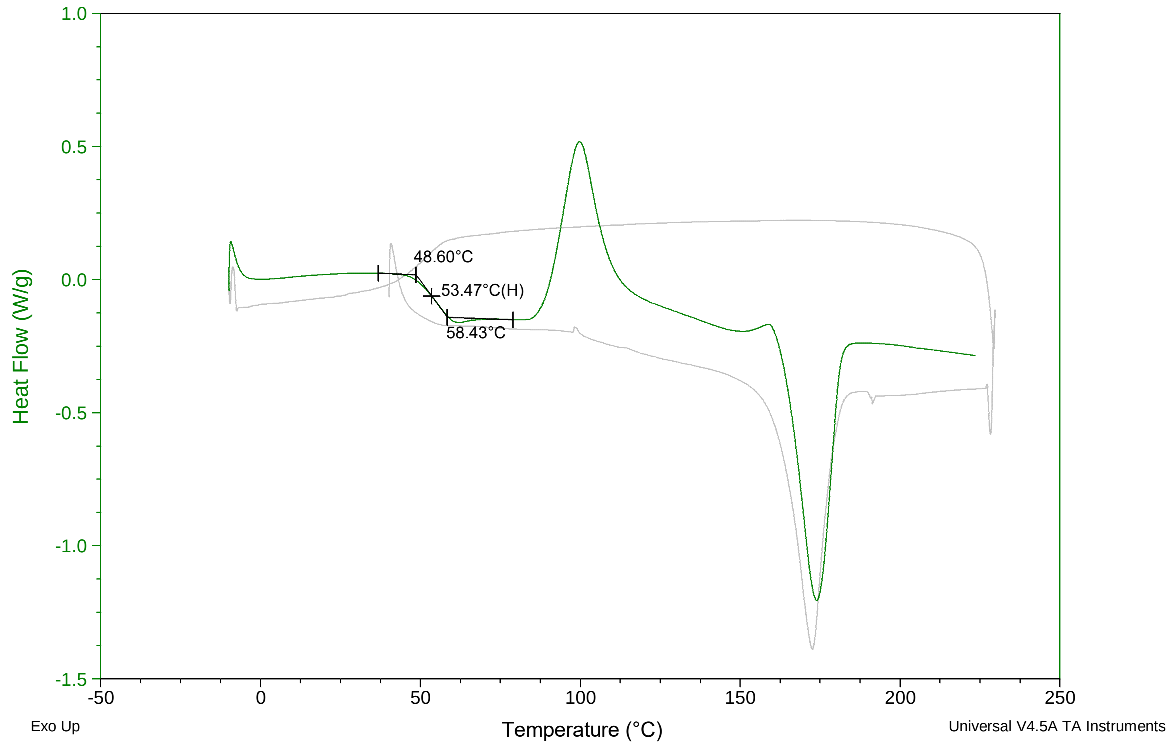

Figure S226: DSC thermogram of 10a (10 wt%) in PLA with 2nd heating cycle in green.

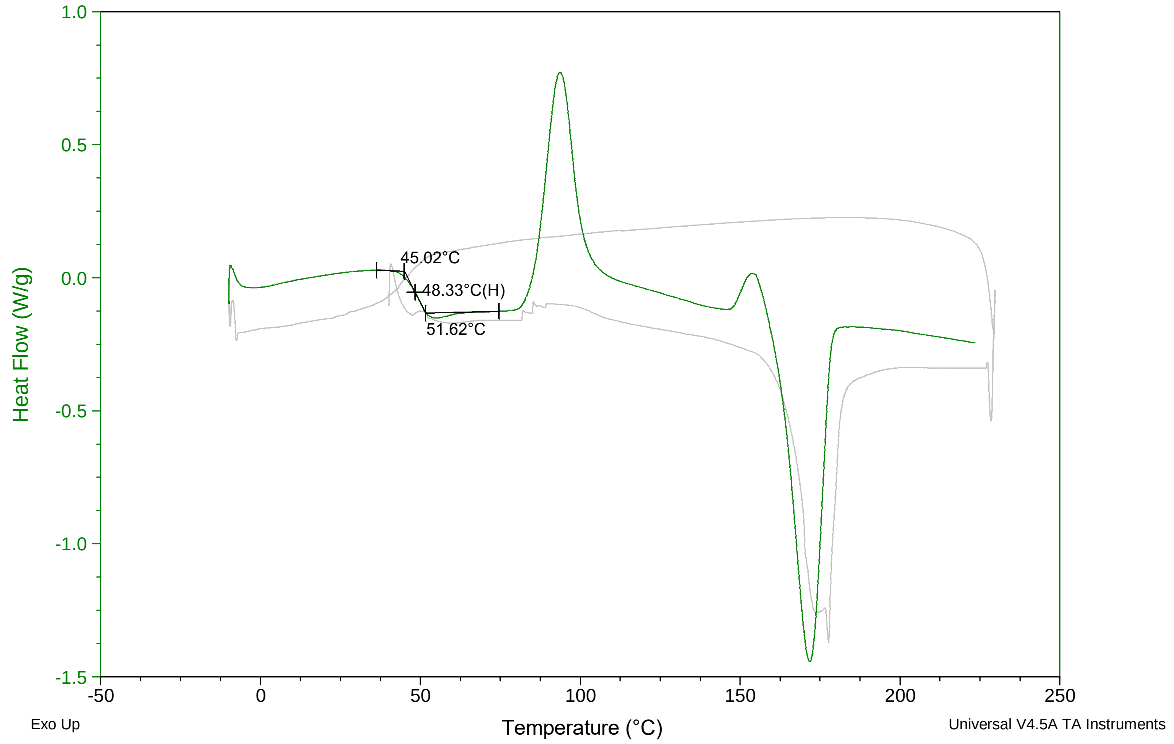

Figure S227: DSC thermogram of 11a (10 wt%) in PLA with 2nd heating cycle in green.

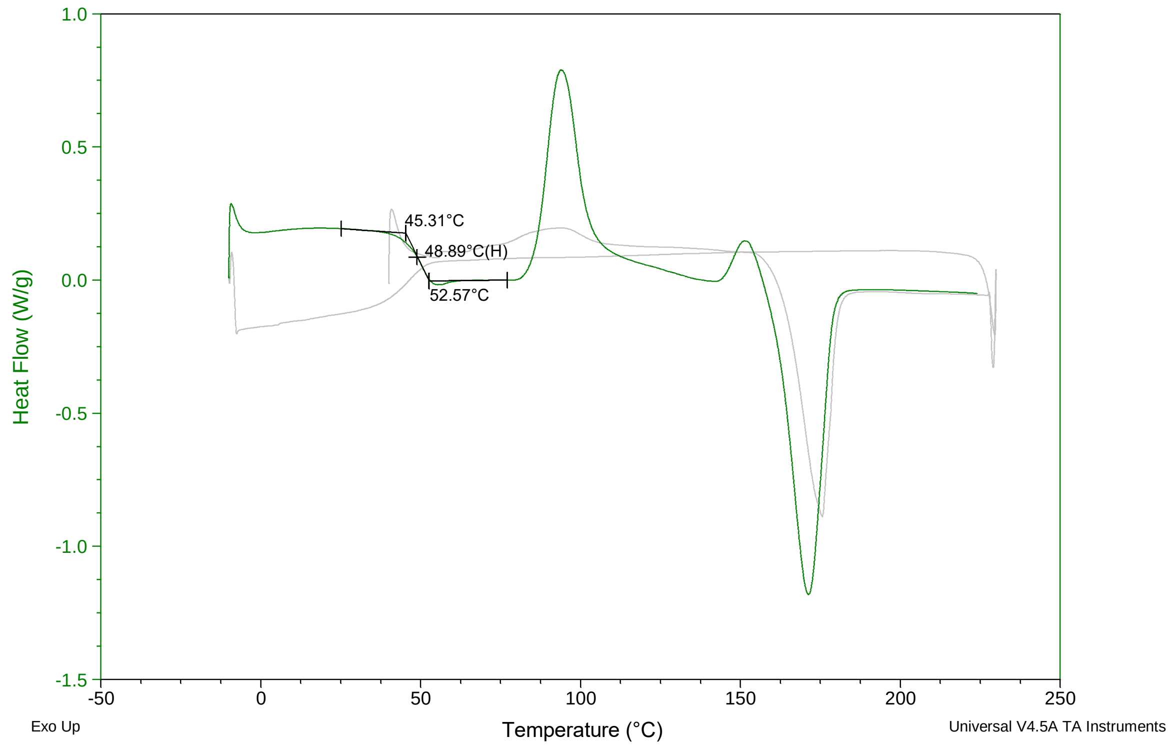

Figure S228: DSC thermogram of 8b (10 wt%) in PLA with 2nd heating cycle in green.

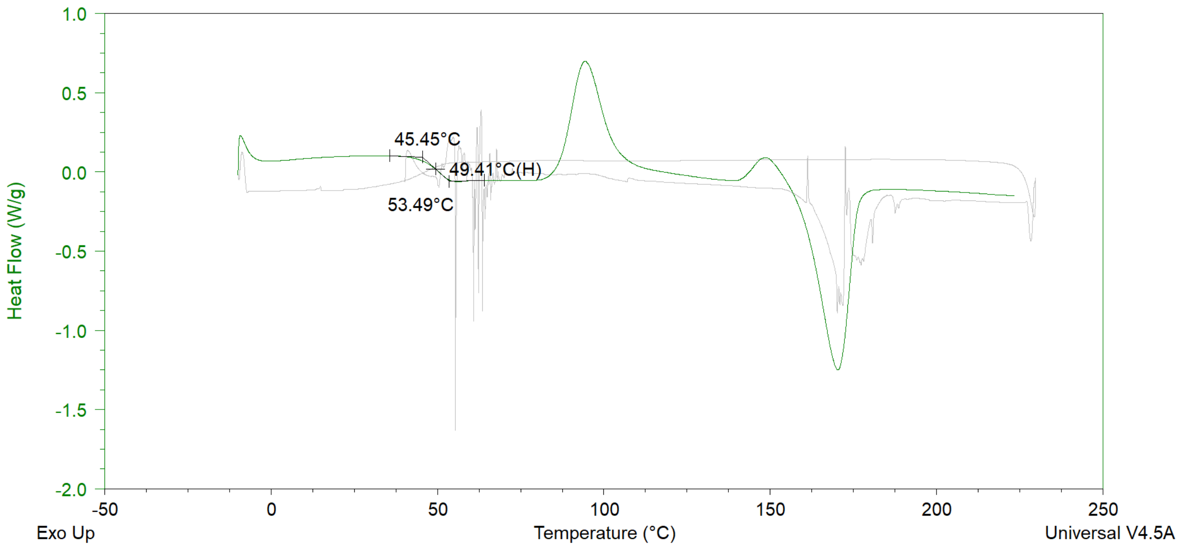

Figure S229: DSC thermogram of 8b (10 wt%) in PLA with 2nd heating cycle in green (2nd measurement).

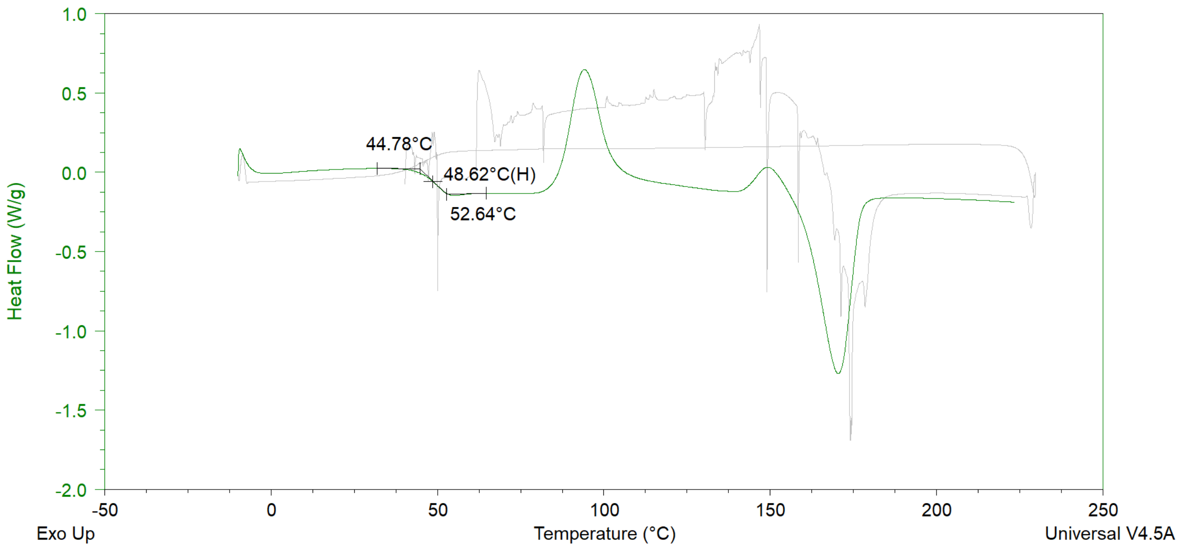

Figure S230: DSC thermogram of 8b (10 wt%) in PLA with 2nd heating cycle in green (3rd measurement).

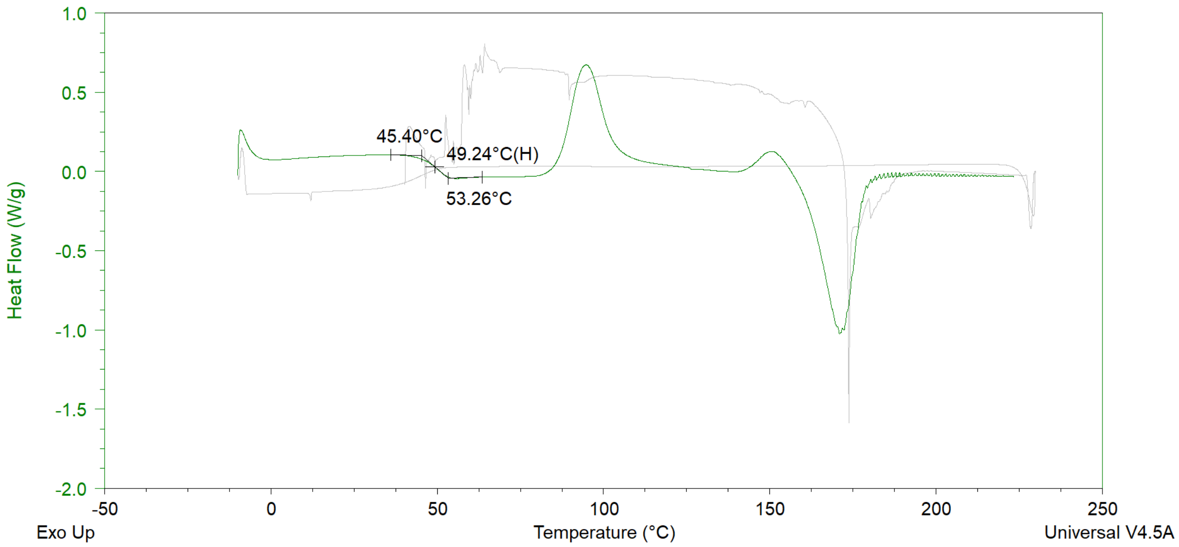

Figure S231: DSC thermogram of 8b (10 wt%) in PLA with 2nd heating cycle in green (4th measurement).

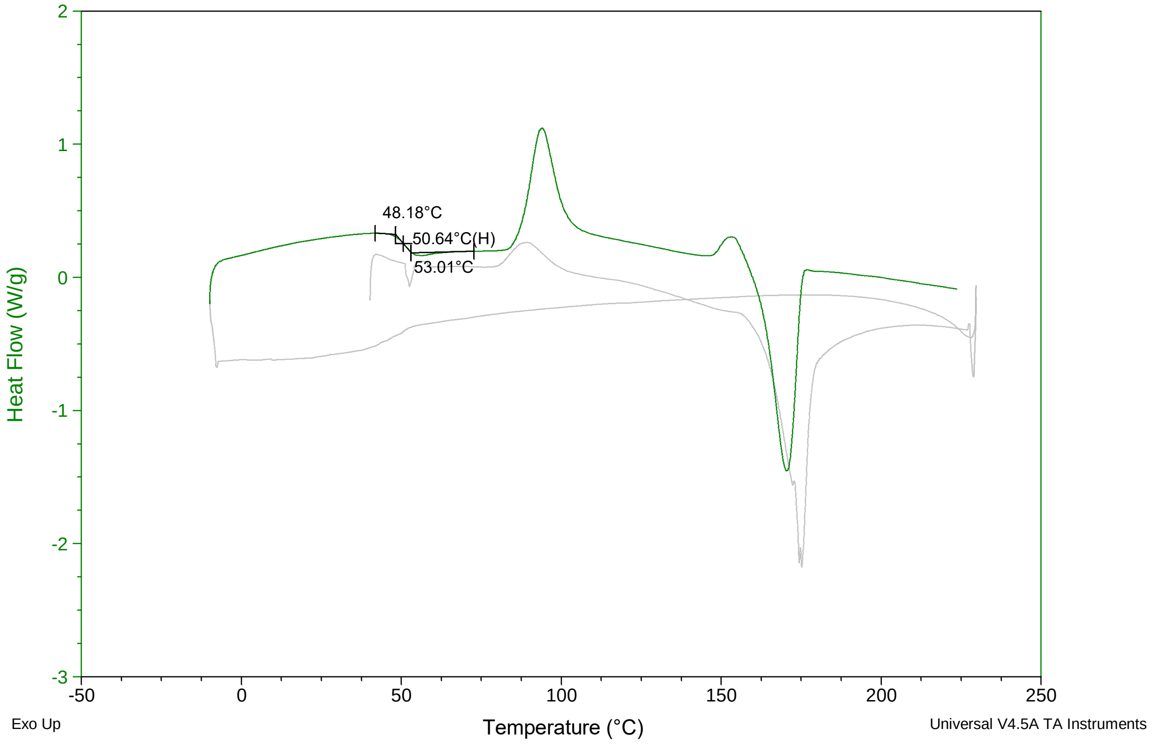

Figure S232: DSC thermogram of 8c (10 wt%) in PLA with 2nd heating cycle in green.

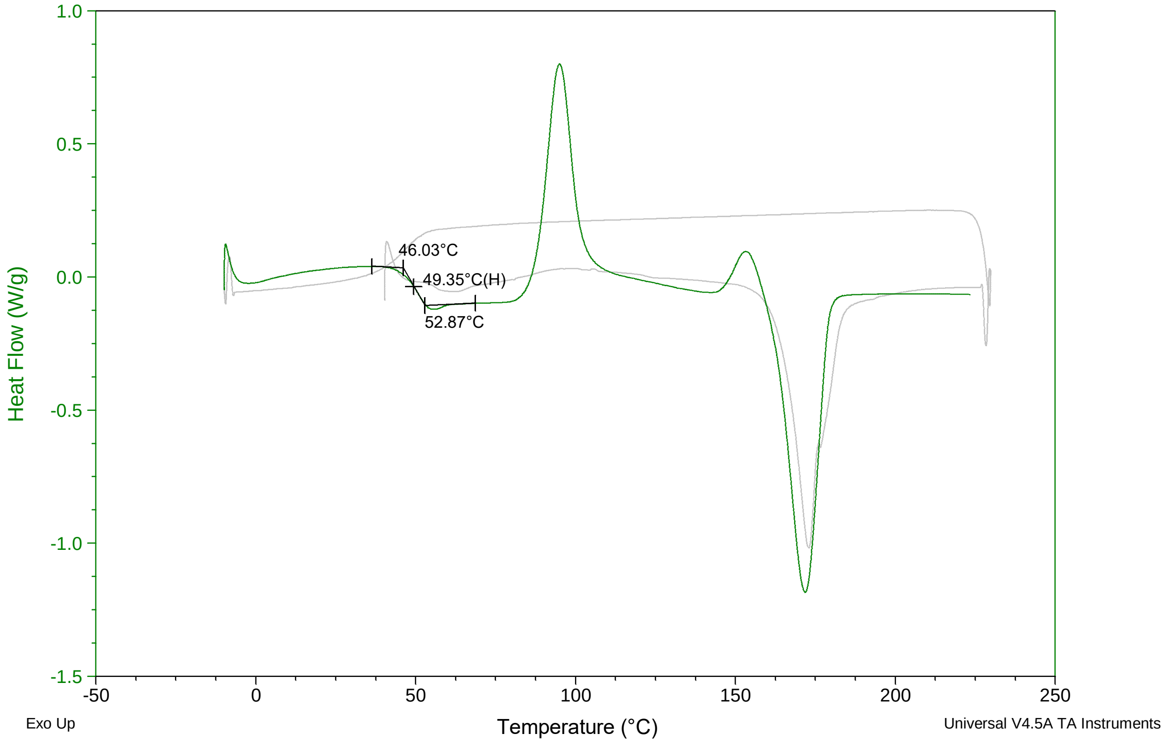

Figure S233: DSC thermogram of 8d (10 wt%) in PLA with 2nd heating cycle in green.

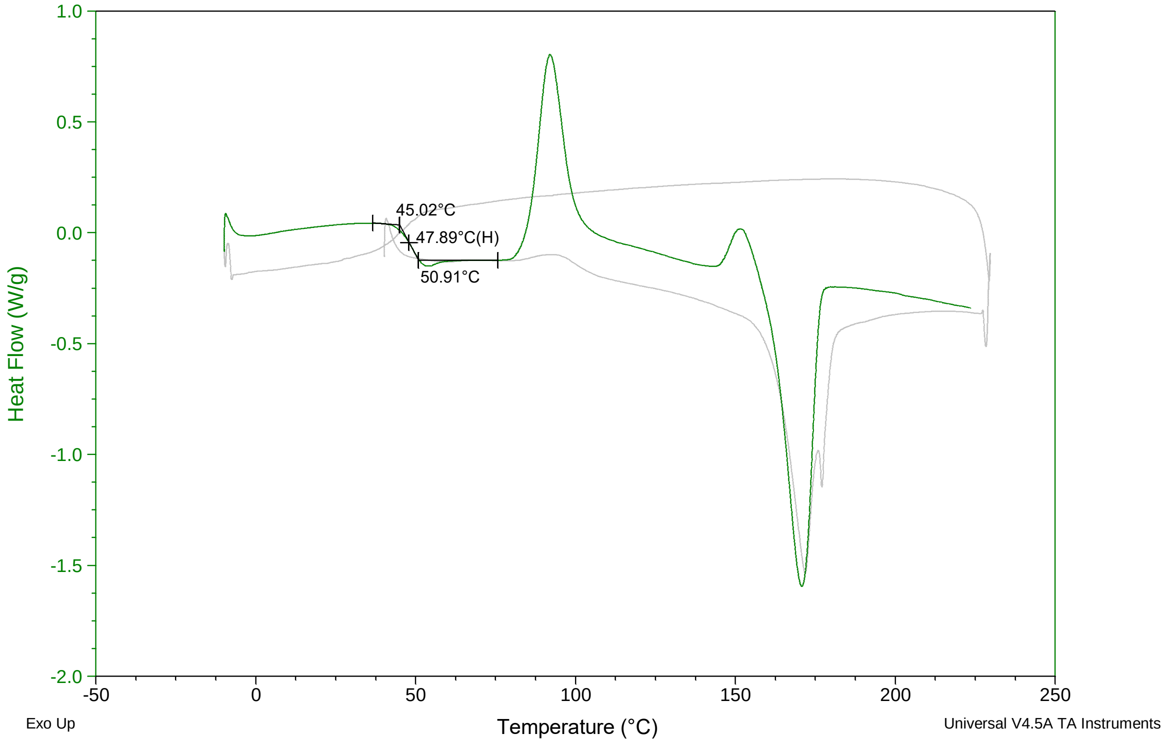

Figure S234: DSC thermogram of 11b (10 wt%) in PLA with 2nd heating cycle in green.

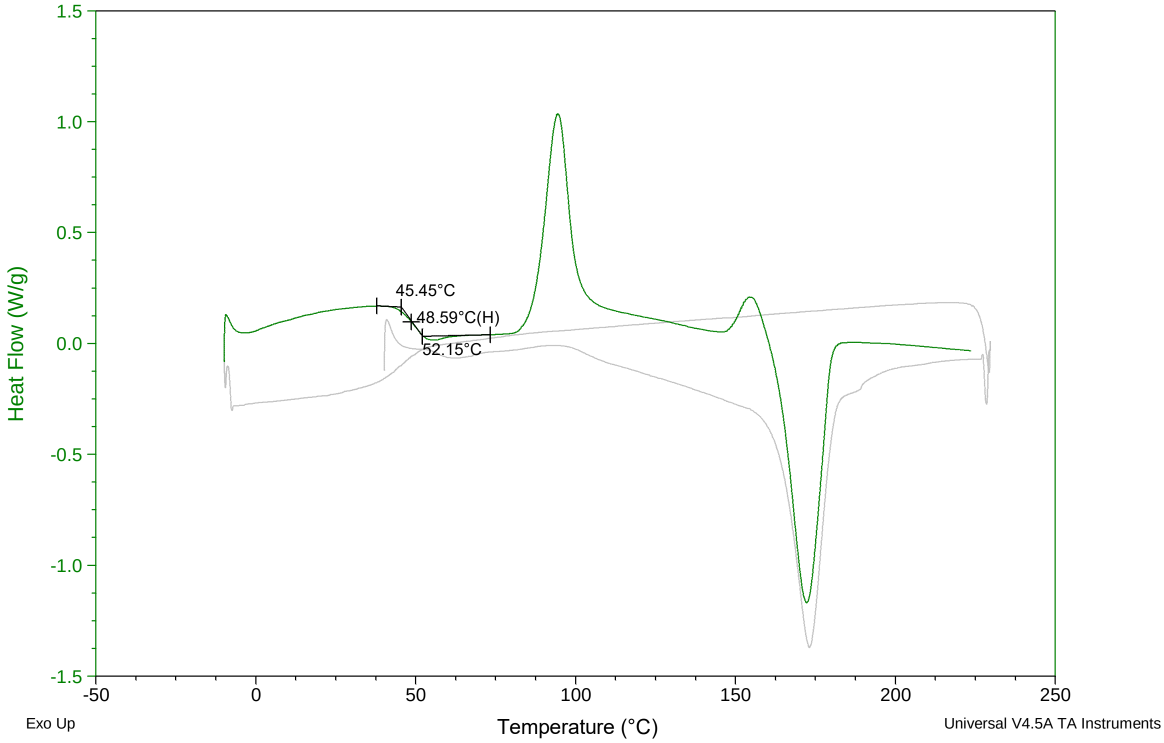

Figure S235: DSC thermogram of 11c (10 wt%) in PLA with 2nd heating cycle in green.

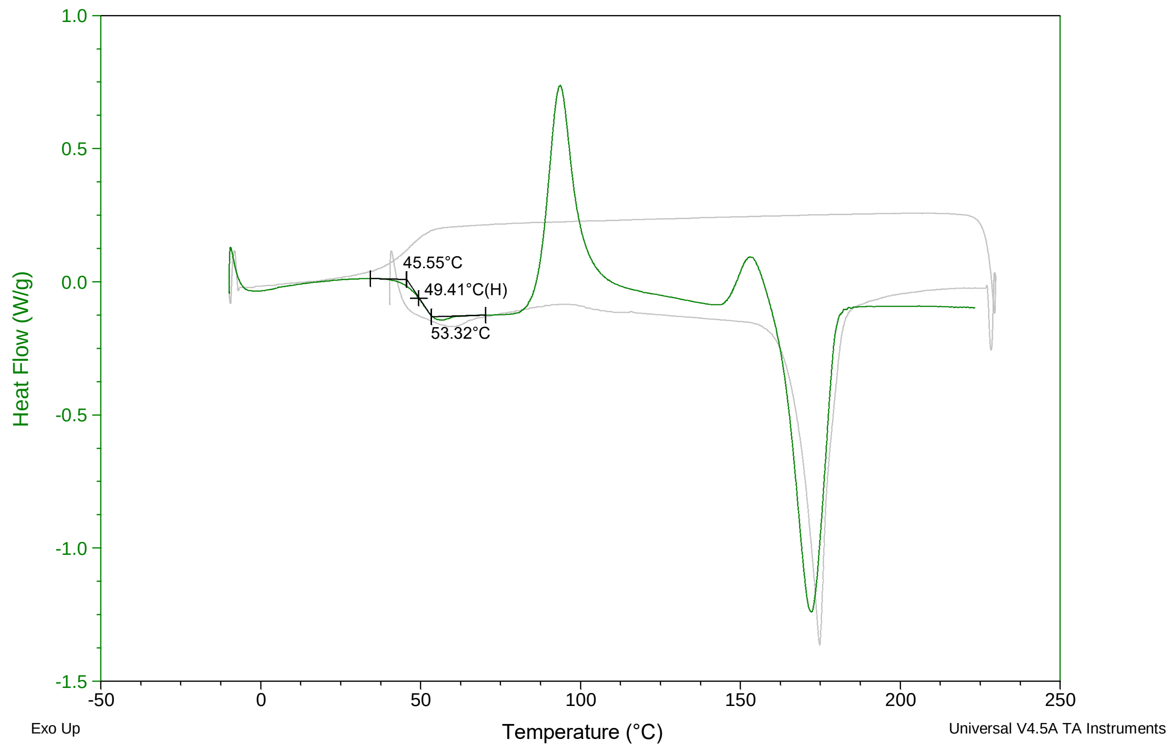

Figure S236: DSC thermogram of 11d (10 wt%) in PLA with 2nd heating cycle in green.

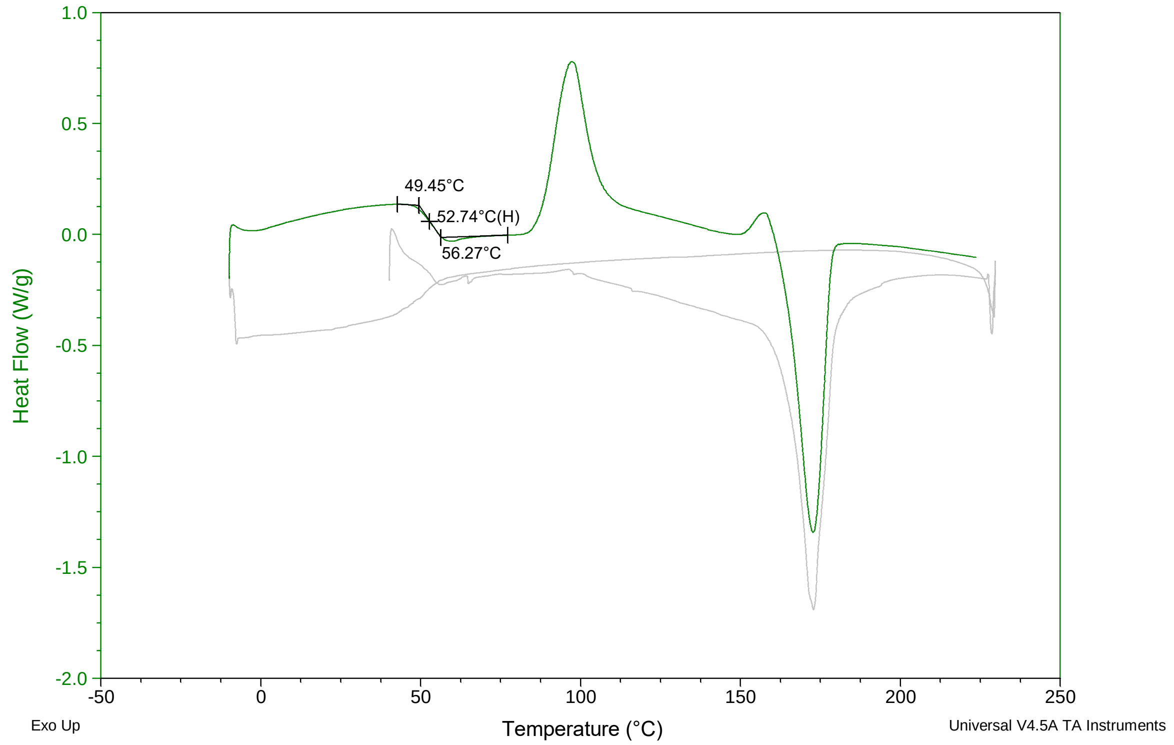

Figure S237: DSC thermogram of 12a (10 wt%) in PLA with 2nd heating cycle in green.

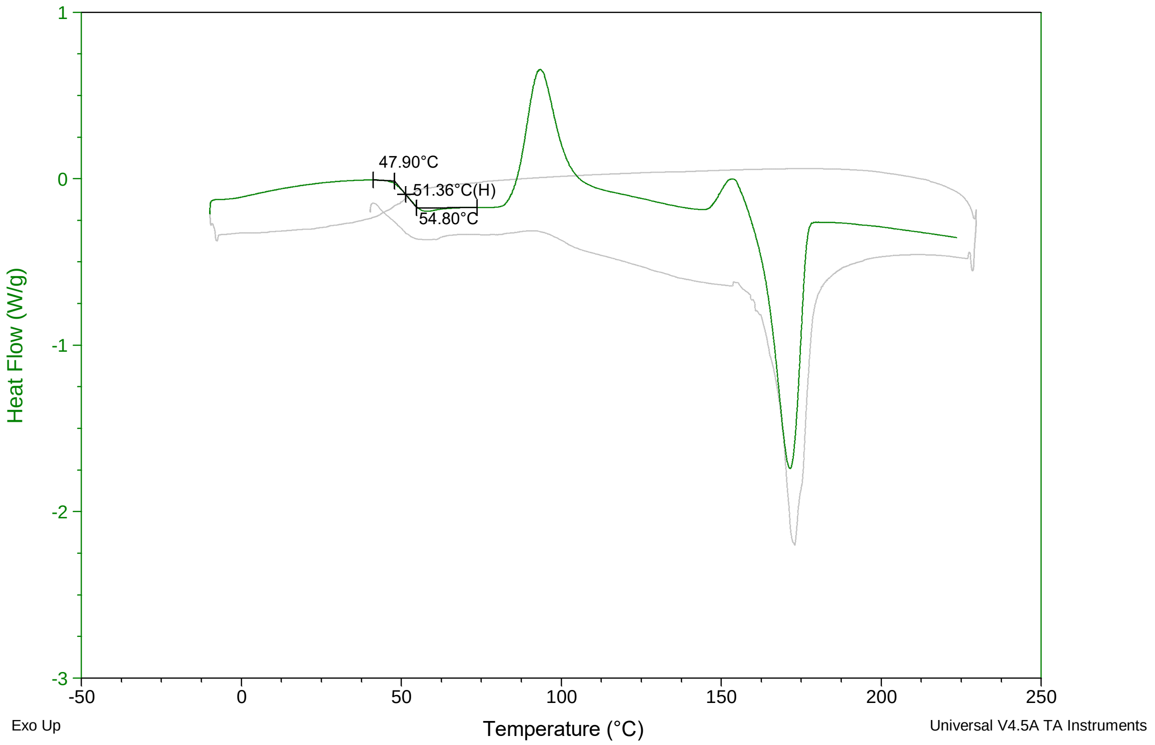

Figure S238: DSC thermogram of 12b (10 wt%) in PLA with 2nd heating cycle in green.

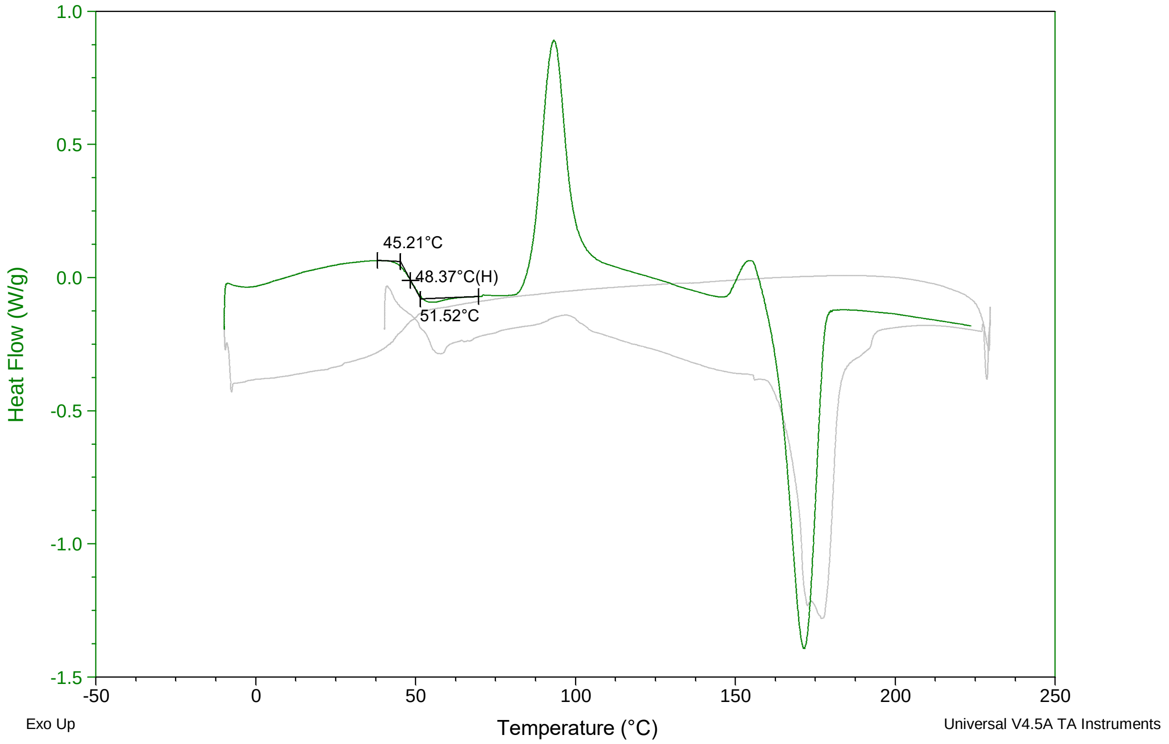

Figure S239: DSC thermogram of 12c (10 wt%) in PLA with 2nd heating cycle in green.

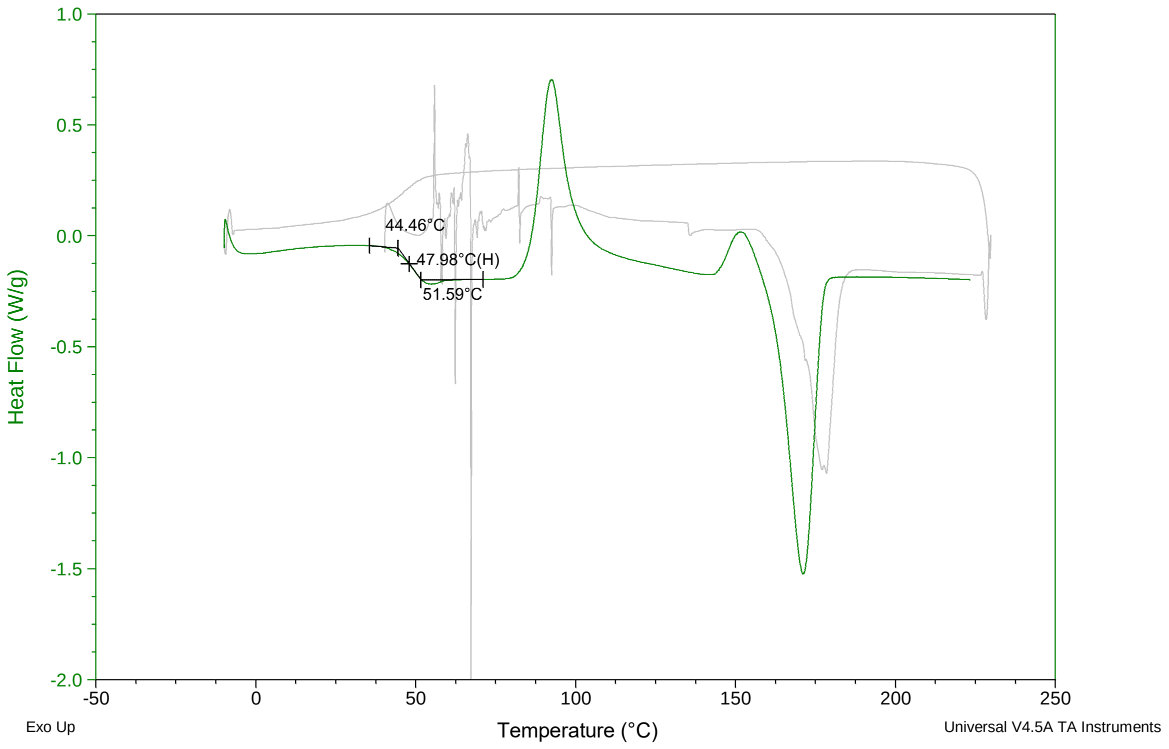

Figure S240: DSC thermogram of 12d (10 wt%) in PLA with 2nd heating cycle in green.

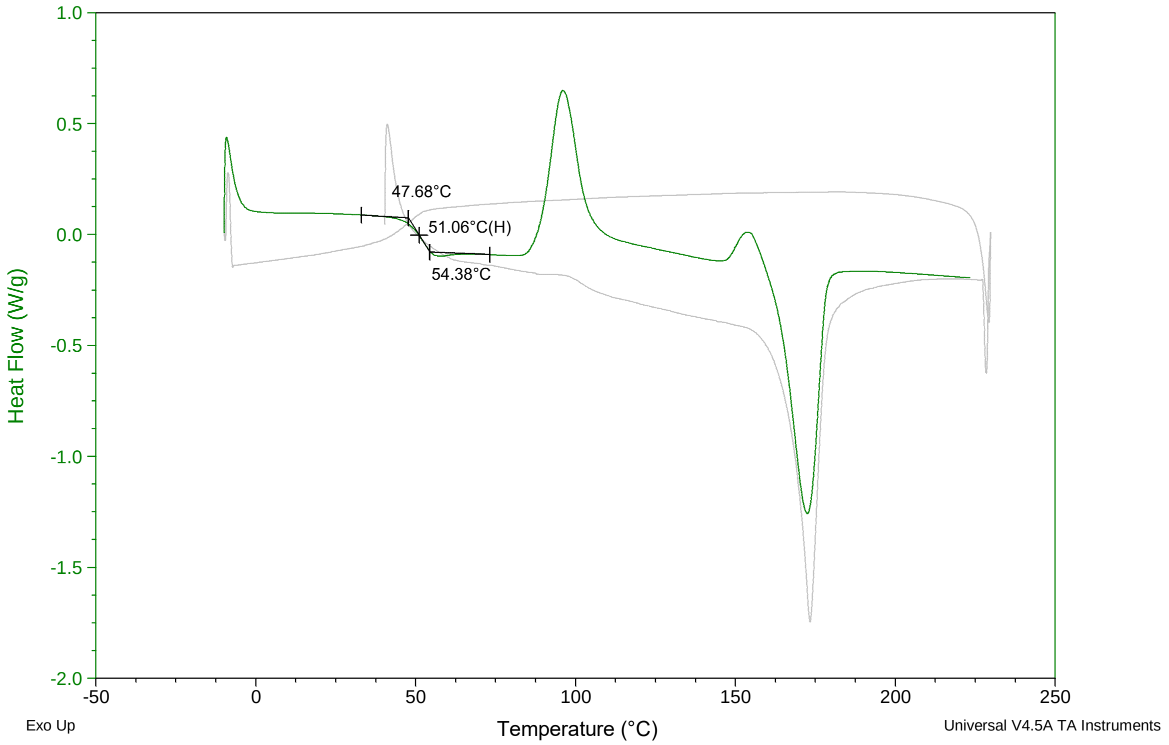

Figure S241: DSC thermogram of model mixture of 8a-8d [AGO-2024-0006 before filtration over silica] (10 wt%) in PLA with 2nd heating cycle in green.

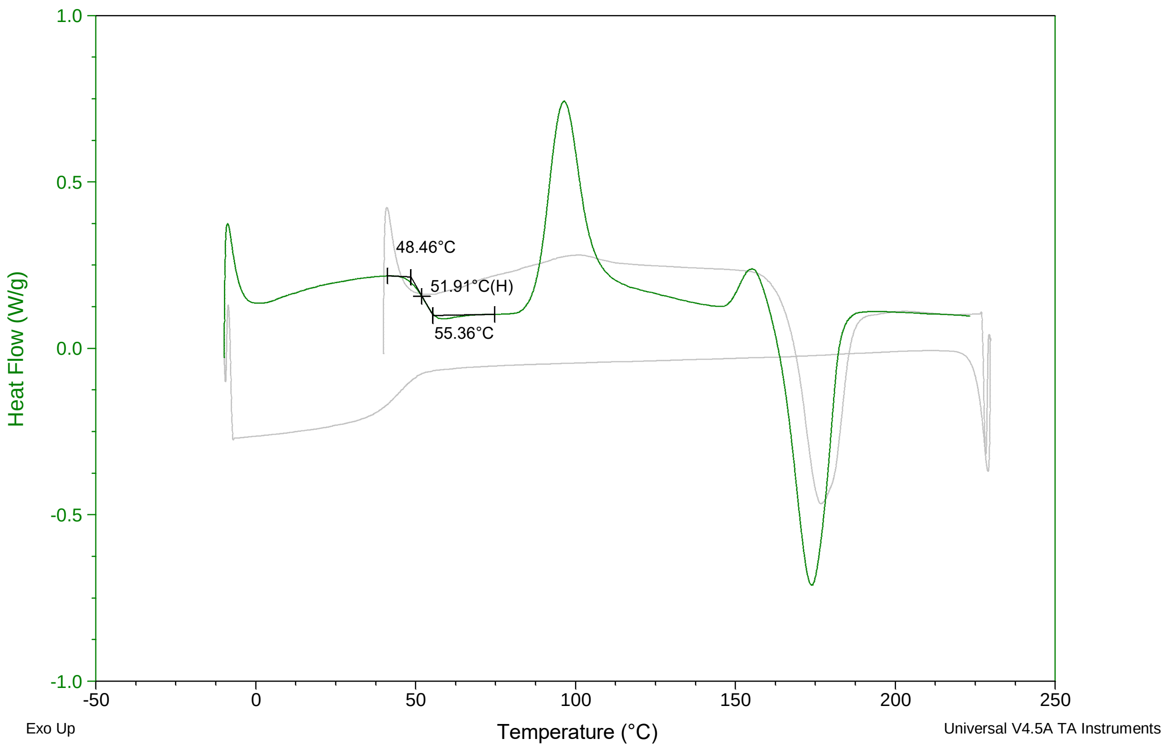

Figure S242: DSC thermogram of model mixture of 8a-8d [AGO-2024-0006 before filtration over silica] (10 wt%) in PLA with 2nd heating cycle in green (2nd measurement).

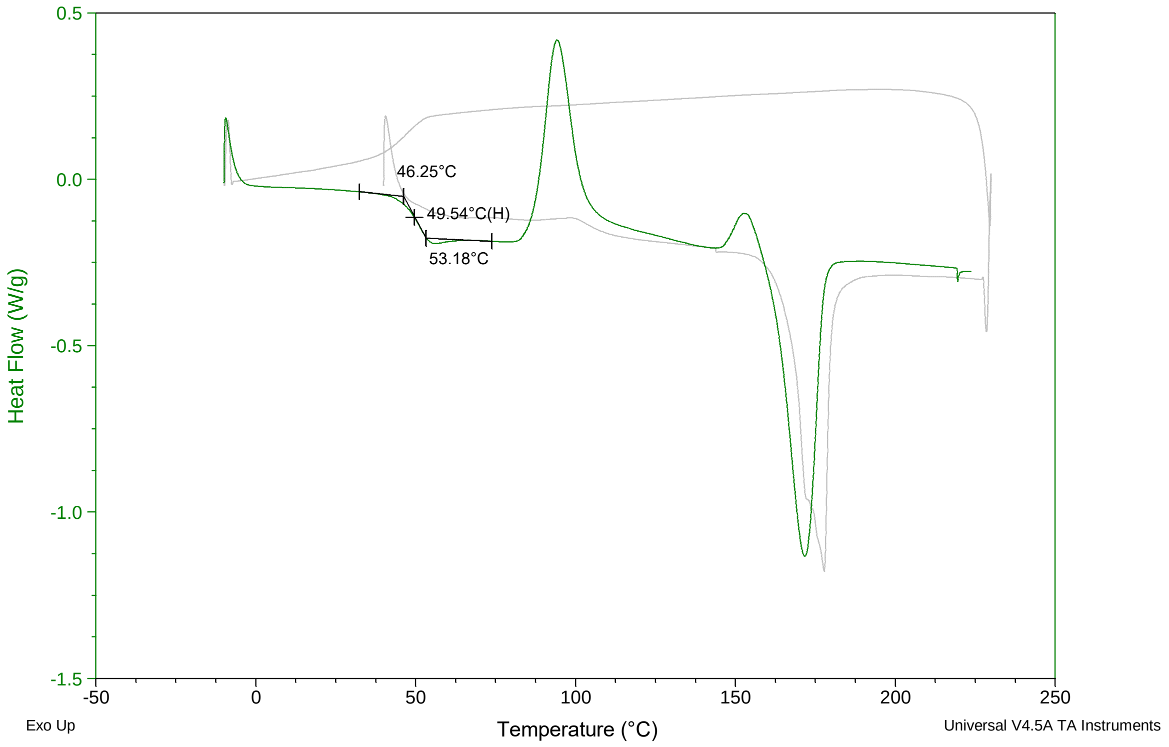

Figure S243: DSC thermogram of model mixture of 8a-8d [AGO-2024-0006 after filtration over silica] (10 wt%) in PLA with 2nd heating cycle in green

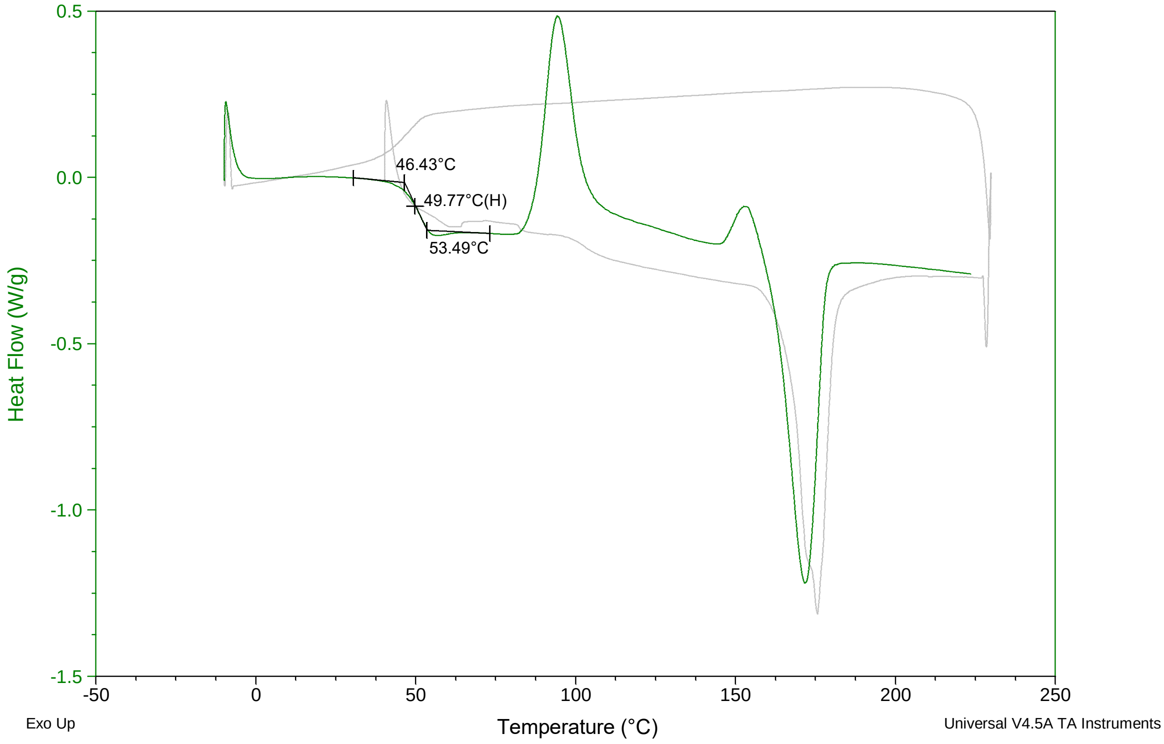

Figure S244: DSC thermogram of model mixture of 8a-8d [AGO-2024-0006 after filtration over silica] (10 wt%) in PLA with 2nd heating cycle in green (2nd measurement).

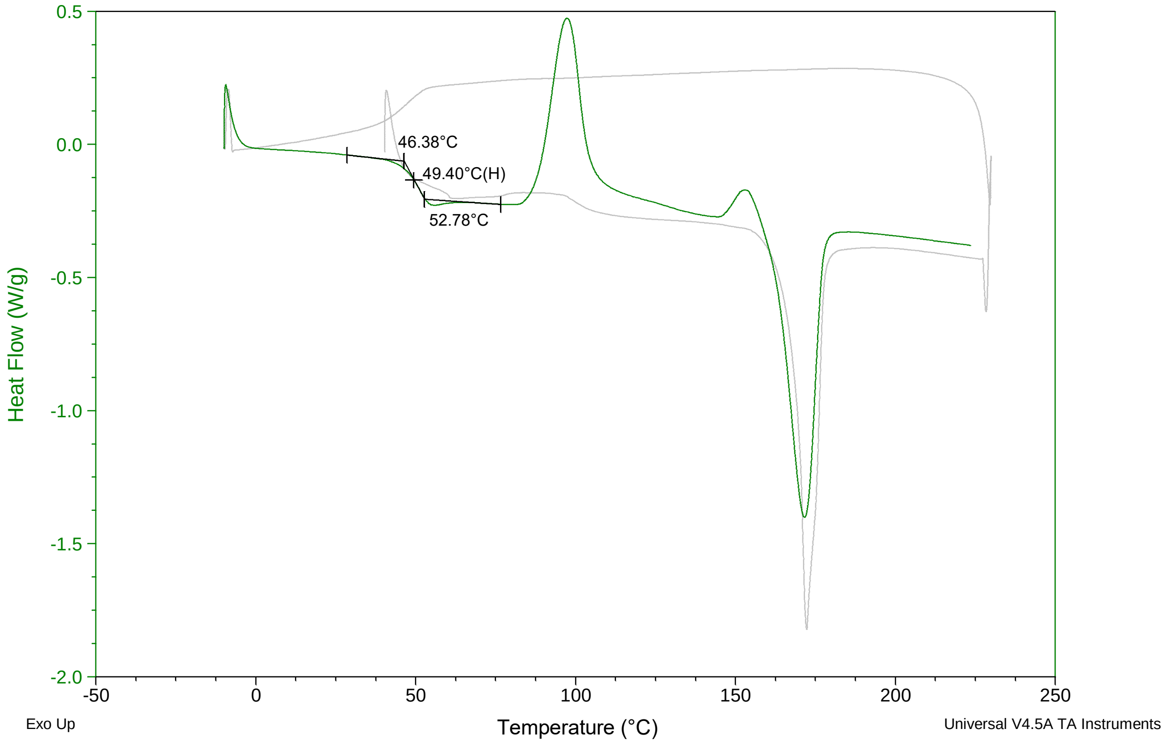

Figure S245: DSC thermogram of ball mill-derived mixture of 8a-8d [AGO-2024-0011] (10 wt%) in PLA with 2nd heating cycle in green.

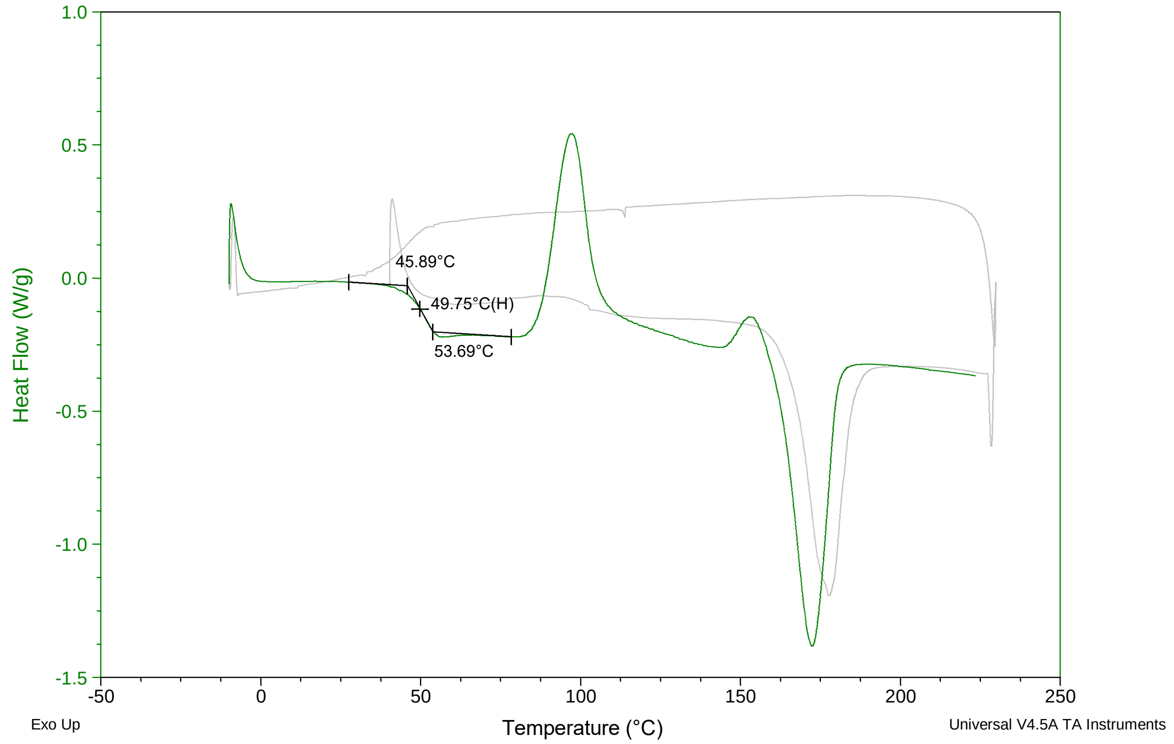

Figure S246: DSC thermogram of ball mill-derived mixture of 8a-8d [AGO-2024-0011] (10 wt%) in PLA with 2nd heating cycle in green (2nd measurement).

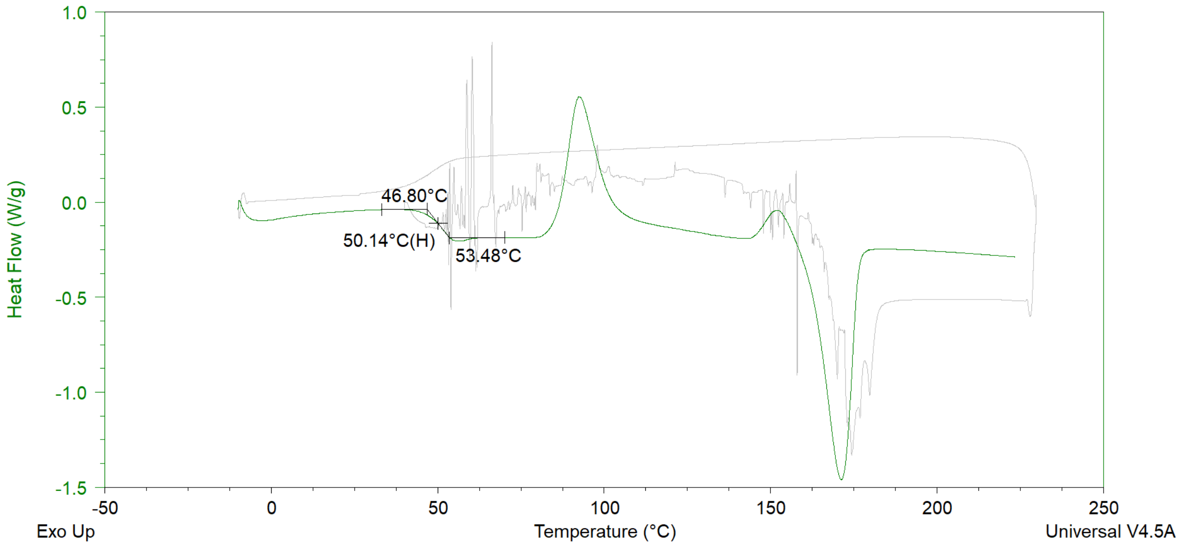

Figure S247: DSC thermogram of ball mill-derived mixture of 8a-8d [AGO-2024-0011] (10 wt%) in PLA with 2nd heating cycle in green (3rd measurement).

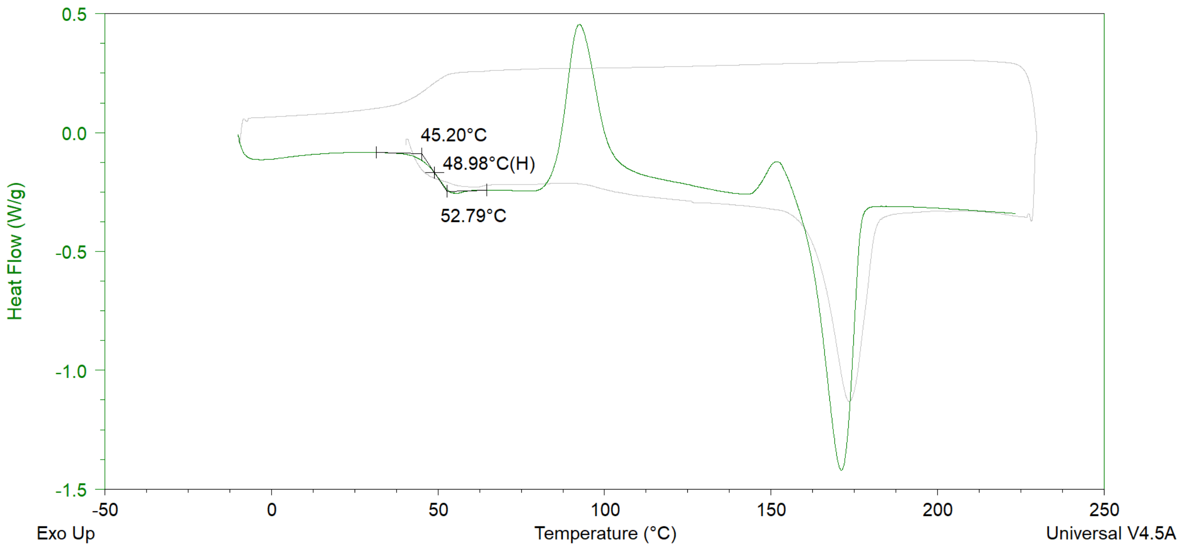

Figure S248: DSC thermogram of ball mill-derived mixture of 8a-8d [AGO-2024-0011] (10 wt%) in PLA with 2nd heating cycle in green (4th measurement).

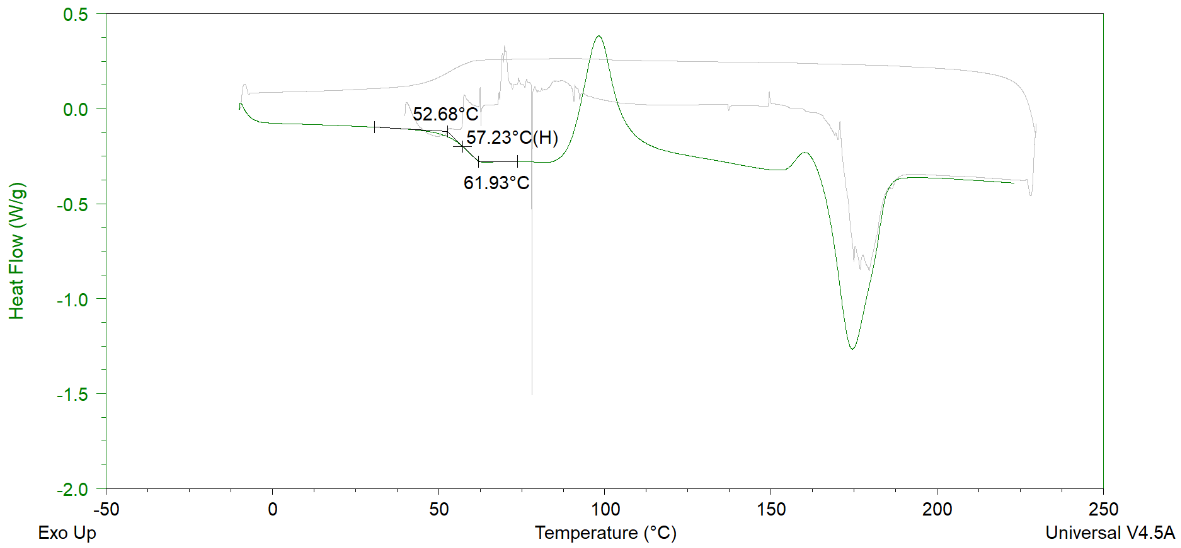

Figure S249: DSC thermogram of DEHP (5 wt%) in PLA with 2nd heating cycle in green.

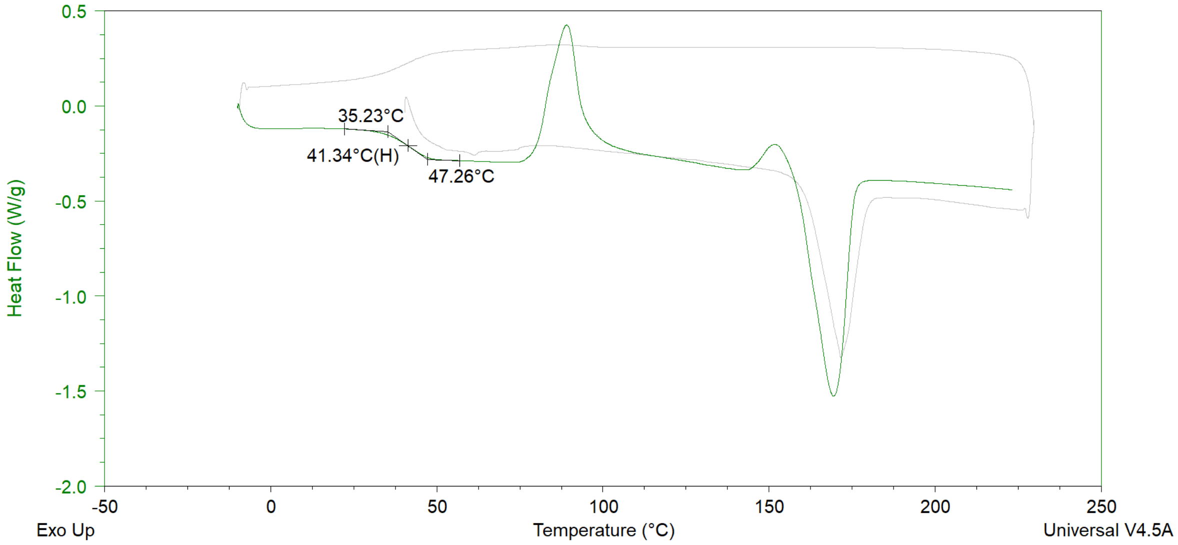

Figure S250: DSC thermogram of DEHP (20 wt%) in PLA with 2nd heating cycle in green.

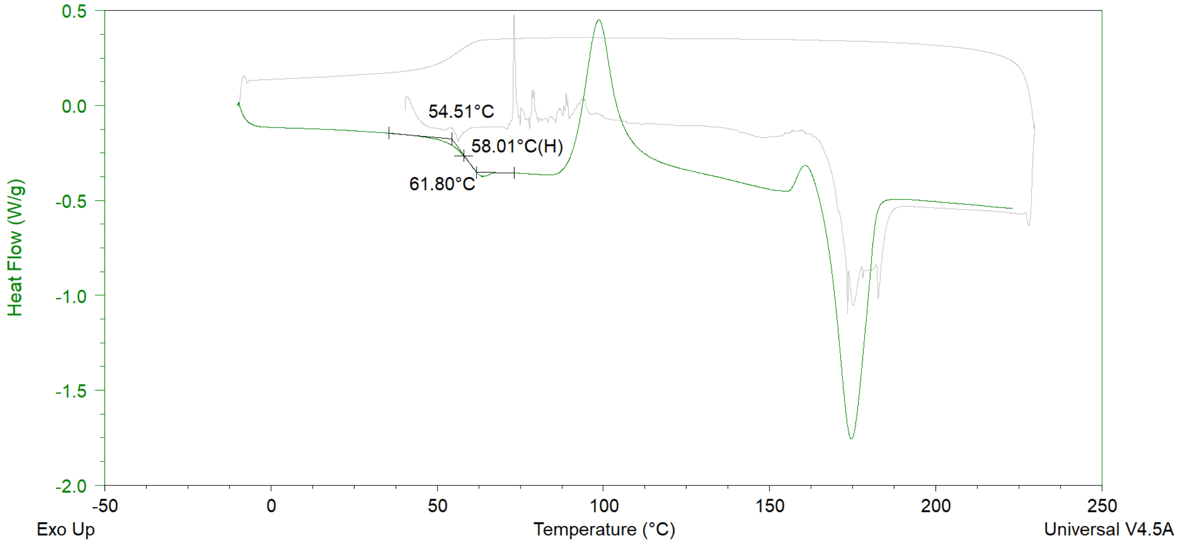

Figure S251: DSC thermogram of TBAC (5 wt%) in PLA with 2nd heating cycle in green.

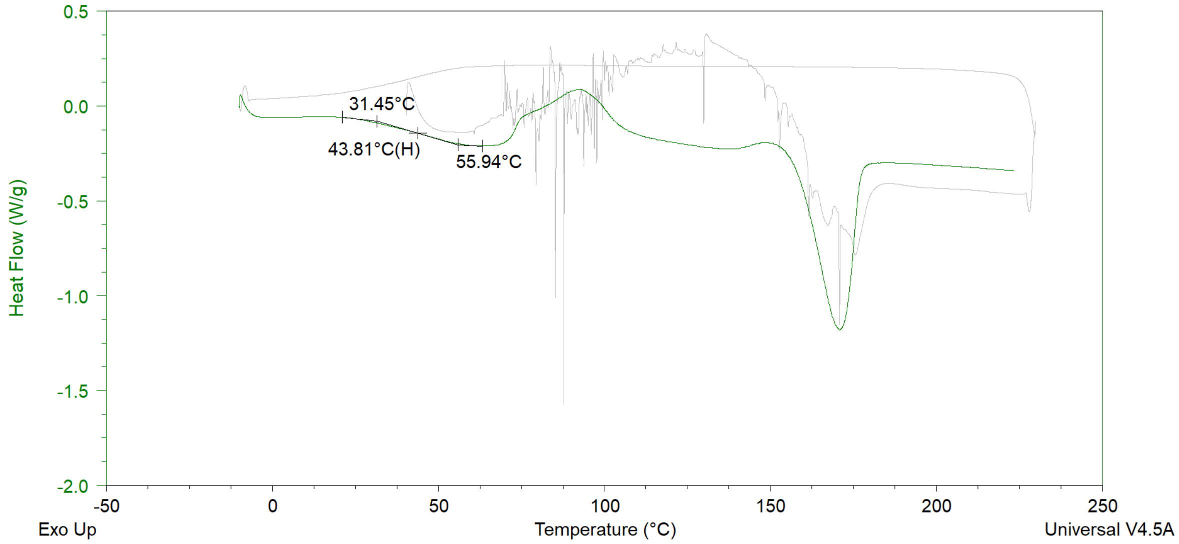

Figure S252: DSC thermogram of TBAC (20 wt%) in PLA with 2nd heating cycle in green.

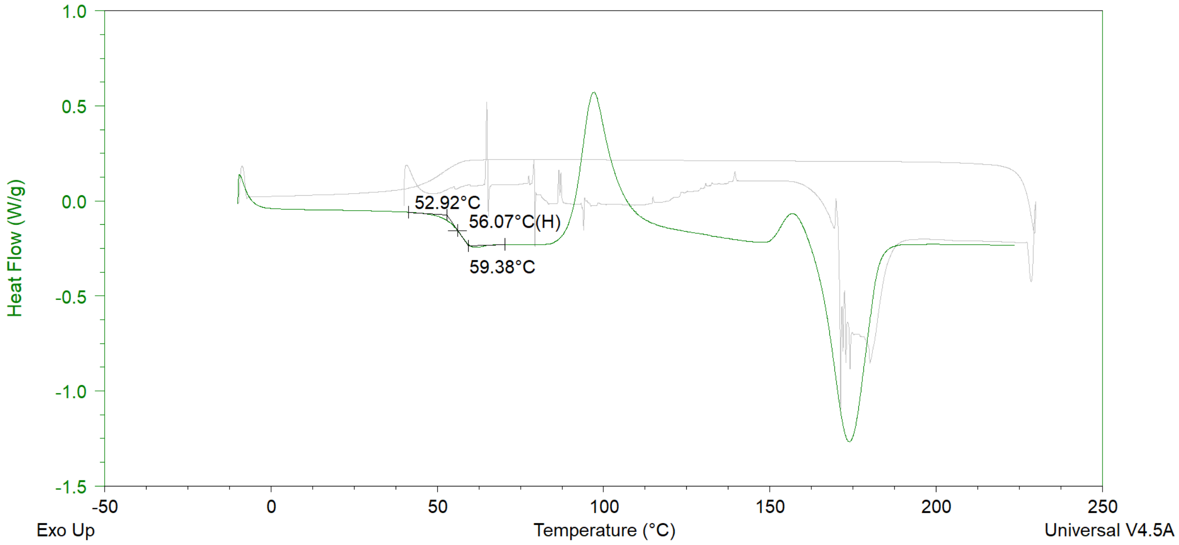

Figure S253: DSC thermogram of model mixture of 8a-8d [AGO-2024-0009 after filtration over silica] (5 wt%) in PLA with 2nd heating cycle in green.

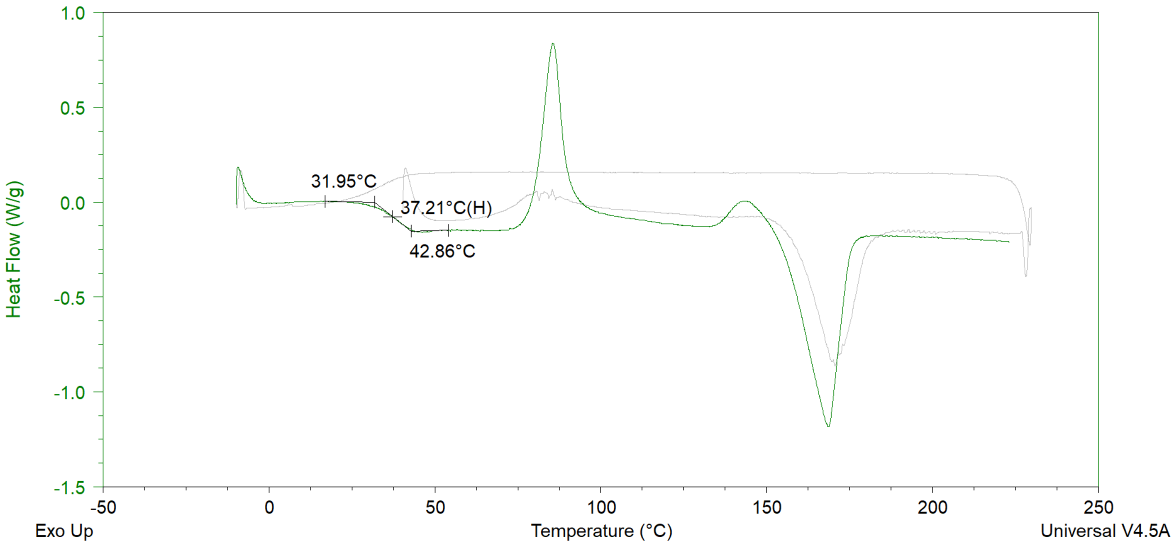

Figure S254: DSC thermogram of model mixture of 8a-8d [AGO-2024-0009 after filtration over silica] (20 wt%) in PLA with 2nd heating cycle in green.

References

[1] Mestrelab Research S.L., *MestReNova,* Version 12.0.1-20560, **2018**.

[2] a) N. R. Babij, E. O. McCusker, G. T. Whiteker, B. Canturk, N. Choy, L. C. Creemer, C. V. D. Amicis, N. M. Hewlett, P. L. Johnson, J. A. Knobelsdorf, F. Li, B. A. Lorsbach, B. M. Nugent, S. J. Ryan, M. R. Smith, Q. Yang, *Org. Process Res. Dev.* **2016**, *20*, 661-667; b) G. R. Fulmer, A. J. M. Miller, N. H. Sherden, H. E. Gottlieb, A. Nudelman, B. M. Stoltz, J. E. Bercaw, K. I. Goldberg, *Organometallics* **2010**, *29*, 2176-2179.

[3] U. Couhorn, R. Dronskowski, *Z. Anorg. Allg. Chem.* **2003**, *629*, 2554-2558.

[4] V. S. Pfennig, R. C. Villella, J. Nikodemus, C. Bolm, *Angew. Chem. Int. Ed.* **2022**, *61*, e202116514.

[5] L. Vugrin, I. Halasz, H. Geneste, *New J. Chem.* **2023**, *47*, 7466-7469.

[6] T. E. Hurst, J. A. Deichert, L. Kapeniak, R. Lee, J. Harris, P. G. Jessop, V. Snieckus, *Org. Lett.* **2019**, *21*, 3882-3885.

[7] L. C. Chetty, H. G. Kruger, P. I. Arvidsson, T. Naicker, T. Govender, *Synthesis* **2022**, *54*, 4827-4833.

[8] Y. Hu, R. Sang, R. Vroemans, G. Mollaert, R. Razzaq, H. Neumann, H. Junge, R. Franke, R. Jackstell, B. U. W. Maes, M. Beller, *Angew. Chem. Int. Ed.* **2023**, *62*, e202214706.

[9] a) X.-G. Zheng, L.-H. Tang, N. Zhang, Q.-H. Gao, C.-F. Zhang, Z.-B. Zhu, *Energy & Fuels* **2003**, *17*, 896-900; b) J. Yu, L. Sun, C. Ma, Y. Qiao, H. Yao, *Waste Manage. (Oxford)* **2016**, *48*, 300-314.

[10] P. P. R. Cruz, L. C. da Silva, R. A. Fiuza-Jr, H. Polli, *J. Appl. Polym. Sci.* **2021**, *138*, 50598.

[11] a) D.-W. Gao, Z.-D. Wen, *Sci. Total Environ.* **2016**, *541*, 986-1001; b) M. Bocqué, C. Voirin, V. Lapinte, S. Caillol, J.-J. Robin, *J. Polym. Sci., Part A: Polym. Chem.* **2016**, *54*, 11-33; c) H. Zhu, J. Yang, M. Wu, Q. Wu, J. Liu, J. Zhang, *ACS Sustainable Chem. Eng.* **2021**, *9*, 15322-15330; d) W. Xuan, K. Odelius, M. Hakkarainen, *Eur. Polym. J.* **2021**, *157*, 110649; e) C. Ver Elst, R. Vroemans, M. Bal, S. Sergeyev, C. Mensch, B. U. W. Maes, *Angew. Chem. Int. Ed.* **2023**, *62*, e202309597.

[12] R. Pinnataip, B. P. Lee, *ACS Omega* **2021**, *6*, 5113-5118.

[13] M. T. Rubino, D. Maggi, A. Laghezza, F. Loiodice, P. Tortorella, *Arch. Pharm.* **2011**, *344*, 557-563.

[14] Y. Luo, K. Sun, L. Li, L. Gao, G. Wang, Y. Qu, L. Xiang, L. Chen, Y. Hu, J. Qi, *ChemMedChem* **2011**, *6*, 1986-1989.

[15] A. Inayat, A. van Assche, J. H. Clark, T. J. Farmer, *Sustain. Chem. Pharm.* **2018**, *7*, 41-49.

[16] a) E. Budsberg, R. Morales-Vera, J. T. Crawford, R. Bura, R. Gustafson, *Biotechnol. Biofuels* **2020**, *13*, 154; b) Z. Jiang, D. Hu, Z. Zhao, Z. Yi, Z. Chen, K. Yan, *Processes* **2021**, *9*, 1234; c) Z. Yu, X. Lu, J. Xiong, N. Ji, *ChemSusChem* **2019**, *12*, 3915-3930.

[17] A. B. Lutjen, M. A. Quirk, A. M. Barbera, E. M. Kolonko, *Biorg. Med. Chem.* **2018**, *26*, 5291-5298.

[18] D. Prat, A. Wells, J. Hayler, H. Sneddon, C. R. McElroy, S. Abou-Shehada, P. J. Dunn, *Green Chem.* **2016**, *18*, 288-296.

[19] R. D. Offenhauer, *J. Chem. Educ.* **1964**, *41*, 39.

[20] A. Jakob, M. Grilc, J. Teržan, B. Likozar, *Processes* **2021**, *9*, 924.

[21] W. Chen, H. Hu, Q. Cai, S. Zhang, *Energy & Fuels* **2020**, *34*, 2238-2245.

[22] a) W. J. Elliott, J. Fried, *J. Org. Chem.* **1978**, *43*, 2708-2710; b) P. R. Jones, *Chem. Rev.* **1963**, *63*, 461-487; c) J. Cason, E. J. Reist, *J. Org. Chem.* **1958**, *23*, 1492-1496; d) M. G. Al-Shaal, W. Ciptonugroho, F. J. Holzhäuser, J. B. Mensah, P. J. C. Hausoul, R. Palkovits, *Catal. Sci. Technol.* **2015**, *5*, 5168-5173; e) X. Yi, M. G. Al-Shaal, W. Ciptonugroho, I. Delidovich, X. Wang, R. Palkovits, *ChemSusChem* **2017**, *10*, 1494-1500; f) G. H. Schmid, L. S. J. Weiler, *Can. J. Chem.* **1965**, *43*, 1242-1246; g) M. V. Bhatt, K. M. Kamath, M. Ravindranathan, *J. Chem. Soc. C: Org.* **1971**, 1772-1777.

[23] C. Beemelmanns, D. Lentz, H.-U. Reissig, *Chem. Eur. J.* **2011**, *17*, 9720-9730.

[24] J. S. Zakhari, I. Kinoyama, M. S. Hixon, A. Di Mola, D. Globisch, K. D. Janda, *Biorg. Med. Chem.* **2011**, *19*, 6203-6209.

[25] a) M. R. Charan Raja, A. B. Velappan, D. Chellappan, J. Debnath, S. Kar Mahapatra, *Eur. J. Med. Chem.* **2017**, *139*, 503-518; b) Y.-S. Bao, M. Baiyin, B. Agula, M. Jia, B. Zhaorigetu, *J. Org. Chem.* **2014**, *79*, 6715-6719.

[26] M. Nguyen, B. Meunier, A. Robert, *Eur. J. Inorg. Chem.* **2017**, 3198-3204.

[27] S. Sato, D. Gondo, T. Wada, S. Kanehashi, K. Nagai, *J. Appl. Polym. Sci.* **2013**, *129*, 1607-1617.

[28] a) T. Ohe, R. Umezawa, Y. Kitagawara, D. Yasuda, K. Takahashi, S. Nakamura, A. Abe, S. Sekine, K. Ito, K. Okunushi, H. Morio, T. Furihata, N. Anzai, T. Mashino, *Bioorg. Med. Chem. Lett.* **2018**, *28*, 3708-3711; b) C. Glas, E. Naydenova, S. Lechner, N. Wössner, L. Yang, J. C. B. Dietschreit, H. Sun, M. Jung, B. Kuster, C. Ochsenfeld, F. Bracher, *Eur. J. Med. Chem.* **2022**, *240*, 114594.

[29] D. Talukdar, S. Panda, R. Borah, D. Manna, *J. Phys. Chem. B* **2014**, *118*, 7541-7553.

[30] a) J. Beyer, S. Lang-Fugmann, A. Mühlbauer, W. Steglich, *Synthesis* **1998**, 1047-1051; b) J. M. Khurana, S. Chauhan, G. Bansal, *Monatsh. Chem.* **2004**, *135*, 83-87.

[31] A. Endo, N. Kitahara, H. Oka, Y. Miguchi-Fukazawa, A. Terahara, *Eur. J. Biochem.* **1978**, *82*, 257-259.
